# Proteotyping of knockout mouse strains reveals sex- and strain-specific signatures in blood plasma

**DOI:** 10.1038/s41540-021-00184-8

**Published:** 2021-05-28

**Authors:** Yassene Mohammed, Sarah A. Michaud, Helena Pětrošová, Juncong Yang, Milan Ganguly, David Schibli, Ann M. Flenniken, Lauryl M. J. Nutter, Hibret A. Adissu, K. C. Kent Lloyd, Colin McKerlie, Christoph H. Borchers

**Affiliations:** 1 University of Victoria—Genome BC Proteomics Centre, Victoria, BC Canada; 2 Center for Proteomics and Metabolomics, Leiden University Medical Center, Leiden, Netherlands; 3 The Center for Phenogenomics, Toronto, ON Canada; 4 The Hospital for Sick Children, Toronto, ON Canada; 5 Sinai Health Lunenfeld-Tanenbaum Research Institute, Toronto, ON Canada; 6 Covance Inc., Chantilly, VA USA; 7 Department of Surgery, School of Medicine, and Mouse Biology Program, University of California, Davis, CA USA; 8 Proteomics Centre, Segal Cancer Centre, Lady Davis Institute, Jewish General Hospital, McGill University, Montreal, QC Canada; 9 Gerald Bronfman Department of Oncology, Jewish General Hospital, Montreal, QC Canada; 10 Department of Data Intensive Science and Engineering, Skolkovo Institute of Science and Technology, Skolkovo Innovation Center, Moscow, Russia

**Keywords:** Biochemistry, Molecular biology, Systems biology, Genetics

## Abstract

We proteotyped blood plasma from 30 mouse knockout strains and corresponding wild-type mice from the International Mouse Phenotyping Consortium. We used targeted proteomics with internal standards to quantify 375 proteins in 218 samples. Our results provide insights into the manifested effects of each gene knockout at the plasma proteome level. We first investigated possible contamination by erythrocytes during sample preparation and labeled, in one case, up to 11 differential proteins as erythrocyte originated. Second, we showed that differences in baseline protein abundance between female and male mice were evident in all mice, emphasizing the necessity to include both sexes in basic research, target discovery, and preclinical effect and safety studies. Next, we identified the protein signature of each gene knockout and performed functional analyses for all knockout strains. Further, to demonstrate how proteome analysis identifies the effect of gene deficiency beyond traditional phenotyping tests, we provide in-depth analysis of two strains, *C8a*^*−/−*^ and *Npc2*^*+/−*^. The proteins encoded by these genes are well-characterized providing good validation of our method in homozygous and heterozygous knockout mice. Ig alpha chain C region, a poorly characterized protein, was among the differentiating proteins in *C8a*^*−/−*^. In *Npc2*^*+/−*^ mice, where histopathology and traditional tests failed to differentiate heterozygous from wild-type mice, our data showed significant difference in various lysosomal storage disease-related proteins. Our results demonstrate how to combine absolute quantitative proteomics with mouse gene knockout strategies to systematically study the effect of protein absence. The approach used here for blood plasma is applicable to all tissue protein extracts.

## Introduction

*Mus musculus* is the most used animal model in scientific research. It has high similarity with humans at the molecular level with 99% of human genes having homologs in the mouse genome^1^. Mice can model many human diseases, making them suitable to study rare monogenic disorders and complex multigenic diseases such as cancer, diabetes, and even anxiety^2–9^. Current genome manipulation techniques to knock out or silence a specific gene have allowed many human conditions to be reproduced in mice, enabling the study of disease mechanism and progression^10–14^.

These studies were largely performed using high throughput analytical methods. Analysis of mouse tissues in the context of health and disease has been done previously using microarray and deep sequencing technologies^15,16^. Although genes are the original template for proteins, it is the expressed proteins and their differential abundance that principally determine the function of cells and tissues. Hence, parallel to the various sequencing efforts, comprehensive studies at the proteome level have been performed in recent years and provided insight into the proteins that are differentially expressed between cells, tissues, organs, or organ systems, or are related to a specific condition or disease. These studies have revealed functional genomics insights beyond that derived from sequencing alone^17–20^. Such efforts have included the analysis of cells and tissues from wild type, transgenic, knockin, and knockout strains, and mice labeled in vivo with isotope using mass spectrometry-based methods^21–24^. Mass spectrometry is a versatile technique that allows system-wide study of the proteome^25^. In a typical bottom-up workflow, proteins are digested into peptides for analysis using liquid chromatography coupled to mass spectrometry (LC-MS/MS)^26^. Differential expression is inferred from mass spectrum signal intensity and good comparability across groups can be achieved using labeling approaches such as isobaric tagging using Tandem Mass Tag (TMT), Isobaric Tag for Relative and Absolute Quantitation (iTRAQ), or Stable Isotope Labeling with Amino acids in Cell culture (SILAC) ^27^. Multiple reaction monitoring (MRM) is considered the gold standard in quantitative measurements^28–30^. When combined with heavy labeled internal standards, high precision and accuracy were achieved while multiplexing assays for hundreds of proteins within a single experiment.

We set out to conduct a systematic comparison using large-scale, targeted proteomic analysis of the impacts caused by single-gene disruption. Two hundred and eighteen plasma samples from 90 female and 90 male mice for 30 knockout (KO) strains and 38 corresponding wild-type controls were analyzed. All KO strains and controls were on the C57BL/6N genetic background. The mutant mice were produced and phenotyped through a standardized pipeline of sequential tests by the International Mouse Phenotyping Consortium (IMPC). The KO gene targets were selected on the basis of their known involvement in diverse biological processes, with the goal of evaluating how plasma proteomics can complement clinical in vivo and terminal phenotyping tests (Table 1). We first chose homozygous (HOM) and heterozygous (HET) strains to study the effect of protein ablation (HOM) and reduced protein abundance (HET). Approximately 30% of the KO strains produced by the IMPC are embryo lethal or subviable^11^, so it was important to test if the proteomic analysis was sensitive enough to detect changes in heterozygous mice. We also included female and male mice to study sexual dimorphism at the plasma proteome level and determine possible interaction with gene KO related protein abundances. Further, we purposely included KO strains with various protein expression profiles including secreted, widely expressed, ubiquitous, and tissue-specific, as well as proteins with no known tissue specificity.

**Table 1:** List of knockout strains with the corresponding gene and protein annotations.

Knockout allele	Knockout gene (NCBI Gene ID)	Zygocyty^a^	UniProtKB accession	UniProtKB accession of human ortholog	Protein name	Protein function^b^	Gene Ontology biological process annotations	Significantly differentiated proteins (*p* value <0.05, log2 FC > 2)	Erythrocyte-specific proteins in the significantly differentiated proteins
*A2m*^*tm1b(NCOM)Mfgc*^	*A2m* (232345)	HOM	Q6GQT1	P01023	Alpha-2-macroglobulin-P	Protease inhibitor with activity against all four classes of proteinases	Female pregnancy; negative regulation of complement activation, lectin pathway; stem cell differentiation		
*Ahcy*^*tm1b(EUCOMM)Hmgu*^	*Ahcy* (269378)	HET	P50247	P23526	Adenosylhomocysteinase	Competitive inhibitor of *S*-adenosyl-l-methionine-dependent methyl transferase reactions	Chronic inflammatory response to antigenic stimulus; circadian sleep/wake cycle; one-carbon metabolic process; response to nutrient; *S*-adenosylhomocysteine catabolic process; *S*-adenosylmethionine cycle	C1QA; Ig heavy chain V region MOPC 47A; SERPINF1	
*Atp5b*^*tm1b(EUCOMM)Hmgu*^	*Atp5f1b* (11947)	HET	P56480	P06576	ATP synthase subunit beta, mitochondrial	Mitochondrial membrane ATP synthase produces ATP from ADP in the presence of a proton gradient across the membrane	Angiogenesis; ATP biosynthetic process; ATP metabolic process; cellular response to interleukin-7; lipid metabolic process; mitochondrial ATP synthesis coupled proton transport; negative regulation of cell adhesion involved in substrate-bound cell migration; positive regulation of blood vessel endothelial cell migration; proton transmembrane transport; receptor-mediated endocytosis; regulation of intracellular pH	ITIH1	
*Atp6v0d1*^*tm1b(EUCOMM)Hmgu*^	*Atp6v0d1* (11972)	HET	P51863	P61421	V-type proton ATPase subunit d 1	Subunit of the integral membrane V0 complex of vacuolar ATPase, which provides most of the energy required for transport processes in the vacuolar system	Brain development; cellular iron ion homeostasis; cellular response to increased oxygen levels; cilium assembly; vacuolar acidification; vacuolar transport		
*C8a*^*tm1b(EUCOMM)Hmgu*^	*C8a* (230558)	HOM	Q8K182	P07357	Complement component C8 alpha chain	Constituent of the membrane attack complex (MAC) that plays a key role in the innate and adaptive immune responses	Complement activation; complement activation, alternative pathway; complement activation, classical pathway; cytolysis	BOGM; CA1; CA2; C8A; C8B; C8G; HBZ; Ig alpha chain C region; ISG15	BPGM; CA1; CA2; HBZ; ISG15
*Cdk4*^*tm1b(NCOM)Mfgc*^	*Cdk4* (12567)	HET	P30285	P11802	Cyclin-dependent kinase 4	Serine/threonine-protein kinase component of the protein kinascyclin D-CDK4 (DC) complexes that are involved in regulation of G1 phase of the cell cycle	Adipose tissue development; animal organ regeneration; cell division; cellular response to insulin stimulus; cellular response to interleukin-4; cellular response to ionomycin; cellular response to lipopolysaccharide; cellular response to phorbol 13-acetate 12-myristate; circadian rhythm; G1/S transition of mitotic cell cycle; lens development in camera-type eye; negative regulation of cell cycle arrest; positive regulation of apoptotic process; positive regulation of cell population proliferation; positive regulation of cell size; positive regulation of fibroblast proliferation; positive regulation of G2/M transition of mitotic cell cycle; positive regulation of translation; protein phosphorylation; regulation of cell cycle; regulation of cell population proliferation; regulation of gene expression; regulation of insulin receptor signaling pathway; regulation of lipid biosynthetic process; regulation of lipid catabolic process; regulation of multicellular organism growth; response to drug; response to hyperoxia; response to lead ion; response to organic substance; response to testosterone; response to toxic substance; signal transduction		
*Dhfr*^*tm1b(EUCOMM)Wtsi*^	*Dhfr* (13361)	HET	P00375	P00374	Dihydrofolate reductase	Key enzyme in folate metabolism; involved in thymidylate, glycine, purine, and DNA precursor synthesis	Axon regeneration; dihydrofolate metabolic process; folic acid metabolic process; negative regulation of translation; one-carbon metabolic process; oxidation–reduction process; positive regulation of nitric-oxide synthase activity; regulation of removal of superoxide radicals; response to methotrexate; response to nicotine; tetrahydrobiopterin biosynthetic process; tetrahydrofolate biosynthetic process; tetrahydrofolate metabolic process	Ig heavy chain V region MOPC 47A; SERPINF1; KLKB1	
*Dync1li1*^*em1(IMPC)Tcp*^	*Dync1li1* (235661)	HET	Q8R1Q8	Q9Y6G9	Cytoplasmic dynein 1 light intermediate chain 1	Non-catalytic accessory component of the cytoplasmic dynein 1 complex that is thought to be involved in linking dynein to cargos and to adapter proteins that regulate dynein function	Cell cycle; cell division; cellular response to nerve growth factor stimulus; microtubule-based movement; microtubule cytoskeleton organization; positive regulation of mitotic cell cycle spindle assembly checkpoint; regulation of centrosome cycle		
*G6pd2*^*em1(IMPC)Tcp*^	*G6pd2* (14380)	HOM	P97324	P11413	Glucose-6-phosphate 1-dehydrogenase 2	Provide reducing power (NADPH) and pentose phosphates for fatty acid and nucleic acid synthesis	Glucose-6-phosphate metabolic process; glucose metabolic process; NADP metabolic process; pentose-phosphate shunt		
*Galc*^*tm1b(KOMP)Wtsi*^	*Galc* (14420)	HET	P54818	P54803	Galactocerebrosidase	Hydrolyzes the galactose ester bonds of galactosylceramide, galactosylsphingosine, lactosylceramide, and monogalactosyldiglyceride	Galactosylceramide catabolic process; myelination	Ig heavy chain V region MOPC 47A	
*Gnpda1*^*tm1b(KOMP)Mbp*^	*Gnpda1* (26384)	HET	O88958	P46926	Glucosamine-6-phosphate isomerase 1	May trigger calcium oscillations in mammalian eggs	Acrosome reaction; fructose 6-phosphate metabolic process; fructose biosynthetic process; generation of precursor metabolites and energy; glucosamine catabolic process; glucosamine metabolic process; *N*-acetylglucosamine catabolic process; *N*-acetylneuraminate catabolic process; UDP-*N*-acetylglucosamine biosynthetic process	Ig heavy chain V region MOPC 47A	
*Idh1*^*tm1b(EUCOMM)Wtsi*^	*Idh1* (15926)	HOM	O88844	O75874	Isocitrate dehydrogenase cytoplasmic	Catalyzes the reversible oxidative decarboxylation of isocitrate to yield α-ketoglutarate (α-KG) as part of the Krebs cycle	2-Oxoglutarate metabolic process; female gonad development; glutathione metabolic process; glyoxylate cycle; isocitrate metabolic process; NADP metabolic process; regulation of phospholipid biosynthetic process; regulation of phospholipid catabolic process; response to organic cyclic compound; response to oxidative stress; response to steroid hormone; tricarboxylic acid cycle		
*Iqgap1*^*tm1b(EUCOMM)Wtsi*^	Iqgap1 (29875)	HOM	Q9JKF1	P46940	Ras GTPase-activating-like protein IQGAP1	Plays a crucial role in regulating the dynamics and assembly of the actin cytoskeleton	Cell migration; cellular response to calcium ion; cellular response to epidermal growth factor stimulus; cellular response to fibroblast growth factor stimulus; cellular response to platelet-derived growth factor stimulus; epidermal growth factor receptor signaling pathway; fibroblast growth factor receptor signaling pathway; fibroblast migration; glomerular visceral epithelial cell development; negative regulation of dephosphorylation; neuron projection extension; platelet-derived growth factor receptor signaling pathway; positive regulation of cellular protein localization; positive regulation of dendrite development; positive regulation of focal adhesion assembly; positive regulation of MAPK cascade; positive regulation of MAP kinase activity; positive regulation of peptidyl-tyrosine autophosphorylation; positive regulation of protein kinase activity; positive regulation of vascular associated smooth muscle cell migration; regulation of actin cytoskeleton organization; regulation of cytokine production; regulation of mitotic cell cycle; response to angiotensin	CD5L; IGHG1; Ig gamma-2A chain C region sec; IGKC; Ig kappa chain V-II region 7S3; Ig kappa chain V-V region MOPC 149; IGHM; Jchain; SERPINF1; PSMA5	
*Lmbrd1*^*tm1b(EUCOMM)Hmgu*^	*Lmbrd1* (68421)	HET	Q8K0B2	Q9NUN5	Probable lysosomal cobalamin transporter	Probable lysosomal cobalamin transporter	Insulin receptor internalization; negative regulation of glucose import; negative regulation of insulin receptor signaling pathway; negative regulation of protein kinase B signaling		
*Mfap4*^*tm1b(NCOM)Mfgc*^	*Mfap4* (76293)	HOM	Q9D1H9	P55083	Microfibril-associated glycoprotein 4	Potentially involved in calcium-dependent cell adhesion or intercellular interactions	Cell adhesion; cellular response to UV-B; complement activation, lectin pathway; elastic fiber assembly; regulation of collagen metabolic process; supramolecular fiber organization; UV protection	MFAP4	
*Mmachc*^*tm1.1(NCOM)Mfgc*^	*Mmachc* (67096)	HET	Q9CZD0	Q9Y4U1	Methylmalonic aciduria and homocystinuria type C protein homolog	Catalyzes the reductive dealkylation of cyanocobalamin to cob(II)alamin, and the glutathione-dependent reductive demethylation of methylcobalamin and adenosylcobalamin	Cobalamin metabolic process; demethylation; glutathione metabolic process; oxidation–reduction process		
*Mvk*^*em1(IMPC)Tcp*^	*Mvk* (17855)	HET	Q9R008	Q03426	Mevalonate kinase	Catalyzes the phosphorylation of mevalonate to mevalonate 5-phosphate, a key step in isoprenoid and cholesterol biosynthesis	Cholesterol biosynthetic process; isopentenyl diphosphate biosynthetic process, mevalonate pathway; isoprenoid biosynthetic process; negative regulation of inflammatory response; negative regulation of translation		
*Nek2*^*em1(IMPC)Tcp*^	*Nek2* (18005)	HOM	O35942	P51955	Serine/threonine-protein kinase Nek2	Protein kinase which is involved in the control of centrosome separation and bipolar spindle formation in mitotic cells and chromatin condensation in meiotic cells	Blastocyst development; cell division; centrosome separation; chromosome segregation; meiotic cell cycle; mitotic sister chromatid segregation; mitotic spindle assembly; negative regulation of centriole–centriole cohesion; negative regulation of DNA binding; positive regulation of telomerase activity; positive regulation of telomere capping; positive regulation of telomere maintenance via telomerase; protein autophosphorylation; protein phosphorylation; regulation of attachment of spindle microtubules to kinetochore; regulation of mitotic centrosome separation		
*Npc2*^*tm1e(EUCOMM)Wtsi*^	*Npc2* (67963)	HET	Q9Z0J0	P61916	NPC intracellular cholesterol transporter 2	Intracellular cholesterol transporter which acts in concert with NPC1 and plays an important role in the egress of cholesterol from the lysosomal compartment	Cholesterol efflux; cholesterol homeostasis; cholesterol metabolic process; cholesterol transport; intracellular cholesterol transport; intracellular sterol transport; sterol transport	ACTG1; ENO1; CD97; EEF1A1; Ig heavy chain V region MOPC 47A; SERPINF1; PFN1; SELP; TNC; TALDO1; VCL	ACTG1
*Pebp1*^*em1(IMPC)Tcp*^	*Pebp1* (23980)	HOM	P70296	P30086	Phosphatidylethanolamine-binding protein 1	Serine protease inhibitor with activity against thrombin, neuropsin and chymotrypsin, tissue type plasminogen activator and elastase, and RAF1	Aging; eating behavior; hippocampus development; MAPK cascade; negative regulation of MAPK cascade; negative regulation of protein phosphorylation; positive regulation of acetylcholine metabolic process; positive regulation of cAMP-mediated signaling; positive regulation of mitotic nuclear division; regulation of neurotransmitter levels; regulation of the force of heart contraction; response to activity; response to calcium ion; response to cAMP; response to corticosterone; response to drug; response to electrical stimulus; response to ethanol; response to heat; response to oxidative stress; response to toxic substance; response to wounding; sperm capacitation	FN1	
*Phyh*^*tm1c(EUCOMM)Wtsi*^	*Phyh* (16922)	HOM	O35386	O14832	Phytanoyl-CoA dioxygenase, peroxisomal	Converts phytanoyl-CoA to 2-hydroxyphytanoyl-CoA.	2-Oxobutyrate catabolic process; 2-oxoglutarate metabolic process; fatty acid alpha-oxidation; isoprenoid metabolic process; methyl-branched fatty acid metabolic process		
*Pipox*^*tm1b(EUCOMM)Wtsi*^	*Pipox* (19193)	HOM	Q9D826	Q9P0Z9	Peroxisomal sarcosine oxidase	Metabolizes sarcosine, l-pipecolic acid and l-proline	l-lysine catabolic process to acetyl-CoA via l-pipecolate; oxidation–reduction process; tetrahydrofolate metabolic process	C1QA; KLKB1	
*Plk1*^*tm1b(EUCOMM)Hmgu*^	*Plk1* (18817)	HET	Q07832	P53350	Serine/threonine-protein kinase PLK1	Serine/threonine-protein kinase that performs several important functions throughout M phase of the cell cycle	Centrosome cycle; establishment of mitotic spindle orientation; establishment of protein localization; female meiosis chromosome segregation; G2/M transition of mitotic cell cycle; homologous chromosome segregation; microtubule bundle formation; mitotic cell cycle; mitotic cytokinesis; mitotic sister chromatid segregation; mitotic spindle assembly checkpoint; negative regulation of apoptotic process; negative regulation of cyclin-dependent protein serine/threonine kinase activity; negative regulation of transcription by RNA polymerase II; nuclear envelope disassembly; peptidyl-serine phosphorylation; polar body extrusion after meiotic divisions; positive regulation of peptidyl-threonine phosphorylation; positive regulation of proteasomal ubiquitin-dependent protein catabolic process; positive regulation of proteolysis; positive regulation of ubiquitin-protein ligase activity; positive regulation of ubiquitin-protein transferase activity; protein destabilization; protein localization to chromatin; protein localization to nuclear envelope; protein localization to organelle; protein phosphorylation; protein ubiquitination; regulation of cytokinesis; regulation of mitotic cell cycle; regulation of mitotic metaphase/anaphase transition; regulation of mitotic spindle assembly; regulation of protein binding; regulation of protein localization to cell cortex; signal transduction involved in G2 DNA damage checkpoint; synaptonemal complex disassembly		
*Pmm2*^*tm1b(EUCOMM)Hmgu*^	*Pmm2* (54128)	HET	Q9Z2M7	O15305	Phosphomannomutase 2	Involved in the synthesis of the GDP-mannose and dolichol-phosphate-mannose.	GDP-mannose biosynthetic process; mannose metabolic process; protein N-linked glycosylation; protein targeting to ER		
*Ptpn12*^*tm1b(NCOM)Mfgc*^	*Ptpn12* (19248)	HET	P35831	Q05209	Tyrosine-protein phosphatase non-receptor type 12	Dephosphorylates a range of proteins, and thereby regulates cellular signaling cascades	Cellular response to epidermal growth factor stimulus; peptidyl-tyrosine dephosphorylation; protein dephosphorylation; regulation of epidermal growth factor receptor signaling pathway; tissue regeneration	APOD; Ig heavy chain V region MOPC 47A	
*Pttg1*^*tm1b(EUCOMM)Wtsi*^	*Pttg1* (30939)	HOM	Q9CQJ7	O95997	Securin	Regulatory protein, which plays a central role in chromosome stability, in the p53/TP53 pathway, and DNA repair	Cell division; cellular process; chromosome segregation; DNA repair; homologous chromosome segregation; mitotic sister chromatid cohesion; negative regulation of cell population proliferation; negative regulation of mitotic sister chromatid separation; regulation of cell growth	IGHG1; IGHM; ITIH3; PSMA1	
*Rock1*^*tm1b(NCOM)Mfgc*^	*Rock1* (19877)	HET	P70335	Q13464	Rho-associated protein kinase 1	Protein kinase which is a key regulator of actin cytoskeleton and cell polarity	Actin cytoskeleton organization; actomyosin structure organization; apical constriction; apoptotic process; bleb assembly; cortical actin cytoskeleton organization; cytoskeleton organization; embryonic morphogenesis; I-kappaB kinase/NF-kappaB signaling; leukocyte cell–cell adhesion; leukocyte migration; leukocyte tethering or rolling; membrane to membrane docking; mitotic cytokinesis; mRNA destabilization; myoblast migration; negative regulation of amyloid-beta formation; negative regulation of amyloid precursor protein catabolic process; negative regulation of angiogenesis; negative regulation of bicellular tight junction assembly; negative regulation of myosin-light-chain-phosphatase activity; negative regulation of neuron apoptotic process; negative regulation of protein binding; neuron projection arborization; neuron projection development; peptidyl-serine phosphorylation; peptidyl-threonine phosphorylation; positive regulation of amyloid-beta clearance; positive regulation of autophagy; positive regulation of cardiac muscle hypertrophy; positive regulation of connective tissue replacement; positive regulation of focal adhesion assembly; positive regulation of gene expression; positive regulation of MAPK cascade; protein localization to plasma membrane; protein phosphorylation; regulation of actin cytoskeleton organization; regulation of actin filament-based process; regulation of angiotensin-activated signaling pathway; regulation of blood vessel diameter; regulation of cell junction assembly; regulation of cell migration; regulation of establishment of endothelial barrier; regulation of keratinocyte differentiation; regulation of microtubule cytoskeleton organization; regulation of neuron differentiation; regulation of synaptic vesicle endocytosis; response to angiotensin; response to transforming growth factor beta; Rho protein signal transduction		
*Sra1*^*tm1b(EUCOMM)Hmgu*^	*Sra1* (24068)	HOM	Q80VJ2	Q9HD15	Steroid receptor RNA activator 1	Functional RNA which acts as a transcriptional coactivator that selectively enhances steroid receptor-mediated transactivation	Apoptotic process; cell differentiation; cellular response to estrogen stimulus; negative regulation of myoblast differentiation; positive regulation of transcription by RNA polymerase II; regulation of apoptotic process; regulation of mitotic cell cycle; regulation of transcription by RNA polymerase II	ENO1; BPGM; CA1; CA2; BLVRB; HBA; HBB-B1; HBZ; Ig heavy chain V region MOPC 47 A; PRDX2; PRDX6; SOD1; ISG15	BPGM; CA1; CA2; BLVRB; HBA; HBB-B1; HBZ; PRDX2; PRDX6; SOD1; ISG15
*Ulk3*^*em2(IMPC)Tcp*^	*Ulk3* (71742)	HOM	Q3U3Q1	Q6PHR2	Serine/threonine-protein kinase ULK3	Serine/threonine-protein kinase that acts as a regulator of Sonic hedgehog (SHH) signaling and autophagy	Autophagy; fibroblast activation; negative regulation of smoothened signaling pathway; positive regulation of smoothened signaling pathway; protein autophosphorylation; smoothened signaling pathway	C1QC; FGL1	
*Ywhaz*^*tm1b(EUCOMM)Hmgu*^	*Ywhaz* (22631)	HET	P63101	P63104	14-3-3 protein zeta/delta	Adapter protein implicated in the regulation of a large spectrum of both general and specialized signaling pathways	Establishment of Golgi localization; Golgi reassembly; histamine secretion by mast cell; protein phosphorylation; protein targeting; protein targeting to mitochondrion; regulation of cell death; regulation of ERK1 and ERK2 cascade; regulation of synapse maturation; response to drug; signal transduction; synaptic target recognition		

^a^*HOM* homozygote, *HET* heterozygote.

^b^As described in the UniProtKB database.

Selection of proteins measured was based on their involvement in various biological pathways and detectability^31^. The abundances of 375 plasma proteins were measured using MRM assays validated according to the CPTAC guidelines^32^ (Supplementary Table 1 and Supplementary Fig. 1). The measured plasma protein concentrations provided a molecular phenotype for each KO strain in addition to the clinical in vivo and terminal test phenotype data from the IMPC. To our knowledge, this is the first large-scale analysis of plasma proteins in KO mice.

## Results

We proteotyped 30 mouse KO strains and corresponding wild-type controls using quantitative targeted proteomics. We realize that the number of strains analyzed is small compared to other phenotyping test and interpretation studies; e.g. Karp et al.^33^ analyzed 2186 strains for sexual dimorphism in 238 standard IMPC phenotyping tests. Proteotyping on that scale, i.e. >54,000 samples would require a large coordinated effort in addition to the associated high operational costs. Recognizing this limitation, strain selection was particularly important to address the questions of proteotyping capabilities to detect protein abundance differences between HOM and HET genotypes, female and male mice, and protein expression profiles. Our results identified differences for all three criteria suggesting that proteotyping by current state-of-the-art quantitative methods is possible, biologically relevant, and scalable.

**Fig. 1:**
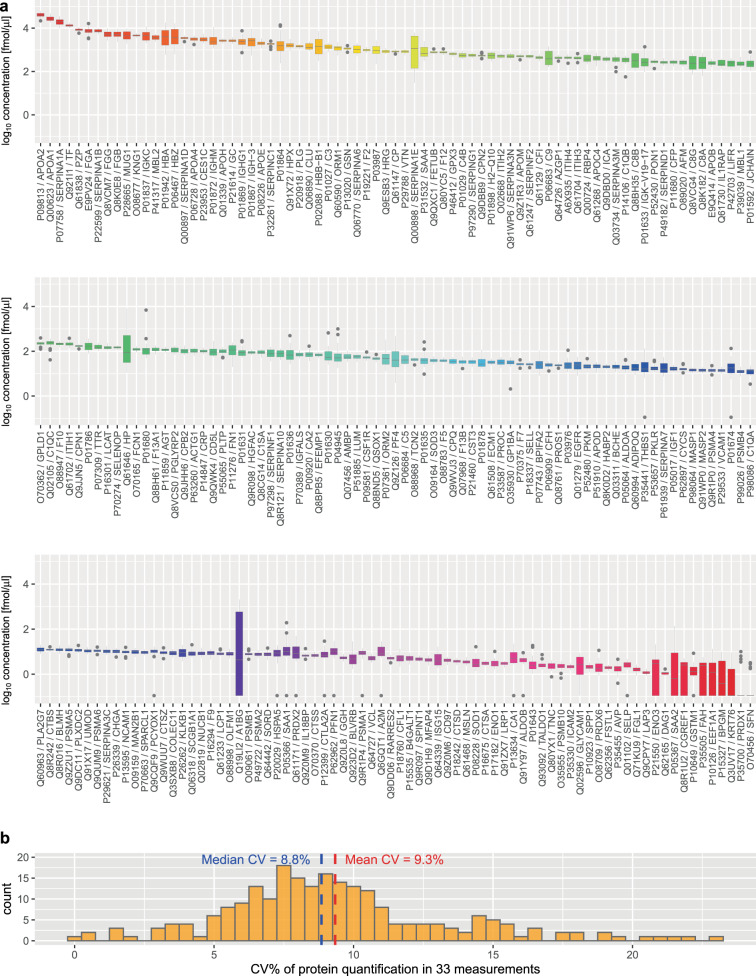
Dynamic range and variance. **a** Dynamic range of determined plasma protein concentrations in controls. Boxplots show median and interquartile range (center line, median; box limits, upper and lower quartiles; whiskers, 1.5× interquartile range; points, outliers). *N* = 38. Note that the *y*-axis is presented on log_10_ scale. **b** Histogram of protein coefficient of variation (CV) based on *N* = 33 measurements of 226 proteins in a pooled sample. Individual data for all proteins are available in Supplementary Table 1.

Of the 375 measured proteins, 284 were detectable, and 234 were quantifiable with a minimum of 5% of all measurements above the lower limit of quantification (LLOQ). Two hundred and twenty-six proteins were quantified within the dynamic range of the assays in all three mice of at least one mouse strain and sex (Fig. 1, Supplementary Fig. 2 and Supplementary Table 1); therefore, we used the minimal set of 226 proteins in our subsequent analyses. The determined concentrations of these proteins spanned five orders of magnitude, ranging from 0.27 to 6.2 × 10^4^ fmol/μL plasma, demonstrating the large dynamic range that is quantifiable using LC-MRM/MS (Fig. 1a). Overall, these measurements had very good precision^34–36^ with an average coefficient of variation (CV) of 9.3%, and all were below 23% (Fig. 1b).

### Proteins originating from erythrocytes and platelets

**Fig. 2:**
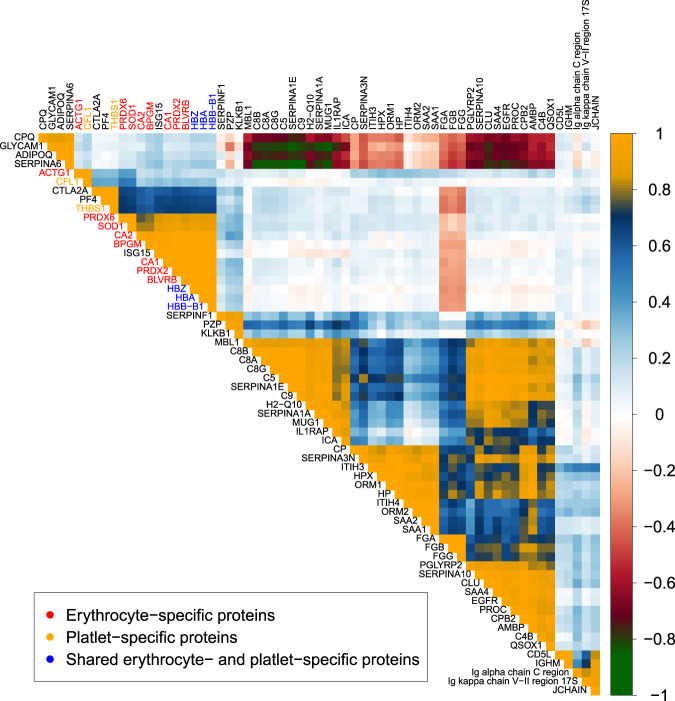
A reduced correlation matrix of measured proteins, showing good clustering of erythrocyte and platelet-specific proteins. A minimum absolute Pearson’s correlation of 0.8 was applied to reduce the dimension of the matrix.

Due to daily differences in sample collection and processing, plasma samples routinely contain variable amounts of proteins originating from red blood cells and platelets. Recently, Geyer et al.^33^ identified the contaminating proteins from erythrocytes and platelets in human plasma, which can be used as indicators of differences in sample processing and should be excluded from inference analysis between biological groups, unless independence of sample handling can be established. We measured 12 erythrocyte- and 10 platelet-specific intracellular proteins that were previously identified by Geyer et al. as common contaminants. Correlation analysis in all samples between all proteins (Supplementary Fig. 3 and Fig. 2 filtered for minimum absolute Pearson coefficient of 0.8) showed the clustering of these proteins in correlated groups, indicating their amounts measured in some of our samples are in fact artifacts of sample processing. In addition to the erythrocyte proteins identified by Geyer et al.^37^, a strong correlation was also observed with Ubiquitin-like protein ISG15. This intracellular protein is involved in erythroid differentiation^38^; therefore, we concluded ISG15 also originated from erythrocytes during sample collection. In our further analyses, these 22 reported erythrocyte contaminants plus ISG15 were closely examined. Specifically, if any of these 23 proteins were significantly altered in a comparison between groups, we determined if the other erythrocyte proteins showed a similar trend. This allowed us to determine if the differential expression was an artifact of sample collection or a signature of the gene KO. As our sample collection method produces platelet-rich plasma, we opted to consider platelet proteins as part of our samples. Our results (Table 1) emphasize the importance of carefully considering whether the presence of intracellular proteins in plasma reflects a biological condition, or if they are the result of sample processing.

### Sexual dimorphism

**Fig. 3:**
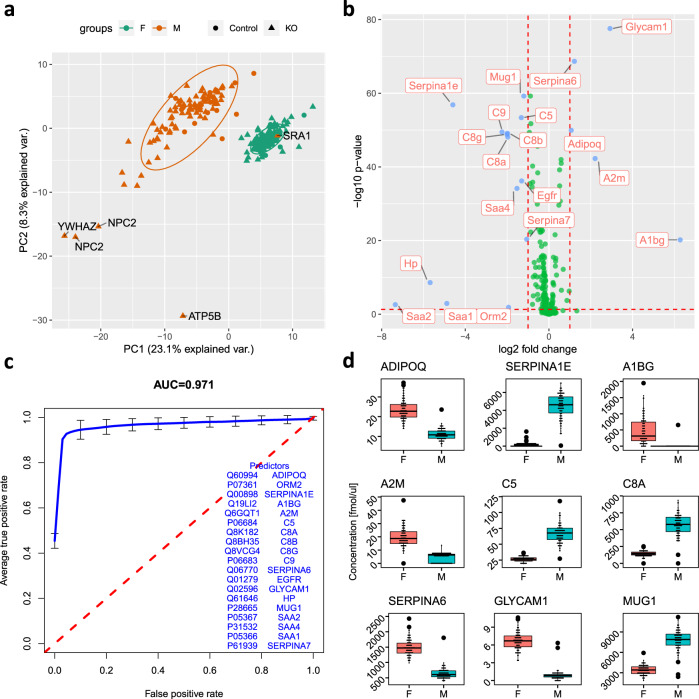
Clear discrimination between male and female mice. **a** PC1 and PC2 projection of PCA analysis on all measured proteins shows two groups that can clearly be mapped to male and female mice. **b** Volcano plot of all measured proteins annotated with the significant discriminators. Positive values on the *x*-axis indicates increase in the abundance in the plasma of male mice. **c** Average ROC curve with cross validation using logistic regression on top discriminators showing *C*-statistics of 97% for the discrimination between males and females. **d** Boxplots of selected discriminating proteins between male and female mice (center line, median; box limits, upper and lower quartiles; whiskers, 1.5× interquartile range; points, outliers).

Various studies have previously demonstrated sex-specific differences in protein expression^39–41^. Similar to findings in brain, urine, and apheresis platelet supernatant, we observed a clear protein signature associated with sex for mouse plasma, in both KO and control mice. Using principle component analysis (PCA), wild type and KO mice clustered clearly according to sex (with the exception of one outlier from SRA1) in PC1–PC2 plane, which explained 31.4% of the variance in our dataset (Fig. 3a). Proteins with a high significant difference in expression (*p* value < 0.01 and fold change of 2) were identified (Fig. 3b), and can be used to create a sex-specific protein signature (Fig. 3c). The top discriminating proteins have been previously reported to have differential expression in males and females, including Adiponectin (ADIPOQ), Alpha-1-antitrypsin (SERPINA1E), Alpha-1B-glycoprotein (A1BG), Alpha-2-macroglobulin-P (A2M), and the complement components (C5, C8A, C8B, C8G)^37,42–47^. The concentrations of these proteins in the plasma of male and female mice are shown in Fig. 3d. Similarly, Transcortin (SERPINA6)^48^, Epidermal growth factor receptor (EGFR)^49^, Glycosylation-dependent cell adhesion molecule-1 (GLYCAM1)^50^, Haptoglobin (HP)^51^, Murinoglobulin-1 (MUG1)^52,53^, Serum amyloid A proteins (SAA2, SAA4, SAA1)^54^, and Thyroxine-binding globulin (SERPINA7)^55^ were reported previously as sexually dimorphic.

We were able to measure good discrimination over a wide dynamic range spanning from a few fmol/μL in glycosylation-dependent cell adhesion molecule (1 GLYCAM1), up to thousands in Alpha-1-antitrypsin 1–5 (SERPINA1E) and corticosteroid-binding globulin (SERPINA6) as shown in Fig. 3d.

When we compared the profile obtained in our work to a recent study on sexual dimorphism in human plasma proteins^56^, in which 142 proteins were identified to be differential between females and males, only three proteins were shared: adiponectin, a1-antitrypsin, and thyroxine-binding globulin.

Previously it was shown that 56.6% of the phenotypic continuous (non-categorical) measurements performed by the IMPC are associated with sex^57^. Our results extend these findings to show sexual dimorphism at the molecular level in plasma.

### Correlation with standard phenotyping tests

**Fig. 4:**
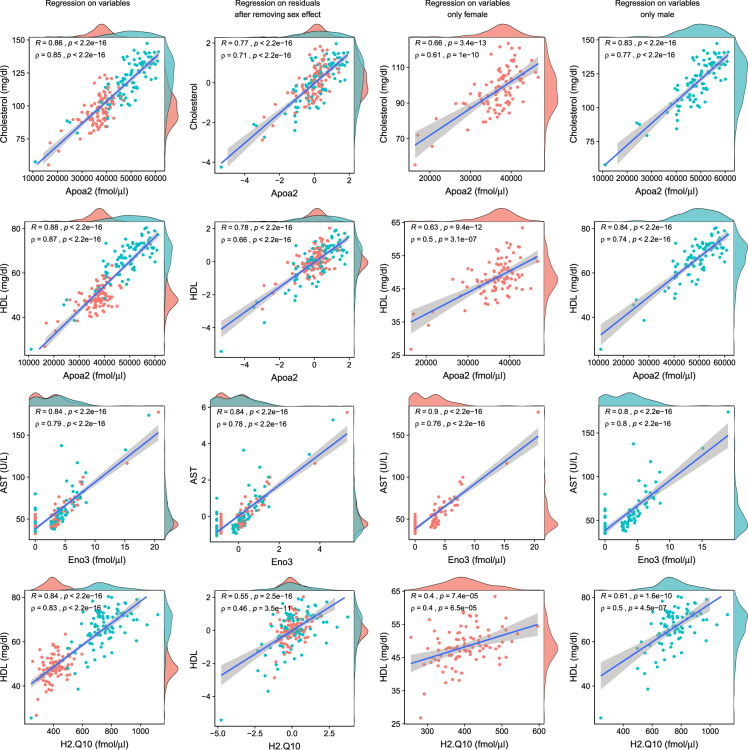
Top correlations between classical clinical phenotyping tests and protein abundances measured by MRM-MS with internal standards. For the correlations we included measurements from the wild-type mice and all gene knockout strains. Colors of dots and marginal histograms indicate sex with blue refer to male.

The mice used here were characterized as part of the IMPC program^58^ using standardized tests to measure biological parameters from the hematological, metabolic, cardiovascular, musculoskeletal, and neurological systems^59^. Since our study focused on blood plasma, we compared our proteomic data with available clinical chemistry, hematology, and body composition measurements. We obtained several good correlations between the proteomic and traditional phenotyping measurements despite their separation in time, space, and technology, i.e. correlated values were obtained from measurements on frozen samples performed years apart at different locations using different technologies. Figure 4 shows selected correlations found with Spearman correlation of around 0.8. Strong correlations identified were between high-density lipoprotein (HDL) and cholesterol with apolipoproteins A1 and A2, which was expected given the role of these proteins as major structural components of the high-density lipoprotein complex. Aspartate aminotransferase (AST) is an enzyme involved in amino acid metabolism and its level in blood is often used as an indicator of liver function and damage. In our data, we identified a correlation between AST and beta-enolase, both are enzymes essential for glycolysis/gluconeogenesis^60^. H-2 class I histocompatibility antigen Q10 has been noted to associate with lipids in C57BL/6 mouse plasma in other studies^61^, and was found to correlate with measured HDL in our data as well. For these correlations, sexual dimorphism was a clear confounding factor as can be seen in Fig. 4. However, despite decreases in strength, correlations persist after adjusting for sex effect and performing the regression on the residuals. Similarly, regression on stratified data showed similar trends.

The goal of phenotyping KO mice is to identify the consequences of gene dysfunction, which in turn can provide insight into gene function, gene pleiotropy (comorbidities), and generate hypotheses for mechanisms of disease. For the strains examined in this study, none of the standard tests considered here (clinical chemistry, hematology, or body composition) discriminated knockouts from their corresponding controls using the IMPC’s standard statistical analyses^62^. In such cases, molecular level investigation may identify differences, as discussed below, aiding the detailed characterization of a KO mouse strain.

### Proteomic phenotyping of gene deficiency in knockout mice

**Fig. 5:**
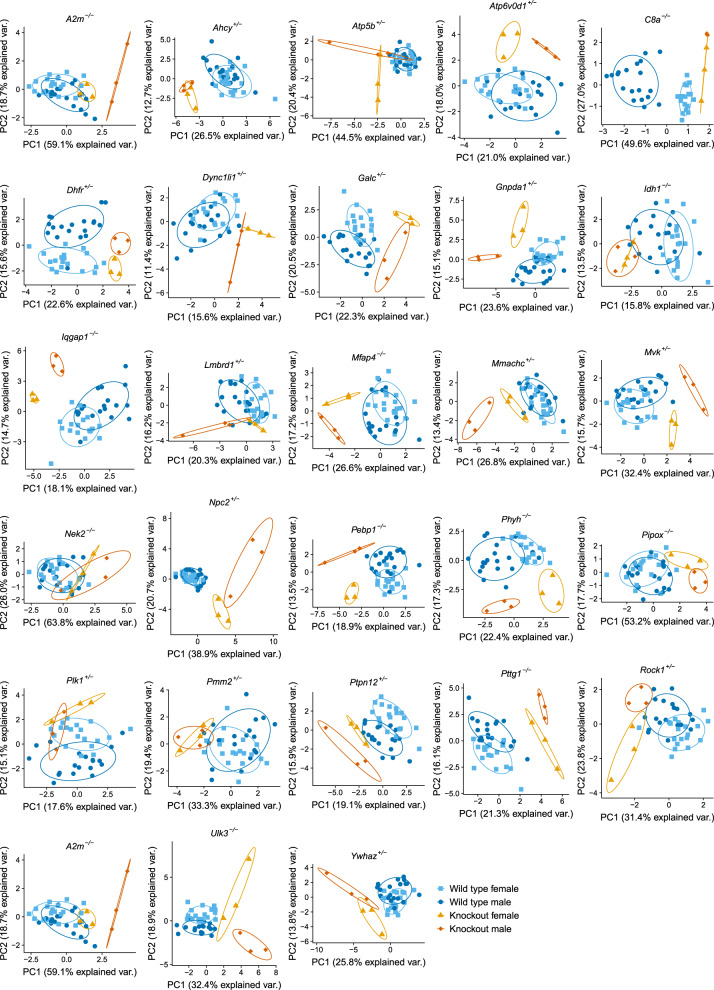
Separation between knockout and wild-type mice using protein concentration determined by targeted proteomics. Each plot represents the plane of the first two principle components performed on selected proteins (Supplementary Table 1).

Although the largest variation in plasma protein abundance was linked to sexual dimorphism (Fig. 3), we were able to determine proteomic signatures specific to 28 gene knockouts (Fig. 5). Here we used simple PCA on the proteins selected by Least Absolute Shrinkage and Selection Operator—LASSO^63^ (Supplementary Table 2) to demonstrate the possible grouping of samples in the PC1 and PC2 plane. For two of the KO strains, *G6pd2* and *Sra1*, no discriminating proteins were found. In this analysis we removed all erythrocyte-specific proteins for simplicity. The discrimination observed highlights how targeted proteomics with simple data analysis can be used for molecular phenotyping. We have also previously shown possible discrimination between co-housed and co-raised littermate wild type and KO mice (thus much less effect of possible environmental variables) using our targeted proteomics assays and data analysis^64^.

For each KO strain, we next identified proteins significantly affected by the absence of the gene using Mann–Whitney–Wilcoxon test and calculated the fold change of proteins based on the mean values. We continued our analysis with the proteins differentially expressed (twofold difference in abundance between groups with *p* value < 0.05) which are listed in Table 1. The number of these proteins ranged from zero as seen in *Idh1*^*−/−*^ and *A2m*^*−/−*^ mice, to a strong effect with up to 10 and more differentiating proteins as seen in *Npc2*^*+/−*^ and *Iqgap1*^*−/−*^ mice. Our analysis confirmed the expected absence of protein, when measured, in the corresponding gene KO mouse, as in the case of C8A in the *C8a*^*−/−*^ strain. Similar confirmation was also demonstrated in a parallel work, in which we confirmed expected absence of proteins in gene knockdown experiments by targeted proteomics^64^.

We further performed multiple overrepresentation analyses (ORAs) using the differentially expressed proteins obtained by Mann–Whitney–Wilcoxon test in combination with the discriminating proteins selected by LASSO^63^. ORA allows identification of known functions, processes, and diseases that are associated with a set of genes or proteins of interest^65^. We performed systematic ORA using multiple knowledgebases including Gene Ontology Terms—GO^66^, Molecular Signatures—MsigDB^67^, molecular pathway using Kyoto Encyclopedia of Genes and Genomes—KEGG^68^ as well as Reactome^60^, Disease Ontology—DO^69^, diseases and their gene associations using DisGeNET^70^, and Medical Subject Headings—MeSH for processes and diseases^71^. While some of these resources are overlapping in context, they differ in content and curation method, hence reporting different views. For disease-related analyses, the human orthologs were used. When both mouse proteins and human orthologs were available in a resource, as for Reactome and MeSH processes, we performed parallel analyses. In total, we performed 10 ORA for each mouse KO mouse strain. The results are included in Supplementary ORA-report 1 for discriminating proteins from Mann–Whitney–Wilcoxon test, and Supplementary ORA-report 2 for using the combined protein list of the statistical test and LASSO regression.

### *C8a*^*−/−*^ and *Npc2*^*+/*−^ strains

**Fig. 6:**
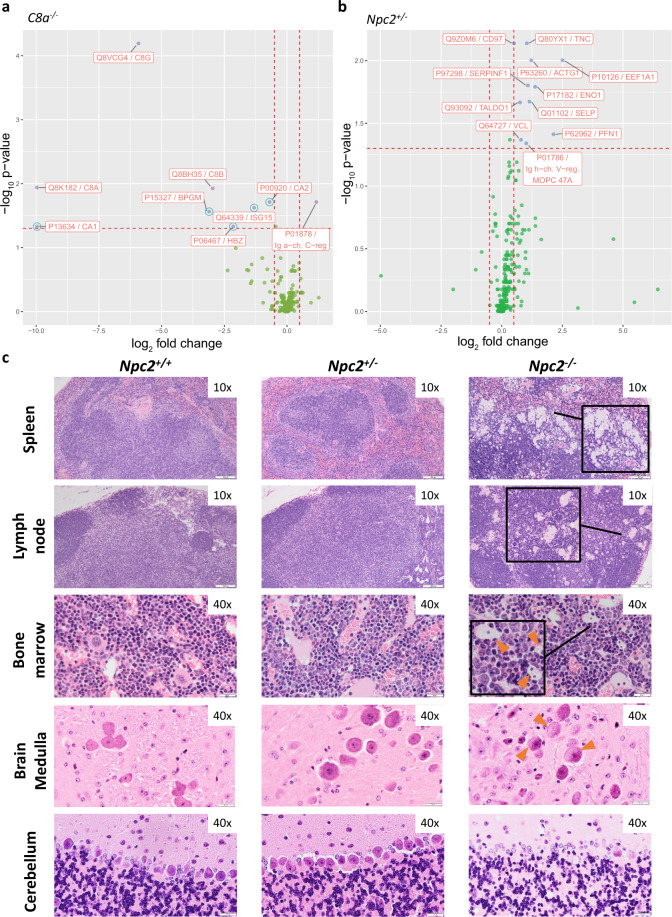
Plasma proteome profiles of *C8a*^*−/−*^ and *Npc2*^+/−^ mouse strains. **a** Differences in plasma proteome profiles between *C8a*^*−/−*^ mice and C57BL/6NCrl background controls; data points in blue circles represent erythrocyte-originating proteins likely introduced during sample collection. **b** Differences in plasma proteome profiles between *Npc2*^*+/−*^ mice and C57BL/6NCrl background controls. **c** Images of hematoxylin and eosin (HE)-stained tissue sections from spleen, lymph node, bone marrow, brain medulla, and cerebellum of wild type, heterozygous and homozygous *Npc2* KO, i.e. *Npc2*^*+/+*^, *Npc2*^*+/−*^, and *Npc2*^*−/−*^ respectively, all females. The absence of the Npc2 protein in *Npc2*^*−/−*^ mice is reflected in histopathological changes compared to *Npc2*^*+/+*^, while *Npc2*^*+/−*^ shows no such changes. In spleen and lymph node hemolymphatic histiocytosis (foamy cells, enlarged lipid-laden macrophages) are visible only in *Npc2*^*−/−*^. Brain sections show an example of widespread neuronal microvesicular cytoplasmic vacuolation in vestibular nuclei of the medulla oblongata in the homozygous mice. Sections of cerebellum show Purkinje cell loss and degeneration in *Npc2*^*−/−*^, but not in *Npc2*^*+/+*^ nor *Npc2*^*+/−*^. Although no pathological difference was observed in the tissue sections of *Npc2*^*+/−*^, there was a clear discriminating protein profile quantified in collected blood plasma of these mice compared to the background wild type.

**Fig. 7:**
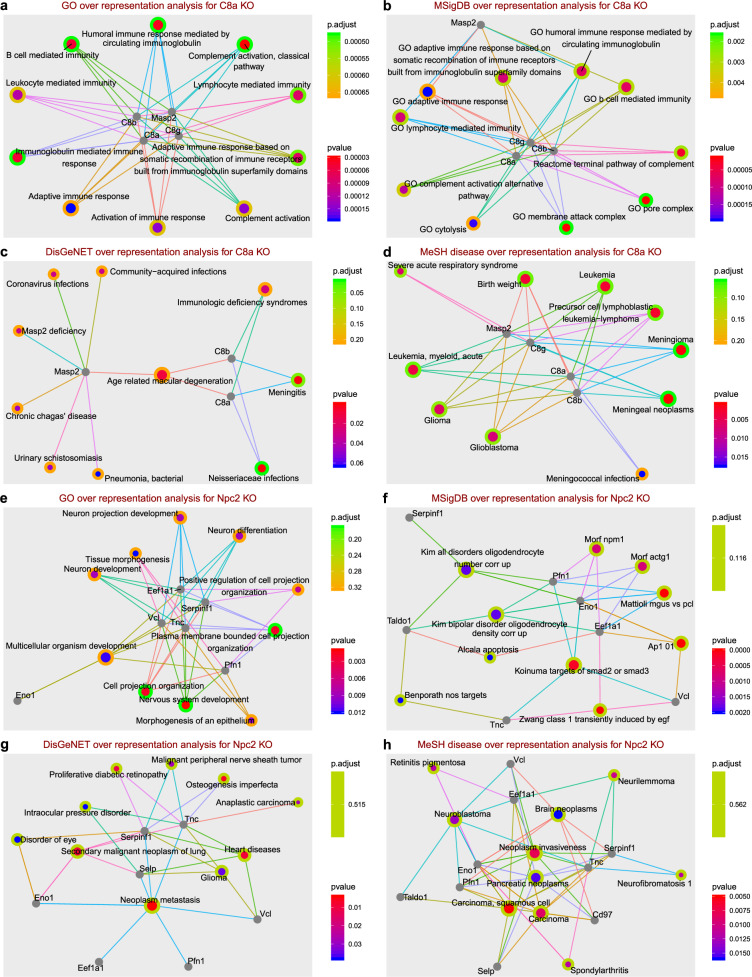
Overrepresentation analysis using discriminating proteins in *C8a*^*−/−*^ and *Npc2*^*+/−*^ mice. **a**–**d** Overrepresentation analyses of discriminating proteins in *C8a*^*−/−*^ mice using gene ontology—GO, molecular signature—MsigDB, disease-gene association—DisGeNET, and medical subject heading for human diseases—MeSH. **e**–**h** Overrepresentation analyses of significantly discriminating proteins in *Npc2*^*+/−*^ mice. For *C8a*^*−/−*^ all discriminating proteins from the significance test (Table 1 and Fig. 6a) as well as LASSO regression (Supplementary Table 2) were used, where for *Npc2*^*+/−*^ discriminating proteins from only the significance test were used (Table 1 and Fig. 6b). Details on protein selection are in text under “Proteomic phenotyping of gene deficiency in knockout mice using plasma”. Additional overrepresentation analyses, including molecular pathways using KEGG and Reactome knowledgebases, MeSH processes in mouse and human as well as Disease Ontology can be found in the Supplementary Material; in Supplementary ORA-report 1 discriminating proteins form the significance test were used, and in Supplementary ORA-report 2 discriminating proteins form the significance test as well as LASSO regression were used. Other KO mouse strains are also included in the two overrepresentation analysis reports. Gray circles refer to proteins, colored circles to ORA corresponding annotations, color corresponds to *p* value and Benjamini–Hochberg adjusted *p* value as in the color key, and size of annotation circles corresponds to number of connections.

Here we report in-depth analysis of two knockouts, *C8a*^*−/−*^ and *Npc2*^*+/−*^. Figure 6 shows volcano plots with differentially abundant proteins for these two KO strains, while Fig. 7 represents part of the functional analyses performed. The complete results from all KO strains are included in Table 1 and in the Supplementary Materials.

### *C8a*^*−/−*^ mice

The C8 alpha (C8A) protein combines with C8 beta (C8B) and C8 gamma (C8G) to form the complement component 8 (C8) protein complex, which plays a key role in the immune response by participating in the assembly of the membrane attack complex (MAC)^72^. In response to infection, MAC forms a pore in the pathogen cell membranes, resulting in cell lysis and death. Full KO of the *C8a* gene was confirmed by MRM analysis, since C8A was measured in control mice but not detected in the *C8a*^*−/−*^ (*C8a*^*tm1b(EUCOMM)Hmgu*^) mice (Fig. 6a). The concentration of C8B was decreased in all *C8a*^*−/−*^ males, and no C8B was detected in any of the *C8a*^*−/−*^ females. Concentration of C8G was also decreased in all *C8a* KO mice. C8A, C8B, and C8G are encoded by separate genes, indicating that in the absence of the C8A, the C8 complex does not form and C8B and C8G are cleared from the circulation.

The difference between wild type and *C8a*^*−/−*^ KO mice on the plasma protein level can clearly be seen in Fig. 6a and in Fig. 5. Two hundred and forty-six phenotyping tests for *C8a*^*−/−*^ mice performed originally by the IMPC reported no significance between wild type and KO using the IMPC’s standard statistical analysis (Supplementary Fig. 4 and Supplementary Table 3). The IMPC’s automated statistical analysis uses significance at a threshold of 0.0001 for unadjusted *p* value obtained by regression analysis. When applying the criteria we used to evaluate differences in protein abundances (Benjamini–Hochberg adjusted *p* value threshold of 0.05) to the IMCP phenotyping tests, we obtained a single significance corresponding to the difference in neutrophil differential count between wild type and KO. Furthermore, we used a non-parametric test to compare protein abundances, which is more suitable for low sample number but usually also more stringent (a parametric t-test in case of *C8a*^*−/−*^—which corresponds to a regression analysis with varying intercept—produced 13 additional differentiated proteins besides the 10 included in our study).

ORAs of *C8a*^*−/−*^ included five proteins, C8A, C8B, C8G, MASP2, and Ig alpha chain C region, which were obtained by hypothesis testing (Table 1) and selected by LASSO regression (Supplementary Table 2) as discriminators (Fig. 7a–d). While ISG15 levels showed a significant change between KO and control samples, it had a high correlation with erythrocyte-originating proteins and thus we concluded it was a sample preparation contaminant although not reported as such by Geyer et al.^33^ Nonetheless, we performed the ORAs with and without ISG15 to test its effect, which agreed with the rest of the protein set. Processes and functions overrepresented in this set of proteins showed various immune system related entries which reflected the role of the complement 8 complex and MASP2 in the complement system (as well as ISG15 in the innate immune system when included). Both mediated and adaptive immune responses were represented, which was expected given the terminal role of complement 8 in the innate immune response through its classical, alternative, and lectin pathways. MASP2, on the other hand, takes part only in the lectin pathway of the complement. When included, ISG15 also enriched immune system related functions through its antiviral and DNA repair roles. To that end, the proteins differentiated in their abundance in *C8a*^*−/−*^ mice fall into two categories: those with direct interaction with C8A within the complement 8 complex and those indirectly affected through the impact of C8A absence on the innate immune system.

Disease-related ORA results could be linked to an impaired innate immune system, including various infections^73^ and leukemia^74^. *Neisseriaceae* infections, for example, are linked specifically to the deficiency of complement 8 which hinders the formation of MAC^75,76^. Ig alpha chain C region was the only protein upregulated in *C8a*^*−/−*^ compared to the control mice. Having this protein in a set of discriminating proteins that enriches for innate immune system functions is noteworthy. Among the 20 measured immunoglobins, Ig alpha chain C region is the only statistically significant discriminator in *C8a*^*−/−*^ KO mice. Currently, little is known about this protein^77^, and ORA results did not link it to any available annotation in the different knowledge bases we used. Recent studies associated Ig alpha chain C region with Duchenne muscular dystrophy (in *Mdx4cv* mouse model), prion effect on liver (in *PrPC* KO mice), as well as the glycoproteome of prion infected mice^78–80^. While our experiments were not sufficient to conclude a direct link of the Ig alpha chain C region to the complement system or immune system, our data suggest a possible link with additional studies necessary to confirm this. In conclusion, targeted proteomics analysis using *C8a*^*−/−*^ mice was able to detect the effect of immunodeficiency resulting from an impaired complement system.

### *Npc2*^*+/−*^ mice

NPC1 and NPC2 are endosomal/lysosomal proteins involved in the transport of cholesterol. In humans, mutations in either *NPC1* or *NPC2* lead to the development of Niemann–Pick disease type C (NPC disease), a lysosomal storage disorder with a broad spectrum of visceral and neurological symptoms resulting from cellular accumulation of cholesterol and glycolipids. Individual lysosomal storage disorders are rare but collectively affect 1 in 5000 births with NPC disease affecting 1 in 10,000 (ref. ^81^). In addition to the aggressive cerebral and visceral inflammation which are hallmarks of NPC disease, generalized immune dysfunction and hematological defects, such as thrombocytopenia and anemia, also occur^82,83^. NPC disease, as with most lysosomal storage diseases, is inherited in an autosomal recessive manner, affecting homozygous individuals only. In our study we included heterozygous KO and wild-type mice for the proteomics analysis, and performed histopathology on tissue sections from wild type, heterozygous, and homozygous animals (Fig. 6b). Heterozygous mice were primarily phenotyped because *Npc2*^−/−^ animals were emaciated, ataxic, and needed to be euthanized at clinical endpoint at ~10 weeks of age. Consequently, all *Npc2*^*−/−*^ histopathology was done on 10-week-old animals. Phenotyping tests for *Npc2*^*+/−*^ mice performed by IMPC are included in Supplementary Fig. 5 and Supplementary Table 4. Analysis of the plasma proteome of *Npc2*^*+/−*^ (*Npc2*^*tm1e.1(EUCOMM)Wtsi*^) mice revealed dysregulation of proteins involved in hemostasis, particularly in platelet degranulation (actin, ACTG1; P-selectin, SELP; vinculin, VCL). Several proteins associated with exosomes were also upregulated (actin, ACTG1; CD97 antigen, CD97; Elongation factor 1-alpha-1, EEF1S1; vinculin, VCL) indicating trafficking dysregulation. Histopathological examination of spleen, lymph node, bone marrow, brain, and cerebellum sections revealed no difference between wild type and heterozygous mice; in contrast, homozygous mice showed common lysosomal storage disease phenotypes (Fig. 6c). This corroborates previous histopathological observations obtained in a zebrafish model for Npc1 (ref. ^84^), in which liver tissue sections of wild type and heterozygous larvae were similar, but different from homozygous larvae. While the known effect of NPC2 dysregulation, i.e. NPC disease is autosomal recessive, which can be confirmed by the absence of pathological phenotype in heterozygous mice, changes at the plasma proteome level in *Npc2*^*+/−*^ were quantifiable. In an attempt to investigate these changes, we performed multiple ORA.

Initially we included 18 proteins for ORAs, ENO1, CD97, EEF1A1, Ig heavy chain V region MOPC 47A, SERPINF1, PFN1, SELP, TNC, TALDO1, VCL, ORM2, PZP, CP, FETUB, FCN1, SPINT1, CTLA2A, and SAA1. This set was obtained by combining the results from hypothesis testing and LASSO regression (Table 1 and Supplementary Table 2). Although various associations were found, these had high *p* values. Reducing the analysis to only those proteins found significant by Mann–Whitney–Wilcoxon test (Table 1) improved the ORA adjusted *p* values; nevertheless, these were still above 0.1 (Fig. 7e–h, Supplementary ORA-report 1 and Supplementary ORA-report 2). Taking into account the investigatory nature of such analysis to drive hypothesis generation and future research, we investigated the overrepresented entries based on ordered *p* values. Multiple entries were related to neural development specifically and to cell growth in general. While NPC2 is mainly associated with metabolism and NPC disease, disease ORAs resulted in multiple cancer associations. A direct characterization of the relation of serum NPC2 to cancer has been reported previously^85^, and has linked upregulated NPC2 levels to breast, colon, and lung cancers, and downregulated levels to kidney and liver cancers in humans. Our results extend these findings and suggest that mice with NPC2 deficiency express a cancer-related protein profile in blood plasma. Further validation of these results is needed and may shed light on the less understood role of NPC2 in cancer. Including a targeted proteomics assay for NPC2 in future analyses will be beneficial to assess its level in the heterozygous KO mice. Furthermore, proteomics analysis of brain, spleen, lymph node, liver, and other tissues will advance the characterization of the *Npc2*^*+/−*^ KO mice. The identification of heterologous pathways and disease areas affected by gene ablation (homozygous null) or dosage (heterozygous null) demonstrates the potential for proteomic analyses to increase knowledge about gene and protein function. We believe that complementary proteomic analyses may augment current methodologies to assign significance to variants^86,87^ or disease risk^88^ by assessing impacts on pathways known to be involved in disease.

## Discussion

Proteomic phenotyping of KO mice using MRM mass spectrometry is a promising method for studying and understanding the function of genes beyond what can be determined through clinical in vivo and terminal test phenotyping alone^89^. Here we presented a broad proteotyping approach that can be incorporated as a complimentary test in large or small-scale phenotyping studies. We characterized the plasma protein profile of single-gene KO strains deficient for 30 genes. Our validated assays successfully quantified 226 proteins covering five orders of magnitude. All protein measurements had excellent precision with an average CV of 9.3%.

A strong sex-specific signature in measured plasma proteins was identified including 19 up- and downregulated proteins between female and male mice with *C*-statistics of 0.97, hence a sexually dimorphic blood plasma proteome signature. The differentiating proteins spanned a wide dynamic concentration range, from a few fmol/μL in Glycosylation-dependent cell adhesion molecule-1 (GLYCAM1), up to thousands in Alpha-1-antitrypsin 1–5 (SERPINA1E) and corticosteroid-binding globulin (SERPINA6). Alpha-1B-glycoprotein (A1BG) was undetectable in male animals, acting as a clear binary discriminator. We carefully investigated intracellular erythrocyte-originating proteins present in the measured plasma samples using correlation analysis and comparison to previous work^33^. It was possible to determine whether these erythrocyte-specific proteins are likely an artifact of sample processing, or a true effect of the deficiency of the gene KO.

The effect of gene KO observed in plasma ranged from no measured effect as seen in *Idh1*^*−/−*^ and *A2m*^*−/−*^ mice to a strong effect as seen in *Npc2*^*+/−*^ and *Iqgap1*^*−/−*^ mice where multiple proteins differentiated significantly compared to wild-type controls. We were able to detect changes in protein abundances in homozygous as well as heterozygous KO mice. We also carried out ORAs of the plasma protein profile of all knockouts covering protein functions, involvement in biological processes, and association with diseases. We highlighted insights from *C8a*^*−/−*^ and *Npc2*^*+/−*^ mice, where a clear plasma molecular profile was observed. Absence of C8A in *C8a*^*−/−*^ mice was confirmed by our measurement and resulted in a plasma signature associated with (impaired) complement system. The presence of Ig alpha chain C region in *C8a* knockouts highlighted how proteotyping approaches help to generate hypotheses for less characterized proteins—in this case suggesting a role in the innate immune system. Functional studies using the mice described here, or other models, are needed to test this hypothesis. Mutations in human *NPC2* leads to the development of Niemann–Pick disease type C, an autosomal recessive disorder^90,91^. Histopathological examination of various tissues from the *Npc2* KO strain confirmed the presence of disease-related phenotypes in homozygous mice, but not in heterozygous mice. However, we were able to quantify changes in the plasma proteome of *Npc2*^*+/−*^ mice. This clearly shows that proteomics is complementary to other standardized phenotyping tests. The proteomic signature detected in blood plasma of the NPC2-deficient mice was associated with cancer. Confirming previous studies that associated NPC2 levels with various cancers as measured directly by ELISA^85^, our results associated the blood plasma protein signature of NPC2 deficiency to cancer. We expect that measurement of additional tissues will provide a more comprehensive proteomics phenotype of the gene KO. Indeed, these types of studies may be developed to complement standard genetic screens to assess disease predisposition and risk, particularly for polygenic diseases or when assessing variants of unknown significance. We also compared our proteomic measurements to standard phenotyping tests relevant to plasma, including clinical chemistry, hematology, and body composition measurements. Several correlations were identified between plasma protein concentration and these biological parameters. While we focused our discussion on two KO strains, *C8a*^*−/−*^ and *Npc2*^*+/−*^, our work includes measured abundances, determined discriminating proteins, and ORAs for all 30 KO mouse strains that we studied. Our data, in conjunction with available IMPC phenotyping results, provide an enriched resource and will help researchers interested in these proteins, or the pathways and functions their absence affects, to better formulate their hypotheses and develop experiments to test them.

## Materials

### Mouse plasma samples

Plasma samples for 30 KO strains (Table 1) were obtained from The Centre for Phenogenomics, which is part of the International Mouse Phenotyping Consortium (IMPC)^58^. Samples were collected from three male and three female mice of each KO line, as well as 19 female and 19 male C57BL/6NCrl wild-type mice collected at a similar time. All sample collection was performed in the morning before noon. Whole-blood samples were collected in tubes containing heparin from the retro-orbital sinus under isoflurane anesthetic. Samples were spun at 5000*g* for 10 min at 8 °C. The plasma layer was removed, aliquoted, stored at −80 °C, shipped on dry ice to the University of Victoria, and stored again at −80 °C until analyzed. All experimental procedures on animals received approval from the Animal Care Committee of The Centre for Phenogenomics and were conducted in accordance with the guidelines of the Canadian Council on Animal Care. The corresponding license numbers are AUPs 153, 275, 277, and 279. All mutant mouse lines used for plasma proteotyping are available from the Canadian Mouse Mutant Repository (CMMR) at The Centre for Phenogenomics.

### Pathology

Wild type and homozygous mice were euthanized at 10 weeks of age, heterozygous mice were euthanized at 16 weeks of age, and a complete necropsy and comprehensive tissue collection for histopathology was done. Fresh tissues were immersion fixed in 10% neutral buffered formalin, paraffin-embedded, sectioned at 4–5 μm, and stained with HE. The tissues collected and processed from each mouse for histopathology included lung, thyroid, trachea, esophagus, heart, thymus, brown adipose tissue, mesenteric lymph node, adrenal gland, liver, spleen, kidney, urinary bladder, mammary gland, uterus, and ovary (from females) or testis, epididymiis, prostate, and seminal vesicle (from males), sternum, pancreas, skeletal muscle, salivary glands, stomach, duodenum, ileum, jejunum with Peyer’s patch, cecum, colon, rectum, eye, ear, spinal cord, brain, femur, tibia, knee joint, and skin (snout, pinna, dorsal, ventral, tail base)^92^. Histopathology evaluation was done by veterinary pathologists (H.A.A., C.M.) and images were captured using a microscope-mounted Olympus DP71 digital camera (Olympus Life Science Imaging Systems Inc., Markham, ON, Canada).

### Surrogate peptide internal standards and assays

Proteotypic peptide surrogates were selected for each protein and chemically synthesized^31^. First, surrogates were selected by in-silico using PeptidePicker^93^. For synthesis of the heavy labeled peptides, ^13^C/^15^N N-Fmoc l-arginine and l-lysine (98% isotopic enrichment, Cambridge Isotope Laboratories, Andover, MA, USA) were coupled to TentaGelTM R TRT resins (RAPP Polymere, Tübingen, Germany). For synthesis of unlabeled peptides, Wang resins preloaded with non-modified N-Fmoc lysine and arginine were purchased from Matrix Innovations (Quebec City, QC, Canada). All peptides were synthesized and purified in house^94^. Synthesis was performed using dimethylformamide with a 10× or 20× amino acid excess, using 40% piperidine for Fmoc deprotection, and HCTU(1 eq)/NMM (2 eq) as activator/base reagents. After cleaved from the resin, the synthetic peptides were purified by reverse-phase HPLC on an Onyx silica monolithic C_18_ column (100 × 10 mm id, 2 μm particles; Phenomenex; Torrance, CA, USA). The peptide elution profiles were monitored by UV absorbance at 230 nm (Ultimate 3000; Dionex; Sunnyvale, CA, USA) and the fractions of interest were measured by MALDI‐TOF‐MS using an Ultraflex III TOF/TOF mass spectrometer (Bruker Daltonik; Bremen, Germany). Fractions containing more than 80% of the target peptide were pooled and lyophilized. Each synthetic peptide was characterized by capillary zone electrophoresis (CZE) to assess the purity, and by amino acid analyses (AAA) to determine its absolute concentration. The results of CZE and AAA were later used to estimate the endogenous surrogate peptide concentration by reference to the exact amount of the spiked-in synthetic heavy labeled peptide. Peptide specific instrument parameters were characterized using an Agilent 6495 Triple Quadrupole mass spectrometer. Peptide assays were validated according to the Clinical Proteome Tumor Analysis Consortium (CPTAC) guidelines for assay characterization^32^ to assess the response curve, repeatability, selectivity, stability, and reproducible detection of endogenous peptide^31^. In total, assays measuring 375 peptide surrogates covering same number of proteins were established.

### Sample preparation and measurement

A brief explanation is included here with additional details provided in the supplementary materials. Mouse plasma samples were processed using the Tecan Evo (Männedorf, Switzerland) liquid handling robot and all 218 samples were randomized over three 96-well plates. A pooled reference plasma sample (BioReclamationIVT; Westbury, NY, USA) was used for quality control and normalization with 9–12 reference samples per plate inserted semi-randomly. Additional eight samples for establishing the standard curve were included on the first plate, and three curve quality control samples were included on each plate. Tryptic digestion and sample measurement were performed in a standardized way as detailed in Supplementary Materials. An 8-point external calibration curve was established for quantification using synthetic light peptides (ranging in concentration from 1 to 1000× assay LLOQ) spiked at known concentration into digested bovine serum albumin (Sigma Aldrich, Oakville, ON, CA) as a simplified background matrix^95^, while synthetic heavy labeled peptides were added to all samples at 100× assay LLOQ as the normalizer.

### Quantification and data analysis

Endogenous analyte concentrations were calculated from the endogenous/heavy ratio using regression analysis of the standard curves (1/*x*^2^ weighting)^96^. Raw data were processed using Skyline^97^, including inspection and correction of peak integration. This step ensures that the beginning and end of the eluted peptides are included. Normalization was performed within each plate against the pooled control sample, which were measured on each plate multiple time. If the measured concentration of a specific protein was below the assay’s LLOQ for more than half of the pooled control samples within a plate, the original reported value of each sample for that specific protein was considered more trustworthy and kept unchanged. LASSO was used for identifying the minimal set of best discriminators between KO and wild-type mice that allow best discrimination^63^. Two-sided Mann–Whitney–Wilcoxon test was used to compare protein abundances between KO and wild-type mice and *p* values were adjusted with the Benjamini–Hochberg method for multiple testing. Protein fold changes were determined by calculating the ratio of mean concentrations of KO to wild-type mice. Volcano plots were used to represent *p* values and fold change. ORAs and the required hypergeometric test were performed using the quantified proteins as a background. Entries in seven knowledgebases were used for ORA including GO^66^, MsigDB^67^, molecular pathway using KEGG^68^ and Reactome^60^, DO^69^, diseases and their gene associations using DisGeNET^70^, and MeSH for processes and diseases^71^.

All data analysis and visualization were performed using R and various libraries including ggplot^98^ for visualization, glmnet^99^ for regression and statistical analysis, and ClusterProfiler^100^ for ORA.

### Integration with standard phenotyping tests

Raw data for phenotyping tests were retrieved from the IMPC (www.mousephenotype.org) for our individual mice. Tests for integration with proteomic data included clinical chemistry (i.e. levels of sodium (mmol/L), potasium (mmol/L), chloride (mmol/L), BUN (mg/dL), creatinine (mg/dL), protein (g/L), albumin (g/L), bilirubin (mg/dL), calcium (mg/dL), phosphate (mg/dL), AST (U/L), ALT (U/L), ALP (U/L), cholesterol (mg/dL), HDL (mg/dL), triglycerol (mg/dL), glucose (mg/dL), body composition (body weight (g), fat mass (g), lean mass(g), bone mineral density—BMD (g/cm^2^), bone mineral content—BMC (g), body length (cm), BMC/weight (ratio), lean/weight (ratio), fat/weight (ratio), head weight (g), bone area (cm^2^) (BMC/BMD)), and hematology (WBC (10^3^/μL), RBC (10^6^/μL), Hgb (g/dL), HCT (%), MCV (fL), MCH (pg), MCHC (g/dL), PLT (10^3^/μL), MPV (fL), RDW %, NE %, LY %, MO %, EO %, BA %, NE (10^3^ μm/L), LY (10^3^ μm/L), MO (10^3^ μm/L), EO (10^3^ μm/L), and BA (10^3^ μm/L)). Relationships between the phenotyping tests and proteomic results were assessed using Pearson correlation coefficients.

### Reporting summary

Further information on research design is available in the Nature Research Reporting Summary linked to this article.

## Supplementary information

Supplementary Information

Supplementary Data 1

Reporting Summary

## Data Availability

All protein concentration values are available in the supplementary material file in Dataset 1.

## Code availability

Data processing and analysis methods used are described in the “Quantification and data analysis” section and are all based on publically available open source software tools and packages. No custom code or mathematical algorithms were used for the data analysis.
