# Supplementary material for: Proteotyping of knockout mouse strains reveals sex- and strain-specific signatures in blood plasma
Source: NPJ Syst Biol Appl. 2021 May 28;7:25. doi: 10.1038/s41540-021-00184-8 (PMC8163790; doi:10.1038/s41540-021-00184-8)
Supplement: Supplementary file 1 — Supplementary Information [file 41540_2021_184_MOESM1_ESM.docx]

**Supplementary information for: Proteotyping of knockout mouse strains reveals sex- and strain-specific signatures in blood plasma**

Yassene Mohammed^1, 2*^, Sarah A. Michaud^1*^, Helena Pětrošová^1^, [Juncong Yang](javascript:;)^1^, Milan Ganguly^3, 5^, David Schibli^1^, Ann M. Flenniken^3,4^, Lauryl M. J. Nutter^3,5^, Hibret A. Adissu^6^, K.C. Kent Lloyd^7^, Colin McKerlie^3,5^, Christoph H. Borchers^8-10*^

^1^University of Victoria - Genome BC Proteomics Centre, Victoria, BC, Canada

^2^Center for Proteomics and Metabolomics, Leiden University Medical Center, Leiden, Netherlands

^3^The Center for Phenogenomics, Toronto, ON, Canada

^4^Sinai Health Lunenfeld-Tanenbaum Research Institute, Toronto, ON, Canada

^5^The Hospital for Sick Children, Toronto, ON, Canada

^6^Covance Inc, Chantilly, VA, USA

^7^Department of Surgery, School of Medicine, and Mouse Biology Program, University of California, Davis, CA, USA

^8^Proteomics Centre, Segal Cancer Centre, Lady Davis Institute, Jewish General Hospital, McGill University, Montreal, Quebec, Canada

^9^Gerald Bronfman Department of Oncology, Jewish General Hospital, Montreal, Quebec, Canada

^10^Department of Data Intensive Science and Engineering, Skolkovo Institute of Science and Technology, Skolkovo Innovation Center, Nobel St., Moscow 143026, Russia

Corresponding: Yassene Mohammed ([yassene@proteincentre.com](mailto:yassene@proteincentre.com)) – ORCID: 0000-0003-3265-3332

Sarah A. Michaud (sarah@proteincentre.com) – ORCID: [0000-0001-7243-9168](https://orcid.org/0000-0001-7243-9168)

Christoph H. Borchers (christoph.borchers@mcgill.ca) – ORCID: 0000-0003-2394-6512

**Content**

|  | Page |
| --- | --- |
| **Supplementary extended description of sample preparation and measurement.** | 3 |
| **Supplementary Figure 1.** Top 10 pathways covered by the used plasma assays. | 5 |
| **Supplementary Figure 2.** A heatmap with hierarchical clustering summarizing all measured samples and determined protein concentrations. | 6 |
| **Supplementary Figure 3.** A correlation matrix of measured proteins. | 7 |
| **Supplementary Figure 4.** A screenshot showing a summary of the phenotypic tests performed on C8a^-/-^ strain. | 8 |
| **Supplementary Figure 5.** A screenshot showing a summary of the phenotypic tests performed on *Npc2^+/-^* strain. | 9 |
| **Supplementary Table 1.** Dynamic range of determined plasma protein concentrations in controls. | 10 |
| **Supplementary Table 2.** Discriminating proteins for each knockout using LASSO regression. | 15 |
| **Supplementary Table 3.** Phenotyping tests performed by IMPC on C8a^-/-^ mice used (C8a^tm1b(EUCOMM)Hmgu^) shown in Figure S4. | 17 |
| **Supplementary Table 4.** Phenotyping tests performed by IMPC on *Npc2^+/-^* mice (Npc2^tm1e.1(EUCOMM)Wtsi^) shown in Figure S5. | 22 |
| **Supplementary ORA-report 1.** Over representation analyses using discriminating proteins from Mann-Whitney-Wilcoxon test. | 30 |
| **Supplementary ORA-report 2.** Over representation analyses using combined discriminating protein lists from Mann-Whitney-Wilcoxon test and LASSO regression. | 44 |
| **Supplementary Dataset 1.** Protein concentration measured in individual samples (fmol/μl) | 74 |

**Supplementary extended description of sample preparation and measurement**

**Sample preparation**

Individual mouse plasma samples were processed using the Tecan Evo (Männedorf, Switzerland) liquid handling robot. All 218 samples were randomized over three 96 well plates for processing. Knockouts and control samples collected from same center, project and time period as the knockout samples were placed on the same plate (N = 3 males and 3 females). A pooled reference plasma sample (BioReclamationIVT; Westbury, NY, USA) was used for quality control and normalization. The pooled reference plasma was inserted semi-randomly in the sample order, with one pooled sample between every 8 samples. The plate layout was generated using in-house written software logic for sample placement with random generator. An additional 8 samples for establishing the standard curve was included on the first plate, and 3 curve quality control samples were included on each plate. Tryptic digestion was performed as previously described ^1^. Briefly, 10 µL of plasma was diluted with 20 µL of 9 M urea, 300 mM Tris pH 8.0, and 20 mM dithiothreitol, and incubated at 37 °C for 30 min. Samples were alkylated by adding 40 mM iodoacetamide and incubating for 30 min at room temperature in the dark, and subsequently diluted 10 fold in 100 mM Tris, pH 8.0. Trypsin was added at a protein:enzyme concentration ratio of 20:1, and samples were digested for 18 h overnight at 37 °C. Trypsin was inactivated by addition of formic acid to a final concentration of 1% (v/v), and the samples were spiked with synthetic stable-isotope labelled peptide mixtures. Digests were then desalted and concentrated by solid phase extraction, using OASIS HLB 96-well µElution plates with 30 µm particle size (Waters), according to manufacturer instructions. Eluted samples were lyophilized and re-suspended in 0.1% aqueous formic acid. Twenty micrograms of protein digest was injected on the LC column for each MRM-MS run.

**Liquid chromatography-mass spectrometry**

A panel of 375 surrogate peptides covering the same number of proteins was used for analysis (Figure 1, Supplementary Table 1). Samples were analyzed by scheduled MRM on an Agilent 6495 Triple Quadrupole mass spectrometer operated in positive mode, and connected to a 1290 Infinity UHPLC system via a Jet Stream ESI source (Agilent Technologies). Peptides were separated over a one-hour gradient at a flow rate of 0.4 mL/min. The mobile phases were 0.1% formic acid in water (solution A) and 0.1% formic acid in acetonitrile (solution B); the gradient was as follows (%B, time in min): 2, 0; 7, 2; 30, 50; 45, 53; 80, 53.5; 98, 56 with a 4-min equilibration (with 2% B) after each gradient. Targeted MS acquisitions were performed using 1-min detection windows, ≤900 ms cycle time, and ≥9 ms dwell times. The top performing transition was used to monitor each peptide.

**References**

1. Michaud SA*, et al.* Molecular phenotyping of laboratory mouse strains using 500 multiple reaction monitoring mass spectrometry plasma assays. *Commun Biol* **1**, 78 (2018).

**Supplementary Figure 1. Top 10 pathways covered by the used plasma assays.**

**
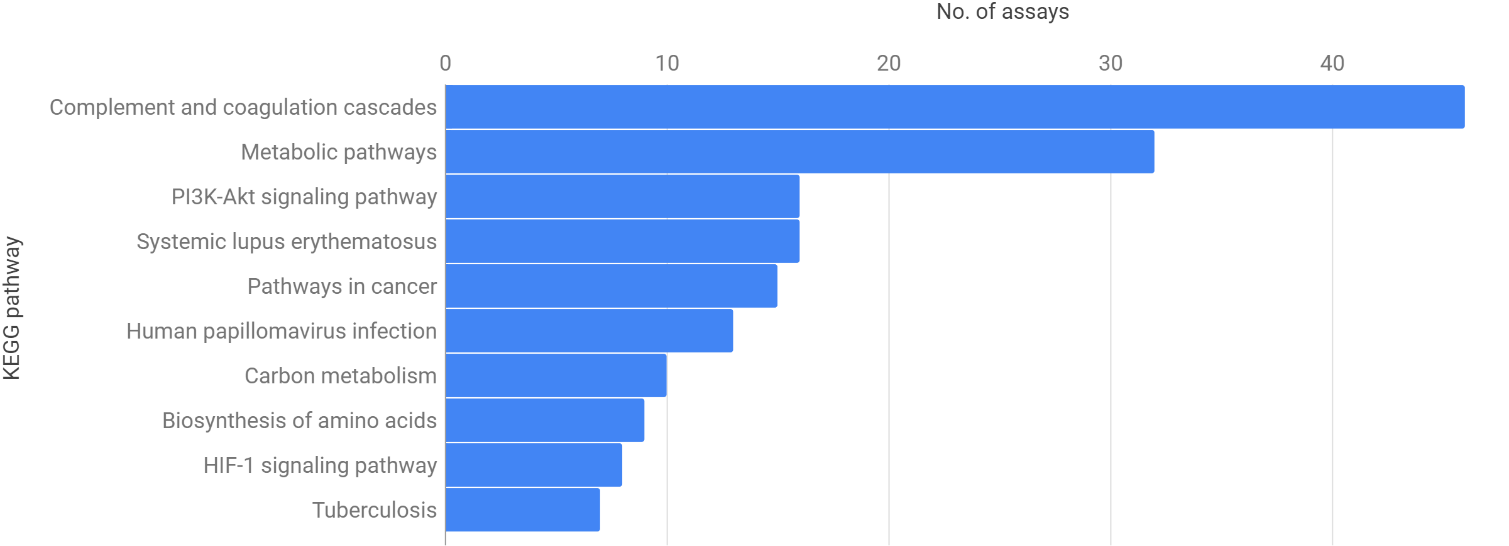
**

**Supplementary Figure 2. A heatmap with hierarchical clustering summarizing all measured samples and determined protein concentrations.** Concentration values are in z-score on blue-to-red scale. On the right a metadata panel represents project name, knocked out gene, zygosity, sex, and 96-well plate. The clear inherited discrimination in the data is between male and female as can be seen in the sex annotation.


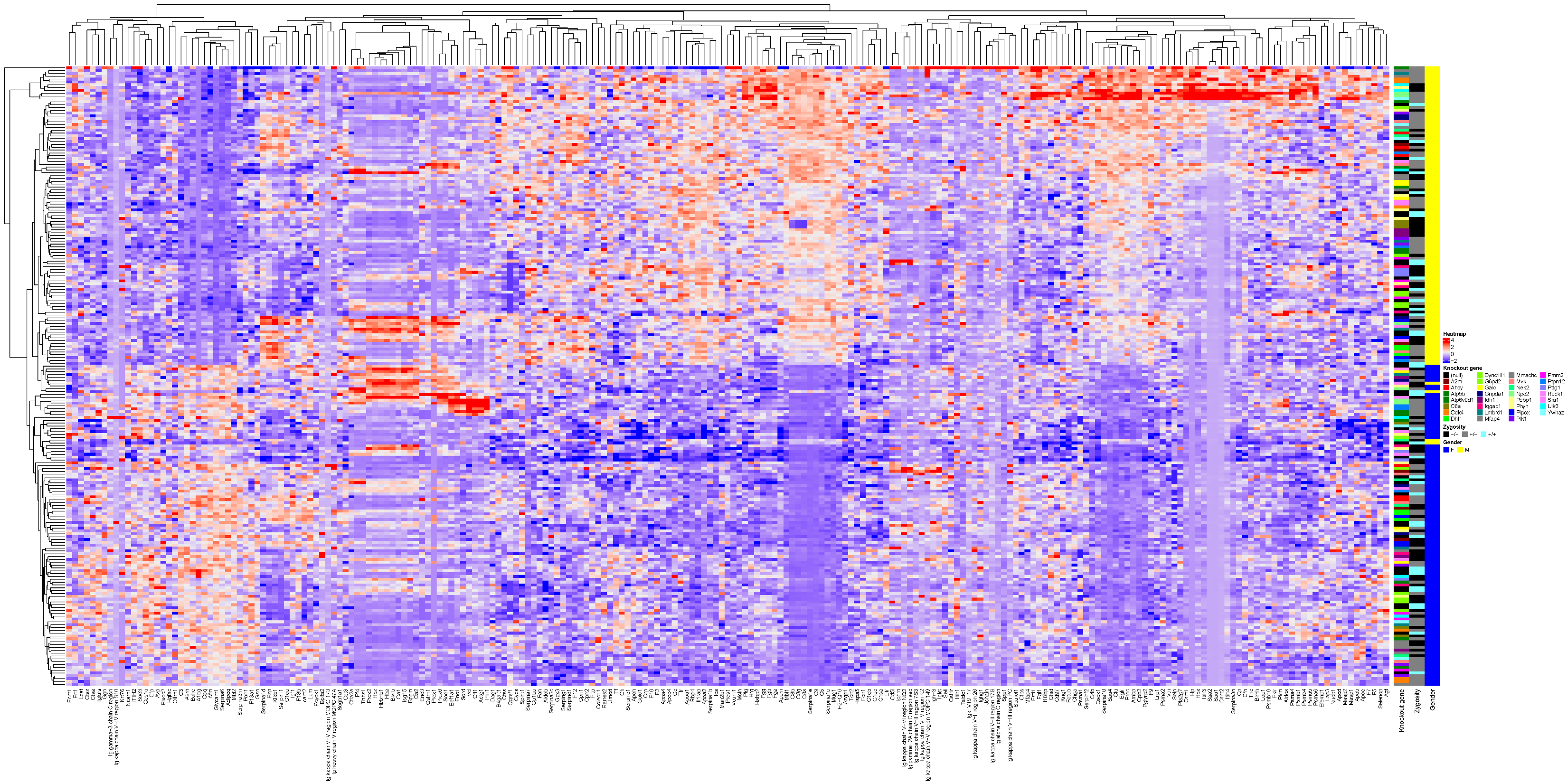


**Supplementary Figure 3. A correlation matrix of measured proteins**.


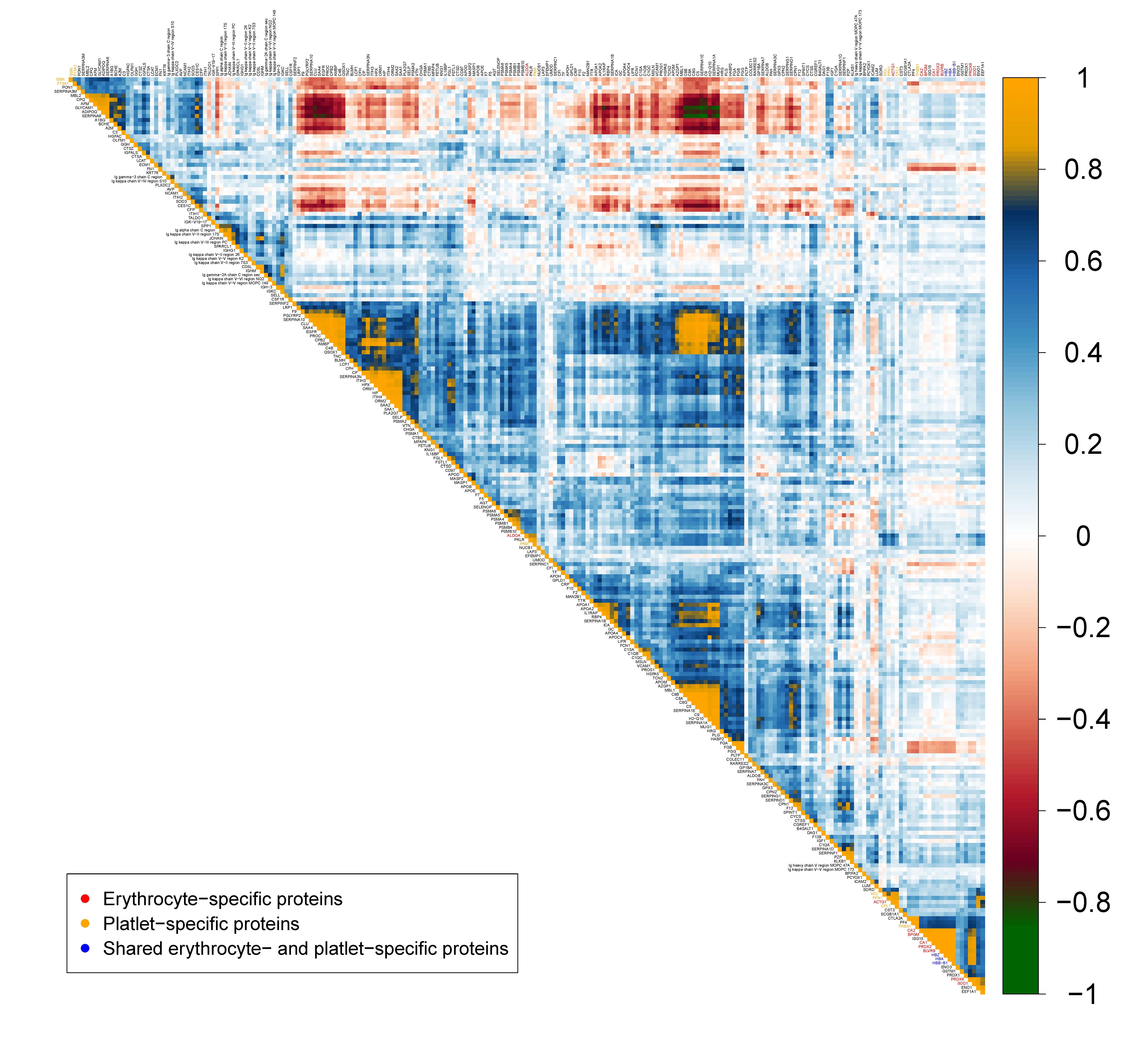


**Supplementary Figure 4. A screenshot showing a summary of the phenotypic tests performed on** [***C8a***](http://www.informatics.jax.org/allele/MGI:5637199)***^-/-^* strain**. A full list is included in supplementary Table S3 and details on the individual tests can be obtained from IMPC using the following link: www.mousephenotype.org/data/genes/MGI:2668347. The plot demonstrates the breadth of the phenotyping tests performed.

**
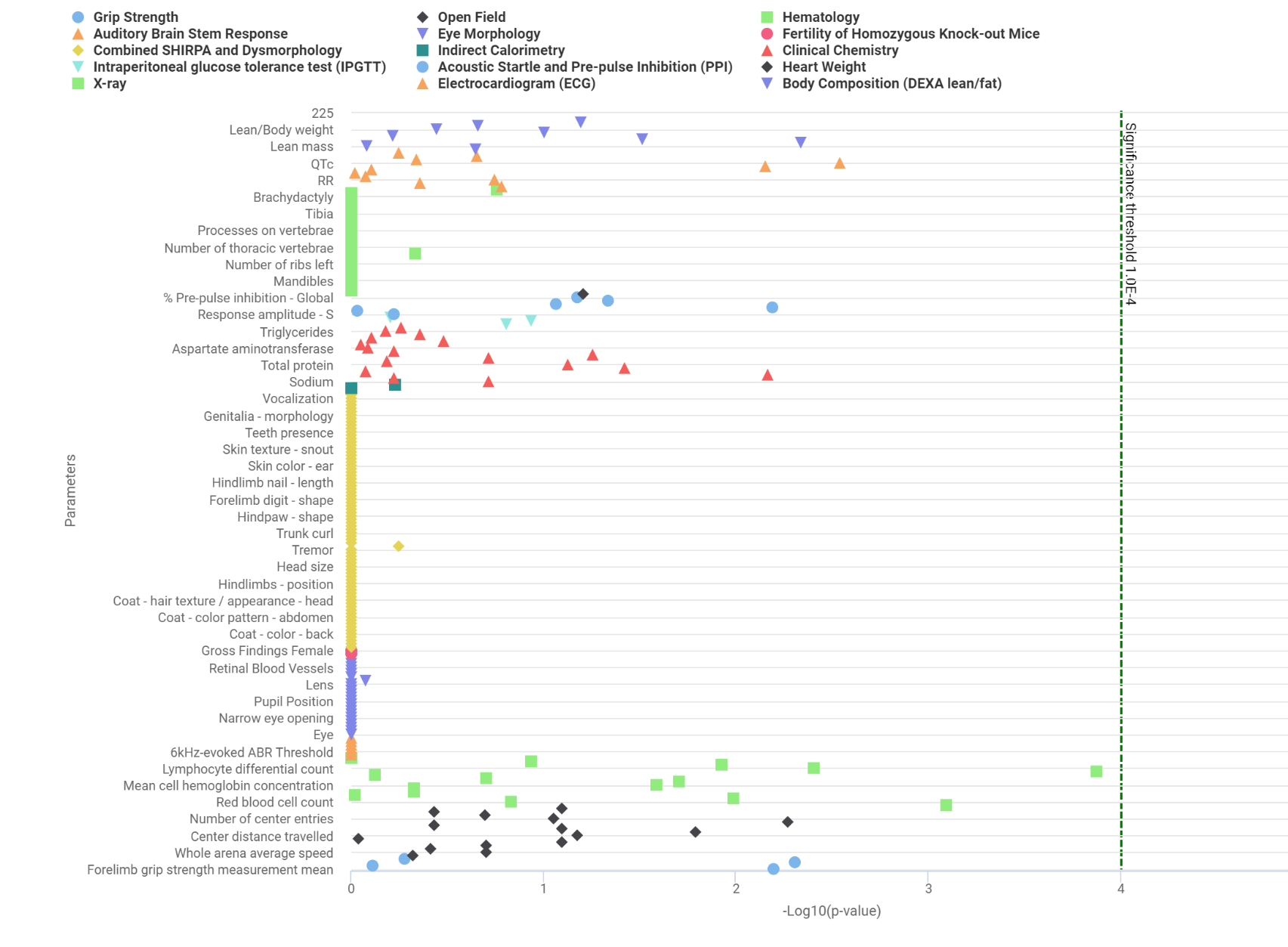
**

**Supplementary Figure 5.** **A screenshot showing a summary of the phenotypic tests performed on *Npc2^+/-^* strain.** A full list is included in supplementary Table S4 and details on the individual tests can be obtained from IMPC using the following link: www.mousephenotype.org/data/genes/MGI:1915213.

**
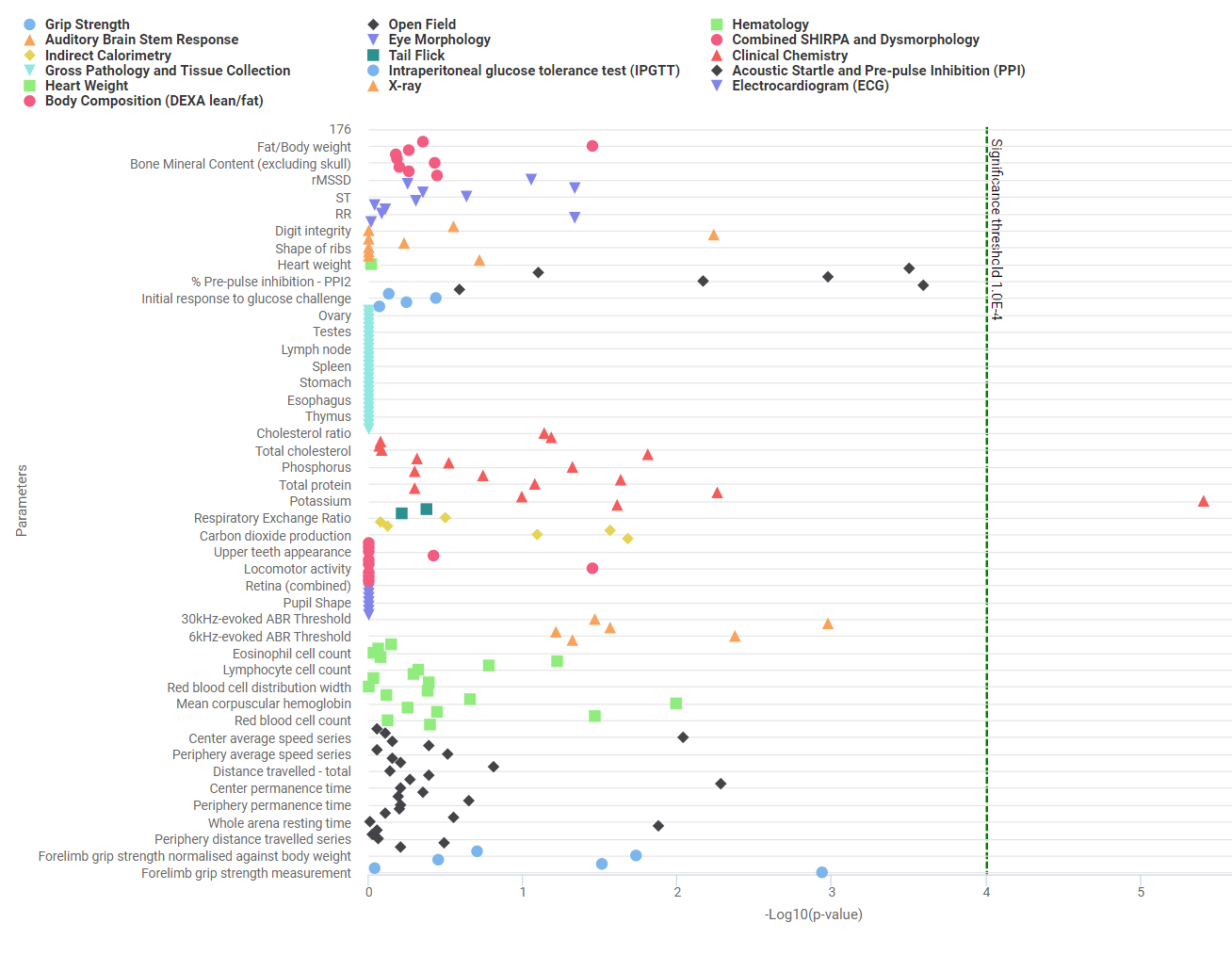
**

**Supplementary Table 1. Dynamic range of determined plasma protein concentrations in controls.**

| Protein name | Protein accession | Concentration in control mice (19 female and 19 male) | | | | | | CV in 33 pooled samples |  |
| --- | --- | --- | --- | --- | --- | --- | --- | --- | --- |
|  |  | Minimum | First quartile | Median | Mean | Third quartile | Maximum |  | |
| Sfn | O70456 | 0 | 0 | 0 | 0.324 | 0 | 3.125 | 0.247 | |
| Dag1 | Q62165 | 0.721 | 0.865 | 1.038 | 1.024 | 1.1 | 1.587 | 7.567 | |
| Lap3 | Q9CPY7 | 0.75 | 0.936 | 1.023 | 1.087 | 1.204 | 1.601 | 6.628 | |
| Prdx1 | P35700 | 0 | 0 | 0 | 1.115 | 0 | 9.942 | 1.495 | |
| Krt76 | Q3UV17 | 0 | 0 | 0 | 1.259 | 1.588 | 8.976 | 0.378 | |
| Gstm1 | P10649 | 0 | 0.791 | 1.189 | 1.427 | 1.962 | 8.395 | 5.964 | |
| Fgl1 | Q71KU9 | 0.744 | 1.264 | 1.542 | 1.527 | 1.791 | 2.498 | 10.38 | |
| Eef1a1 | P10126 | 0 | 0 | 1.109 | 1.781 | 3.075 | 13.079 | 1.913 | |
| Fah | P35505 | 0 | 0 | 2.332 | 1.855 | 3.071 | 5.587 | 2.814 | |
| Fstl1 | Q62356 | 1.291 | 1.641 | 1.831 | 1.858 | 2.081 | 2.422 | 14.246 | |
| Avp | P35455 | 0.286 | 1.259 | 1.662 | 1.871 | 2.106 | 4.499 | 8.323 | |
| Spp1 | P10923 | 1.143 | 1.685 | 1.915 | 2.029 | 2.397 | 3.128 | 9.11 | |
| Cgref1 | Q8R1U2 | 0 | 0 | 2.45 | 2.035 | 3.246 | 4.961 | 1.436 | |
| Bpgm | P15327 | 0 | 0 | 0.617 | 2.052 | 3.775 | 9.652 | 8.25 | |
| Icam2 | P35330 | 1.387 | 1.804 | 2.046 | 2.083 | 2.336 | 2.905 | 14.584 | |
| Psmb10 | O35955 | 0.562 | 1.818 | 2.12 | 2.181 | 2.655 | 3.703 | 4.151 | |
| Prdx6 | O08709 | 0.766 | 1.183 | 1.703 | 2.234 | 2.815 | 11.313 | 8.596 | |
| Tnc | Q80YX1 | 0.655 | 1.679 | 2.211 | 2.271 | 2.862 | 3.968 | 3.056 | |
| Selp | Q01102 | 0 | 1.931 | 2.691 | 2.375 | 3.162 | 4.559 | 5.74 | |
| Taldo1 | Q93092 | 1.511 | 1.985 | 2.322 | 2.615 | 2.778 | 7.258 | 5.481 | |
| Lrp1 | Q91ZX7 | 1.856 | 2.581 | 2.848 | 2.9 | 3.278 | 3.793 | 7.231 | |
| Ctsa | P16675 | 2.172 | 2.556 | 2.931 | 2.994 | 3.37 | 4.018 | 9.431 | |
| Eno1 | P17182 | 1.533 | 2.193 | 2.729 | 3.135 | 3.716 | 10.127 | 9.358 | |
| Ig kappa chain V-V region MOPC 173 | P01643 | 0.947 | 1.598 | 2.399 | 3.269 | 3.439 | 18.656 | 14.63 | |
| Eno3 | P21550 | 0 | 0 | 2.908 | 3.361 | 4.296 | 20.91 | 5.826 | |
| Glycam1 | Q02596 | 0 | 0.856 | 2.786 | 3.369 | 5.629 | 8.367 | 5.391 | |
| Msln | Q61468 | 2.271 | 2.985 | 3.449 | 3.452 | 3.859 | 5.593 | 6.115 | |
| Sod1 | P08228 | 1.28 | 1.98 | 2.615 | 3.604 | 4.576 | 16.438 | 7.606 | |
| Ctsd | P18242 | 2.412 | 3.282 | 3.737 | 3.675 | 4.064 | 4.941 | 18.103 | |
| Cd97 | Q9Z0M6 | 2.432 | 3.538 | 4.023 | 4.013 | 4.655 | 5.702 | 7.337 | |
| Aldob | Q91Y97 | 0 | 3.388 | 4.375 | 4.038 | 4.926 | 8.89 | 8.347 | |
| Mfap4 | Q9D1H9 | 2.375 | 3.622 | 4.166 | 4.361 | 4.861 | 6.699 | 6.329 | |
| Spint1 | Q9R097 | 3.526 | 3.959 | 4.619 | 4.673 | 5.274 | 6.654 | 8.725 | |
| B4galt1 | P15535 | 2.577 | 3.978 | 4.593 | 4.99 | 6.118 | 8.651 | 4.831 | |
| Isg15 | Q64339 | 1.8 | 2.408 | 3.695 | 5.005 | 7.542 | 16.361 | 8.742 | |
| Rarres2 | Q9DD06 | 3.104 | 4.471 | 4.989 | 5.011 | 5.615 | 6.926 | 6.569 | |
| Vcl | Q64727 | 3.111 | 4.123 | 5.092 | 5.225 | 6.078 | 7.704 | 6.823 | |
| Ggh | Q9Z0L8 | 3.638 | 4.65 | 5.137 | 5.301 | 5.83 | 7.794 | 7.856 | |
| Cfl1 | P18760 | 2.018 | 3.699 | 5.158 | 5.372 | 6.633 | 13.059 | 6.466 | |
| Ca1 | P13634 | 0 | 3.061 | 4.978 | 6.384 | 9.221 | 22.298 | 8.231 | |
| Blvrb | Q923D2 | 1.583 | 2.702 | 4.65 | 6.521 | 7.28 | 28.152 | 6.146 | |
| Ctss | O70370 | 4.675 | 5.976 | 6.521 | 6.692 | 7.484 | 9.718 | 11.129 | |
| Il18bp | Q9Z0M9 | 4.483 | 6.072 | 6.619 | 6.806 | 7.121 | 13.537 | 11.191 | |
| Sord | Q64442 | 4.25 | 6.497 | 7.426 | 7.832 | 8.75 | 14.336 | 7.186 | |
| Psma2 | P49722 | 5.167 | 6.582 | 7.643 | 7.877 | 8.603 | 11.483 | 6.438 | |
| Psmb1 | O09061 | 4.492 | 7.022 | 7.686 | 7.929 | 8.278 | 12.97 | 5.795 | |
| Lcp1 | Q61233 | 6.253 | 7.154 | 7.976 | 8.238 | 8.799 | 13.018 | 9.684 | |
| Psma1 | Q9R1P4 | 0 | 7.358 | 9.089 | 8.255 | 10.439 | 13.846 | 4.077 | |
| Olfm1 | O88998 | 4.852 | 6.783 | 8.214 | 8.345 | 9.33 | 13.337 | 5.043 | |
| Ctla2a | P12399 | 0 | 5.557 | 6.853 | 8.466 | 9.563 | 29.117 | 6.746 | |
| Nucb1 | Q02819 | 5.159 | 7.117 | 8.569 | 8.633 | 9.729 | 14.74 | 7.623 | |
| F9 | P16294 | 4.437 | 7.24 | 8.288 | 8.641 | 10.227 | 13.007 | 9.176 | |
| Scgb1a1 | Q06318 | 5.475 | 6.65 | 8.622 | 8.788 | 9.823 | 20.045 | 8.22 | |
| Colec11 | Q3SXB8 | 5.471 | 7.096 | 9.03 | 8.798 | 10.207 | 12.76 | 5.144 | |
| Saa2 | P05367 | 0 | 0 | 1.722 | 8.887 | 8.768 | 137.95 | 12.414 | |
| Hspa5 | P20029 | 0 | 6.052 | 8.611 | 8.926 | 11.719 | 18.619 | 3.361 | |
| Ctsz | Q9WUU7 | 4.785 | 7.854 | 8.334 | 8.957 | 9.953 | 14.667 | 4.587 | |
| Sparcl1 | P70663 | 6.772 | 8.09 | 9.583 | 9.304 | 10.383 | 12.253 | 15.458 | |
| Klkb1 | P26262 | 3.566 | 5.834 | 9.055 | 9.369 | 12.696 | 19.853 | 13.145 | |
| Man2b1 | O09159 | 5.758 | 8.109 | 9.897 | 9.716 | 11.296 | 14.356 | 3.967 | |
| Ncam1 | P13595 | 5.066 | 8.683 | 9.766 | 9.906 | 10.667 | 15.461 | 5.661 | |
| Chga | P26339 | 5.658 | 8.811 | 10.069 | 10.118 | 11.708 | 14.512 | 5.396 | |
| Pcyox1 | Q9CQF9 | 0 | 9.024 | 10.575 | 10.209 | 11.82 | 16.606 | 3.386 | |
| Pfn1 | P62962 | 0 | 7.2 | 10.533 | 10.365 | 13.922 | 30.547 | 2.781 | |
| Serpina3c | P29621 | 6.982 | 8.946 | 10.511 | 10.738 | 11.834 | 17.543 | 5.115 | |
| Umod | Q91X17 | 7.644 | 9.509 | 11.116 | 10.91 | 12.089 | 15.526 | 6.758 | |
| Psma6 | Q9QUM9 | 7.3 | 9.844 | 10.789 | 10.914 | 11.909 | 15.87 | 8.367 | |
| A2m | Q6GQT1 | 0 | 6.4 | 7.918 | 11.301 | 16.055 | 28.475 | 10.452 | |
| Plxdc2 | Q9DC11 | 8.883 | 10.176 | 11.747 | 11.748 | 12.743 | 18.847 | 6.637 | |
| Prdx2 | Q61171 | 0 | 4.91 | 8.313 | 12.09 | 16.53 | 50.295 | 7.069 | |
| Psma5 | Q9Z2U1 | 6.112 | 10.561 | 12.21 | 12.187 | 13.467 | 19.247 | 4.996 | |
| Blmh | Q8R016 | 7.933 | 10.747 | 11.795 | 12.197 | 13.497 | 18.854 | 7.505 | |
| Ctbs | Q8R242 | 9.586 | 11.405 | 12.15 | 12.2 | 12.928 | 16.298 | 9.254 | |
| Pla2g7 | Q60963 | 7.86 | 10.261 | 11.964 | 12.556 | 14.627 | 19.935 | 7.507 | |
| Psmb4 | P99026 | 7.265 | 10.765 | 12.851 | 12.684 | 14.018 | 20.022 | 5.883 | |
| C1qa | P98086 | 3.293 | 9.421 | 13.29 | 12.889 | 14.941 | 22.622 | 7.387 | |
| Vcam1 | P29533 | 9.775 | 12.375 | 13.766 | 14.021 | 15.387 | 19.26 | 11.024 | |
| Masp2 | Q91WP0 | 10.268 | 12.415 | 14.085 | 14.117 | 15.779 | 18.7 | 1.546 | |
| Psma4 | Q9R1P0 | 8.636 | 12.309 | 13.828 | 14.195 | 15.485 | 22.63 | 7.697 | |
| Masp1 | P98064 | 10.429 | 12.869 | 14.646 | 14.585 | 16.226 | 18.423 | 14.996 | |
| Cycs | P62897 | 10.763 | 14.459 | 15.733 | 16.366 | 17.265 | 27.629 | 3.505 | |
| Igf1 | P05017 | 11.488 | 13.899 | 16.45 | 16.837 | 19.442 | 24.985 | 8.528 | |
| Pklr | P53657 | 11.54 | 14.442 | 16.287 | 18.647 | 19.771 | 39.175 | 7.409 | |
| Saa1 | P05366 | 0 | 6.65 | 9.947 | 18.693 | 15.98 | 191.65 | 14.554 | |
| Aldoa | P05064 | 10.344 | 15.7 | 18.934 | 20.208 | 22.369 | 42.118 | 9.227 | |
| Adipoq | Q60994 | 8.575 | 13.473 | 17.255 | 20.3 | 26.341 | 37.957 | 6.891 | |
| Bche | Q03311 | 11.875 | 17.017 | 18.695 | 20.589 | 23.934 | 34.021 | 8.594 | |
| Habp2 | Q8K0D2 | 15.627 | 18.001 | 21.093 | 20.638 | 22.433 | 26.883 | 11.605 | |
| Serpina7 | P61939 | 7.2 | 10.989 | 15.694 | 20.749 | 28.677 | 54.351 | 8.841 | |
| Apod | P51910 | 11.044 | 17.909 | 21.224 | 21.549 | 25.012 | 32.505 | 9.706 | |
| Pkm | P52480 | 11.363 | 17.308 | 20.287 | 22.041 | 25.045 | 46.431 | 6.864 | |
| Ig kappa chain V-III region PC | P01674 | 0 | 10.586 | 17.892 | 23.501 | 27.616 | 133.04 | 5.284 | |
| Pros1 | Q08761 | 17.314 | 21.805 | 23.124 | 23.524 | 25.24 | 31.028 | 6.682 | |
| Cfh | P06909 | 13.061 | 21.133 | 25.189 | 24.562 | 27.982 | 34.266 | 7.464 | |
| Egfr | Q01279 | 7.943 | 15.653 | 24.437 | 25.645 | 32.98 | 56.079 | 10.897 | |
| Sell | P18337 | 18.323 | 23.798 | 25.646 | 26.117 | 28.152 | 34.501 | 8.223 | |
| Ig kappa chain V-II region 17S | P03976 | 11.511 | 16.587 | 21.855 | 26.193 | 30.971 | 110.46 | 5.474 | |
| F7 | P70375 | 17.279 | 24.14 | 26.921 | 26.831 | 28.907 | 41.065 | 8.887 | |
| Bpifa2 | P07743 | 7.897 | 16.532 | 25.641 | 29.259 | 35.307 | 107.6 | 8.196 | |
| Thbs1 | P35441 | 0 | 11.668 | 16.061 | 30.037 | 32.08 | 132.8 | 17.253 | |
| Gp1ba | O35930 | 1.956 | 26.803 | 31.883 | 32.357 | 37.524 | 53.453 | 7.372 | |
| Ecm1 | Q61508 | 23.544 | 28.294 | 31.647 | 32.781 | 36.901 | 46.039 | 8.856 | |
| Proc | P33587 | 17.772 | 25.597 | 31.503 | 33.43 | 41.316 | 55.895 | 9.717 | |
| Cst3 | P21460 | 19.767 | 28.279 | 34.239 | 34.008 | 38.391 | 52.066 | 7.396 | |
| F13b | Q07968 | 16.735 | 28.805 | 33.69 | 34.403 | 38.026 | 56.612 | 6.426 | |
| Cpq | Q9WVJ3 | 22.7 | 28.173 | 34.402 | 35.611 | 42.961 | 53.654 | 11.268 | |
| F5 | O88783 | 31.322 | 34.255 | 37.013 | 38.352 | 41.689 | 52.992 | 9.798 | |
| Ig alpha chain C region | P01878 | 16.327 | 20.646 | 26.297 | 38.831 | 43.386 | 116.64 | 0.726 | |
| Sod3 | O09164 | 25.706 | 33.925 | 39.644 | 39.472 | 43.376 | 57.635 | 10.146 | |
| Tcn2 | O88968 | 24.351 | 38.017 | 41.572 | 41.685 | 47.328 | 53.362 | 6.998 | |
| C5 | P06684 | 24.191 | 27.83 | 40.544 | 46.562 | 64.129 | 84.452 | 9.063 | |
| Ig kappa chain V-V region K2 | P01635 | 9.399 | 27.594 | 39.764 | 48.448 | 47.781 | 259.07 | 7.997 | |
| Qsox1 | Q8BND5 | 32.334 | 42.234 | 47.41 | 48.694 | 51.882 | 71.771 | 12.514 | |
| Csf1r | P09581 | 37.419 | 48.147 | 53.167 | 54.341 | 57.441 | 91.778 | 11.224 | |
| Lum | P51885 | 33.036 | 47.87 | 56.456 | 57.904 | 67.246 | 91.747 | 8.97 | |
| Ambp | Q07456 | 33.253 | 46.042 | 55.078 | 59.159 | 72.003 | 104.4 | 7.611 | |
| Orm2 | P07361 | 12.891 | 25.882 | 47.403 | 59.432 | 73.898 | 325.95 | 19.438 | |
| Efemp1 | Q8BPB5 | 49.369 | 61.36 | 67.852 | 69.189 | 76.558 | 94.08 | 6.232 | |
| Igfals | P70389 | 47.652 | 60.34 | 69.182 | 72.713 | 82.157 | 112.07 | 9.227 | |
| Ca2 | P00920 | 36.563 | 57.065 | 70.079 | 74.379 | 86.928 | 167.75 | 9.276 | |
| Serpina10 | Q8R121 | 40.1 | 55.44 | 78.086 | 82.15 | 106.585 | 131.66 | 8.922 | |
| Serpinf1 | P97298 | 36.056 | 56.788 | 76.76 | 85.965 | 115.735 | 157.03 | 3.98 | |
| Ig kappa chain V-II region 7S3 | P01630 | 24.996 | 39.661 | 53.73 | 86.159 | 96.915 | 659.9 | 6.693 | |
| Pf4 | Q9Z126 | 3.723 | 19.782 | 39.781 | 87.355 | 97.145 | 463.94 | 10.734 | |
| C1sa | Q8CG14 | 52.585 | 78.555 | 88.037 | 89.247 | 105.293 | 122.51 | 7.473 | |
| Hgfac | Q9R098 | 67.728 | 80.199 | 86.779 | 89.49 | 97.665 | 118.48 | 10.167 | |
| Ig kappa chain V-V region MOPC 149 | P01636 | 27.43 | 42.412 | 67.562 | 92.972 | 109.5 | 497.16 | 7.53 | |
| Ig kappa chain V-VI region NQ2 | P04945 | 17.852 | 35.374 | 49.77 | 96.386 | 76.52 | 994.06 | 14.796 | |
| Pltp | P55065 | 50.929 | 85.819 | 99.258 | 99.996 | 116.535 | 148.42 | 9.56 | |
| Ig kappa chain V-II region 26 | P01631 | 52.128 | 63.906 | 87.542 | 100.781 | 107.595 | 294.16 | 12.014 | |
| Crp | P14847 | 71.981 | 91.346 | 99.464 | 101.512 | 111.588 | 143.03 | 10.135 | |
| Actg1 | P63260 | 62.805 | 89.51 | 100.6 | 105.959 | 125.263 | 149.62 | 8.779 | |
| Cpb2 | Q9JHH6 | 67.356 | 83.218 | 103.77 | 106.801 | 122.267 | 178.9 | 14.795 | |
| Cd5l | Q9QWK4 | 55.34 | 65.981 | 91.736 | 109.396 | 125.213 | 287.79 | 9.802 | |
| Pglyrp2 | Q8VCS0 | 69.595 | 91.071 | 113.89 | 115.897 | 141.175 | 178.74 | 14.486 | |
| Agt | P11859 | 87.566 | 105.555 | 117.685 | 116.644 | 125.897 | 156.82 | 11.514 | |
| F13a1 | Q8BH61 | 70.84 | 104.703 | 115.29 | 119.988 | 139.32 | 181.38 | 5.073 | |
| Fn1 | P11276 | 19.561 | 68.559 | 113.33 | 121.095 | 179.28 | 240.43 | 6.682 | |
| Fcn1 | O70165 | 88.009 | 110.05 | 119.92 | 122.45 | 134.15 | 166.01 | 8.916 | |
| Selenop | P70274 | 112.65 | 137.222 | 148.48 | 148.388 | 158.59 | 178 | 17.922 | |
| Lcat | P16301 | 111.26 | 128.965 | 145.225 | 150.413 | 162.037 | 227.1 | 10.592 | |
| Ttr | P07309 | 104.3 | 129.697 | 145.27 | 159.089 | 187.8 | 237.25 | 7.381 | |
| Cpn1 | Q9JJN5 | 126.89 | 161.54 | 174.06 | 172.434 | 182.61 | 206.54 | 21.824 | |
| Ig heavy chain V region MOPC 47A | P01786 | 78.568 | 115.897 | 168.235 | 182.223 | 231.02 | 365.76 | 16.262 | |
| F10 | O88947 | 151.2 | 193.58 | 218.755 | 216.793 | 232.135 | 279.48 | 10.526 | |
| Itih1 | Q61702 | 137.29 | 185.515 | 206.145 | 218.963 | 236.765 | 380.46 | 7.995 | |
| Gpld1 | O70362 | 124.84 | 204.282 | 231.715 | 228.038 | 245.235 | 391.9 | 9.899 | |
| C1qc | Q02105 | 41.212 | 202.933 | 247.98 | 235.449 | 273.572 | 362.68 | 6.868 | |
| Mbl1 | P39039 | 151.96 | 189.555 | 216.64 | 237.731 | 278.235 | 467.47 | 10.44 | |
| Lifr | P42703 | 115.93 | 172.352 | 251.3 | 246.661 | 308.303 | 408.37 | 11.52 | |
| Apob | E9Q414 | 159.75 | 210.215 | 234.76 | 248.603 | 287.212 | 378.37 | 7.749 | |
| Il1rap | Q61730 | 132.92 | 186.02 | 218.245 | 253.207 | 332.757 | 386.96 | 14.598 | |
| Jchain | P01592 | 109.22 | 166.24 | 223.865 | 257.213 | 305.777 | 790.52 | 9.426 | |
| Afm | O89020 | 167.9 | 217.007 | 279.24 | 265.497 | 311.522 | 365.21 | 15.41 | |
| Cfp | P11680 | 173.21 | 239.007 | 277.265 | 272.309 | 306.892 | 353.28 | 14.999 | |
| Serpind1 | P49182 | 189.05 | 237.745 | 272.265 | 276.446 | 295.467 | 403.98 | 5.461 | |
| Pon1 | P52430 | 137 | 253.405 | 283.72 | 280.494 | 311.505 | 380.62 | 9.608 | |
| Ig kappa chain V-IV region S10 | P01680 | 48.351 | 73.119 | 116.285 | 295.554 | 136.555 | 6899.8 | 8.937 | |
| C8a | Q8K182 | 61.776 | 133.458 | 258.885 | 311.506 | 459.055 | 716.89 | 11.121 | |
| C8g | Q8VCG4 | 70.294 | 125.545 | 250.11 | 317.857 | 507.385 | 717.55 | 12.102 | |
| A1bg | Q19LI2 | 0 | 0 | 85.035 | 348.77 | 588.183 | 2050.6 | 8.076 | |
| C1qb | P14106 | 56.79 | 275.618 | 347.415 | 351.422 | 420.855 | 539.58 | 6.171 | |
| Igk-V19-17 | P01633 | 94.963 | 177.085 | 266.625 | 354.41 | 400.257 | 1382.5 | 12.561 | |
| Serpina3m | Q03734 | 98.232 | 292.163 | 374.81 | 366.751 | 439.2 | 594.96 | 8.004 | |
| Ica | Q9DBD0 | 265.43 | 324.915 | 380.57 | 374.897 | 429.017 | 521.5 | 12.633 | |
| C8b | Q8BH35 | 110.56 | 151.965 | 334.06 | 414.892 | 679.44 | 894.59 | 6.296 | |
| Apoc4 | Q61268 | 176.45 | 329.245 | 415.405 | 419.426 | 504.835 | 739.12 | 9.462 | |
| Itih3 | Q61704 | 256.09 | 374.587 | 406.775 | 423.232 | 471.627 | 670.82 | 13.375 | |
| Itih4 | A6X935 | 241.43 | 397.865 | 425.595 | 432.768 | 475.125 | 617.82 | 18.929 | |
| Azgp1 | Q64726 | 302.44 | 392.973 | 415.91 | 433.598 | 467.085 | 571.68 | 17.554 | |
| Rbp4 | Q00724 | 220.79 | 291.34 | 369.025 | 436.226 | 573.533 | 783.08 | 8.005 | |
| Cfi | Q61129 | 302.69 | 394.017 | 438.605 | 437.245 | 490.58 | 611.72 | 9.959 | |
| Serpinf2 | Q61247 | 376.48 | 454.64 | 509.415 | 505.04 | 560.185 | 616.29 | 6.246 | |
| Apom | Q9Z1R3 | 348.01 | 470.277 | 507.3 | 512.022 | 552.648 | 685.84 | 20.78 | |
| ITIH2 | O02668 | 368.05 | 449.048 | 528.685 | 523.006 | 577.515 | 700.94 | 10.504 | |
| Serpina3n | Q91WP6 | 358.57 | 437.05 | 486.795 | 524.312 | 601.878 | 764.53 | 8.594 | |
| Cpn2 | Q9DBB9 | 389.9 | 506.04 | 540.24 | 549.961 | 569.992 | 764.6 | 10.338 | |
| Serping1 | P97290 | 412.43 | 498.062 | 546.9 | 561.419 | 636.57 | 747.97 | 3.638 | |
| H2-Q10 | P01898 | 294.48 | 396.175 | 546.03 | 562.895 | 719.19 | 898.53 | 12.783 | |
| C4b | P01029 | 362.25 | 459.457 | 547.77 | 575.912 | 679.565 | 916.79 | 9.643 | |
| C9 | P06683 | 159.49 | 202.947 | 408.435 | 582.707 | 873.48 | 1402.1 | 10.568 | |
| Gpx3 | P46412 | 481.24 | 595.62 | 648.045 | 653.282 | 717.065 | 828.22 | 9.344 | |
| F12 | Q80YC5 | 577.07 | 709.735 | 762.405 | 781.617 | 835.685 | 1103.5 | 9.968 | |
| Fetub | Q9QXC1 | 655.88 | 720.048 | 765.905 | 788.427 | 832.957 | 1095.6 | 16.29 | |
| Vtn | P29788 | 552.48 | 743.365 | 823.915 | 824.404 | 933.445 | 1076 | 8.112 | |
| Hp | Q61646 | 10.833 | 31.381 | 87.066 | 826.471 | 506.345 | 8341.9 | 6.999 | |
| Cp | Q61147 | 643.05 | 788.185 | 847.805 | 843.182 | 901.028 | 1066.5 | 21.381 | |
| Hrg | Q9ESB3 | 655 | 743.503 | 860.59 | 873.309 | 954.91 | 1176.1 | 10.945 | |
| Saa4 | P31532 | 313.25 | 500.462 | 689.395 | 911.912 | 1316.65 | 2501.8 | 7.258 | |
| F2 | P19221 | 725.55 | 902.525 | 977.75 | 980.246 | 1060.825 | 1318.6 | 8.364 | |
| Ig gamma-3 chain C region | P03987 | 222.3 | 675.082 | 917.83 | 1091.676 | 1442.025 | 2697.5 | 9.99 | |
| Serpina6 | Q06770 | 510.75 | 707.675 | 1036.33 | 1120.068 | 1524.375 | 2060.9 | 14.286 | |
| Gsn | P13020 | 772.88 | 1053.275 | 1168.95 | 1159.702 | 1232.05 | 1562.9 | 15.276 | |
| Orm1 | Q60590 | 546.11 | 966.433 | 1235.65 | 1251.605 | 1418.15 | 2279.4 | 10.192 | |
| C3 | P01027 | 788.24 | 1136 | 1357.5 | 1396.679 | 1689.8 | 2090.4 | 9.225 | |
| Plg | P20918 | 1064.8 | 1340.4 | 1442.85 | 1459.489 | 1605.975 | 1744.6 | 13.288 | |
| Clu | Q06890 | 923.03 | 1052.6 | 1247.65 | 1490.444 | 1946.075 | 2388.6 | 10.412 | |
| Hpx | Q91X72 | 889.36 | 1342.725 | 1586.45 | 1677.548 | 1956.65 | 3085.5 | 9.932 | |
| Serpinc1 | P32261 | 1048.9 | 1765.45 | 1870 | 1882.205 | 2009.3 | 2518 | 11.828 | |
| Hbb-b1 | P02088 | 308.24 | 644.683 | 1447.75 | 1989.891 | 3069.775 | 7333.6 | 19.002 | |
| Apoe | P08226 | 1224.4 | 1754.425 | 1997.9 | 2047.116 | 2304.35 | 3293.1 | 8.076 | |
| Serpina1e | Q00898 | 29.762 | 140.768 | 1474.1 | 2298.309 | 4320.625 | 6200.7 | 10.978 | |
| Apoh | Q01339 | 1964.9 | 2442.175 | 2628.3 | 2650.195 | 2869.625 | 3301.4 | 13.472 | |
| Gc | P21614 | 1882.5 | 2377.225 | 2569.8 | 2654.021 | 2909.7 | 3816.6 | 8.711 | |
| Ighg1 | P01869 | 741.35 | 1742.95 | 2404.45 | 2680.885 | 3041.575 | 7569.6 | 11.907 | |
| Ig gamma-2A chain C region sec | P01864 | 241.52 | 894.753 | 1534.2 | 2690.59 | 2446.2 | 14023 | 9.478 | |
| Igh-3 | P01867 | 542.86 | 1501.55 | 2638.4 | 2798.006 | 3417.5 | 9503.8 | 16.696 | |
| Ces1c | P23953 | 1982.1 | 2550.475 | 2979.6 | 3098.579 | 3655.925 | 5437.7 | 8.192 | |
| Apoa4 | P06728 | 1754.1 | 2722.5 | 3163.2 | 3164.576 | 3567.125 | 4488.7 | 10.097 | |
| Ighm | P01872 | 1185 | 1756.6 | 2309.9 | 3185.463 | 3822.2 | 11343 | 6.654 | |
| Serpina1d | Q00897 | 2388 | 3226 | 3512.85 | 3529.332 | 3792.425 | 4546 | 15.753 | |
| Mbl2 | P41317 | 2400.8 | 3024.2 | 3584.85 | 3762.271 | 4554.975 | 5368.8 | 12.764 | |
| Kng1 | O08677 | 3609.3 | 4324.625 | 4710.75 | 4632.729 | 4916.325 | 5703.8 | 10.683 | |
| Igkc | P01837 | 2088.8 | 2753.425 | 4615.25 | 4933.85 | 6481.775 | 11999 | 15.145 | |
| Mug1 | P28665 | 1930.4 | 3111.95 | 4415.15 | 5106.184 | 6869.425 | 9528.3 | 12.957 | |
| Hbz | P06467 | 624.67 | 1855.525 | 3599.8 | 5302.005 | 8921.375 | 16592 | 10.353 | |
| Hba | P01942 | 598.42 | 1645.725 | 4009.35 | 5368.007 | 7942.275 | 18066 | 9.092 | |
| Fgb | Q8K0E8 | 2303.2 | 4159.425 | 5092.8 | 5378.568 | 6690.1 | 9410.3 | 10.226 | |
| Fgg | Q8VCM7 | 2679.2 | 4096.425 | 5323.05 | 5480.224 | 6696.45 | 9863.6 | 7.907 | |
| Serpina1b | P22599 | 5223.7 | 6576.75 | 7090.1 | 7404.368 | 8594.425 | 9837.9 | 13.862 | |
| Fga | E9PV24 | 3299.2 | 6317.85 | 7263.2 | 7725.832 | 8821.675 | 16362 | 8.987 | |
| Pzp | Q61838 | 5869.7 | 7795.75 | 8291.7 | 8402.979 | 9240.825 | 10841 | 20.432 | |
| Tf | Q921I1 | 10370 | 12276 | 12888.5 | 13237.842 | 14445 | 17233 | 15.391 | |
| Serpina1a | P07758 | 9753.8 | 14913 | 19522 | 20233.942 | 24759.25 | 35575 | 8.736 | |
| Apoa1 | Q00623 | 18604 | 22674.5 | 26410 | 28076.316 | 32892.25 | 47823 | 8.494 | |
| Apoa2 | P09813 | 21804 | 35543.25 | 40072 | 42280.553 | 48248.25 | 61161 | 22.852 | |

**Supplementary Table 2. Discriminating proteins for each knockout using LASSO regression.**

| Knockout gene | Discriminating plasma proteins using LASSO |
| --- | --- |
| *A2m* | F12; Ig heavy chain V region MOPC 47A; Spint1; Serpinf1 |
| *Ahcy* | Hspa5; Serpina1d; Apoa4; B4galt1; Cpn1; Ctsd; F7; C1qa; C1qc; Ig heavy chain V region MOPC 47A; Ig kappa chain V-II region 26; Ig kappa chain V-II region 7S3; Igf1; Kng1; Klkb1; Psma4; Psmb1; Psmb10; Tnc; Isg15; Pros1 |
| *Atp5b* | F5; Cfl1; Cfi; Ig heavy chain V region MOPC 47A; Itih1; Nucb1 |
| *Atp6v0d1* | Pzp; Apoa4; Ces1c; Cd5l; Cgref1; Cfl1; Cycs; Fn1; Ig alpha chain C region; Ighm; Icam2; Kng1; Masp1; Klkb1; Tnc |
| *C8a* | C8a; C8g; Ig alpha chain C region; Masp2 |
| *Cdk4* | Orm2; Cgref1; Hpx; Igh-3; Saa2; Saa1 |
| *Dhfr* | Hspa5; Pzp; Chga; F7; Ecm1; Ig heavy chain V region MOPC 47A; Ig kappa chain V-II region 7S3; Ig kappa chain V-V region MOPC 149; Lum; Nucb1; Serpinf1; Klkb1; Lcp1; Proc; Pon1; Ttr |
| *Dync1li1* | Serpinf2; Apod; B4galt1; Apoh; Blmh; Ggh; Ig alpha chain C region; Ig heavy chain V region MOPC 47A; Ig kappa chain V-II region 17S; Ig kappa chain V-V region K2; Itih1; Spint1; Man2b1; Pcyox1; Pfn1; Lrp1; Psma5; Saa2; Umod; Vcam1 |
| *Galc* | Bpifa2; F5; C1qc; Aldob; Igh-3; Ig gamma-3 chain C region; Ig heavy chain V region MOPC 47A; Ig kappa chain V-II region 26; Spp1; Pla2g7; Psmb1; Ctla2a; Tf; Umod |
| *Gnpda1* | Orm2; Apod; Bpifa2; Cpn2; Cd97; F5; F12; C1qb; C1qc; Cfh; Fetub; Fgl1; Hpx; Igh-3; Ig heavy chain V region MOPC 47A; Igkc; Ig kappa chain V-II region 7S3; Ig kappa chain V-V region K2; Kng1; Spint1; Man2b1; Spp1; Serpina3n; Pros1 |
| *Idh1* | Apob; Chga; Cst3; Cycs; Ctbs; Eef1a1; Ig alpha chain C region; Ig heavy chain V region MOPC 47A; Icam2; Kng1; Lifr; Sell; Msln; Pglyrp2; Psmb10; Rbp4; Tnc; Isg15; Umod; Scgb1a1; Vtn |
| *Iqgap1* | Serpinf2; Apoa4; Apob; Apoc4; B4galt1; Bpifa2; Ctss; Cgref1; F13a1; C1qb; Ctbs; Egfr; Sod3; Hpx; Ighg1; Jchain; Il18bp; Kng1; Spint1; Masp1; Pglyrp2; Ncam1; Gpld1; Pfn1; Psma6; Psma5; Psma2; Psma1; Psmb1; Psmb10; Vtn |
| *Lmbrd1* | Serpinf2; Agt; Apoa4; Bpifa2; Cgref1; F5; C4b; Egfr; Fn1; Ig alpha chain C region; Ig heavy chain V region MOPC 47A; Itih1; Man2b1; Masp2; Olfm1; Serping1; Selenop; Saa2; Tnc |
| *Mfap4* | Serpinc1; Apod; Cgref1; Cycs; Ig alpha chain C region; Ig heavy chain V region MOPC 47A; Krt76; Mfap4; Plxdc2; Selenop; Tf |
| *Mmachc* | Serpinc1; Apod; Apoe; Cgref1; F10; Colec11; Cfi; Ecm1; Fgl1; Gpx3; Ig alpha chain C region; Ig heavy chain V region MOPC 47A; Man2b1; Masp2; Rarres2; Tnc |
| *Mvk* | Agt; Cp; F12; Fgl1; Ighg1; Ig heavy chain V region MOPC 47A; Ig kappa chain V-II region 17S; Ig kappa chain V-V region K2; Itih3; Serpinf1; Isg15 |
| *Nek2* | C1qa; Serpinf1; Tnc |
| *Npc2* | Orm2; Pzp; Eno1; Cp; Eef1a1; Fetub; Fcn1; Ig heavy chain V region MOPC 47A; Spint1; Serpinf1; Ctla2a; Saa1; Tnc; Vcl |
| *Pebp1* | Serpinf2; Apob; Apod; Ctsd; Ecm1; Sod3; Fgl1; Fn1; Gsn; Ig alpha chain C region; Ig kappa chain V-V region K2; Krt76; Kng1; Ctsa; Masp1; Nucb1; Serping1; Psma4; F2; Serpina3c; Pros1 |
| *Phyh* | Serpina1d; Apoa1; Ctsd; Cd97; Efemp1; Fetub; Igh-3; Ig heavy chain V region MOPC 47A; Itih1; Masp2; Mfap4; Klkb1; Serping1; Plg; Pla2g7; Serpina3n; Vcam1 |
| *Pipox* | C1qa; Ig heavy chain V region MOPC 47A; Spint1; Klkb1; Pros1 |
| *Plk1* | Apod; Ctsd; Cd97; Chga; Cst3; Cycs; Hrg; Ighg1; Ig heavy chain V region MOPC 47A; Itih1; Masp2; Olfm1; Serping1; Lrp1; Tnc; Isg15; Vcam1; Vtn |
| *Pmm2* | Pzp; Blmh; Ctsd; C1sa; Cfi; Fstl1; Ig heavy chain V region MOPC 47A; Icam2; Klkb1 |
| *Ptpn12* | Apod; Ctsd; Cp; F7; F12; F13a1; Colec11; Crp; Dag1; Fgl1; Gsn; Ig heavy chain V region MOPC 47A; Ig kappa chain V-III region PC; Masp2; Pfn1; Cfp; Rarres2; Serpina3c; Qsox1 |
| *Pttg1* | Serpinf2; Apoa4; Apob; Ctsd; Cp; Cfl1; C1sa; Aldob; Ig alpha chain C region; Ighg1; Ig heavy chain V region MOPC 47A; Igfals; Itih1; Itih3; Krt76; Kng1; Sell; Psma5; Psma1; Psmb10; Vtn |
| *Rock1* | F13a1; Fetub; Fah; Ig heavy chain V region MOPC 47A; Csf1r |
| *Ulk3* | Orm2; Serpina1d; Pzp; Apoe; Ctsz; C1qc; C1sa; Cfh; Fgl1; Hgfac; Ig gamma-3 chain C region; Ig kappa chain V-IV region S10; Ctsa; Psma5 |
| *Ywhaz* | A2m; Agt; Apob; Chga; Cycs; Lap3; Fgl1; Fn1; Ig alpha chain C region; Ighg1; Ig heavy chain V region MOPC 47A; Icam2; Spint1; Mfap4; Pla2g7; Psma2; Serpina3m; Sparcl1; Tnc |

**Supplementary Table 3. Phenotyping tests performed by IMPC on C8a^-/-^ mice used (C8a^tm1b(EUCOMM)Hmgu^) shown in Supplementary Figure 4.** Details on the individual tests can be found on IMPC website using the following link: www.mousephenotype.org/data/genes/MGI:2668347

| Number | Procedure / Parameter | Life stage | Zygosity | Significant | P Value |
| --- | --- | --- | --- | --- | --- |
| 1 | Combined SHIRPA and Dysmorphology / Forelimb nail - shape | Early adult | HOM | Not significant | 1 |
| 2 | Gross Pathology and Tissue Collection / Lung | Early adult | HOM | Not significant | - |
| 3 | Combined SHIRPA and Dysmorphology / Coat - color pattern - back | Early adult | HOM | Not significant | 1 |
| 4 | Combined SHIRPA and Dysmorphology / Forelimbs - size | Early adult | HOM | Not significant | 1 |
| 5 | X-ray / Ulna | Early adult | HOM | Not significant | 1 |
| 6 | Combined SHIRPA and Dysmorphology / Genitalia - size | Early adult | HOM | Not significant | 1 |
| 7 | Combined SHIRPA and Dysmorphology / Skin color - ear | Early adult | HOM | Not significant | 1 |
| 8 | Hematology / Hematocrit | Early adult | HOM | Not significant | 0.95 |
| 9 | Combined SHIRPA and Dysmorphology / Coat - color - abdomen | Early adult | HOM | Not significant | 1 |
| 10 | Eye Morphology / Lens Opacity | Early adult | HOM | Not significant | 0.84 |
| 11 | Combined SHIRPA and Dysmorphology / Skin color - tail | Early adult | HOM | Not significant | 1 |
| 12 | Hematology / Neutrophil differential count | Early adult | HOM | Not significant | 1.34×10-4 |
| 13 | Clinical Chemistry / Total bilirubin | Early adult | HOM | Not significant | 0.19 |
| 14 | Hematology / Mean platelet volume | Early adult | HOM | Not significant | 0.2 |
| 15 | Gross Pathology and Tissue Collection / Thymus | Early adult | HOM | Not significant | - |
| 16 | Combined SHIRPA and Dysmorphology / Genitalia - presence | Early adult | HOM | Not significant | 1 |
| 17 | Eye Morphology / Narrow eye opening | Early adult | HOM | Not significant | 1 |
| 18 | Electrocardiogram (ECG) / PR | Early adult | HOM | Not significant | 0.96 |
| 19 | Eye Morphology / Corneal vascularization | Early adult | HOM | Not significant | 1 |
| 20 | Combined SHIRPA and Dysmorphology / Contact righting | Early adult | HOM | Not significant | 1 |
| 21 | Gross Pathology and Tissue Collection / Stomach | Early adult | HOM | Not significant | - |
| 22 | Eye Morphology / Pupil Dilation | Early adult | HOM | Not significant | 1 |
| 23 | Hematology / Red blood cell distribution width | Early adult | HOM | Not significant | 0.75 |
| 24 | Gross Pathology and Tissue Collection / Heart | Early adult | HOM | Not significant | - |
| 25 | Combined SHIRPA and Dysmorphology / Skin texture - tail | Early adult | HOM | Not significant | 1 |
| 26 | Combined SHIRPA and Dysmorphology / Trunk curl | Early adult | HOM | Not significant | 1 |
| 27 | X-ray / Fusion of ribs | Early adult | HOM | Not significant | 1 |
| 28 | Eye Morphology / Retina (combined) | Early adult | HOM | Not significant | - |
| 29 | Grip Strength / Forelimb and hindlimb grip strength normalised against body weight | Early adult | HOM | Not significant | 0.52 |
| 30 | Acoustic Startle and Pre-pulse Inhibition (PPI) / % Pre-pulse inhibition - Global | Early adult | HOM | Not significant | 6.68×10-2 |
| 31 | X-ray / Radius | Early adult | HOM | Not significant | 1 |
| 32 | X-ray / Pelvis | Early adult | HOM | Not significant | 0.46 |
| 33 | Clinical Chemistry / Total cholesterol | Early adult | HOM | Not significant | 0.78 |
| 34 | Clinical Chemistry / Total protein | Early adult | HOM | Not significant | 7.40×10-2 |
| 35 | Gross Pathology and Tissue Collection / Lymph node | Early adult | HOM | Not significant | - |
| 36 | Combined SHIRPA and Dysmorphology / Skin color - whole body | Early adult | HOM | Not significant | 1 |
| 37 | Clinical Chemistry / Aspartate aminotransferase | Early adult | HOM | Not significant | 0.82 |
| 38 | Combined SHIRPA and Dysmorphology / Hindlimb digit - shape | Early adult | HOM | Not significant | 1 |
| 39 | Combined SHIRPA and Dysmorphology / Teeth presence | Early adult | HOM | Not significant | 1 |
| 40 | Gross Pathology and Tissue Collection / Epididymis | Early adult | HOM | Not significant | - |
| 41 | Gross Pathology and Tissue Collection / Small intestine | Early adult | HOM | Not significant | - |
| 42 | Combined SHIRPA and Dysmorphology / Activity (body position) | Early adult | HOM | Not significant | 1 |
| 43 | Combined SHIRPA and Dysmorphology / Head morphology | Early adult | HOM | Not significant | 1 |
| 44 | Intraperitoneal glucose tolerance test (IPGTT) / Initial response to glucose challenge | Early adult | HOM | Not significant | 0.12 |
| 45 | Combined SHIRPA and Dysmorphology / Hindlimb nail - number | Early adult | HOM | Not significant | 1 |
| 46 | Electrocardiogram (ECG) / RR | Early adult | HOM | Not significant | 0.18 |
| 47 | Electrocardiogram (ECG) / rMSSD | Early adult | HOM | Not significant | 0.56 |
| 48 | Body Composition (DEXA lean/fat) / Lean mass | Early adult | HOM | Not significant | 0.83 |
| 49 | Clinical Chemistry / Alanine aminotransferase | Early adult | HOM | Not significant | 0.88 |
| 50 | Gross Pathology and Tissue Collection / Esophagus | Early adult | HOM | Not significant | - |
| 51 | Combined SHIRPA and Dysmorphology / Coat - color pattern - abdomen | Early adult | HOM | Not significant | 1 |
| 52 | Combined SHIRPA and Dysmorphology / Hindpaw - size | Early adult | HOM | Not significant | 1 |
| 53 | Hematology / Platelet count | Early adult | HOM | Not significant | 1.97×10-2 |
| 54 | Combined SHIRPA and Dysmorphology / Forelimb digit - shape | Early adult | HOM | Not significant | 1 |
| 55 | Combined SHIRPA and Dysmorphology / Mouth morphology | Early adult | HOM | Not significant | 1 |
| 56 | Body Composition (DEXA lean/fat) / Bone Mineral Density (excluding skull) | Early adult | HOM | Not significant | 4.59×10-3 |
| 57 | X-ray / Skull shape | Early adult | HOM | Not significant | 1 |
| 58 | Gross Pathology and Tissue Collection / Skin | Early adult | HOM | Not significant | - |
| 59 | Combined SHIRPA and Dysmorphology / Hindlimbs - position | Early adult | HOM | Not significant | 1 |
| 60 | Combined SHIRPA and Dysmorphology / Skin color - back paws | Early adult | HOM | Not significant | 1 |
| 61 | Open Field / Number of rears - total | Early adult | HOM | Not significant | 0.37 |
| 62 | Gross Pathology and Tissue Collection / Trachea | Early adult | HOM | Not significant | - |
| 63 | Clinical Chemistry / Albumin | Early adult | HOM | Not significant | 0.65 |
| 64 | Combined SHIRPA and Dysmorphology / Tail - presence | Early adult | HOM | Not significant | 1 |
| 65 | Combined SHIRPA and Dysmorphology / Forelimbs - shape | Early adult | HOM | Not significant | 1 |
| 66 | Combined SHIRPA and Dysmorphology / Skin texture - snout | Early adult | HOM | Not significant | 1 |
| 67 | Auditory Brain Stem Response / 12kHz-evoked ABR Threshold | Early adult | HOM | Not significant | 1 |
| 68 | X-ray / Clavicle | Early adult | HOM | Not significant | 1 |
| 69 | Combined SHIRPA and Dysmorphology / Forepaw - size | Early adult | HOM | Not significant | 1 |
| 70 | Open Field / Whole arena resting time | Early adult | HOM | Not significant | 0.48 |
| 71 | Gross Pathology and Tissue Collection / Uterus | Early adult | HOM | Not significant | - |
| 72 | Intraperitoneal glucose tolerance test (IPGTT) / Area under glucose response curve | Early adult | HOM | Not significant | 0.63 |
| 73 | Combined SHIRPA and Dysmorphology / Startle response | Early adult | HOM | Not significant | 1 |
| 74 | X-ray / Fusion of vertebrae | Early adult | HOM | Not significant | 1 |
| 75 | Acoustic Startle and Pre-pulse Inhibition (PPI) / Response amplitude - S | Early adult | HOM | Not significant | 0.6 |
| 76 | Eye Morphology / Eye | Early adult | HOM | Not significant | 1 |
| 77 | Combined SHIRPA and Dysmorphology / Skin texture - back paws | Early adult | HOM | Not significant | 1 |
| 78 | Gross Pathology and Tissue Collection / Urinary bladder | Early adult | HOM | Not significant | - |
| 79 | Clinical Chemistry / Calcium | Early adult | HOM | Not significant | 5.53×10-2 |
| 80 | Eye Morphology / Eyelid closure | Early adult | HOM | Not significant | 1 |
| 81 | Electrocardiogram (ECG) / ST | Early adult | HOM | Not significant | 7.06×10-3 |
| 82 | Eye Morphology / Iris Pigmentation | Early adult | HOM | Not significant | 1 |
| 83 | Combined SHIRPA and Dysmorphology / Coat - hair texture / appearance - abdomen | Early adult | HOM | Not significant | 1 |
| 84 | X-ray / Syndactylism | Early adult | HOM | Not significant | 1 |
| 85 | Combined SHIRPA and Dysmorphology / Ears | Early adult | HOM | Not significant | 1 |
| 86 | Body Composition (DEXA lean/fat) / Bone Area | Early adult | HOM | Not significant | 6.37×10-2 |
| 87 | Auditory Brain Stem Response / 6kHz-evoked ABR Threshold | Early adult | HOM | Not significant | 1 |
| 88 | Clinical Chemistry / Alkaline phosphatase | Early adult | HOM | Not significant | 0.33 |
| 89 | Eye Morphology / Pupil Position | Early adult | HOM | Not significant | 1 |
| 90 | Gross Pathology and Tissue Collection / Liver | Early adult | HOM | Not significant | - |
| 91 | Combined SHIRPA and Dysmorphology / Upper teeth appearance | Early adult | HOM | Not significant | 1 |
| 92 | Eye Morphology / Corneal opacity | Early adult | HOM | Not significant | 1 |
| 93 | Combined SHIRPA and Dysmorphology / Hindlimb nail - shape | Early adult | HOM | Not significant | 1 |
| 94 | Gross Pathology and Tissue Collection / Gall bladder | Early adult | HOM | Not significant | - |
| 95 | Clinical Chemistry / Urea (Blood Urea Nitrogen - BUN) | Early adult | HOM | Not significant | 0.84 |
| 96 | Eye Morphology / Pupil Light Response | Early adult | HOM | Not significant | 1 |
| 97 | Eye Morphology / Cornea | Early adult | HOM | Not significant | 1 |
| 98 | Grip Strength / Forelimb and hindlimb grip strength measurement mean | Early adult | HOM | Not significant | 0.77 |
| 99 | Combined SHIRPA and Dysmorphology / Hindlimb digit - size | Early adult | HOM | Not significant | 1 |
| 100 | Combined SHIRPA and Dysmorphology / Forelimb nail - length | Early adult | HOM | Not significant | 1 |
| 101 | Acoustic Startle and Pre-pulse Inhibition (PPI) / % Pre-pulse inhibition - PPI4 | Early adult | HOM | Not significant | 4.58×10-2 |
| 102 | Combined SHIRPA and Dysmorphology / Coat - color - head | Early adult | HOM | Not significant | 1 |
| 103 | Acoustic Startle and Pre-pulse Inhibition (PPI) / % Pre-pulse inhibition - PPI3 | Early adult | HOM | Not significant | 8.53×10-2 |
| 104 | Indirect Calorimetry / Respiratory Exchange Ratio | Early adult | HOM | Not significant | 0.59 |
| 105 | Electrocardiogram (ECG) / CV | Early adult | HOM | Not significant | 0.43 |
| 106 | Combined SHIRPA and Dysmorphology / Vibrissae - presence | Early adult | HOM | Not significant | 1 |
| 107 | Eye Morphology / Eye Hemorrhage or Blood Presence | Early adult | HOM | Not significant | 1 |
| 108 | Combined SHIRPA and Dysmorphology / Coat - hair texture / appearance - head | Early adult | HOM | Not significant | 1 |
| 109 | Eye Morphology / Pupil Shape | Early adult | HOM | Not significant | 1 |
| 110 | Eye Morphology / Retinal Blood Vessels Structure | Early adult | HOM | Not significant | 1 |
| 111 | Open Field / Center resting time | Early adult | HOM | Not significant | 1.61×10-2 |
| 112 | Open Field / Whole arena average speed | Early adult | HOM | Not significant | 0.2 |
| 113 | Body Composition (DEXA lean/fat) / Lean/Body weight | Early adult | HOM | Not significant | 0.36 |
| 114 | Body Composition (DEXA lean/fat) / Bone Mineral Content (excluding skull) | Early adult | HOM | Not significant | 3.04×10-2 |
| 115 | Eye Morphology / Fusion between cornea and lens | Early adult | HOM | Not significant | 1 |
| 116 | Open Field / Center permanence time | Early adult | HOM | Not significant | 7.99×10-2 |
| 117 | Combined SHIRPA and Dysmorphology / Locomotor activity | Early adult | HOM | Not significant | 0.57 |
| 118 | Auditory Brain Stem Response / 30kHz-evoked ABR Threshold | Early adult | HOM | Not significant | 1 |
| 119 | Body Composition (DEXA lean/fat) / Fat/Body weight | Early adult | HOM | Not significant | 0.22 |
| 120 | Grip Strength / Forelimb grip strength normalised against body weight | Early adult | HOM | Not significant | 4.91×10-3 |
| 121 | Combined SHIRPA and Dysmorphology / Upper lip morphology | Early adult | HOM | Not significant | 1 |
| 122 | Combined SHIRPA and Dysmorphology / Unexpected behaviors | Early adult | HOM | Not significant | 1 |
| 123 | Gross Pathology and Tissue Collection / Adrenal gland | Early adult | HOM | Not significant | - |
| 124 | Combined SHIRPA and Dysmorphology / Forepaw - shape | Early adult | HOM | Not significant | 1 |
| 125 | Clinical Chemistry / Glucose | Early adult | HOM | Not significant | 0.55 |
| 126 | Combined SHIRPA and Dysmorphology / Aggression | Early adult | HOM | Not significant | 1 |
| 127 | Hematology / Monocyte differential count | Early adult | HOM | Not significant | 1.18×10-2 |
| 128 | Body Composition (DEXA lean/fat) / BMC/Body weight | Early adult | HOM | Not significant | 9.83×10-2 |
| 129 | Eye Morphology / Optic Disc | Early adult | HOM | Not significant | 1 |
| 130 | Combined SHIRPA and Dysmorphology / Forelimb digit - number | Early adult | HOM | Not significant | 1 |
| 131 | Combined SHIRPA and Dysmorphology / Coat - color - back | Early adult | HOM | Not significant | 1 |
| 132 | Combined SHIRPA and Dysmorphology / Lower lip morphology | Early adult | HOM | Not significant | 1 |
| 133 | Open Field / Center average speed | Early adult | HOM | Not significant | 0.37 |
| 134 | Clinical Chemistry / Potassium | Early adult | HOM | Not significant | 0.6 |
| 135 | Gross Pathology and Tissue Collection / Ovary | Early adult | HOM | Not significant | - |
| 136 | Gross Pathology and Tissue Collection / Mammary gland | Early adult | HOM | Not significant | - |
| 137 | X-ray / Scapulae | Early adult | HOM | Not significant | 1 |
| 138 | Hematology / Red blood cell count | Early adult | HOM | Not significant | 0.15 |
| 139 | Gross Pathology and Tissue Collection / Spleen | Early adult | HOM | Not significant | - |
| 140 | Combined SHIRPA and Dysmorphology / Forelimbs - position | Early adult | HOM | Not significant | 1 |
| 141 | Hematology / Mean cell volume | Early adult | HOM | Not significant | 0.47 |
| 142 | Gross Pathology and Tissue Collection / Seminal vesicle | Early adult | HOM | Not significant | - |
| 143 | Gross Pathology and Tissue Collection / Eye with optic nerve | Early adult | HOM | Not significant | - |
| 144 | Combined SHIRPA and Dysmorphology / Skin texture - front paws | Early adult | HOM | Not significant | 1 |
| 145 | Combined SHIRPA and Dysmorphology / Hindlimb digit - number | Early adult | HOM | Not significant | 1 |
| 146 | Electrocardiogram (ECG) / HR | Early adult | HOM | Not significant | 0.17 |
| 147 | Acoustic Startle and Pre-pulse Inhibition (PPI) / % Pre-pulse inhibition - PPI1 | Early adult | HOM | Not significant | 0.92 |
| 148 | Clinical Chemistry / Phosphorus | Early adult | HOM | Not significant | 0.59 |
| 149 | Clinical Chemistry / Creatinine | Early adult | HOM | Not significant | 3.75×10-2 |
| 150 | Combined SHIRPA and Dysmorphology / Vocalization | Early adult | HOM | Not significant | 1 |
| 151 | Body Composition (DEXA lean/fat) / Body length | Early adult | HOM | Not significant | 0.61 |
| 152 | Combined SHIRPA and Dysmorphology / Tremor | Early adult | HOM | Not significant | 1 |
| 153 | Combined SHIRPA and Dysmorphology / Limb grasp | Early adult | HOM | Not significant | 1 |
| 154 | X-ray / Humerus | Early adult | HOM | Not significant | 1 |
| 155 | Combined SHIRPA and Dysmorphology / Lower teeth appearance | Early adult | HOM | Not significant | 1 |
| 156 | Electrocardiogram (ECG) / QRS | Early adult | HOM | Not significant | 0.78 |
| 157 | Open Field / Periphery resting time | Early adult | HOM | Not significant | 0.2 |
| 158 | Auditory Brain Stem Response / Click-evoked ABR threshold | Early adult | HOM | Not significant | 1 |
| 159 | Eye Morphology / Iris/Pupil | Early adult | HOM | Not significant | 1 |
| 160 | Combined SHIRPA and Dysmorphology / Tail - morphology | Early adult | HOM | Not significant | 1 |
| 161 | X-ray / Shape of vertebrae | Early adult | HOM | Not significant | 1 |
| 162 | X-ray / Fibula | Early adult | HOM | Not significant | 1 |
| 163 | Combined SHIRPA and Dysmorphology / Skin texture - ear | Early adult | HOM | Not significant | 1 |
| 164 | Gross Pathology and Tissue Collection / Brain | Early adult | HOM | Not significant | - |
| 165 | Combined SHIRPA and Dysmorphology / Skin color - front paws | Early adult | HOM | Not significant | 1 |
| 166 | Intraperitoneal glucose tolerance test (IPGTT) / Fasted blood glucose concentration | Early adult | HOM | Not significant | 0.16 |
| 167 | Eye Morphology / Persistence of hyaloid vascular system | Early adult | HOM | Not significant | 1 |
| 168 | Clinical Chemistry / HDL-cholesterol | Early adult | HOM | Not significant | 0.44 |
| 169 | Combined SHIRPA and Dysmorphology / Hindlimbs - size | Early adult | HOM | Not significant | 1 |
| 170 | Open Field / Distance travelled - total | Early adult | HOM | Not significant | 0.2 |
| 171 | Combined SHIRPA and Dysmorphology / Snout size | Early adult | HOM | Not significant | 1 |
| 172 | Heart Weight / Heart weight | Early adult | HOM | Not significant | 6.17×10-2 |
| 173 | Combined SHIRPA and Dysmorphology / Forelimb digit - size | Early adult | HOM | Not significant | 1 |
| 174 | Clinical Chemistry / Triglycerides | Early adult | HOM | Not significant | 0.66 |
| 175 | Combined SHIRPA and Dysmorphology / Vibrissae - appearance | Early adult | HOM | Not significant | 1 |
| 176 | Gross Pathology and Tissue Collection / Kidney | Early adult | HOM | Not significant | - |
| 177 | Open Field / Center distance travelled | Early adult | HOM | Not significant | 6.65×10-2 |
| 178 | Fertility of Homozygous Knock-out Mice / Gross Findings Male | Early adult | HOM | Not significant | 1 |
| 179 | X-ray / Femur | Early adult | HOM | Not significant | 1 |
| 180 | Gross Pathology and Tissue Collection / Skeletal muscle | Early adult | HOM | Not significant | - |
| 181 | Gross Pathology and Tissue Collection / Prostate | Early adult | HOM | Not significant | - |
| 182 | Combined SHIRPA and Dysmorphology / Hindlimb nail - length | Early adult | HOM | Not significant | 1 |
| 183 | Eye Morphology / Lens | Early adult | HOM | Not significant | 1 |
| 184 | X-ray / Tibia length | Early adult | HOM | Not significant | 0.17 |
| 185 | Combined SHIRPA and Dysmorphology / Coat - hair texture / appearance - back | Early adult | HOM | Not significant | 1 |
| 186 | Eye Morphology / Synechia | Early adult | HOM | Not significant | 1 |
| 187 | Open Field / Periphery permanence time | Early adult | HOM | Not significant | 7.99×10-2 |
| 188 | Open Field / Periphery average speed | Early adult | HOM | Not significant | 0.92 |
| 189 | Hematology / White blood cell count | Early adult | HOM | Not significant | 8.04×10-4 |
| 190 | Combined SHIRPA and Dysmorphology / Gait | Early adult | HOM | Not significant | 1 |
| 191 | X-ray / Mandibles | Early adult | HOM | Not significant | 1 |
| 192 | Gross Pathology and Tissue Collection / Large intestine | Early adult | HOM | Not significant | - |
| 193 | Electrocardiogram (ECG) / HRV | Early adult | HOM | Not significant | 0.46 |
| 194 | X-ray / Tibia | Early adult | HOM | Not significant | 1 |
| 195 | Open Field / Latency to center entry | Early adult | HOM | Not significant | 5.37×10-3 |
| 196 | X-ray / Digit integrity | Early adult | HOM | Not significant | 1 |
| 197 | Combined SHIRPA and Dysmorphology / Hindlimbs - shape | Early adult | HOM | Not significant | 1 |
| 198 | Clinical Chemistry / Chloride | Early adult | HOM | Not significant | 6.86×10-3 |
| 199 | Acoustic Startle and Pre-pulse Inhibition (PPI) / % Pre-pulse inhibition - PPI2 | Early adult | HOM | Not significant | 6.44×10-3 |
| 200 | Combined SHIRPA and Dysmorphology / Forelimb nail - number | Early adult | HOM | Not significant | 1 |
| 201 | Auditory Brain Stem Response / 18kHz-evoked ABR Threshold | Early adult | HOM | Not significant | 1 |
| 202 | Open Field / Number of center entries | Early adult | HOM | Not significant | 8.78×10-2 |
| 203 | X-ray / Maxilla/Pre-maxilla | Early adult | HOM | Not significant | 1 |
| 204 | Combined SHIRPA and Dysmorphology / Genitalia - morphology | Early adult | HOM | Not significant | 1 |
| 205 | Electrocardiogram (ECG) / QTc | Early adult | HOM | Not significant | 2.87×10-3 |
| 206 | Open Field / Periphery distance travelled | Early adult | HOM | Not significant | 0.38 |
| 207 | X-ray / Zygomatic bone | Early adult | HOM | Not significant | 1 |
| 208 | Eye Morphology / Bulging eye | Early adult | HOM | Not significant | 1 |
| 209 | Grip Strength / Forelimb grip strength measurement mean | Early adult | HOM | Not significant | 6.31×10-3 |
| 210 | Auditory Brain Stem Response / 24kHz-evoked ABR Threshold | Early adult | HOM | Not significant | 1 |
| 211 | Electrocardiogram (ECG) / PQ | Early adult | HOM | Not significant | 0.83 |
| 212 | Hematology / Mean corpuscular hemoglobin | Early adult | HOM | Not significant | 0.47 |
| 213 | Combined SHIRPA and Dysmorphology / Coat - color pattern - head | Early adult | HOM | Not significant | 1 |
| 214 | Combined SHIRPA and Dysmorphology / Coat - hair distribution - abdomen | Early adult | HOM | Not significant | 1 |
| 215 | X-ray / Number of digits | Early adult | HOM | Not significant | 1 |
| 216 | Eye Morphology / Eyelid morphology | Early adult | HOM | Not significant | 1 |
| 217 | Clinical Chemistry / Sodium | Early adult | HOM | Not significant | 0.19 |
| 218 | Combined SHIRPA and Dysmorphology / Tail - length | Early adult | HOM | Not significant | 1 |
| 219 | Hematology / Lymphocyte differential count | Early adult | HOM | Not significant | 3.91×10-3 |
| 220 | Combined SHIRPA and Dysmorphology / Tail - thickness | Early adult | HOM | Not significant | 1 |
| 221 | Eye Morphology / Retinal Blood Vessels | Early adult | HOM | Not significant | 1 |
| 222 | Gross Pathology and Tissue Collection / Thyroid | Early adult | HOM | Not significant | - |
| 223 | X-ray / Processes on vertebrae | Early adult | HOM | Not significant | 1 |
| 224 | X-ray / Shape of ribs | Early adult | HOM | Not significant | 1 |
| 225 | Gross Pathology and Tissue Collection / Pancreas | Early adult | HOM | Not significant | - |
| 226 | Hematology / Eosinophil differential count | Early adult | HOM | Not significant | 0.12 |
| 227 | Combined SHIRPA and Dysmorphology / Skin texture - whole body | Early adult | HOM | Not significant | 1 |
| 228 | X-ray / Teeth | Early adult | HOM | Not significant | 1 |
| 229 | X-ray / Joints | Early adult | HOM | Not significant | 1 |
| 230 | Combined SHIRPA and Dysmorphology / Head size | Early adult | HOM | Not significant | 1 |
| 231 | Eye Morphology / Retinal Blood Vessels Pattern | Early adult | HOM | Not significant | 1 |
| 232 | Hematology / Mean cell hemoglobin concentration | Early adult | HOM | Not significant | 2.59×10-2 |
| 233 | Fertility of Homozygous Knock-out Mice / Gross Findings Female | Early adult | HOM | Not significant | 1 |
| 234 | Combined SHIRPA and Dysmorphology / Skin color - snout | Early adult | HOM | Not significant | 1 |
| 235 | Combined SHIRPA and Dysmorphology / Coat - hair distribution - head | Early adult | HOM | Not significant | 1 |
| 236 | Open Field / Percentage center time | Early adult | HOM | Not significant | 7.99×10-2 |
| 237 | Gross Pathology and Tissue Collection / Spinal cord | Early adult | HOM | Not significant | - |
| 238 | Body Composition (DEXA lean/fat) / Fat mass | Early adult | HOM | Not significant | 0.23 |
| 239 | Gross Pathology and Tissue Collection / Testes | Early adult | HOM | Not significant | - |
| 240 | Viability Primary Screen / Viability Outcome | Early adult | HOM | Not significant | - |
| 241 | X-ray / Brachydactyly | Early adult | HOM | Not significant | 1 |
| 242 | Electrocardiogram (ECG) / QTc Dispersion | Early adult | HOM | Not significant | 0.22 |
| 243 | Combined SHIRPA and Dysmorphology / Hindpaw - shape | Early adult | HOM | Not significant | 1 |
| 244 | Combined SHIRPA and Dysmorphology / Head bobbing | Early adult | HOM | Not significant | 1 |
| 245 | Combined SHIRPA and Dysmorphology / Coat - hair distribution - back | Early adult | HOM | Not significant | 1 |
| 246 | Hematology / Hemoglobin | Early adult | HOM | Not significant | 1.03×10-2 |

**Supplementary Table 4. Phenotyping tests performed by IMPC on *Npc2^+/-^* mice (Npc2^tm1e.1(EUCOMM)Wtsi^) shown in Supplementary Figure 5.** Details on the individual tests can be found on IMPC website using the following link: www.mousephenotype.org/data/genes/MGI:1915213

| Number | Procedure / Parameter | Life stage | Zygosity | Significant | P Value |
| --- | --- | --- | --- | --- | --- |
| 1 | Viability Primary Screen / Number of anzygous females | Early adult | ANZ | Not significant | - |
| 2 | Viability Primary Screen / Total anzygous females | Early adult | ANZ | Not significant | - |
| 3 | Viability Primary Screen / Total of hemizygous males | Early adult | HEM | Not significant | - |
| 4 | Viability Primary Screen / Number of hemizygous males | Early adult | HEM | Not significant | - |
| 5 | Acoustic Startle and Pre-pulse Inhibition (PPI) / Response amplitude - PP1 | Early adult | HET | Not significant | - |
| 6 | Acoustic Startle and Pre-pulse Inhibition (PPI) / % Pre-pulse inhibition - PPI2 | Early adult | HET | Not significant | 6.82×10-3 |
| 7 | Acoustic Startle and Pre-pulse Inhibition (PPI) / Response amplitude - PP4_S | Early adult | HET | Not significant | - |
| 8 | Acoustic Startle and Pre-pulse Inhibition (PPI) / Response amplitude - PP3 | Early adult | HET | Not significant | - |
| 9 | Acoustic Startle and Pre-pulse Inhibition (PPI) / Response amplitude - PP2 | Early adult | HET | Not significant | - |
| 10 | Acoustic Startle and Pre-pulse Inhibition (PPI) / Response amplitude - PP2_S | Early adult | HET | Not significant | - |
| 11 | Acoustic Startle and Pre-pulse Inhibition (PPI) / Response amplitude - PP4 | Early adult | HET | Not significant | - |
| 12 | Acoustic Startle and Pre-pulse Inhibition (PPI) / Response amplitude - PP3_S | Early adult | HET | Not significant | - |
| 13 | Acoustic Startle and Pre-pulse Inhibition (PPI) / % Pre-pulse inhibition - PPI3 | Early adult | HET | Not significant | 1.05×10-3 |
| 14 | Acoustic Startle and Pre-pulse Inhibition (PPI) / % Pre-pulse inhibition - PPI4 | Early adult | HET | Not significant | 7.96×10-2 |
| 15 | Acoustic Startle and Pre-pulse Inhibition (PPI) / Response amplitude - S | Early adult | HET | Not significant | 0.26 |
| 16 | Acoustic Startle and Pre-pulse Inhibition (PPI) / Response amplitude - BN | Early adult | HET | Not significant | - |
| 17 | Acoustic Startle and Pre-pulse Inhibition (PPI) / % Pre-pulse inhibition - PPI1 | Early adult | HET | Not significant | 2.55×10-4 |
| 18 | Acoustic Startle and Pre-pulse Inhibition (PPI) / Response amplitude - PP1_S | Early adult | HET | Not significant | - |
| 19 | Acoustic Startle and Pre-pulse Inhibition (PPI) / % Pre-pulse inhibition - Global | Early adult | HET | Not significant | 3.14×10-4 |
| 20 | Auditory Brain Stem Response / 24kHz-evoked ABR Threshold | Early adult | HET | Not significant | 1.06×10-3 |
| 21 | Auditory Brain Stem Response / 12kHz-evoked ABR Threshold | Early adult | HET | Not significant | 0.22 |
| 22 | Auditory Brain Stem Response / 30kHz-evoked ABR Threshold | Early adult | HET | Not significant | 0.19 |
| 23 | Auditory Brain Stem Response / 18kHz-evoked ABR Threshold | Early adult | HET | Not significant | 0.29 |
| 24 | Auditory Brain Stem Response / Body weight | Early adult | HET | Not significant | - |
| 25 | Auditory Brain Stem Response / 6kHz-evoked ABR Threshold | Early adult | HET | Not significant | 4.21×10-3 |
| 26 | Auditory Brain Stem Response / Click-evoked ABR threshold | Early adult | HET | Not significant | 0.12 |
| 27 | Body Composition (DEXA lean/fat) / Fat/Body weight | Early adult | HET | Not significant | 3.56×10-2 |
| 28 | Body Composition (DEXA lean/fat) / Body length | Early adult | HET | Not significant | 0.65 |
| 29 | Body Composition (DEXA lean/fat) / Fat mass | Early adult | HET | Not significant | 0.36 |
| 30 | Body Composition (DEXA lean/fat) / Bone Area | Early adult | HET | Not significant | 0.44 |
| 31 | Body Composition (DEXA lean/fat) / Body weight | Early adult | HET | Not significant | - |
| 32 | Body Composition (DEXA lean/fat) / Bone Mineral Density (excluding skull) | Early adult | HET | Not significant | 0.63 |
| 33 | Body Composition (DEXA lean/fat) / Lean mass | Early adult | HET | Not significant | 0.55 |
| 34 | Body Composition (DEXA lean/fat) / Bone Mineral Content (excluding skull) | Early adult | HET | Not significant | 0.37 |
| 35 | Body Composition (DEXA lean/fat) / BMC/Body weight | Early adult | HET | Not significant | 0.66 |
| 36 | Body Composition (DEXA lean/fat) / Lean/Body weight | Early adult | HET | Not significant | 0.54 |
| 37 | Body Weight / Body weight curve | Early adult | HET | Not significant | - |
| 38 | Body Weight / Body weight | Early adult | HET | Not significant | - |
| 39 | Clinical Chemistry / Potassium | Early adult | HET | Significant | 3.90×10-6 |
| 40 | Clinical Chemistry / Urea (Blood Urea Nitrogen - BUN) | Early adult | HET | Not significant | 5.51×10-3 |
| 41 | Clinical Chemistry / Total cholesterol | Early adult | HET | Not significant | 0.82 |
| 42 | Clinical Chemistry / Total protein | Early adult | HET | Not significant | 8.33×10-2 |
| 43 | Clinical Chemistry / Creatinine | Early adult | HET | Not significant | 0.5 |
| 44 | Clinical Chemistry / Total bilirubin | Early adult | HET | Not significant | 0.18 |
| 45 | Clinical Chemistry / Alkaline phosphatase | Early adult | HET | Not significant | 1.54×10-2 |
| 46 | Clinical Chemistry / Aspartate aminotransferase | Early adult | HET | Not significant | 0.3 |
| 47 | Clinical Chemistry / Glucose | Early adult | HET | Not significant | 6.57×10-2 |
| 48 | Clinical Chemistry / Cholesterol ratio | Early adult | HET | Not significant | 7.28×10-2 |
| 49 | Clinical Chemistry / Phosphorus | Early adult | HET | Not significant | 4.72×10-2 |
| 50 | Clinical Chemistry / Calcium | Early adult | HET | Not significant | 0.5 |
| 51 | Clinical Chemistry / HDL-cholesterol | Early adult | HET | Not significant | 0.85 |
| 52 | Clinical Chemistry / Chloride | Early adult | HET | Not significant | 0.1 |
| 53 | Clinical Chemistry / LIH (Hemolysis Severity - available on AU analysers) | Early adult | HET | Not significant | - |
| 54 | Clinical Chemistry / Alanine aminotransferase | Early adult | HET | Not significant | 0.48 |
| 55 | Clinical Chemistry / Albumin | Early adult | HET | Not significant | 2.33×10-2 |
| 56 | Clinical Chemistry / Sodium | Early adult | HET | Not significant | 2.46×10-2 |
| 57 | Clinical Chemistry / Triglycerides | Early adult | HET | Not significant | 0.83 |
| 58 | Combined SHIRPA and Dysmorphology / Snout size | Early adult | HET | Not significant | - |
| 59 | Combined SHIRPA and Dysmorphology / Skin texture - ear | Early adult | HET | Not significant | - |
| 60 | Combined SHIRPA and Dysmorphology / Forelimbs - size | Early adult | HET | Not significant | - |
| 61 | Combined SHIRPA and Dysmorphology / Head morphology | Early adult | HET | Not significant | 1 |
| 62 | Combined SHIRPA and Dysmorphology / Tremor | Early adult | HET | Not significant | - |
| 63 | Combined SHIRPA and Dysmorphology / Skin texture - tail | Early adult | HET | Not significant | - |
| 64 | Combined SHIRPA and Dysmorphology / Teeth presence | Early adult | HET | Not significant | - |
| 65 | Combined SHIRPA and Dysmorphology / Hindpaw - size | Early adult | HET | Not significant | - |
| 66 | Combined SHIRPA and Dysmorphology / Hindlimb digit - number | Early adult | HET | Not significant | - |
| 67 | Combined SHIRPA and Dysmorphology / Vocalization | Early adult | HET | Not significant | 1 |
| 68 | Combined SHIRPA and Dysmorphology / Vibrissae - presence | Early adult | HET | Not significant | - |
| 69 | Combined SHIRPA and Dysmorphology / Coat - color pattern - back | Early adult | HET | Not significant | - |
| 70 | Combined SHIRPA and Dysmorphology / Tail - morphology | Early adult | HET | Not significant | - |
| 71 | Combined SHIRPA and Dysmorphology / Hindlimbs - shape | Early adult | HET | Not significant | - |
| 72 | Combined SHIRPA and Dysmorphology / Skin texture - front paws | Early adult | HET | Not significant | - |
| 73 | Combined SHIRPA and Dysmorphology / Forelimb nail - length | Early adult | HET | Not significant | - |
| 74 | Combined SHIRPA and Dysmorphology / Forelimb nail - shape | Early adult | HET | Not significant | - |
| 75 | Combined SHIRPA and Dysmorphology / Lower teeth appearance | Early adult | HET | Not significant | 1 |
| 76 | Combined SHIRPA and Dysmorphology / Startle response | Early adult | HET | Not significant | - |
| 77 | Combined SHIRPA and Dysmorphology / Coat - color - back | Early adult | HET | Not significant | - |
| 78 | Combined SHIRPA and Dysmorphology / Coat - color - abdomen | Early adult | HET | Not significant | 1 |
| 79 | Combined SHIRPA and Dysmorphology / Activity (body position) | Early adult | HET | Not significant | - |
| 80 | Combined SHIRPA and Dysmorphology / Coat - hair distribution - head | Early adult | HET | Not significant | - |
| 81 | Combined SHIRPA and Dysmorphology / Head size | Early adult | HET | Not significant | - |
| 82 | Combined SHIRPA and Dysmorphology / Head bobbing | Early adult | HET | Not significant | - |
| 83 | Combined SHIRPA and Dysmorphology / Genitalia - size | Early adult | HET | Not significant | - |
| 84 | Combined SHIRPA and Dysmorphology / Upper teeth appearance | Early adult | HET | Not significant | 1 |
| 85 | Combined SHIRPA and Dysmorphology / Coat - hair texture / appearance - head | Early adult | HET | Not significant | - |
| 86 | Combined SHIRPA and Dysmorphology / Coat - hair distribution - abdomen | Early adult | HET | Not significant | 1 |
| 87 | Combined SHIRPA and Dysmorphology / Lower lip morphology | Early adult | HET | Not significant | - |
| 88 | Combined SHIRPA and Dysmorphology / Locomotor activity | Early adult | HET | Not significant | 3.51×10-2 |
| 89 | Combined SHIRPA and Dysmorphology / Skin color - ear | Early adult | HET | Not significant | - |
| 90 | Combined SHIRPA and Dysmorphology / Tail - length | Early adult | HET | Not significant | - |
| 91 | Combined SHIRPA and Dysmorphology / Skin color - whole body | Early adult | HET | Not significant | 1 |
| 92 | Combined SHIRPA and Dysmorphology / Skin texture - back paws | Early adult | HET | Not significant | - |
| 93 | Combined SHIRPA and Dysmorphology / Forepaw - size | Early adult | HET | Not significant | - |
| 94 | Combined SHIRPA and Dysmorphology / Hindlimb nail - shape | Early adult | HET | Not significant | - |
| 95 | Combined SHIRPA and Dysmorphology / Hindpaw - shape | Early adult | HET | Not significant | - |
| 96 | Combined SHIRPA and Dysmorphology / Hindlimbs - position | Early adult | HET | Not significant | - |
| 97 | Combined SHIRPA and Dysmorphology / Skin texture - snout | Early adult | HET | Not significant | - |
| 98 | Combined SHIRPA and Dysmorphology / Contact righting | Early adult | HET | Not significant | - |
| 99 | Combined SHIRPA and Dysmorphology / Hindlimb digit - shape | Early adult | HET | Not significant | - |
| 100 | Combined SHIRPA and Dysmorphology / Coat - color pattern - abdomen | Early adult | HET | Not significant | - |
| 101 | Combined SHIRPA and Dysmorphology / Skin color - snout | Early adult | HET | Not significant | - |
| 102 | Combined SHIRPA and Dysmorphology / Upper lip morphology | Early adult | HET | Not significant | - |
| 103 | Combined SHIRPA and Dysmorphology / Hindlimb nail - number | Early adult | HET | Not significant | - |
| 104 | Combined SHIRPA and Dysmorphology / Coat - hair texture / appearance - back | Early adult | HET | Not significant | - |
| 105 | Combined SHIRPA and Dysmorphology / Gait | Early adult | HET | Not significant | - |
| 106 | Combined SHIRPA and Dysmorphology / Trunk curl | Early adult | HET | Not significant | - |
| 107 | Combined SHIRPA and Dysmorphology / Forelimb digit - number | Early adult | HET | Not significant | - |
| 108 | Combined SHIRPA and Dysmorphology / Hindlimbs - size | Early adult | HET | Not significant | - |
| 109 | Combined SHIRPA and Dysmorphology / Ears | Early adult | HET | Not significant | - |
| 110 | Combined SHIRPA and Dysmorphology / Forelimb digit - size | Early adult | HET | Not significant | 1 |
| 111 | Combined SHIRPA and Dysmorphology / Coat - hair texture / appearance - abdomen | Early adult | HET | Not significant | - |
| 112 | Combined SHIRPA and Dysmorphology / Hindlimb nail - length | Early adult | HET | Not significant | - |
| 113 | Combined SHIRPA and Dysmorphology / Skin texture - whole body | Early adult | HET | Not significant | 0.38 |
| 114 | Combined SHIRPA and Dysmorphology / Skin color - tail | Early adult | HET | Not significant | - |
| 115 | Combined SHIRPA and Dysmorphology / Tail elevation | Early adult | HET | Not significant | - |
| 116 | Combined SHIRPA and Dysmorphology / Genitalia - morphology | Early adult | HET | Not significant | - |
| 117 | Combined SHIRPA and Dysmorphology / Forelimb nail - number | Early adult | HET | Not significant | - |
| 118 | Combined SHIRPA and Dysmorphology / Skin color - front paws | Early adult | HET | Not significant | - |
| 119 | Combined SHIRPA and Dysmorphology / Tail - presence | Early adult | HET | Not significant | - |
| 120 | Combined SHIRPA and Dysmorphology / Tail - thickness | Early adult | HET | Not significant | - |
| 121 | Combined SHIRPA and Dysmorphology / Forelimb digit - shape | Early adult | HET | Not significant | - |
| 122 | Combined SHIRPA and Dysmorphology / Skin color - back paws | Early adult | HET | Not significant | - |
| 123 | Combined SHIRPA and Dysmorphology / Vibrissae - appearance | Early adult | HET | Not significant | - |
| 124 | Combined SHIRPA and Dysmorphology / Unexpected behaviors | Early adult | HET | Not significant | - |
| 125 | Combined SHIRPA and Dysmorphology / Forelimbs - shape | Early adult | HET | Not significant | - |
| 126 | Combined SHIRPA and Dysmorphology / Genitalia - presence | Early adult | HET | Not significant | - |
| 127 | Combined SHIRPA and Dysmorphology / Transfer arousal | Early adult | HET | Not significant | - |
| 128 | Combined SHIRPA and Dysmorphology / Coat - color - head | Early adult | HET | Not significant | - |
| 129 | Combined SHIRPA and Dysmorphology / Mouth morphology | Early adult | HET | Not significant | - |
| 130 | Combined SHIRPA and Dysmorphology / Coat - hair distribution - back | Early adult | HET | Not significant | - |
| 131 | Combined SHIRPA and Dysmorphology / Limb grasp | Early adult | HET | Not significant | - |
| 132 | Combined SHIRPA and Dysmorphology / Coat - color pattern - head | Early adult | HET | Not significant | - |
| 133 | Combined SHIRPA and Dysmorphology / Hindlimb digit - size | Early adult | HET | Not significant | - |
| 134 | Combined SHIRPA and Dysmorphology / Forelimbs - position | Early adult | HET | Not significant | - |
| 135 | Combined SHIRPA and Dysmorphology / Touch escape | Early adult | HET | Not significant | - |
| 136 | Combined SHIRPA and Dysmorphology / Aggression | Early adult | HET | Not significant | - |
| 137 | Combined SHIRPA and Dysmorphology / Forepaw - shape | Early adult | HET | Not significant | - |
| 138 | Electrocardiogram (ECG) / CV | Early adult | HET | Not significant | 4.57×10-2 |
| 139 | Electrocardiogram (ECG) / PQ | Early adult | HET | Not significant | 0.78 |
| 140 | Electrocardiogram (ECG) / HR | Early adult | HET | Not significant | 0.96 |
| 141 | Electrocardiogram (ECG) / QTc | Early adult | HET | Not significant | 0.44 |
| 142 | Electrocardiogram (ECG) / pNN5(6>ms) | Early adult | HET | Not significant | - |
| 143 | Electrocardiogram (ECG) / RR | Early adult | HET | Not significant | 0.82 |
| 144 | Electrocardiogram (ECG) / ST | Early adult | HET | Not significant | 0.23 |
| 145 | Electrocardiogram (ECG) / Mean SR amplitude | Early adult | HET | Not significant | - |
| 146 | Electrocardiogram (ECG) / Mean R amplitude | Early adult | HET | Not significant | - |
| 147 | Electrocardiogram (ECG) / QTc Dispersion | Early adult | HET | Not significant | 0.56 |
| 148 | Electrocardiogram (ECG) / rMSSD | Early adult | HET | Not significant | 8.74×10-2 |
| 149 | Electrocardiogram (ECG) / HRV | Early adult | HET | Not significant | 4.59×10-2 |
| 150 | Electrocardiogram (ECG) / PR | Early adult | HET | Not significant | 0.91 |
| 151 | Electrocardiogram (ECG) / Number of signals | Early adult | HET | Not significant | - |
| 152 | Electrocardiogram (ECG) / QRS | Early adult | HET | Not significant | 0.49 |
| 153 | Eye Morphology / Pupil Dilation | Early adult | HET | Not significant | - |
| 154 | Eye Morphology / Retinal Pigmentation | Early adult | HET | Not significant | - |
| 155 | Eye Morphology / Eye Hemorrhage or Blood Presence | Early adult | HET | Not significant | - |
| 156 | Eye Morphology / Optic Disc | Early adult | HET | Not significant | 1 |
| 157 | Eye Morphology / Iris/Pupil | Early adult | HET | Not significant | 1 |
| 158 | Eye Morphology / Persistence of hyaloid vascular system | Early adult | HET | Not significant | - |
| 159 | Eye Morphology / Retinal Blood Vessels Pattern | Early adult | HET | Not significant | - |
| 160 | Eye Morphology / Retinal Structure | Early adult | HET | Not significant | - |
| 161 | Eye Morphology / Retina | Early adult | HET | Not significant | - |
| 162 | Eye Morphology / Iris Pigmentation | Early adult | HET | Not significant | 1 |
| 163 | Eye Morphology / Synechia | Early adult | HET | Not significant | - |
| 164 | Eye Morphology / Cornea | Early adult | HET | Not significant | - |
| 165 | Eye Morphology / Retinal Blood Vessels Structure | Early adult | HET | Not significant | - |
| 166 | Eye Morphology / Eye | Early adult | HET | Not significant | - |
| 167 | Eye Morphology / Retinal Blood Vessels | Early adult | HET | Not significant | 1 |
| 168 | Eye Morphology / Pupil Position | Early adult | HET | Not significant | 1 |
| 169 | Eye Morphology / Eyelid closure | Early adult | HET | Not significant | - |
| 170 | Eye Morphology / Retina (combined) | Early adult | HET | Not significant | 1 |
| 171 | Eye Morphology / Eyelid morphology | Early adult | HET | Not significant | - |
| 172 | Eye Morphology / Narrow eye opening | Early adult | HET | Not significant | - |
| 173 | Eye Morphology / Bulging eye | Early adult | HET | Not significant | - |
| 174 | Eye Morphology / Corneal opacity | Early adult | HET | Not significant | 1 |
| 175 | Eye Morphology / Corneal vascularization | Early adult | HET | Not significant | - |
| 176 | Eye Morphology / Pupil Light Response | Early adult | HET | Not significant | - |
| 177 | Eye Morphology / Lens Opacity | Early adult | HET | Not significant | - |
| 178 | Eye Morphology / Fusion between cornea and lens | Early adult | HET | Not significant | - |
| 179 | Eye Morphology / Pupil Shape | Early adult | HET | Not significant | 1 |
| 180 | Eye Morphology / Lens | Early adult | HET | Not significant | - |
| 181 | Grip Strength / Forelimb grip strength measurement mean | Early adult | HET | Not significant | 3.04×10-2 |
| 182 | Grip Strength / Forelimb and hindlimb grip strength measurement mean | Early adult | HET | Not significant | 0.35 |
| 183 | Grip Strength / Forelimb grip strength measurement | Early adult | HET | Not significant | 1.15×10-3 |
| 184 | Grip Strength / Forelimb grip strength normalised against body weight | Early adult | HET | Not significant | 1.83×10-2 |
| 185 | Grip Strength / Body weight | Early adult | HET | Not significant | - |
| 186 | Grip Strength / Forelimb and hindlimb grip strength measurement | Early adult | HET | Not significant | 0.91 |
| 187 | Grip Strength / Forelimb and hindlimb grip strength normalised against body weight | Early adult | HET | Not significant | 0.2 |
| 188 | Gross Pathology and Tissue Collection / Kidney | Early adult | HET | Not significant | 1 |
| 189 | Gross Pathology and Tissue Collection / Lung | Early adult | HET | Not significant | 1 |
| 190 | Gross Pathology and Tissue Collection / Ovary | Early adult | HET | Not significant | 1 |
| 191 | Gross Pathology and Tissue Collection / Trachea | Early adult | HET | Not significant | 1 |
| 192 | Gross Pathology and Tissue Collection / Body Weight | Early adult | HET | Not significant | - |
| 193 | Gross Pathology and Tissue Collection / Thyroid | Early adult | HET | Not significant | 1 |
| 194 | Gross Pathology and Tissue Collection / Testes | Early adult | HET | Not significant | 1 |
| 195 | Gross Pathology and Tissue Collection / Large intestine | Early adult | HET | Not significant | 1 |
| 196 | Gross Pathology and Tissue Collection / Skin | Early adult | HET | Not significant | 1 |
| 197 | Gross Pathology and Tissue Collection / Urinary bladder | Early adult | HET | Not significant | 1 |
| 198 | Gross Pathology and Tissue Collection / Lymph node | Early adult | HET | Not significant | 1 |
| 199 | Gross Pathology and Tissue Collection / Liver | Early adult | HET | Not significant | 1 |
| 200 | Gross Pathology and Tissue Collection / Seminal vesicle | Early adult | HET | Not significant | 1 |
| 201 | Gross Pathology and Tissue Collection / Pancreas | Early adult | HET | Not significant | 1 |
| 202 | Gross Pathology and Tissue Collection / Epididymis | Early adult | HET | Not significant | 1 |
| 203 | Gross Pathology and Tissue Collection / Small intestine | Early adult | HET | Not significant | 1 |
| 204 | Gross Pathology and Tissue Collection / Esophagus | Early adult | HET | Not significant | 1 |
| 205 | Gross Pathology and Tissue Collection / Uterus | Early adult | HET | Not significant | 1 |
| 206 | Gross Pathology and Tissue Collection / Eye with optic nerve | Early adult | HET | Not significant | 1 |
| 207 | Gross Pathology and Tissue Collection / Thymus | Early adult | HET | Not significant | 1 |
| 208 | Gross Pathology and Tissue Collection / Heart | Early adult | HET | Not significant | 1 |
| 209 | Gross Pathology and Tissue Collection / Skeletal muscle | Early adult | HET | Not significant | 1 |
| 210 | Gross Pathology and Tissue Collection / Brain | Early adult | HET | Not significant | 1 |
| 211 | Gross Pathology and Tissue Collection / Mammary gland | Early adult | HET | Not significant | 1 |
| 212 | Gross Pathology and Tissue Collection / Gall bladder | Early adult | HET | Not significant | 1 |
| 213 | Gross Pathology and Tissue Collection / Prostate | Early adult | HET | Not significant | 1 |
| 214 | Gross Pathology and Tissue Collection / Stomach | Early adult | HET | Not significant | 1 |
| 215 | Gross Pathology and Tissue Collection / Comments (in English) | Early adult | HET | Not significant | - |
| 216 | Gross Pathology and Tissue Collection / Spleen | Early adult | HET | Not significant | 1 |
| 217 | Gross Pathology and Tissue Collection / Spinal cord | Early adult | HET | Not significant | 1 |
| 218 | Gross Pathology and Tissue Collection / Images | Early adult | HET | Not significant | - |
| 219 | Gross Pathology and Tissue Collection / Adrenal gland | Early adult | HET | Significant | 0 |
| 220 | Heart Weight / Body weight | Early adult | HET | Not significant | - |
| 221 | Heart Weight / Heart weight normalised against body weight | Early adult | HET | Not significant | - |
| 222 | Heart Weight / Heart weight | Early adult | HET | Not significant | 0.96 |
| 223 | Hematology / Eosinophil cell count | Early adult | HET | Not significant | 0.92 |
| 224 | Hematology / Sample clotted | Early adult | HET | Not significant | - |
| 225 | Hematology / Basophil differential count | Early adult | HET | Not significant | 0.71 |
| 226 | Hematology / Neutrophil differential count | Early adult | HET | Not significant | 0.41 |
| 227 | Hematology / Mean cell hemoglobin concentration | Early adult | HET | Not significant | 0.22 |
| 228 | Hematology / Hemoglobin | Early adult | HET | Not significant | 3.41×10-2 |
| 229 | Hematology / Mean cell volume | Early adult | HET | Not significant | 0.55 |
| 230 | Hematology / Neutrophil cell count | Early adult | HET | Not significant | 0.93 |
| 231 | Hematology / Hematocrit | Early adult | HET | Not significant | 0.36 |
| 232 | Hematology / Eosinophil differential count | Early adult | HET | Not significant | 0.83 |
| 233 | Hematology / Red blood cell count | Early adult | HET | Not significant | 0.74 |
| 234 | Hematology / White blood cell count | Early adult | HET | Not significant | 0.4 |
| 235 | Hematology / Lymphocyte cell count | Early adult | HET | Not significant | 0.47 |
| 236 | Hematology / Basophil cell count | Early adult | HET | Not significant | 0.86 |
| 237 | Hematology / Lymphocyte differential count | Early adult | HET | Not significant | 0.51 |
| 238 | Hematology / Monocyte differential count | Early adult | HET | Not significant | 0.17 |
| 239 | Hematology / Monocyte cell count | Early adult | HET | Not significant | 5.95×10-2 |
| 240 | Hematology / Mean platelet volume | Early adult | HET | Not significant | 0.41 |
| 241 | Hematology / Platelet count | Early adult | HET | Not significant | 0.76 |
| 242 | Hematology / Mean corpuscular hemoglobin | Early adult | HET | Not significant | 1.02×10-2 |
| 243 | Hematology / Red blood cell distribution width | Early adult | HET | Not significant | 0.99 |
| 244 | Indirect Calorimetry / Respiratory Exchange Ratio | Early adult | HET | Not significant | 0.31 |
| 245 | Indirect Calorimetry / Total food intake | Early adult | HET | Not significant | 0.74 |
| 246 | Indirect Calorimetry / Body weight after experiment | Early adult | HET | Not significant | - |
| 247 | Indirect Calorimetry / Carbon dioxide production | Early adult | HET | Not significant | 8.09×10-2 |
| 248 | Indirect Calorimetry / Total activity (no. of fine movement + no. of beam cuts) | Early adult | HET | Not significant | - |
| 249 | Indirect Calorimetry / Heat production (metabolic rate) | Early adult | HET | Not significant | 2.70×10-2 |
| 250 | Indirect Calorimetry / Oxygen consumption | Early adult | HET | Not significant | 2.09×10-2 |
| 251 | Indirect Calorimetry / Respiratory Exchange Ratio series | Early adult | HET | Not significant | - |
| 252 | Indirect Calorimetry / Ambulatory activity (no. of beam cuts) | Early adult | HET | Not significant | - |
| 253 | Indirect Calorimetry / Cumulative food intake | Early adult | HET | Not significant | 0.83 |
| 254 | Indirect Calorimetry / Body weight before experiment | Early adult | HET | Not significant | - |
| 255 | Intraperitoneal glucose tolerance test (IPGTT) / Blood glucose concentration | Early adult | HET | Not significant | 0.85 |
| 256 | Intraperitoneal glucose tolerance test (IPGTT) / Body Weight | Early adult | HET | Not significant | - |
| 257 | Intraperitoneal glucose tolerance test (IPGTT) / Fasted blood glucose concentration | Early adult | HET | Not significant | 0.57 |
| 258 | Intraperitoneal glucose tolerance test (IPGTT) / Initial response to glucose challenge | Early adult | HET | Not significant | 0.36 |
| 259 | Intraperitoneal glucose tolerance test (IPGTT) / Area under glucose response curve | Early adult | HET | Not significant | 0.74 |
| 260 | Open Field / Periphery resting time series | Early adult | HET | Not significant | 0.94 |
| 261 | Open Field / Periphery resting time | Early adult | HET | Not significant | 0.63 |
| 262 | Open Field / Whole arena resting time | Early adult | HET | Not significant | 0.98 |
| 263 | Open Field / Whole arena permanence | Early adult | HET | Not significant | - |
| 264 | Open Field / Center permanence time | Early adult | HET | Not significant | 0.61 |
| 265 | Open Field / Periphery permanence time | Early adult | HET | Not significant | 0.61 |
| 266 | Open Field / Distance travelled | Early adult | HET | Not significant | 0.88 |
| 267 | Open Field / Percentage center time | Early adult | HET | Not significant | 0.61 |
| 268 | Open Field / Center distance travelled series | Early adult | HET | Not significant | 0.88 |
| 269 | Open Field / Distance travelled - total | Early adult | HET | Not significant | 0.72 |
| 270 | Open Field / Periphery average speed series | Early adult | HET | Not significant | 0.3 |
| 271 | Open Field / Number of center entries | Early adult | HET | Not significant | 0.4 |
| 272 | Open Field / Center average speed series | Early adult | HET | Not significant | 9.10×10-3 |
| 273 | Open Field / Latency to center entry | Early adult | HET | Not significant | 0.54 |
| 274 | Open Field / Whole arena average speed series | Early adult | HET | Not significant | 0.32 |
| 275 | Open Field / Periphery permanence time series | Early adult | HET | Not significant | 0.7 |
| 276 | Open Field / Center distance travelled | Early adult | HET | Not significant | 0.64 |
| 277 | Open Field / Periphery distance travelled | Early adult | HET | Not significant | 0.77 |
| 278 | Open Field / Center resting time | Early adult | HET | Not significant | 0.44 |
| 279 | Open Field / Periphery average speed | Early adult | HET | Not significant | 0.22 |
| 280 | Open Field / Center permanence time series | Early adult | HET | Not significant | 0.69 |
| 281 | Open Field / Number of Rears | Early adult | HET | Not significant | 1.33×10-2 |
| 282 | Open Field / Whole arena resting time series | Early adult | HET | Not significant | 0.61 |
| 283 | Open Field / Center resting time series | Early adult | HET | Not significant | 0.4 |
| 284 | Open Field / Percentage center movement time | Early adult | HET | Not significant | 0.87 |
| 285 | Open Field / Number of rears - total | Early adult | HET | Not significant | 0.15 |
| 286 | Open Field / Number of center entries series | Early adult | HET | Not significant | 0.78 |
| 287 | Open Field / Periphery distance travelled series | Early adult | HET | Not significant | 0.86 |
| 288 | Open Field / Whole arena average speed | Early adult | HET | Not significant | 0.28 |
| 289 | Open Field / Center average speed | Early adult | HET | Not significant | 5.20×10-3 |
| 290 | Tail Flick / Latency to tail flick | Early adult | HET | Not significant | 0.61 |
| 291 | Tail Flick / Average latency to tail flick | Early adult | HET | Not significant | 0.42 |
| 292 | Tissue Embedding and Block Banking / Spleen | Early adult | HET | Not significant | - |
| 293 | Tissue Embedding and Block Banking / Lung | Early adult | HET | Not significant | - |
| 294 | Tissue Embedding and Block Banking / Lymph node | Early adult | HET | Not significant | - |
| 295 | Tissue Embedding and Block Banking / Trachea | Early adult | HET | Not significant | - |
| 296 | Tissue Embedding and Block Banking / Ovary | Early adult | HET | Not significant | - |
| 297 | Tissue Embedding and Block Banking / Right Adrenal gland | Early adult | HET | Not significant | - |
| 298 | Tissue Embedding and Block Banking / Esophagus | Early adult | HET | Not significant | - |
| 299 | Tissue Embedding and Block Banking / Prostate | Early adult | HET | Not significant | - |
| 300 | Tissue Embedding and Block Banking / Testes | Early adult | HET | Not significant | - |
| 301 | Tissue Embedding and Block Banking / Gall bladder | Early adult | HET | Not significant | - |
| 302 | Tissue Embedding and Block Banking / Heart | Early adult | HET | Not significant | - |
| 303 | Tissue Embedding and Block Banking / Left Kidney | Early adult | HET | Not significant | - |
| 304 | Tissue Embedding and Block Banking / Eye 1 with optic nerve (optic nerve parallel to cassette surface) | Early adult | HET | Not significant | - |
| 305 | Tissue Embedding and Block Banking / Stomach | Early adult | HET | Not significant | - |
| 306 | Tissue Embedding and Block Banking / Eye 2 with optic nerve (within skull) | Early adult | HET | Not significant | - |
| 307 | Tissue Embedding and Block Banking / Mammary gland | Early adult | HET | Not significant | - |
| 308 | Tissue Embedding and Block Banking / Urinary bladder | Early adult | HET | Not significant | - |
| 309 | Tissue Embedding and Block Banking / Seminal vesicles | Early adult | HET | Not significant | - |
| 310 | Tissue Embedding and Block Banking / Thyroid | Early adult | HET | Not significant | - |
| 311 | Tissue Embedding and Block Banking / Right Kidney | Early adult | HET | Not significant | - |
| 312 | Tissue Embedding and Block Banking / Left Adrenal gland | Early adult | HET | Not significant | - |
| 313 | Tissue Embedding and Block Banking / Thymus | Early adult | HET | Not significant | - |
| 314 | Tissue Embedding and Block Banking / Skin | Early adult | HET | Not significant | - |
| 315 | Tissue Embedding and Block Banking / Skeletal muscle | Early adult | HET | Not significant | - |
| 316 | Tissue Embedding and Block Banking / Liver | Early adult | HET | Not significant | - |
| 317 | Tissue Embedding and Block Banking / Small intestine | Early adult | HET | Not significant | - |
| 318 | Tissue Embedding and Block Banking / Large intestine | Early adult | HET | Not significant | - |
| 319 | Tissue Embedding and Block Banking / Pancreas | Early adult | HET | Not significant | - |
| 320 | Tissue Embedding and Block Banking / Epididymis | Early adult | HET | Not significant | - |
| 321 | Tissue Embedding and Block Banking / Uterus | Early adult | HET | Not significant | - |
| 322 | Tissue Embedding and Block Banking / Spinal cord | Early adult | HET | Not significant | - |
| 323 | Tissue Embedding and Block Banking / Brain | Early adult | HET | Not significant | - |
| 324 | Viability Primary Screen / Number of heterozygous males | Early adult | HET | Not significant | - |
| 325 | Viability Primary Screen / Heterozygous animals viability | Early adult | HET | Not significant | - |
| 326 | Viability Primary Screen / Number of heterozygous females | Early adult | HET | Not significant | - |
| 327 | Viability Primary Screen / Total heterozygous females | Early adult | HET | Not significant | - |
| 328 | Viability Primary Screen / Total heterozygotes | Early adult | HET | Not significant | - |
| 329 | Viability Primary Screen / Total heterozygous males | Early adult | HET | Not significant | - |
| 330 | X-ray / XRay Images Whole Body Lateral Orientation | Early adult | HET | Not significant | - |
| 331 | X-ray / XRay Images Skull Dorso Ventral Orientation | Early adult | HET | Not significant | - |
| 332 | X-ray / Tibia | Early adult | HET | Not significant | - |
| 333 | X-ray / Number of cervical vertebrae | Early adult | HET | Not significant | - |
| 334 | X-ray / Number of ribs right | Early adult | HET | Not significant | - |
| 335 | X-ray / Clavicle | Early adult | HET | Not significant | - |
| 336 | X-ray / Processes on vertebrae | Early adult | HET | Not significant | - |
| 337 | X-ray / Skull shape | Early adult | HET | Not significant | 0.19 |
| 338 | X-ray / Comment on XRay image | Early adult | HET | Not significant | - |
| 339 | X-ray / Syndactylism | Early adult | HET | Not significant | - |
| 340 | X-ray / Fusion of vertebrae | Early adult | HET | Not significant | 1 |
| 341 | X-ray / Brachydactyly | Early adult | HET | Not significant | - |
| 342 | X-ray / Shape of vertebrae | Early adult | HET | Not significant | - |
| 343 | X-ray / XRay Images Skull Lateral Orientation | Early adult | HET | Not significant | - |
| 344 | X-ray / Zygomatic bone | Early adult | HET | Not significant | 1 |
| 345 | X-ray / Number of digits | Early adult | HET | Not significant | 1 |
| 346 | X-ray / Mandibles | Early adult | HET | Not significant | - |
| 347 | X-ray / Ulna | Early adult | HET | Not significant | - |
| 348 | X-ray / Number of ribs left | Early adult | HET | Not significant | - |
| 349 | X-ray / Maxilla/Pre-maxilla | Early adult | HET | Not significant | - |
| 350 | X-ray / Fibula | Early adult | HET | Not significant | - |
| 351 | X-ray / Joints | Early adult | HET | Not significant | - |
| 352 | X-ray / Pelvis | Early adult | HET | Not significant | 0.58 |
| 353 | X-ray / Humerus | Early adult | HET | Not significant | - |
| 354 | X-ray / Femur | Early adult | HET | Not significant | - |
| 355 | X-ray / Fusion of ribs | Early adult | HET | Not significant | - |
| 356 | X-ray / Tibia length | Early adult | HET | Not significant | 0.28 |
| 357 | X-ray / Scapulae | Early adult | HET | Not significant | - |
| 358 | X-ray / Number of thoracic vertebrae | Early adult | HET | Not significant | - |
| 359 | X-ray / Number of lumbar vertebrae | Early adult | HET | Not significant | - |
| 360 | X-ray / XRay Images Whole Body Dorso Ventral | Early adult | HET | Not significant | - |
| 361 | X-ray / Radius | Early adult | HET | Not significant | - |
| 362 | X-ray / Alive | Early adult | HET | Not significant | - |
| 363 | X-ray / Number of pelvic vertebrae | Early adult | HET | Not significant | - |
| 364 | X-ray / Teeth | Early adult | HET | Not significant | 1 |
| 365 | X-ray / Shape of ribs | Early adult | HET | Not significant | 1 |
| 366 | X-ray / Digit integrity | Early adult | HET | Not significant | 1 |
| 367 | Viability Primary Screen / Homozygous males viability | Early adult | HOM | Not significant | - |
| 368 | Viability Primary Screen / Number of WT males | Early adult | HOM | Not significant | - |
| 369 | Viability Primary Screen / Total homozygous females | Early adult | HOM | Not significant | - |
| 370 | Viability Primary Screen / Litter female parent 1 specimen id | Early adult | HOM | Not significant | - |
| 371 | Viability Primary Screen / Litter date of birth | Early adult | HOM | Not significant | - |
| 372 | Viability Primary Screen / Number of WT females | Early adult | HOM | Not significant | - |
| 373 | Viability Primary Screen / Total WT males | Early adult | HOM | Not significant | - |
| 374 | Viability Primary Screen / Homozygous animals viability | Early adult | HOM | Not significant | - |
| 375 | Viability Primary Screen / Total homozygous males | Early adult | HOM | Not significant | - |
| 376 | Viability Primary Screen / Homozygous females viability | Early adult | HOM | Not significant | - |
| 377 | Viability Primary Screen / Number of homozygous males | Early adult | HOM | Not significant | - |
| 378 | Viability Primary Screen / Total WT females | Early adult | HOM | Not significant | - |
| 379 | Viability Primary Screen / Litter male parent specimen id | Early adult | HOM | Not significant | - |
| 380 | Viability Primary Screen / Total homozygotes | Early adult | HOM | Not significant | - |
| 381 | Viability Primary Screen / Number of homozygous females | Early adult | HOM | Not significant | - |
| 382 | Viability Primary Screen / Total WTs | Early adult | HOM | Not significant | - |
| 383 | Viability Primary Screen / Total females | Early adult | N/A | Not significant | - |
| 384 | Viability Primary Screen / Total males | Early adult | N/A | Not significant | - |
| 385 | Viability Primary Screen / Total pups | Early adult | N/A | Not significant | - |

**Supplementary ORA-report 1.** Over representation analyses using discriminating proteins from Mann-Whitney-Wilcoxon test reported in Table 1.

**
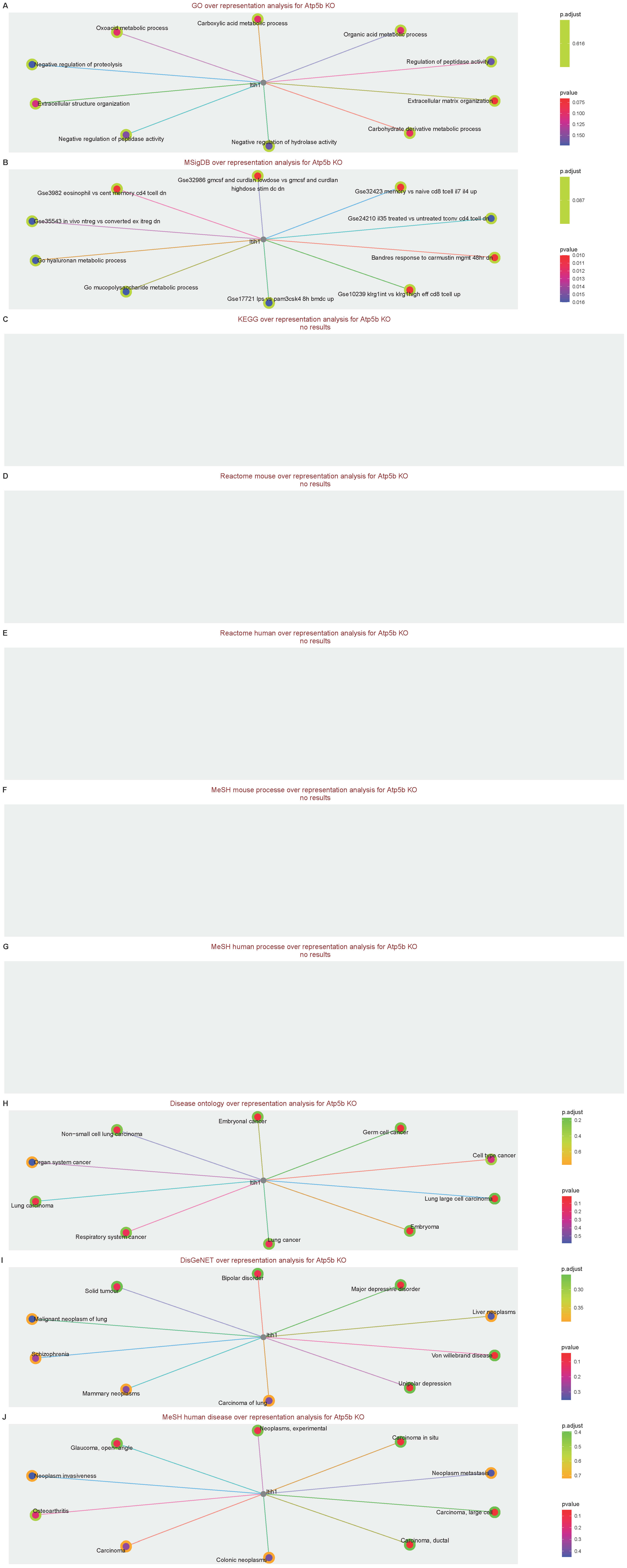
**

**
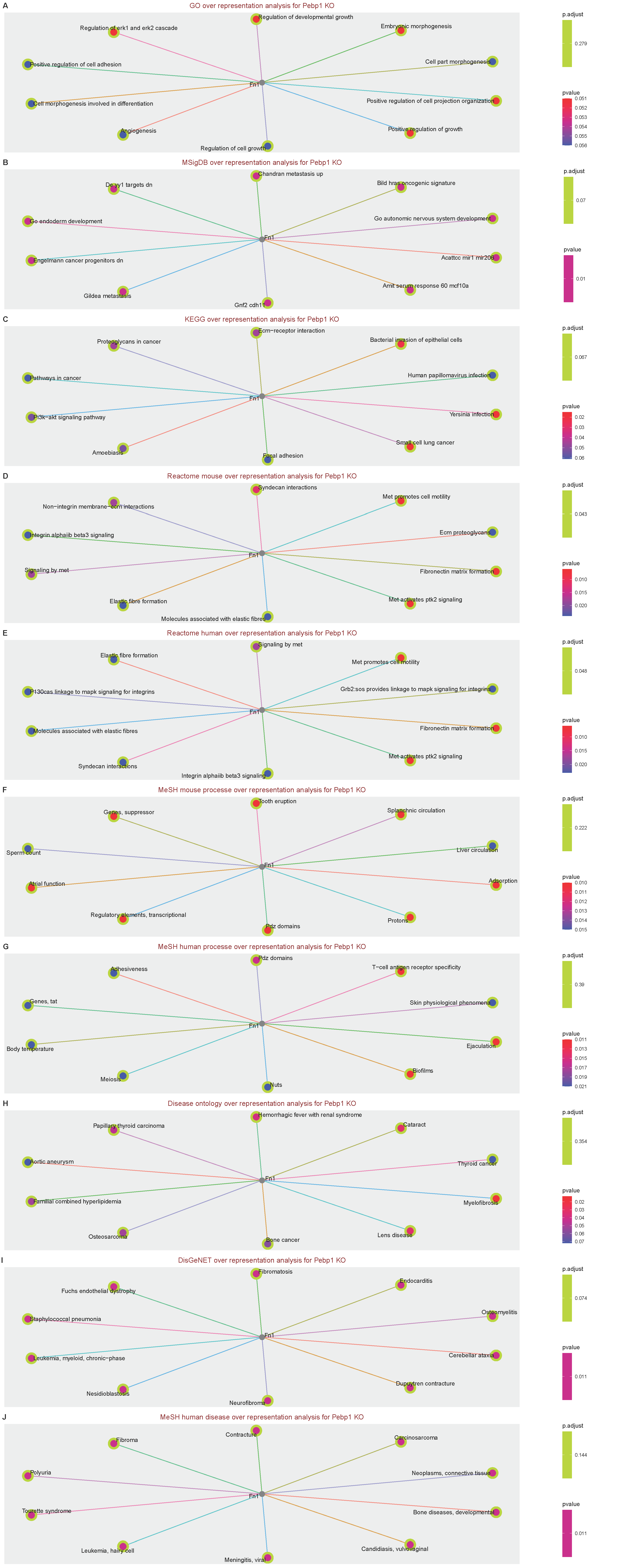
**

**
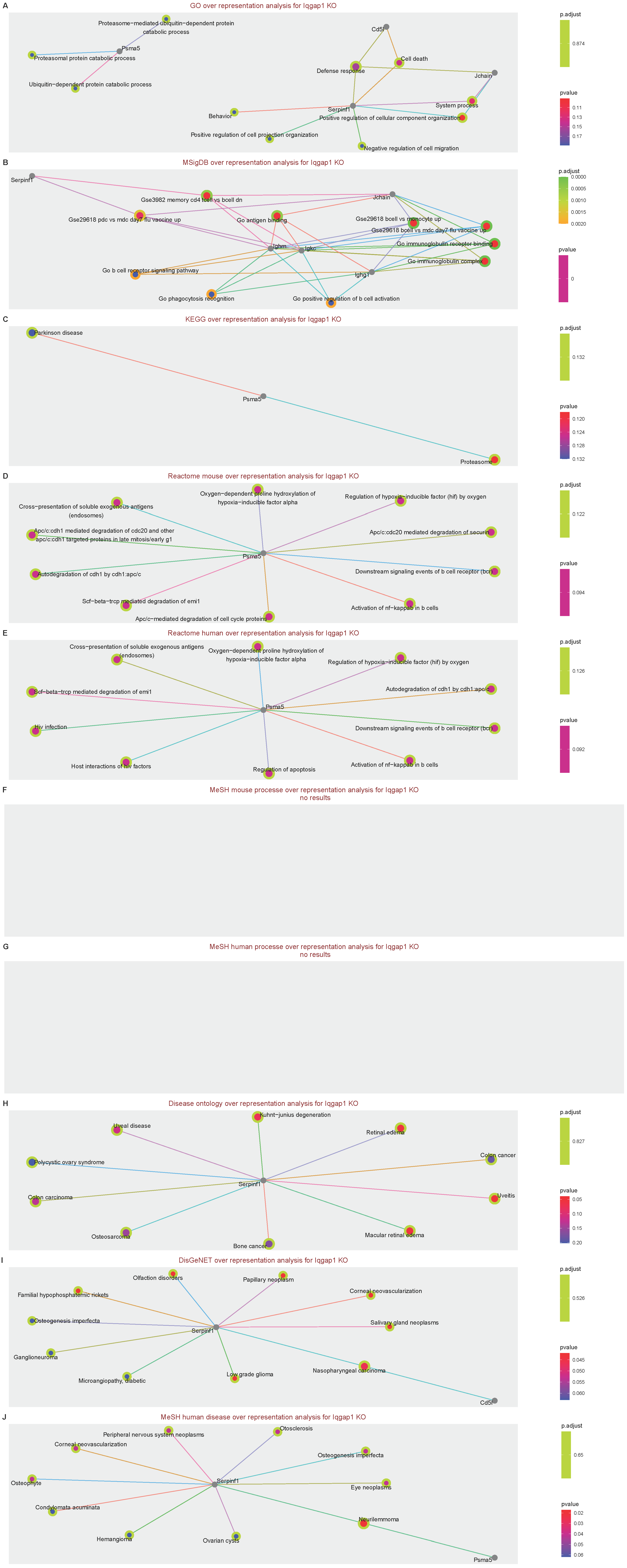
**

**
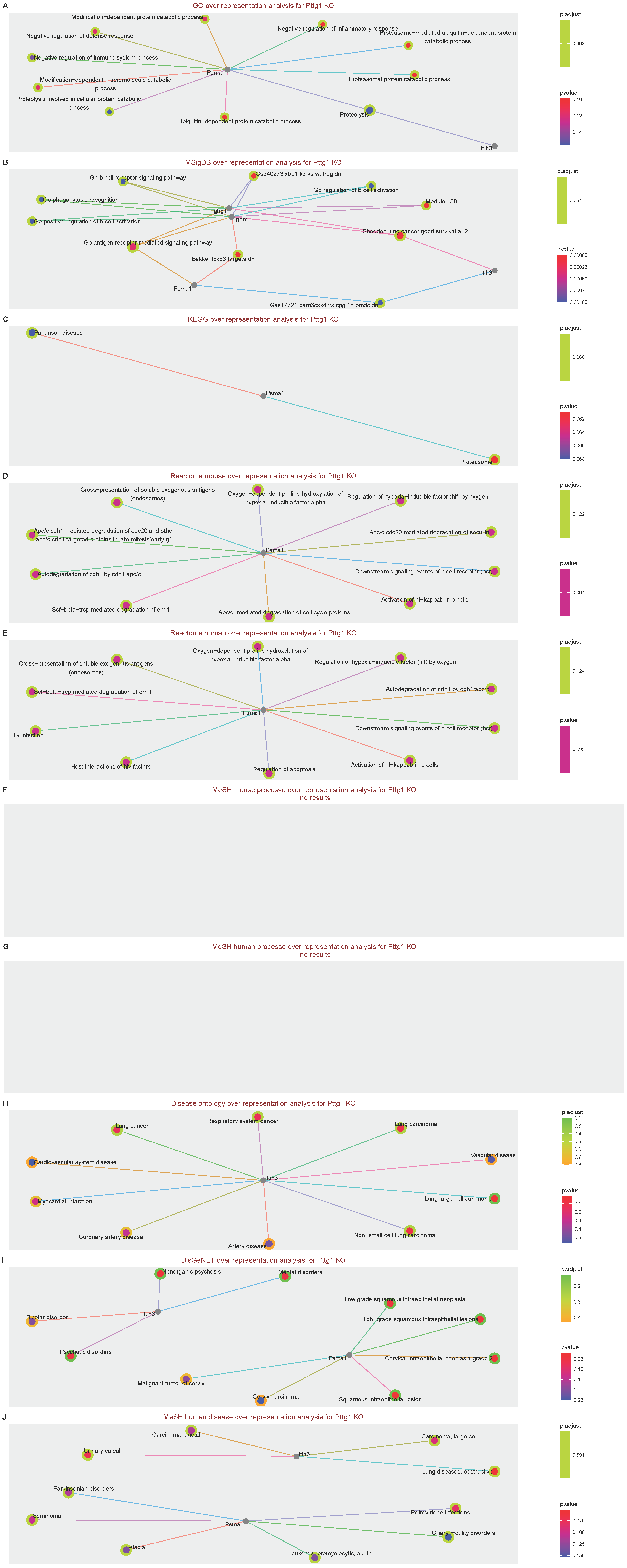
**

**
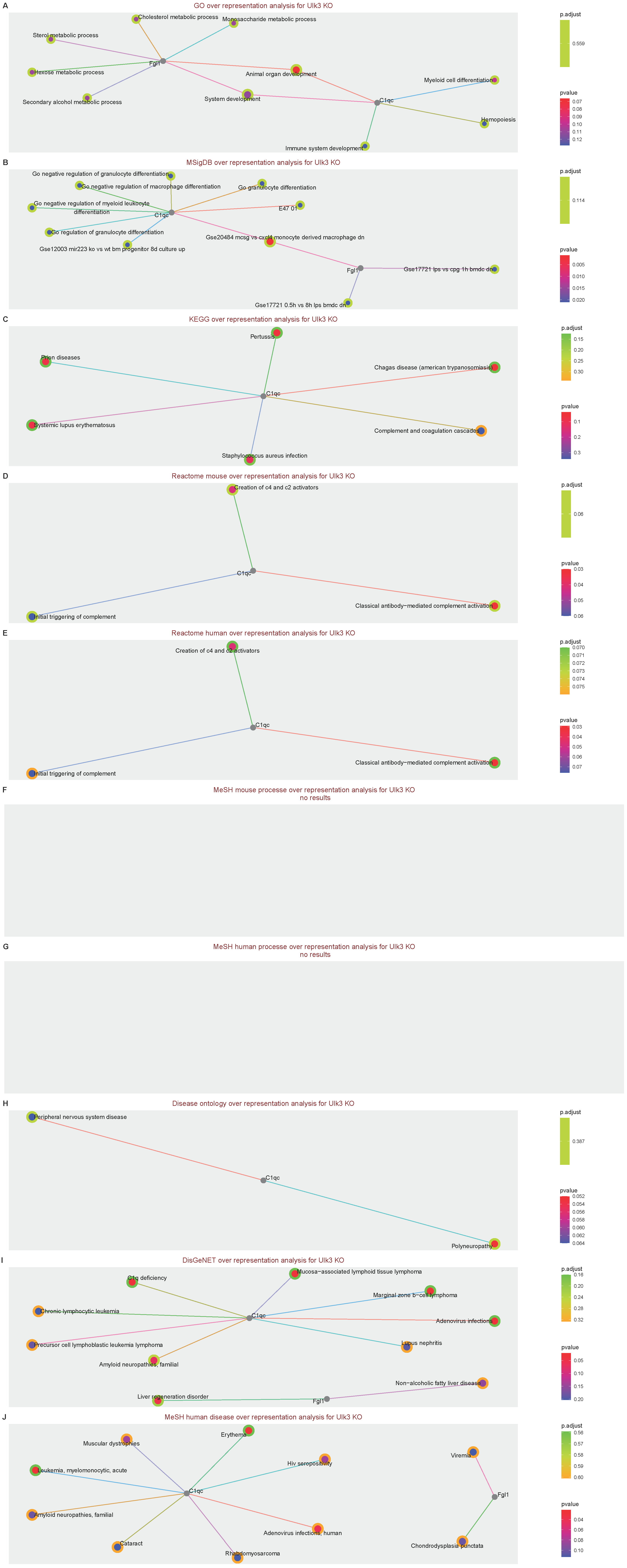
**

**
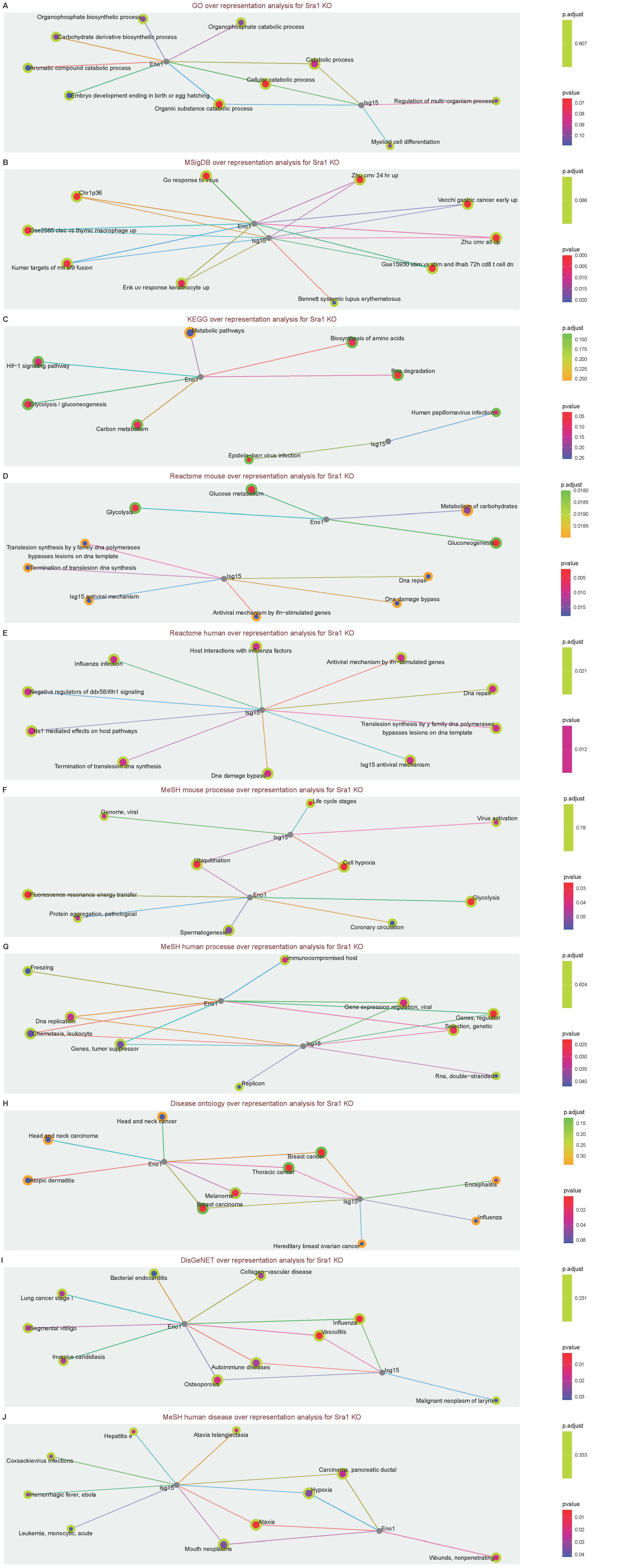
**

**
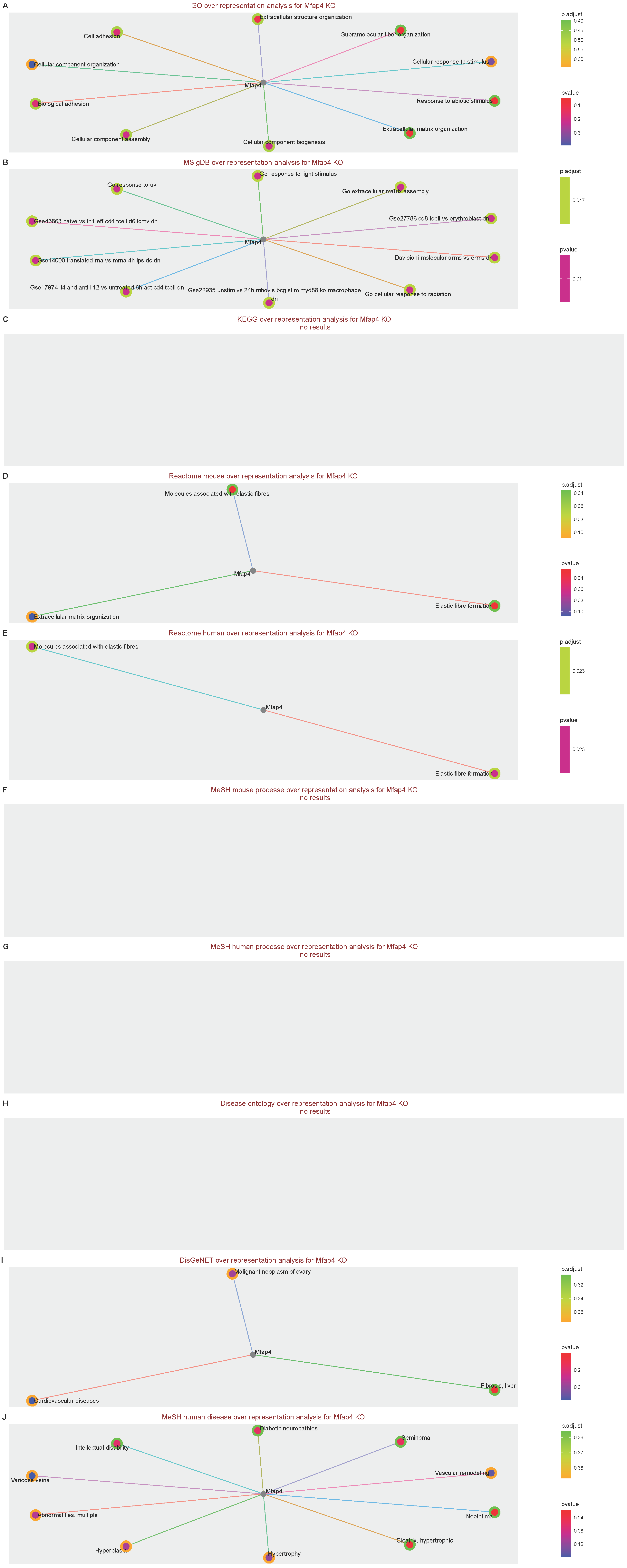
**

**
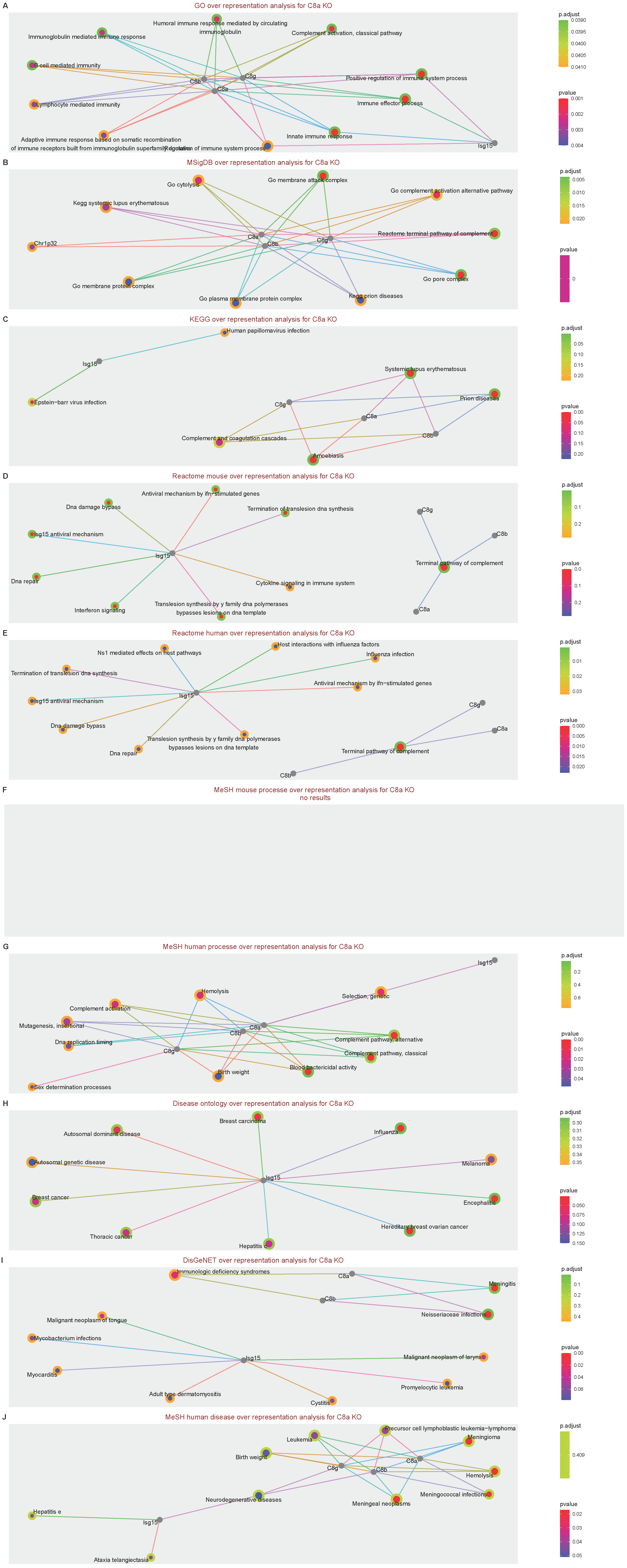
**

**
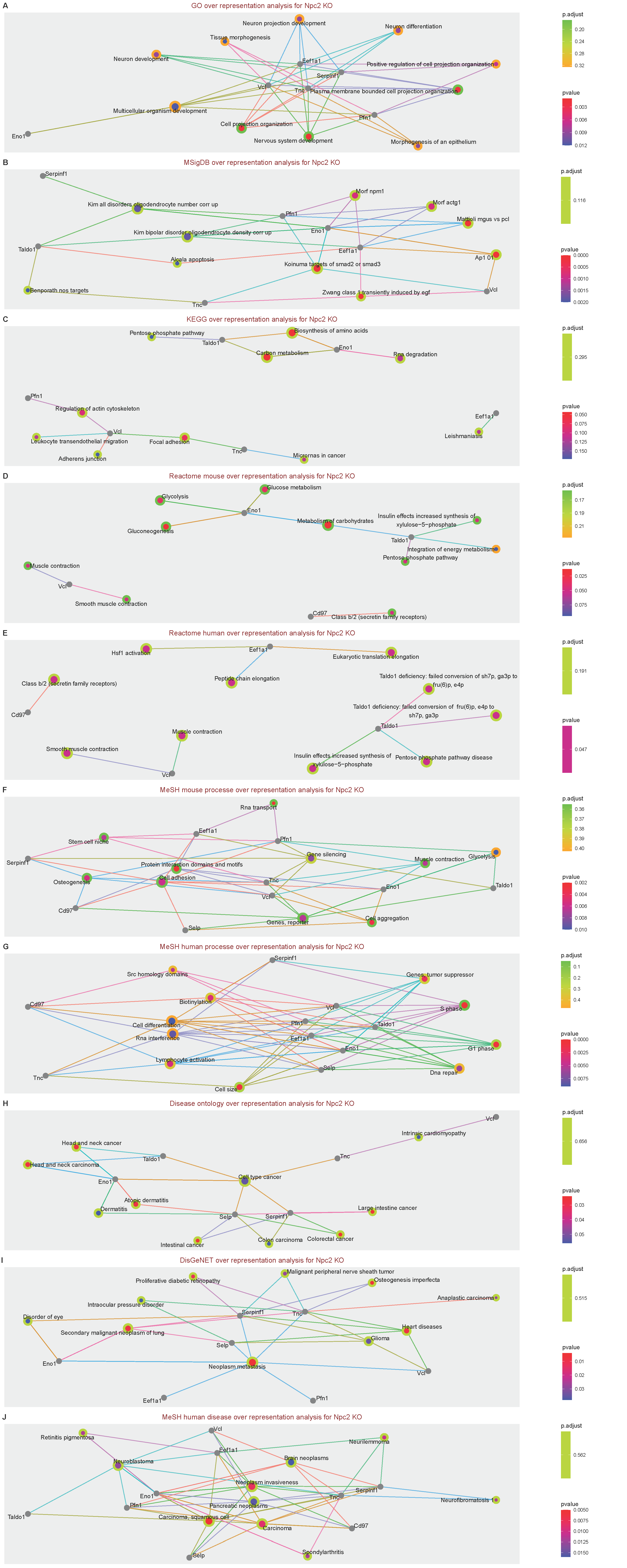
**

**
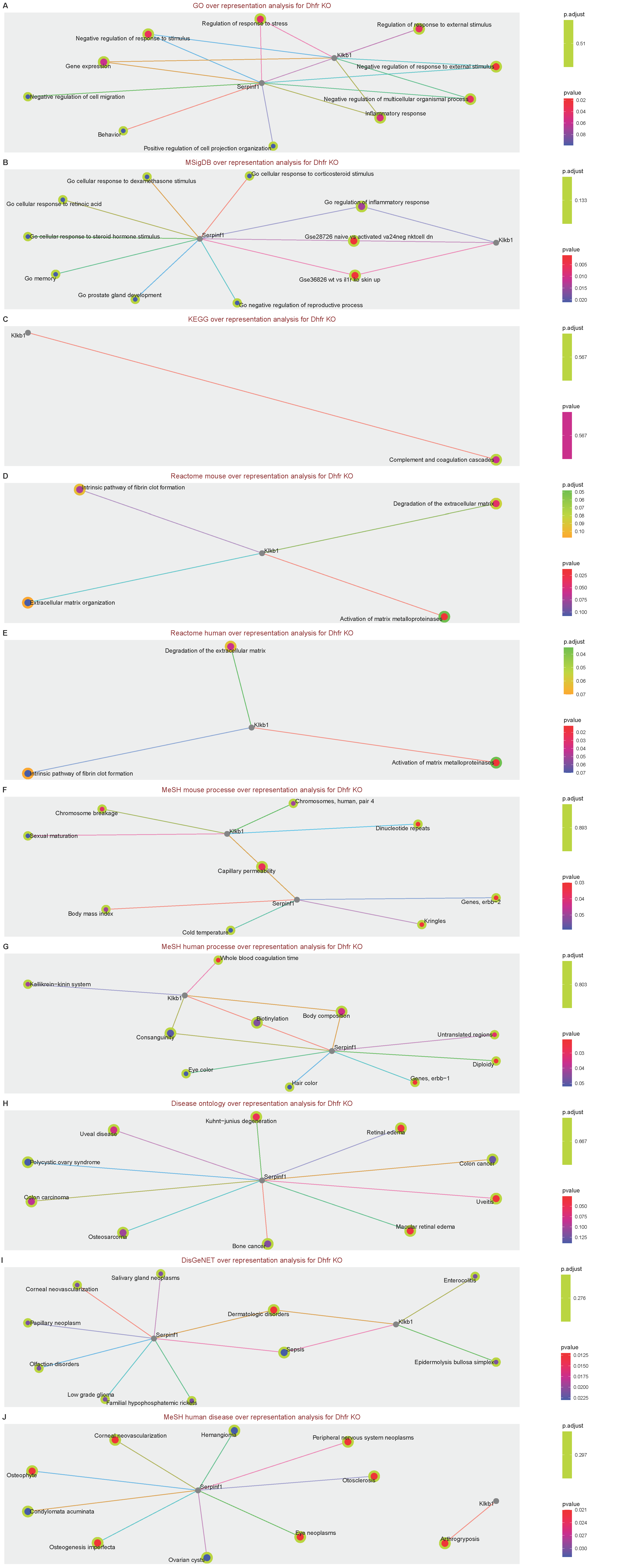
**

**
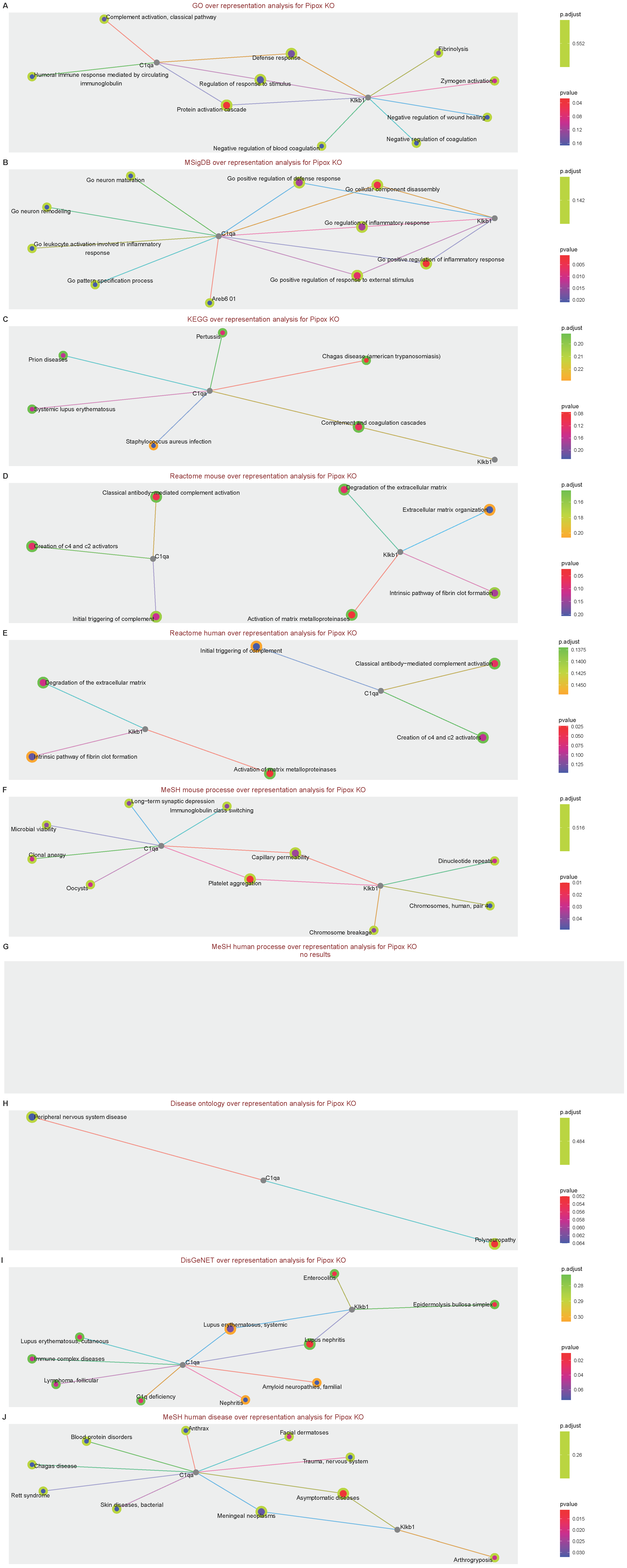
**

**
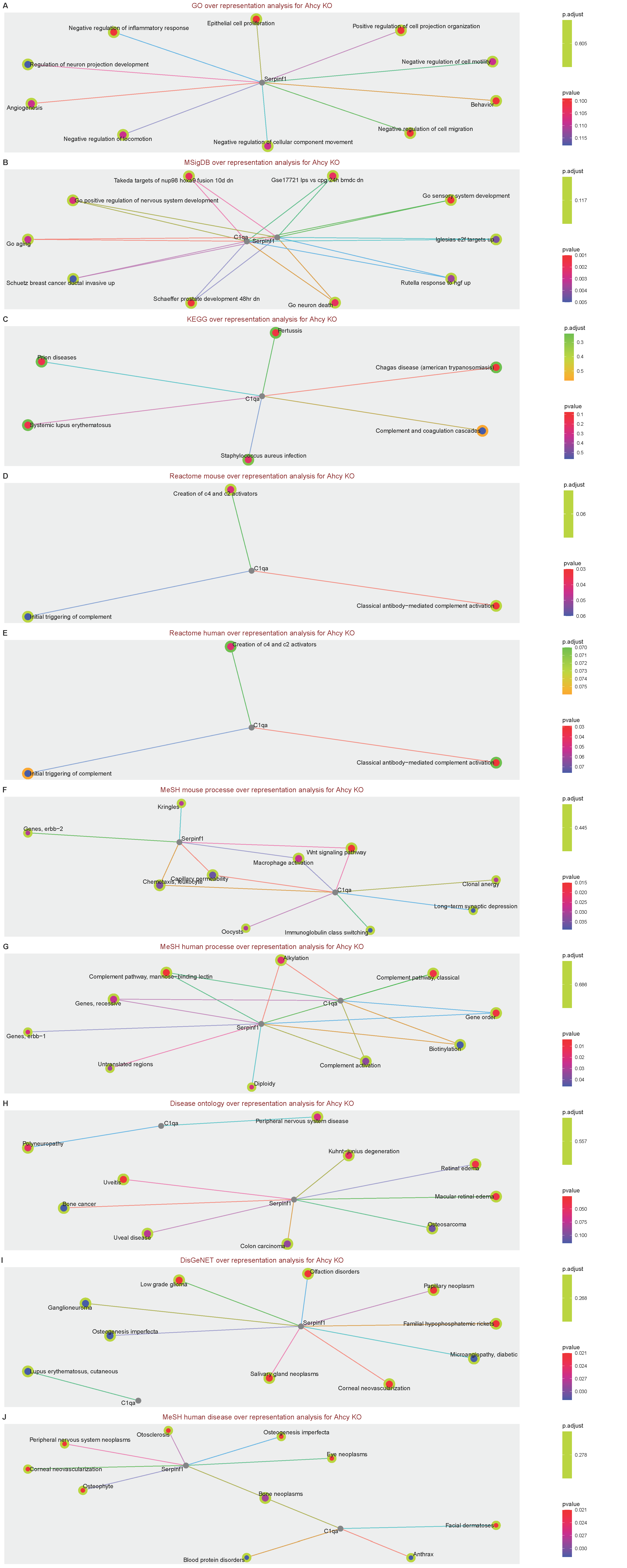
**

**
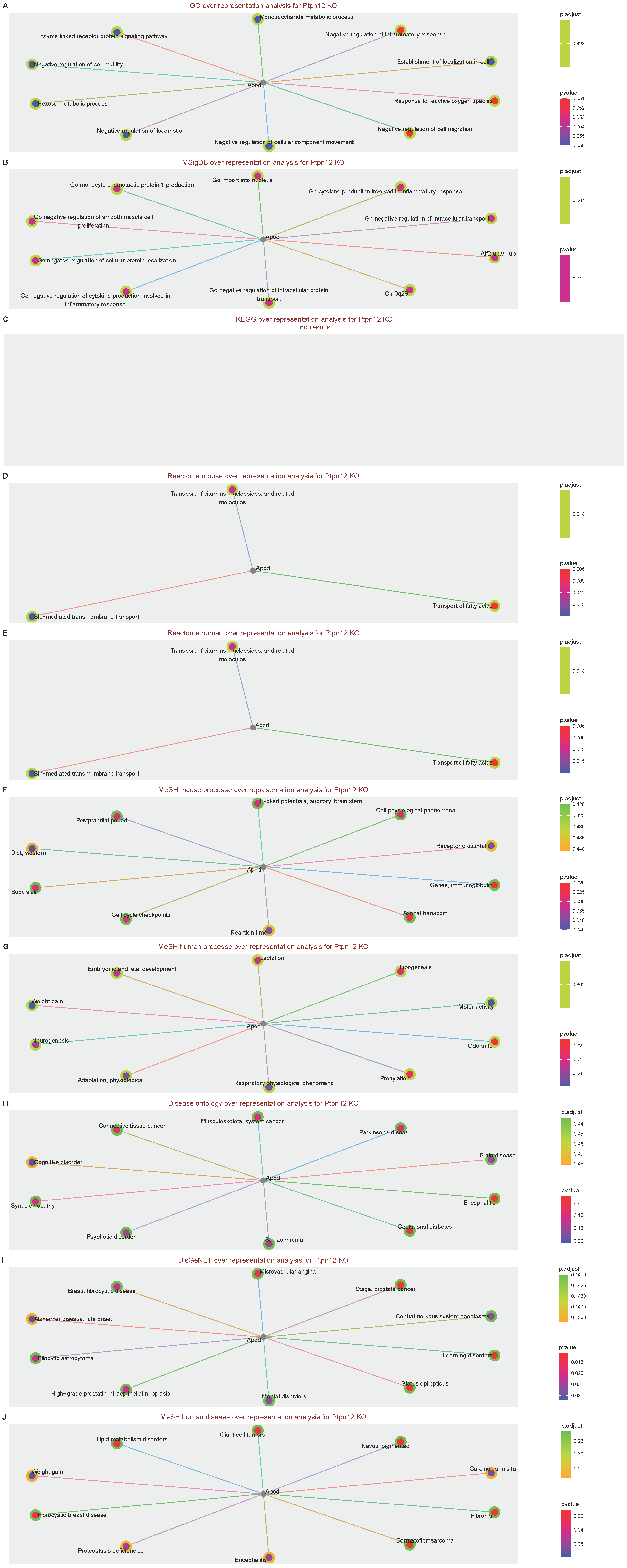
**

**Supplementary ORA-report 2.** Over representation analyses using combined discriminating protein lists from Mann-Whitney-Wilcoxon test (Table 1) and LASSO regression (Supplementary Table 2).

**
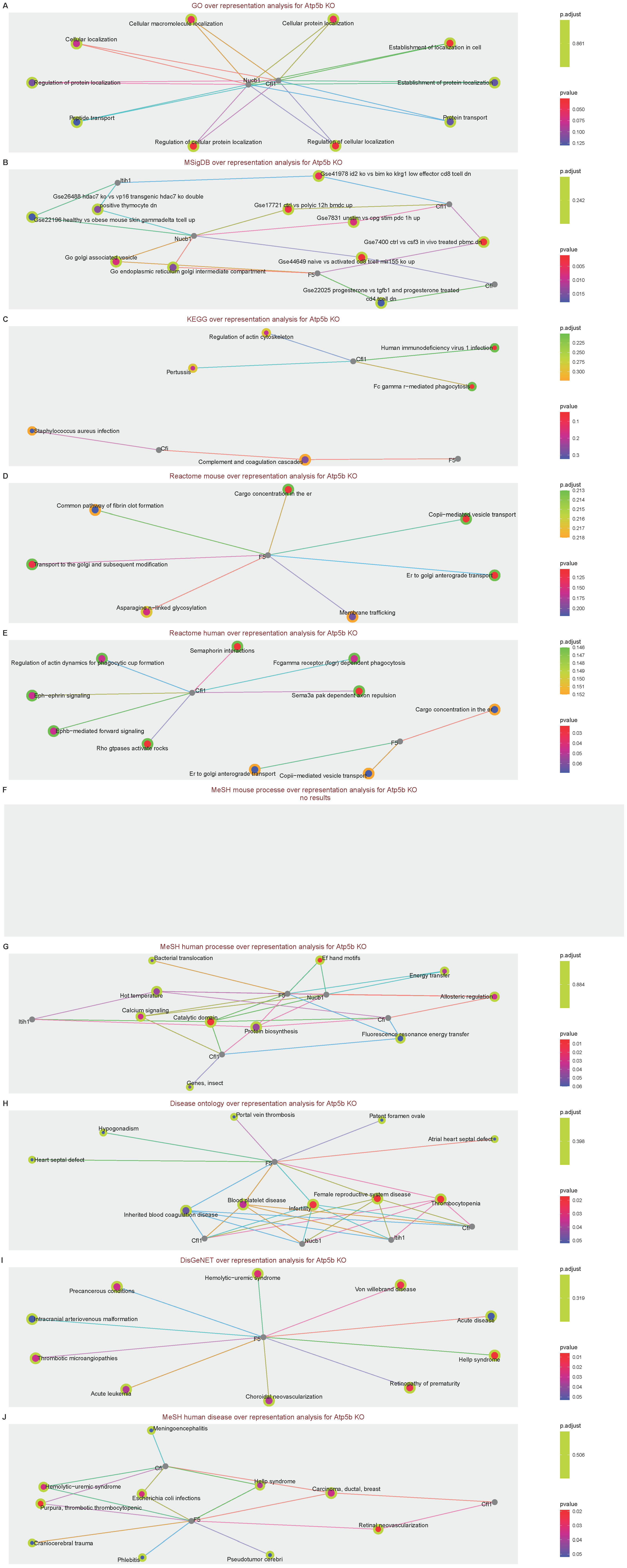
**

**
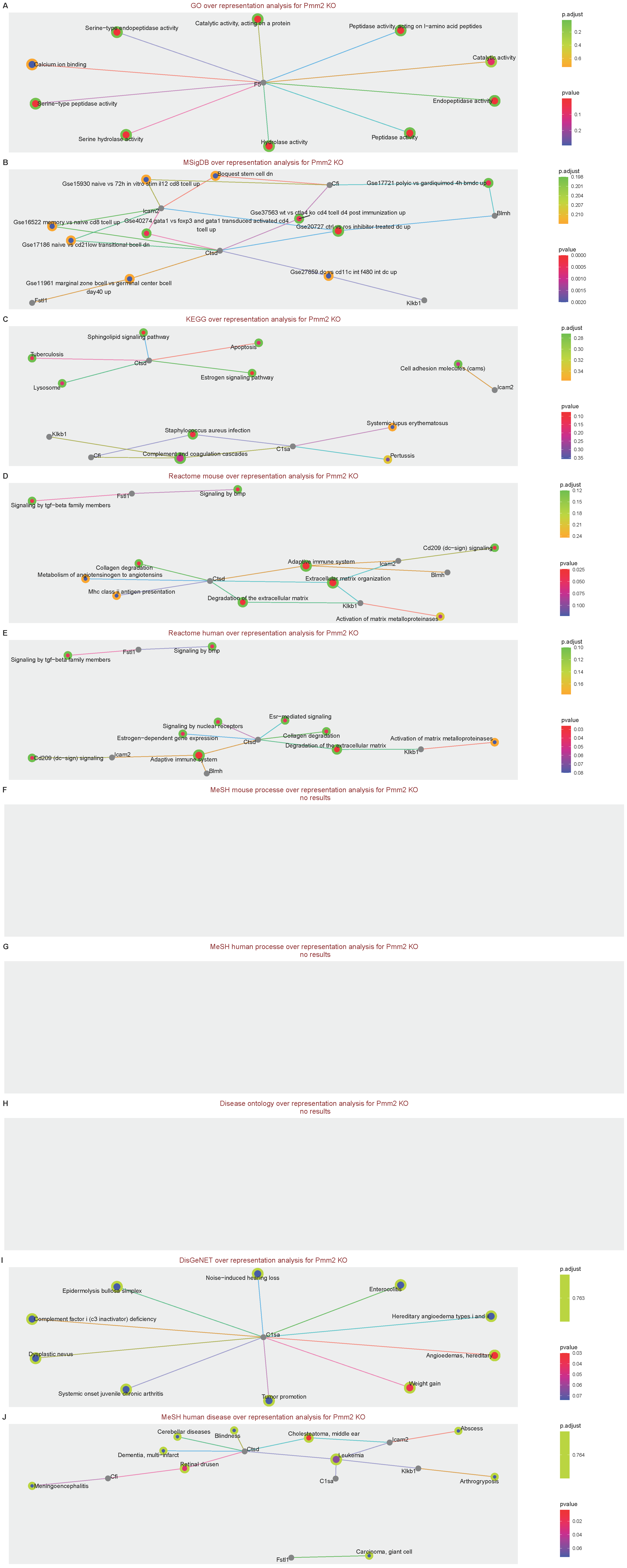
**

**
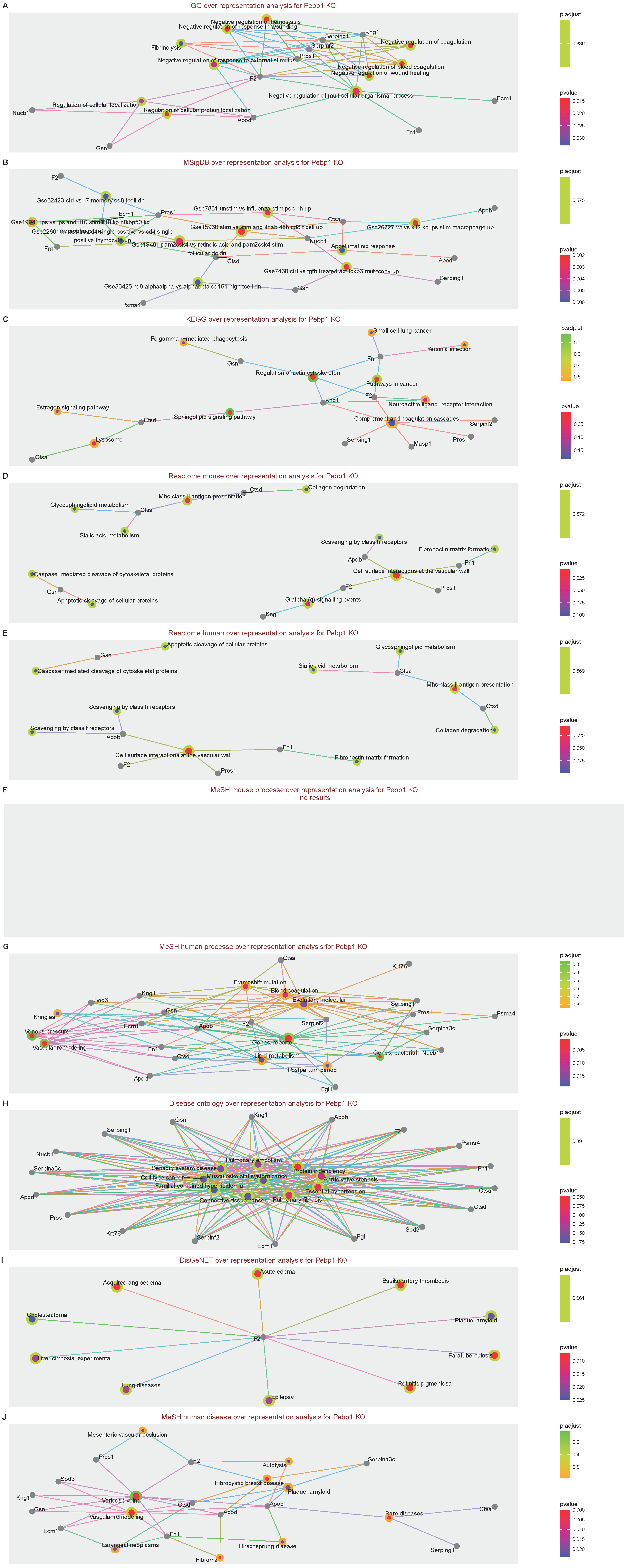
**


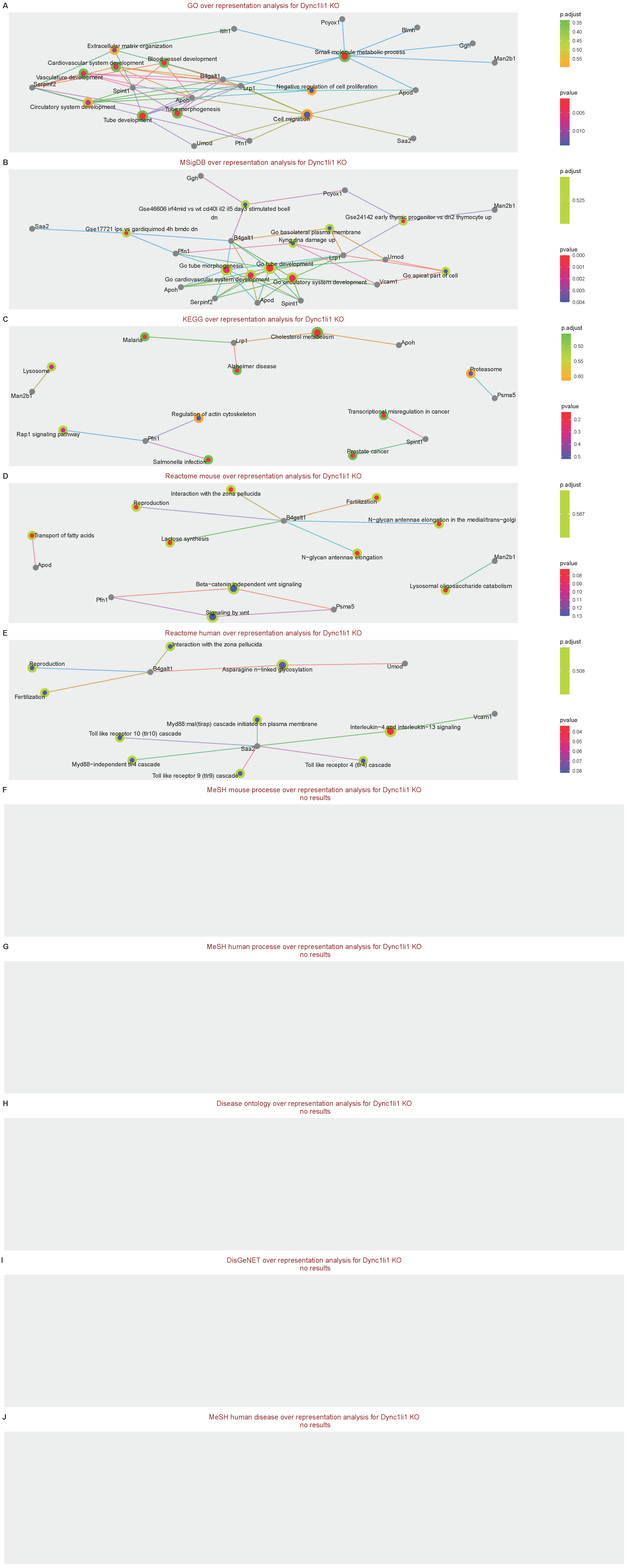

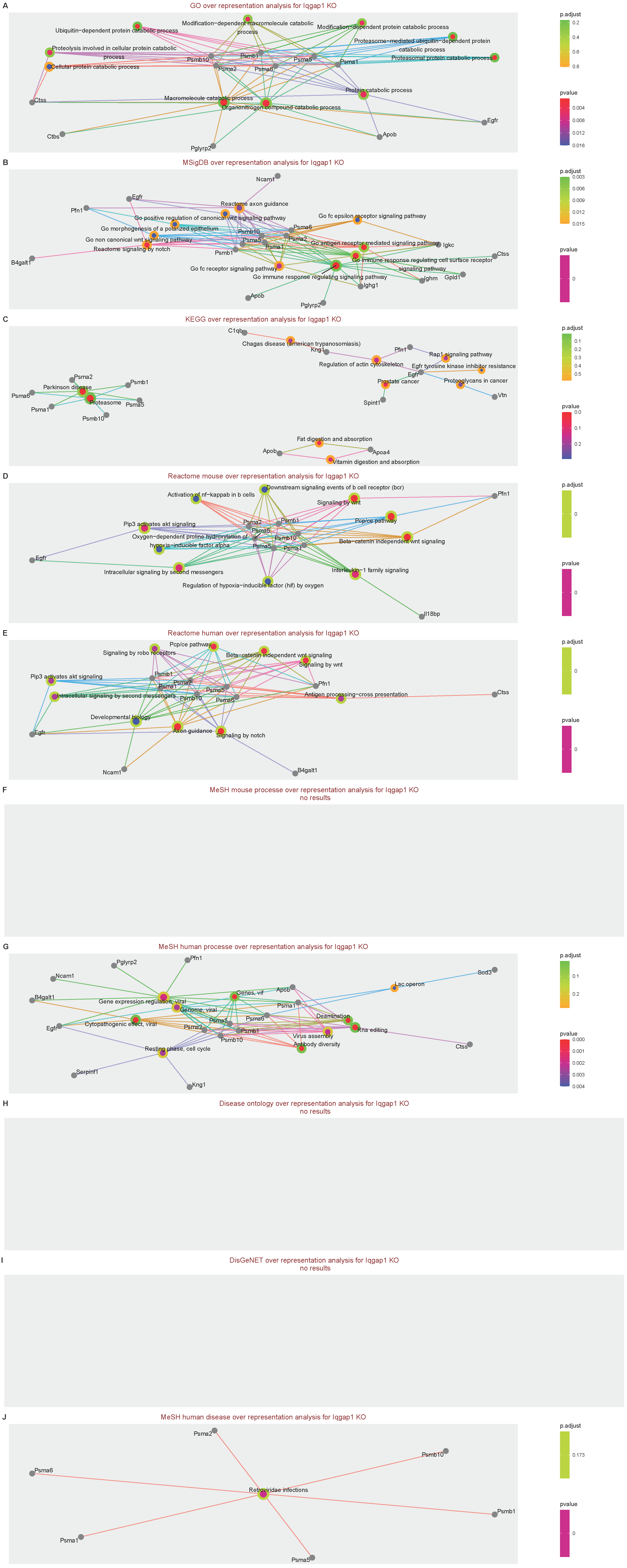

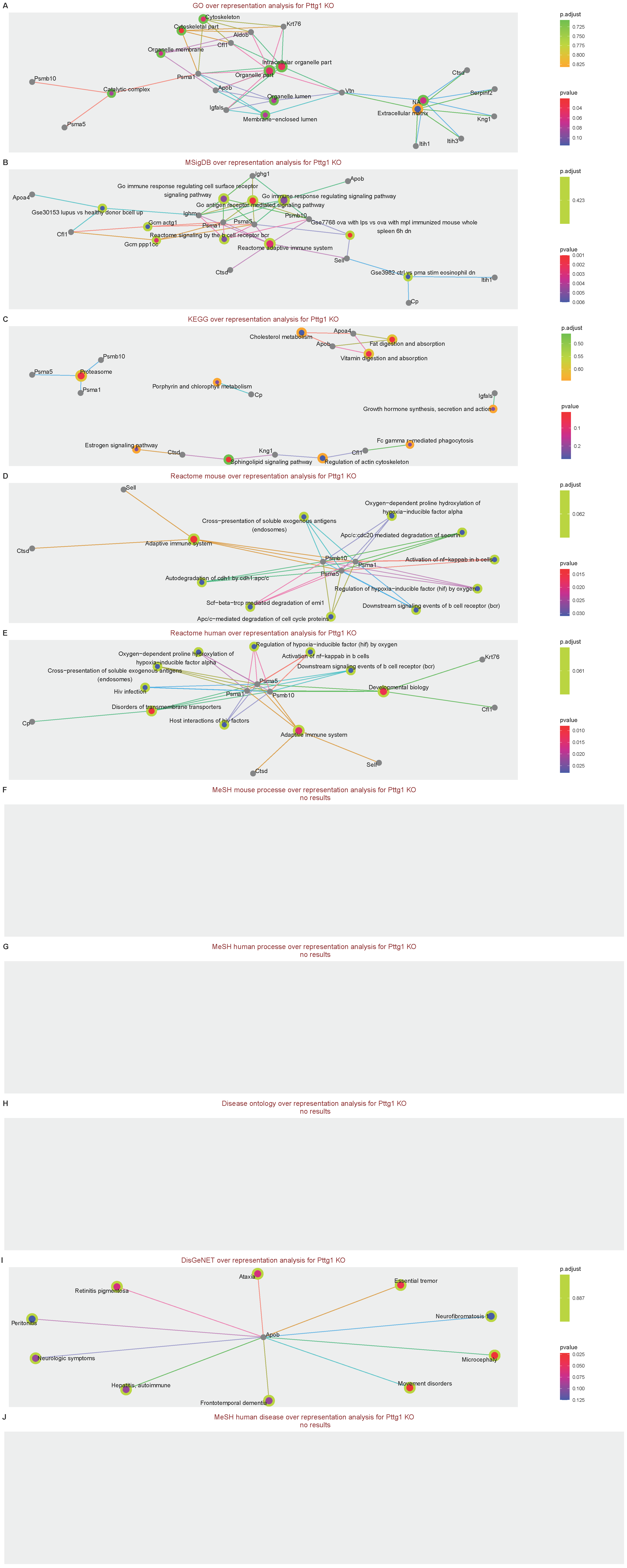

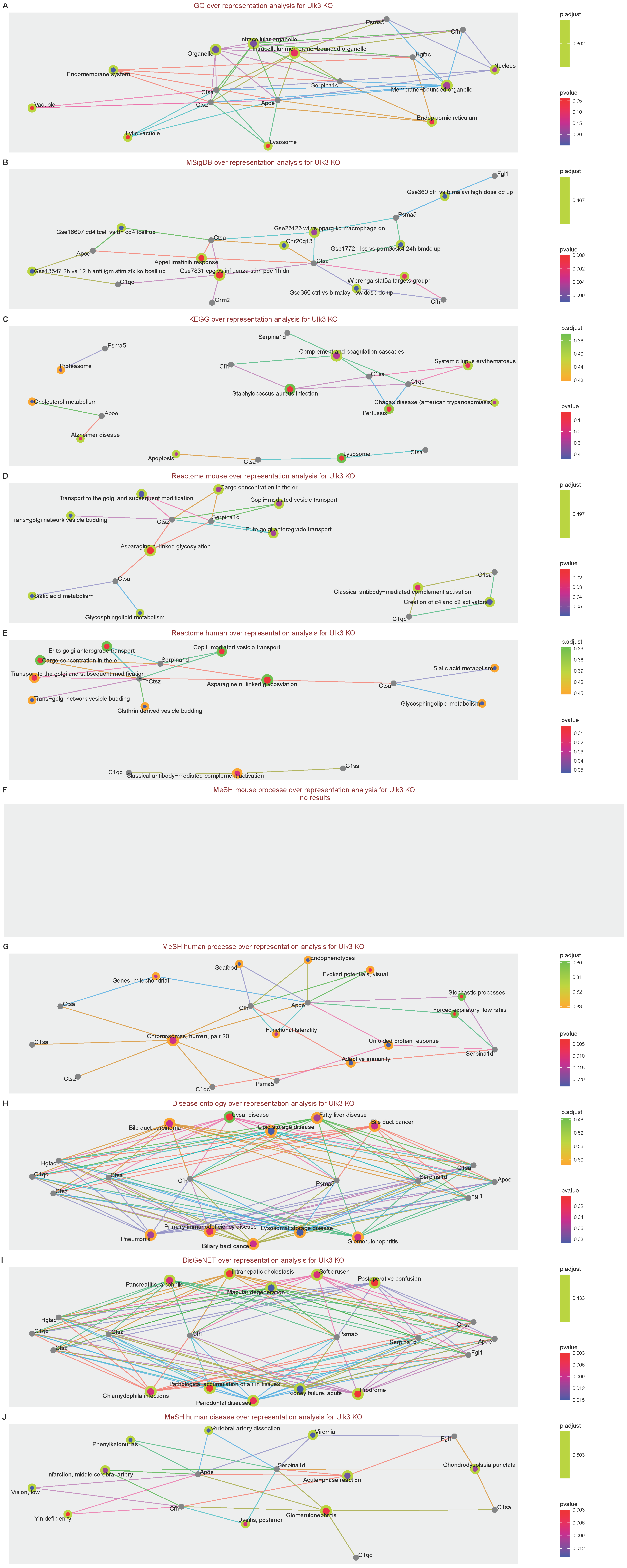


**
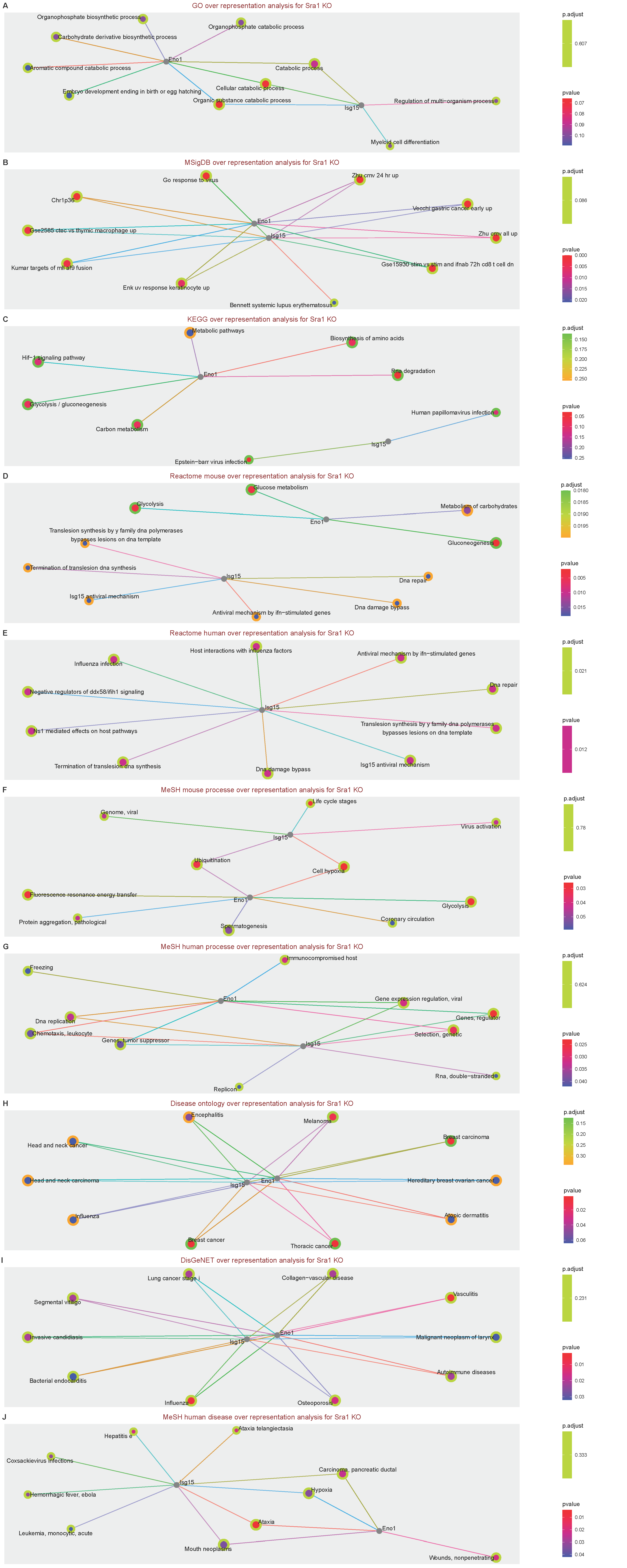
**


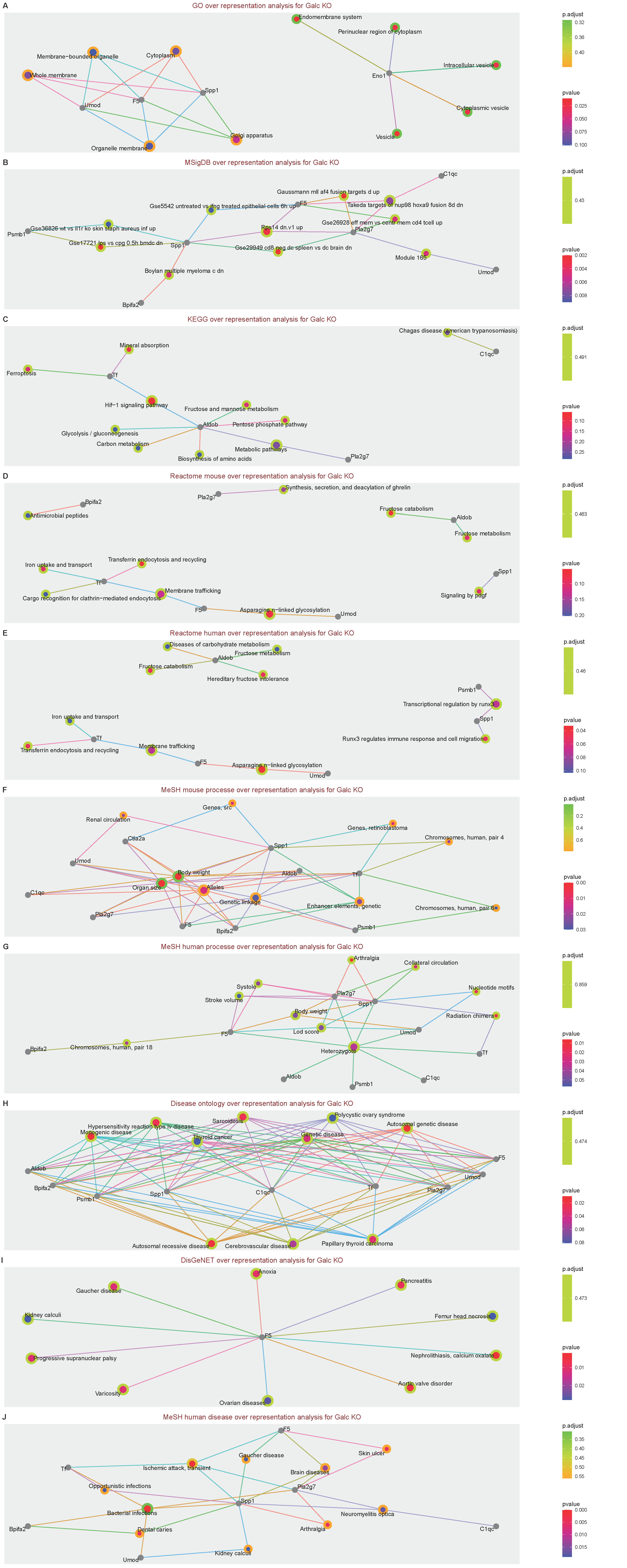

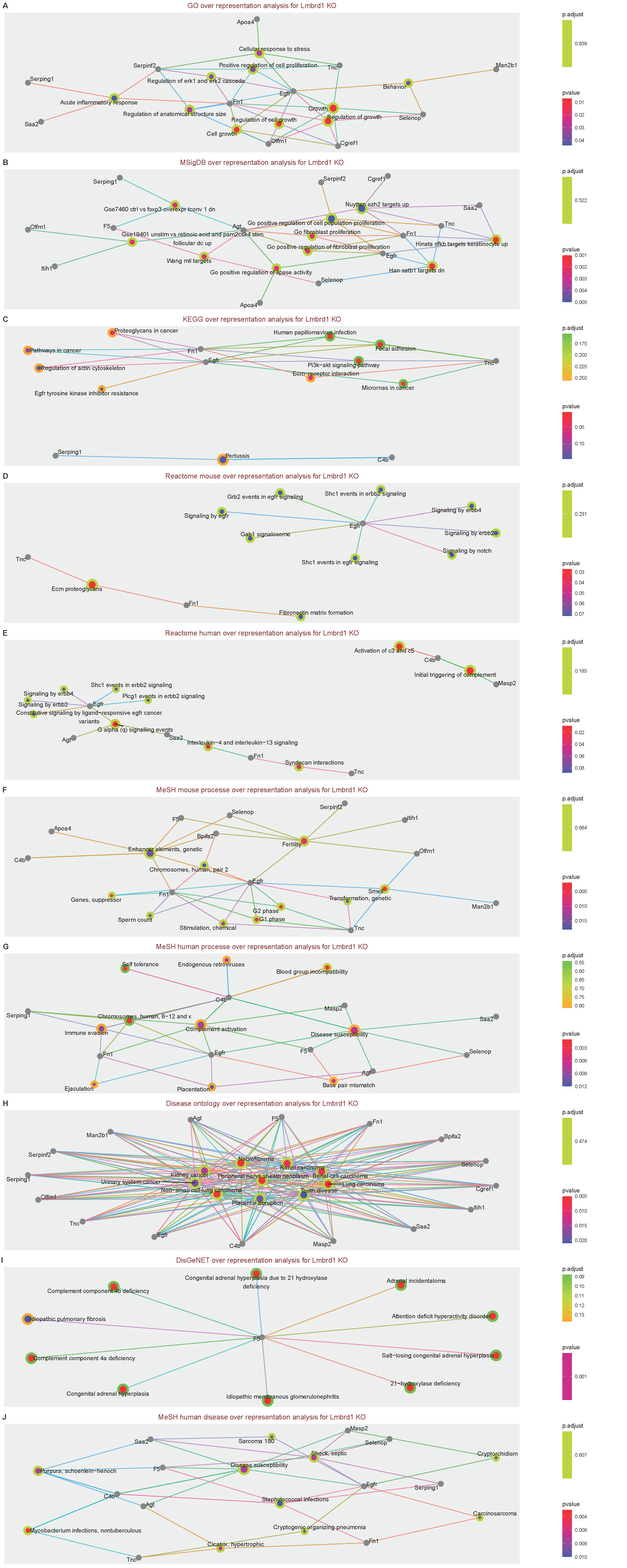

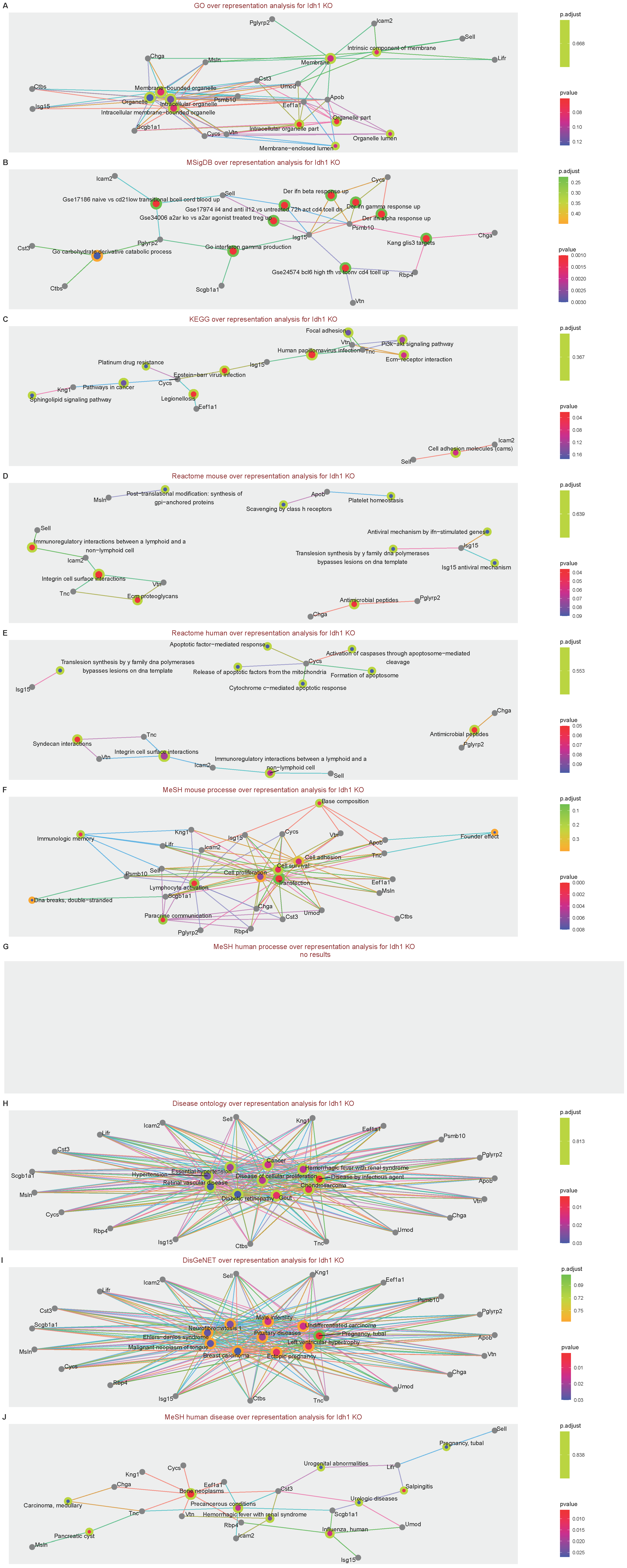

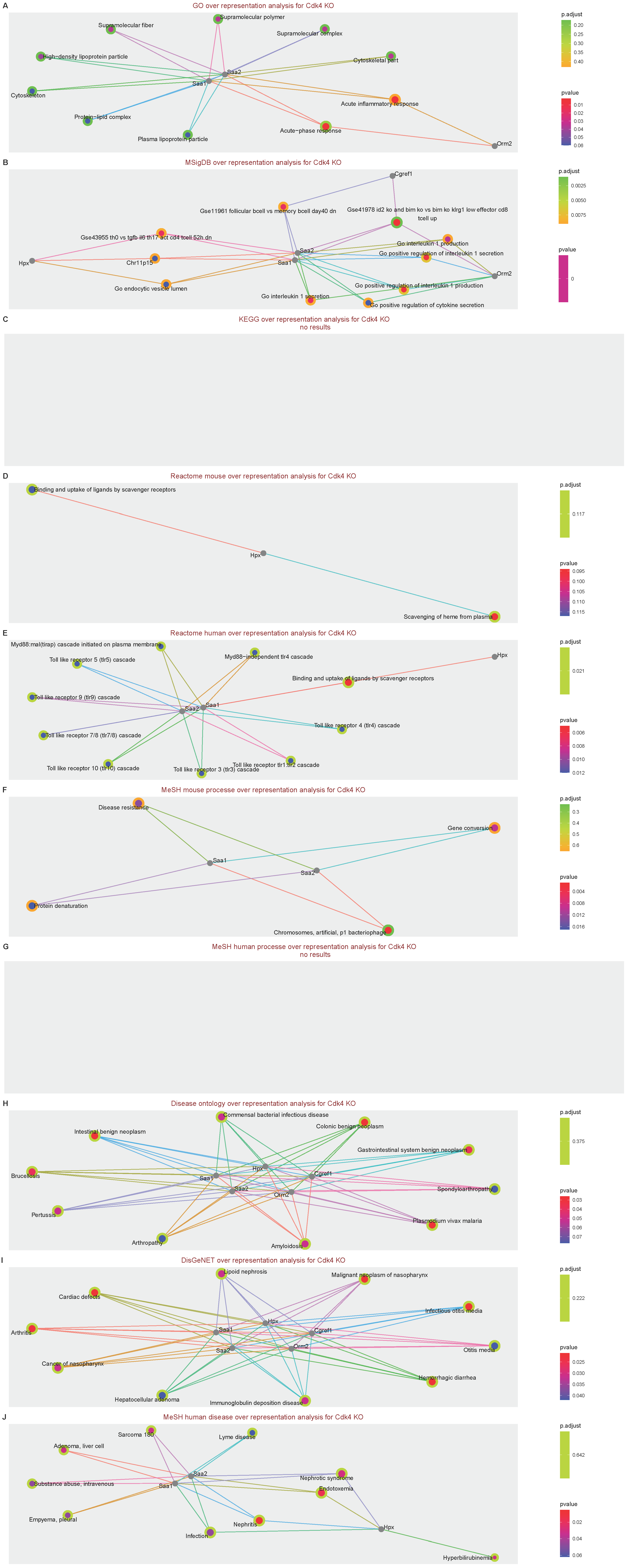

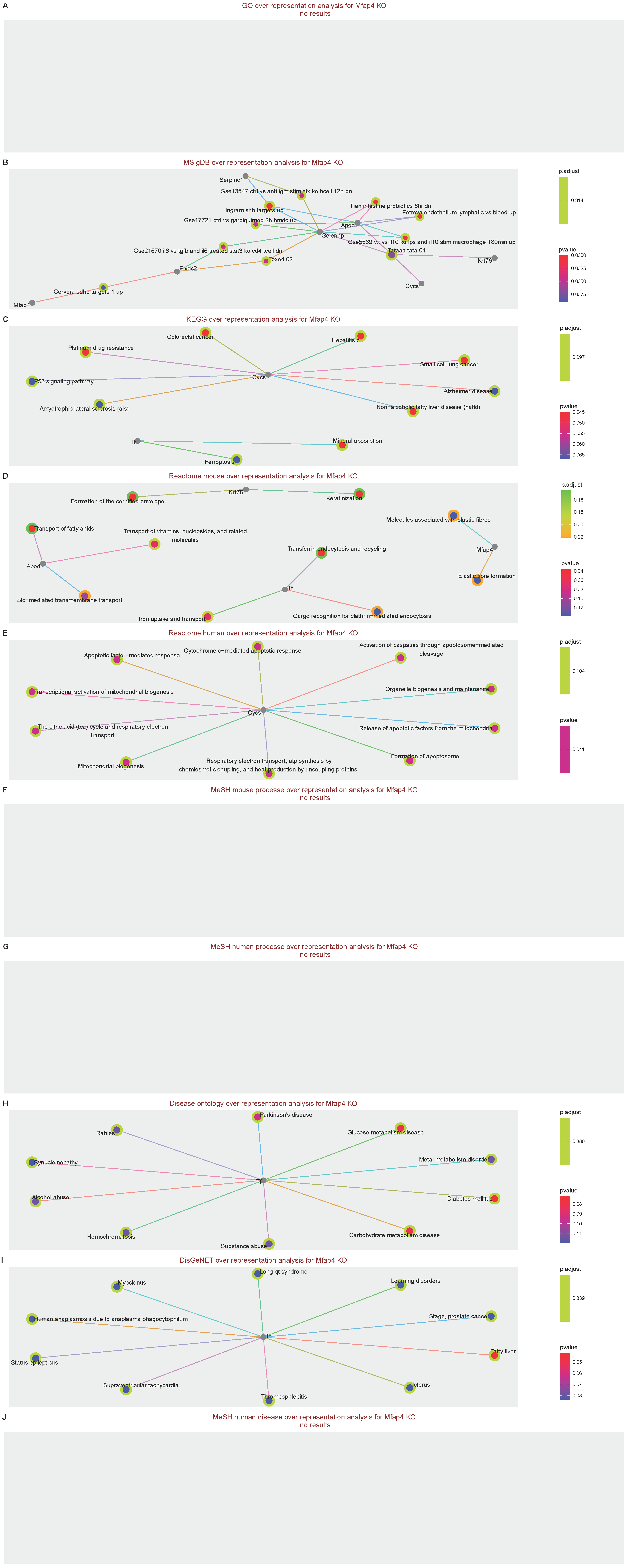

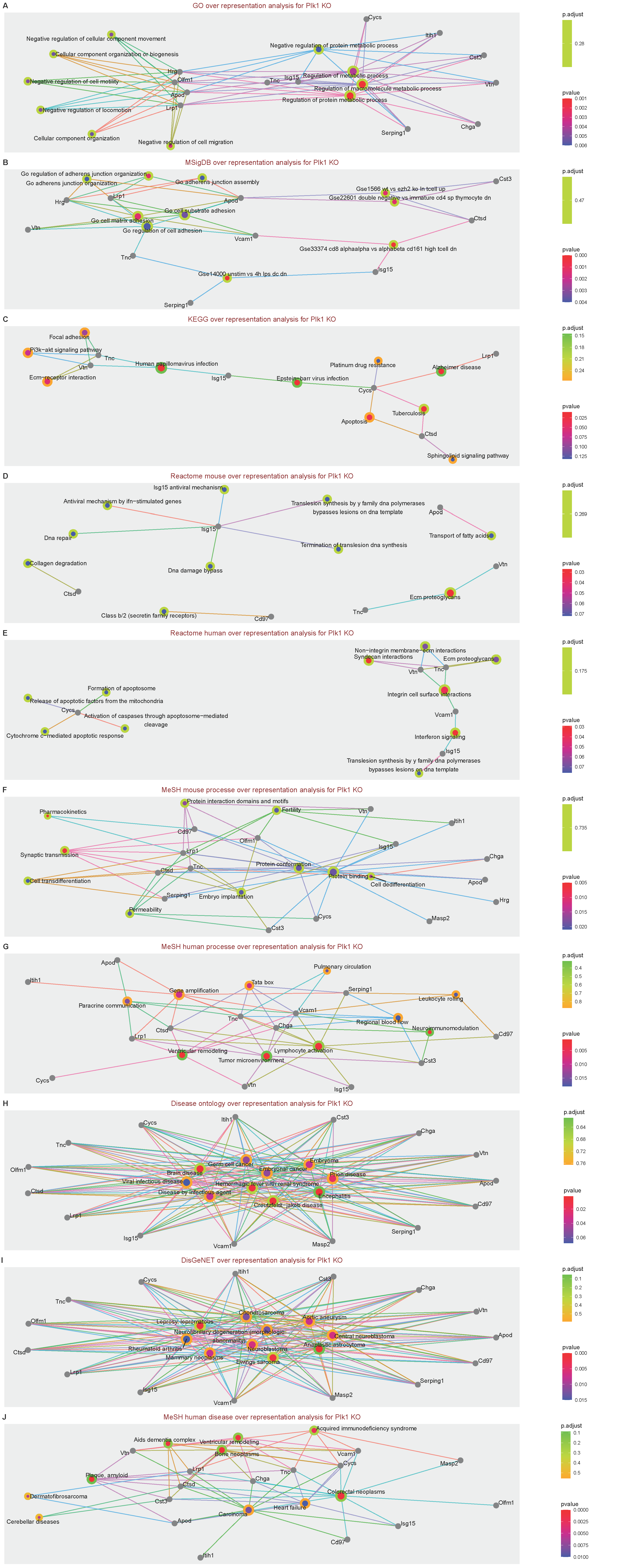

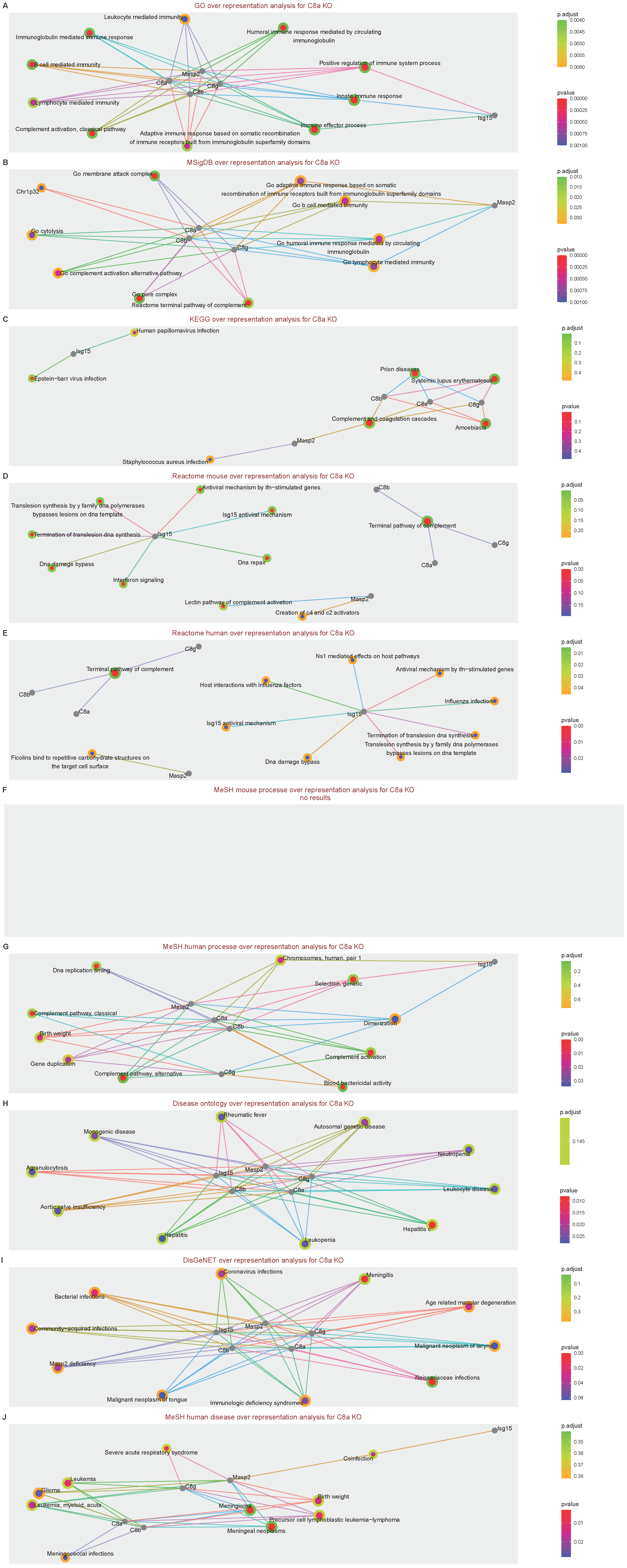

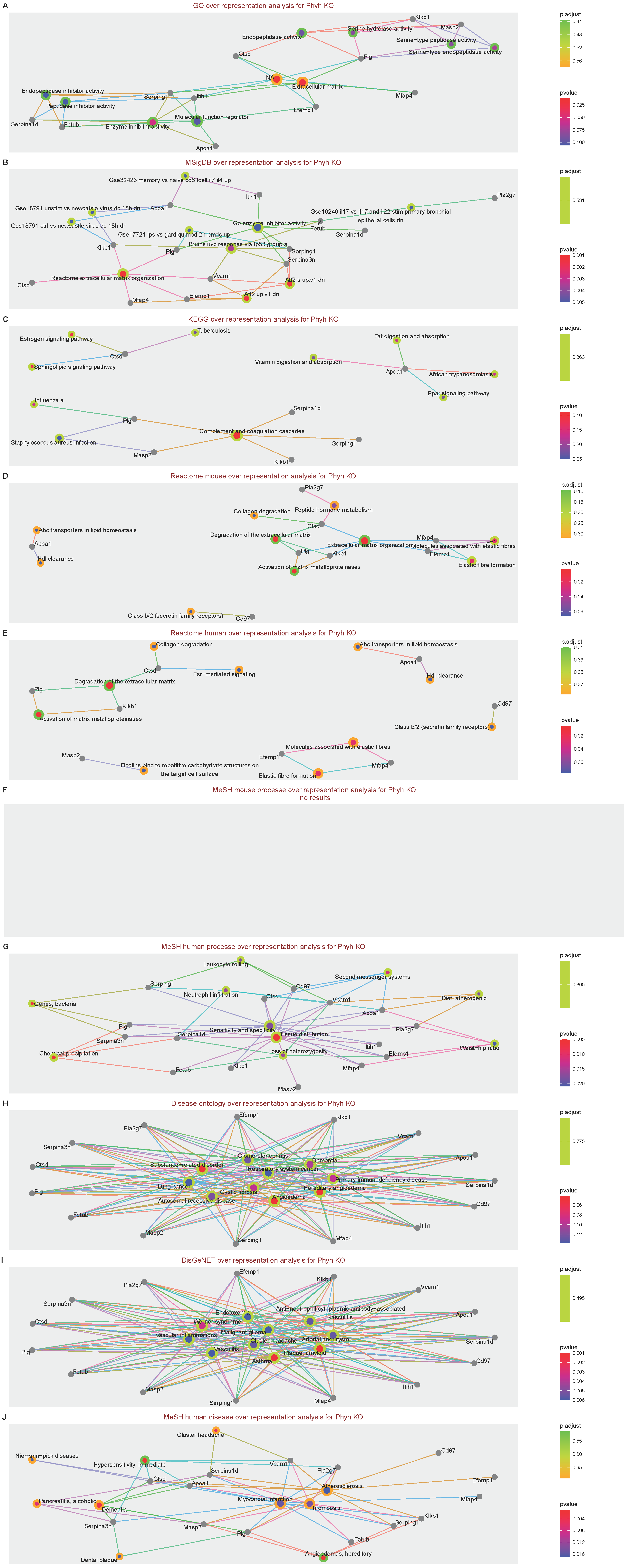

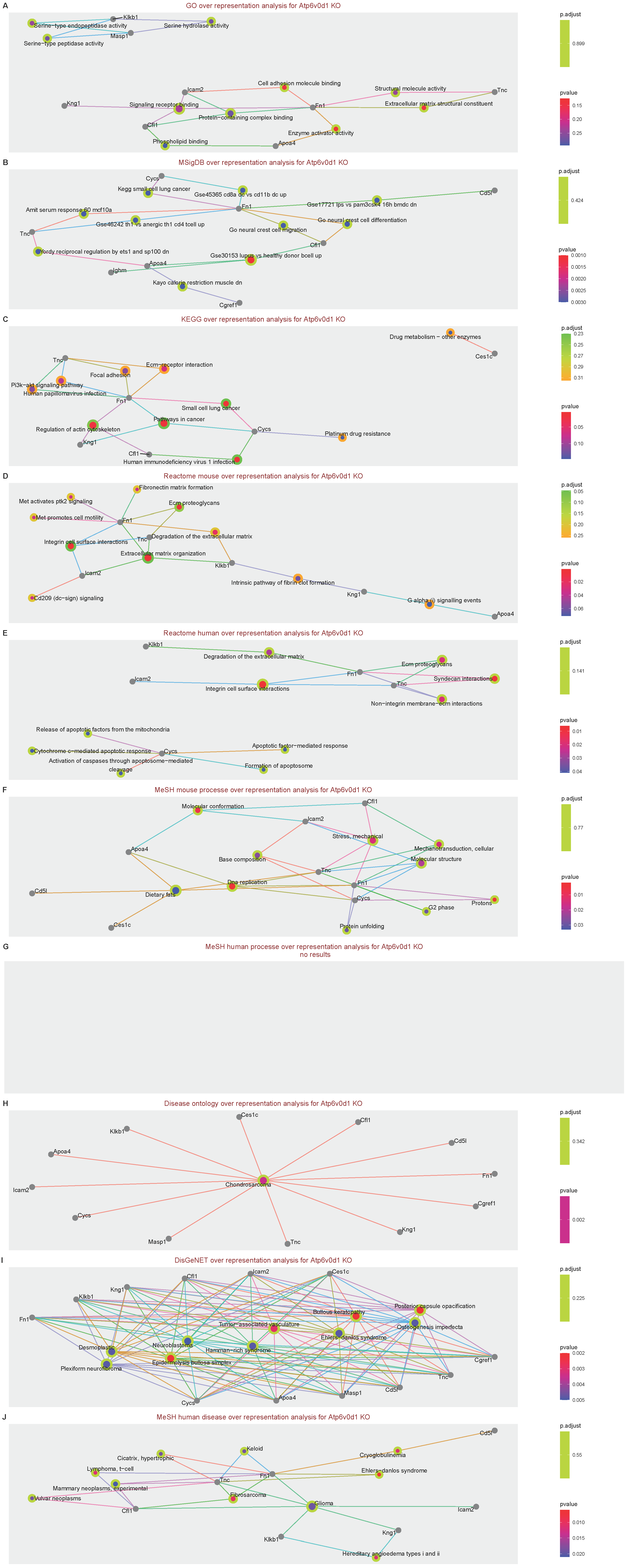

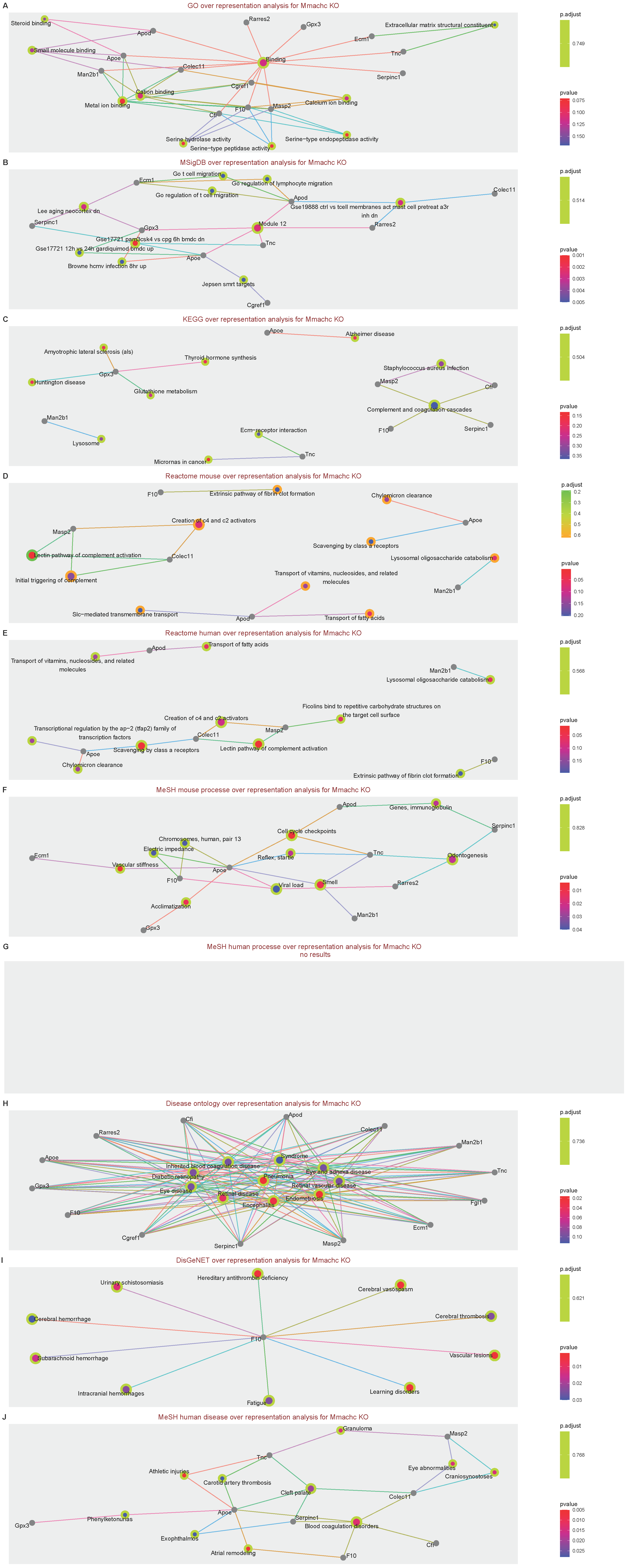

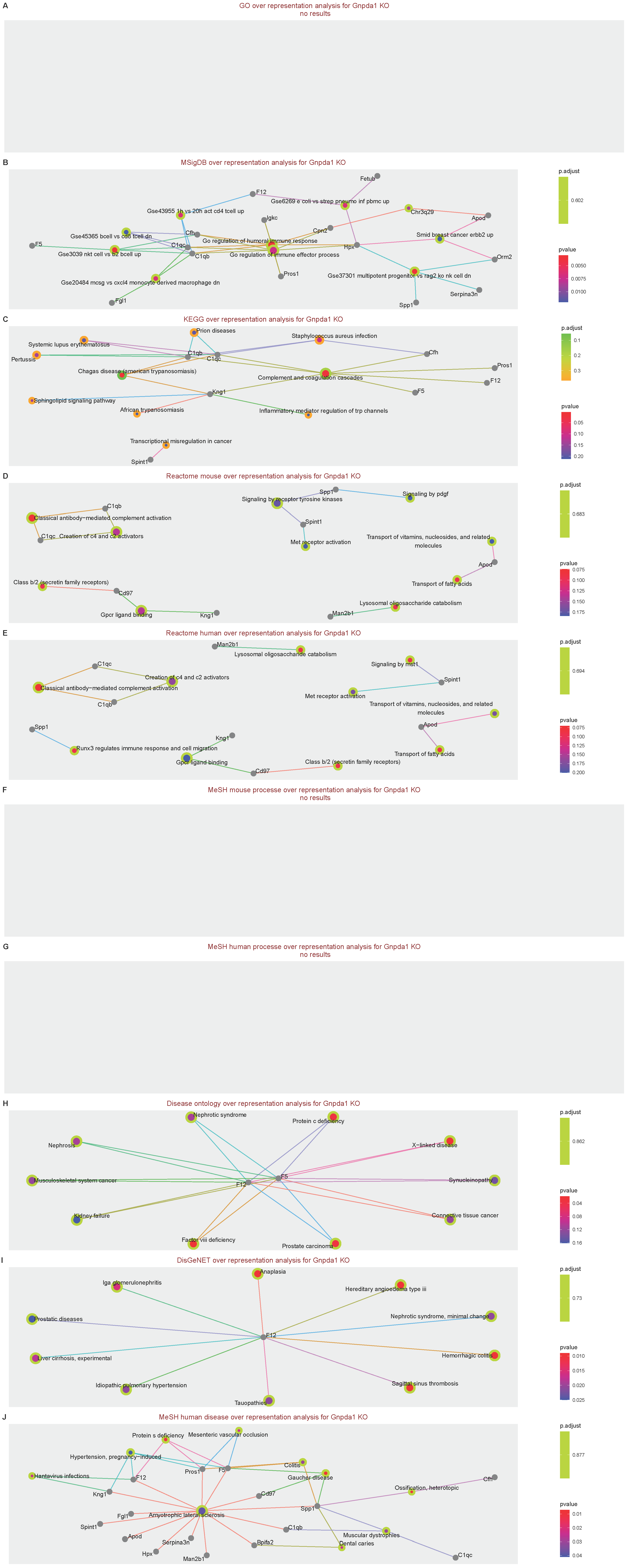

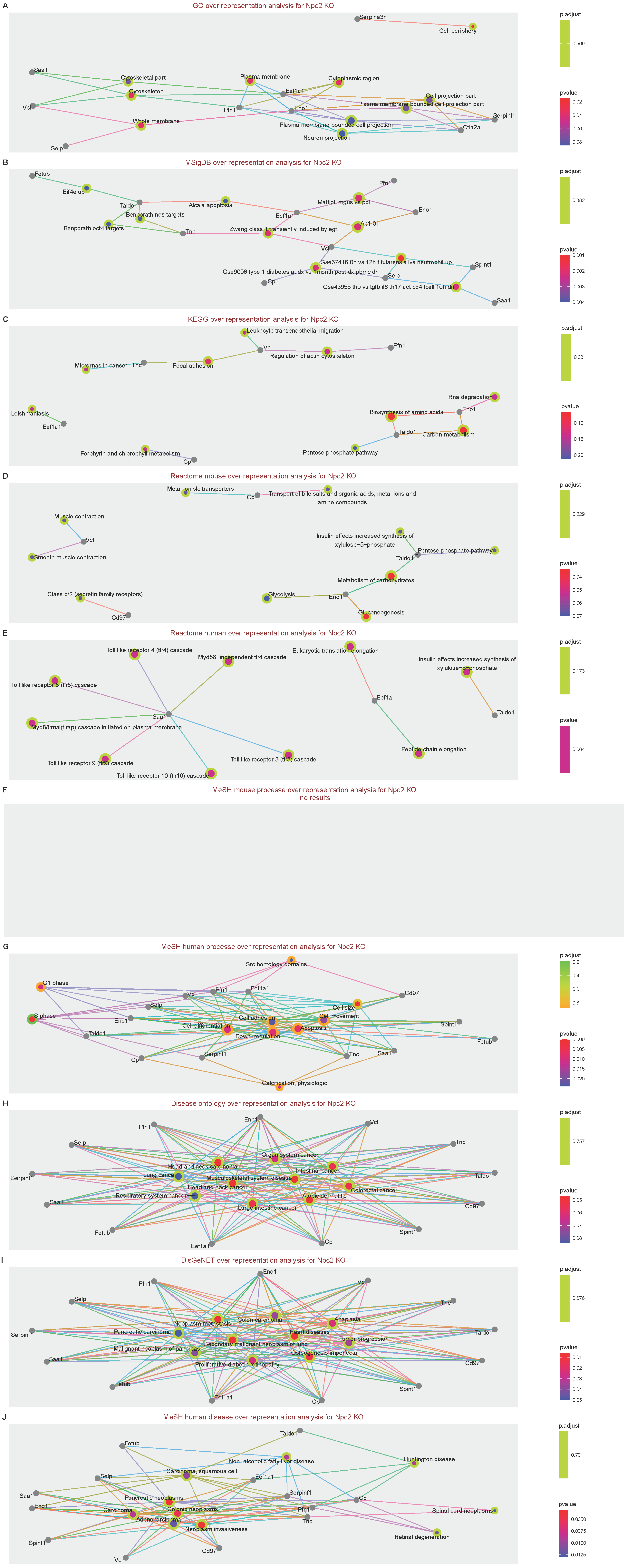

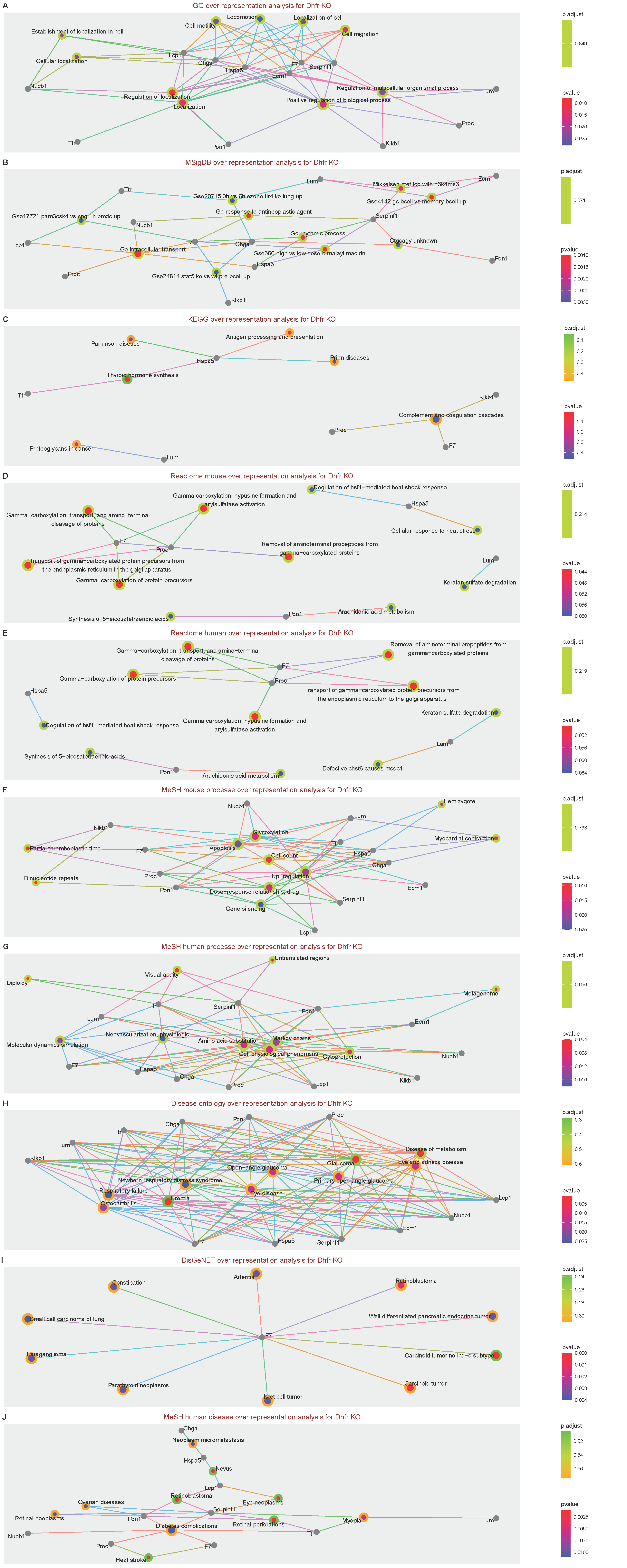

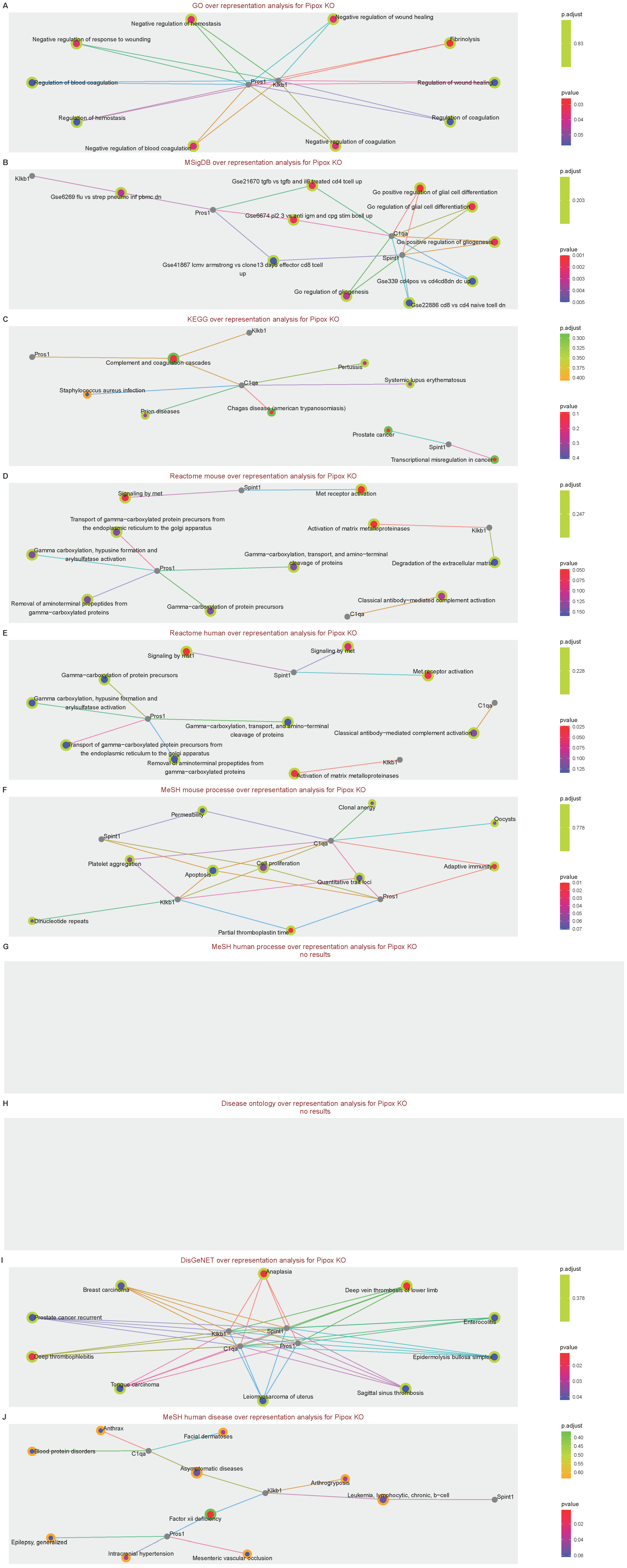

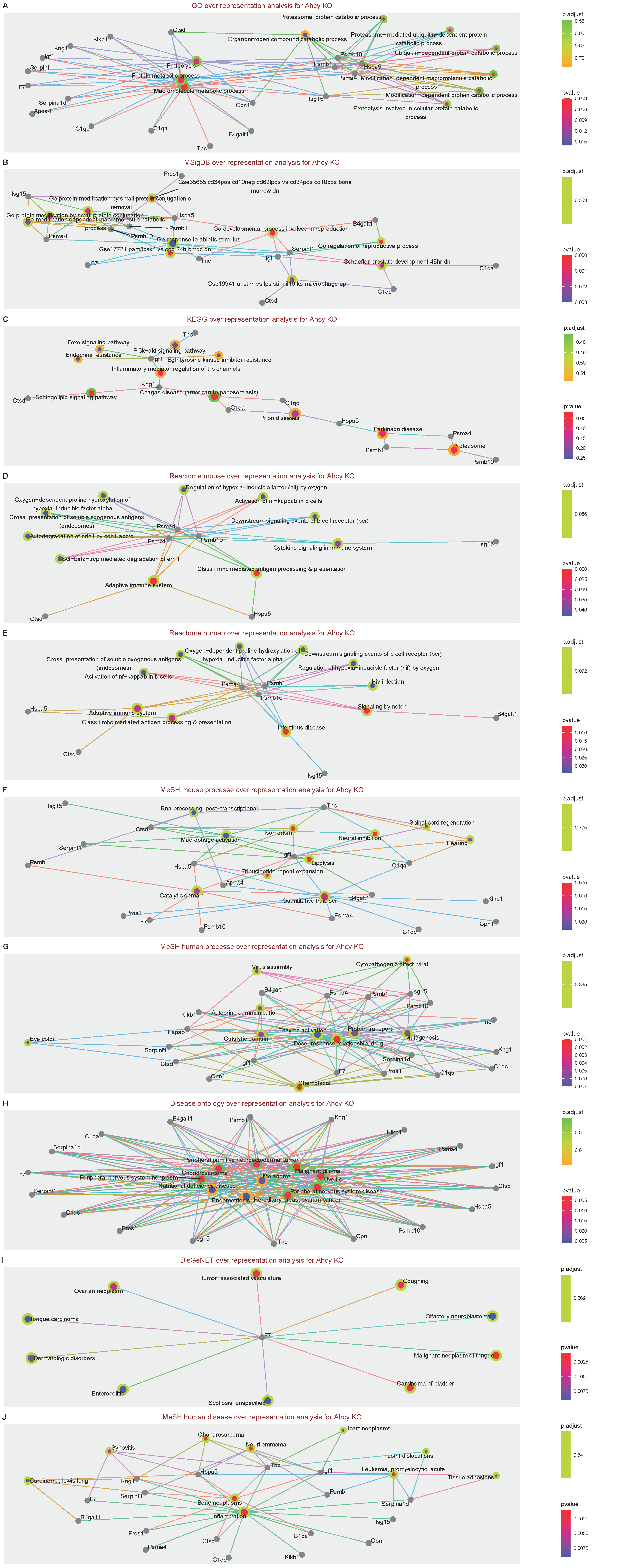

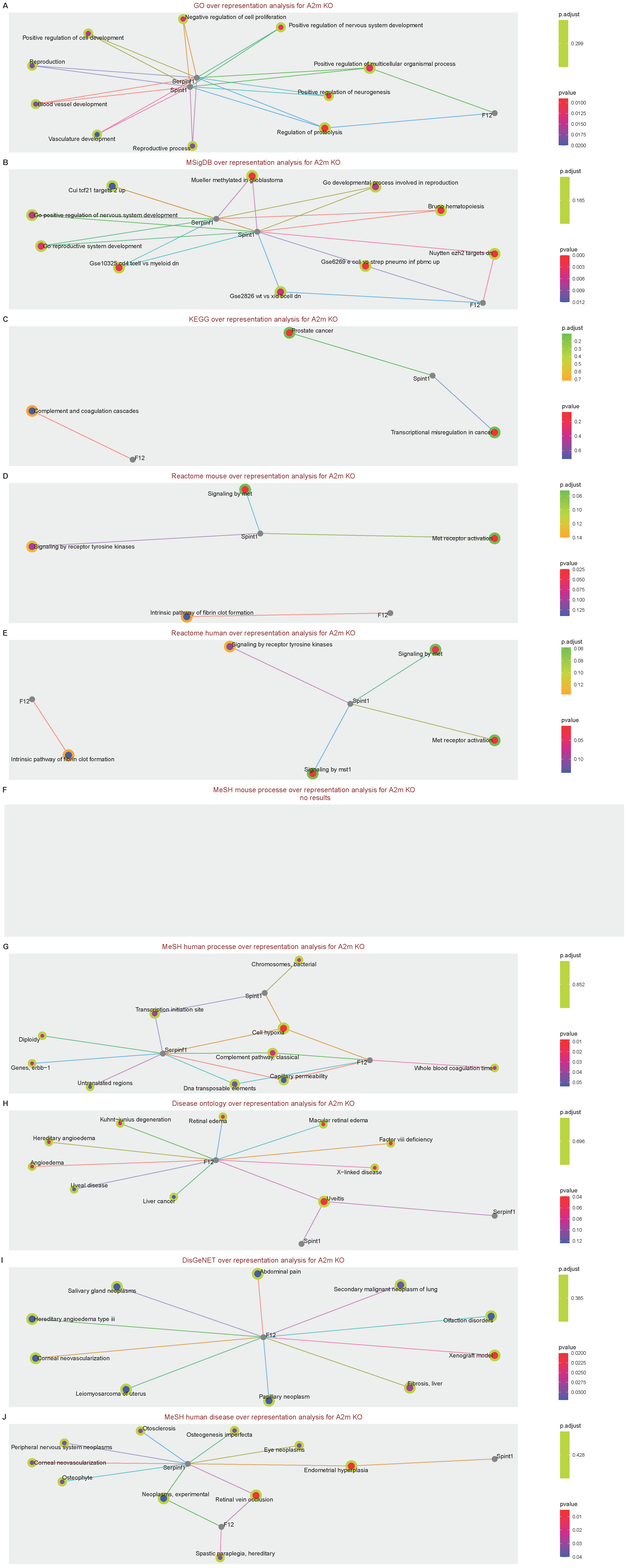

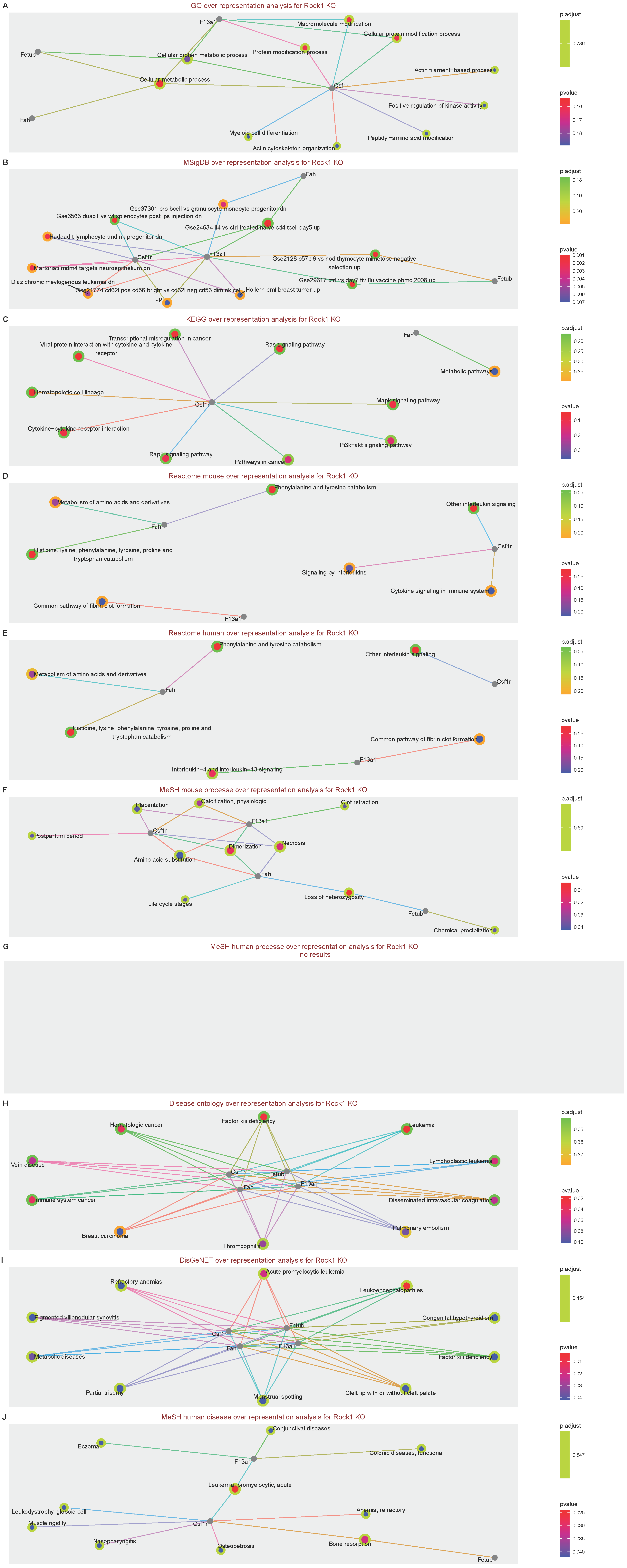

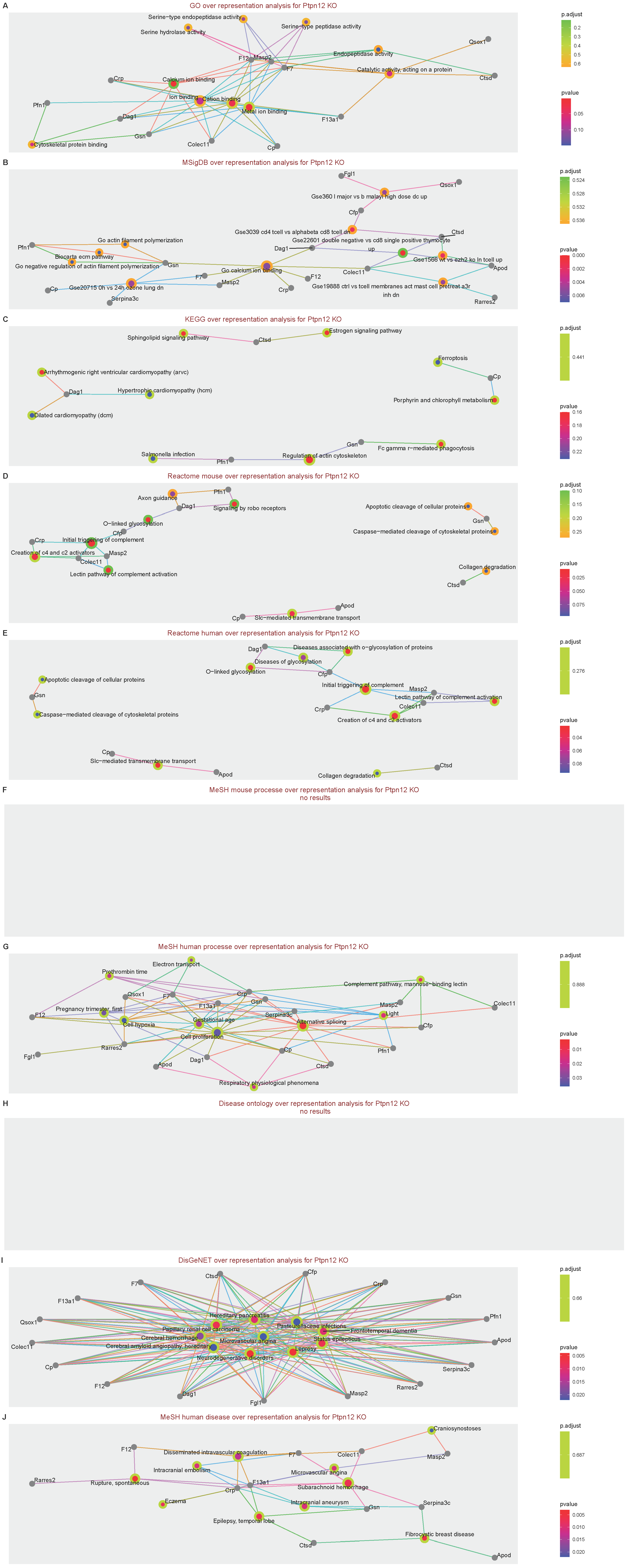

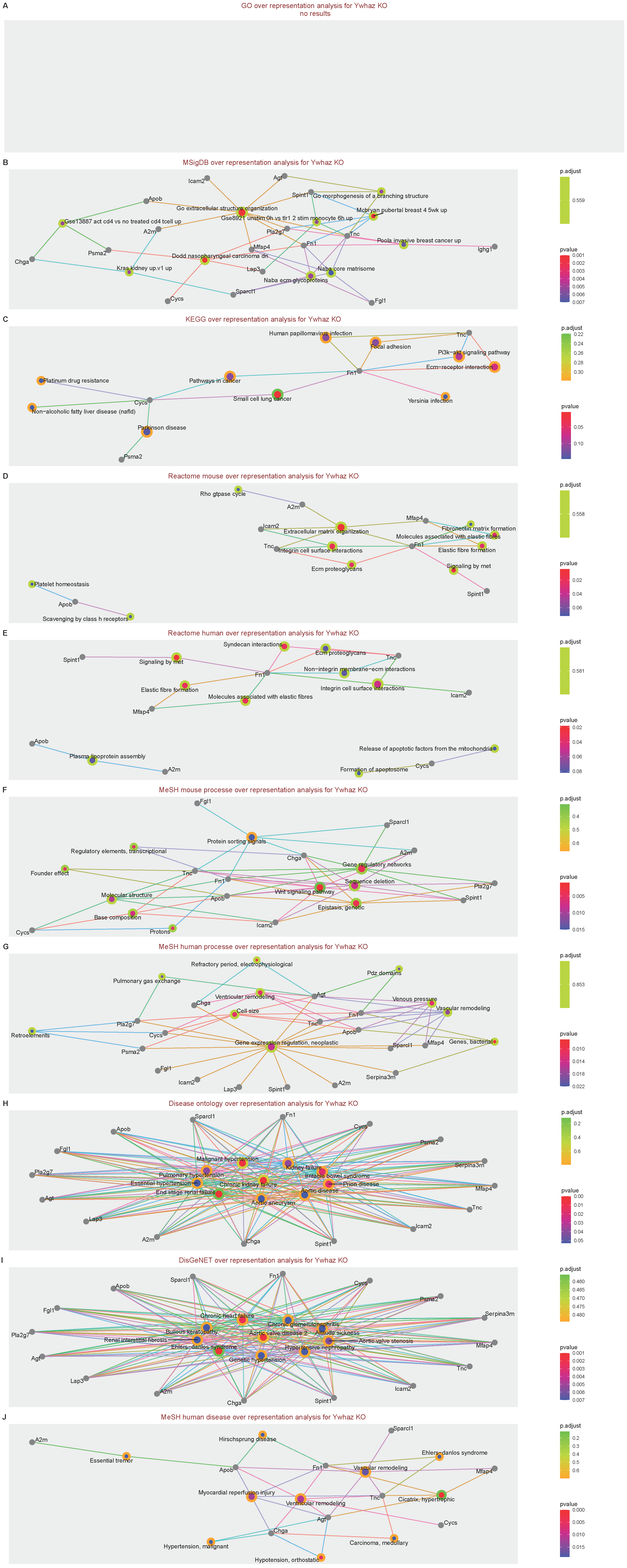

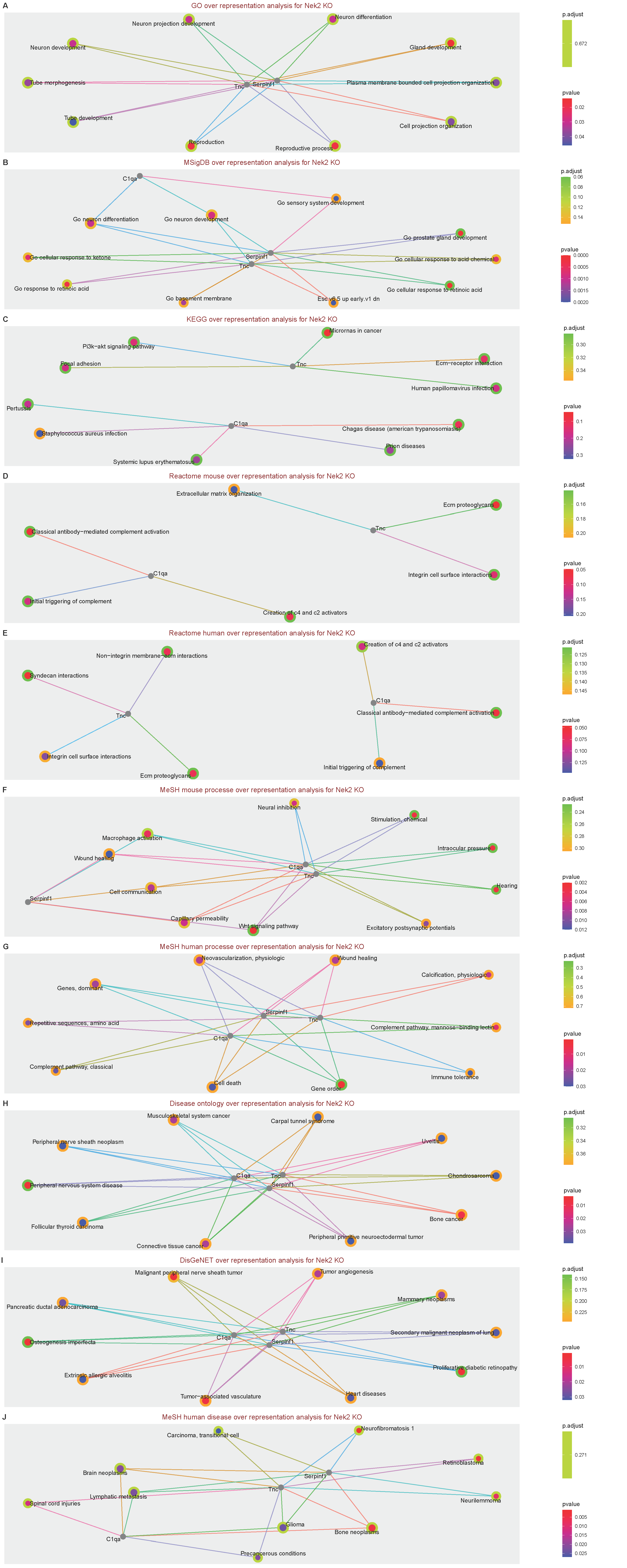

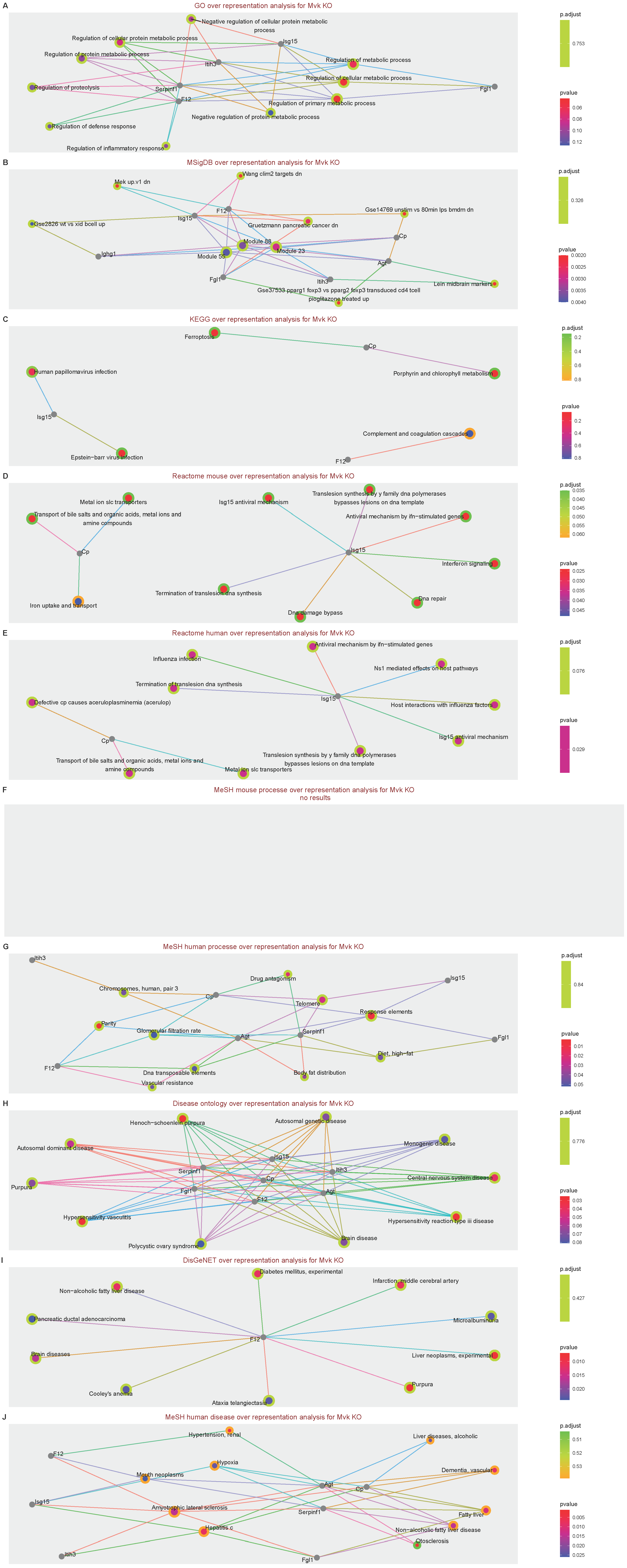


**Supplementary Dataset 1.** Protein concentration measured in individual samples (fmol/μl).

| Sample | Project | Gene symbol | Mouse line name | Zygosity | Gender | Mouse BCode | date collected | Sfn | Hspa5 | Actg1 | Adipoq | Afm | Orm1 | Orm2 | Serpina1a | Serpina1b | Serpina1d | Serpina1e | A1bg | Serpinf2 | Pzp | A2m | Eno1 | Agt | Serpinc1 | Apoa1 | Apoa2 | Apoa4 | Apob | Apoc4 | Apod | Apoe | Apom | B4galt1 | Apoh | Eno3 | Bpgm | Blmh | Bpifa2 | Ca1 | Ca2 | Ces1c | Cpb2 | Cpn1 | Cpn2 | Cpq | Ctsd | Ctss | Ctsz | Cd5l | Cd97 | Cgref1 | Cp | Bche | Chga | Clu | F9 | F5 | F7 |
| --- | --- | --- | --- | --- | --- | --- | --- | --- | --- | --- | --- | --- | --- | --- | --- | --- | --- | --- | --- | --- | --- | --- | --- | --- | --- | --- | --- | --- | --- | --- | --- | --- | --- | --- | --- | --- | --- | --- | --- | --- | --- | --- | --- | --- | --- | --- | --- | --- | --- | --- | --- | --- | --- | --- | --- | --- | --- | --- | --- |
| 448371 | **KOMP2** | **(null)** | **C57BL/6NCrl** | **+/+** | **F** | **448371** | **10/12/2016** | NA | 11.09 | 121.36 | 20.67 | 263.85 | 919.92 | 64.51 | 18972.28 | 6157.36 | 3001.08 | 456.06 | 307.52 | 488.80 | 6436.98 | 19.96 | 2.70 | 130.20 | 2077.69 | 24261.80 | 36238.95 | 2777.81 | 244.73 | 355.02 | 27.52 | 2134.73 | 484.12 | 5.62 | 3201.15 | 0.00 | 0.00 | 12.03 | 23.25 | 2.84 | 56.54 | 4174.87 | 85.19 | 151.46 | 533.02 | 39.46 | 3.75 | 4.75 | 9.40 | 73.22 | 2.68 | 0.00 | 789.75 | 19.32 | 11.99 | 1134.23 | 6.91 | 40.83 | 39.79 |
| 115863 | **NorCOMM2** | **(null)** | **C57BL/6NCrl** | **+/+** | **F** | **115863** | **6/5/2014** | NA | 10.81 | 140.28 | 22.78 | 320.23 | 1319.12 | 56.84 | 13754.24 | 6368.78 | 3063.95 | 125.89 | 887.13 | 443.64 | 7161.66 | 14.43 | 10.15 | 110.77 | 1716.48 | 25071.24 | 39555.75 | 2999.43 | 217.35 | 299.38 | 21.54 | 2431.97 | 500.12 | 7.72 | 2793.88 | 20.63 | 6.04 | 13.57 | 52.81 | 15.80 | 92.01 | 4642.99 | 79.30 | 163.48 | 500.80 | 49.49 | 3.89 | 4.81 | 13.12 | 100.33 | 5.36 | 0.00 | 781.97 | 27.52 | 10.91 | 984.26 | 7.26 | 39.12 | 28.07 |
| 432638 | **KOMP2** | **(null)** | **C57BL/6NCrl** | **+/+** | **F** | **432638** | **9/1/2016** | NA | 4.75 | 64.77 | 15.76 | 216.58 | 720.56 | 35.84 | 13982.22 | 5189.37 | 2271.14 | 121.02 | 313.97 | 428.79 | 5336.38 | 28.54 | 1.64 | 124.50 | 1599.09 | 20054.97 | 28224.35 | 1707.27 | 228.01 | 171.65 | 20.19 | 1287.82 | 374.70 | 3.54 | 2594.21 | 0.00 | 0.00 | 9.20 | 86.00 | 0.00 | 34.93 | 3060.01 | 64.23 | 119.36 | 369.38 | 26.98 | 2.77 | 4.37 | 6.90 | 130.87 | 3.30 | 0.00 | 617.12 | 16.70 | 5.98 | 863.52 | 4.23 | 34.88 | 20.81 |
| 237715 | **NorCOMM2** | **(null)** | **C57BL/6NCrl** | **+/+** | **F** | **237715** | **4/21/2015** | NA | 12.00 | 113.33 | 21.18 | 268.49 | 1319.74 | 78.31 | 17073.45 | 6687.64 | 3090.48 | 174.24 | 947.32 | 396.96 | 6233.34 | 16.71 | 3.49 | 126.36 | 1997.91 | 24424.50 | 34141.28 | 3156.23 | 213.85 | 244.59 | 22.94 | 1933.21 | 511.23 | 7.63 | 2917.00 | 5.82 | 0.00 | 9.92 | 33.81 | 8.42 | 78.54 | 3049.63 | 62.95 | 161.94 | 501.72 | 46.41 | 4.51 | 4.33 | 8.71 | 95.11 | 4.40 | 0.00 | 815.77 | 23.11 | 8.39 | 986.13 | 6.59 | 35.59 | 28.22 |
| 496095 | **K2P2** | **(null)** | **C57BL/6NCrl** | **+/+** | **F** | **496095** | **2/14/2017** | NA | 7.33 | 140.12 | 33.42 | 308.65 | 1302.50 | 71.44 | 17980.05 | 6336.86 | 3570.39 | 75.71 | 785.70 | 519.42 | 7499.04 | 14.84 | 3.42 | 111.54 | 2012.07 | 28862.74 | 39796.61 | 3226.99 | 233.15 | 499.05 | 20.16 | 1822.24 | 538.65 | 6.02 | 2725.69 | 0.00 | 0.00 | 10.45 | 18.23 | 0.00 | 59.77 | 5533.75 | 97.34 | 170.37 | 536.11 | 47.73 | 3.89 | 5.57 | 11.72 | 114.26 | 4.64 | 0.00 | 912.97 | 25.50 | 9.20 | 1126.00 | 5.94 | 41.02 | 32.24 |
| 229694 | **NorCOMM2** | **(null)** | **C57BL/6NCrl** | **+/+** | **F** | **229694** | **3/26/2015** | NA | 6.06 | 117.43 | 23.33 | 301.67 | 858.92 | 50.03 | 18126.69 | 6815.49 | 3061.47 | 372.27 | 272.40 | 406.47 | 6376.80 | 14.70 | 4.46 | 117.53 | 1981.61 | 27248.93 | 34304.16 | 2544.22 | 257.90 | 307.33 | 22.58 | 1958.66 | 437.62 | 3.68 | 2718.07 | 6.59 | 5.70 | 12.36 | 19.77 | 9.22 | 78.00 | 3012.39 | 72.93 | 145.47 | 499.81 | 34.69 | 3.08 | 4.41 | 12.92 | 66.83 | 4.63 | 0.00 | 675.17 | 21.11 | 9.41 | 1045.26 | 6.91 | 40.94 | 24.23 |
| 417794 | **KOMP2** | **(null)** | **C57BL/6NCrl** | **+/+** | **F** | **417794** | **7/21/2016** | NA | 7.00 | 105.87 | 24.46 | 267.53 | 1176.87 | 62.90 | 16413.03 | 6482.70 | 2949.25 | 558.74 | 156.92 | 378.17 | 6476.35 | 15.85 | 2.59 | 134.48 | 2316.51 | 22091.18 | 34708.88 | 2413.60 | 227.06 | 357.71 | 23.53 | 1470.63 | 455.60 | 3.28 | 2841.60 | 0.00 | 0.00 | 9.38 | 19.16 | 0.00 | 47.45 | 4266.05 | 83.97 | 164.75 | 488.88 | 48.74 | 3.26 | 6.15 | 8.54 | 139.68 | 3.82 | 0.00 | 731.85 | 24.30 | 9.73 | 878.48 | 5.53 | 34.44 | 31.53 |
| 220169 | **NorCOMM2** | **(null)** | **C57BL/6NCrl** | **+/+** | **F** | **220169** | **2/12/2015** | NA | 8.86 | 92.49 | 29.12 | 306.94 | 1211.39 | 48.78 | 18782.45 | 7649.68 | 3283.52 | 151.87 | 584.57 | 553.95 | 6833.54 | 28.79 | 2.13 | 140.47 | 2133.84 | 23196.53 | 39534.61 | 3572.49 | 226.67 | 355.76 | 19.77 | 1889.03 | 543.96 | 3.23 | 2393.58 | 3.08 | 2.19 | 10.55 | 36.25 | 4.17 | 65.39 | 4047.53 | 80.13 | 166.14 | 587.30 | 44.26 | 3.65 | 5.81 | 11.45 | 201.31 | 4.13 | 3.49 | 884.76 | 27.91 | 11.61 | 1261.15 | 6.12 | 44.36 | 24.63 |
| 170955 | **NorCOMM2** | **(null)** | **C57BL/6NCrl** | **+/+** | **F** | **170955** | **10/9/2014** | NA | 7.34 | 89.16 | 32.21 | 355.10 | 1194.05 | 39.48 | 12547.39 | 7470.96 | 3494.64 | 133.68 | 1500.54 | 536.17 | 8333.77 | 28.81 | 3.60 | 127.99 | 2003.23 | 22877.44 | 36957.70 | 2573.37 | 250.68 | 397.16 | 26.77 | 3560.22 | 502.24 | 3.09 | 2916.88 | 2.89 | 3.84 | 11.43 | 54.45 | 9.80 | 87.67 | 3719.85 | 79.57 | 168.74 | 573.76 | 56.65 | 4.39 | 7.38 | 15.09 | 79.91 | 3.69 | 2.84 | 931.17 | 33.61 | 6.93 | 1032.52 | 9.50 | 30.65 | 26.86 |
| 319739 | **NorCOMM2** | **(null)** | **C57BL/6NCrl** | **+/+** | **F** | **319739** | **9/17/2015** | NA | 6.25 | 100.35 | 23.37 | 349.90 | 1014.96 | 49.54 | 15270.03 | 7135.70 | 3089.40 | 240.75 | 1948.70 | 432.12 | 7496.12 | 23.68 | 1.99 | 137.12 | 2197.41 | 26529.18 | 37329.64 | 3668.41 | 342.68 | 457.28 | 19.91 | 2255.10 | 523.57 | 3.92 | 3393.52 | 3.37 | 1.30 | 9.68 | 23.58 | 3.43 | 50.08 | 3938.19 | 79.43 | 175.11 | 532.94 | 47.57 | 5.06 | 6.24 | 13.83 | 82.84 | 4.21 | 4.82 | 861.56 | 28.88 | 11.13 | 1057.79 | 9.38 | 31.57 | 27.30 |
| 198415 | **NorCOMM2** | **(null)** | **C57BL/6NCrl** | **+/+** | **F** | **198415** | **12/23/2014** | NA | 9.17 | 86.04 | 21.14 | 303.99 | 1380.95 | 62.99 | 14864.59 | 6682.68 | 3126.85 | 231.45 | 272.98 | 461.48 | 7743.52 | 29.30 | 1.48 | 124.49 | 1966.75 | 20123.49 | 36197.06 | 3295.21 | 280.99 | 450.89 | 23.45 | 2157.80 | 482.45 | 2.44 | 2596.90 | 0.00 | 0.00 | 12.24 | 31.26 | 3.02 | 55.44 | 3023.83 | 75.32 | 141.55 | 474.07 | 40.19 | 3.66 | 6.78 | 10.37 | 63.72 | 3.36 | 2.82 | 746.36 | 19.61 | 11.06 | 1249.87 | 10.87 | 32.93 | 36.07 |
| 296903 | **NorCOMM2** | **(null)** | **C57BL/6NCrl** | **+/+** | **F** | **296903** | **7/21/2015** | NA | 8.29 | 98.72 | 34.12 | 326.58 | 547.60 | 43.46 | 16145.12 | 6868.79 | 3235.74 | 425.60 | 482.10 | 359.99 | 7916.98 | 18.70 | 2.33 | 107.49 | 1956.79 | 25552.42 | 21511.06 | 3252.69 | 180.51 | 301.60 | 11.03 | 1477.67 | 342.38 | 4.68 | 2421.54 | 3.96 | 2.68 | 17.43 | 6.89 | 6.66 | 63.71 | 4256.00 | 84.25 | 149.48 | 485.85 | 46.86 | 3.50 | 9.51 | 7.20 | 129.72 | 3.04 | 4.50 | 693.20 | 21.73 | 9.98 | 1142.65 | 6.80 | 35.45 | 20.52 |
| 38540 | **NorCOMM2** | **(null)** | **C57BL/6NCrl** | **+/+** | **F** | **38540** | **12/11/2013** | NA | 0.00 | 93.01 | 22.46 | 306.67 | 1298.43 | 96.99 | 12890.61 | 5289.30 | 3019.89 | 60.28 | 826.51 | 426.84 | 7112.66 | 16.71 | 3.55 | 101.67 | 2052.03 | 21621.61 | 37271.43 | 2651.52 | 205.39 | 344.73 | 26.79 | 2046.98 | 478.70 | 3.81 | 2447.95 | 2.62 | 4.00 | 10.66 | 24.52 | 7.79 | 90.96 | 3927.68 | 74.27 | 147.11 | 454.42 | 44.50 | 4.11 | 6.36 | 10.75 | 248.96 | 3.48 | 3.36 | 794.53 | 26.35 | 8.56 | 956.27 | 8.46 | 31.31 | 28.40 |
| 349028 | **NorCOMM2** | **(null)** | **C57BL/6NCrl** | **+/+** | **F** | **349028** | **11/5/2015** | NA | 3.65 | 93.67 | 23.80 | 260.23 | 916.81 | 40.44 | 14192.00 | 9166.50 | 3403.10 | 138.39 | 432.96 | 412.74 | 7794.40 | 24.57 | 3.35 | 105.55 | 1371.50 | 21135.00 | 39828.00 | 3191.20 | 378.37 | 285.59 | 24.23 | 2330.10 | 404.85 | 5.71 | 2433.60 | 2.91 | 3.68 | 11.15 | 37.07 | 11.40 | 80.87 | 3462.00 | 82.43 | 158.73 | 500.81 | 37.86 | 3.71 | 6.38 | 8.04 | 100.61 | 4.46 | 3.52 | 922.40 | 26.13 | 12.49 | 1094.50 | 7.38 | 33.49 | 24.97 |
| 38537 | **NorCOMM2** | **(null)** | **C57BL/6NCrl** | **+/+** | **F** | **38537** | **12/11/2013** | NA | 11.76 | 81.01 | 27.84 | 326.90 | 1709.80 | 153.15 | 13281.00 | 6247.70 | 3480.60 | 187.24 | 388.72 | 469.74 | 8354.30 | 13.93 | 1.76 | 117.47 | 2125.40 | 22449.00 | 38234.00 | 2730.30 | 320.30 | 326.70 | 30.99 | 2636.90 | 551.89 | 6.42 | 2464.40 | 3.35 | 2.44 | 12.71 | 15.94 | 6.30 | 56.20 | 3509.00 | 89.83 | 168.67 | 483.66 | 39.53 | 3.85 | 6.16 | 9.43 | 89.66 | 4.17 | 3.39 | 773.33 | 22.76 | 10.37 | 1043.60 | 4.90 | 32.95 | 28.13 |
| 55856 | **NorCOMM2** | **(null)** | **C57BL/6NCrl** | **+/+** | **F** | **55856** | **1/22/2014** | NA | 5.32 | 100.10 | 32.75 | 365.21 | 1489.40 | 65.80 | 11098.00 | 5854.80 | 4129.90 | 46.08 | 1091.70 | 511.62 | 9285.00 | 10.99 | 4.05 | 120.87 | 1808.90 | 23460.00 | 40527.00 | 2356.80 | 302.70 | 309.24 | 18.83 | 1918.80 | 488.57 | 3.83 | 2650.70 | 4.33 | 4.90 | 11.83 | 50.58 | 9.32 | 89.43 | 3777.90 | 86.87 | 184.16 | 567.06 | 46.63 | 4.04 | 8.67 | 11.16 | 63.89 | 3.69 | 0.00 | 910.01 | 23.60 | 9.18 | 1102.50 | 8.34 | 40.95 | 26.68 |
| 144712 | **NorCOMM2** | **(null)** | **C57BL/6NCrl** | **+/+** | **F** | **144712** | **8/7/2014** | NA | 5.85 | 66.50 | 29.40 | 317.98 | 1007.00 | 45.89 | 15461.00 | 5923.00 | 3518.00 | 111.95 | 620.98 | 507.21 | 8578.40 | 13.76 | 2.24 | 88.25 | 1794.90 | 19908.00 | 34309.00 | 2841.30 | 159.75 | 363.80 | 24.37 | 1571.40 | 490.21 | 4.49 | 2755.90 | 2.78 | 2.62 | 8.88 | 18.53 | 5.19 | 92.35 | 3090.60 | 79.74 | 171.43 | 531.74 | 43.23 | 3.77 | 6.90 | 8.19 | 137.81 | 4.14 | 2.39 | 893.97 | 32.73 | 8.65 | 1046.50 | 7.02 | 35.04 | 27.90 |
| 257642 | **NorCOMM2** | **(null)** | **C57BL/6NCrl** | **+/+** | **F** | **257642** | **5/21/2015** | NA | 6.12 | 137.44 | 37.16 | 290.37 | 1548.10 | 88.28 | 14748.00 | 6779.00 | 4095.40 | 29.76 | 639.24 | 571.20 | 7401.80 | 8.11 | 2.61 | 107.11 | 1843.40 | 19869.00 | 39419.00 | 3135.20 | 204.88 | 413.81 | 19.49 | 1544.80 | 448.09 | 3.31 | 2104.40 | 2.69 | 2.86 | 15.21 | 18.73 | 4.77 | 75.75 | 3568.40 | 90.98 | 153.32 | 511.77 | 39.51 | 4.20 | 7.16 | 8.91 | 106.67 | 4.83 | 3.14 | 892.88 | 22.55 | 12.56 | 934.29 | 10.32 | 35.56 | 30.02 |
| 209240 | **NorCOMM2** | **(null)** | **C57BL/6NCrl** | **+/+** | **F** | **209240** | **1/13/2015** | NA | 7.05 | 122.23 | 19.93 | 306.00 | 1176.50 | 75.20 | 9753.80 | 5952.40 | 3507.70 | 126.28 | 192.09 | 528.65 | 7799.80 | 13.41 | 2.91 | 104.16 | 1602.50 | 24183.00 | 28746.00 | 2141.70 | 333.73 | 345.84 | 27.81 | 1656.00 | 504.10 | 5.04 | 2285.00 | 4.42 | 0.00 | 18.85 | 28.00 | 3.43 | 40.25 | 3192.30 | 77.34 | 142.18 | 398.67 | 44.80 | 3.32 | 9.16 | 11.04 | 63.14 | 3.96 | 2.66 | 830.51 | 22.79 | 14.51 | 923.86 | 8.43 | 32.89 | 22.75 |
| 410682 | **KOMP2** | **(null)** | **C57BL/6NCrl** | **+/+** | **M** | **410682** | **6/16/2016** | NA | 13.40 | 119.88 | 8.64 | 160.33 | 1762.88 | 100.89 | 31458.13 | 8688.12 | 3669.49 | 5950.63 | 0.00 | 504.61 | 7406.95 | 0.00 | 3.54 | 165.60 | 2267.62 | 28059.37 | 47125.15 | 3933.12 | 311.63 | 666.51 | 29.69 | 2398.95 | 524.04 | 4.14 | 3090.14 | 7.54 | 0.00 | 13.01 | 27.32 | 3.31 | 47.71 | 2715.84 | 150.77 | 156.29 | 670.83 | 24.30 | 3.28 | 5.67 | 10.35 | 100.47 | 3.62 | 0.00 | 822.04 | 16.65 | 9.32 | 1982.56 | 8.61 | 46.58 | 28.61 |
| 307708 | **NorCOMM2** | **(null)** | **C57BL/6NCrl** | **+/+** | **M** | **307708** | **8/18/2015** | NA | 9.75 | 80.71 | 11.85 | 168.35 | 954.75 | 11.88 | 33677.01 | 8324.00 | 3537.57 | 4331.64 | 0.00 | 491.07 | 7328.12 | 7.90 | 2.73 | 160.01 | 2682.20 | 40592.97 | 57058.76 | 2844.77 | 257.88 | 453.54 | 14.98 | 2103.49 | 538.40 | 5.12 | 3259.95 | 0.00 | 0.00 | 11.23 | 18.30 | 3.61 | 49.04 | 3750.19 | 114.04 | 171.89 | 501.54 | 34.85 | 2.79 | 5.12 | 8.38 | 84.16 | 2.99 | 0.00 | 808.81 | 14.76 | 9.92 | 2096.69 | 6.67 | 40.68 | 30.55 |
| 441143 | **KOMP2** | **(null)** | **C57BL/6NCrl** | **+/+** | **M** | **441143** | **9/22/2016** | NA | 9.62 | 59.33 | 9.72 | 206.61 | 1486.24 | 33.48 | 31272.96 | 7249.82 | 3401.95 | 4215.00 | 0.00 | 480.31 | 7910.97 | 0.00 | 2.44 | 143.91 | 2522.31 | 33475.81 | 54680.73 | 4368.87 | 300.84 | 449.86 | 19.98 | 2631.50 | 475.80 | 3.77 | 3052.55 | 0.00 | 4.48 | 8.08 | 14.38 | 5.81 | 59.63 | 2445.75 | 114.35 | 165.92 | 534.87 | 27.79 | 3.19 | 5.80 | 7.91 | 291.75 | 2.93 | 0.00 | 835.84 | 15.48 | 7.67 | 1665.42 | 7.36 | 44.57 | 24.76 |
| 237609 | **NorCOMM2** | **(null)** | **C57BL/6NCrl** | **+/+** | **M** | **237609** | **4/23/2015** | NA | 3.14 | 65.89 | 12.63 | 172.35 | 1351.94 | 13.76 | 38078.36 | 9514.43 | 3200.23 | 6045.95 | 0.00 | 453.84 | 7166.57 | 0.00 | 1.95 | 131.19 | 2134.89 | 38483.99 | 46635.53 | 4010.50 | 226.59 | 460.35 | 19.28 | 1899.97 | 653.51 | 3.29 | 2763.70 | 0.00 | 0.00 | 11.99 | 9.52 | 7.66 | 84.54 | 2461.22 | 135.15 | 163.04 | 597.79 | 25.44 | 3.94 | 4.14 | 9.07 | 69.15 | 3.13 | 0.00 | 774.04 | 12.65 | 9.60 | 1919.23 | 8.57 | 30.65 | 24.11 |
| 510072 | **K2P2** | **(null)** | **C57BL/6NCrl** | **+/+** | **M** | **510072** | **4/19/2017** | NA | 5.15 | 88.44 | 12.52 | 293.86 | 1353.60 | 14.87 | 24978.12 | 8551.86 | 3637.91 | 4668.67 | 0.00 | 472.48 | 8833.84 | 7.30 | 2.03 | 151.24 | 2374.99 | 42421.02 | 56020.28 | 2647.29 | 300.06 | 405.65 | 17.72 | 3176.02 | 531.95 | 5.95 | 3285.64 | 0.00 | 0.00 | 10.06 | 39.01 | 0.00 | 58.89 | 2936.47 | 122.63 | 187.85 | 507.27 | 28.88 | 3.62 | 5.39 | 10.23 | 87.12 | 4.67 | 2.51 | 919.47 | 17.76 | 7.55 | 2072.93 | 10.33 | 51.85 | 23.22 |
| 198312 | **NorCOMM2** | **(null)** | **C57BL/6NCrl** | **+/+** | **M** | **198312** | **12/23/2014** | NA | 11.02 | 100.75 | 11.92 | 270.63 | 1350.29 | 29.22 | 24583.16 | 8643.15 | 3505.52 | 2804.94 | 0.00 | 459.72 | 9085.67 | 0.00 | 4.72 | 141.80 | 1680.47 | 30380.56 | 59241.33 | 3702.84 | 254.97 | 493.86 | 15.32 | 1848.54 | 460.29 | 2.77 | 2634.63 | 6.79 | 4.80 | 10.83 | 11.76 | 13.76 | 101.94 | 2676.97 | 110.34 | 182.84 | 530.44 | 36.22 | 2.92 | 4.62 | 9.58 | 66.97 | 3.70 | 0.00 | 796.72 | 17.87 | 7.56 | 1568.68 | 9.77 | 41.82 | 31.39 |
| 483043 | **K2P2** | **(null)** | **C57BL/6NCrl** | **+/+** | **M** | **483043** | **12/29/2016** | NA | 3.54 | 79.00 | 10.77 | 184.19 | 931.10 | 12.92 | 22613.68 | 6506.98 | 2779.58 | 2756.48 | 0.00 | 432.07 | 7258.85 | 0.00 | 2.11 | 92.91 | 1982.46 | 27558.15 | 46702.65 | 2535.65 | 215.27 | 499.59 | 19.50 | 2797.89 | 376.47 | 3.71 | 2968.90 | 0.00 | 0.00 | 7.30 | 10.87 | 0.00 | 42.19 | 2047.34 | 105.53 | 150.15 | 466.74 | 23.45 | 2.30 | 5.45 | 7.09 | 82.09 | 2.46 | 2.28 | 644.84 | 11.41 | 5.26 | 1162.48 | 7.85 | 32.54 | 16.74 |
| 34418 | **NorCOMM2** | **(null)** | **C57BL/6NCrl** | **+/+** | **M** | **34418** | **11/20/2013** | NA | 12.02 | 137.41 | 8.07 | 165.39 | 2107.03 | 88.14 | 27539.44 | 8664.16 | 3097.70 | 6199.26 | 0.00 | 429.04 | 6914.40 | 0.00 | 4.77 | 117.29 | 1586.22 | 33698.52 | 34283.13 | 2379.33 | 332.30 | 315.75 | 17.77 | 2217.37 | 583.70 | 6.88 | 2507.16 | 3.83 | 10.13 | 14.87 | 12.42 | 21.07 | 155.66 | 2261.16 | 128.48 | 156.74 | 558.30 | 23.98 | 4.42 | 7.25 | 8.15 | 68.62 | 3.53 | 3.82 | 909.94 | 16.98 | 7.95 | 1909.29 | 10.49 | 40.87 | 20.97 |
| 257539 | **NorCOMM2** | **(null)** | **C57BL/6NCrl** | **+/+** | **M** | **257539** | **5/20/2015** | NA | 9.00 | 88.03 | 13.26 | 232.08 | 1082.74 | 26.23 | 27702.01 | 8562.30 | 3673.22 | 3489.30 | 0.00 | 548.80 | 8183.79 | 6.62 | 1.80 | 121.03 | 2307.76 | 34816.98 | 51220.47 | 3049.57 | 224.98 | 597.46 | 13.27 | 2668.63 | 541.03 | 5.15 | 2681.90 | 0.00 | 0.00 | 13.31 | 12.50 | 2.45 | 72.92 | 3204.50 | 105.20 | 177.25 | 536.74 | 34.64 | 3.35 | 7.84 | 4.92 | 268.42 | 2.80 | 5.59 | 793.71 | 17.93 | 8.52 | 2025.17 | 12.19 | 32.96 | 28.01 |
| 348925 | **NorCOMM2** | **(null)** | **C57BL/6NCrl** | **+/+** | **M** | **348925** | **11/3/2015** | NA | 3.02 | 144.25 | 14.12 | 203.53 | 1212.80 | 25.31 | 24958.30 | 6178.43 | 2954.83 | 6366.94 | 0.00 | 397.17 | 7891.61 | 0.00 | 3.33 | 91.97 | 2250.69 | 25258.21 | 28077.63 | 3373.46 | 290.02 | 395.80 | 27.89 | 1895.63 | 436.41 | 5.93 | 2019.73 | 2.81 | 0.00 | 14.63 | 7.37 | 0.00 | 56.81 | 2835.32 | 106.89 | 165.63 | 502.55 | 33.68 | 4.04 | 7.35 | 8.06 | 113.65 | 4.00 | 5.78 | 865.02 | 17.25 | 10.76 | 1552.10 | 7.91 | 36.90 | 22.32 |
| 91833 | **NorCOMM2** | **(null)** | **C57BL/6NCrl** | **+/+** | **M** | **91833** | **4/29/2014** | NA | 12.97 | 124.09 | 8.65 | 226.21 | 2285.61 | 321.63 | 26574.02 | 9612.12 | 3602.75 | 5087.33 | 0.00 | 508.58 | 9005.72 | 7.37 | 2.46 | 128.25 | 2047.78 | 33396.74 | 44791.03 | 4361.41 | 309.28 | 524.16 | 15.14 | 2152.93 | 564.32 | 6.04 | 2877.00 | 0.00 | 0.00 | 15.86 | 24.57 | 0.00 | 53.40 | 3144.63 | 171.35 | 177.15 | 586.83 | 29.91 | 4.17 | 7.61 | 8.51 | 68.52 | 3.72 | 6.39 | 1005.97 | 16.10 | 12.96 | 2286.52 | 10.42 | 38.48 | 27.78 |
| 365958 | **NorCOMM2** | **(null)** | **C57BL/6NCrl** | **+/+** | **M** | **365958** | **12/17/2015** | NA | 9.98 | 122.62 | 9.94 | 197.87 | 1565.76 | 48.27 | 25431.98 | 8789.21 | 3361.52 | 5929.41 | 0.00 | 563.87 | 8646.88 | 5.68 | 2.95 | 109.82 | 2086.61 | 36417.86 | 55367.00 | 2971.52 | 219.21 | 514.44 | 26.83 | 2324.94 | 674.75 | 4.86 | 3129.76 | 2.76 | 2.42 | 11.81 | 16.79 | 5.03 | 51.22 | 2765.58 | 146.26 | 182.47 | 666.33 | 29.69 | 3.96 | 8.15 | 7.93 | 133.30 | 4.04 | 4.65 | 895.15 | 12.49 | 10.58 | 2404.71 | 11.82 | 39.70 | 27.75 |
| 209136 | **NorCOMM2** | **(null)** | **C57BL/6NCrl** | **+/+** | **M** | **209136** | **1/13/2015** | NA | 9.15 | 103.54 | 10.72 | 237.59 | 1435.70 | 22.75 | 20461.39 | 8210.13 | 3920.15 | 3706.17 | 0.00 | 534.12 | 8507.51 | 7.03 | 2.65 | 109.64 | 2550.29 | 38522.80 | 51651.60 | 3491.14 | 264.96 | 451.25 | 15.95 | 2402.67 | 490.18 | 3.61 | 2519.80 | 4.46 | 0.00 | 11.35 | 23.16 | 3.38 | 64.67 | 2689.79 | 114.39 | 170.41 | 540.14 | 32.05 | 3.16 | 6.63 | 8.22 | 187.41 | 2.62 | 2.46 | 947.54 | 17.40 | 11.58 | 2053.86 | 8.47 | 39.06 | 20.97 |
| 229586 | **NorCOMM2** | **(null)** | **C57BL/6NCrl** | **+/+** | **M** | **229586** | **3/24/2015** | NA | 11.55 | 149.62 | 14.37 | 228.57 | 1135.20 | 41.92 | 23759.00 | 7603.90 | 3610.80 | 4374.20 | 0.00 | 461.65 | 9576.80 | 6.38 | 4.18 | 126.82 | 1942.40 | 36458.00 | 48418.00 | 3427.20 | 222.33 | 561.33 | 18.58 | 2614.30 | 572.19 | 6.82 | 2463.00 | 0.00 | 0.00 | 12.32 | 16.05 | 6.15 | 75.87 | 2510.40 | 136.96 | 186.67 | 503.60 | 27.18 | 2.89 | 8.08 | 6.86 | 65.96 | 3.93 | 3.28 | 762.50 | 18.80 | 8.77 | 1761.50 | 9.99 | 37.65 | 27.22 |
| 187594 | **NorCOMM2** | **(null)** | **C57BL/6NCrl** | **+/+** | **M** | **187594** | **11/25/2014** | NA | 11.32 | 83.20 | 16.61 | 212.10 | 904.71 | 14.79 | 22400.00 | 8910.40 | 3894.30 | 2392.50 | 0.00 | 414.03 | 9479.40 | 6.39 | 1.86 | 102.75 | 1987.40 | 33238.00 | 46769.00 | 3537.20 | 192.10 | 739.12 | 18.28 | 1354.00 | 441.58 | 4.55 | 2878.50 | 8.46 | 0.00 | 9.99 | 28.96 | 4.68 | 56.90 | 2119.40 | 106.00 | 179.40 | 555.29 | 29.37 | 3.06 | 6.85 | 8.19 | 55.34 | 2.43 | 2.16 | 810.64 | 16.54 | 9.85 | 1399.20 | 11.35 | 35.17 | 25.78 |
| 281969 | **NorCOMM2** | **(null)** | **C57BL/6NCrl** | **+/+** | **M** | **281969** | **6/23/2015** | NA | 13.82 | 89.29 | 12.36 | 209.08 | 1355.30 | 21.64 | 33808.00 | 8783.50 | 4000.80 | 4324.80 | 0.00 | 616.29 | 10303.00 | 6.41 | 2.19 | 110.50 | 2418.90 | 47823.00 | 56575.00 | 3932.00 | 305.09 | 505.54 | 14.67 | 2368.30 | 652.92 | 4.16 | 2880.10 | 2.62 | 0.00 | 9.21 | 35.68 | 0.00 | 79.29 | 2194.50 | 149.46 | 206.54 | 697.86 | 31.28 | 4.29 | 5.93 | 7.98 | 55.93 | 3.70 | 3.14 | 905.34 | 17.92 | 9.04 | 1962.60 | 13.01 | 40.72 | 25.93 |
| 115756 | **NorCOMM2** | **(null)** | **C57BL/6NCrl** | **+/+** | **M** | **115756** | **6/5/2014** | NA | 7.09 | 95.57 | 15.21 | 220.33 | 832.02 | 18.29 | 20328.00 | 7486.60 | 3844.60 | 5153.10 | 0.00 | 454.54 | 10166.00 | 6.94 | 4.21 | 87.57 | 1048.90 | 25855.00 | 23927.00 | 2895.10 | 208.69 | 255.36 | 16.87 | 2249.30 | 417.45 | 5.09 | 2258.20 | 3.66 | 4.40 | 11.66 | 13.42 | 13.03 | 103.39 | 2599.90 | 107.09 | 174.32 | 504.13 | 31.85 | 3.55 | 6.65 | 9.53 | 64.68 | 2.65 | 2.55 | 787.34 | 16.96 | 8.94 | 1767.30 | 7.84 | 33.99 | 21.14 |
| 38433 | **NorCOMM2** | **(null)** | **C57BL/6NCrl** | **+/+** | **M** | **38433** | **12/11/2013** | NA | 18.62 | 124.18 | 13.24 | 216.59 | 1957.40 | 96.06 | 24427.00 | 7133.00 | 3748.90 | 4212.50 | 0.00 | 431.89 | 9276.90 | 6.82 | 3.76 | 104.63 | 1633.60 | 31357.00 | 45660.00 | 3577.10 | 235.73 | 502.15 | 20.12 | 1885.60 | 623.68 | 5.36 | 2300.90 | 13.07 | 4.11 | 13.52 | 22.42 | 11.13 | 79.98 | 2269.80 | 151.36 | 178.58 | 591.27 | 26.91 | 3.93 | 7.68 | 8.67 | 220.53 | 4.58 | 4.28 | 1066.50 | 13.04 | 11.66 | 2155.80 | 11.90 | 35.43 | 22.72 |
| 144609 | **NorCOMM2** | **(null)** | **C57BL/6NCrl** | **+/+** | **M** | **144609** | **8/7/2014** | NA | 14.22 | 94.01 | 15.12 | 279.75 | 952.91 | 20.71 | 21596.00 | 6573.90 | 4546.00 | 2573.20 | 0.00 | 454.94 | 10841.00 | 6.76 | 3.96 | 120.42 | 1934.00 | 36294.00 | 61161.00 | 3731.70 | 185.23 | 517.19 | 17.01 | 2218.10 | 530.93 | 4.10 | 3078.20 | 0.00 | 7.89 | 9.28 | 31.90 | 21.73 | 127.86 | 1982.10 | 103.39 | 193.32 | 764.60 | 37.01 | 3.23 | 6.18 | 7.09 | 94.26 | 3.74 | 2.81 | 845.56 | 14.30 | 6.18 | 1811.30 | 11.09 | 33.59 | 24.66 |
| 204873 | **NorCOMM2** | **A2m** | **A2m_tm1b_C10** | **-/-** | **F** | **204873** | **12/23/2014** | NA | 4.07 | 73.77 | 25.18 | 343.41 | 1163.90 | 63.04 | 12850.00 | 7161.30 | 3423.70 | 457.81 | 128.15 | 437.31 | 7381.50 | 0.00 | 2.31 | 87.55 | 1645.50 | 24429.00 | 32261.00 | 3048.10 | 301.55 | 283.48 | 22.01 | 1965.70 | 428.85 | 4.30 | 2304.30 | 0.00 | 0.00 | 8.94 | 45.41 | 3.58 | 42.71 | 3353.90 | 85.75 | 154.16 | 462.82 | 44.73 | 3.29 | 6.73 | 3.68 | 124.28 | 2.91 | 3.36 | 724.79 | 22.13 | 11.92 | 982.69 | 6.56 | 33.41 | 28.27 |
| 204868 | **NorCOMM2** | **A2m** | **A2m_tm1b_C10** | **-/-** | **F** | **204868** | **12/23/2014** | NA | 6.64 | 121.12 | 25.47 | 350.43 | 1250.30 | 90.88 | 14369.00 | 6259.20 | 3535.90 | 243.03 | 707.51 | 467.24 | 8399.60 | 0.00 | 2.94 | 102.79 | 1787.60 | 26068.00 | 38729.00 | 3757.50 | 220.10 | 308.26 | 22.87 | 3117.70 | 508.45 | 4.56 | 3029.90 | 0.00 | 1.58 | 12.16 | 59.34 | 3.91 | 75.32 | 3812.30 | 98.41 | 172.56 | 567.99 | 35.98 | 3.12 | 6.36 | 9.71 | 123.23 | 2.59 | 3.43 | 925.65 | 24.14 | 8.89 | 1224.70 | 9.32 | 37.52 | 30.11 |
| 204872 | **NorCOMM2** | **A2m** | **A2m_tm1b_C10** | **-/-** | **F** | **204872** | **12/23/2014** | NA | 6.66 | 90.61 | 22.75 | 373.50 | 1296.40 | 78.10 | 17165.00 | 7761.70 | 3280.10 | 226.55 | 184.29 | 431.24 | 8120.30 | 0.00 | 2.23 | 92.39 | 1952.90 | 23640.00 | 39225.00 | 3307.80 | 334.85 | 315.87 | 29.54 | 3018.40 | 438.16 | 4.30 | 2131.90 | 6.68 | 1.27 | 11.10 | 34.77 | 3.39 | 48.72 | 2884.20 | 73.62 | 160.93 | 448.09 | 32.63 | 3.70 | 6.36 | 8.72 | 252.70 | 4.19 | 3.25 | 845.46 | 21.92 | 9.93 | 1081.90 | 8.75 | 36.05 | 24.18 |
| 197966 | **NorCOMM2** | **A2m** | **A2m_tm1b_C10** | **-/-** | **M** | **197966** | **12/18/2014** | NA | 16.00 | 87.37 | 8.26 | 222.78 | 1213.70 | 29.97 | 29967.00 | 11997.00 | 4449.10 | 3762.80 | 0.00 | 598.84 | 10239.00 | 0.00 | 3.00 | 113.70 | 2144.60 | 39425.00 | 59928.00 | 3651.90 | 291.95 | 604.76 | 19.75 | 2430.70 | 626.92 | 7.46 | 2761.60 | 3.09 | 1.91 | 11.56 | 36.75 | 6.36 | 75.75 | 2513.70 | 150.62 | 201.70 | 667.37 | 30.28 | 2.14 | 6.68 | 6.67 | 131.96 | 3.69 | 2.79 | 961.75 | 15.81 | 8.42 | 2353.60 | 9.74 | 41.92 | 30.89 |
| 197962 | **NorCOMM2** | **A2m** | **A2m_tm1b_C10** | **-/-** | **M** | **197962** | **12/18/2014** | NA | 8.17 | 77.39 | 11.74 | 254.77 | 1554.00 | 42.70 | 28412.00 | 7691.30 | 4419.50 | 5109.80 | 0.00 | 522.16 | 9519.00 | 0.00 | 3.09 | 118.82 | 1946.60 | 32758.00 | 54816.00 | 4089.90 | 291.36 | 642.79 | 22.04 | 2407.80 | 560.43 | 4.51 | 2603.20 | 6.44 | 2.42 | 8.99 | 55.91 | 7.28 | 57.28 | 2265.00 | 145.73 | 202.98 | 528.71 | 25.98 | 3.83 | 8.34 | 8.67 | 132.96 | 4.66 | 5.55 | 985.13 | 18.46 | 11.35 | 2124.70 | 11.04 | 50.46 | 35.81 |
| 197964 | **NorCOMM2** | **A2m** | **A2m_tm1b_C10** | **-/-** | **M** | **197964** | **12/18/2014** | NA | 5.35 | 79.20 | 14.58 | 292.08 | 1325.10 | 24.58 | 21544.00 | 8778.30 | 4220.80 | 3438.80 | 0.00 | 480.42 | 11960.00 | 0.00 | 2.28 | 99.86 | 1601.90 | 41056.00 | 58198.00 | 3860.00 | 277.71 | 710.86 | 17.25 | 1559.40 | 527.28 | 4.28 | 3084.90 | 2.90 | 0.00 | 9.03 | 35.77 | 3.69 | 48.66 | 2333.80 | 125.41 | 190.42 | 597.40 | 34.99 | 3.55 | 9.00 | 9.87 | 103.26 | 3.57 | 2.78 | 1000.80 | 14.56 | 15.02 | 1785.00 | 12.19 | 42.77 | 26.90 |
| 181060 | **NorCOMM2** | **Ahcy** | **Ahcy_tm1b_E07** | **+/-** | **F** | **181060** | **11/6/2014** | NA | 0.00 | 75.05 | 21.60 | 303.59 | 1030.20 | 60.78 | 16642.00 | 8771.80 | 4073.90 | 187.46 | 1169.30 | 571.56 | 8792.20 | 22.95 | 1.83 | 92.79 | 1960.40 | 22079.00 | 38033.00 | 3241.80 | 397.10 | 343.09 | 27.94 | 2010.90 | 494.93 | 3.49 | 2559.50 | 3.04 | 1.14 | 12.53 | 85.12 | 3.22 | 53.74 | 3669.90 | 95.02 | 185.19 | 583.09 | 51.06 | 3.97 | 5.77 | 11.89 | 206.89 | 4.65 | 2.69 | 805.38 | 25.21 | 8.98 | 1082.30 | 9.29 | 37.13 | 32.93 |
| 179053 | **NorCOMM2** | **Ahcy** | **Ahcy_tm1b_E07** | **+/-** | **F** | **179053** | **10/23/2014** | NA | 6.25 | 84.80 | 23.54 | 346.15 | 1343.30 | 61.89 | 16469.00 | 5666.70 | 3999.20 | 291.12 | 1001.70 | 481.14 | 8603.30 | 14.89 | 1.84 | 88.79 | 1831.50 | 14865.00 | 37843.00 | 3332.00 | 278.93 | 326.30 | 20.63 | 2836.50 | 507.26 | 5.31 | 2455.10 | 0.00 | 0.00 | 10.76 | 42.84 | 2.61 | 46.27 | 3451.10 | 93.98 | 182.51 | 503.53 | 55.67 | 7.58 | 6.04 | 12.99 | 74.46 | 4.73 | 2.54 | 761.38 | 22.27 | 10.52 | 1072.80 | 7.15 | 37.09 | 31.02 |
| 181059 | **NorCOMM2** | **Ahcy** | **Ahcy_tm1b_E07** | **+/-** | **F** | **181059** | **11/6/2014** | NA | 4.78 | 78.95 | 35.96 | 393.50 | 1970.00 | 90.73 | 17639.00 | 7336.60 | 4498.10 | 89.81 | 982.48 | 560.16 | 9610.70 | 9.00 | 2.06 | 121.50 | 2024.90 | 25103.00 | 37316.00 | 3581.70 | 307.75 | 360.41 | 20.47 | 2797.90 | 477.41 | 7.59 | 2649.90 | 15.38 | 0.98 | 10.49 | 31.61 | 5.03 | 53.96 | 3476.20 | 93.47 | 187.13 | 616.42 | 46.00 | 4.08 | 7.72 | 14.92 | 200.60 | 3.52 | 1.98 | 814.19 | 25.26 | 11.38 | 1270.50 | 9.24 | 43.83 | 34.31 |
| 178951 | **NorCOMM2** | **Ahcy** | **Ahcy_tm1b_E07** | **+/-** | **M** | **178951** | **10/23/2014** | NA | 8.54 | 115.13 | 9.95 | 218.06 | 1910.20 | 82.86 | 27190.00 | 9703.20 | 3981.50 | 5629.30 | 0.00 | 489.34 | 8962.20 | 0.00 | 3.80 | 110.57 | 2040.50 | 32072.00 | 45274.00 | 3844.20 | 259.71 | 569.19 | 27.25 | 1721.00 | 496.51 | 8.53 | 2332.90 | 3.66 | 2.03 | 12.08 | 12.19 | 7.99 | 67.02 | 2052.60 | 158.29 | 187.74 | 571.51 | 21.77 | 3.69 | 7.50 | 11.23 | 64.24 | 2.94 | 4.19 | 843.83 | 12.58 | 11.37 | 2304.40 | 13.48 | 43.11 | 29.23 |
| 178948 | **NorCOMM2** | **Ahcy** | **Ahcy_tm1b_E07** | **+/-** | **M** | **178948** | **10/23/2014** | NA | 10.25 | 97.91 | 11.52 | 254.85 | 1787.70 | 46.93 | 30926.00 | 7921.50 | 4285.60 | 7062.70 | 0.00 | 564.73 | 9760.20 | 6.43 | 2.08 | 95.85 | 1839.60 | 33033.00 | 41426.00 | 3918.40 | 203.37 | 436.20 | 19.93 | 2136.70 | 561.27 | 6.19 | 2497.30 | 3.51 | 1.27 | 12.57 | 20.16 | 3.68 | 43.17 | 2251.20 | 145.24 | 195.02 | 575.93 | 24.65 | 3.89 | 8.08 | 8.91 | 64.50 | 4.51 | 3.01 | 886.92 | 13.27 | 10.04 | 1995.40 | 9.23 | 40.74 | 31.10 |
| 178950 | **NorCOMM2** | **Ahcy** | **Ahcy_tm1b_E07** | **+/-** | **M** | **178950** | **10/23/2014** | NA | 7.86 | 91.78 | 15.80 | 241.51 | 1232.60 | 42.03 | 25273.00 | 7493.20 | 4669.40 | 2738.80 | 0.00 | 585.62 | 11175.00 | 6.95 | 2.19 | 112.87 | 1981.40 | 34736.00 | 54928.00 | 3879.80 | 320.41 | 814.47 | 20.93 | 1665.10 | 513.86 | 6.38 | 2757.70 | 7.22 | 0.00 | 11.88 | 24.31 | 0.00 | 47.95 | 2568.40 | 159.64 | 201.52 | 730.71 | 31.84 | 3.03 | 8.55 | 7.45 | 106.17 | 4.06 | 3.73 | 897.76 | 17.15 | 10.00 | 2052.60 | 11.20 | 38.62 | 34.16 |
| 143963 | **NorCOMM2** | **Atp5b** | **Atp5b_tm1b_B07** | **+/-** | **F** | **143963** | **8/7/2014** | NA | 9.88 | 365.50 | 17.30 | 276.88 | 1194.84 | 57.40 | 18074.24 | 7425.65 | 3369.71 | 217.72 | 502.39 | 489.32 | 6540.63 | 20.41 | 10.24 | 122.19 | 2134.04 | 23603.95 | 36506.47 | 2937.82 | 327.41 | 314.64 | 24.62 | 2670.20 | 486.54 | 3.97 | 2929.32 | 0.00 | 0.00 | 10.06 | 29.80 | 0.00 | 49.41 | 4473.35 | 85.13 | 162.79 | 412.44 | 49.07 | 3.75 | 5.30 | 11.52 | 70.37 | 3.87 | 0.00 | 750.35 | 30.09 | 9.27 | 1131.80 | 5.30 | 43.56 | 31.06 |
| 143961 | **NorCOMM2** | **Atp5b** | **Atp5b_tm1b_B07** | **+/-** | **F** | **143961** | **8/7/2014** | NA | 6.94 | 118.76 | 15.33 | 271.04 | 999.23 | 44.55 | 19471.07 | 6550.01 | 3025.62 | 336.74 | 186.93 | 422.12 | 6698.45 | 21.82 | 4.32 | 139.82 | 1780.60 | 24853.97 | 36581.49 | 3204.12 | 222.60 | 265.31 | 29.29 | 1985.69 | 482.02 | 2.97 | 2683.71 | 3.34 | 0.00 | 11.55 | 53.47 | 8.58 | 70.07 | 3903.56 | 68.41 | 159.96 | 436.42 | 42.11 | 3.74 | 5.03 | 10.46 | 91.52 | 4.73 | 0.00 | 786.02 | 20.02 | 9.26 | 1214.50 | 7.00 | 53.18 | 30.01 |
| 143960 | **NorCOMM2** | **Atp5b** | **Atp5b_tm1b_B07** | **+/-** | **F** | **143960** | **8/7/2014** | NA | 11.64 | 175.91 | 18.40 | 295.49 | 1136.20 | 66.26 | 23718.30 | 7186.38 | 3112.73 | 391.48 | 294.61 | 489.00 | 6864.19 | 20.18 | 5.27 | 125.20 | 2001.10 | 27854.23 | 39193.47 | 3137.25 | 217.17 | 321.29 | 31.83 | 2085.51 | 548.09 | 3.46 | 2986.55 | 0.00 | 0.00 | 12.32 | 76.09 | 0.00 | 40.43 | 3993.82 | 85.90 | 181.65 | 567.30 | 39.23 | 3.33 | 4.82 | 10.11 | 81.26 | 4.15 | 0.00 | 783.31 | 26.39 | 7.64 | 1270.63 | 7.48 | 36.39 | 33.35 |
| 118347 | **NorCOMM2** | **Atp5b** | **Atp5b_tm1b_B07** | **+/-** | **M** | **118347** | **6/11/2014** | NA | 9.67 | 121.73 | 11.35 | 167.19 | 1842.78 | 144.74 | 30416.66 | 7704.27 | 3540.05 | 6039.82 | 0.00 | 477.46 | 7975.98 | 7.67 | 7.84 | 102.48 | 2239.82 | 29347.80 | 51116.16 | 3572.32 | 383.83 | 445.88 | 18.70 | 2787.27 | 586.30 | 4.75 | 2755.87 | 19.02 | 0.00 | 10.68 | 19.81 | 7.57 | 74.28 | 2422.65 | 148.27 | 164.65 | 503.86 | 30.38 | 2.45 | 6.24 | 14.44 | 116.88 | 3.55 | 5.47 | 844.30 | 13.31 | 10.61 | 2271.08 | 10.42 | 42.91 | 30.64 |
| 118346 | **NorCOMM2** | **Atp5b** | **Atp5b_tm1b_B07** | **+/-** | **M** | **118346** | **6/11/2014** | NA | 8.51 | 76.52 | 8.29 | 172.44 | 1874.68 | 33.48 | 25349.54 | 7877.48 | 3608.71 | 4184.24 | 0.00 | 421.29 | 8349.45 | 8.12 | 2.50 | 121.46 | 2128.93 | 29064.85 | 40976.25 | 3734.28 | 291.57 | 492.31 | 17.91 | 2203.10 | 490.61 | 3.83 | 2289.07 | 5.03 | 0.00 | 10.93 | 22.16 | 3.36 | 47.67 | 2974.23 | 129.94 | 163.72 | 599.02 | 33.49 | 2.89 | 6.05 | 5.10 | 99.06 | 3.20 | 0.00 | 760.70 | 14.55 | 7.94 | 1750.36 | 8.82 | 43.95 | 26.96 |
| 143858 | **NorCOMM2** | **Atp5b** | **Atp5b_tm1b_B07** | **+/-** | **M** | **143858** | **8/7/2014** | NA | 18.26 | 137.76 | 7.74 | 116.31 | 2386.57 | 1705.00 | 10747.57 | 2345.65 | 1502.30 | 3095.42 | 0.00 | 364.16 | 2204.85 | 0.00 | 4.04 | 114.37 | 1832.27 | 17581.19 | 10957.27 | 3005.86 | 222.43 | 156.26 | 14.69 | 1690.87 | 246.03 | 6.78 | 2171.70 | 0.00 | 0.00 | 12.35 | 7.72 | 7.93 | 39.19 | 2877.75 | 161.36 | 155.90 | 535.01 | 30.84 | 5.16 | 12.58 | 20.52 | 462.39 | 5.73 | 12.16 | 815.77 | 26.54 | 13.98 | 2648.47 | 4.82 | 50.63 | 14.04 |
| 281686 | **NorCOMM2** | **Atp6v0d1** | **Atp6v0d1_tm1b_E01** | **+/-** | **F** | **281686** | **6/24/2015** | NA | 6.40 | 95.26 | 26.45 | 222.67 | 870.53 | 78.30 | 18011.73 | 7978.15 | 3540.29 | 39.28 | 79.57 | 401.09 | 6258.82 | 16.85 | 1.63 | 111.43 | 2134.29 | 22864.46 | 33099.25 | 3557.82 | 212.68 | 365.82 | 14.46 | 1734.87 | 428.64 | 4.50 | 2451.65 | 0.00 | 0.00 | 13.43 | 22.90 | 0.00 | 28.48 | 4202.93 | 76.39 | 158.96 | 444.27 | 41.33 | 3.12 | 7.90 | 9.91 | 64.80 | 3.72 | 4.15 | 817.55 | 20.85 | 11.66 | 1184.03 | 7.12 | 35.09 | 25.47 |
| 281679 | **NorCOMM2** | **Atp6v0d1** | **Atp6v0d1_tm1b_E01** | **+/-** | **F** | **281679** | **6/24/2015** | NA | 7.55 | 57.36 | 19.40 | 296.88 | 1016.66 | 49.22 | 15489.80 | 6015.71 | 2461.17 | 262.63 | 775.22 | 396.10 | 6423.29 | 16.30 | 1.30 | 109.50 | 1676.32 | 26326.90 | 30918.94 | 2455.59 | 233.88 | 345.96 | 28.29 | 1853.36 | 464.56 | 3.39 | 2370.35 | 0.00 | 0.00 | 9.39 | 24.07 | 0.00 | 36.35 | 4198.79 | 78.58 | 155.03 | 464.02 | 39.89 | 3.52 | 4.52 | 8.48 | 62.83 | 3.18 | 4.19 | 732.05 | 24.13 | 7.90 | 1029.09 | 6.13 | 27.15 | 28.15 |
| 281678 | **NorCOMM2** | **Atp6v0d1** | **Atp6v0d1_tm1b_E01** | **+/-** | **F** | **281678** | **6/24/2015** | NA | 6.90 | 76.32 | 28.13 | 221.85 | 1343.75 | 124.64 | 12174.07 | 7254.78 | 3371.46 | 42.95 | 34.06 | 551.51 | 5757.86 | 14.90 | 2.46 | 117.32 | 2525.33 | 19377.14 | 35855.71 | 3181.82 | 176.08 | 334.21 | 11.51 | 1099.28 | 493.07 | 2.55 | 2087.98 | 2.62 | 2.37 | 12.25 | 123.54 | 4.83 | 65.07 | 5189.46 | 86.28 | 158.43 | 582.89 | 42.09 | 3.78 | 8.21 | 4.73 | 35.80 | 3.85 | 3.88 | 1005.10 | 23.52 | 7.84 | 1166.11 | 5.13 | 28.58 | 25.15 |
| 281575 | **NorCOMM2** | **Atp6v0d1** | **Atp6v0d1_tm1b_E01** | **+/-** | **M** | **281575** | **6/23/2015** | NA | 13.34 | 89.47 | 14.22 | 182.45 | 819.08 | 24.62 | 26002.00 | 9314.83 | 3653.53 | 3485.30 | 0.00 | 398.72 | 8225.45 | 6.75 | 2.50 | 128.81 | 2068.15 | 35950.57 | 50629.52 | 4497.25 | 318.18 | 341.63 | 13.85 | 1901.25 | 536.21 | 4.21 | 3334.42 | 0.00 | 2.05 | 11.08 | 17.31 | 5.98 | 59.47 | 3551.81 | 114.95 | 167.06 | 471.96 | 31.23 | 4.12 | 8.21 | 8.10 | 59.91 | 3.76 | 4.39 | 853.96 | 17.82 | 6.99 | 2199.84 | 8.54 | 32.46 | 22.04 |
| 281582 | **NorCOMM2** | **Atp6v0d1** | **Atp6v0d1_tm1b_E01** | **+/-** | **M** | **281582** | **6/23/2015** | NA | 15.44 | 80.83 | 12.58 | 157.96 | 1396.20 | 42.85 | 26541.91 | 7914.97 | 2972.11 | 5504.42 | 0.00 | 522.79 | 7209.33 | 7.13 | 1.80 | 136.76 | 2311.01 | 33581.70 | 40565.57 | 4984.74 | 336.27 | 540.66 | 22.31 | 2385.81 | 536.84 | 5.10 | 2385.46 | 2.75 | 0.00 | 6.64 | 14.84 | 3.58 | 48.72 | 3441.74 | 144.95 | 169.66 | 397.76 | 31.64 | 3.17 | 7.40 | 7.63 | 77.92 | 3.38 | 5.78 | 837.00 | 16.73 | 11.42 | 1768.75 | 8.54 | 43.04 | 29.66 |
| 281584 | **NorCOMM2** | **Atp6v0d1** | **Atp6v0d1_tm1b_E01** | **+/-** | **M** | **281584** | **6/23/2015** | NA | 6.72 | 109.50 | 11.73 | 188.43 | 1680.47 | 70.88 | 28901.25 | 8738.89 | 3697.83 | 4786.78 | 0.00 | 508.02 | 8408.19 | 6.59 | 2.32 | 124.91 | 2486.39 | 44394.13 | 51837.07 | 5284.37 | 386.00 | 563.05 | 17.82 | 2545.06 | 545.23 | 4.40 | 3343.67 | 2.83 | 0.00 | 12.55 | 19.51 | 0.00 | 55.84 | 2302.77 | 131.32 | 185.83 | 586.37 | 32.32 | 4.16 | 10.09 | 11.01 | 87.13 | 4.49 | 5.25 | 970.72 | 14.68 | 10.55 | 2610.89 | 10.32 | 44.91 | 29.58 |
| 222890 | **NorCOMM2** | **C8a** | **C8a_tm1b_F01** | **-/-** | **F** | **222890** | **2/18/2015** | NA | 4.10 | 108.22 | 22.05 | 257.78 | 1077.53 | 38.20 | 14318.66 | 7080.52 | 3131.10 | 86.48 | 166.23 | 368.42 | 4225.16 | 31.63 | 2.62 | 69.25 | 1494.90 | 26955.36 | 36267.11 | 2901.05 | 259.64 | 490.81 | 12.50 | 2824.96 | 427.72 | 2.58 | 2253.17 | 3.21 | 1.48 | 12.20 | 6.85 | 0.00 | 38.28 | 4264.17 | 79.21 | 147.20 | 434.31 | 24.00 | 2.90 | 7.47 | 7.34 | 83.64 | 3.79 | 3.16 | 823.82 | 16.11 | 10.88 | 887.73 | 6.64 | 33.92 | 22.03 |
| 222888 | **NorCOMM2** | **C8a** | **C8a_tm1b_F01** | **-/-** | **F** | **222888** | **2/18/2015** | NA | 11.98 | 95.69 | 21.29 | 289.97 | 1085.15 | 95.50 | 14213.29 | 6833.96 | 2497.75 | 193.35 | 267.92 | 493.40 | 7236.03 | 30.56 | 2.15 | 128.37 | 2001.55 | 24319.31 | 32167.93 | 3080.21 | 230.20 | 399.97 | 37.79 | 2319.53 | 484.75 | 3.69 | 2846.06 | 3.58 | 0.00 | 13.75 | 21.74 | 0.00 | 45.98 | 3504.47 | 78.96 | 147.35 | 479.87 | 32.54 | 3.37 | 5.61 | 10.31 | 98.43 | 4.09 | 3.71 | 792.01 | 20.27 | 9.63 | 1063.62 | 6.45 | 42.93 | 27.28 |
| 222895 | **NorCOMM2** | **C8a** | **C8a_tm1b_F01** | **-/-** | **F** | **222895** | **2/18/2015** | NA | 8.60 | 110.91 | 21.64 | 316.08 | 1257.92 | 73.86 | 14446.11 | 6566.65 | 3215.85 | 89.40 | 847.52 | 529.39 | 7328.44 | 19.19 | 2.90 | 113.38 | 2198.31 | 29074.36 | 37494.40 | 3282.83 | 307.43 | 467.49 | 29.47 | 2785.39 | 501.07 | 5.87 | 2580.14 | 3.32 | 0.00 | 10.11 | 20.47 | 0.00 | 40.10 | 4184.03 | 91.59 | 176.32 | 553.43 | 47.31 | 3.77 | 6.26 | 13.95 | 91.18 | 3.85 | 4.29 | 826.07 | 26.27 | 8.50 | 1258.73 | 6.39 | 36.36 | 35.21 |
| 233514 | **NorCOMM2** | **C8a** | **C8a_tm1b_F01** | **-/-** | **M** | **233514** | **4/8/2015** | NA | 14.95 | 88.60 | 14.36 | 172.72 | 1585.81 | 41.79 | 33091.08 | 8435.93 | 3225.41 | 4146.67 | 0.00 | 582.74 | 7361.20 | 6.26 | 1.85 | 140.21 | 1996.74 | 45062.61 | 46768.10 | 3725.90 | 281.63 | 581.24 | 17.68 | 2274.24 | 529.07 | 4.93 | 3237.18 | 3.79 | 0.00 | 10.65 | 13.47 | 0.00 | 49.08 | 3220.63 | 126.46 | 188.18 | 590.76 | 28.19 | 3.36 | 7.00 | 9.74 | 89.25 | 3.57 | 6.83 | 862.37 | 13.68 | 8.20 | 2160.57 | 8.78 | 35.51 | 28.30 |
| 233523 | **NorCOMM2** | **C8a** | **C8a_tm1b_F01** | **-/-** | **M** | **233523** | **4/8/2015** | NA | 8.57 | 116.65 | 11.52 | 193.20 | 1501.98 | 27.89 | 29956.99 | 9098.55 | 3640.40 | 6123.89 | 0.00 | 489.06 | 7891.13 | 7.08 | 2.72 | 113.62 | 2199.54 | 34614.70 | 49445.64 | 3415.28 | 278.46 | 599.50 | 16.55 | 2821.06 | 515.02 | 5.20 | 2557.01 | 7.82 | 0.00 | 11.94 | 15.60 | 0.00 | 43.57 | 2771.84 | 150.35 | 180.55 | 688.04 | 28.72 | 3.48 | 6.35 | 6.66 | 90.43 | 3.50 | 4.59 | 830.80 | 13.13 | 10.38 | 2672.60 | 12.35 | 36.92 | 31.08 |
| 233515 | **NorCOMM2** | **C8a** | **C8a_tm1b_F01** | **-/-** | **M** | **233515** | **4/8/2015** | NA | 11.78 | 94.61 | 10.82 | 201.32 | 1297.83 | 44.24 | 27905.73 | 8677.73 | 3322.81 | 6474.85 | 0.00 | 602.47 | 8475.51 | 0.00 | 1.30 | 126.15 | 1996.29 | 46379.01 | 47238.70 | 4239.44 | 254.73 | 564.16 | 27.63 | 2294.45 | 537.07 | 4.09 | 2964.89 | 0.00 | 0.00 | 12.63 | 17.87 | 0.00 | 47.99 | 3751.37 | 130.47 | 189.85 | 703.23 | 28.98 | 3.05 | 8.22 | 9.46 | 91.81 | 3.13 | 4.84 | 917.53 | 15.59 | 11.57 | 1994.76 | 10.91 | 40.76 | 30.90 |
| 206829 | **NorCOMM2** | **Cdk4** | **Cdk4_tm1b_F08** | **+/-** | **F** | **206829** | **1/8/2015** | NA | 4.49 | 130.67 | 21.65 | 349.11 | 1077.63 | 82.44 | 14247.41 | 8024.73 | 3347.04 | 240.13 | 962.00 | 543.84 | 8130.77 | 23.75 | 2.36 | 116.96 | 1936.64 | 19794.66 | 34783.32 | 2413.67 | 227.58 | 212.37 | 27.54 | 2136.93 | 456.29 | 3.41 | 2171.65 | 4.06 | 0.00 | 13.86 | 64.97 | 0.00 | 62.52 | 4153.78 | 76.87 | 164.00 | 586.67 | 44.28 | 4.13 | 6.36 | 9.73 | 180.23 | 5.32 | 4.74 | 921.35 | 32.42 | 7.86 | 1248.56 | 8.82 | 36.76 | 25.85 |
| 206828 | **NorCOMM2** | **Cdk4** | **Cdk4_tm1b_F08** | **+/-** | **F** | **206828** | **1/6/2015** | NA | 5.58 | 101.68 | 23.01 | 273.64 | 980.79 | 82.97 | 13941.33 | 6168.30 | 3036.11 | 170.62 | 522.13 | 491.46 | 6483.88 | 15.66 | 1.93 | 101.73 | 1975.70 | 19621.60 | 29277.30 | 2761.81 | 270.00 | 343.27 | 22.89 | 1647.30 | 375.17 | 4.36 | 2286.98 | 0.00 | 0.00 | 11.02 | 23.51 | 3.14 | 50.98 | 4325.63 | 69.18 | 147.53 | 444.58 | 49.49 | 3.05 | 5.40 | 6.54 | 106.85 | 3.36 | 4.75 | 801.60 | 20.74 | 7.18 | 1176.78 | 6.10 | 35.45 | 23.92 |
| 206822 | **NorCOMM2** | **Cdk4** | **Cdk4_tm1b_F08** | **+/-** | **F** | **206822** | **1/6/2015** | NA | 4.70 | 127.79 | 19.67 | 279.39 | 1144.21 | 77.52 | 12922.72 | 6143.80 | 3060.53 | 456.12 | 639.64 | 453.86 | 7090.41 | 13.77 | 2.09 | 89.90 | 2149.85 | 18373.34 | 32880.23 | 3191.90 | 265.11 | 311.06 | 24.60 | 2137.37 | 431.15 | 4.02 | 2394.91 | 3.03 | 0.00 | 9.24 | 29.31 | 2.58 | 49.68 | 3926.73 | 70.94 | 157.47 | 542.09 | 33.43 | 3.54 | 5.21 | 7.84 | 99.80 | 3.37 | 4.00 | 768.21 | 23.25 | 11.60 | 985.95 | 7.33 | 34.37 | 26.59 |
| 190208 | **NorCOMM2** | **Cdk4** | **Cdk4_tm1b_F08** | **+/-** | **M** | **190208** | **11/25/2014** | NA | 19.52 | 87.31 | 10.61 | 188.20 | 1301.94 | 63.79 | 26743.62 | 8852.80 | 3693.10 | 5278.52 | 0.00 | 412.26 | 8728.31 | 7.39 | 4.14 | 101.35 | 1729.26 | 35804.55 | 49886.64 | 4988.23 | 301.56 | 661.06 | 25.04 | 2117.15 | 559.51 | 4.60 | 2716.34 | 15.13 | 3.21 | 10.34 | 18.69 | 7.55 | 51.50 | 2188.88 | 127.60 | 175.56 | 574.12 | 26.67 | 3.42 | 6.18 | 8.68 | 107.41 | 2.09 | 5.19 | 857.90 | 13.78 | 9.39 | 1815.76 | 13.69 | 44.01 | 21.17 |
| 190207 | **NorCOMM2** | **Cdk4** | **Cdk4_tm1b_F08** | **+/-** | **M** | **190207** | **11/25/2014** | NA | 11.82 | 89.55 | 13.63 | 219.28 | 2365.53 | 270.88 | 28849.07 | 9771.19 | 3644.07 | 5392.29 | 0.00 | 586.53 | 8183.41 | 8.25 | 2.42 | 145.58 | 2590.36 | 44293.54 | 53412.62 | 5372.31 | 351.12 | 569.33 | 27.36 | 2414.24 | 596.55 | 5.12 | 2849.97 | 4.90 | 1.26 | 17.84 | 16.95 | 2.81 | 61.93 | 2489.38 | 160.37 | 186.51 | 576.11 | 22.63 | 3.51 | 8.32 | 8.41 | 81.10 | 3.81 | 5.49 | 1244.28 | 14.68 | 8.19 | 2153.22 | 9.94 | 41.19 | 29.19 |
| 190206 | **NorCOMM2** | **Cdk4** | **Cdk4_tm1b_F08** | **+/-** | **M** | **190206** | **11/25/2014** | NA | 14.67 | 102.32 | 6.61 | 177.57 | 3209.83 | 350.97 | 38686.88 | 9288.50 | 3977.97 | 5354.81 | 0.00 | 659.38 | 8390.96 | 6.56 | 2.72 | 125.18 | 2150.63 | 40711.03 | 56311.15 | 5608.16 | 463.16 | 751.64 | 25.41 | 3253.29 | 593.36 | 5.85 | 3659.75 | 3.62 | 1.46 | 19.52 | 19.79 | 0.00 | 55.45 | 2425.05 | 178.75 | 194.88 | 660.14 | 24.90 | 4.56 | 9.02 | 8.99 | 85.53 | 3.55 | 5.64 | 1110.21 | 17.03 | 11.81 | 2319.84 | 10.63 | 46.81 | 39.77 |
| 122069 | **NorCOMM2** | **Dhfr** | **Dhfr_tm1b_H07** | **+/-** | **F** | **122069** | **6/19/2014** | NA | 7.74 | 95.45 | 34.72 | 322.13 | 1044.50 | 45.70 | 16173.00 | 6606.90 | 3941.20 | 175.50 | 114.35 | 507.96 | 9109.00 | 15.44 | 3.86 | 127.96 | 1885.40 | 21178.00 | 38710.00 | 2971.00 | 303.98 | 241.60 | 19.90 | 2276.80 | 453.56 | 4.87 | 2295.40 | 4.57 | 2.97 | 11.24 | 34.96 | 9.42 | 90.18 | 3458.90 | 71.46 | 170.39 | 596.83 | 39.79 | 3.27 | 7.44 | 9.94 | 108.69 | 3.57 | 3.11 | 839.82 | 25.01 | 13.64 | 1081.10 | 7.34 | 31.95 | 36.11 |
| 122067 | **NorCOMM2** | **Dhfr** | **Dhfr_tm1b_H07** | **+/-** | **F** | **122067** | **6/19/2014** | NA | 7.60 | 87.15 | 25.07 | 287.67 | 929.98 | 40.77 | 15575.00 | 5757.50 | 3699.30 | 264.24 | 7.22 | 423.07 | 9284.50 | 14.69 | 2.15 | 122.69 | 1970.30 | 22458.00 | 38725.00 | 2875.00 | 338.22 | 244.38 | 10.32 | 2196.60 | 438.21 | 5.70 | 2880.50 | 7.09 | 2.30 | 14.32 | 58.36 | 6.12 | 65.31 | 3867.20 | 87.15 | 170.28 | 656.32 | 43.86 | 3.08 | 7.44 | 11.30 | 91.08 | 4.69 | 2.25 | 843.16 | 22.32 | 11.06 | 1100.80 | 7.87 | 32.87 | 32.36 |
| 122066 | **NorCOMM2** | **Dhfr** | **Dhfr_tm1b_H07** | **+/-** | **F** | **122066** | **6/19/2014** | NA | 3.69 | 100.58 | 26.75 | 285.75 | 832.27 | 52.03 | 11199.00 | 5719.60 | 2878.50 | 273.17 | 209.78 | 484.46 | 9687.90 | 21.07 | 2.06 | 105.31 | 1554.50 | 27405.00 | 37833.00 | 2739.10 | 344.09 | 364.62 | 30.24 | 2649.00 | 484.64 | 7.57 | 2974.50 | 3.21 | 0.83 | 13.28 | 73.61 | 4.47 | 47.57 | 2762.30 | 81.33 | 162.60 | 473.60 | 39.89 | 3.46 | 5.99 | 9.59 | 61.03 | 4.13 | 1.81 | 816.79 | 22.87 | 9.01 | 973.13 | 9.49 | 47.06 | 27.74 |
| 121964 | **NorCOMM2** | **Dhfr** | **Dhfr_tm1b_H07** | **+/-** | **M** | **121964** | **6/19/2014** | NA | 0.00 | 84.13 | 14.04 | 236.19 | 1223.00 | 20.26 | 22036.00 | 8600.90 | 4444.10 | 2512.30 | 0.00 | 412.05 | 9807.00 | 6.62 | 2.38 | 119.92 | 1920.80 | 35382.00 | 58835.00 | 3198.70 | 213.01 | 610.60 | 18.50 | 2367.20 | 517.84 | 3.59 | 3224.30 | 6.34 | 1.79 | 10.74 | 54.83 | 4.85 | 52.13 | 2303.60 | 117.97 | 194.69 | 608.97 | 34.95 | 3.12 | 7.35 | 6.55 | 76.47 | 3.98 | 3.24 | 978.76 | 20.86 | 10.94 | 1712.10 | 10.53 | 35.26 | 27.26 |
| 121962 | **NorCOMM2** | **Dhfr** | **Dhfr_tm1b_H07** | **+/-** | **M** | **121962** | **6/19/2014** | NA | 8.47 | 79.55 | 12.47 | 256.84 | 924.94 | 21.13 | 26021.00 | 7835.90 | 3866.70 | 3686.70 | 0.00 | 475.80 | 10292.00 | 7.11 | 2.10 | 107.39 | 2017.00 | 32777.00 | 49234.00 | 2730.40 | 173.11 | 451.37 | 16.79 | 2086.00 | 528.22 | 5.21 | 2526.30 | 2.62 | 0.00 | 9.79 | 24.63 | 2.65 | 50.37 | 3041.10 | 113.77 | 184.16 | 598.87 | 29.91 | 2.82 | 8.39 | 6.28 | 98.00 | 3.77 | 2.83 | 878.49 | 18.54 | 9.90 | 1633.60 | 10.97 | 44.47 | 28.74 |
| 129282 | **NorCOMM2** | **Dhfr** | **Dhfr_tm1b_H07** | **+/-** | **M** | **129282** | **7/9/2014** | NA | 5.07 | 81.39 | 11.72 | 249.96 | 1212.20 | 38.30 | 31730.00 | 7355.00 | 4420.00 | 4506.10 | 0.00 | 523.96 | 11432.00 | 6.88 | 2.85 | 94.43 | 2116.60 | 29353.00 | 51773.00 | 3627.90 | 332.96 | 693.73 | 14.63 | 1758.10 | 473.53 | 5.22 | 2895.80 | 8.54 | 0.00 | 11.61 | 27.39 | 2.50 | 50.68 | 2275.00 | 162.94 | 224.00 | 675.09 | 32.04 | 3.97 | 7.00 | 5.45 | 100.24 | 4.08 | 2.60 | 988.63 | 16.89 | 14.20 | 2284.00 | 13.52 | 38.40 | 28.67 |
| 428089 | **K2P2** | **Dync1li1** | **Dync1li1_em1_del** | **+/-** | **F** | **428089** | **8/18/2016** | NA | 6.41 | 77.63 | 31.38 | 263.11 | 684.76 | 27.77 | 16816.56 | 6219.45 | 2986.15 | 1631.06 | 1030.64 | 390.25 | 6716.27 | 16.58 | 2.00 | 92.41 | 1457.85 | 13143.96 | 16341.14 | 2858.50 | 268.77 | 254.45 | 14.30 | 1469.36 | 266.57 | 1.88 | 1927.44 | 4.85 | 0.00 | 10.12 | 13.45 | 0.00 | 46.50 | 4123.68 | 72.92 | 135.89 | 432.46 | 39.28 | 2.98 | 6.06 | 12.99 | 155.03 | 3.04 | 0.00 | 532.69 | 19.77 | 9.15 | 1162.95 | 4.52 | 36.30 | 18.76 |
| 420778 | **K2P2** | **Dync1li1** | **Dync1li1_em1_del** | **+/-** | **F** | **420778** | **8/5/2016** | NA | 3.78 | 91.07 | 19.22 | 253.45 | 1218.89 | 60.86 | 17072.38 | 7191.02 | 3449.51 | 71.62 | 698.41 | 368.22 | 6516.26 | 20.53 | 3.98 | 110.75 | 2232.68 | 21985.08 | 42963.36 | 2913.88 | 317.95 | 402.04 | 14.33 | 2774.65 | 472.64 | 4.13 | 2391.94 | 7.08 | 2.47 | 9.31 | 19.60 | 3.39 | 60.06 | 4268.29 | 70.52 | 161.17 | 592.48 | 50.82 | 3.58 | 5.42 | 13.87 | 122.84 | 3.26 | 0.00 | 739.61 | 24.11 | 11.45 | 1109.25 | 5.56 | 35.20 | 31.37 |
| 428090 | **K2P2** | **Dync1li1** | **Dync1li1_em1_del** | **+/-** | **F** | **428090** | **8/18/2016** | NA | 6.70 | 88.23 | 21.19 | 325.78 | 1361.23 | 60.06 | 16035.19 | 7481.45 | 3637.91 | 28.83 | 1117.47 | 459.36 | 7310.76 | 20.00 | 2.33 | 120.94 | 2675.70 | 25143.99 | 46663.17 | 2854.02 | 276.03 | 512.16 | 15.02 | 2202.47 | 435.01 | 3.25 | 2764.33 | 0.00 | 0.00 | 9.52 | 15.96 | 0.00 | 47.12 | 5354.64 | 102.28 | 170.18 | 823.93 | 54.62 | 3.11 | 5.60 | 4.79 | 198.61 | 3.24 | 0.00 | 795.99 | 25.50 | 7.92 | 1066.78 | 7.08 | 36.69 | 28.46 |
| 420676 | **K2P2** | **Dync1li1** | **Dync1li1_em1_del** | **+/-** | **M** | **420676** | **8/3/2016** | NA | 7.71 | 94.56 | 7.71 | 140.73 | 1408.41 | 38.58 | 35022.46 | 8299.82 | 3416.03 | 4602.31 | 0.00 | 423.26 | 6609.81 | 0.00 | 3.24 | 118.03 | 2042.65 | 37591.69 | 53693.59 | 2999.04 | 230.03 | 388.16 | 18.99 | 2708.07 | 555.00 | 5.51 | 2531.03 | 6.97 | 7.15 | 9.12 | 47.95 | 10.91 | 76.52 | 2386.73 | 127.77 | 160.97 | 505.31 | 26.51 | 3.15 | 5.94 | 6.45 | 118.68 | 4.36 | 0.00 | 793.82 | 15.11 | 7.99 | 2136.73 | 8.87 | 36.57 | 28.71 |
| 420674 | **K2P2** | **Dync1li1** | **Dync1li1_em1_del** | **+/-** | **M** | **420674** | **8/3/2016** | NA | 6.27 | 115.73 | 8.74 | 180.98 | 2301.10 | 186.12 | 27007.54 | 8165.01 | 3545.09 | 4723.16 | 0.00 | 392.94 | 7941.88 | 0.00 | 2.71 | 141.67 | 2440.40 | 40926.44 | 50450.83 | 3944.02 | 265.44 | 505.02 | 15.29 | 2193.84 | 570.20 | 3.53 | 2805.58 | 0.00 | 0.00 | 14.27 | 13.75 | 0.00 | 40.25 | 2718.38 | 145.53 | 184.07 | 677.93 | 28.25 | 3.68 | 7.93 | 8.42 | 204.04 | 3.79 | 4.75 | 911.46 | 14.52 | 10.55 | 2164.43 | 10.55 | 44.23 | 28.85 |
| 420673 | **K2P2** | **Dync1li1** | **Dync1li1_em1_del** | **+/-** | **M** | **420673** | **8/3/2016** | NA | 6.86 | 56.45 | 6.14 | 222.99 | 1119.27 | 19.63 | 26075.25 | 7775.65 | 3330.91 | 2988.24 | 0.00 | 417.98 | 7646.23 | 0.00 | 1.79 | 144.00 | 2327.59 | 38034.31 | 55549.42 | 2433.36 | 208.93 | 466.46 | 14.86 | 1882.72 | 481.16 | 4.08 | 2623.56 | 0.00 | 1.10 | 8.33 | 10.99 | 3.26 | 42.27 | 2621.81 | 108.43 | 166.00 | 481.68 | 27.60 | 3.00 | 7.14 | 9.06 | 99.91 | 3.61 | 3.97 | 767.35 | 14.81 | 7.84 | 2082.85 | 10.00 | 31.17 | 22.47 |
| 410978 | **KOMP2** | **G6pd2** | **G6pd2_em1_del** | **-/-** | **F** | **410978** | **6/16/2016** | NA | 3.97 | 91.31 | 19.57 | 292.17 | 1202.06 | 49.16 | 17018.86 | 7033.28 | 3323.21 | 124.77 | 475.34 | 371.44 | 6687.09 | 23.89 | 2.33 | 179.66 | 1882.44 | 28614.15 | 40176.66 | 2706.47 | 381.67 | 318.65 | 19.93 | 2592.90 | 508.20 | 2.99 | 2869.07 | 0.00 | 1.83 | 10.47 | 29.70 | 6.17 | 45.10 | 5249.82 | 78.61 | 164.50 | 570.05 | 50.67 | 3.65 | 4.57 | 11.54 | 63.27 | 3.34 | 0.00 | 805.64 | 26.04 | 7.00 | 1192.79 | 7.23 | 38.15 | 26.14 |
| 410980 | **KOMP2** | **G6pd2** | **G6pd2_em1_del** | **-/-** | **F** | **410980** | **6/16/2016** | NA | 2.35 | 126.28 | 19.94 | 299.03 | 1306.22 | 64.51 | 13745.67 | 6817.52 | 3301.05 | 230.57 | 786.86 | 435.39 | 7051.29 | 19.37 | 2.55 | 128.51 | 1899.27 | 26572.88 | 38243.84 | 3305.15 | 297.43 | 398.81 | 24.81 | 2182.49 | 524.79 | 3.55 | 3085.45 | 0.00 | 0.00 | 9.44 | 35.99 | 0.00 | 41.58 | 3783.68 | 91.37 | 159.43 | 509.44 | 43.06 | 3.59 | 5.25 | 9.62 | 300.64 | 2.98 | 0.00 | 813.20 | 17.04 | 10.92 | 1053.31 | 5.82 | 46.08 | 25.79 |
| 410975 | **KOMP2** | **G6pd2** | **G6pd2_em1_del** | **-/-** | **F** | **410975** | **6/16/2016** | NA | 7.16 | 100.59 | 18.67 | 285.36 | 1239.02 | 60.15 | 15671.27 | 5880.77 | 2924.99 | 72.57 | 282.17 | 566.43 | 7114.38 | 20.36 | 2.49 | 154.48 | 1803.72 | 17911.63 | 36584.45 | 2612.54 | 367.32 | 303.83 | 19.01 | 2133.58 | 409.06 | 4.24 | 3024.67 | 0.00 | 2.13 | 12.41 | 21.04 | 0.00 | 55.58 | 4114.11 | 73.14 | 149.74 | 655.03 | 38.51 | 3.35 | 5.87 | 7.59 | 101.78 | 4.00 | 2.72 | 781.64 | 23.31 | 7.72 | 1259.78 | 7.17 | 48.68 | 32.33 |
| 410881 | **KOMP2** | **G6pd2** | **G6pd2_em1_del** | **-/-** | **M** | **410881** | **6/16/2016** | NA | 8.56 | 102.64 | 8.41 | 171.21 | 1667.61 | 43.72 | 34585.75 | 7664.14 | 3442.85 | 5376.01 | 0.00 | 410.40 | 6566.63 | 7.48 | 3.52 | 139.36 | 2044.35 | 36374.01 | 46998.80 | 3792.29 | 293.92 | 439.38 | 23.41 | 2234.76 | 537.18 | 3.65 | 2774.77 | 0.00 | 0.00 | 11.21 | 14.08 | 5.82 | 57.32 | 2899.83 | 134.24 | 168.65 | 551.70 | 32.12 | 3.23 | 5.55 | 9.49 | 116.83 | 2.06 | 0.00 | 753.17 | 13.07 | 9.21 | 1793.02 | 7.91 | 38.51 | 24.13 |
| 420416 | **KOMP2** | **G6pd2** | **G6pd2_em1_del** | **-/-** | **M** | **420416** | **8/3/2016** | NA | 9.52 | 76.30 | 8.13 | 177.78 | 1233.23 | 16.01 | 27192.71 | 8195.67 | 3386.64 | 2554.68 | 0.00 | 449.78 | 7131.02 | 0.00 | 2.03 | 117.94 | 2260.06 | 48170.93 | 57463.48 | 3305.54 | 227.93 | 524.69 | 18.88 | 1918.06 | 559.94 | 2.57 | 3502.85 | 4.56 | 1.14 | 9.78 | 21.37 | 4.81 | 51.46 | 3013.61 | 100.96 | 167.72 | 545.91 | 31.38 | 2.50 | 4.67 | 5.42 | 243.09 | 2.88 | 0.00 | 789.33 | 18.90 | 8.38 | 1807.99 | 8.31 | 35.68 | 25.73 |
| 410882 | **KOMP2** | **G6pd2** | **G6pd2_em1_del** | **-/-** | **M** | **410882** | **6/16/2016** | NA | 11.60 | 107.96 | 9.10 | 188.76 | 1478.81 | 61.97 | 27582.33 | 7380.58 | 3470.05 | 5163.05 | 0.00 | 491.62 | 6677.82 | 0.00 | 2.46 | 109.00 | 2305.75 | 33139.30 | 45704.65 | 3447.44 | 277.36 | 448.94 | 24.19 | 2378.33 | 571.25 | 4.63 | 2475.79 | 0.00 | 0.00 | 13.66 | 13.21 | 0.00 | 41.00 | 2261.45 | 116.63 | 158.58 | 575.61 | 27.06 | 3.42 | 5.50 | 8.04 | 81.53 | 3.56 | 0.00 | 773.61 | 11.31 | 11.01 | 1876.10 | 8.79 | 44.81 | 27.38 |
| 39183 | **NorCOMM2** | **Galc** | **Galc_tm1b_A06** | **+/-** | **F** | **39183** | **12/4/2013** | NA | 6.83 | 84.75 | 23.17 | 237.73 | 938.93 | 58.51 | 16424.11 | 6877.40 | 3640.78 | 82.18 | 308.83 | 440.25 | 7023.75 | 13.98 | 2.82 | 113.38 | 2115.71 | 24095.41 | 37093.85 | 2153.15 | 243.58 | 251.28 | 20.70 | 1841.68 | 468.67 | 4.73 | 2904.96 | 2.84 | 2.21 | 10.83 | 35.11 | 4.79 | 69.06 | 4201.76 | 75.81 | 141.81 | 432.53 | 38.90 | 3.09 | 5.54 | 12.20 | 130.15 | 3.71 | 3.29 | 750.85 | 18.09 | 6.92 | 1042.58 | 7.40 | 33.34 | 26.90 |
| 40391 | **NorCOMM2** | **Galc** | **Galc_tm1b_A06** | **+/-** | **F** | **40391** | **12/16/2013** | NA | 6.99 | 83.84 | 22.68 | 249.10 | 1173.79 | 76.25 | 15027.17 | 6964.18 | 3396.95 | 58.94 | 213.38 | 471.22 | 7900.41 | 26.12 | 3.54 | 120.64 | 2018.79 | 29072.20 | 34683.67 | 3451.62 | 251.92 | 205.40 | 23.86 | 2128.83 | 490.07 | 3.66 | 2455.46 | 9.91 | 6.80 | 8.84 | 63.36 | 17.45 | 107.00 | 4289.33 | 78.39 | 168.86 | 627.76 | 38.24 | 3.40 | 7.34 | 10.99 | 124.09 | 4.50 | 2.92 | 876.44 | 23.66 | 8.66 | 1408.94 | 6.77 | 31.71 | 28.12 |
| 39181 | **NorCOMM2** | **Galc** | **Galc_tm1b_A06** | **+/-** | **F** | **39181** | **12/4/2013** | NA | 8.01 | 86.83 | 22.73 | 281.07 | 997.13 | 68.26 | 16256.52 | 6957.49 | 3353.70 | 55.34 | 182.87 | 386.19 | 8222.14 | 28.48 | 3.19 | 114.86 | 2313.70 | 24683.84 | 40008.16 | 3096.18 | 249.49 | 295.68 | 22.39 | 2335.86 | 463.99 | 3.31 | 2715.93 | 0.00 | 3.08 | 11.33 | 56.31 | 7.65 | 82.18 | 3857.84 | 78.24 | 162.49 | 557.14 | 48.03 | 3.43 | 7.35 | 9.15 | 111.32 | 3.84 | 3.08 | 866.33 | 24.60 | 10.37 | 1274.44 | 9.37 | 35.25 | 23.95 |
| 43707 | **NorCOMM2** | **Galc** | **Galc_tm1b_A06** | **+/-** | **M** | **43707** | **12/30/2013** | NA | 8.95 | 117.01 | 11.51 | 203.14 | 1567.96 | 50.73 | 29183.25 | 8751.65 | 4156.84 | 4456.66 | 0.00 | 493.02 | 8028.04 | 0.00 | 2.99 | 136.32 | 2014.31 | 35708.28 | 47563.28 | 3305.69 | 227.36 | 474.48 | 23.05 | 2356.18 | 543.10 | 7.06 | 2752.21 | 6.74 | 2.54 | 12.57 | 14.89 | 6.66 | 68.79 | 3231.14 | 132.59 | 164.60 | 569.00 | 29.73 | 3.84 | 6.83 | 9.48 | 99.17 | 3.63 | 3.35 | 853.59 | 16.98 | 8.11 | 1939.59 | 9.29 | 35.55 | 31.05 |
| 40288 | **NorCOMM2** | **Galc** | **Galc_tm1b_A06** | **+/-** | **M** | **40288** | **12/16/2013** | NA | 5.90 | 102.15 | 13.19 | 192.32 | 1052.56 | 35.47 | 24615.09 | 9582.04 | 4074.69 | 3139.78 | 0.00 | 446.49 | 9280.68 | 7.50 | 2.16 | 111.50 | 1818.79 | 29552.46 | 46086.39 | 3857.95 | 300.83 | 579.56 | 16.57 | 1978.33 | 508.24 | 2.53 | 2983.18 | 2.96 | 3.06 | 13.36 | 21.98 | 7.89 | 51.82 | 2250.97 | 133.96 | 177.62 | 538.28 | 42.23 | 2.11 | 8.01 | 5.99 | 141.91 | 4.09 | 3.64 | 898.14 | 14.38 | 10.26 | 2194.00 | 7.77 | 34.63 | 25.27 |
| 43708 | **NorCOMM2** | **Galc** | **Galc_tm1b_A06** | **+/-** | **M** | **43708** | **12/30/2013** | NA | 6.46 | 146.69 | 12.48 | 204.24 | 1462.07 | 30.48 | 37243.77 | 9258.03 | 3962.33 | 5984.35 | 0.00 | 522.00 | 9086.49 | 0.00 | 3.20 | 107.83 | 2363.05 | 41921.42 | 50541.72 | 3226.84 | 213.91 | 439.45 | 18.66 | 2403.54 | 678.98 | 4.84 | 2731.45 | 4.09 | 2.40 | 10.83 | 50.91 | 4.67 | 60.24 | 2524.73 | 146.49 | 182.60 | 737.38 | 26.67 | 3.47 | 7.50 | 9.59 | 87.69 | 4.12 | 5.09 | 953.78 | 18.24 | 9.66 | 2338.36 | 10.54 | 36.54 | 29.60 |
| 38377 | **NorCOMM2** | **Gnpda1** | **Gnpda1_tm1b_A05** | **+/-** | **F** | **38377** | **12/11/2013** | NA | 9.14 | 84.09 | 25.37 | 339.72 | 1010.60 | 63.70 | 13851.00 | 6173.50 | 3576.30 | 108.17 | 330.46 | 521.76 | 8684.20 | 14.07 | 2.12 | 117.18 | 2029.30 | 20923.00 | 34004.00 | 2873.30 | 295.08 | 257.63 | 22.32 | 1222.00 | 486.81 | 0.00 | 2334.50 | 0.00 | 0.00 | 10.87 | 181.53 | 5.60 | 52.42 | 3374.50 | 81.72 | 163.57 | 558.62 | 44.40 | 2.93 | 7.58 | 9.47 | 153.44 | 4.76 | 3.72 | 741.98 | 17.48 | 8.67 | 1206.50 | 10.50 | 30.33 | 23.98 |
| 37870 | **NorCOMM2** | **Gnpda1** | **Gnpda1_tm1b_A05** | **+/-** | **F** | **37870** | **11/28/2013** | NA | 6.83 | 108.79 | 19.27 | 305.70 | 971.11 | 51.48 | 13516.00 | 5932.00 | 3261.90 | 128.48 | 13.51 | 500.00 | 8387.10 | 22.85 | 3.59 | 106.73 | 2048.60 | 23501.00 | 37087.00 | 2389.40 | 294.18 | 239.47 | 21.01 | 2249.30 | 466.20 | 6.61 | 2341.70 | 0.00 | 6.85 | 10.82 | 75.56 | 17.21 | 129.00 | 3987.80 | 70.73 | 172.17 | 467.07 | 42.97 | 2.93 | 6.50 | 7.41 | 74.26 | 4.17 | 2.36 | 856.67 | 23.72 | 14.53 | 989.48 | 8.22 | 42.65 | 22.99 |
| 37871 | **NorCOMM2** | **Gnpda1** | **Gnpda1_tm1b_A05** | **+/-** | **F** | **37871** | **11/28/2013** | NA | 8.38 | 82.29 | 19.61 | 236.68 | 950.53 | 47.07 | 11175.00 | 5372.70 | 2840.90 | 224.47 | 23.54 | 394.29 | 7529.30 | 22.65 | 2.26 | 130.78 | 1598.10 | 27980.00 | 35157.00 | 2610.10 | 254.18 | 291.04 | 34.58 | 1784.10 | 441.06 | 4.51 | 2716.80 | 4.70 | 3.39 | 9.91 | 62.62 | 7.94 | 83.84 | 3643.60 | 85.81 | 163.32 | 627.59 | 34.30 | 3.90 | 5.94 | 8.86 | 151.19 | 4.61 | 2.68 | 856.00 | 19.66 | 12.13 | 863.79 | 7.98 | 46.31 | 22.17 |
| 38275 | **NorCOMM2** | **Gnpda1** | **Gnpda1_tm1b_A05** | **+/-** | **M** | **38275** | **12/11/2013** | NA | 12.41 | 85.87 | 7.88 | 185.97 | 3135.60 | 571.62 | 26460.00 | 8166.90 | 4035.40 | 4624.00 | 0.00 | 549.98 | 9706.50 | 6.97 | 3.04 | 118.63 | 1899.60 | 35819.00 | 42160.00 | 4363.80 | 294.30 | 496.17 | 22.26 | 1629.70 | 585.90 | 6.76 | 3091.10 | 0.00 | 0.00 | 13.80 | 17.00 | 9.71 | 68.48 | 2988.60 | 169.89 | 204.68 | 687.89 | 23.65 | 3.18 | 7.34 | 7.94 | 116.93 | 3.93 | 3.16 | 1106.40 | 16.37 | 7.32 | 2324.00 | 12.28 | 44.99 | 35.99 |
| 37768 | **NorCOMM2** | **Gnpda1** | **Gnpda1_tm1b_A05** | **+/-** | **M** | **37768** | **11/27/2013** | NA | 11.77 | 153.44 | 9.95 | 174.67 | 1983.70 | 120.13 | 31164.00 | 8213.30 | 4047.60 | 5611.60 | 0.00 | 683.50 | 9437.20 | 0.00 | 5.54 | 115.49 | 2037.90 | 40662.00 | 40347.00 | 3582.50 | 303.05 | 637.41 | 22.47 | 2282.00 | 686.94 | 0.00 | 2371.10 | 0.00 | 2.93 | 15.52 | 13.30 | 10.22 | 94.78 | 1880.90 | 169.82 | 197.60 | 705.90 | 26.70 | 4.41 | 7.61 | 9.97 | 166.87 | 5.17 | 4.40 | 1055.70 | 16.05 | 13.77 | 2121.30 | 12.92 | 46.12 | 20.45 |
| 37769 | **NorCOMM2** | **Gnpda1** | **Gnpda1_tm1b_A05** | **+/-** | **M** | **37769** | **11/28/2013** | NA | 13.01 | 149.44 | 12.28 | 234.06 | 1649.50 | 36.02 | 28352.00 | 9196.00 | 5099.10 | 4618.30 | 0.00 | 502.68 | 12455.00 | 6.68 | 7.01 | 118.68 | 1649.30 | 36821.00 | 47841.00 | 2996.90 | 267.00 | 474.92 | 17.32 | 1666.00 | 562.74 | 7.62 | 2839.30 | 3.22 | 8.80 | 12.13 | 32.21 | 21.80 | 167.46 | 2239.40 | 145.06 | 226.72 | 732.80 | 30.63 | 5.18 | 9.03 | 8.40 | 106.82 | 4.11 | 3.51 | 1128.10 | 18.60 | 11.12 | 2090.90 | 11.58 | 43.32 | 30.99 |
| 116005 | **NorCOMM2** | **Idh1** | **Idh1_tm1b_H02** | **-/-** | **F** | **116005** | **6/5/2014** | NA | 5.01 | 101.26 | 25.95 | 288.81 | 916.18 | 64.63 | 16316.73 | 7094.49 | 3536.24 | 159.80 | 61.74 | 425.37 | 7159.53 | 25.83 | 5.02 | 121.99 | 2164.62 | 26541.08 | 39329.41 | 3348.31 | 300.87 | 497.53 | 17.94 | 3437.52 | 441.49 | 6.80 | 2676.15 | 8.01 | 3.84 | 15.22 | 29.23 | 11.12 | 62.17 | 4158.13 | 90.06 | 164.29 | 614.51 | 37.86 | 4.06 | 6.92 | 9.01 | 70.86 | 3.85 | 0.00 | 849.65 | 19.50 | 7.96 | 1165.61 | 8.03 | 40.71 | 26.67 |
| 116097 | **NorCOMM2** | **Idh1** | **Idh1_tm1b_H02** | **-/-** | **F** | **116097** | **6/5/2014** | NA | 8.50 | 132.65 | 28.78 | 307.35 | 1238.37 | 52.45 | 15874.16 | 8500.13 | 3475.14 | 326.89 | 931.94 | 491.61 | 8280.65 | 16.70 | 3.81 | 136.24 | 2030.77 | 40586.63 | 42037.53 | 2517.47 | 319.30 | 374.66 | 23.60 | 2543.43 | 599.07 | 2.96 | 3036.33 | 5.09 | 2.05 | 12.83 | 222.51 | 4.00 | 66.61 | 4809.24 | 81.90 | 192.38 | 601.63 | 43.54 | 5.15 | 5.92 | 12.69 | 110.09 | 6.30 | 4.96 | 1019.66 | 27.94 | 8.53 | 840.49 | 9.39 | 42.08 | 34.59 |
| 116002 | **NorCOMM2** | **Idh1** | **Idh1_tm1b_H02** | **-/-** | **F** | **116002** | **6/5/2014** | NA | 3.36 | 100.25 | 25.17 | 301.17 | 943.00 | 43.86 | 11614.09 | 6666.58 | 3433.63 | 81.10 | 117.86 | 390.44 | 6894.04 | 30.07 | 3.04 | 111.86 | 2116.49 | 25892.07 | 38257.01 | 3128.92 | 370.78 | 349.84 | 22.64 | 2750.79 | 492.43 | 6.35 | 2555.37 | 4.09 | 1.65 | 11.94 | 53.79 | 6.18 | 54.44 | 4126.82 | 92.90 | 156.36 | 521.55 | 42.50 | 3.68 | 6.25 | 11.61 | 58.20 | 4.16 | 2.99 | 785.91 | 21.46 | 9.22 | 1239.60 | 8.42 | 33.47 | 18.39 |
| 172743 | **NorCOMM2** | **Idh1** | **Idh1_tm1b_H02** | **-/-** | **M** | **172743** | **10/16/2014** | NA | 9.32 | 69.12 | 9.68 | 225.59 | 1628.33 | 27.20 | 33374.08 | 9909.10 | 3491.93 | 5794.49 | 0.00 | 415.37 | 8209.83 | 7.00 | 1.91 | 120.75 | 1850.91 | 31313.43 | 40191.66 | 3225.84 | 340.99 | 405.09 | 17.68 | 2232.94 | 511.24 | 5.63 | 2316.90 | 5.39 | 0.86 | 12.01 | 17.61 | 0.00 | 44.70 | 2750.50 | 131.40 | 176.97 | 639.88 | 29.09 | 4.01 | 7.50 | 10.93 | 156.54 | 3.65 | 3.90 | 831.17 | 16.63 | 8.23 | 1834.69 | 8.76 | 28.89 | 21.20 |
| 172742 | **NorCOMM2** | **Idh1** | **Idh1_tm1b_H02** | **-/-** | **M** | **172742** | **10/16/2014** | NA | 10.40 | 110.90 | 13.25 | 201.12 | 1383.56 | 23.17 | 31818.58 | 7830.93 | 3471.95 | 5447.94 | 0.00 | 466.30 | 9065.47 | 0.00 | 2.27 | 136.98 | 1747.27 | 42931.71 | 40168.97 | 3336.43 | 286.78 | 406.40 | 19.42 | 1793.57 | 539.28 | 4.04 | 2284.41 | 7.52 | 1.58 | 12.32 | 22.57 | 3.50 | 47.85 | 2359.03 | 118.79 | 177.13 | 647.22 | 29.28 | 4.38 | 6.06 | 8.58 | 67.85 | 3.96 | 4.57 | 863.39 | 14.81 | 6.78 | 2173.26 | 12.31 | 37.29 | 32.95 |
| 172741 | **NorCOMM2** | **Idh1** | **Idh1_tm1b_H02** | **-/-** | **M** | **172741** | **10/16/2014** | NA | 8.89 | 75.00 | 12.17 | 234.71 | 1142.51 | 20.26 | 25230.27 | 7628.62 | 3496.28 | 4011.95 | 0.00 | 421.24 | 9459.53 | 5.76 | 1.61 | 128.39 | 1998.08 | 34986.80 | 48742.22 | 3358.99 | 311.76 | 406.77 | 15.64 | 1675.30 | 496.88 | 6.42 | 2542.42 | 4.09 | 0.00 | 9.86 | 18.07 | 0.00 | 55.82 | 2326.66 | 92.55 | 172.56 | 477.70 | 33.68 | 3.56 | 7.01 | 11.54 | 72.45 | 3.44 | 0.00 | 760.28 | 14.88 | 7.75 | 2010.87 | 8.58 | 47.14 | 22.49 |
| 435700 | **KOMP2** | **Iqgap1** | **Iqgap1_tm1b_A11** | **-/-** | **F** | **435700** | **9/8/2016** | NA | 10.35 | 89.02 | 18.73 | 219.28 | 956.47 | 57.03 | 18874.88 | 5607.46 | 2532.49 | 254.39 | 238.94 | 386.97 | 6458.80 | 17.25 | 3.08 | 125.12 | 1465.52 | 26846.74 | 39473.82 | 3122.85 | 295.77 | 411.37 | 27.73 | 2372.97 | 561.87 | 3.35 | 2319.88 | 0.00 | 3.02 | 12.83 | 28.29 | 6.75 | 52.46 | 3843.82 | 83.82 | 148.31 | 402.51 | 38.17 | 3.76 | 3.32 | 12.64 | 55.77 | 4.20 | 0.00 | 719.24 | 19.84 | 9.40 | 905.99 | 5.19 | 44.14 | 33.97 |
| 506105 | **KOMP2** | **Iqgap1** | **Iqgap1_tm1b_A11** | **-/-** | **F** | **506105** | **3/23/2017** | NA | 3.55 | 88.69 | 24.82 | 258.98 | 1076.75 | 46.02 | 17184.77 | 8050.31 | 3031.04 | 56.15 | 28.83 | 407.68 | 6780.46 | 17.41 | 2.86 | 118.33 | 1709.24 | 28600.00 | 42999.89 | 3932.64 | 322.39 | 676.78 | 22.27 | 2198.58 | 460.21 | 2.35 | 2679.95 | 0.00 | 7.02 | 10.23 | 12.95 | 12.18 | 143.32 | 3526.51 | 70.13 | 140.64 | 497.11 | 38.27 | 2.87 | 4.08 | 8.53 | 32.77 | 3.27 | 0.00 | 622.68 | 28.27 | 10.01 | 928.99 | 6.10 | 30.47 | 29.87 |
| 435799 | **KOMP2** | **Iqgap1** | **Iqgap1_tm1b_A11** | **-/-** | **F** | **435799** | **9/8/2016** | NA | 10.37 | 100.83 | 17.44 | 258.62 | 1061.16 | 49.34 | 16530.77 | 6348.37 | 3012.02 | 90.47 | 545.32 | 344.53 | 6734.27 | 32.85 | 2.40 | 114.59 | 1990.77 | 23169.42 | 39576.48 | 3090.05 | 376.31 | 417.36 | 20.68 | 2349.62 | 472.51 | 5.20 | 2593.27 | 6.14 | 0.00 | 11.05 | 423.48 | 0.00 | 44.55 | 3578.01 | 82.16 | 149.21 | 496.92 | 39.81 | 3.64 | 5.20 | 9.40 | 44.97 | 5.86 | 0.00 | 809.82 | 24.24 | 7.48 | 879.65 | 6.96 | 35.02 | 28.88 |
| 435695 | **KOMP2** | **Iqgap1** | **Iqgap1_tm1b_A11** | **-/-** | **M** | **435695** | **9/8/2016** | NA | 11.11 | 108.88 | 8.97 | 209.93 | 1406.55 | 39.31 | 33955.30 | 9013.85 | 3567.06 | 4840.90 | 0.00 | 473.06 | 8071.98 | 0.00 | 2.73 | 149.75 | 2448.70 | 40852.67 | 52563.31 | 4897.38 | 338.75 | 517.91 | 17.63 | 2580.80 | 635.11 | 6.91 | 3159.59 | 0.00 | 0.00 | 13.08 | 14.63 | 4.60 | 63.29 | 3255.20 | 129.04 | 177.35 | 598.18 | 33.26 | 3.09 | 4.78 | 8.10 | 55.24 | 4.14 | 0.00 | 813.03 | 16.28 | 9.84 | 2259.66 | 6.13 | 40.07 | 31.58 |
| 487462 | **KOMP2** | **Iqgap1** | **Iqgap1_tm1b_A11** | **-/-** | **M** | **487462** | **1/4/2017** | NA | 5.73 | 157.47 | 11.36 | 183.96 | 1251.09 | 19.63 | 27899.16 | 7537.83 | 3019.63 | 4940.75 | 0.00 | 278.21 | 7965.52 | 7.77 | 4.03 | 118.40 | 1894.37 | 38370.81 | 25021.08 | 3307.09 | 249.84 | 264.62 | 9.19 | 1369.23 | 450.39 | 3.63 | 2188.40 | 4.39 | 0.00 | 14.93 | 10.40 | 3.43 | 50.58 | 3431.56 | 112.91 | 151.41 | 547.55 | 32.28 | 4.20 | 6.31 | 11.90 | 64.35 | 2.85 | 0.00 | 746.38 | 18.52 | 13.47 | 1667.01 | 8.50 | 35.35 | 16.38 |
| 435693 | **KOMP2** | **Iqgap1** | **Iqgap1_tm1b_A11** | **-/-** | **M** | **435693** | **9/8/2016** | NA | 10.97 | 84.66 | 10.35 | 192.11 | 1117.31 | 40.30 | 30180.11 | 8251.08 | 3511.42 | 3372.52 | 0.00 | 388.16 | 8931.21 | 0.00 | 2.92 | 141.07 | 2171.64 | 35496.87 | 52213.86 | 4907.89 | 306.73 | 751.40 | 18.84 | 1940.89 | 628.81 | 4.00 | 2790.02 | 0.00 | 0.00 | 12.09 | 17.52 | 0.00 | 87.11 | 3304.86 | 120.92 | 170.07 | 587.28 | 29.25 | 2.96 | 4.86 | 6.41 | 123.43 | 4.02 | 0.00 | 844.31 | 16.07 | 10.12 | 2071.06 | 9.34 | 28.85 | 24.99 |
| 107190 | **NorCOMM2** | **Lmbrd1** | **Lmbrd1_tm1b_A10** | **+/-** | **F** | **107190** | **5/22/2014** | NA | 4.66 | 104.80 | 26.15 | 268.34 | 1407.33 | 73.89 | 15940.40 | 7315.43 | 3885.68 | 46.96 | 813.13 | 495.46 | 6755.14 | 18.88 | 4.07 | 107.78 | 2080.23 | 23690.86 | 40023.95 | 2831.28 | 254.95 | 287.29 | 21.12 | 2259.21 | 495.04 | 4.39 | 2622.39 | 4.06 | 4.17 | 17.10 | 45.12 | 10.94 | 103.79 | 3813.04 | 83.88 | 167.02 | 529.37 | 42.84 | 4.20 | 6.55 | 8.18 | 166.69 | 3.52 | 3.44 | 853.13 | 30.53 | 10.31 | 1205.98 | 7.94 | 32.33 | 26.40 |
| 111493 | **NorCOMM2** | **Lmbrd1** | **Lmbrd1_tm1b_A10** | **+/-** | **F** | **111493** | **5/27/2014** | NA | 5.27 | 91.74 | 19.82 | 387.42 | 1428.68 | 82.54 | 14069.78 | 5963.26 | 3039.88 | 166.19 | 276.13 | 437.08 | 7798.91 | 31.47 | 3.66 | 105.68 | 1810.40 | 34231.79 | 37125.42 | 2519.97 | 388.39 | 431.23 | 30.54 | 3040.21 | 518.54 | 3.03 | 2627.63 | 7.51 | 2.69 | 10.97 | 65.50 | 6.20 | 78.27 | 4461.61 | 91.31 | 155.17 | 502.18 | 41.58 | 4.09 | 5.32 | 12.11 | 290.86 | 4.31 | 3.55 | 887.43 | 22.08 | 12.66 | 1087.69 | 8.49 | 40.16 | 31.07 |
| 107189 | **NorCOMM2** | **Lmbrd1** | **Lmbrd1_tm1b_A10** | **+/-** | **F** | **107189** | **5/21/2014** | NA | 2.76 | 107.01 | 26.15 | 321.22 | 1303.74 | 79.56 | 16870.69 | 8304.80 | 3823.71 | 89.30 | 410.28 | 591.10 | 6839.32 | 14.91 | 2.46 | 132.82 | 2260.53 | 23511.30 | 42278.25 | 2278.22 | 387.02 | 369.88 | 22.20 | 2487.76 | 489.53 | 3.48 | 2518.47 | 0.00 | 2.03 | 13.51 | 87.06 | 5.35 | 63.63 | 5172.27 | 91.05 | 178.97 | 697.59 | 43.02 | 3.76 | 6.67 | 11.88 | 112.91 | 4.25 | 3.23 | 793.96 | 30.19 | 8.68 | 1239.40 | 8.36 | 37.77 | 23.74 |
| 72464 | **NorCOMM2** | **Lmbrd1** | **Lmbrd1_tm1b_A10** | **+/-** | **M** | **72464** | **3/12/2014** | NA | 8.21 | 67.72 | 13.96 | 210.00 | 1310.26 | 32.26 | 24409.36 | 7395.12 | 2778.85 | 4083.62 | 0.00 | 529.29 | 8333.29 | 6.97 | 2.19 | 107.44 | 1674.86 | 37617.43 | 41989.18 | 2810.32 | 274.97 | 308.00 | 23.97 | 1597.46 | 461.43 | 5.79 | 2735.56 | 0.00 | 2.40 | 9.55 | 17.23 | 6.20 | 71.47 | 2476.75 | 115.56 | 166.63 | 457.00 | 31.14 | 3.64 | 5.70 | 4.61 | 96.99 | 2.71 | 4.73 | 775.76 | 11.82 | 8.42 | 1825.63 | 8.90 | 31.42 | 19.57 |
| 72463 | **NorCOMM2** | **Lmbrd1** | **Lmbrd1_tm1b_A10** | **+/-** | **M** | **72463** | **3/12/2014** | NA | 6.66 | 136.41 | 8.60 | 156.34 | 2516.44 | 504.91 | 27696.99 | 7888.44 | 2748.64 | 4933.72 | 0.00 | 578.63 | 7199.48 | 7.17 | 3.32 | 99.14 | 1871.95 | 28552.99 | 33929.94 | 2891.47 | 307.04 | 314.32 | 23.26 | 2600.08 | 514.06 | 5.13 | 2685.61 | 3.23 | 2.77 | 16.94 | 39.14 | 9.66 | 75.69 | 2728.96 | 186.64 | 173.16 | 599.87 | 26.21 | 4.51 | 7.64 | 9.82 | 70.58 | 3.57 | 4.81 | 1163.80 | 12.29 | 12.18 | 2436.42 | 13.42 | 38.05 | 30.71 |
| 72460 | **NorCOMM2** | **Lmbrd1** | **Lmbrd1_tm1b_A10** | **+/-** | **M** | **72460** | **3/12/2014** | NA | 12.54 | 78.93 | 9.31 | 195.50 | 1325.60 | 25.08 | 21460.93 | 7678.43 | 3536.34 | 3990.90 | 0.00 | 465.28 | 8452.69 | 6.34 | 4.16 | 104.44 | 1962.94 | 42328.13 | 43041.85 | 2638.54 | 260.38 | 418.39 | 18.31 | 1996.28 | 555.70 | 4.91 | 2641.61 | 6.21 | 6.23 | 10.80 | 22.33 | 23.96 | 146.36 | 3318.82 | 125.04 | 176.62 | 577.56 | 26.94 | 3.45 | 6.50 | 7.88 | 84.47 | 2.86 | 4.54 | 864.77 | 20.64 | 5.09 | 1903.05 | 9.39 | 34.12 | 31.00 |
| 213490 | **NorCOMM2** | **Mfap4** | **Mfap4_tm1b_C11** | **-/-** | **F** | **213490** | **1/20/2015** | NA | 7.21 | 77.86 | 16.75 | 313.92 | 1110.92 | 40.83 | 14446.11 | 6520.78 | 2664.95 | 313.58 | 40.13 | 421.53 | 6260.24 | 30.85 | 2.01 | 137.79 | 2201.44 | 30396.17 | 35166.10 | 2467.66 | 428.30 | 279.06 | 39.17 | 2030.77 | 419.73 | 5.77 | 2672.24 | 0.00 | 0.00 | 10.01 | 64.28 | 0.00 | 43.07 | 4819.01 | 82.79 | 152.46 | 595.55 | 33.49 | 4.28 | 5.13 | 10.79 | 118.69 | 4.01 | 2.93 | 810.08 | 18.26 | 8.70 | 1245.54 | 5.95 | 35.35 | 27.71 |
| 213491 | **NorCOMM2** | **Mfap4** | **Mfap4_tm1b_C11** | **-/-** | **F** | **213491** | **1/21/2015** | NA | 8.74 | 81.42 | 23.96 | 287.87 | 1271.76 | 65.22 | 14289.56 | 7309.86 | 3456.22 | 154.69 | 49.77 | 643.86 | 7234.42 | 20.19 | 1.68 | 132.22 | 2780.17 | 23943.97 | 46039.03 | 3208.27 | 240.21 | 319.08 | 25.37 | 3036.64 | 481.24 | 2.01 | 2619.92 | 0.00 | 0.00 | 11.83 | 26.59 | 0.00 | 38.45 | 4733.88 | 88.61 | 181.55 | 549.74 | 50.41 | 3.37 | 5.89 | 8.48 | 70.92 | 3.12 | 4.16 | 829.61 | 23.40 | 11.46 | 1373.20 | 7.49 | 39.98 | 29.29 |
| 213482 | **NorCOMM2** | **Mfap4** | **Mfap4_tm1b_C11** | **-/-** | **F** | **213482** | **1/20/2015** | NA | 8.15 | 79.11 | 14.62 | 289.68 | 1012.95 | 49.67 | 14590.62 | 7052.57 | 2774.12 | 380.09 | 418.20 | 510.01 | 6611.13 | 26.13 | 2.51 | 126.98 | 2383.87 | 25092.71 | 34150.93 | 3143.59 | 382.59 | 349.90 | 35.13 | 2832.42 | 450.72 | 5.18 | 2690.74 | 0.00 | 0.00 | 11.66 | 42.37 | 3.56 | 44.70 | 3972.69 | 84.66 | 153.38 | 541.57 | 35.26 | 3.81 | 4.36 | 9.65 | 57.93 | 3.95 | 3.45 | 884.84 | 22.71 | 15.59 | 1166.41 | 7.77 | 45.71 | 25.89 |
| 193352 | **NorCOMM2** | **Mfap4** | **Mfap4_tm1b_C11** | **-/-** | **M** | **193352** | **12/11/2014** | NA | 16.95 | 115.47 | 10.83 | 204.58 | 1675.66 | 93.67 | 29011.65 | 8884.09 | 3722.74 | 5548.36 | 0.00 | 417.06 | 7617.41 | 0.00 | 2.90 | 141.22 | 2329.92 | 39797.01 | 47885.88 | 4426.38 | 237.39 | 535.94 | 16.24 | 1773.35 | 510.50 | 4.76 | 2339.30 | 5.24 | 2.97 | 15.17 | 33.33 | 6.59 | 66.28 | 2443.95 | 140.05 | 185.66 | 661.36 | 32.72 | 3.69 | 9.14 | 7.47 | 126.28 | 4.01 | 5.74 | 939.74 | 14.67 | 9.09 | 2619.85 | 9.37 | 39.24 | 29.08 |
| 193349 | **NorCOMM2** | **Mfap4** | **Mfap4_tm1b_C11** | **-/-** | **M** | **193349** | **12/11/2014** | NA | 14.56 | 119.74 | 10.85 | 196.53 | 1736.32 | 54.60 | 25332.63 | 8581.84 | 3845.14 | 5222.86 | 0.00 | 397.95 | 7930.52 | 0.00 | 3.43 | 132.39 | 2117.84 | 41658.57 | 50380.91 | 3429.06 | 269.05 | 454.13 | 20.66 | 2178.12 | 625.37 | 5.30 | 2382.89 | 7.85 | 4.05 | 13.90 | 46.13 | 10.17 | 70.52 | 2966.20 | 139.84 | 193.43 | 614.88 | 28.60 | 4.00 | 7.98 | 9.55 | 85.38 | 3.69 | 5.96 | 871.31 | 19.28 | 9.12 | 2481.83 | 10.27 | 36.10 | 26.34 |
| 193353 | **NorCOMM2** | **Mfap4** | **Mfap4_tm1b_C11** | **-/-** | **M** | **193353** | **12/11/2014** | NA | 6.93 | 90.14 | 11.81 | 234.33 | 1115.73 | 24.90 | 28153.61 | 8734.84 | 3848.13 | 3017.07 | 0.00 | 377.65 | 9214.31 | 6.09 | 2.65 | 112.12 | 2395.17 | 42466.59 | 57586.77 | 4734.31 | 245.49 | 612.26 | 22.48 | 2061.69 | 510.41 | 6.26 | 2818.00 | 0.00 | 2.44 | 13.99 | 19.33 | 6.58 | 51.99 | 3010.14 | 125.45 | 188.72 | 574.01 | 38.19 | 3.04 | 5.24 | 6.72 | 120.85 | 3.65 | 4.85 | 862.91 | 16.84 | 8.72 | 1668.58 | 10.66 | 32.84 | 27.33 |
| 356364 | **NorCOMM2** | **Mmachc** | **Mmachc_tm1.1_A10** | **+/-** | **F** | **356364** | **11/17/2015** | NA | 4.87 | 89.73 | 20.08 | 267.66 | 888.50 | 67.04 | 15279.06 | 6279.99 | 2974.33 | 136.11 | 90.42 | 425.50 | 6742.36 | 24.59 | 2.35 | 110.49 | 2178.95 | 28671.98 | 38665.45 | 2816.11 | 248.17 | 422.37 | 29.27 | 1625.78 | 409.16 | 3.07 | 2390.80 | 0.00 | 0.00 | 10.19 | 21.37 | 4.07 | 49.27 | 4432.84 | 77.90 | 156.91 | 596.12 | 37.43 | 3.04 | 5.25 | 6.21 | 67.37 | 2.81 | 4.26 | 895.23 | 19.46 | 10.61 | 966.21 | 7.20 | 35.69 | 24.55 |
| 356363 | **NorCOMM2** | **Mmachc** | **Mmachc_tm1.1_A10** | **+/-** | **F** | **356363** | **11/17/2015** | NA | 6.82 | 132.52 | 22.95 | 258.93 | 1140.60 | 53.90 | 16005.63 | 7077.89 | 3234.87 | 121.36 | 213.71 | 461.77 | 7769.84 | 19.41 | 3.19 | 120.82 | 2497.35 | 23168.41 | 41319.31 | 3333.94 | 378.51 | 384.26 | 27.70 | 3472.00 | 428.96 | 3.65 | 2329.33 | 0.00 | 0.00 | 10.67 | 24.87 | 3.03 | 45.94 | 4163.33 | 84.29 | 170.24 | 483.01 | 48.29 | 3.42 | 5.91 | 9.14 | 80.10 | 2.79 | 4.12 | 751.39 | 20.98 | 10.54 | 1131.28 | 7.61 | 32.52 | 25.01 |
| 356370 | **NorCOMM2** | **Mmachc** | **Mmachc_tm1.1_A10** | **+/-** | **F** | **356370** | **11/19/2015** | NA | 5.98 | 105.33 | 29.78 | 260.22 | 1916.01 | 50.83 | 15121.50 | 6289.71 | 3590.78 | 58.59 | 743.51 | 401.46 | 7942.17 | 16.01 | 1.94 | 125.63 | 2724.21 | 29330.72 | 42960.95 | 2779.68 | 277.58 | 418.75 | 29.81 | 3158.16 | 442.00 | 3.57 | 2657.03 | 0.00 | 0.00 | 9.75 | 40.33 | 2.83 | 47.19 | 4099.01 | 87.23 | 168.07 | 553.55 | 47.14 | 3.40 | 6.62 | 12.05 | 130.32 | 3.06 | 3.14 | 935.44 | 38.31 | 12.58 | 1030.40 | 8.59 | 47.34 | 23.29 |
| 356155 | **NorCOMM2** | **Mmachc** | **Mmachc_tm1.1_A10** | **+/-** | **M** | **356155** | **11/17/2015** | NA | 11.44 | 213.99 | 6.96 | 127.96 | 2145.33 | 403.82 | 28934.37 | 8521.29 | 3565.10 | 6113.72 | 0.00 | 716.34 | 7857.05 | 0.00 | 5.92 | 160.61 | 2081.24 | 32745.57 | 42303.90 | 4316.59 | 425.55 | 433.60 | 29.87 | 2422.67 | 598.40 | 8.88 | 3162.76 | 7.04 | 0.93 | 26.84 | 12.56 | 3.54 | 50.57 | 2307.23 | 184.85 | 187.30 | 536.74 | 29.33 | 3.01 | 9.36 | 10.02 | 57.84 | 3.67 | 8.34 | 1065.69 | 16.16 | 13.99 | 2550.99 | 11.04 | 49.70 | 29.78 |
| 356156 | **NorCOMM2** | **Mmachc** | **Mmachc_tm1.1_A10** | **+/-** | **M** | **356156** | **11/17/2015** | NA | 9.84 | 208.66 | 10.36 | 164.36 | 2480.54 | 432.24 | 25762.15 | 8459.93 | 2905.41 | 5787.82 | 0.00 | 494.63 | 7432.40 | 6.43 | 3.61 | 123.18 | 1817.90 | 31596.83 | 36365.77 | 4015.86 | 313.45 | 413.71 | 26.68 | 2683.55 | 549.43 | 4.66 | 2595.25 | 0.00 | 0.00 | 17.12 | 12.85 | 2.48 | 50.61 | 2507.64 | 167.11 | 177.76 | 598.78 | 28.81 | 5.26 | 9.00 | 6.76 | 81.68 | 4.91 | 6.60 | 1081.80 | 14.61 | 14.71 | 2217.45 | 9.96 | 36.92 | 29.66 |
| 356153 | **NorCOMM2** | **Mmachc** | **Mmachc_tm1.1_A10** | **+/-** | **M** | **356153** | **11/17/2015** | NA | 9.41 | 96.00 | 10.31 | 173.97 | 2216.93 | 57.27 | 28805.92 | 7614.04 | 3686.83 | 6612.75 | 0.00 | 571.36 | 8674.15 | 6.39 | 2.16 | 141.04 | 2457.06 | 34391.88 | 50252.65 | 4004.98 | 264.40 | 444.00 | 32.63 | 2886.15 | 665.60 | 5.63 | 3007.24 | 3.40 | 0.00 | 9.80 | 15.04 | 4.23 | 56.13 | 3138.79 | 156.91 | 189.74 | 543.74 | 31.54 | 4.31 | 7.80 | 7.25 | 78.36 | 3.93 | 5.51 | 984.28 | 17.93 | 10.94 | 2608.98 | 11.66 | 49.40 | 31.29 |
| 328106 | **NorCOMM2** | **Mvk** | **Mvk_em1_del** | **+/-** | **F** | **328106** | **10/1/2015** | NA | 8.71 | 93.74 | 20.42 | 345.12 | 1399.00 | 60.72 | 14092.00 | 8930.70 | 3622.20 | 102.46 | 1129.30 | 515.04 | 8522.40 | 27.57 | 1.90 | 92.80 | 2004.20 | 23151.00 | 36660.00 | 3508.50 | 229.37 | 457.59 | 16.07 | 2455.30 | 505.75 | 6.75 | 2658.90 | 2.97 | 0.00 | 11.34 | 28.33 | 2.80 | 68.07 | 4244.40 | 91.52 | 176.84 | 657.07 | 47.37 | 4.01 | 7.22 | 16.87 | 87.69 | 4.06 | 4.15 | 948.15 | 32.32 | 12.67 | 1120.10 | 9.35 | 36.31 | 24.90 |
| 328100 | **NorCOMM2** | **Mvk** | **Mvk_em1_del** | **+/-** | **F** | **328100** | **10/1/2015** | NA | 7.04 | 86.74 | 30.71 | 299.67 | 1264.70 | 52.50 | 15303.00 | 6440.40 | 3430.00 | 281.58 | 454.32 | 504.47 | 7660.30 | 14.97 | 1.81 | 114.50 | 1942.20 | 18017.00 | 36313.00 | 3292.70 | 283.66 | 449.05 | 29.57 | 1647.40 | 491.98 | 5.32 | 2344.50 | 2.80 | 0.86 | 10.13 | 28.21 | 0.00 | 56.41 | 3435.20 | 78.64 | 168.98 | 436.02 | 43.76 | 3.84 | 5.68 | 6.79 | 99.69 | 3.19 | 3.76 | 906.84 | 24.56 | 13.99 | 945.20 | 6.99 | 39.84 | 26.88 |
| 328194 | **NorCOMM2** | **Mvk** | **Mvk_em1_del** | **+/-** | **F** | **328194** | **10/1/2015** | NA | 4.23 | 87.20 | 25.29 | 279.28 | 1284.90 | 66.58 | 9917.90 | 5200.90 | 3297.80 | 48.57 | 341.89 | 526.64 | 8770.00 | 30.42 | 2.52 | 103.38 | 2001.40 | 26243.00 | 39998.00 | 2896.50 | 333.22 | 414.30 | 25.80 | 2221.90 | 394.01 | 4.45 | 2740.00 | 0.00 | 0.00 | 7.91 | 24.69 | 0.00 | 53.44 | 3979.50 | 89.77 | 167.38 | 513.27 | 36.20 | 3.68 | 6.94 | 9.28 | 100.81 | 3.92 | 2.04 | 993.86 | 32.58 | 9.94 | 969.97 | 6.35 | 37.72 | 24.21 |
| 328004 | **NorCOMM2** | **Mvk** | **Mvk_em1_del** | **+/-** | **M** | **328004** | **9/29/2015** | NA | 14.94 | 87.30 | 12.70 | 172.78 | 1961.40 | 182.55 | 27314.00 | 8400.40 | 4070.00 | 5886.60 | 0.00 | 496.56 | 9713.30 | 5.99 | 2.39 | 94.48 | 2167.30 | 33196.00 | 44161.00 | 4112.70 | 241.48 | 707.79 | 19.85 | 2434.20 | 633.71 | 0.00 | 2967.20 | 6.20 | 0.00 | 12.65 | 19.16 | 2.93 | 38.44 | 2004.00 | 182.82 | 203.80 | 675.21 | 30.98 | 4.62 | 7.78 | 14.26 | 149.09 | 6.45 | 4.77 | 1050.70 | 23.35 | 16.55 | 2453.10 | 11.48 | 43.16 | 29.46 |
| 328098 | **NorCOMM2** | **Mvk** | **Mvk_em1_del** | **+/-** | **M** | **328098** | **9/29/2015** | NA | 9.61 | 99.71 | 10.98 | 131.92 | 2440.90 | 425.24 | 26944.00 | 7172.70 | 3613.60 | 5265.90 | 0.00 | 589.88 | 8342.60 | 5.97 | 6.82 | 107.70 | 2235.70 | 30651.00 | 47499.00 | 3641.50 | 190.13 | 626.06 | 39.56 | 2757.90 | 619.91 | 6.14 | 2504.20 | 0.00 | 1.71 | 13.10 | 18.41 | 5.11 | 48.30 | 2665.80 | 201.19 | 206.34 | 587.29 | 26.02 | 3.22 | 7.81 | 10.78 | 167.70 | 4.72 | 4.74 | 1115.80 | 19.70 | 10.45 | 1991.00 | 10.71 | 41.18 | 28.91 |
| 345318 | **NorCOMM2** | **Mvk** | **Mvk_em1_del** | **+/-** | **M** | **345318** | **10/27/2015** | NA | 0.00 | 97.14 | 15.63 | 287.03 | 1058.10 | 27.57 | 28094.00 | 7236.20 | 4259.70 | 1324.80 | 0.00 | 540.40 | 11058.00 | 6.51 | 2.43 | 86.63 | 1916.50 | 30814.00 | 59516.00 | 3194.60 | 321.38 | 552.90 | 26.53 | 1860.50 | 467.82 | 5.51 | 2470.20 | 3.00 | 0.00 | 11.67 | 38.20 | 6.40 | 69.41 | 2704.70 | 134.53 | 188.06 | 654.60 | 35.02 | 2.64 | 6.58 | 5.23 | 160.09 | 3.41 | 3.06 | 979.96 | 16.34 | 9.04 | 1568.70 | 10.40 | 45.51 | 22.89 |
| 324602 | **NorCOMM2** | **Nek2** | **Nek2_em1_nhej** | **-/-** | **F** | **324602** | **9/22/2015** | NA | 8.95 | 110.42 | 24.29 | 346.08 | 1603.20 | 69.30 | 15708.00 | 6725.50 | 3957.50 | 89.05 | 1154.80 | 537.46 | 7735.00 | 15.49 | 2.78 | 110.02 | 1964.80 | 19721.00 | 45030.00 | 3621.30 | 312.73 | 500.06 | 25.38 | 3029.50 | 510.63 | 5.19 | 2595.50 | 0.00 | 0.00 | 14.72 | 29.52 | 0.00 | 46.91 | 3224.30 | 80.61 | 183.71 | 642.61 | 54.35 | 4.30 | 7.14 | 9.05 | 116.13 | 2.85 | 2.62 | 874.29 | 27.44 | 10.34 | 1277.00 | 7.63 | 37.79 | 31.60 |
| 324611 | **NorCOMM2** | **Nek2** | **Nek2_em1_nhej** | **-/-** | **F** | **324611** | **9/22/2015** | NA | 8.99 | 98.62 | 26.18 | 298.84 | 1165.80 | 59.21 | 13161.00 | 7538.20 | 3293.00 | 81.56 | 540.68 | 496.85 | 7821.20 | 18.90 | 1.81 | 104.59 | 1602.70 | 23221.00 | 33885.00 | 2881.40 | 384.17 | 439.23 | 21.67 | 2810.70 | 474.18 | 2.73 | 2207.90 | 3.16 | 0.00 | 10.20 | 22.57 | 2.54 | 56.03 | 2862.90 | 97.01 | 164.30 | 610.34 | 46.39 | 3.10 | 7.23 | 10.63 | 61.04 | 4.28 | 2.11 | 772.66 | 26.04 | 7.21 | 1026.40 | 8.23 | 35.79 | 17.26 |
| 324604 | **NorCOMM2** | **Nek2** | **Nek2_em1_nhej** | **-/-** | **F** | **324604** | **9/22/2015** | NA | 9.48 | 85.08 | 34.51 | 278.24 | 1060.00 | 115.75 | 12632.00 | 7126.20 | 3406.00 | 55.78 | 671.28 | 486.93 | 7786.70 | 17.77 | 2.29 | 110.01 | 1669.30 | 23369.00 | 40686.00 | 3040.20 | 281.51 | 364.15 | 22.34 | 3024.60 | 511.43 | 6.30 | 2402.60 | 7.81 | 3.71 | 8.87 | 38.90 | 7.17 | 74.55 | 2856.70 | 86.09 | 170.57 | 600.62 | 47.57 | 2.84 | 7.28 | 10.23 | 104.61 | 4.40 | 3.23 | 881.28 | 17.32 | 13.17 | 971.53 | 8.85 | 32.50 | 26.62 |
| 363815 | **NorCOMM2** | **Nek2** | **Nek2_em1_nhej** | **-/-** | **M** | **363815** | **12/10/2015** | NA | 9.77 | 107.82 | 9.82 | 146.32 | 1878.60 | 115.85 | 21831.00 | 9155.70 | 4151.40 | 4892.00 | 0.00 | 486.43 | 9171.10 | 6.04 | 2.08 | 104.25 | 2083.60 | 35105.00 | 51052.00 | 4039.00 | 272.57 | 613.77 | 24.28 | 2794.90 | 575.31 | 2.73 | 2562.80 | 4.47 | 0.00 | 11.56 | 34.76 | 4.17 | 36.66 | 2431.80 | 145.82 | 180.82 | 521.95 | 25.06 | 2.84 | 8.37 | 7.84 | 83.33 | 4.72 | 3.57 | 924.82 | 13.22 | 15.04 | 2415.00 | 10.10 | 45.94 | 23.83 |
| 366625 | **NorCOMM2** | **Nek2** | **Nek2_em1_nhej** | **-/-** | **M** | **366625** | **12/17/2015** | NA | 12.26 | 96.75 | 10.84 | 231.51 | 1184.10 | 26.12 | 20634.00 | 6804.20 | 3452.70 | 2835.30 | 0.00 | 473.86 | 9392.60 | 0.00 | 2.66 | 92.31 | 1706.30 | 26214.00 | 47215.00 | 3601.20 | 246.39 | 709.56 | 26.03 | 1781.40 | 445.96 | 0.00 | 2362.60 | 0.00 | 0.00 | 8.64 | 18.21 | 5.11 | 64.73 | 1809.70 | 122.97 | 179.31 | 577.92 | 27.14 | 2.88 | 5.43 | 9.56 | 64.39 | 3.44 | 1.58 | 808.46 | 15.16 | 8.90 | 1744.70 | 7.93 | 32.68 | 17.87 |
| 363822 | **NorCOMM2** | **Nek2** | **Nek2_em1_nhej** | **-/-** | **M** | **363822** | **12/10/2015** | NA | 7.85 | 110.54 | 14.17 | 193.71 | 2857.10 | 421.12 | 31678.00 | 8150.60 | 3832.80 | 5159.50 | 0.00 | 595.26 | 10401.00 | 0.00 | 2.74 | 102.73 | 1743.70 | 31775.00 | 45545.00 | 3106.80 | 300.69 | 466.05 | 21.27 | 2070.10 | 489.23 | 9.02 | 2603.50 | 3.75 | 1.56 | 15.17 | 77.94 | 3.55 | 51.37 | 2026.30 | 179.69 | 212.68 | 644.76 | 26.60 | 3.91 | 8.33 | 6.37 | 74.27 | 4.80 | 3.75 | 1014.00 | 13.53 | 12.48 | 2029.80 | 12.74 | 41.07 | 30.92 |
| 75974 | **NorCOMM2** | **Npc2** | **Npc2_tm1e.1_A06** | **+/-** | **F** | **75974** | **4/1/2014** | NA | 12.04 | 385.50 | 25.65 | 305.99 | 1213.60 | 60.84 | 13880.00 | 6217.40 | 3629.50 | 91.47 | 202.60 | 398.06 | 8159.70 | 13.16 | 11.33 | 97.03 | 1849.00 | 23644.00 | 40271.00 | 2283.70 | 268.34 | 467.60 | 22.90 | 2175.70 | 503.88 | 0.00 | 2655.70 | 6.55 | 4.54 | 14.79 | 22.09 | 11.54 | 88.76 | 4303.10 | 86.67 | 162.15 | 666.82 | 36.03 | 3.37 | 5.83 | 8.11 | 130.19 | 5.00 | 3.52 | 739.63 | 24.12 | 8.91 | 1226.30 | 9.61 | 33.26 | 34.23 |
| 75975 | **NorCOMM2** | **Npc2** | **Npc2_tm1e.1_A06** | **+/-** | **F** | **75975** | **4/1/2014** | NA | 12.37 | 430.07 | 23.91 | 331.91 | 1426.10 | 50.08 | 13254.00 | 5989.10 | 3095.70 | 98.31 | 210.14 | 436.24 | 8972.30 | 17.54 | 12.66 | 111.47 | 1793.10 | 30340.00 | 40751.00 | 2357.00 | 262.34 | 285.98 | 27.42 | 1663.60 | 457.59 | 3.35 | 2629.30 | 7.92 | 3.72 | 8.71 | 42.13 | 5.37 | 62.93 | 3829.40 | 72.91 | 170.06 | 562.18 | 44.50 | 3.68 | 6.20 | 9.36 | 72.57 | 5.24 | 3.38 | 873.11 | 24.00 | 8.19 | 1016.50 | 7.24 | 32.13 | 23.74 |
| 75976 | **NorCOMM2** | **Npc2** | **Npc2_tm1e.1_A06** | **+/-** | **F** | **75976** | **4/1/2014** | NA | 10.36 | 122.45 | 26.92 | 341.45 | 1402.60 | 78.91 | 11719.00 | 5712.90 | 3317.90 | 79.90 | 86.65 | 425.22 | 9401.00 | 19.02 | 4.94 | 118.10 | 887.35 | 26568.00 | 41678.00 | 2653.50 | 271.98 | 289.16 | 26.85 | 1978.10 | 561.14 | 7.27 | 2413.40 | 5.40 | 5.63 | 11.49 | 66.04 | 14.83 | 105.97 | 2834.10 | 67.81 | 170.29 | 450.80 | 45.42 | 4.11 | 7.18 | 9.31 | 123.05 | 4.22 | 3.21 | 844.45 | 22.08 | 10.86 | 1144.20 | 8.67 | 37.28 | 21.68 |
| 92278 | **NorCOMM2** | **Npc2** | **Npc2_tm1e.1_A06** | **+/-** | **M** | **92278** | **4/29/2014** | NA | 6.47 | 125.73 | 13.85 | 253.65 | 1318.80 | 37.52 | 25048.00 | 7408.10 | 4771.70 | 3864.50 | 0.00 | 727.24 | 11582.00 | 6.46 | 9.28 | 107.08 | 1892.40 | 38553.00 | 49555.00 | 3379.20 | 316.20 | 475.58 | 22.27 | 1614.40 | 699.11 | 6.99 | 2671.60 | 11.30 | 5.64 | 16.75 | 31.78 | 20.41 | 102.15 | 2452.20 | 133.10 | 194.49 | 638.50 | 36.83 | 3.40 | 7.10 | 9.32 | 62.99 | 4.72 | 3.71 | 1023.20 | 19.78 | 9.38 | 1941.10 | 15.42 | 41.50 | 24.07 |
| 92277 | **NorCOMM2** | **Npc2** | **Npc2_tm1e.1_A06** | **+/-** | **M** | **92277** | **4/29/2014** | NA | 13.46 | 208.27 | 9.72 | 130.38 | 5500.80 | 3837.40 | 25666.00 | 6989.80 | 3463.30 | 6036.10 | 0.00 | 653.08 | 11013.00 | 7.80 | 7.41 | 148.41 | 1585.70 | 33041.00 | 42173.00 | 4515.70 | 293.88 | 476.68 | 32.22 | 2496.70 | 661.68 | 6.99 | 2669.30 | 4.29 | 5.66 | 21.58 | 28.39 | 16.07 | 116.36 | 1756.60 | 246.32 | 193.03 | 687.94 | 26.99 | 5.74 | 10.44 | 14.41 | 126.96 | 7.07 | 5.15 | 1567.30 | 15.30 | 13.48 | 3290.50 | 13.37 | 51.06 | 41.39 |
| 92287 | **NorCOMM2** | **Npc2** | **Npc2_tm1e.1_A06** | **+/-** | **M** | **92287** | **4/29/2014** | NA | 14.84 | 166.72 | 10.76 | 157.58 | 5664.70 | 4367.30 | 25104.00 | 4923.80 | 3169.20 | 5838.70 | 0.00 | 594.03 | 10115.00 | 0.00 | 2.94 | 145.46 | 2052.00 | 29752.00 | 33826.00 | 3788.60 | 414.24 | 492.16 | 32.87 | 3159.60 | 554.41 | 5.65 | 2930.10 | 0.00 | 0.00 | 20.95 | 15.40 | 3.13 | 39.55 | 1727.10 | 262.88 | 179.93 | 684.01 | 24.38 | 5.33 | 11.49 | 11.18 | 254.39 | 5.43 | 5.88 | 1713.90 | 15.35 | 14.42 | 2576.20 | 13.45 | 48.09 | 36.97 |
| 417059 | **KOMP2** | **Pebp1** | **Pebp1_em1_del** | **-/-** | **F** | **417059** | **7/20/2016** | NA | 5.63 | 94.81 | 14.26 | 291.13 | 1193.08 | 35.51 | 16002.01 | 7153.50 | 3177.79 | 213.33 | 300.10 | 418.97 | 6451.62 | 32.23 | 2.13 | 163.59 | 2686.46 | 27225.68 | 38915.09 | 2795.33 | 355.36 | 349.02 | 24.79 | 2275.88 | 552.28 | 3.79 | 3063.52 | 0.00 | 0.00 | 13.76 | 36.63 | 0.00 | 44.45 | 4531.25 | 88.37 | 175.17 | 451.59 | 38.28 | 3.69 | 4.26 | 11.14 | 210.49 | 3.61 | 0.00 | 994.89 | 26.25 | 11.93 | 1027.30 | 7.20 | 45.33 | 31.76 |
| 428681 | **KOMP2** | **Pebp1** | **Pebp1_em1_del** | **-/-** | **F** | **428681** | **8/23/2016** | NA | 5.28 | 106.66 | 30.50 | 319.15 | 1043.20 | 71.83 | 16556.46 | 6564.52 | 3502.77 | 97.46 | 213.89 | 329.08 | 7433.86 | 16.38 | 2.56 | 139.54 | 2043.92 | 24918.65 | 36149.12 | 2782.58 | 334.42 | 319.88 | 19.24 | 1876.73 | 472.64 | 2.96 | 2557.04 | 3.68 | 0.00 | 10.90 | 17.94 | 3.78 | 60.51 | 3027.96 | 76.09 | 163.29 | 550.09 | 44.38 | 3.07 | 5.85 | 11.98 | 96.43 | 4.04 | 3.11 | 729.68 | 21.91 | 10.31 | 921.00 | 6.46 | 35.98 | 28.18 |
| 417051 | **KOMP2** | **Pebp1** | **Pebp1_em1_del** | **-/-** | **F** | **417051** | **7/13/2016** | NA | 5.45 | 86.98 | 27.70 | 246.76 | 680.29 | 26.05 | 14738.98 | 5354.17 | 2647.28 | 629.30 | 459.98 | 380.99 | 6321.16 | 16.93 | 1.79 | 110.98 | 1922.17 | 20703.73 | 20547.35 | 2764.38 | 247.87 | 230.69 | 12.27 | 1911.54 | 371.17 | 4.48 | 2282.39 | 0.00 | 1.79 | 11.92 | 29.11 | 0.00 | 56.56 | 4116.15 | 73.41 | 146.25 | 428.46 | 38.38 | 3.66 | 4.90 | 12.18 | 81.91 | 3.19 | 0.00 | 719.90 | 15.91 | 8.00 | 1124.31 | 4.75 | 29.67 | 17.27 |
| 429694 | **KOMP2** | **Pebp1** | **Pebp1_em1_del** | **-/-** | **M** | **429694** | **8/23/2016** | NA | 12.85 | 175.73 | 7.91 | 160.33 | 3254.29 | 518.15 | 31198.03 | 8537.83 | 3525.78 | 5850.99 | 0.00 | 451.97 | 6746.00 | 7.58 | 3.34 | 164.28 | 2112.20 | 27778.44 | 40468.85 | 2732.45 | 366.31 | 441.70 | 22.60 | 2471.20 | 528.81 | 4.62 | 2495.11 | 0.00 | 0.00 | 15.76 | 13.68 | 0.00 | 35.25 | 2769.88 | 163.14 | 179.50 | 544.97 | 25.18 | 4.42 | 7.67 | 9.38 | 85.65 | 4.65 | 6.17 | 1024.35 | 19.30 | 20.30 | 2614.32 | 9.20 | 44.46 | 26.47 |
| 419964 | **KOMP2** | **Pebp1** | **Pebp1_em1_del** | **-/-** | **M** | **419964** | **7/28/2016** | NA | 9.52 | 81.31 | 9.96 | 219.50 | 1137.03 | 29.38 | 32346.54 | 9707.95 | 3974.11 | 2871.90 | 0.00 | 574.11 | 7906.25 | 6.98 | 2.34 | 153.74 | 2357.10 | 37570.47 | 60034.99 | 4766.37 | 318.06 | 510.74 | 16.51 | 2728.16 | 527.48 | 3.91 | 2991.67 | 0.00 | 0.00 | 11.17 | 23.66 | 3.92 | 43.52 | 2775.68 | 128.66 | 194.58 | 556.02 | 36.06 | 2.13 | 5.62 | 10.33 | 85.47 | 4.03 | 0.00 | 803.93 | 13.40 | 10.53 | 2262.75 | 7.85 | 43.72 | 27.39 |
| 435823 | **KOMP2** | **Pebp1** | **Pebp1_em1_del** | **-/-** | **M** | **435823** | **9/8/2016** | NA | 8.58 | 88.71 | 13.86 | 211.99 | 994.91 | 32.15 | 33829.00 | 8615.11 | 3942.92 | 2088.85 | 0.00 | 421.91 | 9027.58 | 7.06 | 1.73 | 137.41 | 2295.95 | 41247.79 | 58102.16 | 3436.64 | 351.71 | 596.64 | 11.37 | 2581.75 | 513.39 | 3.97 | 3425.67 | 0.00 | 0.00 | 12.81 | 17.86 | 0.00 | 49.68 | 2915.20 | 119.48 | 201.44 | 722.92 | 30.91 | 2.82 | 5.45 | 7.74 | 60.79 | 3.55 | 0.00 | 760.30 | 15.28 | 10.32 | 1452.68 | 9.32 | 47.56 | 23.56 |
| 244286 | **NorCOMM2** | **Phyh** | **Phyh_tm1b_G08** | **-/-** | **F** | **244286** | **5/1/2015** | NA | 7.31 | 100.64 | 20.79 | 273.68 | 1001.61 | 38.15 | 12163.03 | 6544.88 | 2909.65 | 51.97 | 274.96 | 386.15 | 6341.48 | 25.41 | 2.34 | 115.74 | 2541.11 | 32122.52 | 40902.98 | 3798.36 | 253.85 | 497.09 | 20.30 | 2029.04 | 476.58 | 3.26 | 2259.13 | 3.90 | 0.00 | 11.83 | 33.69 | 4.08 | 39.23 | 3614.76 | 69.79 | 143.17 | 468.26 | 34.51 | 3.04 | 6.24 | 7.20 | 158.72 | 3.25 | 3.72 | 757.15 | 26.52 | 8.49 | 725.83 | 7.26 | 28.35 | 22.17 |
| 237159 | **NorCOMM2** | **Phyh** | **Phyh_tm1b_G08** | **-/-** | **F** | **237159** | **4/21/2015** | NA | 5.87 | 62.61 | 17.22 | 270.14 | 904.64 | 54.20 | 12940.79 | 6057.33 | 2691.40 | 48.67 | 311.10 | 374.21 | 7190.11 | 18.74 | 2.55 | 99.70 | 1701.39 | 24966.16 | 37234.93 | 1746.32 | 273.63 | 232.07 | 15.85 | 1626.76 | 393.96 | 2.90 | 2363.67 | 3.34 | 7.24 | 7.33 | 43.00 | 14.63 | 113.10 | 4725.49 | 63.19 | 160.09 | 471.04 | 45.19 | 2.96 | 5.33 | 8.28 | 231.82 | 2.42 | 2.16 | 908.71 | 31.40 | 4.48 | 1115.17 | 10.69 | 31.30 | 19.62 |
| 237165 | **NorCOMM2** | **Phyh** | **Phyh_tm1b_G08** | **-/-** | **F** | **237165** | **4/21/2015** | NA | 5.73 | 164.33 | 25.00 | 249.73 | 769.58 | 34.95 | 10811.25 | 6402.61 | 2706.07 | 40.31 | 76.32 | 431.86 | 6663.30 | 32.69 | 5.20 | 105.36 | 1671.95 | 29235.53 | 39275.15 | 2766.60 | 286.70 | 275.08 | 25.73 | 1911.20 | 421.36 | 3.98 | 2762.60 | 3.39 | 6.88 | 11.06 | 15.05 | 16.92 | 98.48 | 3431.44 | 69.64 | 150.90 | 505.79 | 39.79 | 3.19 | 5.99 | 7.75 | 87.10 | 2.62 | 2.67 | 831.20 | 24.83 | 9.21 | 875.92 | 6.51 | 41.25 | 27.90 |
| 237056 | **NorCOMM2** | **Phyh** | **Phyh_tm1b_G08** | **-/-** | **M** | **237056** | **4/21/2015** | NA | 15.23 | 98.98 | 8.55 | 134.71 | 1958.62 | 237.28 | 22937.15 | 8791.34 | 3257.36 | 4433.04 | 0.00 | 649.29 | 7071.76 | 0.00 | 2.26 | 131.42 | 2094.22 | 42554.20 | 47798.08 | 3881.21 | 325.70 | 537.95 | 27.58 | 2872.53 | 544.26 | 4.61 | 2531.52 | 3.93 | 0.00 | 17.53 | 14.45 | 0.00 | 32.20 | 2366.46 | 200.97 | 178.34 | 610.08 | 28.00 | 3.10 | 7.76 | 6.52 | 154.54 | 3.79 | 6.03 | 1133.88 | 16.13 | 9.65 | 2363.94 | 11.10 | 46.01 | 28.07 |
| 244177 | **NorCOMM2** | **Phyh** | **Phyh_tm1b_G08** | **-/-** | **M** | **244177** | **5/1/2015** | NA | 22.69 | 97.91 | 14.29 | 181.81 | 772.59 | 41.23 | 32120.65 | 9035.36 | 3275.12 | 2941.19 | 0.00 | 617.77 | 8751.69 | 6.68 | 2.31 | 123.74 | 2296.57 | 41928.99 | 57440.76 | 3270.16 | 221.01 | 625.09 | 14.79 | 2146.45 | 490.32 | 4.70 | 2538.31 | 0.00 | 0.00 | 12.75 | 11.44 | 4.53 | 59.83 | 3927.26 | 126.10 | 196.93 | 684.09 | 36.99 | 1.77 | 7.13 | 7.66 | 169.87 | 2.22 | 4.07 | 892.41 | 17.36 | 8.65 | 2450.11 | 7.95 | 40.39 | 27.12 |
| 237057 | **NorCOMM2** | **Phyh** | **Phyh_tm1b_G08** | **-/-** | **M** | **237057** | **4/21/2015** | NA | 14.67 | 70.57 | 8.69 | 149.05 | 1727.10 | 92.12 | 28363.35 | 8618.80 | 2846.91 | 4317.22 | 0.00 | 436.00 | 7579.91 | 6.54 | 2.18 | 120.41 | 2042.52 | 40652.62 | 51282.63 | 3491.24 | 298.04 | 595.53 | 26.37 | 1885.90 | 535.83 | 9.29 | 2418.35 | 2.84 | 3.25 | 13.77 | 15.71 | 7.12 | 67.70 | 2544.26 | 123.70 | 152.08 | 449.80 | 27.33 | 3.64 | 8.11 | 8.47 | 123.29 | 3.63 | 2.87 | 996.59 | 17.08 | 7.15 | 2251.08 | 10.24 | 44.01 | 26.83 |
| 170510 | **NorCOMM2** | **Pipox** | **Pipox_tm1b_A08** | **-/-** | **F** | **170510** | **10/9/2014** | NA | 4.95 | 89.97 | 27.68 | 294.96 | 1013.00 | 40.28 | 17784.00 | 7022.40 | 4222.50 | 149.67 | 391.73 | 412.37 | 9129.10 | 14.98 | 3.91 | 103.80 | 1714.70 | 22849.00 | 36835.00 | 3074.80 | 289.96 | 282.60 | 33.39 | 1780.30 | 516.98 | 4.12 | 2289.20 | 0.00 | 2.63 | 10.62 | 55.05 | 6.32 | 78.12 | 3472.70 | 72.39 | 178.12 | 576.26 | 40.96 | 3.32 | 6.26 | 8.63 | 94.81 | 3.61 | 2.08 | 829.42 | 20.91 | 11.16 | 1015.90 | 7.65 | 33.01 | 30.69 |
| 170501 | **NorCOMM2** | **Pipox** | **Pipox_tm1b_A08** | **-/-** | **F** | **170501** | **10/9/2014** | NA | 6.75 | 122.44 | 30.95 | 317.28 | 902.07 | 66.60 | 12241.00 | 5015.40 | 3418.40 | 134.65 | 264.02 | 470.07 | 8585.20 | 16.60 | 4.25 | 108.89 | 1642.10 | 25526.00 | 33696.00 | 3384.50 | 185.21 | 290.45 | 26.70 | 1698.00 | 412.97 | 4.58 | 2153.90 | 3.60 | 0.00 | 12.36 | 23.50 | 5.21 | 55.41 | 2864.90 | 76.49 | 161.38 | 509.84 | 42.47 | 3.25 | 5.23 | 8.45 | 64.04 | 4.50 | 2.91 | 777.72 | 21.43 | 12.02 | 1008.60 | 7.16 | 30.51 | 25.96 |
| 170500 | **NorCOMM2** | **Pipox** | **Pipox_tm1b_A08** | **-/-** | **F** | **170500** | **10/9/2014** | NA | 11.87 | 123.96 | 27.01 | 375.91 | 1331.10 | 83.11 | 15203.00 | 5531.60 | 3628.90 | 73.68 | 810.61 | 453.63 | 9183.60 | 10.43 | 3.47 | 117.10 | 1869.80 | 25711.00 | 40363.00 | 3342.10 | 293.82 | 282.85 | 28.88 | 1702.00 | 475.10 | 4.74 | 2630.10 | 3.29 | 2.13 | 11.97 | 24.93 | 4.93 | 65.62 | 3743.40 | 87.30 | 175.61 | 625.86 | 48.53 | 4.29 | 5.78 | 12.06 | 49.03 | 3.85 | 3.50 | 825.76 | 24.03 | 8.46 | 965.07 | 8.87 | 34.12 | 39.66 |
| 170499 | **NorCOMM2** | **Pipox** | **Pipox_tm1b_A08** | **-/-** | **M** | **170499** | **10/9/2014** | NA | 12.40 | 108.19 | 15.06 | 197.12 | 1464.10 | 38.39 | 28867.00 | 8395.00 | 4097.40 | 5302.20 | 0.00 | 454.54 | 10860.00 | 6.52 | 4.51 | 97.80 | 1688.40 | 32637.00 | 44745.00 | 2804.90 | 264.57 | 397.98 | 20.68 | 1879.30 | 591.53 | 6.39 | 2456.30 | 4.65 | 3.51 | 13.40 | 27.15 | 11.90 | 101.90 | 2134.60 | 130.21 | 203.74 | 632.96 | 30.08 | 3.34 | 6.93 | 8.10 | 46.22 | 3.49 | 2.45 | 956.13 | 13.96 | 8.70 | 1940.80 | 9.16 | 40.30 | 27.44 |
| 170406 | **NorCOMM2** | **Pipox** | **Pipox_tm1b_A08** | **-/-** | **M** | **170406** | **10/9/2014** | NA | 7.25 | 122.89 | 14.20 | 225.55 | 1926.10 | 29.64 | 31155.00 | 9410.00 | 4657.90 | 6207.00 | 0.00 | 494.93 | 10931.00 | 5.47 | 4.85 | 109.43 | 1800.80 | 41615.00 | 48201.00 | 3739.00 | 299.37 | 503.25 | 26.21 | 2053.50 | 667.80 | 0.00 | 2512.30 | 8.14 | 5.96 | 11.81 | 41.68 | 13.85 | 121.24 | 2408.50 | 145.42 | 203.30 | 738.81 | 33.07 | 4.56 | 8.19 | 10.74 | 69.35 | 5.00 | 2.75 | 915.01 | 14.68 | 11.16 | 2128.60 | 12.18 | 42.82 | 25.49 |
| 172415 | **NorCOMM2** | **Pipox** | **Pipox_tm1b_A08** | **-/-** | **M** | **172415** | **10/16/2014** | NA | 6.96 | 83.91 | 10.54 | 260.00 | 1169.70 | 26.10 | 23437.00 | 7176.50 | 4029.40 | 1699.90 | 0.00 | 374.71 | 11612.00 | 0.00 | 3.51 | 109.11 | 1408.90 | 33477.00 | 58884.00 | 4341.00 | 261.22 | 598.00 | 13.06 | 2718.30 | 459.13 | 8.34 | 3341.70 | 4.49 | 0.00 | 12.61 | 37.15 | 6.23 | 62.65 | 2527.50 | 106.24 | 203.14 | 599.17 | 35.22 | 2.80 | 6.97 | 7.59 | 39.91 | 3.72 | 3.28 | 911.31 | 30.83 | 10.15 | 1555.00 | 9.88 | 41.96 | 22.82 |
| 198681 | **NorCOMM2** | **Plk1** | **Plk1_tm1b_E04** | **+/-** | **F** | **198681** | **12/23/2014** | NA | 7.68 | 81.36 | 21.69 | 295.44 | 1021.28 | 71.92 | 13777.75 | 6906.46 | 3636.05 | 43.92 | 774.23 | 505.16 | 6997.72 | 15.53 | 2.17 | 97.48 | 2181.63 | 20416.63 | 37894.94 | 2647.13 | 275.94 | 336.40 | 23.53 | 1932.06 | 411.77 | 3.25 | 2543.86 | 3.11 | 0.00 | 10.90 | 22.27 | 0.00 | 38.88 | 3520.18 | 63.02 | 149.88 | 572.50 | 44.33 | 3.19 | 5.37 | 6.88 | 69.82 | 3.32 | 3.70 | 716.59 | 23.44 | 8.02 | 992.29 | 5.10 | 32.47 | 22.37 |
| 198682 | **NorCOMM2** | **Plk1** | **Plk1_tm1b_E04** | **+/-** | **F** | **198682** | **12/23/2014** | NA | 5.47 | 90.85 | 17.33 | 278.80 | 1039.93 | 52.54 | 12997.99 | 6587.61 | 3239.60 | 127.47 | 100.64 | 426.42 | 6818.86 | 12.03 | 1.69 | 94.47 | 1967.31 | 24216.55 | 33329.12 | 2929.90 | 239.39 | 367.37 | 28.86 | 2313.26 | 396.39 | 2.92 | 2449.91 | 3.93 | 0.00 | 11.25 | 25.76 | 3.58 | 59.59 | 4281.37 | 74.98 | 150.63 | 520.51 | 38.13 | 2.58 | 4.89 | 8.84 | 80.50 | 2.90 | 3.87 | 808.02 | 21.83 | 9.04 | 1124.94 | 7.63 | 41.61 | 23.44 |
| 221097 | **NorCOMM2** | **Plk1** | **Plk1_tm1b_E04** | **+/-** | **F** | **221097** | **2/12/2015** | NA | 11.00 | 100.63 | 21.36 | 377.69 | 877.65 | 70.64 | 15201.79 | 7298.22 | 3025.88 | 71.23 | 704.73 | 453.93 | 6849.07 | 25.33 | 1.92 | 148.34 | 1807.26 | 28143.04 | 37220.13 | 3084.61 | 272.20 | 285.04 | 22.85 | 1478.75 | 448.56 | 4.15 | 2573.26 | 3.02 | 0.00 | 13.02 | 56.14 | 2.63 | 53.08 | 4882.59 | 74.54 | 166.17 | 600.59 | 44.72 | 3.99 | 7.35 | 11.44 | 155.61 | 3.81 | 3.76 | 823.42 | 19.74 | 5.56 | 1691.03 | 6.65 | 41.57 | 30.85 |
| 215487 | **NorCOMM2** | **Plk1** | **Plk1_tm1b_E04** | **+/-** | **M** | **215487** | **1/27/2015** | NA | 8.41 | 109.03 | 9.28 | 181.50 | 1447.84 | 88.42 | 23124.82 | 8283.03 | 2975.39 | 5504.93 | 0.00 | 452.19 | 7280.91 | 6.51 | 2.53 | 123.59 | 1919.29 | 32176.61 | 44598.65 | 3693.56 | 345.20 | 659.92 | 32.26 | 2423.75 | 557.84 | 5.67 | 2623.83 | 3.50 | 1.13 | 13.18 | 18.91 | 3.46 | 41.86 | 2449.15 | 126.80 | 165.48 | 531.18 | 26.75 | 3.51 | 5.61 | 7.95 | 78.32 | 2.79 | 4.42 | 824.99 | 12.87 | 6.82 | 1682.47 | 8.75 | 26.43 | 29.51 |
| 198577 | **NorCOMM2** | **Plk1** | **Plk1_tm1b_E04** | **+/-** | **M** | **198577** | **12/23/2014** | NA | 18.84 | 98.92 | 8.36 | 155.64 | 1780.94 | 135.90 | 32743.85 | 7914.26 | 3689.73 | 5456.57 | 0.00 | 482.13 | 8863.61 | 0.00 | 2.45 | 127.15 | 2119.74 | 34985.72 | 50079.02 | 3753.15 | 270.44 | 673.61 | 24.92 | 2419.97 | 583.43 | 4.13 | 2958.93 | 0.00 | 0.00 | 9.82 | 16.68 | 0.00 | 38.93 | 2465.60 | 143.61 | 182.78 | 571.34 | 31.37 | 3.81 | 7.95 | 8.81 | 104.47 | 2.82 | 5.50 | 916.40 | 17.41 | 8.06 | 2131.08 | 9.59 | 45.26 | 32.59 |
| 198579 | **NorCOMM2** | **Plk1** | **Plk1_tm1b_E04** | **+/-** | **M** | **198579** | **12/23/2014** | NA | 15.75 | 89.35 | 7.70 | 238.57 | 1538.18 | 65.06 | 24811.79 | 8365.86 | 3320.30 | 3720.85 | 0.00 | 415.15 | 7922.47 | 6.26 | 3.45 | 97.29 | 2177.60 | 37606.62 | 45792.39 | 3317.37 | 215.35 | 595.16 | 28.83 | 1875.74 | 514.57 | 5.73 | 2746.87 | 6.22 | 0.00 | 11.31 | 19.56 | 5.24 | 54.51 | 2556.78 | 122.27 | 172.29 | 673.52 | 32.68 | 3.41 | 7.44 | 4.37 | 84.40 | 3.81 | 3.99 | 835.09 | 13.54 | 8.80 | 2211.72 | 11.00 | 46.93 | 31.88 |
| 294361 | **NorCOMM2** | **Pmm2** | **Pmm2_tm1b_H04** | **+/-** | **F** | **294361** | **7/14/2015** | NA | 8.79 | 106.12 | 14.83 | 292.33 | 1193.29 | 68.95 | 14302.26 | 6366.46 | 2612.10 | 119.06 | 601.31 | 414.68 | 5878.41 | 21.21 | 2.34 | 153.68 | 2273.48 | 25045.97 | 38549.85 | 3577.57 | 292.24 | 338.73 | 24.67 | 2584.90 | 499.38 | 2.02 | 2477.25 | 0.00 | 0.00 | 10.94 | 33.81 | 0.00 | 49.63 | 4707.82 | 76.06 | 162.25 | 461.37 | 33.78 | 3.33 | 4.21 | 12.72 | 99.71 | 3.62 | 3.72 | 764.02 | 23.22 | 13.36 | 922.93 | 3.89 | 50.57 | 25.21 |
| 294362 | **NorCOMM2** | **Pmm2** | **Pmm2_tm1b_H04** | **+/-** | **F** | **294362** | **7/14/2015** | NA | 3.06 | 86.40 | 18.57 | 204.86 | 873.22 | 51.64 | 17318.56 | 6928.06 | 2959.42 | 111.20 | 235.99 | 388.96 | 5707.85 | 11.63 | 2.00 | 132.80 | 2298.72 | 27117.56 | 36387.02 | 3096.37 | 245.65 | 350.91 | 27.05 | 2212.67 | 428.74 | 2.43 | 2659.06 | 0.00 | 0.00 | 8.43 | 38.01 | 0.00 | 45.48 | 4445.97 | 61.87 | 148.78 | 491.39 | 42.30 | 2.93 | 4.53 | 7.09 | 45.83 | 3.62 | 0.00 | 733.11 | 24.18 | 11.75 | 1125.15 | 6.15 | 46.83 | 26.34 |
| 294360 | **NorCOMM2** | **Pmm2** | **Pmm2_tm1b_H04** | **+/-** | **F** | **294360** | **7/14/2015** | NA | 1.68 | 110.94 | 17.83 | 266.23 | 1356.18 | 82.95 | 14474.59 | 6029.32 | 2595.83 | 234.73 | 199.97 | 380.34 | 5481.66 | 9.43 | 2.72 | 120.50 | 1923.24 | 19327.39 | 32628.98 | 2999.04 | 322.89 | 245.91 | 23.63 | 2270.10 | 418.51 | 4.19 | 3130.77 | 3.40 | 0.00 | 11.10 | 14.95 | 0.00 | 36.84 | 3543.61 | 71.96 | 142.39 | 486.66 | 35.67 | 2.96 | 4.72 | 10.59 | 54.69 | 4.21 | 0.00 | 646.84 | 17.20 | 10.65 | 1134.88 | 4.83 | 39.57 | 24.69 |
| 294256 | **NorCOMM2** | **Pmm2** | **Pmm2_tm1b_H04** | **+/-** | **M** | **294256** | **7/14/2015** | NA | 8.91 | 62.09 | 8.59 | 128.90 | 1303.63 | 23.19 | 26455.23 | 8207.46 | 3031.61 | 4504.68 | 0.00 | 403.29 | 6128.70 | 0.00 | 2.06 | 116.83 | 2032.63 | 37258.22 | 53565.26 | 4319.52 | 269.13 | 509.82 | 23.66 | 2640.23 | 514.43 | 3.23 | 2860.51 | 3.20 | 0.00 | 7.43 | 13.52 | 0.00 | 38.13 | 2599.62 | 121.76 | 168.25 | 559.55 | 25.67 | 2.71 | 4.70 | 7.02 | 90.37 | 3.41 | 0.00 | 777.71 | 13.86 | 9.65 | 1849.62 | 8.22 | 37.02 | 33.53 |
| 294259 | **NorCOMM2** | **Pmm2** | **Pmm2_tm1b_H04** | **+/-** | **M** | **294259** | **7/16/2015** | NA | 13.21 | 68.78 | 10.40 | 169.35 | 1267.51 | 22.66 | 27970.87 | 7990.54 | 3212.98 | 4014.61 | 0.00 | 450.16 | 8053.25 | 0.00 | 1.88 | 126.72 | 1652.67 | 35724.24 | 55393.45 | 3455.23 | 292.50 | 444.58 | 18.79 | 2008.62 | 567.30 | 4.84 | 2401.02 | 3.38 | 0.00 | 10.58 | 19.37 | 4.44 | 49.06 | 3136.14 | 111.20 | 158.94 | 609.70 | 28.57 | 2.29 | 5.77 | 6.40 | 84.00 | 3.33 | 0.00 | 749.53 | 13.16 | 9.33 | 1937.19 | 7.72 | 41.91 | 24.78 |
| 294257 | **NorCOMM2** | **Pmm2** | **Pmm2_tm1b_H04** | **+/-** | **M** | **294257** | **7/14/2015** | NA | 6.89 | 78.64 | 10.82 | 190.14 | 1726.14 | 34.22 | 30118.03 | 7878.16 | 3434.86 | 5725.31 | 0.00 | 550.26 | 7878.24 | 0.00 | 2.63 | 135.35 | 2195.61 | 42925.27 | 52983.84 | 3949.09 | 261.55 | 473.00 | 18.84 | 2289.87 | 595.69 | 6.43 | 3045.03 | 0.00 | 0.00 | 7.62 | 15.82 | 7.13 | 77.82 | 3445.00 | 145.90 | 182.28 | 620.33 | 26.80 | 3.37 | 6.11 | 8.23 | 145.61 | 4.39 | 0.00 | 825.41 | 17.09 | 12.35 | 2138.32 | 7.43 | 43.04 | 24.48 |
| 229188 | **NorCOMM2** | **Ptpn12** | **Ptpn12_tm2b_H11** | **+/-** | **F** | **229188** | **3/17/2015** | NA | 6.58 | 140.19 | 19.17 | 398.56 | 1348.80 | 81.68 | 14954.00 | 5970.20 | 3231.60 | 152.24 | 351.38 | 454.17 | 8068.20 | 27.86 | 3.36 | 117.31 | 1705.50 | 28051.00 | 41919.00 | 3003.30 | 314.10 | 370.47 | 40.68 | 2365.00 | 411.70 | 6.67 | 2943.50 | 3.73 | 3.76 | 9.03 | 45.78 | 11.08 | 72.71 | 3478.90 | 91.66 | 171.69 | 488.12 | 35.53 | 3.80 | 5.35 | 9.55 | 60.56 | 4.47 | 1.58 | 963.54 | 21.80 | 10.34 | 1088.60 | 6.16 | 37.75 | 26.38 |
| 229189 | **NorCOMM2** | **Ptpn12** | **Ptpn12_tm2b_H11** | **+/-** | **F** | **229189** | **3/17/2015** | NA | 10.01 | 214.82 | 16.16 | 284.93 | 952.36 | 54.67 | 15611.00 | 5070.00 | 3425.10 | 141.50 | 118.64 | 396.67 | 7372.40 | 16.79 | 5.91 | 128.39 | 1885.70 | 20059.00 | 37069.00 | 2775.60 | 250.21 | 346.77 | 30.12 | 1939.30 | 405.56 | 5.49 | 2402.40 | 3.18 | 0.00 | 10.45 | 35.77 | 5.57 | 47.99 | 4068.70 | 75.92 | 165.30 | 544.94 | 38.68 | 2.87 | 5.22 | 9.01 | 93.40 | 3.99 | 3.11 | 916.28 | 21.10 | 12.21 | 1089.30 | 6.58 | 34.13 | 21.93 |
| 248916 | **NorCOMM2** | **Ptpn12** | **Ptpn12_tm2b_H11** | **+/-** | **F** | **248916** | **4/23/2015** | NA | 13.39 | 274.51 | 25.68 | 370.07 | 1424.30 | 72.77 | 13263.00 | 6422.20 | 3831.70 | 88.16 | 781.43 | 457.16 | 8287.10 | 13.20 | 6.82 | 136.63 | 2160.50 | 21210.00 | 41578.00 | 2800.50 | 315.85 | 381.19 | 27.43 | 2053.00 | 491.94 | 7.67 | 2767.70 | 5.03 | 2.13 | 16.84 | 35.87 | 6.66 | 77.03 | 3597.00 | 97.29 | 181.59 | 613.36 | 49.53 | 4.16 | 7.66 | 13.53 | 111.99 | 4.68 | 3.42 | 834.98 | 18.62 | 13.65 | 1153.10 | 8.55 | 43.92 | 37.42 |
| 229084 | **NorCOMM2** | **Ptpn12** | **Ptpn12_tm2b_H11** | **+/-** | **M** | **229084** | **3/19/2015** | NA | 12.42 | 95.84 | 11.54 | 140.95 | 1467.20 | 80.06 | 27597.00 | 7955.50 | 3318.10 | 4907.40 | 0.00 | 526.51 | 7022.80 | 6.66 | 2.32 | 91.76 | 1886.00 | 30302.00 | 41157.00 | 3696.80 | 289.93 | 424.14 | 27.21 | 2282.40 | 536.62 | 3.98 | 2706.10 | 3.41 | 0.00 | 12.99 | 28.03 | 2.99 | 37.93 | 2202.50 | 140.95 | 165.16 | 515.23 | 22.05 | 2.38 | 6.44 | 7.38 | 56.88 | 3.86 | 4.58 | 911.49 | 14.15 | 10.59 | 2584.40 | 8.71 | 39.49 | 31.00 |
| 229082 | **NorCOMM2** | **Ptpn12** | **Ptpn12_tm2b_H11** | **+/-** | **M** | **229082** | **3/19/2015** | NA | 7.28 | 90.79 | 8.56 | 220.57 | 1195.80 | 58.17 | 22603.00 | 8244.60 | 3827.80 | 2973.00 | 0.00 | 578.89 | 9493.40 | 6.78 | 3.91 | 108.14 | 2126.70 | 31751.00 | 52777.00 | 3962.60 | 232.50 | 426.33 | 29.90 | 2115.60 | 538.59 | 6.08 | 3120.30 | 2.68 | 3.92 | 11.16 | 19.34 | 10.08 | 111.17 | 2226.70 | 127.55 | 186.52 | 518.90 | 30.51 | 1.98 | 8.21 | 7.39 | 154.00 | 3.72 | 3.15 | 881.83 | 14.82 | 9.93 | 1595.50 | 11.03 | 41.51 | 27.27 |
| 229087 | **NorCOMM2** | **Ptpn12** | **Ptpn12_tm2b_H11** | **+/-** | **M** | **229087** | **3/19/2015** | NA | 12.32 | 147.68 | 12.69 | 165.91 | 1972.90 | 178.45 | 26636.00 | 8044.40 | 3811.60 | 5991.10 | 0.00 | 653.88 | 8904.10 | 6.14 | 4.91 | 134.44 | 2094.20 | 34948.00 | 46044.00 | 3254.90 | 291.79 | 414.15 | 26.59 | 2174.30 | 562.93 | 6.24 | 3341.60 | 3.60 | 0.00 | 15.96 | 17.64 | 4.10 | 47.96 | 3018.40 | 157.77 | 192.42 | 549.69 | 29.92 | 3.56 | 6.72 | 7.01 | 82.13 | 4.82 | 3.77 | 1104.10 | 13.92 | 13.40 | 1946.60 | 10.32 | 48.68 | 36.95 |
| 449031 | **KOMP2** | **Pttg1** | **Pttg1_tm1b_F08** | **-/-** | **F** | **449031** | **10/14/2016** | NA | 6.98 | 89.07 | 18.85 | 297.34 | 970.73 | 34.13 | 20158.25 | 6865.49 | 3181.12 | 995.36 | 6.26 | 404.31 | 7185.30 | 23.88 | 4.02 | 125.54 | 1672.49 | 34879.43 | 42317.77 | 3321.50 | 306.19 | 317.05 | 31.29 | 2418.19 | 476.62 | 3.83 | 2679.43 | 6.07 | 7.96 | 11.71 | 32.81 | 14.47 | 66.71 | 4193.69 | 74.53 | 178.29 | 475.08 | 37.32 | 3.42 | 4.96 | 9.06 | 63.59 | 4.00 | 3.14 | 665.83 | 18.30 | 6.07 | 1424.43 | 6.18 | 35.92 | 38.43 |
| 449028 | **KOMP2** | **Pttg1** | **Pttg1_tm1b_F08** | **-/-** | **F** | **449028** | **10/14/2016** | NA | 6.82 | 61.46 | 16.81 | 218.58 | 787.06 | 42.39 | 11226.03 | 4727.86 | 2426.45 | 69.46 | 24.49 | 292.94 | 5541.48 | 13.73 | 1.70 | 96.56 | 1468.71 | 18470.46 | 29979.49 | 2737.22 | 268.57 | 318.30 | 27.38 | 1721.27 | 344.02 | 3.66 | 1850.89 | 0.00 | 0.00 | 8.17 | 23.10 | 3.87 | 35.93 | 3516.74 | 62.20 | 107.13 | 443.07 | 32.31 | 2.41 | 3.69 | 6.65 | 32.40 | 2.84 | 0.00 | 478.67 | 14.03 | 7.40 | 765.40 | 5.61 | 31.58 | 21.77 |
| 493896 | **KOMP2** | **Pttg1** | **Pttg1_tm1b_F08** | **-/-** | **F** | **493896** | **2/2/2017** | NA | 7.15 | 106.90 | 20.77 | 249.87 | 1165.52 | 68.80 | 14364.35 | 6119.55 | 3067.08 | 57.57 | 59.38 | 324.72 | 6549.17 | 17.18 | 4.17 | 85.76 | 1765.58 | 19147.51 | 36515.35 | 4260.06 | 241.49 | 345.96 | 25.03 | 2518.85 | 354.26 | 6.59 | 2404.68 | 0.00 | 4.99 | 8.60 | 22.71 | 8.72 | 65.74 | 4019.06 | 63.06 | 141.38 | 399.32 | 37.39 | 3.14 | 5.14 | 9.24 | 62.95 | 2.45 | 0.00 | 517.67 | 19.61 | 10.29 | 899.30 | 6.38 | 32.24 | 24.27 |
| 481961 | **KOMP2** | **Pttg1** | **Pttg1_tm1b_F08** | **-/-** | **M** | **481961** | **12/21/2016** | NA | 8.79 | 137.86 | 8.26 | 210.37 | 1132.07 | 23.99 | 29404.10 | 9752.44 | 4123.14 | 1329.42 | 0.00 | 384.95 | 7958.25 | 0.00 | 3.46 | 118.73 | 2518.05 | 37865.55 | 60784.23 | 4364.49 | 314.25 | 691.16 | 15.50 | 2179.75 | 421.14 | 4.30 | 3004.93 | 0.00 | 5.29 | 8.23 | 16.79 | 10.16 | 57.99 | 2925.28 | 118.28 | 197.61 | 648.41 | 36.81 | 2.58 | 6.96 | 8.19 | 105.72 | 2.81 | 0.00 | 755.03 | 17.57 | 9.71 | 1842.70 | 9.59 | 41.46 | 23.58 |
| 481962 | **KOMP2** | **Pttg1** | **Pttg1_tm1b_F08** | **-/-** | **M** | **481962** | **12/21/2016** | NA | 7.04 | 197.79 | 14.05 | 212.97 | 1074.99 | 19.86 | 27081.40 | 9397.99 | 3790.18 | 1533.53 | 0.00 | 425.12 | 8007.43 | 7.83 | 5.75 | 99.40 | 2482.04 | 43008.13 | 58073.54 | 3677.05 | 295.46 | 818.47 | 16.46 | 2486.67 | 409.98 | 2.62 | 2854.55 | 3.77 | 0.00 | 11.25 | 20.06 | 5.27 | 47.43 | 2668.01 | 100.35 | 189.77 | 634.95 | 34.05 | 2.53 | 5.20 | 11.56 | 63.66 | 2.45 | 0.00 | 741.83 | 18.39 | 9.88 | 1469.61 | 9.03 | 40.16 | 25.19 |
| 448912 | **KOMP2** | **Pttg1** | **Pttg1_tm1b_F08** | **-/-** | **M** | **448912** | **10/14/2016** | NA | 8.09 | 98.24 | 9.61 | 192.01 | 936.74 | 17.36 | 29475.81 | 8023.91 | 3824.32 | 2300.60 | 0.00 | 313.82 | 7993.52 | 6.79 | 5.03 | 125.23 | 1827.69 | 41517.60 | 54462.57 | 3564.44 | 266.93 | 674.28 | 13.79 | 2853.85 | 504.92 | 5.61 | 2904.47 | 4.35 | 3.76 | 10.28 | 21.95 | 11.08 | 90.79 | 2669.13 | 99.85 | 176.17 | 623.82 | 33.30 | 2.77 | 5.84 | 8.47 | 62.05 | 2.99 | 0.00 | 719.35 | 14.05 | 10.55 | 1653.07 | 8.82 | 35.74 | 25.79 |
| 230294 | **NorCOMM2** | **Rock1** | **Rock1_tm1b_B11** | **+/-** | **F** | **230294** | **3/24/2015** | NA | 5.61 | 115.01 | 27.06 | 317.48 | 1401.90 | 106.82 | 12017.00 | 6599.30 | 3183.10 | 54.63 | 588.46 | 595.01 | 7313.30 | 15.44 | 5.22 | 109.79 | 1530.90 | 22736.00 | 38093.00 | 3837.90 | 254.54 | 411.30 | 31.96 | 2015.10 | 509.73 | 3.98 | 2761.30 | 0.00 | 4.03 | 12.52 | 21.20 | 11.77 | 85.27 | 3091.70 | 95.84 | 174.71 | 577.62 | 41.13 | 3.99 | 7.05 | 7.73 | 154.28 | 3.12 | 2.72 | 893.48 | 30.69 | 8.92 | 1172.30 | 6.95 | 41.37 | 25.11 |
| 230292 | **NorCOMM2** | **Rock1** | **Rock1_tm1b_B11** | **+/-** | **F** | **230292** | **3/24/2015** | NA | 7.20 | 125.47 | 26.19 | 255.05 | 610.31 | 78.96 | 14629.00 | 4132.20 | 2935.50 | 316.37 | 81.41 | 467.62 | 6882.00 | 22.34 | 2.69 | 91.64 | 1542.40 | 19082.00 | 16997.00 | 3619.00 | 163.26 | 259.25 | 16.46 | 1085.00 | 348.39 | 5.33 | 1911.90 | 0.00 | 1.02 | 12.64 | 36.61 | 3.19 | 46.92 | 3625.10 | 78.05 | 133.90 | 476.29 | 32.52 | 3.19 | 7.78 | 8.16 | 72.87 | 3.09 | 2.25 | 678.68 | 11.59 | 10.57 | 1016.60 | 7.20 | 33.60 | 19.69 |
| 230291 | **NorCOMM2** | **Rock1** | **Rock1_tm1b_B11** | **+/-** | **F** | **230291** | **3/25/2015** | NA | 4.68 | 92.83 | 21.85 | 275.77 | 869.28 | 50.67 | 11484.00 | 4751.00 | 2992.00 | 50.32 | 77.92 | 440.56 | 6849.70 | 17.86 | 3.72 | 104.67 | 1523.10 | 18896.00 | 29753.00 | 3057.60 | 182.42 | 332.13 | 25.42 | 1677.60 | 415.72 | 3.59 | 2206.10 | 0.00 | 3.80 | 9.97 | 34.09 | 8.58 | 83.00 | 3529.10 | 80.87 | 149.54 | 473.30 | 36.99 | 2.95 | 6.80 | 4.16 | 48.81 | 2.96 | 2.45 | 708.36 | 16.49 | 9.06 | 940.84 | 6.43 | 37.76 | 16.16 |
| 225684 | **NorCOMM2** | **Rock1** | **Rock1_tm1b_B11** | **+/-** | **M** | **225684** | **3/5/2015** | NA | 10.51 | 90.82 | 11.38 | 199.97 | 1439.50 | 51.73 | 28927.00 | 9005.60 | 3851.00 | 5225.90 | 0.00 | 463.63 | 9117.20 | 7.50 | 2.20 | 126.74 | 2123.20 | 33083.00 | 52411.00 | 3583.40 | 269.04 | 429.43 | 21.78 | 2015.10 | 514.11 | 0.00 | 2790.50 | 4.16 | 0.00 | 10.32 | 19.42 | 3.97 | 48.62 | 2048.30 | 127.24 | 199.48 | 599.34 | 25.22 | 2.95 | 8.76 | 8.00 | 78.68 | 4.74 | 4.62 | 941.68 | 15.22 | 9.12 | 2462.10 | 11.77 | 45.78 | 30.58 |
| 225682 | **NorCOMM2** | **Rock1** | **Rock1_tm1b_B11** | **+/-** | **M** | **225682** | **3/3/2015** | NA | 0.00 | 86.88 | 11.09 | 198.44 | 1689.30 | 25.04 | 32893.00 | 10344.00 | 3988.50 | 5891.30 | 0.00 | 561.47 | 8814.80 | 5.81 | 4.87 | 124.11 | 1890.00 | 37815.00 | 56105.00 | 3332.70 | 292.78 | 584.62 | 22.58 | 2811.30 | 578.67 | 10.85 | 3118.20 | 5.19 | 0.00 | 11.52 | 15.22 | 0.00 | 62.41 | 1953.50 | 160.04 | 209.98 | 762.02 | 24.87 | 3.11 | 7.39 | 10.50 | 64.47 | 3.04 | 2.98 | 1051.20 | 15.80 | 15.41 | 2332.10 | 12.08 | 45.24 | 33.67 |
| 225681 | **NorCOMM2** | **Rock1** | **Rock1_tm1b_B11** | **+/-** | **M** | **225681** | **3/3/2015** | NA | 7.56 | 78.57 | 9.97 | 179.36 | 1775.50 | 85.95 | 26654.00 | 7609.60 | 3710.50 | 4111.80 | 0.00 | 463.13 | 8386.30 | 0.00 | 2.47 | 108.35 | 1910.20 | 42827.00 | 48414.00 | 3544.40 | 271.52 | 606.24 | 20.00 | 1553.40 | 410.51 | 5.75 | 2361.80 | 6.50 | 0.00 | 14.32 | 79.65 | 3.55 | 52.34 | 1867.40 | 128.04 | 190.17 | 471.14 | 30.11 | 2.92 | 5.74 | 7.21 | 72.41 | 3.41 | 3.51 | 871.18 | 10.31 | 12.13 | 2187.70 | 13.98 | 40.66 | 32.39 |
| 35846 | **NorCOMM2** | **Sra1** | **Sra1_tm1b_C01** | **-/-** | **F** | **35846** | **11/28/2013** | NA | 7.73 | 133.35 | 29.58 | 350.47 | 847.03 | 64.99 | 16009.64 | 7721.27 | 4064.94 | 21.85 | 37.61 | 482.15 | 8617.43 | 20.93 | 7.57 | 109.51 | 2079.79 | 28289.06 | 48730.38 | 2863.52 | 203.22 | 364.26 | 27.01 | 1851.19 | 428.50 | 7.03 | 2535.74 | 3.91 | 9.38 | 12.13 | 12.82 | 48.09 | 168.55 | 3771.65 | 81.98 | 165.20 | 536.87 | 45.62 | 3.05 | 6.32 | 7.98 | 148.76 | 2.99 | 3.11 | 740.47 | 26.74 | 9.22 | 1436.02 | 4.39 | 37.49 | 25.12 |
| 39361 | **NorCOMM2** | **Sra1** | **Sra1_tm1b_C01** | **-/-** | **F** | **39361** | **12/11/2013** | NA | 12.21 | 96.45 | 17.62 | 313.09 | 1020.37 | 78.08 | 15413.53 | 7260.05 | 3281.20 | 97.14 | 227.04 | 417.05 | 7565.33 | 32.16 | 3.12 | 142.09 | 1827.18 | 33808.85 | 40148.26 | 2916.92 | 347.40 | 277.86 | 22.10 | 2179.96 | 474.31 | 4.30 | 2637.91 | 0.00 | 5.46 | 12.03 | 34.00 | 11.39 | 70.78 | 5217.81 | 76.78 | 162.11 | 513.30 | 48.24 | 4.40 | 6.51 | 9.82 | 88.29 | 4.65 | 4.17 | 917.60 | 23.24 | 7.29 | 1348.23 | 7.46 | 39.89 | 23.05 |
| 39360 | **NorCOMM2** | **Sra1** | **Sra1_tm1b_C01** | **-/-** | **F** | **39360** | **12/11/2013** | NA | 5.39 | 100.40 | 22.12 | 327.59 | 1437.31 | 89.25 | 18029.79 | 7324.75 | 3504.48 | 84.05 | 1053.32 | 475.50 | 8283.21 | 26.25 | 4.35 | 124.52 | 2351.08 | 30078.15 | 42006.94 | 3193.80 | 324.36 | 331.12 | 20.53 | 2789.06 | 479.51 | 5.15 | 3157.10 | 4.57 | 9.67 | 12.10 | 22.89 | 20.49 | 121.37 | 3658.92 | 80.62 | 171.24 | 675.55 | 59.88 | 4.12 | 7.38 | 13.71 | 102.53 | 3.49 | 2.29 | 870.67 | 30.58 | 11.65 | 1205.68 | 7.56 | 37.33 | 26.43 |
| 69285 | **NorCOMM2** | **Sra1** | **Sra1_tm1b_C01** | **-/-** | **M** | **69285** | **3/4/2014** | NA | 4.91 | 87.18 | 12.94 | 212.41 | 1728.10 | 33.49 | 27643.81 | 8313.61 | 3888.19 | 4329.34 | 0.00 | 559.89 | 8409.42 | 6.98 | 4.81 | 128.92 | 1779.40 | 32839.67 | 48723.48 | 3579.58 | 195.41 | 344.14 | 27.36 | 2419.97 | 532.13 | 4.92 | 2570.07 | 3.26 | 4.74 | 11.10 | 11.88 | 13.74 | 118.15 | 3045.60 | 121.68 | 177.63 | 595.05 | 32.07 | 4.21 | 7.20 | 9.03 | 74.33 | 2.60 | 4.19 | 823.00 | 17.56 | 10.36 | 1864.59 | 12.73 | 35.27 | 27.18 |
| 35950 | **NorCOMM2** | **Sra1** | **Sra1_tm1b_C01** | **-/-** | **M** | **35950** | **11/28/2013** | NA | 13.73 | 79.28 | 14.68 | 214.35 | 1462.18 | 18.80 | 28077.34 | 7574.95 | 3544.74 | 4182.91 | 0.00 | 396.42 | 9376.21 | 7.17 | 3.90 | 97.72 | 2009.05 | 29966.74 | 44507.88 | 3239.51 | 193.01 | 453.54 | 17.88 | 1666.43 | 535.25 | 3.85 | 2296.65 | 4.27 | 5.16 | 11.89 | 15.54 | 18.05 | 100.30 | 2120.41 | 155.07 | 184.18 | 678.25 | 33.02 | 3.62 | 4.42 | 6.99 | 128.21 | 2.89 | 4.26 | 860.48 | 16.17 | 14.95 | 1927.71 | 9.02 | 26.47 | 24.12 |
| 35948 | **NorCOMM2** | **Sra1** | **Sra1_tm1b_C01** | **-/-** | **M** | **35948** | **11/28/2013** | NA | 7.25 | 126.67 | 23.60 | 304.20 | 918.74 | 67.62 | 14611.70 | 6364.54 | 3477.74 | 63.69 | 654.20 | 459.31 | 7814.44 | 14.11 | 6.90 | 88.34 | 2054.60 | 23291.72 | 40626.74 | 2834.38 | 298.05 | 335.00 | 24.25 | 1898.01 | 474.30 | 4.92 | 2508.60 | 6.88 | 8.16 | 14.90 | 43.50 | 19.50 | 133.98 | 3473.80 | 70.33 | 163.61 | 534.38 | 41.87 | 3.41 | 6.36 | 7.06 | 68.31 | 3.16 | 2.28 | 662.62 | 22.39 | 11.28 | 1100.87 | 7.24 | 35.99 | 23.39 |
| 487268 | **K2P2** | **Ulk3** | **Ulk3_em2_del** | **-/-** | **F** | **487268** | **1/4/2017** | NA | 6.84 | 94.62 | 20.75 | 229.20 | 1115.04 | 46.91 | 14764.66 | 6491.31 | 3230.95 | 88.52 | 814.90 | 397.05 | 6217.43 | 15.50 | 2.27 | 140.68 | 2333.34 | 27903.75 | 40169.75 | 3355.66 | 296.18 | 492.47 | 22.87 | 2155.77 | 543.84 | 3.28 | 2623.24 | 0.00 | 0.00 | 11.01 | 30.44 | 0.00 | 41.77 | 5627.17 | 92.11 | 155.08 | 508.80 | 49.39 | 3.28 | 5.27 | 11.70 | 116.30 | 3.66 | 0.00 | 813.28 | 25.57 | 9.62 | 866.40 | 6.05 | 44.06 | 28.21 |
| 487251 | **K2P2** | **Ulk3** | **Ulk3_em2_del** | **-/-** | **F** | **487251** | **12/29/2016** | NA | 3.72 | 74.21 | 21.72 | 332.59 | 1184.10 | 37.14 | 13080.97 | 5674.58 | 2608.01 | 35.72 | 1528.90 | 397.95 | 6051.24 | 20.16 | 2.00 | 127.74 | 2142.35 | 22894.55 | 40541.90 | 2553.85 | 226.18 | 343.67 | 22.41 | 3425.92 | 411.37 | 3.87 | 2768.09 | 0.00 | 1.70 | 8.25 | 28.92 | 5.71 | 61.89 | 3503.72 | 81.67 | 159.85 | 508.32 | 51.60 | 3.30 | 4.67 | 14.56 | 76.90 | 4.18 | 0.00 | 854.62 | 22.83 | 7.45 | 975.66 | 6.73 | 41.61 | 26.93 |
| 487266 | **K2P2** | **Ulk3** | **Ulk3_em2_del** | **-/-** | **F** | **487266** | **1/4/2017** | NA | 3.98 | 116.19 | 21.61 | 262.27 | 1321.80 | 46.74 | 16599.28 | 6495.57 | 2691.70 | 211.41 | 1462.65 | 478.22 | 6130.79 | 12.95 | 2.20 | 145.28 | 1759.62 | 31058.62 | 35059.32 | 2974.32 | 277.52 | 367.69 | 19.18 | 4422.40 | 556.41 | 4.03 | 2776.96 | 3.52 | 2.10 | 11.55 | 40.00 | 3.16 | 61.41 | 4644.31 | 84.14 | 159.05 | 525.75 | 44.76 | 5.38 | 4.89 | 11.84 | 77.02 | 4.88 | 2.91 | 981.65 | 20.79 | 9.09 | 925.70 | 9.09 | 42.99 | 21.90 |
| 487154 | **K2P2** | **Ulk3** | **Ulk3_em2_del** | **-/-** | **M** | **487154** | **1/4/2017** | NA | 9.00 | 131.39 | 9.09 | 114.99 | 3168.71 | 556.29 | 27705.42 | 7800.50 | 2812.77 | 4217.92 | 0.00 | 391.62 | 6774.09 | 0.00 | 5.26 | 124.00 | 1969.04 | 29725.73 | 40612.98 | 3416.49 | 219.56 | 362.20 | 23.09 | 2641.60 | 526.21 | 3.43 | 2544.82 | 5.25 | 0.00 | 14.63 | 17.13 | 5.38 | 56.49 | 2487.78 | 153.53 | 158.33 | 585.09 | 27.54 | 4.14 | 6.69 | 11.47 | 130.71 | 4.71 | 4.71 | 954.85 | 16.82 | 13.12 | 2280.80 | 6.46 | 38.10 | 24.47 |
| 487149 | **K2P2** | **Ulk3** | **Ulk3_em2_del** | **-/-** | **M** | **487149** | **12/29/2016** | NA | 9.90 | 85.70 | 9.16 | 104.71 | 3600.30 | 1356.66 | 26864.11 | 6474.00 | 2645.86 | 4944.97 | 0.00 | 476.20 | 7514.95 | 0.00 | 1.72 | 150.44 | 2300.64 | 34978.47 | 39580.43 | 3374.64 | 406.35 | 491.72 | 24.90 | 2506.12 | 589.17 | 6.04 | 3005.56 | 0.00 | 0.00 | 16.10 | 16.40 | 0.00 | 42.75 | 3064.19 | 251.05 | 176.40 | 555.80 | 23.41 | 4.00 | 7.35 | 9.78 | 113.58 | 3.94 | 5.74 | 1250.35 | 16.75 | 10.75 | 2273.88 | 10.93 | 38.79 | 35.01 |
| 487163 | **K2P2** | **Ulk3** | **Ulk3_em2_del** | **-/-** | **M** | **487163** | **1/4/2017** | NA | 7.20 | 108.78 | 5.28 | 142.22 | 4309.25 | 1613.47 | 23255.90 | 6734.15 | 2662.69 | 4448.67 | 0.00 | 549.28 | 7447.31 | 0.00 | 3.67 | 135.48 | 2231.61 | 31011.13 | 40571.52 | 3835.60 | 333.61 | 543.10 | 26.43 | 2592.27 | 555.27 | 3.91 | 2751.59 | 3.70 | 0.00 | 19.07 | 14.38 | 4.82 | 55.07 | 2278.75 | 178.29 | 172.12 | 551.49 | 26.61 | 3.90 | 7.15 | 12.31 | 96.12 | 4.44 | 4.78 | 1129.34 | 15.98 | 13.75 | 3066.92 | 12.12 | 56.56 | 32.88 |
| 284228 | **NorCOMM2** | **Ywhaz** | **Ywhaz_tm1b_A11** | **+/-** | **F** | **284228** | **6/30/2015** | NA | 6.45 | 108.13 | 16.58 | 283.93 | 834.89 | 62.31 | 17179.00 | 7017.90 | 3399.70 | 413.50 | 56.82 | 471.14 | 8651.60 | 24.24 | 2.22 | 103.00 | 2271.00 | 26406.00 | 42554.00 | 2870.20 | 304.40 | 298.92 | 14.27 | 2671.70 | 372.76 | 3.41 | 2597.80 | 2.64 | 0.00 | 11.66 | 37.77 | 3.13 | 55.79 | 3913.90 | 88.45 | 181.75 | 558.00 | 42.98 | 2.86 | 6.09 | 9.79 | 126.18 | 4.64 | 2.32 | 977.14 | 27.09 | 12.03 | 1513.00 | 7.34 | 52.00 | 32.48 |
| 294641 | **NorCOMM2** | **Ywhaz** | **Ywhaz_tm1b_A11** | **+/-** | **F** | **294641** | **7/14/2015** | NA | 5.83 | 72.16 | 23.45 | 311.66 | 1195.70 | 55.19 | 14258.00 | 7203.50 | 3141.30 | 557.32 | 50.97 | 508.97 | 8674.00 | 22.62 | 1.92 | 91.56 | 2151.80 | 22133.00 | 36857.00 | 3170.70 | 419.25 | 424.60 | 31.16 | 2651.10 | 442.57 | 4.21 | 2728.90 | 6.11 | 1.08 | 11.93 | 17.94 | 3.00 | 54.97 | 3807.70 | 91.49 | 170.48 | 595.72 | 39.25 | 3.27 | 4.45 | 9.58 | 75.15 | 3.70 | 2.85 | 817.37 | 22.41 | 15.36 | 1091.50 | 8.14 | 31.32 | 22.19 |
| 294642 | **NorCOMM2** | **Ywhaz** | **Ywhaz_tm1b_A11** | **+/-** | **F** | **294642** | **7/14/2015** | NA | 3.94 | 93.82 | 22.14 | 226.92 | 764.81 | 108.35 | 10389.00 | 6680.50 | 3015.20 | 34.71 | 2.49 | 400.52 | 7280.10 | 47.54 | 2.73 | 79.80 | 1426.90 | 26825.00 | 26366.00 | 3207.10 | 203.94 | 386.47 | 10.66 | 1463.40 | 402.06 | 4.28 | 2218.00 | 4.84 | 0.00 | 13.73 | 9.98 | 3.46 | 71.66 | 3323.90 | 65.97 | 135.92 | 387.81 | 30.91 | 2.18 | 6.13 | 3.51 | 51.38 | 2.41 | 2.86 | 711.20 | 24.43 | 7.08 | 1089.50 | 5.33 | 28.58 | 15.82 |
| 295044 | **NorCOMM2** | **Ywhaz** | **Ywhaz_tm1b_A11** | **+/-** | **M** | **295044** | **7/16/2015** | NA | 9.47 | 96.75 | 11.51 | 238.71 | 1561.00 | 28.07 | 34707.00 | 8720.40 | 4740.40 | 6176.80 | 0.00 | 526.52 | 10096.00 | 6.52 | 2.93 | 87.68 | 1688.00 | 34711.00 | 40515.00 | 4369.10 | 279.49 | 700.38 | 15.22 | 2028.60 | 608.15 | 7.17 | 2963.10 | 5.12 | 0.00 | 11.96 | 20.37 | 5.24 | 64.28 | 2572.60 | 152.67 | 208.41 | 721.63 | 30.86 | 3.94 | 7.99 | 11.01 | 72.95 | 5.92 | 3.88 | 852.05 | 16.83 | 14.76 | 2198.00 | 10.28 | 39.39 | 26.07 |
| 295043 | **NorCOMM2** | **Ywhaz** | **Ywhaz_tm1b_A11** | **+/-** | **M** | **295043** | **7/16/2015** | NA | 13.64 | 157.14 | 12.61 | 213.68 | 2416.30 | 220.68 | 30535.00 | 8584.50 | 3680.90 | 6970.70 | 0.00 | 665.61 | 9913.90 | 5.85 | 3.27 | 103.83 | 2154.00 | 39688.00 | 44791.00 | 3798.50 | 332.00 | 644.72 | 28.53 | 2252.50 | 583.69 | 4.87 | 2799.00 | 3.79 | 0.00 | 16.74 | 117.48 | 3.09 | 58.01 | 2217.50 | 168.65 | 201.79 | 773.23 | 28.47 | 4.18 | 9.95 | 8.36 | 70.75 | 5.55 | 3.67 | 1033.50 | 14.79 | 13.58 | 2246.90 | 11.57 | 44.80 | 33.38 |
| 295040 | **NorCOMM2** | **Ywhaz** | **Ywhaz_tm1b_A11** | **+/-** | **M** | **295040** | **7/16/2015** | NA | 19.34 | 135.77 | 8.73 | 169.18 | 7553.10 | 3848.80 | 27218.00 | 6960.20 | 3549.60 | 4802.20 | 0.00 | 856.45 | 11379.00 | 0.00 | 4.41 | 147.94 | 1978.50 | 30125.00 | 37803.00 | 3248.20 | 453.00 | 613.88 | 40.37 | 1956.20 | 557.21 | 3.67 | 2729.40 | 0.00 | 1.50 | 24.31 | 17.61 | 3.59 | 65.01 | 1962.70 | 290.82 | 223.73 | 628.35 | 20.35 | 4.24 | 7.92 | 9.79 | 76.79 | 5.98 | 4.01 | 1990.70 | 18.34 | 14.76 | 4023.00 | 16.45 | 49.47 | 37.98 |

| Sample | Project | Gene symbol | Mouse line name | Zygosity | Gender | Mouse BCode | date collected | F10 | F12 | F13a1 | F13b | Cfl1 | Colec11 | C1qa | C1qb | C1qc | C1sa | C3 | C4b | C5 | C8a | C8b | C8g | C9 | Cfh | Cfi | Serpina6 | Crp | Cst3 | Cycs | Lap3 | Ctbs | Dag1 | Efemp1 | Eef1a1 | Egfr | Ecm1 | Sod3 | Fetub | Fga | Fgb | Fgg | Fgl1 | Fn1 | Fcn1 | Blvrb | Fstl1 | Aldob | Aldoa | Fah | Ggh | Gsn | Gpx3 | Gstm1 | Glycam1 | H2-Q10 | Hp | Hba | Hbb-b1 |
| --- | --- | --- | --- | --- | --- | --- | --- | --- | --- | --- | --- | --- | --- | --- | --- | --- | --- | --- | --- | --- | --- | --- | --- | --- | --- | --- | --- | --- | --- | --- | --- | --- | --- | --- | --- | --- | --- | --- | --- | --- | --- | --- | --- | --- | --- | --- | --- | --- | --- | --- | --- | --- | --- | --- | --- | --- | --- | --- | --- |
| 448371 | KOMP2 | (null) | C57BL/6NCrl | +/+ | F | 448371 | 10/12/2016 | 236.36 | 711.23 | 201.47 | 47.40 | 9.04 | 12.46 | 14.78 | 324.78 | 280.47 | 102.33 | 1680.71 | 631.59 | 30.32 | 136.47 | 161.76 | 146.35 | 273.55 | 28.34 | 515.74 | 1205.17 | 93.31 | 29.02 | 12.35 | 1.41 | 10.92 | 0.88 | 75.20 | 0.00 | 17.37 | 40.54 | 42.41 | 704.81 | 8452.29 | 7543.39 | 6917.74 | 2.01 | 250.76 | 141.35 | 3.32 | 2.16 | 2.91 | 22.72 | 0.00 | 5.79 | 1211.24 | 466.86 | 0.00 | 5.50 | 383.08 | 27.94 | 1582.27 | 691.63 |
| 115863 | NorCOMM2 | (null) | C57BL/6NCrl | +/+ | F | 115863 | 6/5/2014 | 223.11 | 572.93 | 129.42 | 25.16 | 15.11 | 9.06 | 14.05 | 391.21 | 262.53 | 93.28 | 1887.01 | 463.87 | 23.89 | 126.49 | 144.01 | 130.11 | 173.65 | 33.16 | 445.94 | 1556.67 | 100.76 | 42.49 | 9.52 | 1.12 | 11.42 | 1.21 | 81.94 | 8.26 | 14.26 | 35.83 | 61.82 | 871.39 | 6495.56 | 4474.42 | 4679.82 | 1.48 | 29.26 | 119.07 | 11.24 | 1.97 | 4.41 | 28.93 | 1.76 | 5.28 | 1153.01 | 653.35 | 7.52 | 7.01 | 380.46 | 9.68 | 13785.97 | 5703.10 |
| 432638 | KOMP2 | (null) | C57BL/6NCrl | +/+ | F | 432638 | 9/1/2016 | 153.71 | 513.65 | 110.32 | 22.48 | 3.29 | 6.00 | 3.11 | 62.27 | 41.94 | 56.95 | 1324.81 | 412.10 | 21.60 | 111.64 | 110.60 | 105.94 | 165.17 | 27.04 | 373.13 | 1094.41 | 75.48 | 28.52 | 7.85 | 0.94 | 9.22 | 0.78 | 50.20 | 0.00 | 13.75 | 31.86 | 45.97 | 807.75 | 6807.71 | 5069.97 | 5440.17 | 1.55 | 203.93 | 99.36 | 2.37 | 1.23 | 0.00 | 10.39 | 0.00 | 5.01 | 927.45 | 487.13 | 0.00 | 4.60 | 291.62 | 16.98 | 1413.45 | 503.18 |
| 237715 | NorCOMM2 | (null) | C57BL/6NCrl | +/+ | F | 237715 | 4/21/2015 | 196.50 | 568.86 | 152.73 | 41.60 | 6.53 | 6.35 | 8.50 | 278.23 | 212.67 | 87.59 | 1735.74 | 358.84 | 23.70 | 141.37 | 133.53 | 138.61 | 217.13 | 20.21 | 416.55 | 1230.21 | 121.21 | 27.39 | 10.26 | 1.31 | 11.19 | 1.08 | 76.37 | 0.95 | 12.51 | 39.51 | 46.05 | 712.49 | 7618.68 | 4594.11 | 4968.20 | 1.78 | 81.03 | 110.62 | 6.38 | 1.62 | 0.00 | 19.64 | 0.00 | 4.66 | 1032.24 | 675.70 | 1.89 | 5.02 | 331.94 | 70.23 | 6639.24 | 2765.43 |
| 496095 | K2P2 | (null) | C57BL/6NCrl | +/+ | F | 496095 | 2/14/2017 | 232.26 | 785.08 | 170.84 | 34.74 | 8.06 | 9.63 | 8.56 | 243.32 | 136.99 | 68.26 | 1473.13 | 577.20 | 26.72 | 152.76 | 132.89 | 125.10 | 249.73 | 30.50 | 502.05 | 1501.34 | 92.08 | 27.15 | 9.47 | 0.91 | 10.97 | 1.08 | 67.44 | 0.00 | 10.99 | 38.35 | 51.53 | 755.28 | 9989.09 | 6933.03 | 7293.95 | 1.81 | 158.37 | 140.30 | 1.96 | 2.20 | 3.91 | 21.99 | 0.00 | 5.01 | 1379.92 | 552.60 | 0.00 | 8.24 | 416.21 | 67.88 | 2138.53 | 781.35 |
| 229694 | NorCOMM2 | (null) | C57BL/6NCrl | +/+ | F | 229694 | 3/26/2015 | 229.24 | 669.12 | 156.54 | 39.72 | 8.97 | 7.17 | 10.76 | 456.71 | 267.42 | 78.76 | 1841.69 | 424.46 | 21.92 | 175.51 | 170.39 | 148.12 | 188.61 | 26.60 | 366.58 | 1351.31 | 99.69 | 26.20 | 9.90 | 1.00 | 11.12 | 1.06 | 57.70 | 0.00 | 18.95 | 37.53 | 53.32 | 741.36 | 5601.27 | 4052.66 | 3454.05 | 1.53 | 45.30 | 138.63 | 9.28 | 1.62 | 0.00 | 21.71 | 0.00 | 5.83 | 1156.94 | 566.82 | 1.74 | 4.73 | 348.88 | 37.18 | 10097.21 | 3367.87 |
| 417794 | KOMP2 | (null) | C57BL/6NCrl | +/+ | F | 417794 | 7/21/2016 | 191.67 | 599.29 | 167.35 | 48.02 | 7.20 | 11.86 | 13.51 | 377.92 | 322.87 | 88.43 | 1769.00 | 534.77 | 27.39 | 202.64 | 211.64 | 172.44 | 254.88 | 25.45 | 505.06 | 1471.68 | 105.34 | 19.41 | 10.32 | 1.30 | 11.50 | 1.05 | 90.92 | 0.00 | 18.72 | 39.08 | 41.65 | 709.13 | 7262.88 | 5644.15 | 6981.38 | 1.65 | 196.18 | 138.26 | 2.69 | 2.12 | 0.00 | 13.57 | 0.00 | 5.64 | 1256.60 | 612.08 | 0.00 | 7.48 | 387.02 | 21.01 | 1179.42 | 586.50 |
| 220169 | NorCOMM2 | (null) | C57BL/6NCrl | +/+ | F | 220169 | 2/12/2015 | 226.14 | 624.46 | 166.80 | 37.81 | 5.64 | 11.18 | 10.85 | 365.23 | 286.44 | 98.30 | 1708.39 | 474.75 | 29.28 | 124.18 | 115.03 | 96.45 | 207.76 | 20.89 | 483.27 | 1673.96 | 108.32 | 35.97 | 14.29 | 1.11 | 10.58 | 1.00 | 72.17 | 1.09 | 14.60 | 33.98 | 54.52 | 778.66 | 5902.05 | 4859.96 | 5997.25 | 1.59 | 155.55 | 107.91 | 6.44 | 1.82 | 4.12 | 13.48 | 3.03 | 4.88 | 1246.34 | 734.23 | 0.80 | 6.04 | 386.67 | 17.59 | 3918.89 | 1376.39 |
| 170955 | NorCOMM2 | (null) | C57BL/6NCrl | +/+ | F | 170955 | 10/9/2014 | 188.76 | 663.74 | 164.24 | 48.55 | 7.47 | 10.86 | 12.72 | 350.05 | 238.25 | 80.17 | 1915.42 | 472.88 | 32.15 | 122.51 | 131.67 | 95.63 | 226.66 | 25.45 | 457.25 | 1292.76 | 116.68 | 51.05 | 14.48 | 1.21 | 12.25 | 1.01 | 71.18 | 3.26 | 17.29 | 30.01 | 42.65 | 788.05 | 6821.54 | 6804.19 | 8264.23 | 1.67 | 129.73 | 123.82 | 11.10 | 1.93 | 2.55 | 16.47 | 2.37 | 7.27 | 1337.41 | 696.75 | 1.27 | 5.87 | 492.59 | 35.74 | 7675.91 | 2918.48 |
| 319739 | NorCOMM2 | (null) | C57BL/6NCrl | +/+ | F | 319739 | 9/17/2015 | 278.82 | 644.54 | 172.63 | 33.93 | 3.21 | 10.53 | 10.46 | 426.08 | 298.45 | 102.83 | 1960.31 | 476.00 | 31.08 | 167.62 | 157.64 | 97.23 | 216.74 | 22.94 | 521.54 | 1496.34 | 110.09 | 35.51 | 16.66 | 1.09 | 11.97 | 1.15 | 98.83 | 0.00 | 17.58 | 30.49 | 48.17 | 797.89 | 8818.79 | 5541.17 | 5752.07 | 1.84 | 186.51 | 110.03 | 2.59 | 1.85 | 4.27 | 15.02 | 0.00 | 7.51 | 1204.31 | 740.19 | 1.23 | 6.09 | 541.86 | 60.14 | 1523.75 | 548.31 |
| 198415 | NorCOMM2 | (null) | C57BL/6NCrl | +/+ | F | 198415 | 12/23/2014 | 177.74 | 601.46 | 156.45 | 30.25 | 4.10 | 8.00 | 12.97 | 274.16 | 192.31 | 80.68 | 1717.56 | 422.33 | 22.76 | 161.80 | 209.90 | 179.38 | 198.38 | 23.20 | 423.01 | 1300.07 | 100.66 | 31.54 | 12.19 | 0.98 | 9.70 | 0.92 | 70.18 | 1.23 | 20.85 | 36.85 | 38.20 | 696.72 | 7522.31 | 5368.83 | 7206.94 | 2.06 | 161.05 | 123.78 | 2.42 | 1.59 | 2.76 | 14.28 | 0.00 | 4.77 | 1181.65 | 561.11 | 1.65 | 5.66 | 422.08 | 162.15 | 1342.83 | 459.33 |
| 296903 | NorCOMM2 | (null) | C57BL/6NCrl | +/+ | F | 296903 | 7/21/2015 | 216.47 | 645.20 | 126.68 | 30.28 | 4.64 | 7.81 | 8.21 | 253.18 | 194.28 | 70.08 | 1191.15 | 463.74 | 26.24 | 152.56 | 220.26 | 147.84 | 289.36 | 18.73 | 566.23 | 1788.98 | 92.41 | 28.27 | 15.89 | 1.40 | 10.88 | 1.15 | 77.75 | 0.00 | 8.62 | 45.10 | 59.65 | 716.01 | 6649.50 | 4748.77 | 4316.57 | 1.00 | 75.56 | 105.80 | 4.44 | 2.18 | 4.83 | 20.56 | 1.84 | 4.52 | 1364.22 | 672.84 | 0.99 | 7.67 | 385.54 | 23.73 | 4204.05 | 1445.78 |
| 38540 | NorCOMM2 | (null) | C57BL/6NCrl | +/+ | F | 38540 | 12/11/2013 | 167.08 | 560.24 | 174.29 | 37.30 | 5.65 | 11.12 | 8.11 | 252.00 | 176.77 | 67.04 | 1113.57 | 457.18 | 25.70 | 104.31 | 139.91 | 114.14 | 172.55 | 17.42 | 469.99 | 1507.90 | 85.98 | 37.41 | 10.85 | 1.09 | 12.23 | 1.00 | 77.79 | 3.00 | 19.39 | 35.74 | 34.86 | 697.19 | 7897.19 | 5219.89 | 5275.40 | 1.60 | 169.04 | 125.96 | 7.22 | 1.97 | 0.00 | 11.23 | 2.04 | 4.78 | 1218.49 | 498.72 | 2.07 | 6.85 | 442.31 | 19.05 | 8992.00 | 3108.02 |
| 349028 | NorCOMM2 | (null) | C57BL/6NCrl | +/+ | F | 349028 | 11/5/2015 | 219.14 | 660.17 | 84.57 | 35.05 | 3.80 | 6.15 | 11.42 | 460.68 | 229.09 | 79.44 | 1619.30 | 471.72 | 27.85 | 133.44 | 161.18 | 127.18 | 219.87 | 28.63 | 411.86 | 1604.50 | 98.47 | 33.83 | 15.65 | 0.97 | 12.47 | 0.90 | 61.93 | 2.94 | 17.41 | 27.88 | 43.93 | 719.92 | 3299.20 | 2303.20 | 2777.00 | 1.22 | 30.38 | 108.18 | 10.22 | 1.94 | 4.46 | 12.83 | 2.48 | 4.26 | 1562.90 | 518.08 | 1.18 | 5.27 | 329.09 | 140.16 | 8374.20 | 3320.00 |
| 38537 | NorCOMM2 | (null) | C57BL/6NCrl | +/+ | F | 38537 | 12/11/2013 | 215.42 | 822.26 | 138.66 | 44.54 | 2.43 | 11.67 | 13.11 | 305.52 | 240.47 | 75.57 | 1532.10 | 397.47 | 26.73 | 133.51 | 213.26 | 122.12 | 216.12 | 23.40 | 402.29 | 1488.10 | 111.40 | 37.80 | 12.66 | 0.95 | 12.18 | 1.10 | 69.30 | 0.00 | 21.48 | 30.61 | 34.65 | 709.38 | 5679.00 | 3859.00 | 2925.10 | 1.37 | 27.66 | 90.43 | 7.05 | 2.03 | 2.91 | 11.75 | 3.11 | 5.90 | 998.02 | 481.24 | 1.09 | 6.36 | 486.69 | 31.68 | 5254.40 | 1914.80 |
| 55856 | NorCOMM2 | (null) | C57BL/6NCrl | +/+ | F | 55856 | 1/22/2014 | 203.20 | 741.43 | 115.77 | 56.61 | 4.26 | 9.81 | 22.62 | 240.99 | 237.91 | 89.95 | 2090.40 | 555.52 | 25.27 | 87.75 | 161.22 | 95.57 | 159.49 | 20.88 | 422.84 | 1804.50 | 90.64 | 41.95 | 17.34 | 1.12 | 16.30 | 1.03 | 61.57 | 3.78 | 13.23 | 29.26 | 43.47 | 835.05 | 7373.00 | 4259.10 | 4223.90 | 2.22 | 70.12 | 147.18 | 7.11 | 1.87 | 4.34 | 22.30 | 0.00 | 5.66 | 1227.40 | 564.16 | 2.04 | 6.31 | 506.91 | 19.19 | 10675.00 | 3876.40 |
| 144712 | NorCOMM2 | (null) | C57BL/6NCrl | +/+ | F | 144712 | 8/7/2014 | 226.28 | 730.57 | 114.81 | 33.94 | 2.02 | 5.47 | 14.34 | 305.20 | 178.65 | 57.67 | 1395.00 | 540.02 | 28.82 | 98.19 | 128.18 | 98.33 | 171.61 | 17.64 | 478.66 | 1869.90 | 91.48 | 43.75 | 15.82 | 1.16 | 13.04 | 0.74 | 76.77 | 0.00 | 14.04 | 28.13 | 44.12 | 752.88 | 5337.90 | 4265.80 | 3326.10 | 1.23 | 33.25 | 120.11 | 7.21 | 1.68 | 4.30 | 14.98 | 0.00 | 6.33 | 1188.10 | 608.89 | 1.56 | 4.52 | 372.23 | 17.93 | 6256.90 | 2224.20 |
| 257642 | NorCOMM2 | (null) | C57BL/6NCrl | +/+ | F | 257642 | 5/21/2015 | 169.84 | 632.12 | 181.38 | 32.92 | 3.22 | 8.90 | 11.92 | 273.05 | 111.46 | 79.32 | 1406.50 | 509.50 | 25.42 | 61.78 | 111.72 | 70.29 | 172.88 | 13.06 | 355.73 | 2060.90 | 91.30 | 23.86 | 16.47 | 1.21 | 11.70 | 0.83 | 63.57 | 0.00 | 9.74 | 25.32 | 52.15 | 722.40 | 7419.00 | 4762.10 | 5052.90 | 2.15 | 97.12 | 111.37 | 4.28 | 1.59 | 3.64 | 18.29 | 0.00 | 7.53 | 1513.90 | 636.26 | 1.08 | 8.37 | 494.13 | 18.15 | 3438.00 | 1445.40 |
| 209240 | NorCOMM2 | (null) | C57BL/6NCrl | +/+ | F | 209240 | 1/13/2015 | 218.37 | 710.56 | 139.54 | 49.70 | 6.59 | 5.71 | 18.37 | 396.20 | 267.49 | 75.45 | 1539.70 | 460.29 | 27.41 | 103.29 | 124.14 | 128.15 | 209.12 | 19.06 | 391.26 | 1434.70 | 101.11 | 33.25 | 17.04 | 1.18 | 12.62 | 1.07 | 86.97 | 1.53 | 14.17 | 37.35 | 41.54 | 706.16 | 7052.70 | 4302.70 | 4865.60 | 1.54 | 173.59 | 109.61 | 2.44 | 1.82 | 4.04 | 22.39 | 2.24 | 6.11 | 1349.80 | 542.26 | 1.51 | 4.06 | 412.27 | 60.87 | 1045.40 | 400.15 |
| 410682 | KOMP2 | (null) | C57BL/6NCrl | +/+ | M | 410682 | 6/16/2016 | 239.31 | 741.61 | 115.08 | 34.62 | 6.12 | 9.74 | 10.68 | 504.59 | 306.54 | 119.80 | 1045.62 | 908.16 | 72.51 | 635.39 | 644.03 | 674.88 | 1361.85 | 34.13 | 510.35 | 558.47 | 138.26 | 26.53 | 10.61 | 0.84 | 10.23 | 1.10 | 82.14 | 0.00 | 51.37 | 32.26 | 34.34 | 1042.75 | 9198.38 | 8761.56 | 8099.02 | 1.35 | 111.87 | 149.49 | 3.86 | 1.98 | 4.25 | 21.78 | 0.00 | 5.25 | 1187.08 | 606.29 | 1.76 | 0.00 | 675.99 | 3616.61 | 2536.10 | 953.94 |
| 307708 | NorCOMM2 | (null) | C57BL/6NCrl | +/+ | M | 307708 | 8/18/2015 | 221.02 | 744.49 | 124.43 | 22.32 | 5.41 | 11.15 | 8.44 | 574.34 | 297.46 | 132.68 | 1215.04 | 782.17 | 54.06 | 488.92 | 615.30 | 539.87 | 755.38 | 31.69 | 478.13 | 717.04 | 112.93 | 34.81 | 9.48 | 1.21 | 11.57 | 1.39 | 94.47 | 0.00 | 27.97 | 24.63 | 39.45 | 742.95 | 6694.75 | 6470.07 | 5913.50 | 1.31 | 73.76 | 174.60 | 2.77 | 1.59 | 3.83 | 16.57 | 2.70 | 4.58 | 1253.46 | 685.49 | 0.00 | 1.08 | 808.70 | 450.58 | 1741.75 | 737.61 |
| 441143 | KOMP2 | (null) | C57BL/6NCrl | +/+ | M | 441143 | 9/22/2016 | 244.12 | 724.28 | 126.98 | 27.19 | 2.62 | 7.30 | 8.34 | 381.47 | 221.47 | 112.03 | 928.29 | 612.83 | 51.61 | 388.64 | 416.00 | 433.13 | 896.11 | 30.39 | 537.72 | 652.97 | 102.15 | 27.33 | 9.04 | 0.76 | 10.56 | 1.03 | 49.57 | 0.00 | 39.68 | 34.88 | 47.95 | 669.08 | 13538.88 | 7271.39 | 9092.59 | 1.08 | 166.03 | 95.09 | 5.65 | 2.04 | 5.13 | 11.32 | 0.00 | 4.22 | 1136.22 | 673.23 | 0.00 | 0.81 | 743.08 | 772.71 | 3855.37 | 1459.90 |
| 237609 | NorCOMM2 | (null) | C57BL/6NCrl | +/+ | M | 237609 | 4/23/2015 | 197.67 | 759.08 | 121.50 | 17.39 | 4.27 | 7.30 | 8.31 | 382.76 | 298.43 | 98.88 | 925.88 | 684.32 | 50.58 | 690.92 | 682.44 | 598.85 | 1156.66 | 29.89 | 448.98 | 471.82 | 111.55 | 26.97 | 9.76 | 0.87 | 14.49 | 1.08 | 57.82 | 1.76 | 30.33 | 26.26 | 33.76 | 683.21 | 7637.11 | 4357.96 | 4749.19 | 1.11 | 96.61 | 173.96 | 5.58 | 1.73 | 0.00 | 20.88 | 1.40 | 4.29 | 1076.43 | 635.02 | 1.19 | 0.88 | 744.03 | 452.88 | 5320.40 | 2275.54 |
| 510072 | K2P2 | (null) | C57BL/6NCrl | +/+ | M | 510072 | 4/19/2017 | 214.66 | 868.64 | 163.17 | 36.89 | 3.89 | 8.91 | 15.33 | 408.99 | 260.77 | 117.49 | 1345.71 | 777.22 | 60.67 | 697.39 | 759.34 | 512.28 | 1015.44 | 28.70 | 378.19 | 711.47 | 119.18 | 33.11 | 10.27 | 1.00 | 9.79 | 0.86 | 58.88 | 0.00 | 27.95 | 31.78 | 42.49 | 883.10 | 10996.23 | 7775.76 | 8945.59 | 1.56 | 260.65 | 132.41 | 2.24 | 1.63 | 4.92 | 16.13 | 1.60 | 5.12 | 1243.84 | 781.01 | 0.00 | 0.87 | 801.34 | 85.32 | 1040.62 | 516.85 |
| 198312 | NorCOMM2 | (null) | C57BL/6NCrl | +/+ | M | 198312 | 12/23/2014 | 259.35 | 653.03 | 120.95 | 29.50 | 6.22 | 6.55 | 17.89 | 376.37 | 306.83 | 116.62 | 1135.19 | 698.84 | 45.84 | 432.58 | 492.35 | 534.05 | 837.33 | 37.32 | 332.51 | 779.55 | 109.40 | 33.10 | 10.78 | 1.46 | 11.67 | 0.82 | 68.19 | 2.87 | 26.32 | 28.46 | 47.19 | 823.00 | 7188.73 | 7264.15 | 6774.75 | 1.36 | 83.52 | 154.45 | 10.56 | 1.51 | 7.83 | 27.48 | 2.56 | 5.35 | 1025.96 | 584.32 | 2.94 | 0.75 | 718.31 | 393.74 | 7097.54 | 3197.35 |
| 483043 | K2P2 | (null) | C57BL/6NCrl | +/+ | M | 483043 | 12/29/2016 | 212.71 | 678.52 | 123.58 | 32.13 | 6.15 | 7.46 | 9.97 | 286.88 | 163.99 | 100.14 | 972.23 | 466.93 | 39.30 | 323.15 | 431.91 | 323.67 | 605.44 | 17.72 | 326.91 | 565.59 | 78.04 | 37.19 | 10.29 | 1.16 | 11.23 | 0.74 | 79.81 | 0.00 | 31.85 | 26.48 | 28.60 | 633.68 | 10484.04 | 5696.48 | 6178.72 | 0.86 | 105.91 | 124.73 | 2.01 | 1.31 | 7.15 | 19.35 | 2.25 | 3.92 | 1023.99 | 567.01 | 1.05 | 0.00 | 593.10 | 291.41 | 856.96 | 292.99 |
| 34418 | NorCOMM2 | (null) | C57BL/6NCrl | +/+ | M | 34418 | 11/20/2013 | 242.17 | 594.13 | 82.94 | 32.78 | 8.85 | 10.98 | 7.53 | 419.47 | 301.45 | 106.63 | 970.14 | 722.88 | 75.35 | 770.41 | 930.74 | 702.46 | 1512.70 | 29.76 | 550.25 | 533.00 | 115.75 | 19.38 | 27.86 | 1.57 | 12.34 | 0.80 | 65.35 | 3.57 | 56.62 | 28.99 | 28.01 | 705.74 | 8277.13 | 4124.47 | 4436.37 | 2.16 | 27.25 | 132.85 | 27.85 | 2.06 | 4.74 | 37.67 | 0.00 | 4.78 | 799.92 | 612.34 | 2.51 | 0.63 | 600.82 | 3483.36 | 18300.86 | 7311.76 |
| 257539 | NorCOMM2 | (null) | C57BL/6NCrl | +/+ | M | 257539 | 5/20/2015 | 238.90 | 682.91 | 128.52 | 25.69 | 3.28 | 7.67 | 7.81 | 429.39 | 240.81 | 116.50 | 1035.90 | 648.58 | 52.37 | 409.80 | 581.17 | 455.52 | 845.00 | 22.89 | 474.60 | 743.68 | 148.28 | 22.94 | 21.32 | 0.80 | 11.35 | 1.17 | 58.87 | 0.00 | 29.75 | 44.07 | 33.15 | 904.64 | 9889.50 | 7077.64 | 8787.51 | 2.48 | 209.21 | 143.87 | 1.82 | 1.41 | 3.65 | 17.69 | 4.84 | 3.92 | 1004.44 | 729.97 | 0.98 | 0.93 | 845.95 | 400.31 | 1137.09 | 420.18 |
| 348925 | NorCOMM2 | (null) | C57BL/6NCrl | +/+ | M | 348925 | 11/3/2015 | 186.02 | 733.65 | 122.56 | 23.22 | 7.56 | 9.96 | 12.37 | 339.95 | 205.79 | 90.84 | 1092.31 | 693.94 | 70.01 | 559.31 | 745.35 | 508.93 | 904.38 | 20.49 | 630.99 | 810.75 | 109.26 | 21.32 | 20.94 | 1.32 | 10.36 | 1.22 | 81.67 | 2.47 | 35.55 | 44.05 | 43.54 | 639.14 | 8872.95 | 6996.92 | 7366.02 | 1.58 | 174.41 | 135.37 | 2.00 | 2.39 | 3.95 | 40.03 | 2.12 | 3.50 | 1139.11 | 675.82 | 1.14 | 0.99 | 569.49 | 34.95 | 606.20 | 313.69 |
| 91833 | NorCOMM2 | (null) | C57BL/6NCrl | +/+ | M | 91833 | 4/29/2014 | 230.49 | 812.32 | 142.61 | 36.43 | 8.24 | 11.58 | 13.75 | 460.95 | 248.23 | 116.59 | 1070.96 | 900.76 | 73.43 | 576.11 | 893.84 | 604.31 | 1374.17 | 31.51 | 511.39 | 539.70 | 98.72 | 29.56 | 18.36 | 1.71 | 13.24 | 1.39 | 73.93 | 2.29 | 42.57 | 32.07 | 32.00 | 884.19 | 17205.81 | 9495.42 | 10791.27 | 1.81 | 223.76 | 154.66 | 4.54 | 2.07 | 6.29 | 29.54 | 2.00 | 5.39 | 1088.60 | 791.77 | 0.69 | 1.53 | 638.52 | 8580.49 | 1851.97 | 790.77 |
| 365958 | NorCOMM2 | (null) | C57BL/6NCrl | +/+ | M | 365958 | 12/17/2015 | 262.93 | 805.67 | 142.53 | 34.20 | 5.93 | 9.63 | 17.39 | 544.39 | 350.70 | 122.83 | 1063.02 | 666.88 | 77.21 | 666.04 | 830.54 | 727.93 | 1458.86 | 25.75 | 425.03 | 536.60 | 134.24 | 36.17 | 19.47 | 0.96 | 12.41 | 0.95 | 80.09 | 1.05 | 50.72 | 32.33 | 29.04 | 867.93 | 10268.59 | 7663.49 | 7624.32 | 1.25 | 188.99 | 142.63 | 5.28 | 1.74 | 4.23 | 15.74 | 2.96 | 4.35 | 1256.79 | 584.14 | 1.25 | 1.45 | 661.84 | 2807.16 | 4210.13 | 1527.54 |
| 209136 | NorCOMM2 | (null) | C57BL/6NCrl | +/+ | M | 209136 | 1/13/2015 | 217.15 | 815.96 | 141.44 | 31.69 | 7.82 | 9.45 | 13.24 | 285.85 | 123.75 | 78.83 | 1185.01 | 557.90 | 52.71 | 491.53 | 683.85 | 473.82 | 869.26 | 21.55 | 373.89 | 777.32 | 122.20 | 44.38 | 17.93 | 1.16 | 10.68 | 1.70 | 68.47 | 2.22 | 37.91 | 32.48 | 33.94 | 919.50 | 9485.28 | 4987.10 | 8809.72 | 1.57 | 230.73 | 118.78 | 3.49 | 1.58 | 5.04 | 16.28 | 2.32 | 4.75 | 954.82 | 628.53 | 1.69 | 0.88 | 774.44 | 842.38 | 1788.15 | 799.08 |
| 229586 | NorCOMM2 | (null) | C57BL/6NCrl | +/+ | M | 229586 | 3/24/2015 | 272.39 | 804.51 | 103.81 | 36.59 | 11.16 | 9.90 | 14.97 | 300.80 | 248.34 | 97.70 | 980.48 | 756.18 | 64.52 | 459.61 | 749.61 | 448.92 | 898.70 | 29.09 | 449.92 | 558.02 | 116.79 | 35.99 | 12.37 | 0.93 | 13.32 | 1.26 | 68.97 | 3.41 | 33.55 | 29.70 | 29.82 | 784.06 | 8836.20 | 7542.20 | 6496.30 | 1.36 | 232.80 | 139.68 | 4.71 | 1.57 | 4.90 | 28.19 | 3.70 | 4.30 | 1098.80 | 728.39 | 0.82 | 1.03 | 800.41 | 992.65 | 4575.40 | 1817.00 |
| 187594 | NorCOMM2 | (null) | C57BL/6NCrl | +/+ | M | 187594 | 11/25/2014 | 229.28 | 762.52 | 103.71 | 40.28 | 3.74 | 9.53 | 16.66 | 269.27 | 157.36 | 68.09 | 1143.80 | 454.00 | 47.78 | 330.67 | 448.76 | 359.53 | 713.89 | 25.94 | 302.69 | 805.15 | 121.29 | 46.31 | 15.06 | 0.84 | 10.21 | 1.18 | 81.52 | 0.85 | 31.78 | 25.56 | 38.23 | 740.36 | 3878.10 | 2942.50 | 3107.00 | 0.74 | 36.77 | 88.01 | 4.34 | 1.63 | 4.86 | 24.04 | 3.39 | 4.68 | 1109.20 | 593.80 | 1.64 | 1.00 | 700.71 | 497.72 | 2646.80 | 921.26 |
| 281969 | NorCOMM2 | (null) | C57BL/6NCrl | +/+ | M | 281969 | 6/23/2015 | 226.76 | 963.41 | 125.27 | 37.40 | 3.91 | 9.99 | 14.39 | 533.34 | 266.32 | 107.31 | 1377.90 | 682.36 | 64.96 | 454.65 | 800.37 | 509.29 | 958.13 | 29.25 | 508.84 | 590.16 | 91.98 | 37.04 | 25.79 | 1.00 | 12.49 | 1.35 | 76.73 | 0.00 | 33.06 | 28.79 | 41.05 | 720.43 | 14222.00 | 7263.90 | 8293.50 | 2.02 | 194.11 | 113.20 | 1.58 | 1.75 | 4.34 | 26.56 | 5.36 | 5.21 | 1057.70 | 744.27 | 0.78 | 0.45 | 778.59 | 1223.10 | 1193.50 | 438.26 |
| 115756 | NorCOMM2 | (null) | C57BL/6NCrl | +/+ | M | 115756 | 6/5/2014 | 191.37 | 789.67 | 80.26 | 25.57 | 5.10 | 7.04 | 18.40 | 432.53 | 248.65 | 81.03 | 1224.00 | 615.20 | 65.88 | 413.42 | 661.05 | 493.37 | 818.02 | 21.93 | 440.45 | 710.18 | 77.47 | 44.36 | 14.42 | 1.03 | 9.59 | 1.21 | 54.42 | 3.13 | 27.39 | 23.54 | 41.72 | 690.24 | 6432.30 | 3646.90 | 3533.10 | 0.94 | 19.56 | 120.13 | 12.79 | 2.39 | 4.47 | 15.67 | 0.00 | 4.54 | 990.29 | 700.88 | 2.04 | 0.62 | 547.87 | 33.49 | 12099.00 | 3942.20 |
| 38433 | NorCOMM2 | (null) | C57BL/6NCrl | +/+ | M | 38433 | 12/11/2013 | 160.36 | 908.69 | 104.66 | 35.21 | 7.71 | 12.76 | 15.51 | 467.59 | 237.23 | 107.27 | 788.24 | 697.38 | 70.71 | 517.97 | 717.67 | 511.84 | 1335.80 | 27.66 | 344.43 | 568.36 | 93.36 | 38.49 | 16.98 | 0.95 | 15.21 | 1.09 | 57.74 | 3.98 | 30.71 | 35.51 | 37.30 | 784.66 | 8778.10 | 6371.80 | 5572.50 | 1.31 | 94.40 | 112.92 | 12.22 | 2.17 | 4.67 | 20.74 | 2.44 | 5.89 | 1190.70 | 688.83 | 2.82 | 1.52 | 572.95 | 5179.10 | 10191.00 | 3245.70 |
| 144609 | NorCOMM2 | (null) | C57BL/6NCrl | +/+ | M | 144609 | 8/7/2014 | 264.95 | 1103.50 | 77.70 | 54.74 | 6.33 | 8.08 | 13.67 | 401.93 | 247.62 | 80.97 | 1364.50 | 602.93 | 49.28 | 403.79 | 632.41 | 390.72 | 546.64 | 26.82 | 427.95 | 887.96 | 85.42 | 40.75 | 20.22 | 0.88 | 12.28 | 0.82 | 61.29 | 5.18 | 30.95 | 27.58 | 36.20 | 826.68 | 3495.70 | 3472.60 | 2679.20 | 0.87 | 34.64 | 119.73 | 16.97 | 1.39 | 7.84 | 18.01 | 0.00 | 4.64 | 1091.20 | 794.70 | 1.98 | 0.60 | 898.53 | 114.15 | 16039.00 | 5914.40 |
| 204873 | NorCOMM2 | A2m | A2m_tm1b_C10 | -/- | F | 204873 | 12/23/2014 | 197.59 | 925.72 | 124.26 | 39.39 | 3.11 | 8.12 | 11.46 | 347.60 | 157.58 | 69.30 | 1471.20 | 457.74 | 26.39 | 213.64 | 206.77 | 176.94 | 260.80 | 23.11 | 373.52 | 1563.60 | 84.00 | 31.29 | 14.93 | 1.24 | 12.36 | 0.84 | 85.74 | 1.20 | 20.36 | 30.44 | 42.19 | 744.87 | 5967.30 | 4337.40 | 4755.20 | 1.23 | 104.95 | 96.99 | 3.36 | 1.75 | 4.49 | 12.80 | 0.00 | 4.93 | 1357.40 | 639.77 | 1.45 | 5.37 | 329.82 | 22.22 | 2079.60 | 769.52 |
| 204868 | NorCOMM2 | A2m | A2m_tm1b_C10 | -/- | F | 204868 | 12/23/2014 | 236.56 | 800.99 | 178.50 | 41.90 | 4.40 | 7.60 | 11.40 | 298.47 | 133.15 | 63.15 | 1308.60 | 350.90 | 24.62 | 137.23 | 161.86 | 122.57 | 267.22 | 22.93 | 344.08 | 1515.50 | 115.71 | 28.11 | 15.62 | 1.03 | 14.14 | 1.07 | 56.05 | 0.00 | 21.52 | 37.11 | 39.69 | 863.81 | 6354.00 | 4886.50 | 4283.10 | 1.94 | 84.89 | 115.82 | 3.36 | 1.52 | 6.12 | 18.94 | 2.98 | 4.97 | 1124.60 | 536.80 | 1.18 | 7.47 | 416.47 | 21.83 | 2081.50 | 897.56 |
| 204872 | NorCOMM2 | A2m | A2m_tm1b_C10 | -/- | F | 204872 | 12/23/2014 | 220.28 | 824.59 | 134.81 | 36.31 | 5.43 | 8.39 | 12.63 | 238.52 | 201.23 | 78.45 | 1404.90 | 407.97 | 23.46 | 209.59 | 257.14 | 195.48 | 237.57 | 23.97 | 406.57 | 1476.00 | 104.11 | 43.81 | 14.75 | 0.82 | 11.75 | 1.15 | 74.78 | 3.60 | 20.83 | 39.64 | 54.21 | 776.09 | 10112.00 | 5403.00 | 5363.00 | 2.21 | 122.10 | 80.68 | 2.62 | 1.85 | 0.00 | 13.76 | 0.00 | 4.89 | 1227.60 | 601.55 | 1.53 | 6.64 | 389.76 | 56.32 | 2244.60 | 767.93 |
| 197966 | NorCOMM2 | A2m | A2m_tm1b_C10 | -/- | M | 197966 | 12/18/2014 | 323.05 | 1129.60 | 115.45 | 30.86 | 3.54 | 6.26 | 16.75 | 336.98 | 149.41 | 99.23 | 935.59 | 547.41 | 64.30 | 634.75 | 609.29 | 553.22 | 1000.70 | 24.46 | 468.51 | 739.74 | 116.78 | 42.44 | 13.58 | 1.23 | 13.78 | 0.99 | 77.05 | 0.00 | 40.47 | 26.37 | 36.34 | 846.48 | 7462.10 | 5094.00 | 5067.20 | 1.24 | 66.84 | 135.02 | 6.74 | 1.60 | 4.54 | 21.83 | 3.16 | 4.13 | 1089.80 | 719.86 | 1.27 | 0.51 | 974.75 | 1289.00 | 3721.40 | 1576.90 |
| 197962 | NorCOMM2 | A2m | A2m_tm1b_C10 | -/- | M | 197962 | 12/18/2014 | 280.91 | 977.56 | 131.44 | 33.66 | 5.17 | 7.31 | 15.72 | 435.73 | 276.15 | 109.01 | 1163.50 | 703.04 | 80.46 | 692.71 | 829.72 | 690.53 | 1326.30 | 27.41 | 465.04 | 661.14 | 130.95 | 41.51 | 19.52 | 0.94 | 11.89 | 1.33 | 60.85 | 2.85 | 48.25 | 29.96 | 40.73 | 915.69 | 5002.70 | 4921.60 | 4770.70 | 1.30 | 33.63 | 124.18 | 5.88 | 1.52 | 3.87 | 24.31 | 0.00 | 4.75 | 1343.60 | 764.52 | 1.77 | 0.86 | 893.16 | 2722.60 | 5162.60 | 1705.40 |
| 197964 | NorCOMM2 | A2m | A2m_tm1b_C10 | -/- | M | 197964 | 12/18/2014 | 212.49 | 889.93 | 146.04 | 39.27 | 3.72 | 9.48 | 19.59 | 479.59 | 173.17 | 92.01 | 1187.00 | 691.34 | 61.24 | 470.09 | 502.45 | 528.64 | 971.31 | 34.57 | 512.03 | 715.33 | 94.03 | 46.60 | 20.97 | 1.14 | 11.85 | 1.24 | 61.32 | 1.53 | 33.34 | 38.33 | 49.27 | 760.17 | 7682.70 | 6894.60 | 7337.90 | 1.06 | 159.65 | 130.28 | 2.81 | 1.77 | 4.45 | 14.17 | 2.69 | 4.92 | 1192.30 | 701.66 | 1.48 | 0.75 | 931.99 | 320.81 | 1609.50 | 579.24 |
| 181060 | NorCOMM2 | Ahcy | Ahcy_tm1b_E07 | +/- | F | 181060 | 11/6/2014 | 256.15 | 951.23 | 148.94 | 54.08 | 3.21 | 7.01 | 22.08 | 410.06 | 375.72 | 97.79 | 1842.30 | 529.54 | 29.71 | 159.78 | 179.34 | 140.13 | 275.64 | 22.06 | 439.94 | 1582.60 | 106.29 | 18.58 | 11.95 | 0.99 | 11.45 | 0.95 | 78.99 | 1.49 | 24.37 | 44.64 | 38.24 | 803.22 | 7394.90 | 5694.20 | 6156.40 | 1.82 | 102.40 | 117.56 | 3.49 | 1.89 | 3.76 | 14.97 | 3.17 | 5.64 | 1342.70 | 619.55 | 0.60 | 5.73 | 404.07 | 21.37 | 1570.10 | 573.49 |
| 179053 | NorCOMM2 | Ahcy | Ahcy_tm1b_E07 | +/- | F | 179053 | 10/23/2014 | 220.63 | 759.40 | 130.60 | 29.29 | 3.28 | 11.91 | 18.23 | 510.10 | 331.90 | 106.04 | 1733.90 | 492.42 | 35.81 | 136.18 | 190.49 | 178.96 | 256.10 | 22.48 | 412.77 | 1632.70 | 122.18 | 40.18 | 13.38 | 0.76 | 14.17 | 1.09 | 66.80 | 1.57 | 17.46 | 38.70 | 41.98 | 764.32 | 6215.30 | 4971.70 | 4995.50 | 1.50 | 91.49 | 87.05 | 1.89 | 1.70 | 4.01 | 11.32 | 0.00 | 5.57 | 1130.40 | 563.10 | 0.83 | 9.07 | 459.42 | 45.48 | 1317.90 | 430.51 |
| 181059 | NorCOMM2 | Ahcy | Ahcy_tm1b_E07 | +/- | F | 181059 | 11/6/2014 | 229.99 | 905.22 | 158.51 | 36.31 | 2.79 | 9.14 | 18.33 | 346.90 | 167.19 | 85.08 | 1728.70 | 481.64 | 31.32 | 139.08 | 184.06 | 146.09 | 280.70 | 22.28 | 414.66 | 2158.10 | 96.65 | 25.78 | 17.73 | 1.24 | 14.11 | 1.20 | 98.20 | 1.73 | 16.98 | 48.18 | 38.15 | 817.23 | 7551.90 | 5357.90 | 6065.00 | 1.66 | 274.36 | 136.52 | 3.48 | 1.80 | 4.97 | 19.12 | 2.58 | 6.54 | 1110.30 | 564.21 | 1.50 | 7.52 | 483.93 | 28.64 | 1949.00 | 833.37 |
| 178951 | NorCOMM2 | Ahcy | Ahcy_tm1b_E07 | +/- | M | 178951 | 10/23/2014 | 233.53 | 850.35 | 127.07 | 43.28 | 5.04 | 10.55 | 21.93 | 595.30 | 432.97 | 105.02 | 1158.70 | 801.88 | 77.76 | 736.47 | 881.07 | 854.18 | 1277.30 | 27.65 | 417.38 | 571.34 | 105.65 | 47.18 | 18.96 | 1.13 | 12.81 | 1.11 | 71.26 | 2.78 | 55.84 | 34.08 | 33.23 | 888.10 | 8188.70 | 6866.00 | 6715.30 | 1.43 | 109.84 | 160.47 | 6.55 | 1.77 | 5.36 | 18.52 | 4.53 | 4.35 | 879.49 | 574.99 | 1.66 | 0.64 | 808.75 | 3008.60 | 4754.20 | 1829.70 |
| 178948 | NorCOMM2 | Ahcy | Ahcy_tm1b_E07 | +/- | M | 178948 | 10/23/2014 | 209.59 | 929.14 | 123.51 | 41.94 | 3.75 | 7.76 | 21.95 | 594.19 | 402.37 | 177.40 | 1435.70 | 633.81 | 68.77 | 722.22 | 734.60 | 716.61 | 1089.20 | 21.27 | 403.84 | 751.39 | 100.33 | 37.25 | 17.81 | 1.08 | 11.45 | 1.50 | 73.50 | 0.00 | 44.66 | 29.87 | 33.97 | 864.60 | 7491.30 | 5989.10 | 8952.40 | 1.35 | 62.27 | 143.66 | 3.11 | 2.25 | 4.51 | 16.44 | 2.42 | 4.72 | 1151.20 | 627.39 | 0.85 | 1.02 | 892.61 | 1410.00 | 2307.40 | 886.08 |
| 178950 | NorCOMM2 | Ahcy | Ahcy_tm1b_E07 | +/- | M | 178950 | 10/23/2014 | 260.31 | 919.01 | 106.23 | 35.91 | 3.60 | 8.17 | 18.98 | 279.43 | 250.93 | 98.90 | 1343.10 | 492.49 | 59.42 | 502.80 | 658.93 | 425.19 | 823.96 | 21.67 | 474.06 | 749.70 | 113.50 | 38.47 | 16.06 | 0.90 | 11.95 | 0.93 | 64.70 | 0.00 | 35.81 | 28.59 | 41.31 | 903.31 | 9721.90 | 6642.90 | 6129.50 | 1.13 | 135.97 | 141.91 | 2.51 | 1.26 | 5.12 | 18.83 | 3.77 | 4.17 | 1357.80 | 636.22 | 1.49 | 0.60 | 846.67 | 502.54 | 1493.50 | 501.71 |
| 143963 | NorCOMM2 | Atp5b | Atp5b_tm1b_B07 | +/- | F | 143963 | 8/7/2014 | 231.81 | 611.40 | 204.57 | 35.20 | 37.18 | 7.57 | 9.55 | 350.18 | 203.89 | 92.34 | 1880.34 | 514.07 | 25.42 | 166.36 | 197.32 | 184.63 | 251.28 | 27.25 | 476.26 | 1387.25 | 98.95 | 32.84 | 12.22 | 0.92 | 11.91 | 1.18 | 66.26 | 6.46 | 23.21 | 41.57 | 49.74 | 802.39 | 9280.51 | 5836.44 | 6613.30 | 2.07 | 165.04 | 147.00 | 3.35 | 2.29 | 4.29 | 24.28 | 0.00 | 5.74 | 1249.82 | 582.14 | 0.00 | 6.33 | 375.94 | 146.25 | 1694.93 | 972.66 |
| 143961 | NorCOMM2 | Atp5b | Atp5b_tm1b_B07 | +/- | F | 143961 | 8/7/2014 | 220.76 | 737.07 | 164.15 | 41.70 | 6.32 | 7.13 | 8.78 | 367.82 | 229.37 | 90.20 | 1754.09 | 560.30 | 28.24 | 192.68 | 196.84 | 191.87 | 241.42 | 32.15 | 563.69 | 1291.64 | 85.08 | 33.19 | 10.94 | 1.03 | 11.23 | 1.12 | 97.61 | 0.00 | 21.83 | 35.93 | 46.88 | 764.52 | 7355.04 | 5146.58 | 4786.57 | 1.71 | 43.04 | 144.59 | 5.78 | 1.74 | 0.00 | 15.15 | 0.00 | 5.70 | 1166.37 | 591.75 | 1.33 | 7.34 | 312.87 | 21.28 | 5982.28 | 2321.07 |
| 143960 | NorCOMM2 | Atp5b | Atp5b_tm1b_B07 | +/- | F | 143960 | 8/7/2014 | 247.20 | 649.21 | 160.29 | 41.25 | 20.41 | 7.67 | 8.07 | 525.71 | 342.09 | 101.79 | 1993.05 | 627.03 | 27.88 | 184.55 | 262.90 | 191.34 | 260.83 | 32.19 | 574.47 | 1450.25 | 105.38 | 31.77 | 11.14 | 0.97 | 10.31 | 1.12 | 100.73 | 0.00 | 26.37 | 48.24 | 55.24 | 734.34 | 10286.36 | 5645.60 | 7450.59 | 2.55 | 156.17 | 130.35 | 3.50 | 2.23 | 0.00 | 19.07 | 0.00 | 6.04 | 1120.12 | 705.30 | 0.00 | 6.60 | 379.57 | 41.06 | 1680.76 | 758.50 |
| 118347 | NorCOMM2 | Atp5b | Atp5b_tm1b_B07 | +/- | M | 118347 | 6/11/2014 | 255.09 | 759.47 | 109.03 | 23.80 | 12.01 | 9.24 | 17.60 | 530.22 | 269.32 | 148.01 | 973.91 | 737.46 | 76.65 | 768.07 | 939.98 | 764.83 | 1247.76 | 33.11 | 678.46 | 406.47 | 126.12 | 34.37 | 9.66 | 0.79 | 13.78 | 1.25 | 67.66 | 5.65 | 44.50 | 27.01 | 36.50 | 822.35 | 8440.65 | 9007.84 | 8129.40 | 1.75 | 88.45 | 153.03 | 6.55 | 2.34 | 4.55 | 39.61 | 1.62 | 3.37 | 1050.31 | 649.37 | 2.92 | 0.00 | 771.31 | 6281.16 | 5777.29 | 2256.05 |
| 118346 | NorCOMM2 | Atp5b | Atp5b_tm1b_B07 | +/- | M | 118346 | 6/11/2014 | 199.28 | 601.64 | 118.90 | 33.18 | 4.16 | 11.13 | 13.63 | 490.24 | 296.22 | 132.45 | 1126.06 | 825.95 | 49.19 | 504.05 | 680.60 | 536.98 | 813.74 | 33.54 | 496.68 | 594.29 | 97.80 | 28.31 | 11.43 | 0.95 | 9.91 | 1.16 | 85.50 | 0.00 | 35.64 | 30.78 | 32.18 | 837.78 | 7916.17 | 6314.87 | 5474.91 | 1.24 | 36.25 | 149.77 | 3.04 | 1.75 | 5.14 | 19.55 | 0.00 | 3.31 | 1064.35 | 623.55 | 1.38 | 0.86 | 584.29 | 286.57 | 1539.67 | 584.50 |
| 143858 | NorCOMM2 | Atp5b | Atp5b_tm1b_B07 | +/- | M | 143858 | 8/7/2014 | 159.09 | 524.36 | 53.86 | 14.64 | 7.08 | 13.51 | 11.47 | 720.67 | 349.83 | 117.96 | 1268.01 | 726.91 | 70.50 | 365.73 | 401.55 | 319.16 | 607.29 | 49.72 | 775.12 | 592.58 | 85.03 | 37.91 | 8.89 | 0.51 | 14.22 | 1.38 | 67.38 | 1.67 | 18.43 | 56.73 | 87.82 | 1098.62 | 12814.57 | 9608.97 | 8783.22 | 6.99 | 144.15 | 97.35 | 4.89 | 3.85 | 7.86 | 26.64 | 0.00 | 7.52 | 797.33 | 415.57 | 1.45 | 5.53 | 245.05 | 8367.41 | 3535.87 | 1355.25 |
| 281686 | NorCOMM2 | Atp6v0d1 | Atp6v0d1_tm1b_E01 | +/- | F | 281686 | 6/24/2015 | 192.22 | 530.07 | 139.71 | 22.70 | 3.35 | 14.96 | 8.38 | 451.88 | 305.15 | 102.02 | 1366.24 | 595.59 | 21.20 | 130.01 | 147.86 | 105.89 | 218.30 | 17.06 | 466.03 | 1614.33 | 98.93 | 37.91 | 19.25 | 1.22 | 10.75 | 0.73 | 88.93 | 0.00 | 8.54 | 46.99 | 48.88 | 732.86 | 8540.55 | 5780.01 | 5648.58 | 2.04 | 277.46 | 123.51 | 1.25 | 1.95 | 4.35 | 16.83 | 2.38 | 4.18 | 1324.27 | 560.60 | 0.75 | 7.20 | 309.84 | 69.03 | 645.70 | 251.88 |
| 281679 | NorCOMM2 | Atp6v0d1 | Atp6v0d1_tm1b_E01 | +/- | F | 281679 | 6/24/2015 | 217.29 | 542.16 | 160.59 | 31.71 | 1.60 | 8.20 | 14.60 | 390.27 | 223.03 | 91.84 | 1724.65 | 535.21 | 20.83 | 142.90 | 167.80 | 149.68 | 232.97 | 23.79 | 409.84 | 1136.87 | 83.77 | 27.65 | 12.08 | 0.80 | 10.06 | 0.71 | 69.28 | 0.00 | 25.86 | 34.21 | 32.83 | 622.57 | 6549.92 | 5242.49 | 5592.02 | 1.67 | 168.77 | 93.87 | 1.60 | 1.81 | 1.96 | 13.02 | 1.55 | 5.01 | 1094.71 | 484.87 | 1.09 | 5.90 | 328.09 | 29.68 | 1072.06 | 393.47 |
| 281678 | NorCOMM2 | Atp6v0d1 | Atp6v0d1_tm1b_E01 | +/- | F | 281678 | 6/24/2015 | 172.42 | 524.97 | 131.92 | 39.81 | 3.57 | 8.02 | 6.56 | 253.44 | 132.67 | 69.97 | 1379.19 | 688.32 | 31.55 | 135.20 | 176.86 | 149.59 | 234.39 | 19.11 | 432.83 | 1801.02 | 105.29 | 24.50 | 18.83 | 0.73 | 10.46 | 0.77 | 79.80 | 2.68 | 7.39 | 40.34 | 58.62 | 631.43 | 8675.78 | 5698.78 | 6219.23 | 1.86 | 173.79 | 155.22 | 4.51 | 1.43 | 3.42 | 13.30 | 2.63 | 4.88 | 1040.99 | 578.93 | 1.26 | 3.56 | 431.20 | 58.92 | 3594.83 | 1509.99 |
| 281575 | NorCOMM2 | Atp6v0d1 | Atp6v0d1_tm1b_E01 | +/- | M | 281575 | 6/23/2015 | 194.88 | 756.72 | 148.23 | 30.81 | 4.42 | 9.50 | 14.06 | 443.74 | 265.14 | 105.37 | 1121.51 | 761.56 | 65.19 | 542.72 | 597.32 | 475.11 | 712.83 | 31.76 | 401.83 | 722.01 | 124.11 | 39.12 | 24.14 | 1.60 | 10.75 | 1.36 | 92.08 | 0.00 | 27.37 | 32.75 | 43.26 | 899.63 | 8566.62 | 6705.81 | 6792.95 | 1.40 | 166.54 | 153.68 | 5.66 | 2.04 | 5.71 | 22.55 | 3.18 | 6.94 | 1213.94 | 702.11 | 1.40 | 0.59 | 809.93 | 648.09 | 3963.26 | 1572.70 |
| 281582 | NorCOMM2 | Atp6v0d1 | Atp6v0d1_tm1b_E01 | +/- | M | 281582 | 6/23/2015 | 204.57 | 764.96 | 116.01 | 29.70 | 3.92 | 10.98 | 10.76 | 535.50 | 338.68 | 127.94 | 1228.76 | 715.89 | 69.62 | 517.47 | 681.78 | 596.12 | 1097.65 | 27.29 | 442.77 | 686.11 | 116.21 | 44.33 | 17.84 | 0.75 | 9.67 | 1.03 | 86.57 | 0.00 | 44.74 | 30.52 | 31.55 | 816.22 | 10137.15 | 6463.34 | 8804.91 | 1.79 | 335.12 | 127.65 | 3.08 | 2.00 | 5.30 | 16.50 | 3.37 | 5.05 | 1051.03 | 661.19 | 0.67 | 0.91 | 637.85 | 1412.06 | 2709.17 | 1041.89 |
| 281584 | NorCOMM2 | Atp6v0d1 | Atp6v0d1_tm1b_E01 | +/- | M | 281584 | 6/23/2015 | 253.91 | 871.37 | 160.51 | 31.19 | 3.58 | 11.98 | 12.25 | 502.81 | 274.80 | 117.63 | 1045.26 | 731.77 | 71.30 | 558.60 | 774.32 | 562.30 | 1277.39 | 23.94 | 526.94 | 607.95 | 107.44 | 32.70 | 26.46 | 1.23 | 12.99 | 1.76 | 65.56 | 2.06 | 37.09 | 35.33 | 35.63 | 841.57 | 12114.10 | 7213.46 | 7612.29 | 2.00 | 273.04 | 121.70 | 4.22 | 1.93 | 7.09 | 28.67 | 3.29 | 5.37 | 1215.08 | 643.59 | 1.11 | 0.84 | 782.22 | 1117.06 | 2912.38 | 1043.18 |
| 222890 | NorCOMM2 | C8a | C8a_tm1b_F01 | -/- | F | 222890 | 2/18/2015 | 232.81 | 551.26 | 68.00 | 15.77 | 4.32 | 17.00 | 8.75 | 339.79 | 250.27 | 82.24 | 1398.65 | 528.19 | 27.34 | 0.00 | 0.00 | 4.41 | 269.50 | 22.34 | 358.04 | 1278.65 | 139.27 | 25.93 | 14.88 | 1.12 | 12.26 | 0.74 | 87.22 | 0.00 | 9.67 | 34.25 | 51.99 | 633.16 | 6838.89 | 4279.26 | 5197.06 | 1.33 | 106.73 | 128.62 | 2.50 | 1.91 | 5.03 | 22.92 | 2.63 | 5.82 | 1265.48 | 559.70 | 0.72 | 5.69 | 437.12 | 689.18 | 1739.32 | 642.02 |
| 222888 | NorCOMM2 | C8a | C8a_tm1b_F01 | -/- | F | 222888 | 2/18/2015 | 193.73 | 704.97 | 164.11 | 38.03 | 3.62 | 11.04 | 13.56 | 546.44 | 326.69 | 103.33 | 1517.43 | 546.46 | 27.95 | 0.00 | 0.00 | 4.46 | 202.64 | 20.04 | 441.24 | 1402.16 | 97.01 | 32.36 | 14.86 | 1.55 | 12.31 | 1.21 | 95.62 | 1.12 | 24.73 | 42.70 | 31.44 | 775.40 | 8618.15 | 4663.60 | 5494.43 | 2.31 | 243.30 | 114.40 | 2.19 | 1.98 | 4.07 | 23.99 | 2.79 | 5.83 | 1131.76 | 574.02 | 1.54 | 6.21 | 374.17 | 228.82 | 912.40 | 299.99 |
| 222895 | NorCOMM2 | C8a | C8a_tm1b_F01 | -/- | F | 222895 | 2/18/2015 | 201.91 | 632.13 | 190.64 | 27.54 | 8.42 | 8.37 | 13.10 | 376.40 | 206.08 | 97.72 | 1670.41 | 468.62 | 25.34 | 0.00 | 0.00 | 4.81 | 268.27 | 23.97 | 557.99 | 1412.82 | 103.21 | 29.64 | 18.11 | 0.91 | 11.96 | 1.45 | 73.36 | 1.62 | 17.93 | 52.22 | 43.74 | 721.11 | 8880.62 | 5404.55 | 6926.54 | 1.55 | 208.20 | 109.44 | 2.37 | 1.68 | 4.13 | 21.11 | 2.86 | 6.09 | 1287.94 | 551.72 | 0.84 | 6.76 | 526.84 | 143.78 | 1125.65 | 399.50 |
| 233514 | NorCOMM2 | C8a | C8a_tm1b_F01 | -/- | M | 233514 | 4/8/2015 | 242.48 | 708.59 | 116.99 | 24.93 | 2.28 | 9.95 | 11.23 | 384.15 | 251.04 | 99.67 | 918.19 | 739.55 | 63.46 | 0.00 | 91.48 | 5.19 | 1277.93 | 27.68 | 570.40 | 573.85 | 112.39 | 27.45 | 15.51 | 0.90 | 11.62 | 1.34 | 64.37 | 0.00 | 40.60 | 26.48 | 34.66 | 790.84 | 10585.11 | 5775.07 | 5899.33 | 1.66 | 92.67 | 125.85 | 2.99 | 1.63 | 6.41 | 21.22 | 2.64 | 4.80 | 958.49 | 670.80 | 0.96 | 1.29 | 790.72 | 1029.12 | 1913.56 | 801.40 |
| 233523 | NorCOMM2 | C8a | C8a_tm1b_F01 | -/- | M | 233523 | 4/8/2015 | 239.73 | 858.84 | 124.82 | 29.90 | 4.21 | 6.59 | 8.93 | 276.24 | 189.70 | 104.40 | 952.56 | 804.41 | 69.71 | 0.00 | 122.59 | 6.62 | 1310.41 | 31.08 | 480.29 | 503.55 | 82.75 | 36.43 | 21.84 | 1.05 | 12.95 | 1.16 | 76.15 | 0.67 | 41.30 | 29.40 | 41.05 | 855.00 | 8204.67 | 5424.93 | 8125.61 | 1.82 | 144.56 | 155.20 | 2.80 | 1.87 | 3.17 | 24.98 | 2.92 | 4.86 | 1089.74 | 633.20 | 1.61 | 0.64 | 497.45 | 2426.68 | 1251.66 | 491.61 |
| 233515 | NorCOMM2 | C8a | C8a_tm1b_F01 | -/- | M | 233515 | 4/8/2015 | 253.75 | 922.80 | 123.89 | 30.44 | 2.51 | 10.12 | 9.18 | 381.06 | 239.89 | 103.96 | 926.43 | 830.16 | 77.44 | 0.00 | 102.79 | 6.17 | 1118.37 | 23.30 | 427.09 | 548.43 | 103.49 | 32.74 | 18.08 | 1.21 | 11.55 | 1.57 | 65.62 | 0.00 | 68.94 | 31.59 | 32.15 | 764.06 | 10262.49 | 6962.91 | 10677.49 | 1.83 | 334.65 | 124.47 | 1.41 | 1.89 | 4.23 | 20.11 | 0.00 | 4.43 | 1115.31 | 644.72 | 0.79 | 1.20 | 648.79 | 2655.85 | 586.72 | 271.29 |
| 206829 | NorCOMM2 | Cdk4 | Cdk4_tm1b_F08 | +/- | F | 206829 | 1/8/2015 | 216.09 | 617.40 | 154.54 | 43.56 | 5.92 | 9.37 | 7.91 | 293.21 | 188.25 | 58.52 | 1583.67 | 567.25 | 28.88 | 225.49 | 204.53 | 172.17 | 247.99 | 26.94 | 515.85 | 1450.34 | 94.21 | 24.44 | 23.36 | 1.17 | 12.30 | 1.25 | 86.36 | 0.00 | 23.17 | 43.14 | 46.51 | 799.90 | 9254.56 | 6099.27 | 5397.38 | 2.46 | 180.02 | 123.01 | 2.47 | 2.13 | 2.69 | 29.57 | 2.53 | 5.46 | 1192.93 | 570.99 | 1.41 | 4.53 | 392.14 | 23.65 | 1272.02 | 407.61 |
| 206828 | NorCOMM2 | Cdk4 | Cdk4_tm1b_F08 | +/- | F | 206828 | 1/6/2015 | 197.59 | 567.71 | 156.09 | 33.21 | 5.04 | 10.63 | 9.35 | 388.07 | 249.54 | 82.82 | 1345.26 | 477.20 | 21.91 | 175.68 | 187.54 | 162.21 | 254.86 | 22.13 | 496.92 | 1533.66 | 108.82 | 21.68 | 16.03 | 1.49 | 12.15 | 0.86 | 76.04 | 0.00 | 24.09 | 40.52 | 43.65 | 612.40 | 6359.69 | 4479.15 | 4983.40 | 1.94 | 118.14 | 122.86 | 2.83 | 2.02 | 2.98 | 21.16 | 2.33 | 5.45 | 1314.85 | 603.01 | 0.00 | 5.36 | 367.61 | 28.10 | 2399.90 | 794.60 |
| 206822 | NorCOMM2 | Cdk4 | Cdk4_tm1b_F08 | +/- | F | 206822 | 1/6/2015 | 228.05 | 648.50 | 175.04 | 46.49 | 4.65 | 9.42 | 10.23 | 354.63 | 306.48 | 95.19 | 1522.15 | 440.50 | 25.26 | 139.90 | 147.98 | 165.35 | 273.20 | 24.54 | 435.67 | 1438.19 | 97.76 | 27.05 | 13.54 | 0.96 | 11.91 | 1.25 | 74.67 | 1.93 | 23.20 | 27.89 | 38.31 | 701.63 | 6044.96 | 4298.83 | 4215.92 | 1.42 | 50.90 | 116.24 | 2.60 | 2.34 | 3.19 | 15.90 | 0.00 | 5.10 | 1155.05 | 447.89 | 0.59 | 4.13 | 359.42 | 19.10 | 1624.35 | 671.61 |
| 190208 | NorCOMM2 | Cdk4 | Cdk4_tm1b_F08 | +/- | M | 190208 | 11/25/2014 | 246.04 | 630.94 | 100.44 | 36.28 | 6.02 | 8.88 | 23.52 | 530.00 | 301.59 | 107.93 | 1196.54 | 820.37 | 52.69 | 796.56 | 713.47 | 680.34 | 1132.72 | 29.56 | 392.84 | 485.40 | 122.39 | 35.41 | 20.79 | 0.90 | 11.06 | 0.96 | 58.75 | 4.96 | 44.62 | 22.68 | 33.27 | 832.93 | 7357.00 | 4863.70 | 6226.56 | 1.16 | 49.26 | 127.87 | 4.60 | 1.73 | 3.72 | 26.02 | 2.59 | 4.53 | 938.01 | 648.54 | 2.50 | 0.64 | 772.96 | 1386.35 | 3951.71 | 1639.01 |
| 190207 | NorCOMM2 | Cdk4 | Cdk4_tm1b_F08 | +/- | M | 190207 | 11/25/2014 | 291.86 | 930.72 | 127.58 | 38.04 | 3.78 | 11.14 | 14.08 | 575.49 | 303.24 | 147.78 | 1477.74 | 818.31 | 76.44 | 714.91 | 804.37 | 751.19 | 1391.43 | 27.38 | 644.78 | 556.13 | 132.53 | 30.29 | 22.92 | 1.06 | 13.72 | 1.34 | 78.40 | 1.83 | 56.13 | 24.16 | 26.52 | 835.20 | 13505.33 | 8921.07 | 11017.08 | 2.09 | 119.13 | 142.47 | 4.22 | 1.93 | 3.51 | 18.92 | 0.00 | 6.16 | 1054.55 | 594.93 | 1.75 | 1.12 | 837.42 | 6290.31 | 2162.76 | 767.01 |
| 190206 | NorCOMM2 | Cdk4 | Cdk4_tm1b_F08 | +/- | M | 190206 | 11/25/2014 | 288.75 | 674.99 | 126.58 | 40.79 | 2.93 | 11.03 | 14.19 | 532.14 | 339.53 | 109.35 | 1263.62 | 984.97 | 82.16 | 824.17 | 898.08 | 699.03 | 1346.12 | 37.51 | 396.41 | 532.07 | 175.36 | 41.82 | 21.71 | 1.56 | 13.37 | 1.11 | 82.27 | 3.03 | 62.19 | 27.85 | 27.71 | 917.78 | 14199.36 | 9733.25 | 11426.25 | 3.00 | 84.54 | 141.44 | 1.61 | 2.02 | 5.36 | 25.51 | 4.77 | 5.89 | 956.19 | 654.88 | 1.18 | 0.77 | 1119.99 | 10628.54 | 1529.12 | 621.95 |
| 122069 | NorCOMM2 | Dhfr | Dhfr_tm1b_H07 | +/- | F | 122069 | 6/19/2014 | 203.28 | 689.99 | 119.75 | 35.72 | 5.33 | 8.70 | 21.46 | 272.61 | 217.86 | 86.09 | 1965.00 | 406.33 | 25.11 | 165.79 | 214.04 | 160.20 | 277.21 | 23.66 | 417.68 | 1489.90 | 80.32 | 44.25 | 18.65 | 1.09 | 14.72 | 1.18 | 62.77 | 2.47 | 18.30 | 34.71 | 46.41 | 800.85 | 5599.30 | 4809.50 | 5013.20 | 1.69 | 54.38 | 144.13 | 7.51 | 2.13 | 3.81 | 12.95 | 4.17 | 5.86 | 1347.10 | 605.33 | 2.30 | 6.58 | 440.46 | 96.53 | 7579.80 | 2762.10 |
| 122067 | NorCOMM2 | Dhfr | Dhfr_tm1b_H07 | +/- | F | 122067 | 6/19/2014 | 213.79 | 690.38 | 148.55 | 42.11 | 3.16 | 9.91 | 19.86 | 545.32 | 224.67 | 82.19 | 1911.40 | 531.37 | 26.68 | 175.48 | 259.31 | 125.61 | 238.19 | 31.36 | 498.77 | 1491.20 | 104.51 | 38.73 | 16.93 | 1.02 | 12.26 | 1.34 | 61.67 | 1.41 | 10.80 | 34.28 | 44.15 | 761.95 | 5958.40 | 4348.20 | 4584.50 | 1.25 | 74.53 | 134.50 | 5.08 | 2.11 | 4.04 | 21.62 | 4.37 | 4.70 | 1460.60 | 584.99 | 1.40 | 10.14 | 357.06 | 25.77 | 4078.90 | 1740.70 |
| 122066 | NorCOMM2 | Dhfr | Dhfr_tm1b_H07 | +/- | F | 122066 | 6/19/2014 | 185.10 | 662.71 | 118.40 | 48.82 | 4.74 | 7.12 | 18.62 | 408.03 | 217.10 | 73.96 | 1710.70 | 450.74 | 33.17 | 129.88 | 212.30 | 138.80 | 138.78 | 25.99 | 393.12 | 1338.50 | 77.55 | 38.17 | 12.93 | 0.88 | 10.28 | 1.11 | 80.98 | 2.20 | 22.14 | 39.37 | 56.08 | 800.21 | 7810.30 | 5297.30 | 4868.00 | 1.97 | 96.51 | 141.52 | 2.74 | 2.35 | 0.00 | 11.08 | 0.00 | 5.95 | 1281.70 | 577.86 | 0.70 | 7.86 | 455.36 | 70.22 | 2279.50 | 762.06 |
| 121964 | NorCOMM2 | Dhfr | Dhfr_tm1b_H07 | +/- | M | 121964 | 6/19/2014 | 221.48 | 1030.80 | 117.22 | 39.40 | 5.72 | 13.74 | 17.57 | 302.75 | 147.76 | 90.69 | 1557.40 | 552.36 | 49.42 | 357.63 | 444.86 | 381.48 | 726.25 | 23.05 | 453.86 | 917.68 | 109.56 | 36.85 | 21.40 | 0.89 | 11.64 | 0.90 | 78.72 | 2.45 | 25.82 | 34.35 | 43.90 | 911.89 | 6899.40 | 6441.20 | 5708.00 | 0.86 | 207.26 | 123.20 | 4.18 | 1.64 | 4.54 | 19.28 | 2.81 | 4.38 | 1146.60 | 718.73 | 1.43 | 0.76 | 710.18 | 122.84 | 3922.90 | 1258.00 |
| 121962 | NorCOMM2 | Dhfr | Dhfr_tm1b_H07 | +/- | M | 121962 | 6/19/2014 | 245.82 | 839.08 | 121.60 | 40.12 | 3.93 | 9.12 | 20.22 | 308.55 | 309.71 | 114.95 | 1100.50 | 692.43 | 57.39 | 422.48 | 699.19 | 430.27 | 610.64 | 24.93 | 436.23 | 699.05 | 89.89 | 19.87 | 16.97 | 1.27 | 13.96 | 0.98 | 77.55 | 0.87 | 33.74 | 35.69 | 38.12 | 795.26 | 7792.90 | 5919.70 | 6510.50 | 1.08 | 153.74 | 110.36 | 2.69 | 1.68 | 6.35 | 13.64 | 3.42 | 4.42 | 916.56 | 665.63 | 0.67 | 0.45 | 938.40 | 107.88 | 1978.50 | 687.11 |
| 129282 | NorCOMM2 | Dhfr | Dhfr_tm1b_H07 | +/- | M | 129282 | 7/9/2014 | 236.01 | 1049.90 | 129.52 | 40.75 | 4.32 | 8.18 | 12.33 | 226.43 | 237.17 | 103.87 | 1294.00 | 690.39 | 65.06 | 543.63 | 680.19 | 516.37 | 978.22 | 27.68 | 408.83 | 682.23 | 107.01 | 37.07 | 16.69 | 1.51 | 11.48 | 1.42 | 62.40 | 2.26 | 34.10 | 38.65 | 40.84 | 925.57 | 11685.00 | 6881.90 | 7049.10 | 1.03 | 110.53 | 104.16 | 3.08 | 1.87 | 6.64 | 20.87 | 0.00 | 5.15 | 1123.30 | 657.63 | 2.41 | 0.00 | 653.18 | 295.24 | 1470.70 | 558.83 |
| 428089 | K2P2 | Dync1li1 | Dync1li1_em1_del | +/- | F | 428089 | 8/18/2016 | 163.83 | 587.88 | 126.76 | 24.82 | 2.47 | 6.61 | 10.67 | 377.53 | 242.06 | 90.05 | 1246.04 | 333.63 | 20.55 | 138.79 | 160.38 | 161.39 | 364.89 | 18.82 | 430.14 | 1609.14 | 74.29 | 20.52 | 11.16 | 0.91 | 9.29 | 0.56 | 59.68 | 0.00 | 10.98 | 53.03 | 66.83 | 605.89 | 7189.48 | 4963.64 | 5472.39 | 1.26 | 160.66 | 95.17 | 3.63 | 1.91 | 4.08 | 18.36 | 1.62 | 5.84 | 1170.88 | 563.24 | 1.60 | 6.69 | 281.97 | 17.09 | 2418.60 | 864.87 |
| 420778 | K2P2 | Dync1li1 | Dync1li1_em1_del | +/- | F | 420778 | 8/5/2016 | 232.51 | 601.91 | 126.97 | 31.35 | 5.60 | 6.44 | 8.74 | 275.53 | 204.48 | 81.35 | 1486.67 | 410.97 | 29.91 | 113.39 | 121.31 | 107.19 | 206.20 | 28.52 | 429.19 | 1739.77 | 110.74 | 28.23 | 9.58 | 0.94 | 13.17 | 1.18 | 65.58 | 2.79 | 11.29 | 26.76 | 50.87 | 707.59 | 5603.53 | 3377.50 | 3684.41 | 1.19 | 47.71 | 124.98 | 5.13 | 1.51 | 2.36 | 14.54 | 0.00 | 6.21 | 1112.56 | 452.88 | 1.91 | 6.88 | 400.08 | 18.09 | 3816.38 | 1411.71 |
| 428090 | K2P2 | Dync1li1 | Dync1li1_em1_del | +/- | F | 428090 | 8/18/2016 | 240.95 | 660.62 | 165.33 | 42.19 | 4.72 | 8.40 | 4.44 | 167.89 | 58.85 | 58.43 | 1219.55 | 518.86 | 25.04 | 126.70 | 152.17 | 113.24 | 233.67 | 28.55 | 481.54 | 1741.98 | 98.79 | 21.08 | 10.23 | 0.70 | 11.31 | 0.77 | 64.30 | 0.00 | 9.39 | 30.69 | 52.57 | 877.96 | 7267.52 | 7023.10 | 6956.49 | 1.55 | 293.48 | 102.38 | 1.41 | 1.88 | 4.14 | 17.52 | 1.56 | 6.64 | 1309.82 | 629.73 | 0.00 | 7.69 | 510.64 | 28.07 | 891.16 | 370.49 |
| 420676 | K2P2 | Dync1li1 | Dync1li1_em1_del | +/- | M | 420676 | 8/3/2016 | 239.14 | 618.12 | 96.23 | 28.58 | 6.62 | 9.69 | 11.28 | 398.41 | 345.46 | 139.01 | 806.81 | 785.33 | 76.26 | 618.56 | 478.18 | 600.64 | 1255.76 | 28.62 | 438.90 | 497.62 | 89.74 | 35.04 | 15.46 | 0.80 | 10.93 | 0.96 | 60.58 | 0.00 | 35.41 | 26.35 | 35.54 | 634.16 | 7184.63 | 6693.53 | 5686.35 | 1.21 | 111.04 | 144.57 | 10.95 | 1.79 | 3.82 | 25.40 | 2.23 | 5.71 | 946.24 | 583.24 | 2.11 | 0.00 | 863.84 | 1126.60 | 8196.33 | 3660.44 |
| 420674 | K2P2 | Dync1li1 | Dync1li1_em1_del | +/- | M | 420674 | 8/3/2016 | 258.93 | 682.31 | 140.72 | 37.32 | 5.44 | 10.52 | 20.25 | 365.87 | 366.97 | 162.78 | 1028.26 | 766.94 | 68.77 | 598.11 | 917.30 | 644.88 | 1570.79 | 34.30 | 468.38 | 501.04 | 158.30 | 21.20 | 10.86 | 1.25 | 17.18 | 1.33 | 51.89 | 0.00 | 27.41 | 37.57 | 34.65 | 843.39 | 14092.90 | 10934.14 | 9816.70 | 2.96 | 300.93 | 128.30 | 3.61 | 1.82 | 6.61 | 22.75 | 0.00 | 5.13 | 1069.55 | 747.73 | 0.00 | 1.60 | 778.19 | 4962.86 | 1391.27 | 589.04 |
| 420673 | K2P2 | Dync1li1 | Dync1li1_em1_del | +/- | M | 420673 | 8/3/2016 | 266.44 | 633.29 | 111.87 | 24.81 | 3.58 | 11.31 | 8.46 | 305.86 | 203.00 | 93.42 | 1115.67 | 634.92 | 51.03 | 490.28 | 409.75 | 448.87 | 847.32 | 29.30 | 472.06 | 521.38 | 116.02 | 33.70 | 11.30 | 1.03 | 11.43 | 0.90 | 61.57 | 0.00 | 30.55 | 29.70 | 35.85 | 761.37 | 6341.86 | 5412.01 | 4333.30 | 1.10 | 96.86 | 159.81 | 2.64 | 1.42 | 4.38 | 14.73 | 1.80 | 5.24 | 1036.37 | 579.66 | 0.92 | 1.08 | 723.90 | 473.53 | 1572.41 | 640.13 |
| 410978 | KOMP2 | G6pd2 | G6pd2_em1_del | -/- | F | 410978 | 6/16/2016 | 233.34 | 578.35 | 187.48 | 37.14 | 5.16 | 8.10 | 7.62 | 408.07 | 255.12 | 97.49 | 1807.35 | 571.13 | 26.28 | 167.90 | 171.16 | 143.40 | 269.75 | 31.52 | 575.95 | 1305.49 | 93.94 | 30.58 | 7.85 | 1.03 | 10.82 | 1.12 | 78.04 | 0.00 | 18.70 | 52.32 | 49.14 | 736.09 | 13029.06 | 7659.30 | 7791.37 | 2.93 | 226.71 | 101.22 | 3.08 | 1.82 | 0.00 | 21.80 | 0.00 | 6.25 | 1218.01 | 562.09 | 1.24 | 6.46 | 399.37 | 184.36 | 3085.14 | 1446.40 |
| 410980 | KOMP2 | G6pd2 | G6pd2_em1_del | -/- | F | 410980 | 6/16/2016 | 194.85 | 610.63 | 151.51 | 33.96 | 4.97 | 10.25 | 15.19 | 473.81 | 266.32 | 106.78 | 1657.66 | 471.28 | 24.68 | 154.10 | 156.68 | 158.62 | 219.81 | 28.87 | 480.74 | 1231.50 | 103.16 | 32.86 | 9.56 | 0.64 | 11.54 | 1.13 | 78.11 | 1.36 | 17.09 | 46.73 | 40.55 | 837.93 | 7504.21 | 6154.31 | 5898.82 | 2.33 | 111.13 | 119.84 | 2.61 | 1.92 | 3.40 | 21.15 | 1.80 | 5.13 | 1253.75 | 611.49 | 1.16 | 8.53 | 378.73 | 27.92 | 2065.30 | 688.92 |
| 410975 | KOMP2 | G6pd2 | G6pd2_em1_del | -/- | F | 410975 | 6/16/2016 | 219.09 | 586.54 | 157.20 | 43.95 | 6.88 | 10.11 | 7.33 | 282.34 | 156.70 | 84.51 | 1517.28 | 611.62 | 26.28 | 119.54 | 126.45 | 99.08 | 195.03 | 31.79 | 543.69 | 1532.28 | 85.74 | 32.69 | 9.38 | 1.06 | 10.30 | 1.09 | 60.17 | 0.00 | 11.37 | 39.01 | 41.57 | 685.75 | 8299.88 | 6637.86 | 7597.24 | 2.24 | 147.86 | 132.55 | 4.02 | 1.62 | 0.00 | 18.90 | 1.09 | 5.07 | 1331.71 | 561.34 | 0.00 | 7.55 | 366.42 | 192.86 | 3378.15 | 1108.68 |
| 410881 | KOMP2 | G6pd2 | G6pd2_em1_del | -/- | M | 410881 | 6/16/2016 | 222.87 | 738.23 | 109.30 | 26.44 | 5.25 | 11.69 | 6.23 | 478.54 | 231.66 | 108.45 | 1036.01 | 506.93 | 58.45 | 559.60 | 610.85 | 515.92 | 923.00 | 34.75 | 519.38 | 489.67 | 101.01 | 30.04 | 11.53 | 0.97 | 11.55 | 1.01 | 67.90 | 0.00 | 44.01 | 23.28 | 37.13 | 761.54 | 5279.10 | 4292.49 | 3700.12 | 0.85 | 77.68 | 115.41 | 7.28 | 1.93 | 5.08 | 22.05 | 2.71 | 3.69 | 999.25 | 618.86 | 1.27 | 0.95 | 686.08 | 755.93 | 4905.05 | 2066.80 |
| 420416 | KOMP2 | G6pd2 | G6pd2_em1_del | -/- | M | 420416 | 8/3/2016 | 282.83 | 695.43 | 126.23 | 24.69 | 3.50 | 9.86 | 9.81 | 563.53 | 310.58 | 134.36 | 1056.12 | 578.12 | 45.55 | 405.76 | 478.92 | 345.00 | 811.93 | 26.50 | 461.34 | 740.04 | 138.38 | 36.32 | 13.98 | 1.06 | 11.59 | 0.73 | 69.92 | 0.00 | 34.06 | 27.42 | 41.09 | 725.71 | 8945.62 | 6125.14 | 6561.36 | 0.81 | 157.42 | 131.04 | 4.25 | 1.57 | 4.92 | 15.39 | 2.27 | 4.43 | 929.12 | 629.07 | 1.37 | 1.10 | 670.24 | 258.06 | 2258.67 | 1020.10 |
| 410882 | KOMP2 | G6pd2 | G6pd2_em1_del | -/- | M | 410882 | 6/16/2016 | 231.97 | 659.86 | 130.77 | 28.42 | 4.84 | 11.75 | 16.46 | 362.59 | 247.12 | 125.70 | 1020.81 | 697.40 | 58.88 | 532.88 | 771.37 | 707.86 | 1021.21 | 24.36 | 475.30 | 460.90 | 116.29 | 32.87 | 14.28 | 0.91 | 12.82 | 0.97 | 67.76 | 0.00 | 48.77 | 47.45 | 38.17 | 776.55 | 9972.60 | 8181.70 | 8698.83 | 1.45 | 182.42 | 146.27 | 0.00 | 1.79 | 5.30 | 18.51 | 1.49 | 6.09 | 1171.67 | 555.89 | 0.00 | 1.12 | 706.34 | 1892.32 | 604.99 | 265.13 |
| 39183 | NorCOMM2 | Galc | Galc_tm1b_A06 | +/- | F | 39183 | 12/4/2013 | 169.91 | 652.41 | 158.01 | 24.65 | 3.36 | 8.40 | 12.27 | 218.73 | 252.29 | 69.83 | 1264.66 | 403.32 | 24.84 | 105.27 | 101.38 | 124.98 | 238.57 | 18.24 | 422.56 | 1536.63 | 102.46 | 31.17 | 18.01 | 1.08 | 12.08 | 1.04 | 72.85 | 0.00 | 14.38 | 30.99 | 49.18 | 714.24 | 6558.44 | 5163.69 | 6182.04 | 1.86 | 104.80 | 90.41 | 7.82 | 1.83 | 5.09 | 17.50 | 0.00 | 4.19 | 1145.53 | 572.19 | 1.40 | 6.70 | 324.31 | 29.34 | 4806.38 | 1872.51 |
| 40391 | NorCOMM2 | Galc | Galc_tm1b_A06 | +/- | F | 40391 | 12/16/2013 | 179.25 | 789.37 | 167.00 | 50.08 | 5.62 | 11.48 | 7.54 | 336.55 | 200.48 | 74.04 | 1427.66 | 573.43 | 31.04 | 136.39 | 205.71 | 146.52 | 245.88 | 25.44 | 480.67 | 1693.51 | 114.50 | 48.63 | 16.56 | 1.03 | 11.14 | 0.91 | 57.06 | 3.28 | 17.40 | 41.02 | 54.90 | 831.99 | 10774.40 | 6089.18 | 5518.82 | 1.90 | 124.13 | 147.92 | 17.67 | 1.68 | 4.55 | 22.02 | 1.95 | 5.94 | 1510.46 | 575.26 | 4.28 | 5.98 | 403.94 | 13.16 | 16056.05 | 5930.59 |
| 39181 | NorCOMM2 | Galc | Galc_tm1b_A06 | +/- | F | 39181 | 12/4/2013 | 226.24 | 664.50 | 192.97 | 35.12 | 2.75 | 9.38 | 7.52 | 246.37 | 153.15 | 77.30 | 1425.11 | 569.85 | 26.50 | 116.90 | 155.53 | 131.58 | 237.28 | 24.90 | 494.41 | 1543.74 | 90.81 | 19.77 | 19.44 | 1.27 | 11.90 | 0.90 | 78.15 | 0.00 | 15.71 | 33.41 | 44.59 | 791.62 | 9202.30 | 5967.69 | 5659.41 | 1.60 | 81.72 | 104.51 | 6.41 | 2.20 | 3.67 | 14.72 | 2.90 | 5.06 | 1388.65 | 554.79 | 1.34 | 6.68 | 378.05 | 40.66 | 6983.22 | 2605.62 |
| 43707 | NorCOMM2 | Galc | Galc_tm1b_A06 | +/- | M | 43707 | 12/30/2013 | 263.60 | 795.91 | 126.15 | 29.38 | 3.87 | 9.93 | 9.05 | 454.67 | 288.52 | 90.80 | 983.18 | 757.69 | 63.57 | 487.43 | 620.81 | 466.69 | 962.51 | 25.98 | 474.36 | 644.80 | 123.09 | 34.32 | 18.22 | 1.16 | 11.30 | 1.03 | 85.08 | 4.54 | 52.27 | 29.87 | 32.24 | 818.22 | 6751.72 | 6298.76 | 6448.87 | 1.63 | 97.88 | 159.96 | 6.69 | 2.15 | 6.93 | 24.51 | 3.22 | 4.90 | 1199.86 | 673.16 | 1.26 | 0.86 | 779.77 | 1328.34 | 3407.53 | 1738.01 |
| 40288 | NorCOMM2 | Galc | Galc_tm1b_A06 | +/- | M | 40288 | 12/16/2013 | 215.95 | 840.14 | 137.16 | 25.15 | 6.75 | 11.88 | 11.04 | 464.37 | 206.81 | 90.37 | 721.37 | 727.76 | 57.43 | 579.47 | 639.05 | 498.18 | 881.16 | 25.92 | 565.06 | 812.99 | 102.64 | 32.00 | 10.11 | 1.24 | 9.36 | 0.81 | 60.56 | 0.00 | 38.83 | 31.78 | 42.52 | 822.51 | 8906.18 | 6777.76 | 5572.54 | 0.87 | 95.30 | 110.22 | 7.04 | 2.01 | 4.98 | 15.77 | 0.00 | 3.74 | 1194.69 | 768.00 | 0.66 | 0.42 | 863.50 | 52.73 | 4490.43 | 2094.25 |
| 43708 | NorCOMM2 | Galc | Galc_tm1b_A06 | +/- | M | 43708 | 12/30/2013 | 248.83 | 855.71 | 151.99 | 44.24 | 4.02 | 8.00 | 10.68 | 482.92 | 459.24 | 134.75 | 874.51 | 965.99 | 79.52 | 717.39 | 712.04 | 741.13 | 1672.37 | 25.90 | 490.61 | 682.25 | 91.88 | 28.20 | 19.79 | 1.16 | 12.02 | 1.07 | 64.93 | 0.00 | 45.62 | 35.83 | 34.27 | 826.23 | 10577.75 | 6102.70 | 7966.76 | 1.48 | 110.41 | 153.06 | 3.42 | 1.94 | 8.73 | 23.86 | 4.09 | 4.86 | 1222.01 | 672.55 | 0.88 | 0.73 | 694.19 | 1087.85 | 2506.77 | 985.54 |
| 38377 | NorCOMM2 | Gnpda1 | Gnpda1_tm1b_A05 | +/- | F | 38377 | 12/11/2013 | 196.96 | 798.87 | 150.98 | 42.98 | 3.90 | 8.29 | 10.03 | 214.96 | 139.22 | 63.43 | 1425.80 | 499.12 | 26.06 | 124.01 | 135.00 | 112.61 | 214.40 | 22.59 | 405.82 | 1755.50 | 94.43 | 37.70 | 14.49 | 0.99 | 13.39 | 0.95 | 74.79 | 1.29 | 15.70 | 43.57 | 36.94 | 837.25 | 8226.00 | 5058.20 | 5276.40 | 1.84 | 191.17 | 129.08 | 6.50 | 1.46 | 3.78 | 12.69 | 0.00 | 7.59 | 1102.80 | 733.61 | 1.16 | 6.85 | 466.37 | 27.15 | 4696.70 | 1628.80 |
| 37870 | NorCOMM2 | Gnpda1 | Gnpda1_tm1b_A05 | +/- | F | 37870 | 11/28/2013 | 222.79 | 925.41 | 127.83 | 24.76 | 6.48 | 9.02 | 6.71 | 187.71 | 135.73 | 68.69 | 1265.70 | 422.88 | 31.38 | 156.72 | 196.14 | 162.61 | 230.66 | 28.30 | 427.65 | 1824.60 | 108.15 | 27.32 | 15.64 | 1.06 | 9.79 | 1.01 | 85.71 | 4.54 | 15.19 | 33.13 | 42.22 | 905.68 | 5236.10 | 3383.40 | 4273.20 | 2.01 | 38.73 | 103.06 | 17.24 | 1.80 | 3.99 | 14.73 | 3.62 | 4.50 | 1353.10 | 523.07 | 1.21 | 3.47 | 311.24 | 13.07 | 19194.00 | 6565.00 |
| 37871 | NorCOMM2 | Gnpda1 | Gnpda1_tm1b_A05 | +/- | F | 37871 | 11/28/2013 | 189.56 | 801.46 | 99.97 | 36.42 | 3.57 | 6.33 | 5.76 | 118.70 | 110.96 | 66.16 | 1326.60 | 500.77 | 27.74 | 197.77 | 243.18 | 171.00 | 193.43 | 27.10 | 383.97 | 1288.10 | 86.92 | 41.22 | 13.19 | 0.89 | 10.43 | 1.19 | 46.94 | 1.83 | 21.61 | 30.76 | 45.62 | 751.47 | 6968.00 | 3973.70 | 4549.20 | 2.20 | 34.17 | 97.92 | 9.94 | 1.68 | 4.24 | 20.26 | 2.52 | 4.92 | 1254.30 | 656.58 | 0.82 | 6.93 | 274.10 | 275.51 | 6829.00 | 2364.30 |
| 38275 | NorCOMM2 | Gnpda1 | Gnpda1_tm1b_A05 | +/- | M | 38275 | 12/11/2013 | 225.37 | 1002.90 | 97.06 | 31.45 | 3.58 | 11.78 | 17.04 | 412.23 | 267.36 | 86.57 | 1187.20 | 772.90 | 93.90 | 602.04 | 719.60 | 619.34 | 1357.40 | 40.27 | 494.31 | 575.62 | 131.14 | 33.78 | 20.47 | 1.06 | 13.57 | 1.42 | 60.52 | 2.32 | 59.94 | 35.95 | 28.33 | 972.24 | 8567.50 | 7056.90 | 7388.30 | 1.54 | 64.33 | 128.06 | 8.92 | 1.57 | 6.96 | 18.77 | 3.06 | 4.86 | 965.41 | 597.13 | 1.01 | 0.81 | 865.15 | 7198.60 | 6318.10 | 2778.00 |
| 37768 | NorCOMM2 | Gnpda1 | Gnpda1_tm1b_A05 | +/- | M | 37768 | 11/27/2013 | 283.89 | 793.90 | 93.92 | 41.73 | 7.98 | 9.91 | 14.55 | 256.65 | 190.64 | 90.94 | 989.11 | 651.00 | 81.79 | 655.88 | 1134.40 | 676.19 | 1333.10 | 31.88 | 372.37 | 482.04 | 100.52 | 29.21 | 18.22 | 1.07 | 12.98 | 1.03 | 49.98 | 5.60 | 45.44 | 33.24 | 27.02 | 1038.10 | 11371.00 | 7788.20 | 8902.10 | 3.21 | 158.95 | 116.43 | 10.14 | 2.45 | 4.99 | 28.29 | 1.82 | 4.94 | 1137.70 | 673.59 | 1.30 | 0.74 | 667.92 | 6903.00 | 6882.30 | 2805.50 |
| 37769 | NorCOMM2 | Gnpda1 | Gnpda1_tm1b_A05 | +/- | M | 37769 | 11/28/2013 | 244.02 | 1053.00 | 124.73 | 45.63 | 10.44 | 12.21 | 9.27 | 93.10 | 82.70 | 88.90 | 873.04 | 837.03 | 68.98 | 619.43 | 992.45 | 659.76 | 1099.80 | 28.56 | 434.11 | 762.45 | 118.20 | 47.26 | 20.27 | 1.22 | 13.49 | 1.23 | 81.13 | 0.00 | 41.00 | 35.09 | 34.22 | 959.17 | 11267.00 | 6268.60 | 7951.30 | 1.49 | 121.75 | 118.76 | 19.97 | 1.90 | 6.79 | 22.88 | 4.17 | 5.44 | 1255.50 | 830.45 | 2.09 | 0.52 | 713.46 | 573.52 | 19741.00 | 6284.50 |
| 116005 | NorCOMM2 | Idh1 | Idh1_tm1b_H02 | -/- | F | 116005 | 6/5/2014 | 230.45 | 672.35 | 131.16 | 35.54 | 6.94 | 8.37 | 9.55 | 396.69 | 295.26 | 96.23 | 1313.23 | 528.16 | 24.83 | 189.70 | 242.11 | 218.25 | 313.06 | 26.58 | 585.09 | 1473.74 | 95.33 | 21.68 | 15.76 | 0.96 | 9.95 | 0.87 | 63.36 | 6.36 | 20.42 | 32.65 | 45.68 | 727.41 | 5314.32 | 4265.43 | 3791.54 | 1.85 | 28.23 | 111.88 | 8.75 | 2.49 | 5.89 | 22.85 | 0.00 | 5.26 | 1150.18 | 624.27 | 3.72 | 7.02 | 411.58 | 37.96 | 8169.14 | 3526.27 |
| 116097 | NorCOMM2 | Idh1 | Idh1_tm1b_H02 | -/- | F | 116097 | 6/5/2014 | 242.13 | 643.07 | 134.26 | 44.13 | 7.76 | 8.67 | 18.65 | 402.77 | 363.76 | 103.84 | 1936.87 | 602.97 | 27.29 | 166.73 | 191.13 | 166.32 | 248.37 | 30.03 | 516.93 | 1434.44 | 136.00 | 28.00 | 18.10 | 1.17 | 10.59 | 1.07 | 73.62 | 3.63 | 21.85 | 37.66 | 44.57 | 902.36 | 9207.24 | 6203.91 | 5612.04 | 2.34 | 67.17 | 121.57 | 6.25 | 2.07 | 3.74 | 19.35 | 2.24 | 5.25 | 1272.73 | 719.65 | 1.48 | 8.41 | 412.99 | 150.63 | 3837.35 | 1633.32 |
| 116002 | NorCOMM2 | Idh1 | Idh1_tm1b_H02 | -/- | F | 116002 | 6/5/2014 | 194.39 | 581.32 | 162.43 | 53.52 | 7.76 | 10.97 | 19.19 | 370.65 | 279.09 | 115.34 | 1724.74 | 626.47 | 24.51 | 181.43 | 207.21 | 138.82 | 240.39 | 30.05 | 454.75 | 1479.76 | 106.90 | 24.49 | 15.83 | 1.08 | 8.68 | 1.11 | 85.50 | 3.86 | 18.54 | 34.08 | 42.99 | 753.50 | 7719.16 | 5226.25 | 6611.45 | 1.98 | 80.18 | 108.18 | 5.79 | 2.38 | 4.72 | 20.08 | 1.96 | 5.08 | 1110.86 | 537.21 | 1.69 | 7.16 | 403.44 | 44.81 | 4446.87 | 1487.46 |
| 172743 | NorCOMM2 | Idh1 | Idh1_tm1b_H02 | -/- | M | 172743 | 10/16/2014 | 230.24 | 685.72 | 134.99 | 26.98 | 3.21 | 11.86 | 12.52 | 502.90 | 274.18 | 109.28 | 1126.90 | 657.55 | 58.20 | 656.82 | 641.35 | 501.73 | 993.65 | 23.89 | 465.74 | 664.18 | 90.14 | 29.20 | 16.07 | 1.17 | 9.05 | 1.15 | 65.42 | 0.00 | 38.69 | 46.06 | 35.36 | 843.71 | 9595.38 | 6141.55 | 7281.23 | 1.88 | 202.68 | 137.23 | 2.44 | 2.32 | 4.16 | 22.13 | 0.00 | 5.28 | 979.41 | 694.96 | 1.18 | 0.81 | 635.43 | 684.94 | 972.50 | 469.61 |
| 172742 | NorCOMM2 | Idh1 | Idh1_tm1b_H02 | -/- | M | 172742 | 10/16/2014 | 244.73 | 729.41 | 141.59 | 37.76 | 5.83 | 5.98 | 17.75 | 615.19 | 278.67 | 99.90 | 1019.84 | 772.40 | 72.88 | 730.78 | 719.33 | 701.97 | 1140.81 | 29.78 | 499.35 | 574.49 | 99.60 | 27.09 | 20.24 | 1.12 | 10.18 | 0.90 | 91.45 | 2.69 | 42.13 | 34.87 | 34.40 | 718.11 | 9055.60 | 6380.19 | 6811.66 | 1.48 | 150.75 | 126.00 | 3.61 | 2.21 | 4.95 | 26.99 | 3.44 | 5.11 | 950.18 | 712.25 | 1.49 | 0.65 | 728.33 | 551.95 | 3097.45 | 1072.20 |
| 172741 | NorCOMM2 | Idh1 | Idh1_tm1b_H02 | -/- | M | 172741 | 10/16/2014 | 240.42 | 860.47 | 130.74 | 32.34 | 2.60 | 10.32 | 13.50 | 454.24 | 236.71 | 108.16 | 1098.83 | 670.16 | 61.28 | 516.78 | 616.78 | 483.26 | 895.58 | 26.38 | 365.20 | 804.50 | 88.75 | 28.68 | 22.25 | 1.34 | 10.31 | 1.50 | 73.13 | 2.17 | 40.90 | 33.80 | 36.44 | 824.19 | 8366.40 | 6314.70 | 6473.27 | 1.51 | 225.87 | 106.27 | 1.95 | 1.53 | 2.63 | 24.92 | 0.00 | 4.40 | 1049.38 | 551.97 | 0.79 | 0.99 | 549.92 | 232.12 | 969.84 | 357.14 |
| 435700 | KOMP2 | Iqgap1 | Iqgap1_tm1b_A11 | -/- | F | 435700 | 9/8/2016 | 216.78 | 621.64 | 180.17 | 41.07 | 5.07 | 10.27 | 8.52 | 476.01 | 292.84 | 119.89 | 1626.36 | 504.46 | 23.83 | 171.66 | 189.75 | 167.17 | 214.93 | 32.53 | 444.04 | 1033.72 | 93.05 | 28.84 | 9.00 | 1.00 | 9.43 | 1.50 | 78.78 | 0.00 | 15.08 | 27.88 | 50.58 | 695.61 | 6801.13 | 5612.87 | 5094.34 | 1.72 | 82.14 | 131.58 | 7.51 | 1.95 | 0.00 | 16.88 | 1.31 | 4.16 | 1184.53 | 566.07 | 1.11 | 4.83 | 396.37 | 122.35 | 3715.25 | 1715.87 |
| 506105 | KOMP2 | Iqgap1 | Iqgap1_tm1b_A11 | -/- | F | 506105 | 3/23/2017 | 249.16 | 616.27 | 172.95 | 32.31 | 5.41 | 9.90 | 9.37 | 301.07 | 196.63 | 79.75 | 1364.05 | 505.00 | 28.23 | 132.84 | 151.25 | 123.47 | 221.55 | 21.19 | 474.95 | 1238.80 | 113.43 | 25.37 | 9.27 | 1.24 | 10.92 | 1.09 | 53.10 | 0.00 | 10.51 | 30.25 | 53.82 | 578.86 | 7912.29 | 6093.08 | 6602.75 | 1.45 | 87.52 | 119.37 | 14.88 | 1.77 | 0.00 | 21.18 | 0.00 | 4.31 | 1158.22 | 587.49 | 0.00 | 6.20 | 481.60 | 70.63 | 12823.95 | 4830.91 |
| 435799 | KOMP2 | Iqgap1 | Iqgap1_tm1b_A11 | -/- | F | 435799 | 9/8/2016 | 237.70 | 581.05 | 169.22 | 35.73 | 5.31 | 10.79 | 14.13 | 498.58 | 219.91 | 108.07 | 2061.53 | 556.01 | 22.95 | 171.97 | 161.81 | 156.97 | 227.38 | 27.87 | 454.24 | 1363.69 | 121.53 | 33.37 | 7.71 | 1.14 | 8.24 | 1.02 | 64.30 | 0.00 | 14.31 | 38.93 | 55.45 | 700.45 | 9847.14 | 6588.76 | 6519.51 | 2.30 | 75.35 | 162.80 | 3.33 | 1.98 | 0.00 | 17.54 | 0.00 | 5.14 | 1220.66 | 648.29 | 0.00 | 6.69 | 441.43 | 81.95 | 2205.69 | 870.26 |
| 435695 | KOMP2 | Iqgap1 | Iqgap1_tm1b_A11 | -/- | M | 435695 | 9/8/2016 | 346.34 | 764.53 | 159.34 | 32.30 | 4.34 | 10.62 | 8.38 | 533.36 | 322.25 | 113.65 | 1314.61 | 564.15 | 58.84 | 577.32 | 636.19 | 554.79 | 857.43 | 30.09 | 488.75 | 857.84 | 153.07 | 35.88 | 10.18 | 1.16 | 10.28 | 1.24 | 93.14 | 0.00 | 25.94 | 32.27 | 49.01 | 823.68 | 8591.33 | 8958.85 | 8298.53 | 1.97 | 64.47 | 188.72 | 3.90 | 1.95 | 0.00 | 22.19 | 2.33 | 3.72 | 977.17 | 708.42 | 0.95 | 0.00 | 687.85 | 814.97 | 2326.18 | 830.83 |
| 487462 | KOMP2 | Iqgap1 | Iqgap1_tm1b_A11 | -/- | M | 487462 | 1/4/2017 | 179.69 | 703.47 | 138.20 | 25.46 | 5.71 | 8.68 | 12.03 | 547.56 | 358.05 | 131.31 | 1210.04 | 686.40 | 58.83 | 581.61 | 576.04 | 550.97 | 865.31 | 34.27 | 501.60 | 666.07 | 82.41 | 21.23 | 13.84 | 1.65 | 10.35 | 1.06 | 66.77 | 0.00 | 12.42 | 31.94 | 47.35 | 776.23 | 10828.09 | 8705.78 | 8068.74 | 1.68 | 158.99 | 150.85 | 2.27 | 1.79 | 16.11 | 45.25 | 5.67 | 4.66 | 908.97 | 809.21 | 1.77 | 0.00 | 498.72 | 240.93 | 2296.08 | 798.66 |
| 435693 | KOMP2 | Iqgap1 | Iqgap1_tm1b_A11 | -/- | M | 435693 | 9/8/2016 | 247.12 | 699.10 | 149.68 | 27.49 | 4.43 | 12.39 | 13.83 | 646.18 | 324.29 | 130.45 | 1424.77 | 793.12 | 52.95 | 558.54 | 434.29 | 535.97 | 848.24 | 36.33 | 472.74 | 661.33 | 137.32 | 40.28 | 12.06 | 0.90 | 11.10 | 1.13 | 70.33 | 0.00 | 22.31 | 37.79 | 42.50 | 857.20 | 10520.47 | 7823.41 | 9958.08 | 1.11 | 136.48 | 144.92 | 2.93 | 1.44 | 5.64 | 24.96 | 0.00 | 3.48 | 977.51 | 793.26 | 0.00 | 0.00 | 677.17 | 1020.66 | 2599.73 | 998.42 |
| 107190 | NorCOMM2 | Lmbrd1 | Lmbrd1_tm1b_A10 | +/- | F | 107190 | 5/22/2014 | 194.93 | 718.64 | 120.81 | 41.80 | 7.19 | 8.06 | 9.48 | 314.74 | 213.74 | 83.47 | 1382.49 | 603.26 | 28.55 | 157.20 | 131.01 | 125.21 | 222.82 | 23.26 | 354.72 | 1938.95 | 102.51 | 29.93 | 16.82 | 1.24 | 13.48 | 1.14 | 68.27 | 2.87 | 12.14 | 20.17 | 45.74 | 648.05 | 5355.65 | 3406.94 | 3189.37 | 2.48 | 26.62 | 84.35 | 11.10 | 1.74 | 4.27 | 17.79 | 3.27 | 4.89 | 1479.21 | 776.76 | 2.05 | 5.13 | 415.56 | 105.65 | 11360.80 | 3772.43 |
| 111493 | NorCOMM2 | Lmbrd1 | Lmbrd1_tm1b_A10 | +/- | F | 111493 | 5/27/2014 | 226.84 | 644.54 | 151.92 | 35.80 | 6.78 | 9.73 | 21.65 | 526.25 | 223.84 | 91.41 | 1888.30 | 595.24 | 26.41 | 199.66 | 226.28 | 180.43 | 289.83 | 23.06 | 520.07 | 1195.02 | 123.94 | 27.47 | 15.14 | 0.92 | 11.62 | 0.91 | 76.59 | 3.58 | 24.18 | 31.78 | 36.52 | 817.98 | 7860.60 | 4877.42 | 5807.76 | 2.66 | 125.81 | 105.24 | 6.31 | 1.96 | 3.84 | 17.32 | 0.00 | 4.66 | 1278.42 | 624.84 | 2.89 | 6.86 | 468.33 | 33.62 | 4938.68 | 2113.69 |
| 107189 | NorCOMM2 | Lmbrd1 | Lmbrd1_tm1b_A10 | +/- | F | 107189 | 5/21/2014 | 196.36 | 653.18 | 154.03 | 34.05 | 3.36 | 10.95 | 8.75 | 368.83 | 228.46 | 94.18 | 1344.79 | 627.08 | 24.95 | 163.72 | 200.90 | 188.86 | 269.06 | 25.11 | 556.58 | 1704.96 | 126.80 | 21.47 | 16.44 | 1.02 | 10.97 | 1.27 | 73.82 | 2.12 | 17.72 | 29.62 | 45.36 | 743.66 | 7122.40 | 5180.13 | 5083.06 | 2.47 | 59.78 | 139.66 | 5.14 | 1.57 | 4.41 | 10.92 | 1.67 | 5.97 | 1474.03 | 753.77 | 1.39 | 7.35 | 441.81 | 28.12 | 4705.49 | 1390.55 |
| 72464 | NorCOMM2 | Lmbrd1 | Lmbrd1_tm1b_A10 | +/- | M | 72464 | 3/12/2014 | 226.26 | 736.52 | 106.30 | 32.94 | 4.01 | 8.16 | 14.76 | 301.07 | 171.66 | 93.97 | 996.40 | 675.20 | 50.90 | 513.47 | 725.53 | 507.73 | 889.40 | 22.51 | 434.29 | 561.02 | 94.59 | 35.38 | 15.06 | 1.37 | 8.87 | 1.16 | 71.10 | 2.01 | 49.88 | 30.01 | 30.64 | 698.49 | 6848.15 | 4275.12 | 4394.25 | 1.08 | 33.33 | 99.67 | 7.43 | 2.15 | 6.96 | 14.71 | 3.43 | 3.57 | 1084.67 | 546.27 | 1.29 | 0.56 | 613.01 | 1428.01 | 6805.64 | 2383.68 |
| 72463 | NorCOMM2 | Lmbrd1 | Lmbrd1_tm1b_A10 | +/- | M | 72463 | 3/12/2014 | 243.65 | 763.37 | 103.83 | 36.35 | 6.67 | 11.43 | 11.76 | 432.10 | 342.11 | 115.85 | 1379.75 | 1140.92 | 68.55 | 909.96 | 852.14 | 668.74 | 1351.40 | 27.22 | 469.09 | 590.10 | 135.42 | 26.73 | 23.17 | 1.64 | 15.85 | 0.95 | 69.02 | 3.51 | 70.18 | 30.97 | 24.26 | 783.25 | 9460.04 | 7279.35 | 9909.90 | 3.91 | 93.69 | 138.76 | 7.41 | 2.51 | 4.99 | 27.28 | 2.76 | 5.74 | 835.98 | 599.29 | 1.01 | 1.02 | 486.87 | 12590.08 | 6495.76 | 2121.17 |
| 72460 | NorCOMM2 | Lmbrd1 | Lmbrd1_tm1b_A10 | +/- | M | 72460 | 3/12/2014 | 256.59 | 733.58 | 94.85 | 28.89 | 6.10 | 9.01 | 11.44 | 375.05 | 273.19 | 114.09 | 940.13 | 827.76 | 64.60 | 637.01 | 722.73 | 555.59 | 868.83 | 25.92 | 463.57 | 625.80 | 97.17 | 24.78 | 18.87 | 1.16 | 11.18 | 0.93 | 55.53 | 2.56 | 54.73 | 26.86 | 31.97 | 739.23 | 4259.39 | 2890.81 | 3440.57 | 0.94 | 26.65 | 119.05 | 20.10 | 1.65 | 6.58 | 18.36 | 3.21 | 4.09 | 994.08 | 583.95 | 2.04 | 0.51 | 797.46 | 149.63 | 15561.71 | 6710.26 |
| 213490 | NorCOMM2 | Mfap4 | Mfap4_tm1b_C11 | -/- | F | 213490 | 1/20/2015 | 186.10 | 654.86 | 145.08 | 24.34 | 3.70 | 9.22 | 11.45 | 608.77 | 397.57 | 113.72 | 1788.05 | 632.47 | 28.19 | 194.46 | 211.22 | 160.03 | 285.61 | 25.90 | 489.01 | 982.51 | 113.70 | 27.76 | 16.01 | 0.79 | 11.44 | 0.81 | 71.12 | 0.84 | 28.56 | 29.47 | 39.52 | 737.03 | 8251.68 | 5370.64 | 5583.70 | 2.38 | 85.81 | 106.02 | 2.35 | 2.06 | 3.44 | 11.87 | 0.00 | 5.00 | 1040.27 | 556.81 | 1.00 | 7.08 | 301.01 | 174.72 | 1173.76 | 472.74 |
| 213491 | NorCOMM2 | Mfap4 | Mfap4_tm1b_C11 | -/- | F | 213491 | 1/21/2015 | 223.92 | 720.80 | 162.78 | 40.66 | 3.42 | 15.25 | 12.00 | 479.43 | 271.75 | 88.49 | 1787.58 | 681.71 | 28.68 | 216.23 | 260.78 | 193.45 | 274.27 | 29.47 | 478.69 | 1699.04 | 138.24 | 29.05 | 19.63 | 1.24 | 10.87 | 1.02 | 57.13 | 0.00 | 27.24 | 35.19 | 44.36 | 705.53 | 9738.71 | 5191.44 | 7463.06 | 2.10 | 98.66 | 134.68 | 1.61 | 1.77 | 5.16 | 15.77 | 2.35 | 6.28 | 1243.95 | 611.70 | 0.56 | 5.21 | 501.27 | 39.25 | 690.87 | 283.55 |
| 213482 | NorCOMM2 | Mfap4 | Mfap4_tm1b_C11 | -/- | F | 213482 | 1/20/2015 | 234.81 | 638.08 | 157.71 | 39.61 | 6.30 | 10.14 | 7.50 | 296.87 | 150.16 | 70.77 | 1742.03 | 419.93 | 26.35 | 244.54 | 240.60 | 171.35 | 224.21 | 25.97 | 558.77 | 1161.65 | 94.00 | 33.19 | 14.34 | 1.03 | 12.81 | 1.47 | 53.68 | 0.00 | 33.81 | 37.35 | 41.49 | 820.15 | 8898.92 | 5643.89 | 6235.97 | 3.03 | 65.37 | 130.70 | 2.77 | 2.50 | 3.51 | 13.81 | 3.27 | 5.71 | 1180.10 | 752.45 | 0.87 | 7.14 | 342.05 | 73.97 | 1676.41 | 696.00 |
| 193352 | NorCOMM2 | Mfap4 | Mfap4_tm1b_C11 | -/- | M | 193352 | 12/11/2014 | 239.10 | 771.79 | 127.36 | 27.35 | 4.63 | 14.60 | 12.68 | 466.87 | 279.61 | 120.08 | 1150.52 | 851.55 | 73.04 | 721.98 | 866.83 | 813.99 | 1357.23 | 31.21 | 415.73 | 564.83 | 113.08 | 36.49 | 17.10 | 1.29 | 12.71 | 1.24 | 71.57 | 0.00 | 32.92 | 34.69 | 37.57 | 906.45 | 12113.05 | 7312.25 | 7891.49 | 1.93 | 242.57 | 154.03 | 3.02 | 2.48 | 5.79 | 33.00 | 3.39 | 5.16 | 1119.65 | 736.31 | 1.73 | 0.75 | 657.51 | 3658.53 | 3843.02 | 1614.68 |
| 193349 | NorCOMM2 | Mfap4 | Mfap4_tm1b_C11 | -/- | M | 193349 | 12/11/2014 | 293.20 | 744.65 | 110.15 | 44.20 | 6.58 | 8.10 | 16.54 | 390.21 | 257.21 | 93.00 | 1230.08 | 723.53 | 67.11 | 885.51 | 859.69 | 611.62 | 1133.69 | 31.17 | 520.68 | 495.48 | 116.33 | 35.20 | 20.02 | 1.37 | 12.72 | 1.28 | 66.16 | 3.31 | 42.77 | 25.40 | 35.88 | 800.40 | 5876.18 | 5099.11 | 5830.74 | 1.37 | 69.59 | 125.13 | 7.70 | 1.64 | 5.72 | 26.22 | 3.75 | 4.75 | 1010.69 | 611.70 | 1.70 | 1.26 | 714.80 | 2621.39 | 7253.28 | 2721.37 |
| 193353 | NorCOMM2 | Mfap4 | Mfap4_tm1b_C11 | -/- | M | 193353 | 12/11/2014 | 262.16 | 774.82 | 133.91 | 30.25 | 4.79 | 11.08 | 13.92 | 260.47 | 164.48 | 101.64 | 886.43 | 751.72 | 63.23 | 449.30 | 491.66 | 403.48 | 822.28 | 23.77 | 454.48 | 831.43 | 116.53 | 27.86 | 31.25 | 1.25 | 11.14 | 1.37 | 72.68 | 2.02 | 40.09 | 26.93 | 41.33 | 841.91 | 7747.13 | 5305.05 | 6071.54 | 1.12 | 129.78 | 130.62 | 5.36 | 1.95 | 6.38 | 20.15 | 3.23 | 4.90 | 1135.18 | 658.18 | 1.73 | 1.06 | 692.71 | 777.25 | 3284.86 | 1136.71 |
| 356364 | NorCOMM2 | Mmachc | Mmachc_tm1.1_A10 | +/- | F | 356364 | 11/17/2015 | 216.60 | 668.82 | 155.77 | 26.17 | 4.26 | 6.82 | 8.67 | 298.09 | 196.73 | 85.90 | 1717.09 | 473.04 | 23.10 | 151.02 | 173.29 | 166.23 | 214.32 | 25.23 | 557.32 | 1337.19 | 88.93 | 24.90 | 15.35 | 1.44 | 10.27 | 1.01 | 57.27 | 0.00 | 21.62 | 34.61 | 41.26 | 641.96 | 6005.31 | 4037.19 | 5327.15 | 1.47 | 58.64 | 112.62 | 6.03 | 1.75 | 4.22 | 11.01 | 1.86 | 5.25 | 1153.70 | 476.73 | 0.99 | 4.70 | 414.94 | 22.33 | 3882.02 | 1635.22 |
| 356363 | NorCOMM2 | Mmachc | Mmachc_tm1.1_A10 | +/- | F | 356363 | 11/17/2015 | 242.00 | 737.45 | 195.45 | 51.33 | 8.16 | 8.38 | 12.90 | 369.12 | 313.86 | 115.77 | 1858.44 | 591.17 | 29.85 | 168.06 | 199.32 | 144.09 | 254.53 | 20.56 | 517.71 | 1565.95 | 108.42 | 20.90 | 15.99 | 1.04 | 10.71 | 0.98 | 84.57 | 2.99 | 23.81 | 42.87 | 43.19 | 792.02 | 7477.41 | 5709.88 | 5516.31 | 2.09 | 229.65 | 131.11 | 3.58 | 2.17 | 3.79 | 14.29 | 0.00 | 5.70 | 1548.04 | 501.29 | 0.87 | 5.64 | 449.20 | 55.98 | 1772.65 | 662.86 |
| 356370 | NorCOMM2 | Mmachc | Mmachc_tm1.1_A10 | +/- | F | 356370 | 11/19/2015 | 263.29 | 655.69 | 165.34 | 43.25 | 3.57 | 9.08 | 15.98 | 407.80 | 280.31 | 108.90 | 1498.25 | 543.52 | 26.74 | 138.64 | 157.45 | 144.60 | 280.50 | 24.60 | 527.49 | 1580.96 | 114.28 | 29.44 | 22.54 | 0.75 | 12.78 | 1.07 | 83.14 | 0.00 | 20.95 | 43.03 | 37.13 | 677.98 | 8230.33 | 4942.81 | 7022.48 | 1.88 | 256.44 | 120.16 | 3.53 | 1.44 | 2.54 | 12.53 | 2.22 | 6.28 | 1326.86 | 509.19 | 0.92 | 6.72 | 492.95 | 33.82 | 1570.25 | 704.30 |
| 356155 | NorCOMM2 | Mmachc | Mmachc_tm1.1_A10 | +/- | M | 356155 | 11/17/2015 | 284.62 | 789.28 | 121.03 | 23.53 | 9.13 | 6.03 | 14.40 | 578.86 | 396.47 | 135.33 | 1197.48 | 1119.94 | 76.04 | 934.84 | 834.27 | 694.80 | 2246.12 | 32.03 | 569.17 | 422.35 | 117.60 | 28.75 | 11.75 | 1.45 | 13.48 | 1.29 | 70.78 | 3.75 | 65.58 | 42.97 | 27.63 | 1036.95 | 10727.08 | 9136.60 | 8584.68 | 4.07 | 288.76 | 149.40 | 2.86 | 2.91 | 9.76 | 34.54 | 3.94 | 5.58 | 1190.14 | 551.17 | 1.32 | 1.37 | 802.26 | 14980.55 | 2821.21 | 947.50 |
| 356156 | NorCOMM2 | Mmachc | Mmachc_tm1.1_A10 | +/- | M | 356156 | 11/17/2015 | 254.78 | 711.21 | 123.26 | 33.89 | 4.95 | 8.84 | 13.13 | 497.14 | 376.13 | 121.81 | 1095.24 | 1030.17 | 92.03 | 646.26 | 803.70 | 719.84 | 1564.81 | 25.75 | 628.99 | 462.23 | 107.08 | 36.60 | 22.78 | 1.06 | 13.80 | 1.11 | 66.14 | 3.31 | 47.82 | 34.01 | 26.48 | 887.91 | 13450.65 | 10333.63 | 12640.65 | 4.41 | 202.25 | 162.04 | 2.28 | 3.39 | 3.71 | 37.75 | 2.48 | 5.74 | 1237.95 | 615.29 | 0.72 | 1.14 | 685.35 | 11528.56 | 1086.04 | 513.70 |
| 356153 | NorCOMM2 | Mmachc | Mmachc_tm1.1_A10 | +/- | M | 356153 | 11/17/2015 | 320.01 | 783.39 | 145.66 | 29.33 | 4.50 | 8.99 | 20.13 | 700.67 | 367.88 | 125.58 | 1203.15 | 970.58 | 71.92 | 693.93 | 1205.63 | 598.75 | 1309.76 | 31.42 | 490.34 | 497.19 | 95.58 | 37.23 | 16.20 | 1.73 | 13.17 | 0.99 | 105.67 | 2.37 | 59.37 | 44.01 | 35.97 | 861.76 | 10342.31 | 7796.69 | 9785.29 | 1.98 | 249.02 | 149.84 | 4.94 | 2.18 | 4.98 | 24.40 | 0.00 | 5.60 | 1249.85 | 539.92 | 1.25 | 1.13 | 756.75 | 3716.64 | 2029.24 | 864.80 |
| 328106 | NorCOMM2 | Mvk | Mvk_em1_del | +/- | F | 328106 | 10/1/2015 | 222.16 | 807.75 | 142.56 | 40.71 | 3.96 | 8.49 | 9.99 | 274.10 | 196.59 | 62.61 | 1627.00 | 442.25 | 32.91 | 131.56 | 189.39 | 137.70 | 209.16 | 26.19 | 462.11 | 1345.90 | 95.59 | 31.38 | 19.86 | 0.81 | 13.18 | 0.80 | 76.88 | 1.05 | 17.85 | 30.82 | 43.41 | 747.50 | 6950.50 | 4935.30 | 5324.50 | 2.25 | 124.22 | 110.05 | 2.08 | 2.21 | 3.97 | 17.57 | 3.13 | 5.01 | 1254.50 | 627.14 | 0.75 | 4.75 | 381.65 | 30.41 | 1381.40 | 677.05 |
| 328100 | NorCOMM2 | Mvk | Mvk_em1_del | +/- | F | 328100 | 10/1/2015 | 217.40 | 709.91 | 172.29 | 29.95 | 3.23 | 9.92 | 8.33 | 324.65 | 157.69 | 68.06 | 1498.30 | 427.17 | 31.10 | 145.75 | 232.73 | 189.58 | 226.30 | 23.54 | 513.29 | 1265.10 | 95.62 | 32.85 | 14.81 | 1.04 | 9.40 | 0.98 | 72.82 | 0.00 | 24.38 | 36.58 | 39.51 | 733.94 | 5936.00 | 3829.50 | 4416.70 | 2.08 | 175.85 | 110.49 | 3.34 | 2.13 | 0.00 | 14.10 | 0.00 | 5.57 | 1044.10 | 652.51 | 1.07 | 5.64 | 365.52 | 20.20 | 2367.30 | 741.69 |
| 328194 | NorCOMM2 | Mvk | Mvk_em1_del | +/- | F | 328194 | 10/1/2015 | 230.17 | 784.43 | 146.80 | 47.82 | 5.56 | 6.94 | 13.61 | 332.71 | 185.15 | 88.12 | 1774.30 | 509.24 | 30.51 | 133.09 | 172.01 | 146.17 | 197.43 | 26.57 | 444.93 | 1473.00 | 98.32 | 39.11 | 15.22 | 0.92 | 13.15 | 1.03 | 64.23 | 0.00 | 15.14 | 37.90 | 32.68 | 816.75 | 9333.80 | 4789.60 | 6495.60 | 2.20 | 157.42 | 106.62 | 2.30 | 2.02 | 0.00 | 12.52 | 2.64 | 5.84 | 1090.80 | 646.80 | 1.49 | 5.69 | 463.72 | 77.81 | 1121.40 | 302.44 |
| 328004 | NorCOMM2 | Mvk | Mvk_em1_del | +/- | M | 328004 | 9/29/2015 | 215.87 | 932.50 | 91.15 | 34.52 | 3.43 | 13.65 | 20.79 | 449.44 | 296.52 | 123.65 | 1125.00 | 746.78 | 78.96 | 764.29 | 829.74 | 744.75 | 1763.20 | 29.22 | 517.73 | 531.91 | 93.59 | 36.55 | 16.87 | 1.22 | 13.26 | 1.03 | 66.90 | 1.92 | 39.62 | 35.12 | 32.15 | 798.23 | 11363.00 | 9041.20 | 9329.90 | 2.17 | 171.50 | 124.76 | 2.23 | 2.28 | 5.11 | 31.74 | 3.25 | 7.12 | 1172.60 | 965.39 | 1.42 | 1.33 | 952.37 | 6697.40 | 1138.70 | 404.29 |
| 328098 | NorCOMM2 | Mvk | Mvk_em1_del | +/- | M | 328098 | 9/29/2015 | 240.05 | 1085.80 | 81.53 | 23.32 | 7.33 | 8.29 | 9.89 | 595.22 | 395.02 | 122.73 | 1241.40 | 738.37 | 73.07 | 567.77 | 845.88 | 594.01 | 1773.90 | 36.41 | 526.37 | 672.03 | 104.91 | 49.95 | 13.69 | 1.13 | 12.52 | 1.21 | 59.02 | 6.41 | 65.94 | 26.54 | 37.90 | 870.53 | 9661.30 | 7348.20 | 7737.80 | 3.49 | 270.47 | 115.39 | 7.10 | 2.29 | 8.57 | 22.01 | 4.75 | 6.11 | 900.86 | 689.47 | 18.05 | 1.44 | 861.37 | 11477.00 | 2466.30 | 1209.30 |
| 345318 | NorCOMM2 | Mvk | Mvk_em1_del | +/- | M | 345318 | 10/27/2015 | 261.96 | 1046.60 | 149.17 | 40.44 | 4.47 | 8.12 | 18.14 | 267.73 | 209.72 | 87.10 | 1060.90 | 698.68 | 52.45 | 351.80 | 471.74 | 385.36 | 573.27 | 28.27 | 409.49 | 911.43 | 113.70 | 30.31 | 22.02 | 0.93 | 12.34 | 1.07 | 60.17 | 1.43 | 30.99 | 32.20 | 34.68 | 864.87 | 9867.40 | 4964.30 | 7812.80 | 1.14 | 110.29 | 111.49 | 3.84 | 1.30 | 4.67 | 15.60 | 2.96 | 3.71 | 1111.30 | 709.24 | 1.32 | 1.12 | 708.36 | 343.90 | 3040.30 | 1170.00 |
| 324602 | NorCOMM2 | Nek2 | Nek2_em1_nhej | -/- | F | 324602 | 9/22/2015 | 268.32 | 842.51 | 194.37 | 43.61 | 7.24 | 8.77 | 15.87 | 399.79 | 356.08 | 95.75 | 1927.50 | 629.16 | 28.70 | 137.37 | 173.74 | 118.82 | 231.53 | 27.95 | 435.68 | 1809.00 | 126.60 | 35.25 | 15.56 | 1.38 | 14.22 | 1.34 | 80.16 | 2.68 | 19.25 | 44.58 | 45.51 | 828.42 | 7719.90 | 4983.60 | 5520.20 | 1.87 | 127.43 | 112.98 | 1.78 | 1.94 | 4.51 | 20.63 | 2.81 | 5.05 | 1245.60 | 479.30 | 0.52 | 6.33 | 511.34 | 83.15 | 895.76 | 297.62 |
| 324611 | NorCOMM2 | Nek2 | Nek2_em1_nhej | -/- | F | 324611 | 9/22/2015 | 181.77 | 726.00 | 149.49 | 41.28 | 5.12 | 9.06 | 17.78 | 329.97 | 241.86 | 97.69 | 1567.30 | 465.61 | 30.48 | 129.34 | 150.64 | 128.15 | 223.82 | 22.13 | 390.63 | 1443.80 | 72.00 | 33.66 | 16.48 | 1.01 | 13.35 | 1.12 | 54.36 | 0.00 | 16.76 | 44.63 | 42.08 | 731.31 | 8132.10 | 5670.30 | 5263.40 | 2.08 | 211.01 | 98.66 | 2.79 | 2.03 | 3.34 | 29.82 | 0.00 | 7.23 | 1139.30 | 700.18 | 1.01 | 5.69 | 321.16 | 201.50 | 2131.80 | 626.34 |
| 324604 | NorCOMM2 | Nek2 | Nek2_em1_nhej | -/- | F | 324604 | 9/22/2015 | 210.68 | 735.25 | 98.48 | 32.40 | 4.34 | 6.67 | 13.53 | 491.87 | 349.59 | 90.78 | 1441.00 | 576.30 | 27.81 | 119.37 | 114.10 | 114.09 | 211.17 | 29.78 | 454.46 | 1445.10 | 89.50 | 43.88 | 14.21 | 1.13 | 13.17 | 1.13 | 58.39 | 0.00 | 13.79 | 24.37 | 47.74 | 669.37 | 4479.90 | 2929.20 | 2676.00 | 1.35 | 31.44 | 132.44 | 6.07 | 2.58 | 3.74 | 20.46 | 0.00 | 5.92 | 1344.00 | 439.26 | 1.54 | 8.46 | 501.21 | 93.62 | 5917.80 | 2215.70 |
| 363815 | NorCOMM2 | Nek2 | Nek2_em1_nhej | -/- | M | 363815 | 12/10/2015 | 233.63 | 807.15 | 101.67 | 45.56 | 3.00 | 7.73 | 23.51 | 552.47 | 270.44 | 140.92 | 973.51 | 610.08 | 68.47 | 636.78 | 703.06 | 541.40 | 1281.80 | 23.03 | 603.97 | 495.97 | 94.38 | 46.28 | 12.97 | 0.72 | 14.14 | 1.08 | 63.40 | 1.53 | 55.57 | 37.49 | 25.19 | 897.28 | 9189.20 | 7791.60 | 7635.30 | 1.31 | 107.44 | 150.71 | 2.70 | 1.76 | 7.44 | 20.25 | 3.19 | 4.52 | 1105.10 | 625.57 | 1.07 | 0.51 | 794.63 | 4710.80 | 2224.60 | 873.54 |
| 366625 | NorCOMM2 | Nek2 | Nek2_em1_nhej | -/- | M | 366625 | 12/17/2015 | 201.96 | 869.52 | 90.17 | 31.42 | 5.18 | 4.82 | 12.22 | 175.54 | 177.68 | 87.78 | 1161.60 | 523.83 | 60.01 | 343.74 | 439.99 | 343.37 | 702.02 | 19.53 | 410.79 | 731.41 | 99.65 | 20.62 | 17.98 | 0.94 | 9.70 | 0.87 | 70.93 | 1.84 | 41.75 | 28.01 | 30.35 | 591.32 | 8762.30 | 6167.10 | 7339.90 | 1.32 | 195.65 | 112.55 | 3.65 | 1.29 | 5.30 | 13.63 | 3.25 | 3.73 | 766.82 | 472.76 | 1.43 | 0.36 | 715.58 | 25.68 | 2787.90 | 881.10 |
| 363822 | NorCOMM2 | Nek2 | Nek2_em1_nhej | -/- | M | 363822 | 12/10/2015 | 198.34 | 913.18 | 82.34 | 31.11 | 3.51 | 10.69 | 25.09 | 535.42 | 321.80 | 100.68 | 1183.10 | 896.16 | 99.80 | 619.33 | 806.82 | 614.11 | 1292.10 | 28.35 | 511.12 | 670.46 | 122.88 | 31.77 | 15.62 | 0.80 | 12.62 | 1.00 | 69.52 | 0.00 | 51.73 | 35.99 | 30.40 | 961.96 | 10731.00 | 7669.80 | 6514.20 | 1.83 | 210.56 | 127.41 | 2.62 | 2.24 | 6.32 | 21.20 | 2.53 | 5.87 | 963.00 | 611.16 | 1.61 | 1.14 | 735.57 | 6354.70 | 1586.40 | 764.86 |
| 75974 | NorCOMM2 | Npc2 | Npc2_tm1e.1_A06 | +/- | F | 75974 | 4/1/2014 | 190.10 | 703.41 | 132.61 | 36.30 | 35.82 | 9.02 | 16.59 | 238.76 | 214.11 | 75.18 | 1788.80 | 436.99 | 26.05 | 97.10 | 171.30 | 141.67 | 237.95 | 21.64 | 451.56 | 1758.90 | 101.36 | 35.88 | 14.49 | 1.11 | 11.02 | 1.04 | 88.30 | 10.66 | 15.49 | 38.15 | 48.32 | 681.56 | 7379.00 | 4494.80 | 4623.70 | 1.70 | 146.28 | 97.86 | 11.45 | 1.63 | 5.25 | 38.17 | 2.15 | 8.42 | 1156.50 | 596.03 | 1.67 | 6.79 | 473.11 | 23.57 | 8957.20 | 3192.00 |
| 75975 | NorCOMM2 | Npc2 | Npc2_tm1e.1_A06 | +/- | F | 75975 | 4/1/2014 | 183.54 | 791.56 | 110.12 | 48.19 | 45.74 | 7.07 | 12.78 | 287.43 | 148.42 | 63.73 | 1784.90 | 401.12 | 31.31 | 156.47 | 165.99 | 143.04 | 228.80 | 23.67 | 486.41 | 1250.50 | 96.55 | 45.95 | 19.34 | 0.96 | 10.94 | 1.05 | 56.02 | 14.74 | 20.48 | 34.07 | 36.19 | 701.34 | 6790.10 | 4492.00 | 4478.70 | 1.40 | 64.77 | 84.38 | 5.73 | 1.94 | 3.94 | 34.43 | 0.00 | 6.55 | 1106.80 | 497.81 | 2.21 | 8.07 | 486.58 | 23.44 | 4822.10 | 1714.10 |
| 75976 | NorCOMM2 | Npc2 | Npc2_tm1e.1_A06 | +/- | F | 75976 | 4/1/2014 | 212.49 | 835.06 | 81.33 | 49.86 | 8.47 | 5.27 | 10.21 | 250.86 | 112.06 | 82.95 | 1659.80 | 526.94 | 34.75 | 129.33 | 187.60 | 130.95 | 229.03 | 28.73 | 359.52 | 1491.50 | 96.81 | 36.83 | 17.93 | 1.22 | 11.23 | 1.29 | 77.15 | 5.61 | 15.87 | 20.40 | 45.05 | 862.76 | 1875.50 | 1593.50 | 1596.40 | 1.08 | 20.49 | 87.36 | 12.50 | 2.00 | 4.53 | 21.58 | 0.00 | 6.47 | 1164.80 | 650.70 | 2.50 | 9.96 | 477.50 | 20.12 | 12335.00 | 4710.60 |
| 92278 | NorCOMM2 | Npc2 | Npc2_tm1e.1_A06 | +/- | M | 92278 | 4/29/2014 | 248.30 | 977.14 | 132.46 | 51.56 | 9.70 | 6.33 | 29.50 | 616.80 | 256.80 | 107.86 | 1601.40 | 733.81 | 64.88 | 562.46 | 649.78 | 559.53 | 895.09 | 27.43 | 438.06 | 779.09 | 118.27 | 36.99 | 23.19 | 1.41 | 10.84 | 1.13 | 67.86 | 10.49 | 41.84 | 36.15 | 31.16 | 1045.50 | 6356.10 | 6444.10 | 7191.00 | 0.89 | 41.09 | 114.59 | 15.23 | 1.45 | 4.08 | 30.61 | 3.61 | 4.90 | 1269.50 | 628.74 | 4.12 | 0.49 | 747.93 | 228.22 | 12909.00 | 4269.20 |
| 92277 | NorCOMM2 | Npc2 | Npc2_tm1e.1_A06 | +/- | M | 92277 | 4/29/2014 | 273.15 | 1015.70 | 83.32 | 34.29 | 7.60 | 10.20 | 41.86 | 917.25 | 530.15 | 142.98 | 1449.00 | 1007.30 | 118.00 | 714.41 | 800.71 | 854.57 | 1273.40 | 53.31 | 446.29 | 497.68 | 125.73 | 36.92 | 16.79 | 1.17 | 14.52 | 1.40 | 67.74 | 7.77 | 73.65 | 31.30 | 33.65 | 1059.70 | 10682.00 | 9155.40 | 10193.00 | 5.65 | 44.86 | 135.15 | 12.82 | 4.23 | 2.68 | 37.00 | 0.00 | 7.32 | 1264.70 | 612.59 | 1.77 | 0.82 | 742.02 | 18022.00 | 12962.00 | 5020.60 |
| 92287 | NorCOMM2 | Npc2 | Npc2_tm1e.1_A06 | +/- | M | 92287 | 4/29/2014 | 208.21 | 853.25 | 83.25 | 33.89 | 4.45 | 11.17 | 12.64 | 440.56 | 223.51 | 114.20 | 1283.10 | 921.01 | 95.36 | 566.71 | 746.78 | 591.04 | 1352.90 | 42.89 | 536.58 | 512.49 | 124.08 | 47.67 | 19.38 | 1.43 | 13.67 | 1.19 | 75.43 | 3.47 | 82.76 | 37.78 | 24.89 | 1083.00 | 16650.00 | 11410.00 | 12172.00 | 5.18 | 113.42 | 109.73 | 2.96 | 4.51 | 3.76 | 21.78 | 2.49 | 5.53 | 1034.20 | 708.21 | 0.71 | 0.83 | 693.53 | 25046.00 | 1504.90 | 600.01 |
| 417059 | KOMP2 | Pebp1 | Pebp1_em1_del | -/- | F | 417059 | 7/20/2016 | 211.81 | 692.31 | 150.00 | 37.46 | 4.99 | 12.07 | 7.86 | 570.08 | 470.39 | 111.13 | 1789.50 | 601.32 | 24.66 | 205.88 | 238.45 | 227.36 | 291.56 | 33.97 | 452.48 | 1103.18 | 111.32 | 44.93 | 11.35 | 0.99 | 9.34 | 1.54 | 68.85 | 0.00 | 17.87 | 51.39 | 57.27 | 788.38 | 9869.13 | 6831.04 | 8052.92 | 2.24 | 369.73 | 146.13 | 1.41 | 1.42 | 0.00 | 15.75 | 1.81 | 4.81 | 1195.72 | 617.11 | 0.00 | 7.16 | 358.71 | 104.91 | 535.50 | 233.98 |
| 428681 | KOMP2 | Pebp1 | Pebp1_em1_del | -/- | F | 428681 | 8/23/2016 | 235.05 | 685.89 | 206.99 | 46.87 | 9.26 | 5.79 | 10.30 | 323.42 | 215.68 | 76.73 | 1801.37 | 446.41 | 25.26 | 118.49 | 137.61 | 109.05 | 225.23 | 24.39 | 494.99 | 1861.80 | 117.60 | 42.15 | 12.26 | 1.14 | 9.75 | 0.88 | 69.09 | 0.00 | 15.39 | 45.73 | 54.16 | 683.35 | 7993.02 | 6536.32 | 7006.49 | 1.92 | 257.53 | 147.25 | 2.69 | 1.64 | 4.82 | 15.02 | 1.80 | 5.50 | 957.02 | 565.00 | 0.00 | 7.30 | 350.41 | 58.41 | 2082.02 | 851.04 |
| 417051 | KOMP2 | Pebp1 | Pebp1_em1_del | -/- | F | 417051 | 7/13/2016 | 196.08 | 529.67 | 145.75 | 22.80 | 4.64 | 4.52 | 8.87 | 496.93 | 251.25 | 91.93 | 1585.36 | 528.66 | 21.40 | 160.45 | 216.65 | 147.56 | 296.94 | 22.11 | 463.22 | 1370.90 | 84.25 | 28.68 | 9.76 | 1.03 | 9.11 | 0.88 | 74.77 | 0.00 | 9.84 | 55.68 | 57.34 | 647.01 | 9366.42 | 6428.99 | 7159.80 | 2.14 | 365.54 | 122.02 | 2.31 | 2.07 | 3.49 | 23.80 | 1.19 | 5.74 | 1281.05 | 586.78 | 1.04 | 7.99 | 291.54 | 23.49 | 1184.70 | 521.99 |
| 429694 | KOMP2 | Pebp1 | Pebp1_em1_del | -/- | M | 429694 | 8/23/2016 | 262.79 | 711.18 | 124.97 | 40.43 | 7.15 | 10.54 | 12.27 | 521.56 | 445.42 | 133.88 | 1178.35 | 740.75 | 73.34 | 744.91 | 813.49 | 763.83 | 1203.73 | 36.94 | 517.69 | 643.69 | 115.24 | 26.39 | 13.89 | 1.07 | 10.51 | 1.01 | 68.55 | 0.00 | 42.30 | 41.99 | 45.83 | 747.88 | 16142.97 | 11304.34 | 10471.43 | 4.01 | 352.64 | 170.08 | 2.19 | 3.32 | 5.73 | 31.18 | 2.37 | 4.33 | 975.02 | 690.05 | 0.00 | 0.70 | 625.05 | 14536.29 | 1423.05 | 662.59 |
| 419964 | KOMP2 | Pebp1 | Pebp1_em1_del | -/- | M | 419964 | 7/28/2016 | 287.99 | 815.80 | 159.88 | 34.52 | 4.56 | 9.23 | 10.09 | 488.42 | 330.21 | 116.53 | 1190.42 | 635.80 | 52.20 | 460.03 | 557.18 | 441.00 | 923.13 | 34.68 | 528.00 | 918.40 | 132.39 | 27.35 | 9.50 | 1.08 | 12.51 | 1.04 | 63.47 | 0.00 | 28.56 | 34.05 | 51.50 | 932.15 | 10943.42 | 8458.49 | 8961.64 | 1.62 | 226.25 | 120.88 | 2.20 | 2.19 | 6.40 | 19.12 | 2.13 | 4.25 | 1130.43 | 646.87 | 0.00 | 0.00 | 758.10 | 359.08 | 1778.72 | 796.72 |
| 435823 | KOMP2 | Pebp1 | Pebp1_em1_del | -/- | M | 435823 | 9/8/2016 | 280.15 | 826.40 | 128.70 | 31.22 | 2.85 | 8.84 | 10.26 | 348.01 | 202.33 | 119.13 | 1292.05 | 759.85 | 55.92 | 390.19 | 460.09 | 339.97 | 848.84 | 28.23 | 405.86 | 893.71 | 132.72 | 33.71 | 10.86 | 0.85 | 11.65 | 1.08 | 66.61 | 0.00 | 27.55 | 31.86 | 50.56 | 807.55 | 11432.76 | 8790.73 | 8891.01 | 1.74 | 197.72 | 110.62 | 0.00 | 1.48 | 5.67 | 18.07 | 2.02 | 4.48 | 966.23 | 775.93 | 0.00 | 0.81 | 761.15 | 385.78 | 660.68 | 267.73 |
| 244286 | NorCOMM2 | Phyh | Phyh_tm1b_G08 | -/- | F | 244286 | 5/1/2015 | 196.28 | 640.72 | 117.83 | 34.83 | 4.37 | 9.29 | 7.46 | 236.45 | 175.89 | 70.45 | 1085.98 | 421.38 | 19.19 | 143.20 | 134.14 | 109.88 | 196.94 | 22.99 | 400.86 | 1158.69 | 97.98 | 29.91 | 13.84 | 0.99 | 9.29 | 1.35 | 57.22 | 1.85 | 12.21 | 40.08 | 37.80 | 574.61 | 7322.09 | 5537.44 | 4983.50 | 1.14 | 96.54 | 120.42 | 3.34 | 1.66 | 4.27 | 12.17 | 0.00 | 4.66 | 1241.06 | 555.44 | 0.99 | 6.31 | 432.08 | 64.01 | 3347.76 | 1026.43 |
| 237159 | NorCOMM2 | Phyh | Phyh_tm1b_G08 | -/- | F | 237159 | 4/21/2015 | 198.15 | 577.05 | 131.10 | 33.87 | 3.77 | 7.83 | 10.57 | 303.80 | 209.36 | 79.47 | 1161.76 | 426.02 | 24.15 | 167.99 | 187.14 | 141.19 | 222.08 | 28.16 | 462.64 | 1349.14 | 119.27 | 23.23 | 17.78 | 1.22 | 9.59 | 0.46 | 44.68 | 0.00 | 16.17 | 23.92 | 32.41 | 601.51 | 5480.16 | 4410.03 | 5078.03 | 1.62 | 81.79 | 92.32 | 17.30 | 1.34 | 4.59 | 15.47 | 2.70 | 5.05 | 790.74 | 663.55 | 1.15 | 3.57 | 305.46 | 10.78 | 17164.28 | 6240.26 |
| 237165 | NorCOMM2 | Phyh | Phyh_tm1b_G08 | -/- | F | 237165 | 4/21/2015 | 183.74 | 616.07 | 164.21 | 36.01 | 14.34 | 7.57 | 9.46 | 319.30 | 214.46 | 75.19 | 1467.06 | 505.66 | 22.21 | 133.72 | 131.83 | 119.81 | 189.87 | 21.82 | 401.74 | 1373.23 | 106.59 | 25.90 | 12.79 | 1.15 | 11.41 | 0.88 | 48.99 | 4.73 | 19.97 | 17.93 | 51.44 | 717.87 | 6297.02 | 3988.35 | 4720.50 | 1.96 | 71.53 | 84.24 | 14.62 | 1.74 | 3.39 | 17.64 | 2.58 | 4.90 | 1226.36 | 623.19 | 1.14 | 4.53 | 396.42 | 11.45 | 14750.30 | 5679.24 |
| 237056 | NorCOMM2 | Phyh | Phyh_tm1b_G08 | -/- | M | 237056 | 4/21/2015 | 228.17 | 802.75 | 88.37 | 32.82 | 6.37 | 11.96 | 12.20 | 455.42 | 362.47 | 95.18 | 1047.15 | 742.24 | 78.45 | 616.29 | 819.53 | 695.45 | 1542.26 | 35.07 | 532.74 | 550.31 | 144.85 | 41.98 | 17.41 | 0.89 | 11.95 | 0.95 | 59.08 | 0.00 | 66.70 | 38.42 | 30.04 | 727.11 | 12506.34 | 8399.19 | 8365.32 | 2.88 | 332.98 | 130.77 | 2.04 | 1.63 | 4.93 | 17.35 | 2.37 | 5.27 | 1003.78 | 683.88 | 1.33 | 0.86 | 975.83 | 10885.69 | 1765.96 | 608.23 |
| 244177 | NorCOMM2 | Phyh | Phyh_tm1b_G08 | -/- | M | 244177 | 5/1/2015 | 254.68 | 866.60 | 142.03 | 27.00 | 5.91 | 12.97 | 8.64 | 409.82 | 294.58 | 81.78 | 786.13 | 690.33 | 50.78 | 509.19 | 578.48 | 482.07 | 727.37 | 25.92 | 523.87 | 730.63 | 115.47 | 28.60 | 11.57 | 1.31 | 10.07 | 1.18 | 83.57 | 1.57 | 38.70 | 33.28 | 43.07 | 830.07 | 12378.04 | 7158.06 | 9259.05 | 1.38 | 166.40 | 155.85 | 2.93 | 1.88 | 8.02 | 17.00 | 2.01 | 5.43 | 908.34 | 760.12 | 0.54 | 0.77 | 838.70 | 628.80 | 2104.51 | 753.89 |
| 237057 | NorCOMM2 | Phyh | Phyh_tm1b_G08 | -/- | M | 237057 | 4/21/2015 | 220.69 | 660.74 | 107.59 | 27.48 | 4.84 | 10.05 | 11.00 | 330.61 | 199.02 | 109.83 | 875.33 | 528.16 | 61.95 | 701.02 | 831.17 | 645.63 | 1172.20 | 24.64 | 445.19 | 486.40 | 135.24 | 36.74 | 14.64 | 1.21 | 13.09 | 1.05 | 78.40 | 0.00 | 62.69 | 26.15 | 23.66 | 735.84 | 6290.39 | 5590.01 | 6511.13 | 1.93 | 99.33 | 130.09 | 7.14 | 1.27 | 4.48 | 15.49 | 3.27 | 4.23 | 914.20 | 618.81 | 1.07 | 0.77 | 704.48 | 2802.22 | 8268.21 | 2904.33 |
| 170510 | NorCOMM2 | Pipox | Pipox_tm1b_A08 | -/- | F | 170510 | 10/9/2014 | 193.55 | 766.96 | 146.83 | 44.95 | 6.74 | 6.19 | 22.82 | 422.44 | 291.82 | 92.16 | 1371.30 | 611.98 | 24.28 | 158.68 | 226.83 | 137.65 | 255.19 | 23.89 | 372.08 | 1380.10 | 79.13 | 33.76 | 13.08 | 1.09 | 10.75 | 0.94 | 77.94 | 3.31 | 18.86 | 35.30 | 40.45 | 796.56 | 5212.40 | 3492.60 | 4162.50 | 1.77 | 35.84 | 99.91 | 4.68 | 1.72 | 4.77 | 13.53 | 2.44 | 6.07 | 1372.30 | 655.26 | 1.70 | 10.33 | 510.92 | 38.33 | 5201.30 | 1845.20 |
| 170501 | NorCOMM2 | Pipox | Pipox_tm1b_A08 | -/- | F | 170501 | 10/9/2014 | 217.54 | 712.95 | 143.58 | 50.73 | 11.07 | 5.25 | 14.11 | 272.13 | 175.67 | 83.30 | 1525.70 | 349.29 | 21.22 | 94.72 | 185.83 | 103.04 | 162.18 | 26.79 | 467.16 | 1617.80 | 77.55 | 41.82 | 14.31 | 0.94 | 9.53 | 0.79 | 55.05 | 4.54 | 16.34 | 35.25 | 30.22 | 852.48 | 6955.60 | 4484.50 | 5366.30 | 1.62 | 99.40 | 129.32 | 3.28 | 1.95 | 0.00 | 14.10 | 0.00 | 5.32 | 1347.60 | 557.82 | 1.43 | 8.30 | 330.22 | 22.52 | 3022.10 | 1133.40 |
| 170500 | NorCOMM2 | Pipox | Pipox_tm1b_A08 | -/- | F | 170500 | 10/9/2014 | 211.64 | 683.83 | 153.02 | 46.76 | 7.84 | 6.38 | 20.26 | 408.56 | 292.90 | 100.12 | 1908.90 | 602.81 | 28.46 | 122.22 | 150.72 | 109.44 | 238.29 | 25.81 | 387.55 | 1883.50 | 79.83 | 36.12 | 13.55 | 1.42 | 10.92 | 1.23 | 98.08 | 3.46 | 17.11 | 34.43 | 37.86 | 727.68 | 6250.50 | 4969.40 | 5637.20 | 1.83 | 96.63 | 137.65 | 4.90 | 2.19 | 4.26 | 14.59 | 2.61 | 6.02 | 1420.20 | 530.46 | 0.74 | 8.85 | 421.21 | 24.85 | 4090.00 | 1428.60 |
| 170499 | NorCOMM2 | Pipox | Pipox_tm1b_A08 | -/- | M | 170499 | 10/9/2014 | 233.44 | 964.80 | 112.62 | 30.28 | 7.11 | 11.89 | 25.65 | 461.60 | 295.11 | 86.83 | 1095.90 | 653.18 | 75.44 | 688.75 | 714.82 | 686.25 | 1105.30 | 24.48 | 441.40 | 748.98 | 100.05 | 36.39 | 18.68 | 1.10 | 13.35 | 0.97 | 64.56 | 3.61 | 41.10 | 33.18 | 33.92 | 880.64 | 10204.00 | 6946.90 | 6690.30 | 1.35 | 45.42 | 125.53 | 9.99 | 1.52 | 5.31 | 18.53 | 3.15 | 4.30 | 911.30 | 660.33 | 2.81 | 0.89 | 723.18 | 1867.50 | 8597.60 | 3199.20 |
| 170406 | NorCOMM2 | Pipox | Pipox_tm1b_A08 | -/- | M | 170406 | 10/9/2014 | 242.16 | 827.56 | 107.60 | 56.44 | 9.10 | 10.63 | 21.25 | 452.87 | 298.83 | 103.38 | 1261.80 | 730.47 | 86.92 | 775.08 | 1001.80 | 731.49 | 1396.90 | 28.10 | 385.77 | 563.35 | 94.54 | 46.69 | 19.92 | 1.12 | 12.98 | 0.85 | 63.23 | 5.03 | 39.67 | 31.08 | 36.61 | 779.38 | 5914.60 | 5375.90 | 4888.70 | 1.52 | 31.04 | 147.93 | 10.23 | 1.66 | 5.66 | 22.54 | 3.48 | 6.02 | 1245.10 | 905.88 | 2.98 | 1.01 | 685.56 | 930.28 | 11620.00 | 4616.60 |
| 172415 | NorCOMM2 | Pipox | Pipox_tm1b_A08 | -/- | M | 172415 | 10/16/2014 | 239.14 | 725.89 | 91.17 | 34.66 | 4.21 | 11.01 | 20.19 | 274.23 | 257.82 | 95.00 | 1474.30 | 824.07 | 47.57 | 414.59 | 427.71 | 416.25 | 713.90 | 24.68 | 346.91 | 982.83 | 94.34 | 40.73 | 21.12 | 0.78 | 9.72 | 1.03 | 67.81 | 0.00 | 28.66 | 26.47 | 31.99 | 847.86 | 4713.30 | 3401.50 | 3576.20 | 0.76 | 42.52 | 128.09 | 4.51 | 1.45 | 5.56 | 24.11 | 0.00 | 3.99 | 1041.70 | 615.63 | 2.24 | 0.67 | 746.71 | 216.64 | 3529.20 | 1251.40 |
| 198681 | NorCOMM2 | Plk1 | Plk1_tm1b_E04 | +/- | F | 198681 | 12/23/2014 | 211.78 | 646.09 | 127.82 | 40.92 | 5.68 | 6.56 | 12.80 | 353.99 | 189.78 | 71.43 | 1636.11 | 451.15 | 24.31 | 128.25 | 143.22 | 113.41 | 194.61 | 20.11 | 424.92 | 1303.62 | 135.34 | 20.24 | 15.07 | 0.74 | 11.23 | 0.85 | 58.14 | 0.00 | 15.38 | 30.84 | 44.67 | 630.53 | 7501.17 | 5229.58 | 4766.34 | 1.44 | 110.77 | 86.52 | 1.77 | 1.73 | 3.95 | 13.03 | 0.00 | 4.94 | 1118.20 | 512.14 | 1.23 | 6.95 | 381.70 | 26.78 | 1606.21 | 596.01 |
| 198682 | NorCOMM2 | Plk1 | Plk1_tm1b_E04 | +/- | F | 198682 | 12/23/2014 | 191.54 | 769.48 | 137.67 | 38.36 | 4.52 | 9.34 | 10.57 | 337.80 | 282.97 | 88.20 | 1673.90 | 518.08 | 20.50 | 140.44 | 178.11 | 164.84 | 317.91 | 19.64 | 464.58 | 1482.42 | 114.29 | 22.04 | 15.93 | 1.30 | 9.47 | 0.92 | 72.53 | 2.02 | 22.04 | 31.94 | 40.98 | 765.49 | 6540.46 | 5189.82 | 4315.92 | 1.35 | 107.35 | 105.66 | 3.09 | 1.71 | 5.51 | 17.14 | 2.77 | 5.57 | 1322.92 | 493.75 | 1.15 | 7.10 | 399.88 | 19.33 | 1915.99 | 834.70 |
| 221097 | NorCOMM2 | Plk1 | Plk1_tm1b_E04 | +/- | F | 221097 | 2/12/2015 | 236.00 | 663.77 | 162.18 | 36.84 | 5.60 | 7.96 | 8.17 | 356.41 | 177.27 | 82.86 | 1540.01 | 439.98 | 21.80 | 130.13 | 130.03 | 126.54 | 190.06 | 26.02 | 556.90 | 2039.26 | 100.90 | 30.35 | 23.74 | 1.13 | 11.37 | 0.62 | 72.72 | 0.00 | 13.36 | 40.52 | 50.90 | 762.00 | 8471.88 | 4944.02 | 6873.26 | 1.58 | 286.53 | 123.03 | 3.25 | 1.94 | 4.58 | 18.69 | 2.64 | 6.74 | 1310.71 | 593.38 | 0.93 | 8.35 | 434.79 | 38.54 | 2138.44 | 801.72 |
| 215487 | NorCOMM2 | Plk1 | Plk1_tm1b_E04 | +/- | M | 215487 | 1/27/2015 | 243.48 | 646.81 | 135.36 | 17.31 | 8.27 | 9.95 | 8.41 | 437.79 | 326.09 | 124.40 | 1097.79 | 596.71 | 71.82 | 683.03 | 918.27 | 558.35 | 1342.34 | 21.72 | 407.03 | 518.49 | 105.63 | 29.48 | 21.90 | 0.78 | 10.84 | 0.85 | 81.45 | 0.00 | 58.75 | 25.16 | 26.34 | 693.96 | 9926.20 | 6600.87 | 8113.25 | 2.07 | 116.20 | 115.55 | 1.80 | 1.58 | 4.35 | 26.74 | 0.00 | 3.61 | 1078.87 | 512.70 | 0.72 | 0.68 | 708.33 | 2617.38 | 1294.61 | 547.17 |
| 198577 | NorCOMM2 | Plk1 | Plk1_tm1b_E04 | +/- | M | 198577 | 12/23/2014 | 235.03 | 743.21 | 113.83 | 48.01 | 5.98 | 11.88 | 12.31 | 393.20 | 228.82 | 109.94 | 1138.05 | 844.84 | 77.34 | 671.60 | 831.25 | 698.50 | 1425.85 | 28.13 | 503.68 | 504.65 | 114.57 | 28.80 | 18.17 | 1.07 | 12.21 | 1.34 | 57.10 | 2.46 | 54.46 | 29.15 | 31.20 | 789.52 | 10775.45 | 7881.85 | 9103.59 | 2.08 | 186.23 | 135.48 | 1.30 | 1.99 | 3.56 | 19.76 | 2.87 | 5.16 | 921.65 | 704.09 | 1.21 | 0.66 | 1046.11 | 5037.06 | 1483.64 | 538.53 |
| 198579 | NorCOMM2 | Plk1 | Plk1_tm1b_E04 | +/- | M | 198579 | 12/23/2014 | 276.89 | 697.17 | 123.91 | 38.51 | 5.62 | 9.24 | 13.42 | 342.60 | 193.97 | 100.20 | 1209.86 | 579.49 | 69.12 | 654.05 | 766.67 | 518.43 | 1050.06 | 23.58 | 339.85 | 573.90 | 111.57 | 35.39 | 18.51 | 1.17 | 10.91 | 0.95 | 64.60 | 2.64 | 59.00 | 37.94 | 31.70 | 763.92 | 6178.09 | 6492.90 | 6467.04 | 0.72 | 97.24 | 152.42 | 3.32 | 1.59 | 4.72 | 23.84 | 0.00 | 4.58 | 1137.14 | 594.28 | 1.68 | 0.53 | 701.17 | 78.42 | 2906.30 | 1120.15 |
| 294361 | NorCOMM2 | Pmm2 | Pmm2_tm1b_H04 | +/- | F | 294361 | 7/14/2015 | 294.42 | 606.30 | 178.50 | 31.99 | 4.43 | 9.85 | 7.86 | 470.97 | 340.26 | 105.61 | 2142.55 | 505.94 | 23.75 | 175.57 | 219.20 | 163.26 | 228.12 | 34.89 | 670.73 | 1291.73 | 87.61 | 39.55 | 8.93 | 1.11 | 10.18 | 0.91 | 83.86 | 0.00 | 17.47 | 41.93 | 48.68 | 746.32 | 8305.27 | 6213.88 | 6798.60 | 1.96 | 223.01 | 107.29 | 3.55 | 1.70 | 2.74 | 16.65 | 0.00 | 6.67 | 1223.41 | 590.77 | 0.00 | 8.45 | 464.82 | 26.42 | 2336.21 | 973.23 |
| 294362 | NorCOMM2 | Pmm2 | Pmm2_tm1b_H04 | +/- | F | 294362 | 7/14/2015 | 241.13 | 578.56 | 184.50 | 32.71 | 3.80 | 7.94 | 6.91 | 390.29 | 207.74 | 96.56 | 1616.55 | 580.85 | 25.53 | 145.19 | 199.38 | 132.66 | 232.20 | 26.36 | 513.07 | 1372.10 | 104.02 | 28.57 | 13.51 | 1.45 | 9.83 | 1.19 | 89.54 | 0.00 | 15.86 | 40.73 | 47.79 | 682.69 | 6040.92 | 4804.43 | 5500.02 | 2.10 | 71.78 | 108.64 | 1.69 | 1.87 | 0.00 | 18.90 | 0.00 | 5.37 | 1234.41 | 633.53 | 0.00 | 7.70 | 399.43 | 17.91 | 1528.49 | 518.71 |
| 294360 | NorCOMM2 | Pmm2 | Pmm2_tm1b_H04 | +/- | F | 294360 | 7/14/2015 | 198.39 | 562.18 | 154.21 | 35.45 | 11.31 | 6.58 | 9.12 | 462.73 | 292.77 | 105.36 | 1621.85 | 414.56 | 22.63 | 173.17 | 187.88 | 152.47 | 205.06 | 23.94 | 443.33 | 1477.32 | 81.60 | 37.56 | 10.02 | 1.05 | 9.39 | 0.68 | 90.48 | 0.00 | 19.72 | 35.86 | 46.50 | 675.79 | 6711.45 | 5537.16 | 6057.52 | 2.06 | 161.14 | 115.55 | 2.18 | 2.07 | 3.15 | 19.51 | 0.00 | 5.20 | 1334.66 | 489.00 | 0.00 | 5.43 | 335.77 | 29.33 | 984.28 | 493.78 |
| 294256 | NorCOMM2 | Pmm2 | Pmm2_tm1b_H04 | +/- | M | 294256 | 7/14/2015 | 266.00 | 765.60 | 140.37 | 23.83 | 2.90 | 9.51 | 6.52 | 589.17 | 295.48 | 125.69 | 1032.09 | 696.24 | 49.44 | 555.97 | 639.78 | 694.95 | 940.09 | 32.03 | 554.35 | 576.52 | 101.49 | 40.11 | 10.32 | 1.01 | 12.11 | 0.98 | 76.09 | 0.00 | 41.00 | 27.35 | 36.13 | 677.13 | 10356.10 | 6587.65 | 7014.29 | 2.22 | 123.63 | 161.36 | 1.95 | 2.63 | 4.31 | 17.54 | 1.57 | 4.33 | 965.70 | 574.66 | 0.00 | 0.00 | 743.84 | 512.82 | 952.25 | 373.27 |
| 294259 | NorCOMM2 | Pmm2 | Pmm2_tm1b_H04 | +/- | M | 294259 | 7/16/2015 | 208.93 | 717.11 | 139.24 | 26.90 | 3.41 | 10.97 | 11.77 | 338.75 | 255.08 | 142.70 | 992.26 | 647.87 | 54.50 | 494.28 | 633.78 | 499.31 | 1150.68 | 29.64 | 519.74 | 698.32 | 97.68 | 27.94 | 9.90 | 0.78 | 9.28 | 0.98 | 61.07 | 0.00 | 38.48 | 33.29 | 38.12 | 705.04 | 8490.23 | 8306.40 | 7618.00 | 0.82 | 125.06 | 148.27 | 4.79 | 2.04 | 5.17 | 18.89 | 2.06 | 4.12 | 963.38 | 608.18 | 1.08 | 0.00 | 739.17 | 541.95 | 2691.89 | 1199.46 |
| 294257 | NorCOMM2 | Pmm2 | Pmm2_tm1b_H04 | +/- | M | 294257 | 7/14/2015 | 195.01 | 912.89 | 144.03 | 35.23 | 3.16 | 9.08 | 8.77 | 585.02 | 322.14 | 114.34 | 967.66 | 696.41 | 73.30 | 604.32 | 681.81 | 663.71 | 1339.88 | 34.15 | 538.69 | 654.52 | 111.48 | 42.35 | 9.76 | 0.99 | 16.20 | 1.14 | 73.32 | 0.00 | 37.44 | 45.46 | 38.81 | 794.78 | 8533.88 | 5702.83 | 6233.07 | 1.37 | 139.07 | 171.70 | 4.31 | 2.11 | 4.89 | 17.81 | 1.80 | 5.23 | 1172.06 | 704.67 | 1.22 | 1.13 | 713.13 | 166.10 | 4098.65 | 1678.14 |
| 229188 | NorCOMM2 | Ptpn12 | Ptpn12_tm2b_H11 | +/- | F | 229188 | 3/17/2015 | 193.12 | 767.32 | 107.13 | 35.94 | 8.53 | 8.32 | 14.85 | 461.31 | 276.03 | 99.20 | 2101.00 | 504.76 | 30.72 | 161.32 | 218.58 | 150.77 | 230.85 | 25.41 | 406.09 | 1166.00 | 115.40 | 38.06 | 18.09 | 1.11 | 13.65 | 0.87 | 77.48 | 4.25 | 30.10 | 32.02 | 37.61 | 860.53 | 6095.00 | 4411.30 | 3880.90 | 2.31 | 44.62 | 96.08 | 9.50 | 1.81 | 4.47 | 18.05 | 2.39 | 5.95 | 1169.30 | 621.89 | 1.47 | 6.40 | 436.99 | 156.99 | 9255.40 | 3652.50 |
| 229189 | NorCOMM2 | Ptpn12 | Ptpn12_tm2b_H11 | +/- | F | 229189 | 3/17/2015 | 167.86 | 849.38 | 129.55 | 42.69 | 17.73 | 6.89 | 11.87 | 356.08 | 265.80 | 107.37 | 1492.80 | 649.65 | 25.53 | 108.67 | 147.79 | 129.38 | 147.29 | 21.35 | 505.75 | 1680.80 | 85.45 | 33.73 | 16.03 | 0.78 | 10.96 | 1.05 | 61.42 | 6.44 | 19.32 | 36.17 | 38.82 | 652.29 | 6137.60 | 4922.90 | 4725.20 | 1.52 | 167.74 | 93.95 | 3.96 | 1.61 | 0.00 | 21.30 | 0.00 | 6.90 | 1021.90 | 611.67 | 1.16 | 5.32 | 375.91 | 86.89 | 3325.20 | 1183.90 |
| 248916 | NorCOMM2 | Ptpn12 | Ptpn12_tm2b_H11 | +/- | F | 248916 | 4/23/2015 | 221.47 | 687.87 | 115.55 | 35.60 | 22.34 | 5.79 | 17.57 | 303.66 | 200.08 | 96.50 | 2053.10 | 493.59 | 33.23 | 119.27 | 178.98 | 128.20 | 177.88 | 25.14 | 391.00 | 1700.00 | 82.42 | 33.56 | 25.74 | 0.96 | 12.64 | 0.97 | 90.55 | 7.65 | 16.37 | 45.00 | 45.67 | 855.61 | 7766.40 | 5164.90 | 5295.30 | 2.52 | 129.67 | 105.90 | 3.89 | 1.96 | 5.13 | 43.80 | 3.74 | 5.65 | 1267.10 | 510.62 | 0.88 | 8.15 | 451.14 | 286.64 | 4714.20 | 1931.60 |
| 229084 | NorCOMM2 | Ptpn12 | Ptpn12_tm2b_H11 | +/- | M | 229084 | 3/19/2015 | 194.59 | 859.82 | 91.73 | 25.57 | 4.57 | 7.50 | 13.37 | 481.94 | 333.84 | 101.22 | 990.51 | 863.02 | 70.02 | 631.87 | 680.19 | 564.60 | 1218.00 | 24.24 | 436.46 | 642.61 | 86.39 | 26.10 | 10.06 | 0.82 | 12.37 | 0.79 | 59.38 | 1.42 | 46.18 | 31.10 | 25.57 | 812.95 | 7789.20 | 6371.10 | 6757.10 | 1.62 | 95.79 | 156.98 | 4.32 | 1.54 | 4.91 | 14.91 | 0.00 | 4.70 | 857.18 | 591.68 | 0.97 | 0.39 | 524.83 | 1562.10 | 1519.30 | 560.01 |
| 229082 | NorCOMM2 | Ptpn12 | Ptpn12_tm2b_H11 | +/- | M | 229082 | 3/19/2015 | 223.14 | 966.46 | 95.97 | 36.41 | 6.69 | 5.99 | 9.24 | 203.81 | 113.78 | 71.19 | 999.71 | 648.69 | 67.71 | 428.05 | 453.99 | 433.46 | 676.53 | 24.30 | 449.97 | 650.69 | 101.88 | 40.54 | 13.15 | 1.16 | 12.59 | 0.91 | 57.22 | 1.72 | 49.03 | 31.57 | 28.09 | 832.53 | 6988.10 | 4896.90 | 4746.70 | 1.17 | 97.04 | 105.51 | 8.81 | 1.32 | 6.11 | 17.99 | 4.61 | 5.03 | 999.26 | 673.35 | 1.50 | 0.59 | 882.37 | 337.83 | 7586.10 | 2547.40 |
| 229087 | NorCOMM2 | Ptpn12 | Ptpn12_tm2b_H11 | +/- | M | 229087 | 3/19/2015 | 237.85 | 1018.40 | 117.38 | 32.77 | 10.79 | 11.12 | 18.12 | 508.99 | 378.07 | 122.46 | 1188.30 | 742.84 | 57.97 | 669.46 | 744.36 | 538.21 | 1294.50 | 25.60 | 541.95 | 603.39 | 106.53 | 46.29 | 18.05 | 1.36 | 13.31 | 0.79 | 56.68 | 3.28 | 52.92 | 37.19 | 24.33 | 890.38 | 10490.00 | 7753.30 | 6456.80 | 2.66 | 216.76 | 154.28 | 4.05 | 2.09 | 0.00 | 22.84 | 2.33 | 5.79 | 1085.60 | 690.20 | 1.53 | 1.33 | 650.98 | 5078.40 | 2745.00 | 1170.90 |
| 449031 | KOMP2 | Pttg1 | Pttg1_tm1b_F08 | -/- | F | 449031 | 10/14/2016 | 179.39 | 611.39 | 178.48 | 45.76 | 8.58 | 6.52 | 12.47 | 480.06 | 331.59 | 138.35 | 1704.94 | 595.36 | 28.24 | 248.52 | 241.65 | 263.48 | 257.84 | 26.14 | 441.09 | 1035.75 | 129.42 | 29.84 | 15.24 | 0.93 | 10.42 | 1.17 | 79.06 | 1.80 | 26.12 | 36.78 | 46.12 | 771.51 | 5954.48 | 4682.07 | 5728.78 | 1.53 | 78.80 | 96.38 | 11.40 | 2.26 | 3.89 | 18.25 | 0.00 | 5.86 | 1064.25 | 509.73 | 2.42 | 8.77 | 431.48 | 24.36 | 10320.78 | 3976.68 |
| 449028 | KOMP2 | Pttg1 | Pttg1_tm1b_F08 | -/- | F | 449028 | 10/14/2016 | 129.96 | 452.79 | 142.17 | 30.80 | 2.97 | 6.70 | 11.12 | 332.87 | 192.10 | 68.22 | 1365.13 | 374.74 | 19.85 | 90.46 | 111.73 | 90.76 | 201.04 | 16.63 | 307.09 | 1139.03 | 70.34 | 17.01 | 8.86 | 0.82 | 7.85 | 1.26 | 54.72 | 0.00 | 13.84 | 28.96 | 35.88 | 499.87 | 5113.86 | 3835.55 | 3584.43 | 1.39 | 58.48 | 96.43 | 2.57 | 1.33 | 3.19 | 11.39 | 0.00 | 4.11 | 993.26 | 500.29 | 0.00 | 6.53 | 255.85 | 15.66 | 1770.62 | 833.41 |
| 493896 | KOMP2 | Pttg1 | Pttg1_tm1b_F08 | -/- | F | 493896 | 2/2/2017 | 204.27 | 511.21 | 149.80 | 29.56 | 7.20 | 9.83 | 8.58 | 327.67 | 184.71 | 95.40 | 1395.54 | 430.68 | 18.63 | 111.65 | 122.40 | 101.68 | 191.96 | 20.62 | 423.73 | 1705.49 | 71.60 | 27.78 | 9.62 | 0.74 | 10.53 | 0.91 | 79.36 | 2.31 | 11.55 | 32.77 | 45.06 | 678.25 | 3931.35 | 4437.01 | 3034.26 | 1.21 | 64.05 | 120.45 | 11.25 | 2.08 | 4.77 | 19.03 | 0.00 | 5.22 | 1260.72 | 598.17 | 0.90 | 7.13 | 403.22 | 25.81 | 7801.75 | 2916.47 |
| 481961 | KOMP2 | Pttg1 | Pttg1_tm1b_F08 | -/- | M | 481961 | 12/21/2016 | 254.54 | 755.19 | 145.66 | 26.72 | 10.42 | 12.67 | 7.61 | 347.35 | 249.37 | 117.48 | 1044.55 | 662.69 | 40.89 | 350.83 | 379.61 | 358.27 | 543.06 | 30.01 | 547.10 | 908.62 | 146.15 | 24.98 | 15.64 | 1.28 | 10.63 | 1.07 | 68.73 | 0.00 | 22.40 | 39.14 | 49.74 | 828.37 | 9236.64 | 7315.48 | 6509.87 | 0.83 | 109.33 | 125.67 | 6.89 | 2.60 | 8.43 | 24.20 | 2.08 | 4.59 | 1040.98 | 687.27 | 0.00 | 0.93 | 839.69 | 214.33 | 4688.09 | 2071.27 |
| 481962 | KOMP2 | Pttg1 | Pttg1_tm1b_F08 | -/- | M | 481962 | 12/21/2016 | 259.07 | 735.17 | 129.99 | 29.68 | 28.69 | 10.23 | 14.62 | 448.42 | 241.24 | 112.17 | 1308.33 | 597.95 | 43.70 | 386.50 | 407.11 | 405.57 | 677.09 | 24.75 | 451.80 | 793.33 | 138.70 | 32.69 | 12.13 | 1.09 | 11.80 | 1.10 | 80.37 | 4.15 | 26.55 | 36.79 | 40.59 | 653.87 | 8397.64 | 7547.06 | 6379.50 | 1.26 | 228.62 | 159.43 | 4.33 | 1.97 | 10.03 | 28.86 | 1.69 | 3.55 | 896.50 | 586.42 | 1.61 | 0.90 | 754.88 | 138.42 | 2644.45 | 933.85 |
| 448912 | KOMP2 | Pttg1 | Pttg1_tm1b_F08 | -/- | M | 448912 | 10/14/2016 | 225.37 | 695.70 | 139.48 | 23.47 | 8.51 | 13.31 | 13.12 | 484.92 | 421.45 | 106.14 | 1246.53 | 505.13 | 57.77 | 340.82 | 445.87 | 321.85 | 707.40 | 25.74 | 391.81 | 805.71 | 116.81 | 26.13 | 15.88 | 1.58 | 10.74 | 1.56 | 69.41 | 0.00 | 22.92 | 38.22 | 47.49 | 873.89 | 7727.97 | 5817.18 | 8183.87 | 1.10 | 123.37 | 147.74 | 7.84 | 1.33 | 5.16 | 27.42 | 0.00 | 4.76 | 1386.99 | 708.58 | 2.47 | 1.05 | 707.77 | 358.83 | 6760.52 | 3181.86 |
| 230294 | NorCOMM2 | Rock1 | Rock1_tm1b_B11 | +/- | F | 230294 | 3/24/2015 | 239.07 | 810.18 | 87.82 | 33.21 | 6.13 | 9.12 | 17.76 | 389.08 | 220.61 | 79.90 | 1701.60 | 571.03 | 36.59 | 123.59 | 177.68 | 131.78 | 205.27 | 22.22 | 436.47 | 1462.00 | 101.10 | 39.83 | 19.11 | 1.30 | 13.18 | 1.14 | 71.23 | 3.72 | 17.41 | 34.50 | 33.81 | 698.10 | 7055.90 | 3858.50 | 4437.00 | 1.85 | 61.03 | 98.34 | 9.27 | 2.04 | 0.00 | 15.10 | 0.00 | 6.72 | 1226.90 | 446.79 | 1.15 | 7.71 | 378.11 | 216.64 | 9730.60 | 2977.80 |
| 230292 | NorCOMM2 | Rock1 | Rock1_tm1b_B11 | +/- | F | 230292 | 3/24/2015 | 229.24 | 528.07 | 102.62 | 21.28 | 4.60 | 7.14 | 8.93 | 321.92 | 184.98 | 63.37 | 1511.20 | 343.92 | 25.92 | 122.94 | 140.64 | 99.80 | 212.11 | 20.70 | 382.23 | 1352.00 | 66.95 | 23.71 | 16.24 | 1.02 | 12.19 | 0.98 | 61.44 | 2.59 | 8.07 | 39.63 | 53.89 | 661.87 | 7275.40 | 4897.40 | 4997.40 | 1.27 | 126.60 | 103.77 | 3.90 | 2.27 | 0.00 | 16.55 | 0.00 | 3.82 | 1313.10 | 788.02 | 0.78 | 8.25 | 349.26 | 19.58 | 1797.80 | 681.60 |
| 230291 | NorCOMM2 | Rock1 | Rock1_tm1b_B11 | +/- | F | 230291 | 3/25/2015 | 159.08 | 679.78 | 100.02 | 41.84 | 4.47 | 8.01 | 14.56 | 265.35 | 169.61 | 74.86 | 1323.40 | 425.88 | 21.06 | 99.02 | 106.20 | 83.94 | 168.74 | 15.30 | 346.71 | 1598.90 | 83.15 | 24.81 | 11.82 | 0.98 | 12.33 | 0.74 | 76.58 | 3.50 | 12.99 | 34.70 | 34.53 | 656.40 | 3444.40 | 2891.30 | 3310.30 | 1.32 | 39.13 | 102.44 | 7.69 | 1.60 | 4.47 | 14.54 | 2.45 | 5.61 | 1292.80 | 400.93 | 1.48 | 6.31 | 359.07 | 27.33 | 7912.30 | 2634.00 |
| 225684 | NorCOMM2 | Rock1 | Rock1_tm1b_B11 | +/- | M | 225684 | 3/5/2015 | 253.21 | 862.32 | 95.12 | 27.50 | 4.88 | 10.67 | 12.53 | 400.09 | 248.78 | 136.91 | 1134.10 | 755.34 | 83.24 | 567.02 | 641.16 | 618.89 | 1280.10 | 26.89 | 456.27 | 573.96 | 121.45 | 22.11 | 29.43 | 1.05 | 12.52 | 0.87 | 49.96 | 2.96 | 47.18 | 29.99 | 32.94 | 681.59 | 9820.50 | 7378.70 | 7863.90 | 1.71 | 122.08 | 134.24 | 2.22 | 1.53 | 5.64 | 24.12 | 6.57 | 5.09 | 911.21 | 595.85 | 0.61 | 0.70 | 908.64 | 3590.90 | 2220.50 | 847.85 |
| 225682 | NorCOMM2 | Rock1 | Rock1_tm1b_B11 | +/- | M | 225682 | 3/3/2015 | 260.92 | 951.30 | 126.00 | 33.70 | 5.08 | 7.90 | 16.58 | 487.39 | 259.34 | 119.64 | 1023.50 | 797.55 | 76.25 | 586.21 | 939.70 | 663.11 | 1521.00 | 28.71 | 413.31 | 421.91 | 114.04 | 28.74 | 16.52 | 1.25 | 14.04 | 0.88 | 45.36 | 4.60 | 46.78 | 30.85 | 34.05 | 765.82 | 8941.30 | 7971.50 | 6826.50 | 1.98 | 169.25 | 165.24 | 2.01 | 1.66 | 2.74 | 17.14 | 4.43 | 4.01 | 1162.60 | 578.35 | 1.48 | 0.96 | 882.44 | 1181.40 | 1338.20 | 483.47 |
| 225681 | NorCOMM2 | Rock1 | Rock1_tm1b_B11 | +/- | M | 225681 | 3/3/2015 | 227.89 | 786.67 | 103.84 | 42.92 | 2.97 | 6.96 | 21.55 | 413.00 | 252.10 | 111.66 | 1252.00 | 1039.80 | 61.75 | 635.11 | 610.31 | 649.51 | 825.67 | 24.75 | 427.49 | 516.24 | 109.97 | 26.21 | 21.60 | 1.31 | 12.57 | 0.83 | 64.72 | 1.41 | 44.51 | 19.70 | 33.54 | 614.63 | 5941.90 | 4423.20 | 4066.90 | 1.44 | 34.58 | 94.94 | 3.51 | 1.55 | 8.06 | 20.68 | 5.18 | 4.04 | 851.98 | 569.57 | 1.48 | 0.68 | 780.26 | 3907.80 | 2239.30 | 760.41 |
| 35846 | NorCOMM2 | Sra1 | Sra1_tm1b_C01 | -/- | F | 35846 | 11/28/2013 | 247.13 | 649.16 | 154.31 | 34.95 | 11.70 | 6.56 | 9.41 | 335.50 | 235.58 | 93.98 | 1304.54 | 541.12 | 28.21 | 88.05 | 107.44 | 84.57 | 173.94 | 21.21 | 465.25 | 2430.82 | 90.97 | 26.72 | 19.11 | 1.28 | 9.36 | 0.84 | 78.94 | 6.00 | 11.40 | 31.00 | 50.29 | 794.80 | 4926.82 | 3729.43 | 3422.51 | 1.03 | 33.71 | 109.30 | 30.29 | 1.95 | 5.54 | 18.65 | 0.00 | 6.65 | 1471.96 | 430.80 | 1.93 | 9.21 | 474.89 | 12.99 | 32623.67 | 14595.41 |
| 39361 | NorCOMM2 | Sra1 | Sra1_tm1b_C01 | -/- | F | 39361 | 12/11/2013 | 190.96 | 775.56 | 116.96 | 39.37 | 4.80 | 8.19 | 12.26 | 481.49 | 289.46 | 96.87 | 1639.51 | 442.59 | 28.52 | 162.01 | 174.54 | 150.69 | 212.46 | 31.69 | 492.34 | 1885.34 | 125.16 | 32.77 | 12.73 | 1.19 | 11.95 | 0.96 | 62.25 | 0.00 | 14.38 | 25.11 | 51.44 | 786.57 | 4172.53 | 3280.00 | 3401.40 | 1.87 | 39.31 | 114.02 | 10.79 | 1.86 | 3.23 | 13.78 | 0.00 | 6.86 | 1244.99 | 596.05 | 1.87 | 10.63 | 403.42 | 113.59 | 10562.55 | 4476.53 |
| 39360 | NorCOMM2 | Sra1 | Sra1_tm1b_C01 | -/- | F | 39360 | 12/11/2013 | 240.06 | 788.26 | 156.24 | 42.78 | 6.12 | 8.08 | 12.49 | 281.51 | 214.79 | 96.99 | 1755.45 | 431.99 | 24.42 | 159.87 | 208.69 | 153.58 | 229.91 | 26.25 | 414.99 | 1800.04 | 132.29 | 42.01 | 15.47 | 1.35 | 11.06 | 1.41 | 89.40 | 2.54 | 16.01 | 31.27 | 47.82 | 906.66 | 4424.70 | 3169.11 | 4283.31 | 1.52 | 30.67 | 98.80 | 18.42 | 2.44 | 3.62 | 25.00 | 3.20 | 5.93 | 1207.94 | 659.25 | 2.16 | 7.97 | 399.82 | 31.03 | 21331.76 | 7638.79 |
| 69285 | NorCOMM2 | Sra1 | Sra1_tm1b_C01 | -/- | M | 69285 | 3/4/2014 | 239.91 | 713.18 | 112.12 | 43.76 | 6.86 | 7.52 | 15.76 | 352.96 | 336.14 | 96.15 | 1028.25 | 621.48 | 69.87 | 720.12 | 857.46 | 616.29 | 1121.50 | 25.23 | 488.92 | 531.20 | 113.68 | 34.10 | 19.34 | 1.10 | 11.48 | 0.86 | 69.26 | 6.38 | 56.45 | 28.77 | 33.26 | 735.18 | 9488.12 | 4982.46 | 5253.19 | 1.39 | 43.81 | 146.07 | 11.69 | 1.81 | 4.17 | 27.11 | 0.00 | 5.03 | 815.34 | 738.94 | 2.73 | 0.93 | 762.21 | 997.04 | 11744.72 | 4612.92 |
| 35950 | NorCOMM2 | Sra1 | Sra1_tm1b_C01 | -/- | M | 35950 | 11/28/2013 | 257.70 | 677.94 | 158.15 | 31.58 | 7.09 | 8.99 | 10.95 | 452.28 | 291.18 | 95.82 | 1080.03 | 675.25 | 71.75 | 580.69 | 604.85 | 445.10 | 997.82 | 23.51 | 439.69 | 636.08 | 111.02 | 29.66 | 13.59 | 1.26 | 13.01 | 0.82 | 68.02 | 4.15 | 42.22 | 30.81 | 43.45 | 721.79 | 9788.76 | 6849.50 | 7828.69 | 1.19 | 95.85 | 118.04 | 11.87 | 1.73 | 5.11 | 17.49 | 2.80 | 4.24 | 982.36 | 632.98 | 2.81 | 0.38 | 663.20 | 209.91 | 16273.85 | 5194.89 |
| 35948 | NorCOMM2 | Sra1 | Sra1_tm1b_C01 | -/- | M | 35948 | 11/28/2013 | 198.62 | 626.75 | 118.92 | 32.55 | 11.42 | 5.13 | 14.91 | 311.03 | 302.82 | 79.55 | 1758.66 | 651.90 | 27.32 | 127.43 | 181.84 | 109.52 | 207.43 | 24.96 | 421.34 | 1806.16 | 106.87 | 31.72 | 17.47 | 1.53 | 12.86 | 1.01 | 70.68 | 5.67 | 16.37 | 40.08 | 41.78 | 755.32 | 4968.99 | 3941.53 | 3701.83 | 1.68 | 49.45 | 100.06 | 22.04 | 1.79 | 3.02 | 17.54 | 2.45 | 5.08 | 1534.06 | 618.05 | 2.76 | 6.34 | 326.16 | 13.13 | 23238.23 | 7697.71 |
| 487268 | K2P2 | Ulk3 | Ulk3_em2_del | -/- | F | 487268 | 1/4/2017 | 229.48 | 689.89 | 182.58 | 33.50 | 3.14 | 8.01 | 7.04 | 354.81 | 287.61 | 126.19 | 1386.22 | 531.96 | 26.42 | 114.52 | 152.95 | 115.66 | 289.44 | 36.41 | 525.04 | 1446.65 | 116.75 | 24.93 | 10.43 | 1.25 | 9.29 | 0.78 | 63.13 | 0.00 | 13.30 | 46.16 | 39.49 | 610.00 | 9274.91 | 5289.43 | 5798.03 | 1.74 | 174.71 | 100.93 | 2.46 | 1.39 | 4.53 | 15.48 | 2.05 | 4.74 | 1329.55 | 498.49 | 0.00 | 6.71 | 396.57 | 17.75 | 1096.33 | 527.83 |
| 487251 | K2P2 | Ulk3 | Ulk3_em2_del | -/- | F | 487251 | 12/29/2016 | 248.40 | 666.97 | 148.41 | 34.00 | 4.19 | 9.29 | 14.40 | 604.91 | 405.42 | 113.55 | 859.21 | 551.81 | 24.59 | 123.97 | 151.60 | 105.92 | 216.31 | 25.24 | 617.48 | 1154.55 | 98.36 | 30.94 | 10.53 | 0.93 | 11.07 | 0.90 | 54.39 | 0.00 | 15.09 | 33.90 | 49.62 | 667.54 | 8074.07 | 5986.30 | 7484.53 | 1.82 | 229.00 | 153.12 | 3.53 | 1.87 | 4.50 | 11.49 | 2.43 | 5.97 | 1128.27 | 572.22 | 0.00 | 7.31 | 423.97 | 31.77 | 3810.66 | 1462.85 |
| 487266 | K2P2 | Ulk3 | Ulk3_em2_del | -/- | F | 487266 | 1/4/2017 | 220.68 | 618.14 | 181.48 | 39.77 | 4.97 | 11.35 | 16.60 | 453.27 | 311.98 | 102.34 | 1988.15 | 619.61 | 30.35 | 135.02 | 176.97 | 116.02 | 262.57 | 26.16 | 439.33 | 1192.42 | 98.15 | 29.22 | 7.95 | 1.29 | 11.78 | 0.93 | 69.66 | 0.00 | 14.21 | 31.04 | 48.92 | 826.36 | 9574.87 | 7390.30 | 7475.70 | 2.95 | 227.00 | 143.58 | 2.25 | 1.98 | 3.49 | 19.09 | 1.37 | 5.96 | 1124.44 | 685.21 | 0.00 | 5.74 | 319.08 | 54.26 | 2409.09 | 825.68 |
| 487154 | K2P2 | Ulk3 | Ulk3_em2_del | -/- | M | 487154 | 1/4/2017 | 209.69 | 887.25 | 53.98 | 23.85 | 5.12 | 7.19 | 11.04 | 428.91 | 293.01 | 142.98 | 1030.22 | 812.76 | 83.78 | 705.56 | 902.44 | 796.52 | 1590.73 | 35.83 | 522.82 | 467.86 | 93.75 | 25.12 | 12.23 | 1.02 | 11.57 | 1.09 | 65.02 | 3.18 | 43.99 | 23.54 | 35.70 | 880.20 | 6614.77 | 5417.13 | 4429.62 | 2.36 | 75.70 | 145.15 | 6.04 | 2.46 | 4.98 | 27.81 | 1.68 | 5.36 | 905.84 | 745.23 | 2.59 | 1.42 | 604.20 | 6950.67 | 5400.32 | 2244.46 |
| 487149 | K2P2 | Ulk3 | Ulk3_em2_del | -/- | M | 487149 | 12/29/2016 | 247.86 | 781.58 | 123.32 | 29.55 | 4.09 | 10.59 | 15.88 | 612.65 | 376.26 | 138.80 | 1503.94 | 1138.68 | 84.70 | 695.24 | 706.29 | 812.44 | 1572.01 | 51.03 | 569.00 | 376.64 | 123.30 | 25.88 | 11.55 | 1.35 | 12.67 | 0.96 | 77.42 | 0.00 | 47.64 | 35.83 | 31.73 | 1013.63 | 18325.62 | 13404.21 | 14713.81 | 3.02 | 371.76 | 178.86 | 0.00 | 2.12 | 0.00 | 24.04 | 0.00 | 6.70 | 962.06 | 590.35 | 0.00 | 0.92 | 709.67 | 13755.56 | 415.52 | 168.75 |
| 487163 | K2P2 | Ulk3 | Ulk3_em2_del | -/- | M | 487163 | 1/4/2017 | 265.06 | 735.26 | 122.13 | 31.78 | 6.29 | 7.93 | 12.90 | 573.53 | 402.14 | 150.37 | 1678.94 | 1052.50 | 69.32 | 627.45 | 1031.29 | 556.03 | 1174.88 | 52.07 | 744.39 | 447.44 | 99.42 | 26.04 | 13.75 | 1.15 | 12.67 | 0.95 | 69.92 | 1.27 | 56.16 | 41.68 | 38.39 | 961.28 | 15083.44 | 11804.26 | 11831.13 | 3.41 | 354.55 | 163.70 | 3.26 | 2.86 | 0.00 | 22.48 | 0.00 | 5.50 | 1086.15 | 708.26 | 0.00 | 1.26 | 666.42 | 15610.00 | 3400.24 | 1456.76 |
| 284228 | NorCOMM2 | Ywhaz | Ywhaz_tm1b_A11 | +/- | F | 284228 | 6/30/2015 | 222.39 | 851.37 | 111.17 | 31.04 | 4.00 | 8.81 | 9.40 | 269.22 | 196.60 | 87.78 | 1854.10 | 656.18 | 34.62 | 252.78 | 293.85 | 211.27 | 348.63 | 32.31 | 484.95 | 1395.90 | 131.87 | 26.63 | 27.88 | 1.11 | 13.05 | 0.64 | 72.10 | 0.00 | 16.36 | 34.93 | 47.42 | 772.65 | 7318.70 | 6834.90 | 6003.50 | 2.34 | 284.71 | 116.56 | 1.95 | 1.90 | 5.91 | 26.67 | 4.17 | 4.95 | 1185.20 | 660.66 | 1.06 | 4.61 | 595.95 | 182.76 | 1245.30 | 474.43 |
| 294641 | NorCOMM2 | Ywhaz | Ywhaz_tm1b_A11 | +/- | F | 294641 | 7/14/2015 | 229.09 | 768.82 | 126.06 | 37.23 | 3.60 | 7.88 | 16.93 | 422.17 | 264.01 | 95.05 | 1739.50 | 472.49 | 32.43 | 199.20 | 275.51 | 191.01 | 332.22 | 21.83 | 435.40 | 1279.00 | 106.92 | 42.35 | 18.15 | 0.60 | 11.00 | 0.79 | 59.35 | 1.02 | 26.00 | 36.68 | 38.69 | 729.16 | 7898.70 | 5268.90 | 5458.00 | 1.81 | 214.24 | 136.15 | 3.98 | 2.12 | 4.54 | 19.26 | 2.64 | 5.88 | 1365.50 | 605.94 | 1.59 | 6.28 | 386.04 | 93.48 | 2748.40 | 908.22 |
| 294642 | NorCOMM2 | Ywhaz | Ywhaz_tm1b_A11 | +/- | F | 294642 | 7/14/2015 | 195.25 | 614.35 | 101.91 | 34.78 | 2.86 | 8.73 | 15.44 | 336.93 | 175.71 | 80.39 | 1461.70 | 490.42 | 26.26 | 84.58 | 91.22 | 73.88 | 118.04 | 23.79 | 300.64 | 1852.40 | 53.11 | 26.96 | 15.21 | 0.69 | 10.93 | 1.38 | 62.86 | 0.00 | 6.05 | 44.68 | 42.26 | 617.34 | 6144.60 | 4330.70 | 4566.70 | 1.79 | 93.21 | 111.53 | 3.31 | 2.19 | 6.17 | 31.73 | 0.00 | 5.68 | 1262.50 | 643.80 | 1.01 | 4.59 | 363.79 | 59.22 | 1756.10 | 499.24 |
| 295044 | NorCOMM2 | Ywhaz | Ywhaz_tm1b_A11 | +/- | M | 295044 | 7/16/2015 | 280.66 | 902.63 | 133.45 | 35.43 | 3.77 | 8.66 | 19.54 | 436.84 | 282.46 | 113.70 | 1100.60 | 762.12 | 72.68 | 612.56 | 655.32 | 597.38 | 1399.60 | 29.77 | 533.05 | 609.80 | 94.69 | 37.59 | 18.79 | 1.15 | 10.58 | 0.88 | 83.43 | 3.05 | 39.04 | 34.25 | 32.90 | 784.80 | 8435.70 | 8027.80 | 7244.20 | 1.62 | 233.06 | 156.47 | 4.05 | 1.90 | 7.19 | 37.67 | 4.00 | 4.39 | 1148.30 | 734.80 | 1.12 | 0.88 | 972.28 | 1925.30 | 4663.90 | 1570.40 |
| 295043 | NorCOMM2 | Ywhaz | Ywhaz_tm1b_A11 | +/- | M | 295043 | 7/16/2015 | 221.23 | 870.45 | 101.53 | 36.06 | 9.71 | 11.43 | 15.78 | 644.03 | 504.40 | 119.51 | 1809.80 | 1024.20 | 99.64 | 690.43 | 741.79 | 730.38 | 1564.40 | 31.72 | 522.47 | 477.34 | 99.87 | 35.88 | 22.93 | 0.96 | 13.53 | 1.38 | 64.22 | 3.68 | 64.47 | 37.40 | 31.50 | 706.70 | 13406.00 | 9228.30 | 7296.80 | 2.90 | 331.33 | 113.23 | 2.38 | 2.29 | 3.61 | 33.86 | 3.32 | 5.30 | 1168.80 | 625.51 | 1.10 | 0.45 | 696.13 | 7041.20 | 1746.70 | 551.02 |
| 295040 | NorCOMM2 | Ywhaz | Ywhaz_tm1b_A11 | +/- | M | 295040 | 7/16/2015 | 273.89 | 927.56 | 94.54 | 40.02 | 7.46 | 13.05 | 19.07 | 701.47 | 397.34 | 125.99 | 2444.50 | 1263.80 | 94.24 | 553.52 | 890.21 | 595.92 | 1352.90 | 58.60 | 607.91 | 641.91 | 155.53 | 33.99 | 19.78 | 1.10 | 13.80 | 1.29 | 75.58 | 2.41 | 89.82 | 31.80 | 34.27 | 844.85 | 16775.00 | 14661.00 | 14519.00 | 4.03 | 240.15 | 149.37 | 4.92 | 3.89 | 3.10 | 28.55 | 2.40 | 6.44 | 814.89 | 727.70 | 1.25 | 0.96 | 750.94 | 30123.00 | 3117.40 | 1108.80 |

| Sample | Project | Gene symbol | Mouse line name | Zygosity | Gender | Mouse BCode | date collected | Hbz | Hpx | Serpind1 | Hgfac | Hrg | Habp2 | Ig alpha chain C region | Ighg1 | Ig gamma-2A chain C region sec | Igh-3 | Ig gamma-3 chain C region | Ig heavy chain V region MOPC 47A | Igkc | Igk-V19-17 | Ig kappa chain V-II region 17S | Ig kappa chain V-II region 26 | Ig kappa chain V-II region 7S3 | Ig kappa chain V-III region PC | Ig kappa chain V-IV region S10 | Ig kappa chain V-V region K2 | Ig kappa chain V-V region MOPC 149 | Ig kappa chain V-V region MOPC 173 | Ig kappa chain V-VI region NQ2 | Ighm | Jchain | Ica | Igf1 | Igfals | Itih4 | Itih1 | Itih3 | ITIH2 | Icam2 | Il1rap | Il18bp | Krt76 | Kng1 | Spint1 | Lifr | Sell | Lum | Man2b1 | Ctsa | Csf1r | Masp2 | Masp1 | Mbl1 | Mbl2 | Msln | Mfap4 | Mug1 | Pglyrp2 |
| --- | --- | --- | --- | --- | --- | --- | --- | --- | --- | --- | --- | --- | --- | --- | --- | --- | --- | --- | --- | --- | --- | --- | --- | --- | --- | --- | --- | --- | --- | --- | --- | --- | --- | --- | --- | --- | --- | --- | --- | --- | --- | --- | --- | --- | --- | --- | --- | --- | --- | --- | --- | --- | --- | --- | --- | --- | --- | --- | --- |
| 448371 | KOMP2 | (null) | C57BL/6NCrl | +/+ | F | 448371 | 10/12/2016 | 1948.93 | 2334.42 | 234.38 | 79.28 | 908.90 | 24.31 | 40.33 | 1314.21 | 2194.53 | 3279.21 | 703.68 | 179.21 | 4570.38 | 405.05 | 16.95 | 96.27 | 99.51 | 24.04 | 126.94 | 47.34 | 147.01 | 1.84 | 75.52 | 1565.16 | 168.49 | 369.68 | 20.82 | 103.25 | 510.71 | 354.66 | 400.18 | 533.97 | 1.50 | 193.50 | 6.48 | 0.00 | 4688.39 | 6.10 | 287.53 | 18.17 | 44.93 | 11.00 | 3.83 | 50.78 | 15.89 | 15.19 | 214.90 | 4909.44 | 3.12 | 3.71 | 4363.07 | 90.77 |
| 115863 | NorCOMM2 | (null) | C57BL/6NCrl | +/+ | F | 115863 | 6/5/2014 | 15012.57 | 1410.42 | 216.98 | 78.02 | 807.27 | 25.80 | 39.79 | 3794.64 | 1753.76 | 5450.01 | 2722.81 | 69.34 | 6390.43 | 1172.16 | 18.04 | 92.82 | 47.02 | 27.22 | 108.55 | 121.57 | 71.76 | 3.75 | 36.85 | 2086.51 | 215.75 | 308.99 | 15.29 | 100.45 | 496.03 | 376.98 | 339.76 | 525.94 | 1.94 | 146.74 | 7.49 | 0.00 | 4156.75 | 3.58 | 289.41 | 25.05 | 66.91 | 10.12 | 3.65 | 72.03 | 11.86 | 14.94 | 217.05 | 4914.48 | 4.05 | 3.31 | 3983.32 | 91.07 |
| 432638 | KOMP2 | (null) | C57BL/6NCrl | +/+ | F | 432638 | 9/1/2016 | 1914.76 | 1664.59 | 166.06 | 70.18 | 764.52 | 17.48 | 19.27 | 2528.37 | 1595.38 | 3152.21 | 1420.81 | 84.67 | 6688.67 | 165.59 | 14.80 | 61.12 | 49.17 | 16.23 | 65.15 | 45.98 | 39.83 | 1.30 | 35.81 | 3712.92 | 324.39 | 254.44 | 10.61 | 72.52 | 362.07 | 327.76 | 316.13 | 360.01 | 1.81 | 142.08 | 4.49 | 3.67 | 4192.88 | 3.45 | 167.13 | 20.70 | 38.20 | 5.97 | 3.46 | 45.85 | 13.50 | 9.83 | 185.37 | 2728.47 | 2.33 | 2.14 | 2819.10 | 66.15 |
| 237715 | NorCOMM2 | (null) | C57BL/6NCrl | +/+ | F | 237715 | 4/21/2015 | 10007.07 | 1832.98 | 207.76 | 80.89 | 878.10 | 20.88 | 17.44 | 655.73 | 502.67 | 1425.78 | 672.12 | 166.94 | 2289.11 | 94.39 | 16.16 | 75.91 | 29.49 | 17.58 | 74.17 | 30.63 | 43.20 | 1.18 | 20.86 | 1998.76 | 152.77 | 324.32 | 13.03 | 94.89 | 440.57 | 223.70 | 352.26 | 587.80 | 1.65 | 125.53 | 5.08 | 0.00 | 4780.27 | 3.37 | 373.37 | 27.22 | 41.46 | 11.51 | 3.85 | 50.50 | 10.68 | 11.72 | 196.23 | 3450.61 | 2.82 | 3.60 | 3843.45 | 77.29 |
| 496095 | K2P2 | (null) | C57BL/6NCrl | +/+ | F | 496095 | 2/14/2017 | 2422.60 | 1643.30 | 215.60 | 93.09 | 1239.38 | 24.94 | 22.80 | 2717.75 | 1776.24 | 2800.64 | 921.65 | 156.43 | 4623.37 | 300.66 | 11.67 | 64.62 | 154.62 | 22.85 | 127.10 | 26.61 | 223.71 | 2.73 | 63.21 | 3714.40 | 277.80 | 322.55 | 22.13 | 82.20 | 622.78 | 278.07 | 458.49 | 645.49 | 1.67 | 176.76 | 7.25 | 0.00 | 4776.87 | 3.35 | 293.54 | 32.35 | 56.64 | 10.57 | 3.20 | 67.13 | 11.61 | 13.40 | 172.58 | 4305.66 | 3.86 | 5.00 | 3134.57 | 96.50 |
| 229694 | NorCOMM2 | (null) | C57BL/6NCrl | +/+ | F | 229694 | 3/26/2015 | 10941.77 | 1611.97 | 227.88 | 85.55 | 1030.65 | 24.79 | 24.10 | 4521.00 | 666.42 | 2454.83 | 2613.60 | 286.39 | 4206.81 | 297.07 | 13.98 | 97.04 | 141.45 | 5.53 | 78.74 | 33.07 | 62.05 | 1.14 | 32.70 | 1414.96 | 129.54 | 312.37 | 13.52 | 85.29 | 413.19 | 259.78 | 289.32 | 441.64 | 1.67 | 195.15 | 5.55 | 0.00 | 4321.76 | 3.68 | 334.23 | 23.26 | 66.21 | 8.00 | 4.22 | 54.71 | 14.04 | 15.69 | 235.55 | 4223.73 | 3.45 | 3.73 | 4403.33 | 78.28 |
| 417794 | KOMP2 | (null) | C57BL/6NCrl | +/+ | F | 417794 | 7/21/2016 | 1780.45 | 1700.88 | 187.53 | 66.66 | 982.01 | 23.58 | 41.46 | 1644.39 | 697.97 | 5524.30 | 1780.96 | 197.71 | 6223.43 | 465.11 | 21.80 | 150.26 | 49.13 | 23.49 | 126.33 | 47.10 | 81.78 | 1.69 | 55.18 | 4275.97 | 274.32 | 318.91 | 15.49 | 98.75 | 424.84 | 423.30 | 403.07 | 594.16 | 1.69 | 180.13 | 7.05 | 0.00 | 4766.09 | 4.51 | 319.13 | 19.48 | 40.36 | 10.19 | 2.67 | 51.20 | 13.21 | 11.89 | 211.13 | 3881.93 | 3.06 | 4.33 | 3896.65 | 85.68 |
| 220169 | NorCOMM2 | (null) | C57BL/6NCrl | +/+ | F | 220169 | 2/12/2015 | 3658.71 | 1677.80 | 231.86 | 80.30 | 1198.09 | 25.56 | 20.76 | 7087.59 | 901.25 | 2077.37 | 1544.70 | 226.80 | 7451.42 | 159.44 | 16.31 | 106.67 | 34.01 | 22.45 | 151.95 | 20.52 | 121.14 | 1.44 | 27.27 | 5096.18 | 426.22 | 365.48 | 15.25 | 83.30 | 485.57 | 186.03 | 353.88 | 549.84 | 2.07 | 162.61 | 7.01 | 0.00 | 4436.34 | 5.29 | 336.17 | 23.07 | 45.78 | 9.92 | 3.03 | 54.34 | 13.37 | 11.23 | 195.43 | 4555.69 | 3.14 | 4.59 | 3423.70 | 99.73 |
| 170955 | NorCOMM2 | (null) | C57BL/6NCrl | +/+ | F | 170955 | 10/9/2014 | 10426.85 | 2028.64 | 202.02 | 83.59 | 771.91 | 21.38 | 16.82 | 5042.00 | 1266.41 | 2790.37 | 1092.80 | 225.51 | 4855.25 | 314.91 | 17.46 | 270.28 | 28.57 | 6.58 | 71.27 | 28.33 | 110.52 | 4.35 | 18.26 | 1355.41 | 115.32 | 396.95 | 23.54 | 113.56 | 461.64 | 219.15 | 375.57 | 591.57 | 2.61 | 159.41 | 5.83 | 5.75 | 5271.80 | 3.83 | 231.73 | 23.93 | 63.39 | 10.61 | 3.84 | 60.08 | 16.26 | 16.49 | 194.74 | 4086.84 | 2.42 | 2.95 | 3856.78 | 85.13 |
| 319739 | NorCOMM2 | (null) | C57BL/6NCrl | +/+ | F | 319739 | 9/17/2015 | 1800.96 | 1540.22 | 236.31 | 118.96 | 899.25 | 23.77 | 34.33 | 3318.33 | 1343.65 | 3479.57 | 714.20 | 153.67 | 4409.46 | 374.01 | 24.70 | 81.90 | 55.94 | 21.32 | 151.67 | 45.57 | 77.70 | 2.83 | 85.85 | 1699.85 | 161.56 | 399.79 | 19.84 | 106.32 | 484.77 | 221.13 | 360.71 | 695.34 | 2.23 | 174.52 | 5.79 | 8.98 | 5479.78 | 5.70 | 379.75 | 22.53 | 48.02 | 11.95 | 3.89 | 49.50 | 18.48 | 15.48 | 226.12 | 4184.93 | 2.71 | 2.98 | 2993.95 | 96.33 |
| 198415 | NorCOMM2 | (null) | C57BL/6NCrl | +/+ | F | 198415 | 12/23/2014 | 1418.76 | 1513.08 | 186.59 | 84.10 | 812.76 | 17.27 | 18.57 | 2297.83 | 571.86 | 2017.17 | 905.33 | 97.11 | 2925.18 | 144.06 | 16.98 | 100.78 | 32.28 | 25.84 | 120.19 | 21.88 | 87.58 | 2.36 | 77.22 | 1588.66 | 139.11 | 338.53 | 14.31 | 56.93 | 423.89 | 178.96 | 349.32 | 523.89 | 2.06 | 174.01 | 6.50 | 0.00 | 4924.01 | 3.89 | 251.47 | 17.27 | 49.23 | 5.64 | 2.53 | 60.18 | 15.45 | 14.91 | 198.56 | 3435.29 | 2.85 | 4.16 | 4101.89 | 97.82 |
| 296903 | NorCOMM2 | (null) | C57BL/6NCrl | +/+ | F | 296903 | 7/21/2015 | 4330.54 | 942.68 | 201.76 | 105.75 | 962.86 | 21.99 | 63.34 | 1656.26 | 1866.56 | 4671.98 | 1655.08 | 182.22 | 6820.11 | 349.17 | 40.98 | 112.36 | 53.06 | 34.93 | 120.45 | 41.62 | 104.72 | 3.59 | 61.86 | 2194.53 | 243.55 | 396.56 | 12.98 | 59.14 | 489.64 | 197.63 | 450.86 | 698.47 | 2.82 | 191.61 | 6.35 | 3.76 | 4102.19 | 4.80 | 125.27 | 29.39 | 71.55 | 5.18 | 2.44 | 58.83 | 11.23 | 10.38 | 184.65 | 3675.36 | 2.67 | 4.26 | 2899.14 | 112.22 |
| 38540 | NorCOMM2 | (null) | C57BL/6NCrl | +/+ | F | 38540 | 12/11/2013 | 9356.34 | 1809.13 | 184.15 | 103.78 | 812.74 | 19.19 | 23.54 | 1561.32 | 8561.37 | 5447.11 | 1974.89 | 131.59 | 8455.12 | 541.27 | 44.03 | 182.28 | 100.23 | 56.21 | 214.72 | 105.85 | 105.30 | 3.54 | 568.05 | 6268.61 | 377.20 | 326.88 | 19.91 | 89.80 | 469.31 | 174.72 | 284.03 | 651.49 | 2.17 | 165.46 | 7.96 | 0.00 | 3882.17 | 5.07 | 245.51 | 22.35 | 54.55 | 8.07 | 3.90 | 70.50 | 17.98 | 16.80 | 200.48 | 4502.63 | 2.65 | 5.36 | 2633.39 | 82.31 |
| 349028 | NorCOMM2 | (null) | C57BL/6NCrl | +/+ | F | 349028 | 11/5/2015 | 8012.10 | 1773.20 | 224.10 | 80.35 | 748.82 | 15.63 | 25.76 | 4414.10 | 2471.00 | 4749.10 | 1648.90 | 365.76 | 6327.60 | 470.27 | 34.25 | 137.71 | 112.18 | 133.04 | 149.37 | 41.43 | 497.16 | 2.71 | 119.07 | 3003.20 | 208.51 | 300.87 | 18.46 | 64.64 | 424.33 | 177.08 | 419.67 | 553.12 | 2.47 | 200.75 | 6.75 | 0.00 | 3609.30 | 3.99 | 201.84 | 32.98 | 67.58 | 9.41 | 2.99 | 51.72 | 14.80 | 12.97 | 198.19 | 3987.60 | 2.58 | 3.58 | 2951.60 | 83.19 |
| 38537 | NorCOMM2 | (null) | C57BL/6NCrl | +/+ | F | 38537 | 12/11/2013 | 4413.80 | 2100.20 | 273.34 | 81.43 | 893.33 | 19.03 | 26.52 | 2712.20 | 1784.30 | 4179.90 | 896.89 | 210.30 | 5252.70 | 264.72 | 32.44 | 76.59 | 38.95 | 9.06 | 105.99 | 21.99 | 56.04 | 1.64 | 89.35 | 2163.90 | 194.58 | 308.82 | 21.44 | 89.22 | 480.76 | 180.74 | 374.39 | 555.39 | 2.10 | 185.64 | 7.49 | 1.70 | 4881.90 | 5.44 | 243.55 | 25.90 | 74.61 | 12.31 | 3.40 | 64.45 | 17.12 | 16.35 | 170.47 | 4846.80 | 3.96 | 4.56 | 3290.60 | 76.42 |
| 55856 | NorCOMM2 | (null) | C57BL/6NCrl | +/+ | F | 55856 | 1/22/2014 | 10106.00 | 1389.30 | 250.24 | 80.15 | 858.06 | 17.87 | 45.89 | 3919.80 | 1542.90 | 3695.60 | 1115.10 | 272.58 | 4564.30 | 245.89 | 23.52 | 94.29 | 659.90 | 14.02 | 187.89 | 46.85 | 60.76 | 3.49 | 43.62 | 1568.70 | 134.00 | 315.09 | 14.96 | 70.83 | 493.73 | 188.23 | 375.18 | 437.60 | 2.88 | 172.78 | 8.71 | 0.00 | 4722.00 | 5.35 | 232.60 | 28.10 | 91.75 | 11.24 | 3.83 | 65.05 | 13.44 | 15.17 | 209.48 | 5112.00 | 3.64 | 6.23 | 2686.10 | 89.60 |
| 144712 | NorCOMM2 | (null) | C57BL/6NCrl | +/+ | F | 144712 | 8/7/2014 | 6575.00 | 1576.70 | 189.05 | 98.15 | 714.04 | 16.96 | 16.90 | 3099.40 | 4572.40 | 9503.80 | 1684.50 | 126.36 | 9261.20 | 1006.40 | 19.96 | 71.52 | 27.10 | 13.45 | 6899.80 | 53.48 | 89.46 | 4.03 | 74.44 | 3858.80 | 309.97 | 373.67 | 18.63 | 61.19 | 434.03 | 211.86 | 415.37 | 581.55 | 2.59 | 191.13 | 6.39 | 1.27 | 4594.30 | 5.81 | 260.46 | 27.91 | 69.65 | 9.79 | 3.09 | 60.41 | 12.41 | 16.04 | 198.41 | 5087.20 | 2.89 | 4.75 | 2416.30 | 90.30 |
| 257642 | NorCOMM2 | (null) | C57BL/6NCrl | +/+ | F | 257642 | 5/21/2015 | 3623.30 | 1240.10 | 242.11 | 101.65 | 741.73 | 16.55 | 20.85 | 2337.80 | 810.57 | 3838.10 | 1150.40 | 328.80 | 4697.40 | 223.78 | 15.21 | 52.13 | 59.87 | 17.88 | 57.68 | 66.65 | 56.34 | 2.92 | 76.86 | 2726.30 | 246.43 | 290.71 | 14.25 | 80.24 | 448.40 | 158.43 | 271.48 | 565.41 | 2.35 | 200.32 | 6.61 | 1.90 | 4820.60 | 6.04 | 119.84 | 27.96 | 84.74 | 8.11 | 3.25 | 47.76 | 14.80 | 14.27 | 183.02 | 4010.40 | 2.98 | 6.26 | 1930.40 | 103.60 |
| 209240 | NorCOMM2 | (null) | C57BL/6NCrl | +/+ | F | 209240 | 1/13/2015 | 1004.30 | 2236.00 | 202.34 | 91.51 | 831.52 | 19.29 | 20.25 | 852.69 | 988.74 | 2707.30 | 1447.80 | 143.37 | 3088.40 | 215.17 | 32.92 | 55.82 | 69.78 | 7.51 | 86.69 | 46.70 | 54.52 | 2.42 | 60.68 | 1426.20 | 121.32 | 295.16 | 11.49 | 59.64 | 438.29 | 176.72 | 389.87 | 507.41 | 2.91 | 194.49 | 13.54 | 0.00 | 4930.90 | 3.53 | 166.77 | 32.58 | 77.34 | 10.17 | 2.91 | 91.78 | 12.57 | 14.72 | 151.96 | 3806.90 | 3.01 | 5.20 | 2344.20 | 107.68 |
| 410682 | KOMP2 | (null) | C57BL/6NCrl | +/+ | M | 410682 | 6/16/2016 | 2860.43 | 3128.13 | 289.96 | 115.20 | 1256.90 | 28.17 | 21.77 | 2607.98 | 2308.81 | 699.72 | 476.16 | 139.94 | 2455.62 | 251.64 | 10.98 | 90.49 | 123.83 | 6.29 | 55.62 | 30.89 | 29.36 | 1.52 | 43.13 | 1739.35 | 177.59 | 430.11 | 18.82 | 68.62 | 392.32 | 268.24 | 509.81 | 415.10 | 1.38 | 315.32 | 6.69 | 0.00 | 5539.67 | 4.19 | 350.77 | 21.54 | 33.49 | 9.58 | 2.62 | 52.24 | 18.69 | 15.98 | 281.39 | 2887.74 | 3.61 | 3.80 | 9784.80 | 136.68 |
| 307708 | NorCOMM2 | (null) | C57BL/6NCrl | +/+ | M | 307708 | 8/18/2015 | 2468.06 | 1313.30 | 293.32 | 92.22 | 981.41 | 24.65 | 47.54 | 2005.63 | 346.27 | 789.39 | 1239.93 | 97.59 | 2695.69 | 121.40 | 11.95 | 55.78 | 36.76 | 0.00 | 59.99 | 39.42 | 36.60 | 0.91 | 16.52 | 1737.09 | 163.42 | 416.63 | 18.17 | 70.07 | 341.60 | 266.92 | 355.61 | 498.42 | 1.73 | 331.29 | 6.10 | 8.84 | 4879.05 | 5.16 | 256.27 | 23.32 | 32.57 | 10.99 | 3.03 | 60.71 | 18.70 | 11.60 | 307.24 | 2985.96 | 3.91 | 2.13 | 7216.78 | 132.97 |
| 441143 | KOMP2 | (null) | C57BL/6NCrl | +/+ | M | 441143 | 9/22/2016 | 4633.53 | 2245.91 | 308.77 | 81.81 | 922.42 | 24.16 | 20.61 | 2098.24 | 13650.61 | 3185.63 | 1448.97 | 205.94 | 11752.61 | 1374.13 | 26.31 | 291.05 | 102.02 | 56.33 | 120.66 | 89.38 | 78.71 | 2.37 | 103.53 | 6922.14 | 562.86 | 382.06 | 11.94 | 65.35 | 411.45 | 334.37 | 453.85 | 461.63 | 1.31 | 345.09 | 5.40 | 0.78 | 4858.65 | 4.04 | 175.51 | 25.41 | 29.43 | 7.67 | 2.90 | 57.81 | 14.56 | 13.77 | 282.09 | 3071.00 | 3.48 | 3.57 | 7420.52 | 139.20 |
| 237609 | NorCOMM2 | (null) | C57BL/6NCrl | +/+ | M | 237609 | 4/23/2015 | 9371.75 | 1561.68 | 302.01 | 71.78 | 778.95 | 28.66 | 94.06 | 1769.73 | 959.28 | 1479.48 | 499.79 | 94.70 | 2404.78 | 207.28 | 29.80 | 62.23 | 33.21 | 10.21 | 64.73 | 27.42 | 104.17 | 1.21 | 22.49 | 1385.77 | 252.52 | 412.85 | 14.17 | 70.25 | 378.02 | 232.08 | 373.80 | 438.44 | 1.42 | 335.71 | 6.41 | 0.00 | 4150.04 | 4.46 | 351.27 | 22.29 | 33.91 | 10.52 | 3.54 | 42.84 | 12.18 | 10.29 | 237.32 | 2197.64 | 5.73 | 3.42 | 8995.82 | 121.84 |
| 510072 | K2P2 | (null) | C57BL/6NCrl | +/+ | M | 510072 | 4/19/2017 | 1446.58 | 1332.76 | 216.74 | 89.10 | 1235.42 | 24.98 | 26.07 | 2144.94 | 451.20 | 897.21 | 942.66 | 235.81 | 2674.72 | 153.00 | 15.62 | 55.47 | 47.47 | 16.70 | 69.56 | 25.02 | 39.33 | 1.68 | 44.21 | 2014.75 | 187.19 | 499.92 | 15.62 | 76.04 | 389.64 | 261.57 | 384.21 | 546.37 | 2.42 | 365.46 | 5.50 | 2.97 | 4248.82 | 3.64 | 262.15 | 25.99 | 55.12 | 9.12 | 3.52 | 57.15 | 15.46 | 13.47 | 357.22 | 3054.98 | 3.94 | 3.62 | 8158.36 | 156.13 |
| 198312 | NorCOMM2 | (null) | C57BL/6NCrl | +/+ | M | 198312 | 12/23/2014 | 8839.37 | 1187.08 | 244.20 | 94.33 | 990.12 | 18.24 | 116.64 | 1534.01 | 1373.04 | 1534.98 | 647.97 | 75.26 | 2700.48 | 256.34 | 18.50 | 105.19 | 31.56 | 0.00 | 62.33 | 33.92 | 34.99 | 1.13 | 46.24 | 1638.14 | 299.32 | 391.46 | 12.75 | 64.29 | 430.28 | 264.04 | 390.83 | 399.01 | 2.23 | 337.22 | 6.34 | 0.00 | 3819.15 | 3.33 | 214.46 | 25.86 | 52.76 | 7.85 | 2.33 | 49.13 | 12.70 | 15.66 | 249.82 | 2592.81 | 4.03 | 3.34 | 6995.58 | 133.06 |
| 483043 | K2P2 | (null) | C57BL/6NCrl | +/+ | M | 483043 | 12/29/2016 | 861.52 | 1410.42 | 178.27 | 97.30 | 700.00 | 17.65 | 16.33 | 2124.24 | 1154.41 | 539.90 | 497.82 | 128.59 | 2511.45 | 159.17 | 13.99 | 56.95 | 75.63 | 26.27 | 46.80 | 26.89 | 30.44 | 1.62 | 23.84 | 2738.43 | 217.67 | 311.17 | 14.70 | 54.75 | 243.37 | 197.49 | 270.75 | 400.93 | 1.87 | 256.82 | 4.12 | 4.39 | 3562.84 | 3.79 | 172.78 | 19.60 | 45.42 | 8.13 | 2.28 | 38.45 | 13.62 | 13.50 | 188.12 | 2515.73 | 4.28 | 3.22 | 6428.11 | 120.84 |
| 34418 | NorCOMM2 | (null) | C57BL/6NCrl | +/+ | M | 34418 | 11/20/2013 | 15654.60 | 3170.64 | 251.76 | 78.80 | 1087.55 | 28.05 | 25.21 | 2254.01 | 2368.41 | 1664.18 | 910.22 | 209.08 | 3244.36 | 254.90 | 19.12 | 73.19 | 74.80 | 12.09 | 131.43 | 37.78 | 42.41 | 3.27 | 33.64 | 1404.40 | 127.65 | 404.28 | 11.13 | 61.05 | 535.37 | 136.32 | 504.22 | 428.88 | 1.67 | 273.77 | 6.07 | 0.00 | 4712.82 | 5.96 | 441.27 | 22.50 | 44.93 | 6.99 | 2.81 | 48.72 | 17.79 | 15.69 | 307.73 | 3105.36 | 3.20 | 3.06 | 6576.05 | 147.53 |
| 257539 | NorCOMM2 | (null) | C57BL/6NCrl | +/+ | M | 257539 | 5/20/2015 | 978.49 | 1566.29 | 236.72 | 86.79 | 993.04 | 20.88 | 19.37 | 1528.17 | 12998.69 | 3463.30 | 237.77 | 105.74 | 11162.38 | 564.94 | 31.11 | 107.35 | 151.77 | 26.50 | 160.26 | 74.48 | 118.04 | 6.56 | 1016.81 | 10346.88 | 834.64 | 440.55 | 13.09 | 59.11 | 489.83 | 194.17 | 385.73 | 485.63 | 1.98 | 324.61 | 6.04 | 0.00 | 4912.18 | 4.42 | 193.05 | 30.25 | 42.33 | 9.49 | 2.76 | 45.83 | 12.56 | 12.30 | 282.20 | 3404.79 | 3.73 | 3.47 | 6524.60 | 131.53 |
| 348925 | NorCOMM2 | (null) | C57BL/6NCrl | +/+ | M | 348925 | 11/3/2015 | 639.06 | 2633.66 | 238.60 | 68.00 | 882.55 | 24.41 | 40.59 | 1230.70 | 3406.20 | 2147.75 | 986.78 | 174.45 | 4559.37 | 381.12 | 28.14 | 126.84 | 100.52 | 30.07 | 135.38 | 42.26 | 68.12 | 1.41 | 50.36 | 2939.60 | 240.73 | 327.58 | 12.25 | 60.68 | 424.60 | 183.31 | 512.02 | 592.02 | 1.87 | 269.38 | 6.42 | 0.00 | 4359.52 | 4.53 | 334.68 | 23.82 | 68.23 | 5.39 | 2.42 | 55.60 | 13.60 | 12.39 | 222.95 | 3380.70 | 3.09 | 6.70 | 4711.82 | 184.35 |
| 91833 | NorCOMM2 | (null) | C57BL/6NCrl | +/+ | M | 91833 | 4/29/2014 | 2008.23 | 2124.78 | 250.08 | 91.39 | 1112.61 | 25.50 | 41.92 | 2165.43 | 1223.35 | 2061.81 | 882.51 | 128.72 | 3759.10 | 281.77 | 22.57 | 82.99 | 53.18 | 10.56 | 74.03 | 40.02 | 40.93 | 3.14 | 31.33 | 1823.00 | 217.93 | 456.25 | 16.79 | 73.48 | 566.75 | 219.65 | 636.24 | 464.35 | 2.26 | 299.92 | 8.15 | 0.00 | 4575.43 | 5.04 | 304.67 | 23.57 | 51.70 | 9.87 | 2.95 | 57.24 | 17.75 | 16.18 | 313.30 | 2684.98 | 3.65 | 3.57 | 8560.30 | 152.86 |
| 365958 | NorCOMM2 | (null) | C57BL/6NCrl | +/+ | M | 365958 | 12/17/2015 | 3362.13 | 2250.81 | 345.39 | 116.57 | 959.28 | 22.26 | 43.88 | 4366.26 | 8785.92 | 2941.29 | 703.51 | 330.13 | 7126.55 | 397.12 | 113.88 | 181.14 | 71.85 | 44.05 | 359.03 | 263.36 | 205.14 | 18.48 | 54.51 | 3386.39 | 257.60 | 481.28 | 17.53 | 61.95 | 424.52 | 183.59 | 436.67 | 426.02 | 1.86 | 319.55 | 6.08 | 2.68 | 4388.60 | 4.63 | 274.48 | 26.55 | 54.60 | 12.52 | 2.67 | 59.49 | 16.25 | 18.01 | 314.42 | 2728.77 | 4.00 | 6.20 | 8690.17 | 162.08 |
| 209136 | NorCOMM2 | (null) | C57BL/6NCrl | +/+ | M | 209136 | 1/13/2015 | 1918.31 | 1296.43 | 211.17 | 87.48 | 989.59 | 23.43 | 27.46 | 1615.81 | 4728.17 | 2521.38 | 1634.76 | 295.35 | 6372.14 | 570.94 | 20.01 | 110.34 | 174.26 | 22.48 | 118.53 | 29.35 | 56.89 | 15.57 | 241.91 | 6041.66 | 470.56 | 416.08 | 12.75 | 61.42 | 410.35 | 200.24 | 385.60 | 426.76 | 2.07 | 347.83 | 6.66 | 0.00 | 4246.40 | 4.52 | 180.87 | 27.68 | 62.90 | 6.86 | 3.37 | 55.94 | 15.18 | 13.94 | 332.77 | 2821.89 | 3.06 | 4.42 | 7145.42 | 151.40 |
| 229586 | NorCOMM2 | (null) | C57BL/6NCrl | +/+ | M | 229586 | 3/24/2015 | 3684.70 | 1327.20 | 288.83 | 87.81 | 855.70 | 20.41 | 109.55 | 2634.00 | 241.52 | 815.05 | 744.19 | 144.72 | 2429.10 | 225.35 | 21.82 | 63.44 | 173.39 | 8.07 | 55.65 | 9.40 | 38.35 | 2.04 | 28.24 | 1185.00 | 306.99 | 469.87 | 13.37 | 59.37 | 388.78 | 214.74 | 509.90 | 531.88 | 2.49 | 348.39 | 6.98 | 0.40 | 5320.60 | 6.65 | 151.51 | 24.66 | 47.94 | 9.90 | 2.75 | 54.74 | 15.11 | 13.60 | 278.85 | 2455.60 | 3.86 | 3.51 | 6328.90 | 130.49 |
| 187594 | NorCOMM2 | (null) | C57BL/6NCrl | +/+ | M | 187594 | 11/25/2014 | 1918.40 | 892.21 | 261.35 | 107.40 | 733.05 | 17.35 | 116.43 | 2632.20 | 301.93 | 1232.60 | 746.99 | 87.08 | 2643.30 | 157.15 | 33.02 | 83.62 | 25.00 | 15.87 | 103.84 | 19.53 | 54.88 | 2.96 | 55.62 | 1401.90 | 327.07 | 379.42 | 17.41 | 47.65 | 335.83 | 192.38 | 256.09 | 397.11 | 1.74 | 296.99 | 4.97 | 0.00 | 4260.40 | 4.81 | 118.29 | 19.83 | 53.56 | 9.03 | 2.31 | 46.07 | 14.98 | 14.53 | 253.42 | 2432.20 | 3.66 | 3.92 | 5437.30 | 120.22 |
| 281969 | NorCOMM2 | (null) | C57BL/6NCrl | +/+ | M | 281969 | 6/23/2015 | 921.95 | 968.34 | 329.47 | 118.22 | 1045.90 | 25.16 | 56.02 | 1019.90 | 1525.50 | 1009.70 | 644.60 | 90.13 | 2088.80 | 102.78 | 47.36 | 56.65 | 46.93 | 0.00 | 96.39 | 25.20 | 27.43 | 1.64 | 43.75 | 1246.40 | 151.43 | 440.55 | 22.17 | 61.70 | 470.36 | 208.01 | 443.53 | 531.63 | 2.29 | 363.22 | 5.62 | 0.00 | 4526.50 | 5.00 | 275.19 | 30.16 | 61.59 | 12.46 | 2.85 | 43.65 | 16.06 | 14.17 | 467.47 | 3457.20 | 3.51 | 4.87 | 7901.50 | 135.55 |
| 115756 | NorCOMM2 | (null) | C57BL/6NCrl | +/+ | M | 115756 | 6/5/2014 | 10921.00 | 1970.90 | 345.94 | 80.08 | 715.58 | 17.86 | 53.51 | 1479.90 | 1102.30 | 1186.50 | 614.22 | 178.94 | 2634.80 | 149.63 | 26.42 | 62.02 | 41.79 | 11.66 | 126.43 | 27.50 | 41.91 | 1.18 | 40.52 | 1954.10 | 191.29 | 343.27 | 12.65 | 51.96 | 365.35 | 204.28 | 603.90 | 684.95 | 2.18 | 229.86 | 6.49 | 0.00 | 5155.90 | 5.74 | 344.33 | 28.34 | 53.39 | 5.76 | 2.36 | 49.61 | 10.75 | 12.73 | 182.38 | 3576.50 | 3.72 | 6.07 | 4589.60 | 141.57 |
| 38433 | NorCOMM2 | (null) | C57BL/6NCrl | +/+ | M | 38433 | 12/11/2013 | 7451.40 | 1714.80 | 333.71 | 72.30 | 986.51 | 21.46 | 39.85 | 2163.00 | 7744.60 | 2747.20 | 852.94 | 114.85 | 6982.40 | 532.78 | 23.90 | 124.60 | 85.87 | 39.20 | 208.30 | 59.37 | 165.23 | 5.16 | 104.19 | 6533.10 | 495.21 | 443.80 | 16.99 | 66.48 | 411.81 | 199.64 | 549.40 | 488.75 | 2.09 | 304.21 | 6.93 | 0.75 | 4734.80 | 4.67 | 304.53 | 22.96 | 59.94 | 10.03 | 3.20 | 60.67 | 11.78 | 14.82 | 276.39 | 2814.80 | 4.17 | 5.75 | 8925.40 | 118.98 |
| 144609 | NorCOMM2 | (null) | C57BL/6NCrl | +/+ | M | 144609 | 8/7/2014 | 16592.00 | 1233.80 | 332.54 | 81.06 | 1094.60 | 20.44 | 20.08 | 2595.60 | 2858.20 | 2569.50 | 1121.50 | 119.04 | 5308.80 | 287.56 | 48.96 | 77.72 | 54.75 | 35.12 | 89.19 | 47.17 | 147.35 | 1.72 | 35.31 | 3059.70 | 230.44 | 438.69 | 19.71 | 70.94 | 396.59 | 240.02 | 398.91 | 480.20 | 2.13 | 379.65 | 6.44 | 0.00 | 4751.40 | 4.73 | 169.05 | 28.03 | 55.90 | 7.92 | 2.20 | 52.88 | 15.95 | 18.42 | 253.22 | 4723.50 | 3.90 | 4.66 | 6040.90 | 143.03 |
| 204873 | NorCOMM2 | A2m | A2m_tm1b_C10 | -/- | F | 204873 | 12/23/2014 | 2162.40 | 1796.00 | 219.84 | 74.42 | 798.47 | 19.68 | 24.41 | 2160.70 | 3793.30 | 3915.00 | 1247.50 | 210.33 | 5678.60 | 379.32 | 27.61 | 126.37 | 39.68 | 16.50 | 187.23 | 72.71 | 167.21 | 2.26 | 132.09 | 3718.60 | 259.01 | 284.85 | 15.35 | 76.39 | 409.24 | 191.56 | 391.77 | 469.34 | 1.78 | 216.92 | 7.28 | 0.00 | 4291.10 | 6.53 | 221.35 | 25.43 | 81.30 | 8.67 | 3.38 | 51.96 | 14.49 | 12.01 | 193.42 | 3909.70 | 2.86 | 4.01 | 3493.60 | 91.60 |
| 204868 | NorCOMM2 | A2m | A2m_tm1b_C10 | -/- | F | 204868 | 12/23/2014 | 2463.90 | 1811.90 | 304.24 | 104.27 | 823.99 | 21.74 | 31.36 | 844.25 | 1961.50 | 4812.20 | 2357.60 | 210.04 | 6607.40 | 314.99 | 37.21 | 109.62 | 28.84 | 29.05 | 121.08 | 42.91 | 165.85 | 4.49 | 89.91 | 2773.00 | 249.97 | 350.83 | 18.38 | 50.43 | 460.43 | 217.55 | 331.63 | 489.58 | 2.66 | 174.83 | 7.76 | 0.00 | 5891.00 | 5.37 | 194.80 | 32.26 | 63.76 | 11.34 | 3.55 | 50.89 | 16.61 | 16.40 | 202.74 | 4550.10 | 2.72 | 2.51 | 2762.30 | 90.26 |
| 204872 | NorCOMM2 | A2m | A2m_tm1b_C10 | -/- | F | 204872 | 12/23/2014 | 2226.40 | 2028.50 | 215.66 | 75.78 | 744.38 | 17.16 | 28.66 | 1173.00 | 9285.40 | 16871.00 | 1669.80 | 323.62 | 16783.00 | 1226.40 | 36.33 | 243.80 | 195.18 | 141.95 | 264.46 | 269.14 | 433.28 | 8.55 | 885.40 | 7732.40 | 614.78 | 304.75 | 19.20 | 86.05 | 448.07 | 180.37 | 370.65 | 423.64 | 2.36 | 248.44 | 7.09 | 0.00 | 4374.70 | 5.27 | 233.56 | 31.20 | 73.60 | 9.08 | 2.57 | 53.45 | 12.24 | 15.18 | 213.72 | 3814.10 | 2.39 | 4.10 | 4096.70 | 71.18 |
| 197966 | NorCOMM2 | A2m | A2m_tm1b_C10 | -/- | M | 197966 | 12/18/2014 | 3250.10 | 1624.20 | 332.48 | 94.03 | 908.79 | 21.79 | 24.50 | 1453.70 | 2163.10 | 2253.80 | 1308.90 | 307.45 | 4426.20 | 150.15 | 24.14 | 87.63 | 51.94 | 11.39 | 694.78 | 21.08 | 50.31 | 2.66 | 18.46 | 3262.80 | 272.82 | 443.61 | 13.51 | 65.63 | 410.11 | 204.67 | 475.58 | 521.07 | 2.29 | 322.23 | 6.14 | 0.00 | 4482.90 | 5.65 | 310.12 | 26.65 | 50.70 | 10.62 | 2.71 | 52.61 | 12.05 | 18.78 | 266.72 | 3215.50 | 3.59 | 3.49 | 9235.00 | 119.26 |
| 197962 | NorCOMM2 | A2m | A2m_tm1b_C10 | -/- | M | 197962 | 12/18/2014 | 4459.50 | 2079.70 | 359.40 | 101.21 | 1108.20 | 24.20 | 30.84 | 1222.30 | 4084.20 | 2854.30 | 876.22 | 833.99 | 4393.40 | 272.84 | 31.60 | 142.12 | 136.06 | 8.72 | 136.12 | 17.07 | 77.07 | 2.40 | 18.09 | 3245.00 | 222.25 | 467.52 | 21.82 | 74.09 | 480.54 | 220.38 | 521.09 | 513.22 | 2.20 | 408.15 | 6.96 | 0.00 | 5170.80 | 5.95 | 367.89 | 23.95 | 66.04 | 8.81 | 3.01 | 46.71 | 20.00 | 17.55 | 330.68 | 3593.00 | 3.95 | 5.68 | 7590.20 | 144.99 |
| 197964 | NorCOMM2 | A2m | A2m_tm1b_C10 | -/- | M | 197964 | 12/18/2014 | 1555.20 | 1378.80 | 277.28 | 105.26 | 1106.90 | 19.87 | 25.43 | 3922.40 | 2445.50 | 2400.60 | 1451.00 | 384.40 | 4450.30 | 342.93 | 22.84 | 214.14 | 45.83 | 27.92 | 113.22 | 32.17 | 185.90 | 3.41 | 67.13 | 3155.40 | 266.80 | 484.64 | 17.50 | 48.92 | 351.56 | 220.02 | 420.32 | 549.36 | 2.58 | 382.78 | 7.61 | 4.60 | 5108.00 | 7.60 | 223.81 | 22.05 | 75.16 | 6.89 | 3.05 | 59.07 | 13.52 | 16.23 | 279.41 | 3187.90 | 4.50 | 4.32 | 8074.30 | 117.38 |
| 181060 | NorCOMM2 | Ahcy | Ahcy_tm1b_E07 | +/- | F | 181060 | 11/6/2014 | 1435.90 | 2228.50 | 261.19 | 97.74 | 826.92 | 17.27 | 27.71 | 1356.90 | 4001.70 | 5666.50 | 1101.70 | 1335.60 | 6549.30 | 248.67 | 38.78 | 138.66 | 156.26 | 114.76 | 63.05 | 21.40 | 101.33 | 85.67 | 55.12 | 5404.80 | 376.77 | 364.79 | 19.06 | 89.16 | 446.77 | 213.30 | 364.73 | 662.33 | 2.31 | 204.65 | 7.43 | 0.00 | 5624.50 | 4.00 | 271.25 | 21.95 | 54.38 | 9.25 | 4.16 | 44.51 | 13.49 | 18.76 | 244.22 | 5386.40 | 2.31 | 5.53 | 3169.10 | 93.87 |
| 179053 | NorCOMM2 | Ahcy | Ahcy_tm1b_E07 | +/- | F | 179053 | 10/23/2014 | 1215.40 | 1617.40 | 248.05 | 79.17 | 865.78 | 22.21 | 49.63 | 2724.10 | 2015.30 | 3091.30 | 621.44 | 263.23 | 5026.00 | 276.32 | 53.43 | 143.14 | 47.82 | 47.24 | 162.57 | 56.27 | 89.33 | 10.13 | 31.89 | 2156.90 | 193.43 | 293.48 | 25.29 | 102.09 | 465.87 | 200.11 | 332.21 | 564.49 | 2.62 | 209.22 | 5.93 | 0.00 | 5071.50 | 4.24 | 265.11 | 24.15 | 62.57 | 13.28 | 5.21 | 44.15 | 11.93 | 15.09 | 237.55 | 5293.70 | 2.18 | 4.32 | 3658.30 | 89.13 |
| 181059 | NorCOMM2 | Ahcy | Ahcy_tm1b_E07 | +/- | F | 181059 | 11/6/2014 | 2353.30 | 1886.70 | 262.75 | 108.88 | 1001.80 | 24.42 | 53.09 | 2881.00 | 4227.20 | 5171.60 | 1811.00 | 302.60 | 8014.50 | 376.78 | 39.52 | 297.99 | 125.91 | 35.22 | 234.78 | 68.85 | 100.44 | 2.62 | 71.70 | 5830.30 | 452.77 | 356.71 | 19.53 | 76.85 | 506.98 | 183.40 | 354.92 | 511.77 | 2.28 | 215.03 | 7.67 | 0.41 | 5366.10 | 6.35 | 235.68 | 26.62 | 89.47 | 9.59 | 3.84 | 63.26 | 14.57 | 17.90 | 205.16 | 4772.50 | 3.23 | 6.28 | 3390.50 | 96.32 |
| 178951 | NorCOMM2 | Ahcy | Ahcy_tm1b_E07 | +/- | M | 178951 | 10/23/2014 | 4996.70 | 2901.50 | 381.11 | 77.87 | 995.89 | 18.69 | 77.08 | 2451.90 | 3228.60 | 4597.90 | 539.42 | 435.07 | 5780.00 | 244.55 | 16.54 | 275.21 | 24.50 | 12.59 | 76.24 | 57.93 | 145.56 | 3.18 | 177.25 | 1929.10 | 216.85 | 501.23 | 17.77 | 61.76 | 411.25 | 203.50 | 502.45 | 416.00 | 2.19 | 334.74 | 6.38 | 0.00 | 4969.90 | 5.46 | 348.16 | 21.96 | 65.20 | 15.58 | 2.20 | 56.95 | 19.32 | 19.18 | 266.39 | 2460.50 | 4.73 | 6.34 | 8622.40 | 119.55 |
| 178948 | NorCOMM2 | Ahcy | Ahcy_tm1b_E07 | +/- | M | 178948 | 10/23/2014 | 1894.90 | 2317.90 | 351.42 | 74.79 | 1028.60 | 18.90 | 73.58 | 1972.00 | 4004.40 | 4004.30 | 489.90 | 735.09 | 5895.90 | 427.46 | 22.97 | 191.18 | 38.44 | 15.25 | 153.23 | 54.29 | 80.04 | 5.72 | 22.27 | 1606.90 | 214.37 | 492.81 | 17.85 | 80.95 | 361.77 | 180.52 | 387.42 | 420.91 | 2.19 | 335.95 | 6.34 | 0.23 | 5387.90 | 5.00 | 319.83 | 27.98 | 62.65 | 8.79 | 2.68 | 52.86 | 15.11 | 17.41 | 266.89 | 2388.30 | 4.20 | 4.79 | 9071.10 | 124.76 |
| 178950 | NorCOMM2 | Ahcy | Ahcy_tm1b_E07 | +/- | M | 178950 | 10/23/2014 | 1292.10 | 1545.50 | 322.96 | 110.79 | 875.92 | 18.29 | 51.93 | 2494.00 | 1679.00 | 2408.80 | 974.17 | 250.34 | 4284.10 | 363.61 | 20.25 | 85.21 | 33.99 | 5.82 | 70.78 | 58.87 | 60.55 | 3.28 | 46.94 | 3276.70 | 269.96 | 383.24 | 18.67 | 66.71 | 391.31 | 237.82 | 488.96 | 509.36 | 2.17 | 440.42 | 5.50 | 0.00 | 5278.10 | 7.66 | 166.18 | 29.56 | 51.56 | 11.23 | 2.67 | 49.24 | 14.70 | 13.58 | 309.74 | 3032.30 | 4.10 | 3.63 | 7168.40 | 113.07 |
| 143963 | NorCOMM2 | Atp5b | Atp5b_tm1b_B07 | +/- | F | 143963 | 8/7/2014 | 3314.43 | 2117.97 | 234.73 | 73.68 | 873.69 | 22.60 | 33.51 | 2110.18 | 531.71 | 2176.96 | 991.84 | 218.29 | 3671.82 | 204.03 | 24.73 | 130.03 | 39.86 | 33.67 | 113.59 | 21.19 | 51.44 | 0.87 | 49.66 | 1551.44 | 119.29 | 348.76 | 16.85 | 127.53 | 477.69 | 281.89 | 345.63 | 537.81 | 1.44 | 188.98 | 5.91 | 3.05 | 4947.23 | 4.76 | 414.56 | 20.77 | 39.40 | 11.51 | 3.47 | 52.28 | 18.10 | 12.89 | 286.75 | 4944.59 | 2.85 | 3.51 | 3324.04 | 87.91 |
| 143961 | NorCOMM2 | Atp5b | Atp5b_tm1b_B07 | +/- | F | 143961 | 8/7/2014 | 7346.81 | 2018.71 | 253.79 | 86.07 | 781.17 | 22.37 | 25.01 | 1943.45 | 703.31 | 2947.33 | 1153.12 | 245.06 | 3839.70 | 216.40 | 14.85 | 74.30 | 38.93 | 40.25 | 209.25 | 10.53 | 40.36 | 1.39 | 29.43 | 2091.90 | 160.91 | 353.58 | 15.29 | 104.44 | 437.97 | 319.84 | 352.40 | 486.80 | 1.75 | 207.32 | 6.81 | 0.00 | 4996.18 | 5.69 | 412.40 | 20.93 | 49.19 | 9.60 | 4.19 | 56.66 | 14.44 | 14.98 | 251.79 | 4166.07 | 2.72 | 4.24 | 4039.29 | 97.29 |
| 143960 | NorCOMM2 | Atp5b | Atp5b_tm1b_B07 | +/- | F | 143960 | 8/7/2014 | 2851.01 | 1941.76 | 217.28 | 87.25 | 938.61 | 21.90 | 65.44 | 1915.76 | 713.10 | 4328.36 | 1126.78 | 194.93 | 4086.33 | 256.08 | 51.38 | 196.73 | 61.66 | 13.37 | 151.42 | 134.71 | 49.91 | 1.87 | 39.26 | 1327.73 | 182.28 | 369.23 | 15.48 | 113.41 | 440.19 | 433.35 | 391.20 | 649.53 | 1.79 | 166.48 | 5.88 | 0.00 | 5177.99 | 4.77 | 376.24 | 19.22 | 44.17 | 9.75 | 3.68 | 59.32 | 15.68 | 16.32 | 215.94 | 3869.85 | 4.02 | 3.00 | 4000.78 | 78.74 |
| 118347 | NorCOMM2 | Atp5b | Atp5b_tm1b_B07 | +/- | M | 118347 | 6/11/2014 | 7872.15 | 2694.12 | 277.24 | 80.98 | 1066.87 | 27.59 | 21.61 | 2169.00 | 2033.23 | 3224.81 | 692.14 | 335.42 | 4444.03 | 227.12 | 19.97 | 93.18 | 26.02 | 53.53 | 79.85 | 31.08 | 37.66 | 2.05 | 37.49 | 2212.92 | 234.78 | 446.60 | 16.29 | 73.38 | 551.68 | 377.75 | 595.68 | 481.07 | 1.45 | 271.28 | 6.38 | 1.64 | 4562.43 | 4.93 | 320.97 | 22.46 | 27.71 | 10.89 | 3.93 | 51.98 | 11.11 | 14.55 | 324.46 | 2671.71 | 4.02 | 4.06 | 8297.41 | 106.22 |
| 118346 | NorCOMM2 | Atp5b | Atp5b_tm1b_B07 | +/- | M | 118346 | 6/11/2014 | 1731.67 | 2069.40 | 279.09 | 79.38 | 992.47 | 24.65 | 24.20 | 3119.93 | 916.50 | 2035.63 | 1687.49 | 407.64 | 3894.55 | 382.10 | 38.13 | 128.80 | 48.51 | 2.73 | 95.94 | 32.44 | 153.94 | 1.04 | 35.30 | 2249.66 | 192.79 | 362.85 | 16.91 | 58.65 | 423.97 | 313.41 | 384.56 | 434.29 | 1.67 | 331.29 | 5.59 | 0.00 | 4632.35 | 5.62 | 190.24 | 18.89 | 53.56 | 8.22 | 3.04 | 55.76 | 14.54 | 11.93 | 277.93 | 2275.90 | 5.68 | 4.31 | 8512.45 | 108.37 |
| 143858 | NorCOMM2 | Atp5b | Atp5b_tm1b_B07 | +/- | M | 143858 | 8/7/2014 | 3676.98 | 5863.11 | 180.11 | 58.10 | 1159.01 | 31.06 | 825.65 | 11890.47 | 8370.16 | 10542.17 | 4180.26 | 1271.29 | 25987.18 | 1655.61 | 558.56 | 893.58 | 220.03 | 361.05 | 712.78 | 292.04 | 483.93 | 7.29 | 149.51 | 20414.21 | 3347.83 | 251.76 | 11.84 | 61.61 | 925.31 | 631.24 | 1763.18 | 767.02 | 1.73 | 100.80 | 10.41 | 0.00 | 6547.80 | 6.28 | 45.87 | 42.75 | 57.52 | 6.56 | 3.82 | 81.49 | 4.82 | 5.14 | 28.10 | 838.82 | 7.66 | 6.82 | 2413.47 | 142.30 |
| 281686 | NorCOMM2 | Atp6v0d1 | Atp6v0d1_tm1b_E01 | +/- | F | 281686 | 6/24/2015 | 748.19 | 1151.21 | 229.87 | 96.79 | 748.00 | 19.78 | 118.88 | 4632.64 | 1079.91 | 3885.35 | 1200.19 | 251.73 | 4975.03 | 271.88 | 52.79 | 183.28 | 40.07 | 19.34 | 87.24 | 111.76 | 62.36 | 3.26 | 35.10 | 857.85 | 252.28 | 287.64 | 13.42 | 74.14 | 473.26 | 172.99 | 337.78 | 601.47 | 2.47 | 201.04 | 6.96 | 0.00 | 4135.99 | 5.48 | 185.03 | 25.74 | 51.23 | 5.31 | 3.37 | 56.64 | 14.21 | 10.69 | 196.85 | 3920.49 | 2.67 | 4.04 | 2772.28 | 93.70 |
| 281679 | NorCOMM2 | Atp6v0d1 | Atp6v0d1_tm1b_E01 | +/- | F | 281679 | 6/24/2015 | 1050.87 | 2353.41 | 142.37 | 75.43 | 969.89 | 18.55 | 35.90 | 1840.34 | 910.97 | 2339.45 | 1059.78 | 383.95 | 3266.30 | 294.01 | 19.64 | 99.91 | 38.91 | 13.02 | 64.97 | 45.00 | 51.48 | 1.92 | 46.08 | 1476.82 | 142.84 | 287.71 | 14.71 | 83.33 | 435.46 | 177.67 | 352.53 | 511.82 | 2.21 | 137.00 | 6.14 | 0.00 | 3755.61 | 4.61 | 259.08 | 15.96 | 51.79 | 8.50 | 3.11 | 53.42 | 12.99 | 13.73 | 195.35 | 4151.76 | 2.57 | 3.97 | 3667.78 | 91.58 |
| 281678 | NorCOMM2 | Atp6v0d1 | Atp6v0d1_tm1b_E01 | +/- | F | 281678 | 6/24/2015 | 4649.73 | 1045.90 | 233.65 | 91.35 | 1153.30 | 22.82 | 48.27 | 1249.90 | 200.16 | 2052.25 | 1839.80 | 118.87 | 2597.51 | 132.28 | 12.63 | 49.26 | 23.98 | 8.15 | 58.09 | 17.39 | 91.55 | 1.39 | 19.51 | 748.97 | 79.57 | 297.40 | 8.63 | 41.65 | 603.57 | 207.46 | 463.56 | 387.49 | 2.62 | 237.80 | 7.59 | 0.00 | 3740.47 | 4.24 | 86.99 | 33.53 | 50.73 | 2.76 | 2.00 | 58.65 | 13.07 | 8.67 | 213.86 | 2383.63 | 3.60 | 4.76 | 2882.34 | 126.09 |
| 281575 | NorCOMM2 | Atp6v0d1 | Atp6v0d1_tm1b_E01 | +/- | M | 281575 | 6/23/2015 | 5673.28 | 1271.20 | 237.65 | 72.51 | 1029.05 | 20.62 | 70.81 | 2138.09 | 6964.81 | 3819.24 | 649.81 | 241.57 | 6431.05 | 350.50 | 28.39 | 133.18 | 63.84 | 18.80 | 143.11 | 28.09 | 33.09 | 3.16 | 67.44 | 1439.51 | 199.40 | 452.52 | 14.94 | 68.99 | 447.49 | 167.21 | 291.30 | 388.56 | 2.65 | 302.37 | 7.99 | 0.00 | 4649.34 | 6.39 | 190.40 | 27.65 | 63.76 | 10.06 | 4.12 | 65.37 | 14.02 | 12.38 | 346.10 | 2868.06 | 3.84 | 4.59 | 5985.83 | 127.05 |
| 281582 | NorCOMM2 | Atp6v0d1 | Atp6v0d1_tm1b_E01 | +/- | M | 281582 | 6/23/2015 | 2378.06 | 2726.30 | 305.18 | 66.54 | 1002.25 | 20.80 | 104.10 | 1661.13 | 3029.34 | 2481.72 | 664.37 | 164.81 | 3737.77 | 375.40 | 29.64 | 110.72 | 59.08 | 15.43 | 84.15 | 45.20 | 43.26 | 1.80 | 41.57 | 1326.50 | 235.69 | 455.03 | 15.80 | 66.17 | 373.00 | 173.12 | 406.44 | 456.52 | 2.11 | 260.83 | 6.74 | 0.00 | 4815.01 | 5.22 | 303.41 | 20.94 | 40.06 | 13.33 | 2.80 | 61.72 | 18.46 | 14.14 | 310.04 | 3216.93 | 5.21 | 5.26 | 7662.31 | 125.77 |
| 281584 | NorCOMM2 | Atp6v0d1 | Atp6v0d1_tm1b_E01 | +/- | M | 281584 | 6/23/2015 | 2695.62 | 2092.13 | 318.80 | 90.03 | 1231.68 | 25.28 | 85.78 | 3581.43 | 2348.19 | 3262.65 | 651.27 | 182.53 | 6472.15 | 358.66 | 22.19 | 128.03 | 27.52 | 19.78 | 99.97 | 20.13 | 28.39 | 1.46 | 41.32 | 2036.54 | 234.09 | 510.08 | 15.20 | 63.22 | 410.15 | 167.67 | 369.38 | 423.00 | 2.23 | 278.87 | 6.56 | 0.00 | 3985.16 | 6.18 | 326.01 | 24.29 | 56.38 | 10.94 | 3.17 | 58.73 | 18.70 | 13.98 | 264.98 | 3291.31 | 4.02 | 6.62 | 10757.81 | 141.19 |
| 222890 | NorCOMM2 | C8a | C8a_tm1b_F01 | -/- | F | 222890 | 2/18/2015 | 1775.90 | 1044.05 | 189.69 | 79.05 | 561.27 | 18.74 | 83.35 | 5871.96 | 4090.07 | 8782.89 | 763.68 | 214.35 | 9938.26 | 546.49 | 39.69 | 131.70 | 78.40 | 163.85 | 239.15 | 414.12 | 113.88 | 2.53 | 96.30 | 1904.36 | 274.33 | 315.82 | 11.81 | 61.29 | 437.12 | 122.74 | 334.18 | 461.36 | 2.78 | 173.80 | 5.17 | 0.00 | 4116.23 | 5.41 | 149.35 | 28.56 | 49.60 | 6.98 | 3.57 | 54.91 | 8.66 | 7.97 | 189.11 | 1627.20 | 2.19 | 4.92 | 3026.83 | 85.03 |
| 222888 | NorCOMM2 | C8a | C8a_tm1b_F01 | -/- | F | 222888 | 2/18/2015 | 799.98 | 2649.35 | 194.62 | 97.68 | 750.25 | 24.40 | 67.03 | 7088.34 | 6729.11 | 5578.61 | 1061.76 | 358.33 | 7846.07 | 349.70 | 35.25 | 114.69 | 41.99 | 35.32 | 366.58 | 116.37 | 176.31 | 2.56 | 126.95 | 2077.77 | 241.99 | 371.79 | 13.93 | 90.69 | 483.27 | 166.55 | 345.68 | 463.98 | 2.30 | 211.83 | 7.02 | 0.00 | 4914.39 | 6.17 | 138.11 | 27.76 | 49.85 | 7.86 | 2.28 | 52.02 | 21.33 | 16.01 | 223.18 | 3351.06 | 2.17 | 4.25 | 3879.39 | 88.52 |
| 222895 | NorCOMM2 | C8a | C8a_tm1b_F01 | -/- | F | 222895 | 2/18/2015 | 1100.79 | 1694.12 | 199.29 | 118.23 | 941.50 | 24.29 | 56.33 | 2560.37 | 1187.59 | 4165.02 | 1467.90 | 243.84 | 4915.19 | 309.26 | 25.36 | 139.72 | 55.07 | 20.67 | 114.56 | 69.06 | 142.30 | 1.20 | 102.82 | 1666.65 | 213.27 | 387.55 | 16.42 | 93.37 | 535.53 | 194.73 | 314.08 | 398.31 | 2.56 | 164.09 | 8.56 | 0.00 | 4856.62 | 5.53 | 224.15 | 19.45 | 45.85 | 6.14 | 4.32 | 54.75 | 17.76 | 12.26 | 204.17 | 3983.59 | 2.83 | 4.34 | 2660.56 | 103.73 |
| 233514 | NorCOMM2 | C8a | C8a_tm1b_F01 | -/- | M | 233514 | 4/8/2015 | 1887.62 | 1761.85 | 269.20 | 84.66 | 1392.76 | 27.49 | 113.19 | 2862.99 | 1694.14 | 3094.96 | 517.14 | 176.27 | 3964.29 | 180.04 | 36.37 | 99.18 | 35.47 | 31.47 | 137.64 | 38.87 | 44.88 | 2.30 | 28.96 | 1631.62 | 265.79 | 455.24 | 12.05 | 71.33 | 394.48 | 166.45 | 395.67 | 402.43 | 2.31 | 310.11 | 7.42 | 0.00 | 4672.80 | 5.57 | 273.64 | 30.06 | 40.62 | 9.14 | 3.43 | 66.01 | 19.62 | 15.03 | 319.83 | 2606.58 | 3.11 | 4.49 | 8523.06 | 142.99 |
| 233523 | NorCOMM2 | C8a | C8a_tm1b_F01 | -/- | M | 233523 | 4/8/2015 | 1044.12 | 2213.29 | 293.39 | 63.48 | 1335.00 | 26.47 | 145.39 | 1934.91 | 1348.03 | 2112.25 | 915.92 | 231.33 | 4121.44 | 278.09 | 14.35 | 57.20 | 104.23 | 3.21 | 81.28 | 26.96 | 63.65 | 1.42 | 41.89 | 2602.91 | 468.61 | 501.68 | 17.11 | 76.66 | 405.62 | 178.18 | 377.27 | 465.83 | 2.53 | 331.22 | 6.67 | 0.00 | 5009.76 | 5.55 | 352.74 | 30.38 | 66.73 | 9.34 | 3.50 | 51.26 | 16.77 | 16.57 | 335.67 | 2352.27 | 4.04 | 6.30 | 9384.32 | 132.29 |
| 233515 | NorCOMM2 | C8a | C8a_tm1b_F01 | -/- | M | 233515 | 4/8/2015 | 727.28 | 2704.68 | 281.68 | 103.01 | 1176.36 | 24.63 | 58.12 | 2217.21 | 1699.56 | 2348.50 | 815.34 | 224.57 | 4194.64 | 232.80 | 31.53 | 101.45 | 36.68 | 12.14 | 234.48 | 34.01 | 35.19 | 2.73 | 18.83 | 1883.84 | 230.19 | 502.86 | 16.21 | 71.25 | 461.85 | 181.38 | 436.67 | 466.47 | 2.04 | 339.89 | 7.85 | 3.90 | 5259.16 | 6.00 | 328.72 | 26.39 | 41.34 | 6.20 | 2.98 | 61.43 | 24.81 | 17.11 | 322.51 | 2720.35 | 3.42 | 3.59 | 9866.15 | 132.01 |
| 206829 | NorCOMM2 | Cdk4 | Cdk4_tm1b_F08 | +/- | F | 206829 | 1/8/2015 | 981.74 | 2198.77 | 219.15 | 100.64 | 974.63 | 18.15 | 33.74 | 1655.51 | 6193.79 | 4994.66 | 1142.43 | 295.46 | 7014.22 | 379.22 | 20.37 | 141.34 | 55.93 | 24.05 | 168.01 | 92.37 | 73.44 | 2.94 | 32.36 | 6467.19 | 477.09 | 373.03 | 19.88 | 93.81 | 573.52 | 213.31 | 467.58 | 597.73 | 2.25 | 185.92 | 8.07 | 0.00 | 5688.77 | 5.26 | 362.57 | 25.98 | 76.81 | 8.45 | 4.47 | 66.82 | 14.12 | 14.86 | 264.84 | 3764.08 | 2.94 | 4.82 | 4336.73 | 89.25 |
| 206828 | NorCOMM2 | Cdk4 | Cdk4_tm1b_F08 | +/- | F | 206828 | 1/6/2015 | 1606.07 | 2124.89 | 203.53 | 76.78 | 783.29 | 18.45 | 39.09 | 2504.66 | 2842.32 | 5202.12 | 3495.41 | 226.57 | 5952.34 | 197.42 | 32.35 | 150.97 | 48.52 | 19.98 | 154.80 | 36.68 | 65.49 | 2.52 | 43.06 | 2214.96 | 231.44 | 370.25 | 17.53 | 78.47 | 435.51 | 191.58 | 361.26 | 460.47 | 2.05 | 209.44 | 5.56 | 0.00 | 4056.46 | 5.54 | 196.60 | 19.46 | 58.69 | 7.37 | 3.29 | 59.02 | 17.44 | 14.42 | 223.01 | 3835.21 | 2.70 | 3.60 | 3572.76 | 88.69 |
| 206822 | NorCOMM2 | Cdk4 | Cdk4_tm1b_F08 | +/- | F | 206822 | 1/6/2015 | 2140.10 | 2011.47 | 214.48 | 72.74 | 932.35 | 22.96 | 24.89 | 1242.78 | 303.28 | 3044.01 | 1261.04 | 226.36 | 3821.01 | 221.66 | 12.57 | 81.37 | 27.35 | 12.25 | 152.28 | 15.43 | 37.04 | 2.29 | 23.57 | 2314.21 | 157.83 | 373.11 | 18.18 | 81.59 | 422.90 | 176.43 | 319.94 | 492.55 | 1.92 | 182.34 | 6.21 | 0.00 | 4846.49 | 4.15 | 227.73 | 22.01 | 68.95 | 5.82 | 3.76 | 60.76 | 13.74 | 16.08 | 207.95 | 4178.91 | 3.00 | 5.02 | 3958.74 | 107.63 |
| 190208 | NorCOMM2 | Cdk4 | Cdk4_tm1b_F08 | +/- | M | 190208 | 11/25/2014 | 4512.95 | 1939.40 | 229.45 | 72.11 | 1023.95 | 24.40 | 35.13 | 2051.01 | 5860.20 | 12611.49 | 537.81 | 261.70 | 11536.11 | 940.28 | 22.03 | 279.96 | 154.73 | 40.11 | 290.77 | 74.20 | 210.34 | 5.61 | 56.27 | 2323.60 | 228.85 | 465.28 | 16.45 | 78.33 | 413.18 | 224.09 | 509.07 | 503.10 | 2.03 | 276.77 | 5.56 | 0.00 | 4352.00 | 2.89 | 298.63 | 22.11 | 57.02 | 9.61 | 2.49 | 43.78 | 13.86 | 15.72 | 340.94 | 2910.23 | 3.21 | 4.01 | 8769.10 | 128.51 |
| 190207 | NorCOMM2 | Cdk4 | Cdk4_tm1b_F08 | +/- | M | 190207 | 11/25/2014 | 2429.22 | 3789.75 | 233.07 | 90.01 | 1218.93 | 26.57 | 44.83 | 1764.97 | 1763.04 | 2558.20 | 637.19 | 245.63 | 3622.65 | 235.85 | 14.09 | 62.98 | 26.05 | 10.68 | 174.22 | 23.60 | 41.86 | 1.26 | 32.39 | 1912.12 | 167.89 | 518.75 | 12.37 | 86.04 | 533.06 | 205.16 | 531.48 | 420.82 | 1.86 | 334.95 | 6.86 | 0.00 | 4882.40 | 6.61 | 289.93 | 23.74 | 56.19 | 11.33 | 2.75 | 44.91 | 21.21 | 19.33 | 343.60 | 2714.90 | 3.20 | 6.58 | 8021.21 | 154.65 |
| 190206 | NorCOMM2 | Cdk4 | Cdk4_tm1b_F08 | +/- | M | 190206 | 11/25/2014 | 1675.85 | 2955.78 | 301.53 | 99.94 | 1344.54 | 29.47 | 141.30 | 1132.01 | 5777.32 | 3538.46 | 638.41 | 149.64 | 6783.56 | 552.17 | 49.60 | 129.43 | 53.31 | 13.42 | 131.89 | 35.57 | 37.75 | 2.72 | 46.65 | 1996.04 | 444.27 | 423.60 | 14.63 | 56.63 | 648.31 | 179.72 | 717.04 | 515.95 | 2.04 | 286.13 | 7.35 | 0.00 | 5232.69 | 6.98 | 295.92 | 25.39 | 49.62 | 9.48 | 2.49 | 47.61 | 18.46 | 19.27 | 344.84 | 2514.04 | 4.11 | 6.02 | 7684.19 | 147.30 |
| 122069 | NorCOMM2 | Dhfr | Dhfr_tm1b_H07 | +/- | F | 122069 | 6/19/2014 | 8000.90 | 1331.50 | 256.75 | 80.59 | 715.71 | 17.67 | 26.70 | 4427.30 | 585.76 | 3897.70 | 1529.90 | 794.93 | 5797.30 | 360.17 | 44.44 | 99.93 | 57.40 | 8.45 | 93.94 | 51.43 | 57.69 | 2.90 | 48.09 | 3159.10 | 217.61 | 334.27 | 19.96 | 71.28 | 420.16 | 181.42 | 350.11 | 590.26 | 2.69 | 286.78 | 5.90 | 1.39 | 4747.00 | 4.31 | 241.97 | 30.50 | 79.21 | 6.15 | 3.02 | 57.34 | 13.66 | 13.07 | 242.45 | 4603.80 | 3.46 | 4.40 | 3088.70 | 89.46 |
| 122067 | NorCOMM2 | Dhfr | Dhfr_tm1b_H07 | +/- | F | 122067 | 6/19/2014 | 4510.70 | 813.26 | 227.56 | 81.93 | 720.40 | 16.26 | 34.29 | 4220.40 | 2210.90 | 4135.80 | 2704.60 | 716.36 | 5272.00 | 514.06 | 29.06 | 135.77 | 51.89 | 33.33 | 180.33 | 65.55 | 47.14 | 3.25 | 169.04 | 2360.80 | 190.11 | 346.62 | 17.41 | 80.05 | 427.76 | 194.91 | 386.56 | 478.97 | 2.54 | 308.95 | 6.68 | 0.00 | 4673.60 | 8.41 | 176.03 | 32.74 | 72.47 | 6.58 | 2.93 | 60.50 | 9.67 | 10.95 | 214.73 | 4133.10 | 3.89 | 5.54 | 4249.00 | 105.97 |
| 122066 | NorCOMM2 | Dhfr | Dhfr_tm1b_H07 | +/- | F | 122066 | 6/19/2014 | 1989.00 | 1786.70 | 183.70 | 75.52 | 842.92 | 17.69 | 50.80 | 4626.60 | 657.42 | 3011.00 | 1841.10 | 469.52 | 3170.20 | 619.79 | 27.98 | 90.26 | 43.72 | 29.81 | 83.35 | 50.17 | 48.32 | 5.25 | 43.17 | 1338.60 | 194.36 | 308.82 | 17.79 | 78.30 | 471.64 | 221.65 | 335.45 | 521.12 | 2.81 | 162.64 | 6.30 | 0.61 | 3996.00 | 4.89 | 302.15 | 24.16 | 75.65 | 7.65 | 2.71 | 52.27 | 13.08 | 17.91 | 202.11 | 4076.50 | 2.75 | 3.05 | 3467.80 | 88.11 |
| 121964 | NorCOMM2 | Dhfr | Dhfr_tm1b_H07 | +/- | M | 121964 | 6/19/2014 | 3560.50 | 1045.10 | 305.62 | 103.86 | 857.52 | 19.41 | 24.52 | 1252.80 | 1076.00 | 968.48 | 1384.20 | 245.62 | 2514.70 | 165.48 | 17.54 | 81.62 | 33.08 | 16.69 | 468.49 | 68.65 | 36.86 | 2.99 | 18.17 | 2336.70 | 186.60 | 425.79 | 16.25 | 65.23 | 361.82 | 234.86 | 398.66 | 501.22 | 2.28 | 377.16 | 4.89 | 0.00 | 5057.80 | 4.01 | 178.11 | 24.99 | 80.45 | 8.62 | 3.27 | 44.78 | 12.88 | 14.77 | 296.22 | 3135.20 | 4.97 | 5.02 | 8262.60 | 134.51 |
| 121962 | NorCOMM2 | Dhfr | Dhfr_tm1b_H07 | +/- | M | 121962 | 6/19/2014 | 1768.50 | 1213.90 | 309.05 | 79.85 | 966.90 | 16.62 | 31.85 | 2373.00 | 573.94 | 1025.60 | 1053.80 | 308.80 | 3002.40 | 126.41 | 17.44 | 85.67 | 66.28 | 8.58 | 54.72 | 11.87 | 49.25 | 1.66 | 21.68 | 2694.70 | 206.27 | 381.01 | 15.02 | 53.44 | 343.46 | 230.95 | 374.68 | 489.66 | 2.58 | 336.01 | 7.87 | 0.00 | 4589.80 | 5.42 | 231.46 | 24.97 | 74.69 | 9.05 | 2.66 | 60.21 | 12.91 | 15.86 | 340.85 | 3071.80 | 3.48 | 5.41 | 7522.00 | 126.96 |
| 129282 | NorCOMM2 | Dhfr | Dhfr_tm1b_H07 | +/- | M | 129282 | 7/9/2014 | 1632.90 | 1119.60 | 365.26 | 95.48 | 1114.80 | 19.67 | 78.97 | 3329.40 | 1363.60 | 1469.00 | 796.48 | 244.22 | 3860.20 | 194.35 | 15.99 | 130.20 | 46.00 | 10.41 | 136.05 | 27.26 | 35.78 | 1.86 | 28.60 | 3097.50 | 329.91 | 449.09 | 17.31 | 59.23 | 422.09 | 214.08 | 480.01 | 594.46 | 2.14 | 347.72 | 5.54 | 0.50 | 4313.60 | 4.22 | 259.85 | 18.63 | 69.82 | 11.19 | 3.02 | 39.88 | 17.43 | 17.25 | 299.16 | 2983.10 | 3.99 | 3.39 | 9137.90 | 121.72 |
| 428089 | K2P2 | Dync1li1 | Dync1li1_em1_del | +/- | F | 428089 | 8/18/2016 | 2684.80 | 1537.15 | 179.31 | 67.00 | 686.98 | 22.85 | 22.98 | 2252.05 | 4987.93 | 2805.11 | 2842.12 | 249.60 | 6054.18 | 399.24 | 28.93 | 251.58 | 140.46 | 17.07 | 154.42 | 44.51 | 103.75 | 2.22 | 67.12 | 3335.36 | 308.19 | 271.53 | 14.84 | 75.39 | 471.37 | 396.82 | 380.46 | 676.96 | 1.79 | 153.69 | 5.02 | 11.00 | 3710.47 | 3.08 | 272.15 | 25.32 | 63.07 | 6.14 | 2.94 | 59.66 | 12.04 | 9.05 | 148.29 | 4581.65 | 3.12 | 4.38 | 2232.32 | 107.53 |
| 420778 | K2P2 | Dync1li1 | Dync1li1_em1_del | +/- | F | 420778 | 8/5/2016 | 5639.47 | 1533.20 | 193.61 | 92.55 | 1002.47 | 18.93 | 22.37 | 3009.28 | 2726.23 | 5307.39 | 1630.66 | 189.52 | 6135.86 | 394.82 | 30.35 | 204.82 | 102.93 | 6.23 | 310.61 | 38.28 | 91.16 | 1.96 | 71.17 | 3381.40 | 313.14 | 340.61 | 17.68 | 77.86 | 431.82 | 297.63 | 316.82 | 553.63 | 1.73 | 135.14 | 5.89 | 4.19 | 4917.80 | 3.78 | 261.18 | 20.50 | 47.37 | 13.84 | 3.60 | 52.97 | 13.91 | 12.38 | 198.12 | 4056.49 | 3.19 | 3.89 | 2776.07 | 90.00 |
| 428090 | K2P2 | Dync1li1 | Dync1li1_em1_del | +/- | F | 428090 | 8/18/2016 | 1255.62 | 1611.16 | 222.95 | 111.74 | 987.12 | 28.30 | 25.03 | 2339.09 | 15506.96 | 5591.13 | 7953.95 | 906.45 | 15387.41 | 491.09 | 34.95 | 125.18 | 102.26 | 17.37 | 853.26 | 133.32 | 315.90 | 3.46 | 833.98 | 5557.05 | 444.41 | 331.39 | 17.25 | 119.32 | 513.00 | 300.94 | 340.92 | 649.54 | 1.79 | 203.66 | 6.10 | 0.80 | 4663.05 | 2.46 | 264.57 | 22.73 | 47.13 | 11.23 | 4.86 | 68.30 | 15.99 | 14.41 | 223.51 | 4584.39 | 2.71 | 4.01 | 3033.93 | 94.81 |
| 420676 | K2P2 | Dync1li1 | Dync1li1_em1_del | +/- | M | 420676 | 8/3/2016 | 9927.33 | 1807.13 | 252.18 | 88.90 | 1095.85 | 23.47 | 23.39 | 3454.63 | 4049.62 | 2261.10 | 686.46 | 184.73 | 5228.68 | 334.34 | 47.93 | 94.72 | 86.76 | 11.07 | 104.72 | 482.24 | 64.38 | 1.77 | 43.94 | 3465.50 | 213.43 | 417.61 | 11.49 | 53.68 | 340.47 | 293.81 | 382.97 | 367.46 | 1.52 | 287.96 | 6.84 | 0.00 | 3961.04 | 3.16 | 238.03 | 27.32 | 32.47 | 11.10 | 3.14 | 45.76 | 15.08 | 13.82 | 269.34 | 2727.28 | 3.04 | 5.14 | 7796.27 | 131.19 |
| 420674 | K2P2 | Dync1li1 | Dync1li1_em1_del | +/- | M | 420674 | 8/3/2016 | 1856.14 | 2615.85 | 261.27 | 75.10 | 1026.96 | 27.51 | 17.62 | 5093.54 | 4226.40 | 2351.30 | 1109.92 | 309.37 | 6810.52 | 310.51 | 42.79 | 93.35 | 50.65 | 29.79 | 95.86 | 61.91 | 192.77 | 3.20 | 54.88 | 5310.93 | 447.48 | 400.93 | 13.56 | 61.89 | 484.72 | 319.16 | 446.95 | 411.03 | 1.94 | 263.76 | 5.81 | 0.45 | 4787.36 | 3.87 | 321.84 | 27.31 | 47.97 | 11.00 | 3.80 | 63.77 | 13.97 | 13.44 | 267.29 | 3176.63 | 4.12 | 6.83 | 8963.88 | 155.81 |
| 420673 | K2P2 | Dync1li1 | Dync1li1_em1_del | +/- | M | 420673 | 8/3/2016 | 1961.05 | 1278.22 | 230.94 | 90.28 | 824.90 | 26.56 | 18.06 | 2906.68 | 656.28 | 654.49 | 952.47 | 285.38 | 3108.14 | 178.28 | 36.59 | 70.97 | 42.69 | 6.98 | 49.91 | 34.23 | 26.67 | 1.75 | 29.21 | 2943.37 | 186.04 | 354.90 | 14.29 | 68.01 | 352.71 | 212.17 | 312.28 | 409.54 | 1.99 | 332.38 | 5.57 | 0.00 | 4537.66 | 3.56 | 168.72 | 21.91 | 32.91 | 11.37 | 2.72 | 43.64 | 15.01 | 13.33 | 316.44 | 2725.35 | 3.57 | 1.97 | 6849.45 | 128.31 |
| 410978 | KOMP2 | G6pd2 | G6pd2_em1_del | -/- | F | 410978 | 6/16/2016 | 4099.49 | 1680.20 | 207.97 | 98.27 | 921.31 | 26.00 | 42.47 | 1281.30 | 1107.20 | 2508.54 | 1058.44 | 188.29 | 2565.71 | 334.37 | 81.13 | 116.67 | 34.14 | 3.87 | 111.76 | 67.34 | 37.59 | 1.27 | 24.66 | 1665.42 | 172.11 | 351.45 | 18.43 | 97.20 | 490.80 | 325.54 | 342.01 | 549.40 | 1.49 | 168.49 | 5.60 | 1.72 | 5559.68 | 4.95 | 263.38 | 18.13 | 37.63 | 11.50 | 4.14 | 58.15 | 13.76 | 12.79 | 177.50 | 3812.64 | 2.87 | 2.55 | 3720.94 | 69.29 |
| 410980 | KOMP2 | G6pd2 | G6pd2_em1_del | -/- | F | 410980 | 6/16/2016 | 2185.87 | 1722.17 | 212.19 | 88.35 | 853.36 | 19.87 | 27.23 | 2418.34 | 3017.68 | 3685.98 | 1768.14 | 365.02 | 6891.32 | 646.59 | 66.16 | 137.55 | 204.49 | 10.01 | 366.03 | 117.32 | 85.94 | 3.20 | 38.26 | 5064.37 | 442.42 | 310.20 | 17.98 | 95.34 | 495.01 | 300.14 | 399.70 | 681.03 | 1.66 | 148.87 | 7.11 | 1.58 | 4440.34 | 5.25 | 355.84 | 23.61 | 49.00 | 9.80 | 4.36 | 62.11 | 11.04 | 15.04 | 208.07 | 3940.24 | 3.17 | 5.36 | 3997.49 | 92.38 |
| 410975 | KOMP2 | G6pd2 | G6pd2_em1_del | -/- | F | 410975 | 6/16/2016 | 4946.79 | 1557.22 | 215.83 | 75.20 | 848.56 | 21.48 | 35.46 | 6142.13 | 1335.86 | 9163.73 | 1141.51 | 284.87 | 7728.67 | 302.82 | 21.90 | 394.42 | 65.51 | 16.76 | 86.06 | 64.15 | 50.17 | 1.73 | 23.72 | 3128.68 | 270.36 | 288.34 | 13.41 | 70.53 | 487.11 | 329.05 | 388.58 | 672.29 | 2.08 | 176.72 | 7.60 | 0.00 | 4338.85 | 5.56 | 205.85 | 29.13 | 44.72 | 7.19 | 2.91 | 56.84 | 12.28 | 13.05 | 181.79 | 3593.95 | 3.33 | 4.65 | 3335.44 | 80.93 |
| 410881 | KOMP2 | G6pd2 | G6pd2_em1_del | -/- | M | 410881 | 6/16/2016 | 6397.20 | 2271.05 | 268.41 | 70.20 | 920.14 | 26.02 | 41.23 | 2547.74 | 2241.35 | 2177.16 | 946.23 | 192.29 | 4613.87 | 233.62 | 12.64 | 127.23 | 243.43 | 8.23 | 260.52 | 30.29 | 35.99 | 1.21 | 43.60 | 2449.31 | 250.30 | 420.12 | 13.38 | 73.12 | 340.90 | 230.65 | 341.05 | 519.57 | 1.75 | 287.15 | 5.27 | 0.00 | 4320.59 | 5.32 | 336.17 | 21.42 | 41.66 | 11.49 | 2.58 | 47.01 | 16.26 | 14.48 | 301.16 | 2588.87 | 4.10 | 4.16 | 8239.70 | 113.96 |
| 420416 | KOMP2 | G6pd2 | G6pd2_em1_del | -/- | M | 420416 | 8/3/2016 | 3151.53 | 1212.42 | 281.38 | 79.46 | 886.84 | 25.18 | 21.10 | 754.14 | 3063.62 | 2142.25 | 962.63 | 231.06 | 6068.08 | 189.41 | 10.29 | 68.32 | 61.61 | 18.16 | 64.79 | 21.65 | 40.28 | 1.31 | 30.29 | 7440.61 | 546.23 | 373.94 | 16.19 | 72.81 | 354.07 | 285.46 | 331.20 | 433.74 | 1.77 | 331.44 | 5.03 | 4.18 | 4784.55 | 3.52 | 282.02 | 21.97 | 42.95 | 11.13 | 3.29 | 40.50 | 13.00 | 12.79 | 251.38 | 2704.12 | 3.48 | 2.40 | 7554.53 | 125.08 |
| 410882 | KOMP2 | G6pd2 | G6pd2_em1_del | -/- | M | 410882 | 6/16/2016 | 955.04 | 2640.08 | 183.82 | 64.51 | 835.53 | 29.24 | 37.01 | 1321.37 | 2031.87 | 1302.36 | 640.43 | 150.89 | 2573.55 | 152.32 | 8.95 | 137.98 | 40.25 | 6.80 | 73.53 | 27.86 | 30.57 | 2.30 | 34.32 | 1682.97 | 186.68 | 382.72 | 15.17 | 59.15 | 365.39 | 240.19 | 333.53 | 447.10 | 1.95 | 262.64 | 5.80 | 0.00 | 4756.77 | 4.47 | 317.02 | 21.47 | 52.77 | 9.38 | 3.71 | 48.87 | 14.81 | 15.76 | 310.25 | 2994.66 | 3.57 | 5.43 | 7008.93 | 131.18 |
| 39183 | NorCOMM2 | Galc | Galc_tm1b_A06 | +/- | F | 39183 | 12/4/2013 | 5352.36 | 1226.68 | 212.99 | 104.29 | 829.84 | 17.69 | 33.12 | 3092.11 | 5713.31 | 6266.90 | 4154.71 | 368.61 | 9955.34 | 701.45 | 38.67 | 218.92 | 77.62 | 26.90 | 211.94 | 53.16 | 118.79 | 7.03 | 212.56 | 3227.85 | 333.15 | 328.63 | 15.36 | 87.37 | 416.49 | 154.02 | 257.41 | 430.84 | 2.15 | 163.97 | 5.32 | 0.00 | 4166.17 | 4.39 | 199.59 | 21.61 | 47.25 | 8.67 | 2.89 | 46.67 | 12.16 | 14.07 | 196.10 | 4493.83 | 3.19 | 3.27 | 3089.07 | 83.53 |
| 40391 | NorCOMM2 | Galc | Galc_tm1b_A06 | +/- | F | 40391 | 12/16/2013 | 15181.95 | 1578.38 | 197.54 | 70.24 | 788.87 | 20.29 | 29.60 | 3322.82 | 2930.93 | 6621.21 | 2715.90 | 453.23 | 7561.57 | 381.27 | 16.72 | 285.33 | 43.16 | 26.05 | 86.11 | 19.77 | 180.11 | 4.05 | 31.32 | 3681.57 | 242.59 | 331.89 | 16.71 | 85.80 | 525.99 | 206.72 | 415.33 | 574.49 | 2.74 | 195.01 | 6.24 | 0.00 | 4452.88 | 2.96 | 293.63 | 26.17 | 65.91 | 10.09 | 3.84 | 70.32 | 16.95 | 14.04 | 209.33 | 4399.57 | 2.84 | 3.78 | 3934.88 | 79.84 |
| 39181 | NorCOMM2 | Galc | Galc_tm1b_A06 | +/- | F | 39181 | 12/4/2013 | 8646.66 | 1632.65 | 190.80 | 94.45 | 1033.45 | 20.06 | 33.11 | 3437.90 | 942.00 | 4376.55 | 2709.27 | 508.97 | 5468.86 | 354.04 | 20.94 | 305.67 | 54.04 | 15.49 | 113.26 | 21.90 | 61.92 | 1.62 | 77.01 | 3105.16 | 211.48 | 376.05 | 15.10 | 73.96 | 431.43 | 162.18 | 294.94 | 594.99 | 2.67 | 192.18 | 7.95 | 0.00 | 5282.43 | 6.26 | 140.96 | 30.36 | 60.93 | 8.97 | 3.85 | 67.06 | 13.24 | 11.74 | 225.29 | 3625.45 | 3.18 | 3.58 | 4078.55 | 94.75 |
| 43707 | NorCOMM2 | Galc | Galc_tm1b_A06 | +/- | M | 43707 | 12/30/2013 | 4014.01 | 2058.11 | 237.52 | 79.09 | 635.81 | 25.93 | 28.46 | 1984.82 | 3468.54 | 3195.94 | 1088.73 | 635.00 | 4437.93 | 233.25 | 92.11 | 123.08 | 41.52 | 22.58 | 163.20 | 237.24 | 65.07 | 3.56 | 102.25 | 2477.49 | 266.80 | 461.76 | 11.54 | 67.36 | 434.92 | 188.06 | 452.52 | 432.03 | 2.45 | 381.94 | 6.31 | 0.00 | 5211.53 | 5.71 | 222.99 | 28.38 | 47.71 | 7.73 | 2.86 | 49.34 | 16.91 | 13.83 | 252.16 | 2307.72 | 6.02 | 3.82 | 7766.35 | 144.71 |
| 40288 | NorCOMM2 | Galc | Galc_tm1b_A06 | +/- | M | 40288 | 12/16/2013 | 6492.95 | 1510.75 | 255.25 | 72.19 | 1100.75 | 21.17 | 24.40 | 2841.65 | 1326.97 | 3174.48 | 2218.65 | 942.51 | 7030.58 | 513.51 | 22.90 | 357.76 | 83.58 | 12.05 | 197.00 | 55.56 | 58.18 | 2.17 | 40.99 | 6689.12 | 421.47 | 368.79 | 15.77 | 85.32 | 421.41 | 231.13 | 453.99 | 585.80 | 2.35 | 357.68 | 6.85 | 0.00 | 4985.69 | 4.15 | 230.40 | 25.40 | 28.51 | 8.91 | 3.14 | 63.41 | 9.79 | 14.90 | 324.44 | 2321.68 | 4.57 | 4.18 | 8524.20 | 99.37 |
| 43708 | NorCOMM2 | Galc | Galc_tm1b_A06 | +/- | M | 43708 | 12/30/2013 | 2291.62 | 2018.04 | 305.87 | 86.60 | 913.32 | 23.53 | 46.89 | 2114.97 | 1883.66 | 4967.71 | 714.28 | 247.21 | 5995.20 | 284.71 | 25.05 | 101.89 | 52.32 | 25.87 | 61.69 | 89.89 | 62.52 | 1.78 | 110.81 | 2074.58 | 195.90 | 470.19 | 11.86 | 60.37 | 393.24 | 200.26 | 461.32 | 470.00 | 2.39 | 349.16 | 6.64 | 0.00 | 4633.19 | 6.39 | 366.06 | 33.54 | 60.27 | 12.39 | 3.06 | 47.93 | 14.87 | 12.54 | 317.76 | 2803.24 | 3.43 | 4.80 | 8535.50 | 145.27 |
| 38377 | NorCOMM2 | Gnpda1 | Gnpda1_tm1b_A05 | +/- | F | 38377 | 12/11/2013 | 5052.20 | 1436.00 | 239.25 | 103.38 | 728.54 | 17.64 | 31.28 | 2956.80 | 3252.10 | 3953.40 | 2983.60 | 518.15 | 7606.60 | 270.08 | 39.09 | 134.22 | 72.97 | 34.95 | 160.34 | 681.73 | 173.75 | 3.10 | 541.36 | 4001.80 | 317.59 | 333.40 | 18.94 | 71.79 | 441.70 | 178.19 | 329.05 | 429.55 | 2.83 | 195.85 | 8.58 | 0.62 | 4957.50 | 6.32 | 179.05 | 28.77 | 68.71 | 9.86 | 3.72 | 56.30 | 11.48 | 14.18 | 204.95 | 4389.80 | 2.23 | 6.29 | 3218.60 | 93.04 |
| 37870 | NorCOMM2 | Gnpda1 | Gnpda1_tm1b_A05 | +/- | F | 37870 | 11/28/2013 | 15806.00 | 1522.60 | 231.64 | 83.61 | 762.07 | 14.36 | 41.49 | 2860.90 | 1584.00 | 3580.50 | 1718.00 | 378.24 | 4705.50 | 646.77 | 18.09 | 95.03 | 40.96 | 16.93 | 90.02 | 181.32 | 51.59 | 3.77 | 95.25 | 1967.50 | 159.89 | 361.50 | 11.75 | 65.04 | 398.01 | 175.37 | 342.49 | 497.33 | 2.46 | 228.46 | 5.66 | 0.00 | 5671.30 | 6.59 | 202.20 | 24.75 | 57.86 | 7.60 | 3.26 | 50.53 | 11.75 | 13.43 | 187.02 | 4082.10 | 2.80 | 4.39 | 3605.60 | 89.88 |
| 37871 | NorCOMM2 | Gnpda1 | Gnpda1_tm1b_A05 | +/- | F | 37871 | 11/28/2013 | 7499.50 | 1472.20 | 250.52 | 77.85 | 724.90 | 20.68 | 58.84 | 1762.80 | 8513.70 | 4818.40 | 1072.20 | 226.89 | 9794.10 | 251.97 | 23.92 | 158.00 | 29.83 | 7.52 | 59.80 | 24.13 | 53.73 | 2.80 | 67.12 | 3232.80 | 296.27 | 301.97 | 11.94 | 77.64 | 446.91 | 158.53 | 370.23 | 633.51 | 2.30 | 198.26 | 6.94 | 0.00 | 5047.30 | 6.97 | 163.86 | 25.09 | 51.18 | 4.59 | 2.54 | 58.07 | 13.86 | 13.48 | 208.68 | 3058.40 | 3.38 | 3.39 | 3710.10 | 86.83 |
| 38275 | NorCOMM2 | Gnpda1 | Gnpda1_tm1b_A05 | +/- | M | 38275 | 12/11/2013 | 6915.50 | 4016.80 | 351.22 | 96.84 | 1191.20 | 22.65 | 37.08 | 5243.20 | 4112.00 | 6416.00 | 738.76 | 397.89 | 7983.90 | 369.41 | 35.57 | 144.44 | 53.84 | 126.37 | 227.17 | 169.89 | 370.86 | 4.32 | 51.63 | 4381.50 | 288.70 | 450.96 | 14.92 | 68.00 | 587.30 | 195.42 | 705.03 | 448.58 | 1.72 | 346.65 | 7.88 | 0.00 | 5123.10 | 5.73 | 295.86 | 30.93 | 52.96 | 7.70 | 1.76 | 53.48 | 19.37 | 16.88 | 331.93 | 3066.80 | 2.92 | 3.92 | 8424.30 | 137.08 |
| 37768 | NorCOMM2 | Gnpda1 | Gnpda1_tm1b_A05 | +/- | M | 37768 | 11/27/2013 | 7487.60 | 3681.10 | 419.11 | 90.17 | 940.78 | 24.58 | 53.79 | 1794.00 | 6610.70 | 4937.00 | 726.80 | 263.23 | 7814.20 | 371.55 | 50.13 | 130.37 | 101.16 | 19.17 | 166.57 | 28.11 | 105.12 | 4.90 | 219.59 | 4708.10 | 361.24 | 483.88 | 18.90 | 80.12 | 652.62 | 202.64 | 633.53 | 442.88 | 1.84 | 286.61 | 7.84 | 0.00 | 5160.80 | 6.78 | 375.03 | 23.79 | 71.95 | 10.66 | 3.05 | 54.04 | 17.98 | 17.22 | 313.20 | 3102.40 | 3.90 | 6.27 | 8546.00 | 130.25 |
| 37769 | NorCOMM2 | Gnpda1 | Gnpda1_tm1b_A05 | +/- | M | 37769 | 11/28/2013 | 18644.00 | 1772.80 | 307.02 | 87.84 | 1062.70 | 26.55 | 56.40 | 1307.90 | 4019.30 | 2238.20 | 1052.70 | 291.44 | 4556.50 | 279.46 | 24.10 | 78.62 | 35.45 | 16.08 | 62.89 | 24.78 | 74.92 | 2.22 | 44.12 | 2185.60 | 248.94 | 470.12 | 20.48 | 62.14 | 433.23 | 194.56 | 508.74 | 422.99 | 1.98 | 414.14 | 8.36 | 0.50 | 5623.40 | 6.21 | 360.76 | 35.27 | 61.30 | 8.87 | 3.11 | 60.89 | 13.85 | 14.73 | 375.08 | 2654.80 | 3.31 | 4.69 | 11205.00 | 121.89 |
| 116005 | NorCOMM2 | Idh1 | Idh1_tm1b_H02 | -/- | F | 116005 | 6/5/2014 | 10122.19 | 2026.21 | 166.45 | 88.76 | 671.42 | 22.32 | 53.97 | 6058.48 | 566.34 | 4521.97 | 1593.04 | 309.38 | 5846.32 | 195.20 | 26.79 | 217.98 | 77.89 | 10.23 | 127.08 | 22.85 | 56.25 | 2.44 | 51.76 | 1549.62 | 182.01 | 349.49 | 17.06 | 73.83 | 448.61 | 180.53 | 303.68 | 598.37 | 2.33 | 232.18 | 7.07 | 0.00 | 5609.05 | 5.00 | 311.58 | 23.01 | 64.63 | 5.81 | 3.46 | 55.36 | 15.33 | 12.99 | 261.35 | 4406.64 | 5.11 | 4.06 | 3412.71 | 89.34 |
| 116097 | NorCOMM2 | Idh1 | Idh1_tm1b_H02 | -/- | F | 116097 | 6/5/2014 | 4739.45 | 1682.57 | 182.15 | 92.10 | 1023.41 | 19.25 | 92.23 | 2094.18 | 1731.87 | 6358.93 | 2227.85 | 453.79 | 8620.25 | 432.20 | 28.60 | 214.07 | 46.58 | 41.72 | 756.31 | 71.55 | 73.21 | 4.35 | 66.08 | 3101.42 | 342.98 | 391.50 | 14.40 | 96.39 | 503.90 | 201.90 | 433.30 | 625.30 | 2.38 | 149.02 | 7.06 | 0.00 | 4810.79 | 6.18 | 382.71 | 23.62 | 57.00 | 8.61 | 3.66 | 48.34 | 15.03 | 13.23 | 270.18 | 4041.91 | 2.60 | 2.81 | 3680.02 | 96.51 |
| 116002 | NorCOMM2 | Idh1 | Idh1_tm1b_H02 | -/- | F | 116002 | 6/5/2014 | 4742.31 | 1745.53 | 221.17 | 91.91 | 957.30 | 20.86 | 62.60 | 1853.73 | 593.82 | 3169.91 | 784.66 | 253.46 | 3725.76 | 473.02 | 19.29 | 93.24 | 55.30 | 8.94 | 71.72 | 30.45 | 70.05 | 2.01 | 42.73 | 1104.01 | 171.23 | 327.00 | 17.97 | 83.20 | 436.45 | 191.43 | 376.42 | 501.88 | 2.67 | 214.88 | 7.27 | 0.00 | 5239.61 | 5.03 | 347.83 | 17.32 | 88.08 | 5.98 | 2.94 | 48.44 | 14.84 | 16.18 | 214.79 | 4154.43 | 2.49 | 4.03 | 4363.91 | 91.84 |
| 172743 | NorCOMM2 | Idh1 | Idh1_tm1b_H02 | -/- | M | 172743 | 10/16/2014 | 1145.60 | 1708.22 | 209.73 | 66.54 | 928.12 | 25.17 | 90.70 | 1878.54 | 4133.64 | 3888.60 | 1072.26 | 268.72 | 6904.79 | 985.46 | 23.38 | 147.06 | 59.34 | 19.24 | 196.21 | 96.28 | 93.85 | 1.55 | 154.32 | 3838.65 | 472.78 | 395.44 | 15.80 | 73.25 | 394.17 | 173.49 | 349.50 | 500.54 | 2.10 | 309.47 | 7.02 | 0.00 | 5168.50 | 5.30 | 632.80 | 24.00 | 60.13 | 10.50 | 2.91 | 57.61 | 16.99 | 15.27 | 305.11 | 2964.15 | 4.01 | 4.70 | 8693.90 | 99.67 |
| 172742 | NorCOMM2 | Idh1 | Idh1_tm1b_H02 | -/- | M | 172742 | 10/16/2014 | 3392.61 | 1954.45 | 325.29 | 78.87 | 1026.26 | 25.74 | 108.60 | 1069.28 | 2303.99 | 1746.55 | 813.56 | 360.73 | 3487.34 | 216.92 | 20.12 | 82.42 | 36.67 | 10.19 | 86.82 | 31.50 | 30.42 | 8.24 | 55.49 | 1468.34 | 352.90 | 421.27 | 16.36 | 72.56 | 402.95 | 158.30 | 366.53 | 362.46 | 1.73 | 289.35 | 7.02 | 0.00 | 4324.62 | 5.58 | 492.16 | 23.75 | 57.56 | 13.03 | 3.69 | 46.52 | 17.26 | 14.19 | 298.94 | 3023.52 | 3.57 | 5.12 | 9277.17 | 111.91 |
| 172741 | NorCOMM2 | Idh1 | Idh1_tm1b_H02 | -/- | M | 172741 | 10/16/2014 | 929.51 | 1418.75 | 273.99 | 81.99 | 1216.38 | 25.24 | 30.39 | 2231.63 | 1847.38 | 2219.14 | 1008.32 | 301.48 | 3574.92 | 231.75 | 23.01 | 80.99 | 49.96 | 26.88 | 98.98 | 28.80 | 34.19 | 1.40 | 49.71 | 2004.16 | 155.47 | 453.49 | 13.06 | 72.09 | 411.06 | 179.05 | 390.60 | 551.62 | 2.46 | 335.49 | 6.08 | 0.00 | 4914.59 | 4.96 | 338.69 | 20.70 | 61.50 | 10.77 | 3.33 | 54.93 | 15.18 | 13.73 | 293.45 | 2504.29 | 4.48 | 3.85 | 8539.03 | 109.77 |
| 435700 | KOMP2 | Iqgap1 | Iqgap1_tm1b_A11 | -/- | F | 435700 | 9/8/2016 | 5832.71 | 1760.09 | 216.90 | 88.02 | 771.73 | 25.76 | 20.95 | 651.34 | 778.15 | 1760.64 | 445.79 | 133.29 | 2148.07 | 220.70 | 17.48 | 54.24 | 29.18 | 10.10 | 104.92 | 14.88 | 24.52 | 2.41 | 111.38 | 1142.69 | 84.11 | 291.97 | 15.54 | 80.08 | 431.45 | 326.54 | 371.45 | 505.69 | 1.73 | 154.07 | 5.44 | 0.38 | 4372.65 | 2.15 | 400.57 | 22.73 | 36.76 | 12.26 | 3.80 | 50.26 | 14.73 | 12.41 | 253.43 | 4186.30 | 3.51 | 3.68 | 4239.74 | 85.76 |
| 506105 | KOMP2 | Iqgap1 | Iqgap1_tm1b_A11 | -/- | F | 506105 | 3/23/2017 | 11723.62 | 1123.72 | 193.81 | 83.30 | 1058.28 | 26.08 | 19.05 | 610.64 | 504.78 | 3079.41 | 1054.71 | 207.28 | 2954.07 | 311.72 | 16.19 | 64.72 | 25.30 | 15.09 | 244.37 | 9.86 | 23.03 | 1.13 | 45.68 | 788.47 | 86.02 | 266.63 | 10.74 | 58.92 | 400.51 | 232.46 | 347.52 | 589.91 | 1.77 | 221.00 | 3.98 | 3.77 | 3464.26 | 3.28 | 142.12 | 23.56 | 35.47 | 7.85 | 2.22 | 51.98 | 12.07 | 11.90 | 201.23 | 3244.10 | 2.25 | 2.95 | 3018.73 | 88.21 |
| 435799 | KOMP2 | Iqgap1 | Iqgap1_tm1b_A11 | -/- | F | 435799 | 9/8/2016 | 3451.33 | 1642.89 | 223.08 | 74.41 | 738.11 | 23.66 | 19.11 | 505.31 | 422.16 | 2486.36 | 791.08 | 190.03 | 2739.37 | 177.99 | 26.75 | 108.08 | 20.99 | 35.46 | 76.98 | 67.74 | 21.39 | 1.18 | 19.64 | 1061.89 | 105.96 | 356.69 | 15.04 | 89.46 | 458.03 | 241.16 | 351.25 | 538.44 | 2.24 | 190.29 | 5.87 | 0.62 | 4220.36 | 3.33 | 296.13 | 22.09 | 56.72 | 9.29 | 3.93 | 56.75 | 12.91 | 11.92 | 229.65 | 3931.09 | 3.45 | 3.30 | 3658.91 | 92.62 |
| 435695 | KOMP2 | Iqgap1 | Iqgap1_tm1b_A11 | -/- | M | 435695 | 9/8/2016 | 2418.87 | 1545.87 | 334.73 | 95.43 | 1110.06 | 29.65 | 24.77 | 526.22 | 408.57 | 982.06 | 899.10 | 125.15 | 2083.03 | 149.68 | 18.78 | 57.55 | 15.71 | 0.00 | 26.98 | 7.43 | 14.83 | 1.97 | 12.22 | 1135.21 | 98.42 | 397.57 | 14.77 | 60.63 | 474.50 | 304.06 | 450.92 | 549.15 | 1.66 | 345.98 | 5.49 | 19.89 | 4569.23 | 3.04 | 268.77 | 20.50 | 39.06 | 10.88 | 3.89 | 43.70 | 18.85 | 10.57 | 311.64 | 2695.05 | 4.83 | 3.07 | 8583.61 | 90.89 |
| 487462 | KOMP2 | Iqgap1 | Iqgap1_tm1b_A11 | -/- | M | 487462 | 1/4/2017 | 2180.17 | 1220.23 | 244.73 | 67.10 | 776.01 | 26.18 | 23.89 | 749.44 | 1121.70 | 1285.35 | 639.06 | 145.23 | 2077.05 | 167.41 | 14.06 | 71.01 | 25.69 | 10.61 | 88.56 | 14.28 | 21.49 | 1.25 | 15.17 | 1177.61 | 107.83 | 311.78 | 11.48 | 42.71 | 407.06 | 337.17 | 554.77 | 649.05 | 2.05 | 247.78 | 5.42 | 0.00 | 4988.31 | 4.15 | 333.83 | 30.84 | 63.67 | 7.96 | 3.60 | 60.97 | 9.03 | 6.73 | 222.12 | 2763.80 | 4.56 | 4.22 | 4643.22 | 115.13 |
| 435693 | KOMP2 | Iqgap1 | Iqgap1_tm1b_A11 | -/- | M | 435693 | 9/8/2016 | 3012.77 | 1445.40 | 275.39 | 74.65 | 955.07 | 27.22 | 18.60 | 272.60 | 161.19 | 1174.96 | 831.53 | 107.30 | 2643.77 | 55.45 | 16.02 | 51.38 | 24.04 | 3.70 | 41.83 | 10.85 | 23.83 | 1.44 | 14.07 | 3267.07 | 239.12 | 400.72 | 12.79 | 67.36 | 509.78 | 283.68 | 495.51 | 566.92 | 1.81 | 291.16 | 5.04 | 0.00 | 4199.87 | 4.01 | 291.88 | 31.55 | 57.23 | 7.92 | 3.16 | 39.70 | 10.80 | 12.32 | 308.54 | 1956.34 | 3.80 | 3.03 | 10196.18 | 87.19 |
| 107190 | NorCOMM2 | Lmbrd1 | Lmbrd1_tm1b_A10 | +/- | F | 107190 | 5/22/2014 | 9449.13 | 1311.90 | 209.97 | 84.36 | 825.28 | 21.36 | 29.43 | 1860.57 | 1401.61 | 3624.39 | 1430.47 | 1199.28 | 5432.31 | 394.08 | 26.47 | 158.85 | 53.31 | 12.49 | 123.88 | 95.61 | 135.39 | 3.15 | 76.87 | 4436.76 | 367.68 | 357.91 | 17.48 | 85.06 | 508.43 | 167.54 | 318.95 | 439.06 | 2.63 | 226.72 | 7.11 | 0.00 | 4879.89 | 4.36 | 228.12 | 26.07 | 77.00 | 5.92 | 3.58 | 65.77 | 13.04 | 13.07 | 217.10 | 4007.49 | 1.85 | 6.81 | 2235.27 | 120.68 |
| 111493 | NorCOMM2 | Lmbrd1 | Lmbrd1_tm1b_A10 | +/- | F | 111493 | 5/27/2014 | 5863.88 | 2302.01 | 242.73 | 65.21 | 754.26 | 21.93 | 27.52 | 4474.78 | 3083.66 | 3740.12 | 2540.06 | 913.19 | 8695.31 | 276.90 | 23.01 | 353.80 | 69.32 | 22.13 | 721.53 | 63.44 | 64.90 | 4.92 | 50.81 | 10620.54 | 701.18 | 341.42 | 18.92 | 77.28 | 503.21 | 209.06 | 500.45 | 565.23 | 1.99 | 163.29 | 6.07 | 0.00 | 4437.64 | 5.53 | 421.59 | 20.65 | 67.81 | 7.50 | 3.33 | 52.52 | 12.78 | 16.68 | 253.17 | 4729.31 | 3.89 | 4.86 | 4082.18 | 92.70 |
| 107189 | NorCOMM2 | Lmbrd1 | Lmbrd1_tm1b_A10 | +/- | F | 107189 | 5/21/2014 | 4336.99 | 1820.36 | 188.95 | 118.32 | 901.69 | 23.33 | 47.49 | 3786.58 | 1808.91 | 5072.26 | 1439.77 | 333.47 | 7584.97 | 351.73 | 30.46 | 123.85 | 55.23 | 22.32 | 84.16 | 51.92 | 70.86 | 2.65 | 48.89 | 3384.20 | 248.72 | 336.82 | 16.20 | 106.43 | 467.44 | 167.97 | 387.69 | 481.67 | 2.04 | 212.83 | 8.16 | 0.00 | 4752.23 | 4.27 | 274.93 | 24.18 | 44.05 | 3.71 | 2.95 | 68.69 | 14.62 | 12.86 | 310.67 | 4248.13 | 3.13 | 4.86 | 2898.10 | 107.40 |
| 72464 | NorCOMM2 | Lmbrd1 | Lmbrd1_tm1b_A10 | +/- | M | 72464 | 3/12/2014 | 6564.35 | 1799.69 | 218.28 | 72.74 | 919.81 | 18.50 | 121.42 | 2194.55 | 300.12 | 1076.36 | 546.73 | 219.40 | 2900.44 | 172.20 | 15.55 | 103.09 | 21.79 | 15.52 | 53.73 | 21.03 | 39.09 | 1.39 | 26.23 | 1734.33 | 328.83 | 412.44 | 15.72 | 61.75 | 360.43 | 161.86 | 347.56 | 428.02 | 1.89 | 302.05 | 6.65 | 0.00 | 4582.05 | 6.19 | 279.17 | 20.91 | 53.43 | 9.27 | 2.53 | 42.41 | 18.63 | 13.17 | 295.25 | 1823.58 | 4.16 | 2.75 | 7715.62 | 104.72 |
| 72463 | NorCOMM2 | Lmbrd1 | Lmbrd1_tm1b_A10 | +/- | M | 72463 | 3/12/2014 | 4058.21 | 5721.31 | 308.29 | 73.88 | 994.27 | 31.09 | 34.09 | 2152.98 | 1301.95 | 3695.17 | 687.40 | 191.81 | 3841.19 | 325.95 | 12.31 | 84.93 | 24.65 | 5.90 | 84.96 | 18.74 | 816.54 | 2.30 | 22.07 | 1604.98 | 156.14 | 470.67 | 14.85 | 81.56 | 724.40 | 153.44 | 512.93 | 454.46 | 1.65 | 267.11 | 8.49 | 0.00 | 5402.06 | 5.02 | 288.01 | 24.98 | 47.72 | 8.80 | 2.83 | 46.42 | 21.89 | 17.11 | 311.59 | 2952.58 | 3.92 | 5.52 | 5909.90 | 154.19 |
| 72460 | NorCOMM2 | Lmbrd1 | Lmbrd1_tm1b_A10 | +/- | M | 72460 | 3/12/2014 | 16332.88 | 1799.69 | 266.13 | 77.40 | 1029.99 | 21.08 | 54.04 | 2098.39 | 1742.09 | 1862.38 | 1235.37 | 166.30 | 4121.34 | 103.52 | 20.46 | 73.09 | 39.09 | 5.26 | 59.20 | 27.43 | 27.86 | 1.73 | 40.11 | 1880.01 | 210.42 | 420.43 | 15.29 | 69.49 | 417.73 | 165.72 | 396.16 | 478.53 | 2.69 | 318.12 | 7.02 | 0.00 | 4470.53 | 4.59 | 297.89 | 26.71 | 51.12 | 7.85 | 2.90 | 51.65 | 20.11 | 15.74 | 299.77 | 2642.15 | 2.54 | 2.80 | 7131.73 | 124.24 |
| 213490 | NorCOMM2 | Mfap4 | Mfap4_tm1b_C11 | -/- | F | 213490 | 1/20/2015 | 1374.25 | 2442.66 | 167.40 | 64.67 | 727.93 | 22.37 | 67.37 | 1787.63 | 1084.70 | 3245.67 | 7291.28 | 220.98 | 6519.36 | 435.54 | 14.51 | 889.55 | 72.42 | 13.77 | 144.58 | 59.83 | 72.28 | 1.67 | 88.50 | 2899.28 | 247.30 | 326.73 | 14.33 | 89.43 | 434.86 | 177.25 | 401.97 | 466.07 | 1.90 | 209.09 | 5.18 | 0.00 | 3942.14 | 5.41 | 307.68 | 22.63 | 50.54 | 6.27 | 3.27 | 37.33 | 17.78 | 13.14 | 246.06 | 3862.46 | 3.36 | 0.00 | 3434.91 | 84.88 |
| 213491 | NorCOMM2 | Mfap4 | Mfap4_tm1b_C11 | -/- | F | 213491 | 1/21/2015 | 804.51 | 1474.29 | 184.30 | 91.47 | 1081.58 | 22.11 | 53.71 | 1571.71 | 646.59 | 2789.56 | 811.63 | 224.02 | 3659.92 | 277.91 | 23.26 | 62.56 | 58.57 | 9.05 | 63.89 | 39.48 | 34.08 | 4.23 | 28.75 | 1659.17 | 144.08 | 382.50 | 21.77 | 106.05 | 500.51 | 226.58 | 352.72 | 569.22 | 1.90 | 272.08 | 5.89 | 0.00 | 5661.30 | 4.28 | 309.96 | 19.16 | 78.62 | 8.51 | 3.41 | 48.00 | 15.18 | 14.82 | 261.86 | 4696.52 | 3.32 | 0.00 | 4701.76 | 99.82 |
| 213482 | NorCOMM2 | Mfap4 | Mfap4_tm1b_C11 | -/- | F | 213482 | 1/20/2015 | 2022.96 | 2448.49 | 244.29 | 84.11 | 839.37 | 21.52 | 40.96 | 2835.18 | 387.35 | 2016.96 | 1241.47 | 190.95 | 3207.40 | 243.25 | 13.51 | 68.67 | 32.66 | 5.32 | 49.87 | 27.44 | 39.25 | 1.33 | 208.20 | 1119.79 | 117.54 | 365.75 | 19.16 | 89.88 | 445.84 | 210.64 | 347.96 | 454.72 | 2.14 | 186.64 | 6.29 | 0.00 | 4772.79 | 5.07 | 420.35 | 22.24 | 57.10 | 8.37 | 3.38 | 55.25 | 22.10 | 20.58 | 220.72 | 4060.65 | 3.20 | 0.00 | 3478.89 | 96.04 |
| 193352 | NorCOMM2 | Mfap4 | Mfap4_tm1b_C11 | -/- | M | 193352 | 12/11/2014 | 3809.40 | 2344.19 | 231.31 | 80.80 | 1008.17 | 26.96 | 35.10 | 3578.81 | 4344.85 | 4490.96 | 1036.19 | 783.28 | 8446.94 | 415.16 | 29.26 | 108.30 | 73.40 | 144.66 | 184.36 | 120.51 | 147.44 | 3.10 | 137.23 | 3329.28 | 319.68 | 462.10 | 14.21 | 78.74 | 497.71 | 167.80 | 508.38 | 395.30 | 2.41 | 307.78 | 7.67 | 0.00 | 5278.42 | 6.30 | 297.73 | 26.50 | 57.50 | 10.66 | 3.77 | 57.03 | 16.26 | 13.14 | 353.22 | 2417.00 | 4.30 | 0.00 | 9941.77 | 131.00 |
| 193349 | NorCOMM2 | Mfap4 | Mfap4_tm1b_C11 | -/- | M | 193349 | 12/11/2014 | 7524.48 | 2636.53 | 313.72 | 82.81 | 1074.35 | 24.24 | 91.72 | 4108.49 | 2796.25 | 3235.09 | 859.84 | 332.96 | 5096.37 | 277.33 | 38.90 | 137.48 | 78.13 | 42.60 | 190.20 | 59.29 | 100.44 | 5.61 | 42.78 | 2394.66 | 336.81 | 503.86 | 15.25 | 80.52 | 409.66 | 173.23 | 407.47 | 345.62 | 2.40 | 311.96 | 6.71 | 0.00 | 3732.95 | 5.13 | 392.23 | 31.98 | 50.39 | 6.30 | 2.86 | 62.30 | 14.85 | 14.50 | 292.77 | 3320.95 | 4.46 | 0.00 | 7976.61 | 144.65 |
| 193353 | NorCOMM2 | Mfap4 | Mfap4_tm1b_C11 | -/- | M | 193353 | 12/11/2014 | 2954.14 | 1526.01 | 223.25 | 110.58 | 956.52 | 22.19 | 95.34 | 2960.28 | 1095.33 | 4948.80 | 1060.39 | 323.34 | 6743.08 | 237.36 | 24.56 | 176.54 | 32.32 | 15.89 | 97.40 | 54.27 | 70.70 | 2.44 | 64.24 | 2973.62 | 401.90 | 474.60 | 13.90 | 64.09 | 438.38 | 200.38 | 387.65 | 417.57 | 2.45 | 382.96 | 6.44 | 0.00 | 4663.68 | 5.21 | 194.45 | 20.26 | 61.31 | 7.75 | 3.45 | 53.16 | 18.81 | 16.16 | 337.64 | 2521.69 | 3.95 | 0.00 | 7128.83 | 136.24 |
| 356364 | NorCOMM2 | Mmachc | Mmachc_tm1.1_A10 | +/- | F | 356364 | 11/17/2015 | 3105.14 | 1661.26 | 186.24 | 80.40 | 719.32 | 19.30 | 44.20 | 2714.31 | 962.26 | 3262.15 | 1310.57 | 311.34 | 4584.94 | 244.06 | 26.03 | 105.00 | 40.15 | 31.94 | 138.56 | 53.53 | 52.65 | 1.68 | 88.70 | 1687.45 | 163.80 | 314.13 | 13.81 | 78.00 | 453.05 | 210.29 | 429.65 | 559.68 | 2.24 | 237.69 | 5.49 | 0.00 | 4368.14 | 4.69 | 197.53 | 18.45 | 49.73 | 6.05 | 3.26 | 60.83 | 16.94 | 14.52 | 259.80 | 3193.60 | 2.80 | 3.16 | 3995.46 | 76.85 |
| 356363 | NorCOMM2 | Mmachc | Mmachc_tm1.1_A10 | +/- | F | 356363 | 11/17/2015 | 1421.01 | 1926.15 | 175.24 | 82.59 | 750.98 | 22.40 | 45.43 | 2702.88 | 1279.33 | 3461.78 | 1391.43 | 238.46 | 4749.96 | 350.27 | 39.48 | 123.36 | 66.59 | 19.50 | 87.31 | 21.20 | 52.12 | 2.07 | 76.88 | 1660.26 | 180.01 | 324.08 | 17.74 | 103.47 | 438.92 | 182.67 | 342.97 | 620.92 | 2.38 | 222.30 | 7.04 | 3.70 | 5270.49 | 4.21 | 253.97 | 21.62 | 57.03 | 9.14 | 3.39 | 52.80 | 18.06 | 13.71 | 256.38 | 3850.31 | 2.47 | 5.28 | 3584.38 | 79.03 |
| 356370 | NorCOMM2 | Mmachc | Mmachc_tm1.1_A10 | +/- | F | 356370 | 11/19/2015 | 1934.78 | 1508.42 | 216.06 | 85.51 | 856.89 | 22.57 | 32.86 | 1876.77 | 1995.72 | 3581.98 | 1520.31 | 346.15 | 5779.55 | 332.78 | 44.57 | 142.82 | 29.75 | 17.25 | 108.41 | 38.27 | 186.07 | 2.05 | 79.47 | 2094.01 | 233.53 | 387.20 | 15.49 | 78.76 | 447.99 | 210.65 | 315.14 | 524.08 | 2.07 | 222.54 | 5.61 | 10.14 | 4615.84 | 6.58 | 274.93 | 19.09 | 55.59 | 7.12 | 2.85 | 50.29 | 16.04 | 15.27 | 219.04 | 4758.76 | 2.28 | 3.46 | 2980.26 | 86.14 |
| 356155 | NorCOMM2 | Mmachc | Mmachc_tm1.1_A10 | +/- | M | 356155 | 11/17/2015 | 2476.17 | 6485.32 | 239.55 | 71.63 | 1219.04 | 26.26 | 136.85 | 2854.29 | 1338.34 | 4047.86 | 725.49 | 124.41 | 4676.66 | 220.73 | 24.15 | 147.36 | 35.39 | 38.26 | 91.62 | 25.18 | 459.73 | 2.22 | 33.05 | 1345.74 | 329.17 | 423.20 | 15.32 | 64.61 | 742.22 | 179.35 | 829.57 | 411.24 | 1.88 | 280.57 | 7.68 | 0.00 | 6077.27 | 4.97 | 412.99 | 24.46 | 32.05 | 9.39 | 3.63 | 56.04 | 19.02 | 21.30 | 395.44 | 2865.96 | 5.51 | 6.55 | 7651.62 | 148.15 |
| 356156 | NorCOMM2 | Mmachc | Mmachc_tm1.1_A10 | +/- | M | 356156 | 11/17/2015 | 1201.67 | 4358.84 | 265.78 | 52.75 | 1168.60 | 28.78 | 210.62 | 1702.80 | 2884.65 | 5091.68 | 558.97 | 151.81 | 5809.78 | 377.21 | 13.53 | 76.65 | 35.51 | 4.86 | 65.07 | 73.57 | 1197.14 | 2.11 | 30.75 | 1544.05 | 600.03 | 406.42 | 10.95 | 49.78 | 629.72 | 181.98 | 745.92 | 393.44 | 2.32 | 250.55 | 7.88 | 1.23 | 4543.64 | 6.08 | 352.46 | 26.97 | 60.17 | 8.31 | 3.73 | 55.16 | 22.31 | 15.95 | 346.17 | 2319.00 | 4.42 | 6.71 | 6756.12 | 150.14 |
| 356153 | NorCOMM2 | Mmachc | Mmachc_tm1.1_A10 | +/- | M | 356153 | 11/17/2015 | 1920.05 | 3527.63 | 299.92 | 85.19 | 1113.39 | 24.19 | 112.95 | 1420.21 | 1119.94 | 2585.15 | 681.32 | 169.82 | 4125.07 | 257.24 | 26.44 | 78.39 | 35.08 | 5.70 | 61.78 | 147.09 | 237.57 | 3.34 | 47.59 | 2063.26 | 330.94 | 526.79 | 16.94 | 76.86 | 549.62 | 212.53 | 509.69 | 497.77 | 2.08 | 301.79 | 7.53 | 0.00 | 5555.50 | 6.19 | 398.02 | 26.64 | 51.53 | 10.10 | 3.87 | 59.69 | 21.36 | 20.85 | 411.40 | 3384.33 | 4.96 | 8.07 | 8224.32 | 157.11 |
| 328106 | NorCOMM2 | Mvk | Mvk_em1_del | +/- | F | 328106 | 10/1/2015 | 1552.50 | 1393.20 | 231.76 | 97.75 | 790.98 | 21.33 | 34.43 | 7356.40 | 2229.20 | 3025.40 | 877.45 | 318.30 | 6970.10 | 385.62 | 22.09 | 106.06 | 72.66 | 13.87 | 197.08 | 73.84 | 102.06 | 2.70 | 79.48 | 1970.40 | 229.80 | 367.44 | 23.38 | 102.22 | 546.81 | 205.85 | 483.43 | 493.82 | 2.51 | 181.49 | 6.07 | 1.74 | 5009.90 | 5.47 | 257.66 | 27.21 | 64.35 | 7.90 | 4.38 | 55.03 | 12.76 | 14.69 | 229.92 | 4523.90 | 3.18 | 4.92 | 3059.60 | 99.30 |
| 328100 | NorCOMM2 | Mvk | Mvk_em1_del | +/- | F | 328100 | 10/1/2015 | 2265.20 | 1579.20 | 197.48 | 82.23 | 835.20 | 19.12 | 34.00 | 4157.90 | 3119.40 | 6217.80 | 1062.40 | 386.09 | 6898.40 | 190.10 | 38.48 | 138.59 | 219.04 | 7.04 | 897.89 | 1527.00 | 60.64 | 1.90 | 57.63 | 2426.00 | 200.04 | 349.91 | 18.67 | 74.29 | 418.02 | 173.06 | 415.99 | 518.38 | 1.83 | 146.92 | 6.36 | 0.00 | 5328.80 | 6.02 | 254.71 | 20.31 | 60.74 | 8.81 | 3.85 | 57.24 | 14.20 | 16.17 | 187.74 | 4433.40 | 3.51 | 3.73 | 3481.40 | 85.27 |
[truncated: 198,643 more chars]
