# Supplementary material for: Proteotyping of knockout mouse strains reveals sex- and strain-specific signatures in blood plasma
Source: NPJ Syst Biol Appl. 2021 May 28;7:25. doi: 10.1038/s41540-021-00184-8 (PMC8163790; doi:10.1038/s41540-021-00184-8)
Supplement: Supplementary file 3 — Reporting Summary [file 41540_2021_184_MOESM3_ESM.pdf]

nature research

Corresponding author(s): Yassene Mohammed

Last updated by author(s): Apr 19, 2021

Reporting Summary

Nature Research wishes to improve the reproducibility of the work that we publish. This form provides structure for consistency and transparency in reporting. For further information on Nature Research policies, see our Editorial Policies and the Editorial Policy Checklist.

Statistics

For all statistical analyses, confirm that the following items are present in the figure legend, table legend, main text, or Methods section.

n/a	Confirmed	
☐	☒	The exact sample size (*n*) for each experimental group/condition, given as a discrete number and unit of measurement
☐	☒	A statement on whether measurements were taken from distinct samples or whether the same sample was measured repeatedly
☐	☒	The statistical test(s) used AND whether they are one- or two-sided<br>*Only common tests should be described solely by name; describe more complex techniques in the Methods section.*
☐	☒	A description of all covariates tested
☐	☒	A description of any assumptions or corrections, such as tests of normality and adjustment for multiple comparisons
☐	☒	A full description of the statistical parameters including central tendency (e.g. means) or other basic estimates (e.g. regression coefficient) AND variation (e.g. standard deviation) or associated estimates of uncertainty (e.g. confidence intervals)
☒	☐	For null hypothesis testing, the test statistic (e.g. *F*, *t*, *r*) with confidence intervals, effect sizes, degrees of freedom and *P* value noted<br>*Give P values as exact values whenever suitable.*
☒	☐	For Bayesian analysis, information on the choice of priors and Markov chain Monte Carlo settings
☒	☐	For hierarchical and complex designs, identification of the appropriate level for tests and full reporting of outcomes
☒	☐	Estimates of effect sizes (e.g. Cohen's *d*, Pearson's *r*), indicating how they were calculated

Our web collection on statistics for biologists contains articles on many of the points above.

Software and code

Policy information about availability of computer code

Data collection	Raw data was processed using Skyline version 4.1.1.18179, including inspection and correction of peak integration. Endogenous analyte concentrations were calculated from the endogenous/heavy ratio using regression analysis of the standard curves (1/x2 weighting).
Data analysis	All data analysis and visualization were performed using R 3.5.3 and 3.6.3, and its libraries including ggplot for visualization, glmnet for regression and statistical analysis, and ClusterProfiler for over representation analysis. p-values from Mann-Whitney-Wilcoxon tests used to compare protein abundances between knockouts and wild-type were adjusted using Benjamini-Hochberg method for multiple testing. Least absolute shrinkage and selection operator (LASSO) was used for identifying the minimal set of best discriminators between KO and wildtype mice that allow best discrimination. Mann-Whitney-Wilcoxon test used to compare protein abundances between KO and wildtype mice and p-values were adjusted with the Benjamini-Hochberg method for multiple testing. Protein fold changes were determined by calculating the ratio of mean concentrations of KO to wildtype mice. Volcano plots were used to represent p-values and fold change. Over representation analyses (ORA) and the required hypergeometric test were performed using the quantified proteins as a background. Entries in seven knowledgebases were used for ORA including Gene Ontology – GO, Molecular Signatures database – MsigDB, molecular pathway using Kyoto Encyclopedia of Genes and Genomes – KEGG and Reactome, Disease Ontology – DO, diseases and their gene associations using DisGeNET, and Medical Subject Headings – MeSH for processes and diseases.

For manuscripts utilizing custom algorithms or software that are central to the research but not yet described in published literature, software must be made available to editors and reviewers. We strongly encourage code deposition in a community repository (e.g. GitHub). See the Nature Research guidelines for submitting code & software for further information.

Data

Policy information about availability of data

All manuscripts must include a data availability statement. This statement should provide the following information, where applicable:

- Accession codes, unique identifiers, or web links for publicly available datasets
- A list of figures that have associated raw data
- A description of any restrictions on data availability

All protein concentration measurements are available in the supplementary material file "protein_concentrations_y.xlsx"

Field-specific reporting

Please select the one below that is the best fit for your research. If you are not sure, read the appropriate sections before making your selection.

☒ Life sciences ☐ Behavioural & social sciences ☐ Ecological, evolutionary & environmental sciences

For a reference copy of the document with all sections, see nature.com/documents/nr-reporting-summary-flat.pdf

Life sciences study design

All studies must disclose on these points even when the disclosure is negative.

Sample size	Plasma samples for 30 knockout mouse strains (Table 1 in the manuscript) were obtained from The Centre for Phenogenomics, which is part of the International Mouse Phenotyping Consortium (IMPC). Samples were collected from three male and three female mice of each knockout line, as well as 19 female and 19 male C57BL/6NCrl wildtype mice were collected at a similar time. The mass spectrometry method we used (targeted quantification with internal standard) is proven highest in precision and best reproducibility, which reduced technical variability to determined CV below 10% in our current work. Our main group comparisons of differentially abundant proteins were based on a change of twofold in the protein concentration associated with non-parametric Wilcoxon Rank-Sum test p-value threshold of 0.05. With low technical variability and high fold change we believe that the sample number is sufficient. Given the test used, fold change threshold, significance threshold, sample numbers, and determined CV values of the quantified proteins in controls (with a median of 0.248), we calculated a power of 0.97 for the two sided test.
Data exclusions	Proteins with failed quantification were removed.
Replication	All mutant mouse lines used for plasma proteotyping are available from the Canadian Mouse Mutant Repository (CMMR) at The Centre for Phenogenomics.
Randomization	Mouse plasma samples were processed using the Tecan Evo (Männedorf, Switzerland) liquid handling robot and all 218 samples were randomized over three 96 well plates. A pooled reference plasma sample (BioReclamationIVT; Westbury, NY, USA) was used for quality control and normalization with 9 to 12 reference samples per plate inserted semi-randomly. Additional 8 samples for establishing the standard curve were included on the first plate, and 3 curve quality control samples were included on each plate.
Blinding	Samples preparation was automated using a liquid handling system. Sample plate placement was randomized and therefore subsequent sample measurement and data collection. Initial data processing was performed based on the random sample collection order with no additional meta-data about the groups. Blinding was lifted during further data analysis of groups and sex comparisons.

Reporting for specific materials, systems and methods

We require information from authors about some types of materials, experimental systems and methods used in many studies. Here, indicate whether each material, system or method listed is relevant to your study. If you are not sure if a list item applies to your research, read the appropriate section before selecting a response.

Materials & experimental systems

n/a	Involved in the study	
☒	☐	Antibodies
☒	☐	Eukaryotic cell lines
☒	☐	Palaeontology and archaeology
☐	☒	Animals and other organisms
☒	☐	Human research participants
☒	☐	Clinical data
☒	☐	Dual use research of concern

Methods

n/a	Involved in the study	
☒	☐	ChIP-seq
☒	☐	Flow cytometry
☒	☐	MRI-based neuroimaging

Animals and other organisms

Policy information about studies involving animals; ARRIVE guidelines recommended for reporting animal research

Laboratory animals	Samples from 90 female and 90 male mice for 30 knockout strains and 38 corresponding wildtype controls were analyzed. All KO strains and controls were on the C57BL/6N genetic background. The knockout strains included A2m^tm1b(NCOM)Mfgc, Ahcy^tm1b(EUCOMM)Hmgu, Atp5b^tm1b(EUCOMM)Hmgu, Atp6v0d1^tm1b(EUCOMM)Hmgu, C8a^tm1b(EUCOMM)Hmgu, Cdk4^tm1b(NCOM)Mfgc, Dhfr^tm1b(EUCOMM)Wtsi, Dync1li1^em1(IMPC)Tcp, G6pd2^em1(IMPC)Tcp, Galc^tm1b(KOMP)Wtsi, Gnpda1^tm1b(KOMP)Mbp, Idh1^tm1b(EUCOMM)Wtsi, Iqgap1^tm1b(EUCOMM)Wtsi, Lmbrd1^tm1b(EUCOMM)Hmgu, Mfap4^tm1b(NCOM)Mfgc, Mmachc^tm1.1(NCOM)Mfgc, Mvk^em1(IMPC)Tcp, Nek2^em1(IMPC)Tcp, Npc2^tm1e(EUCOMM)Wtsi, Pebp1^em1(IMPC)Tcp, Phyh^tm1c(EUCOMM)Wtsi, Pipox^tm1b(EUCOMM)Wtsi, Plk1^tm1b(EUCOMM)Hmgu, Pmm2^tm1b(EUCOMM)Hmgu, Ptpn12^tm1b(NCOM)Mfgc, Pttg1^tm1b(EUCOMM)Wtsi, Rock1^tm1b(NCOM)Mfgc, Sra1^tm1b(EUCOMM)Hmgu, Ulk3^em2(IMPC)Tcp, Ywhaz^tm1b(EUCOMM)Hmgu
Wild animals	The study did not involve wild animals
Field-collected samples	The study did not involve field-collected samples
Ethics oversight	All experimental procedures on animals received approval from the Animal Care Committee of The Centre for Phenogenomics and were conducted in accordance with the guidelines of the Canadian Council on Animal Care. The corresponding license numbers are AUPs 153, 275, 277, and 279. All mutant mouse lines used for plasma proteotyping are available from the Canadian Mouse Mutant Repository (CMMR) at The Centre for Phenogenomics.

Note that full information on the approval of the study protocol must also be provided in the manuscript.
